# High-valent sulfur fluorides as reactivity switches for PFAS-free benzene–azepine skeletal editing

**DOI:** 10.1039/d5sc08177g

**Published:** 2025-11-19

**Authors:** Chavakula Nagababu, Takuya Muramatsu, Muhamad Zulfaqar Bacho, Shiwei Wu, Seishu Ochiai, Jorge Escorihuela, Norio Shibata

**Affiliations:** a Department of Nanopharmaceutical Sciences, Nagoya Institute of Technology Gokiso, Showa-ku Nagoya 466-8555 Japan nozshiba@nitech.ac.jp; b Department of Engineering, Nagoya Institute of Technology Gokiso, Showa-ku Nagoya 466-8555 Japan; c Departamento de Química Orgánica, Universitat de València Avda. Vicente Andrés Estellés s/n Burjassot 46100 Valencia Spain; d Instituto de Ciencia Molecular (ICMol), Universitat de València Calle Catedrático José Beltrán 2 Paterna Valencia Spain

## Abstract

The development of non-PFAS fluorinated heterocycles is a critical challenge in sustainable molecular design. Here we report a reactivity-switching skeletal editing strategy that transforms benzenes into azepines through visible-light activation mediated by high-valent sulfur fluorides. The pentafluorosulfanyl (SF_5_) and tetrafluorosulfanyl (SF_4_) groups act as powerful electronic activators that stabilize singlet nitrenes, lowering the barrier for 6π-electrocyclization and enabling efficient benzene-to-azepine interconversion in high yields. Mechanistic and DFT studies reveal that the strong electron-withdrawing capacity of SF_5_/SF_4_ is key to promoting this transformation. Importantly, SF_5_ and SF_4_ motifs are PFAS-free fluorine architectures that retain the beneficial physicochemical properties of perfluoroalkyl groups while avoiding environmental persistence. This work establishes a sustainable and general strategy for fluorinated skeletal editing, offering a foundation for future design of PFAS-free agrochemical, medicinal, and material scaffolds.

## Abstract 2

SF_5_- and SF_4_-functionalized azepines were efficiently accessed *via* a PFAS-free benzene–azepine skeletal editing platform.

## Graphical abstract

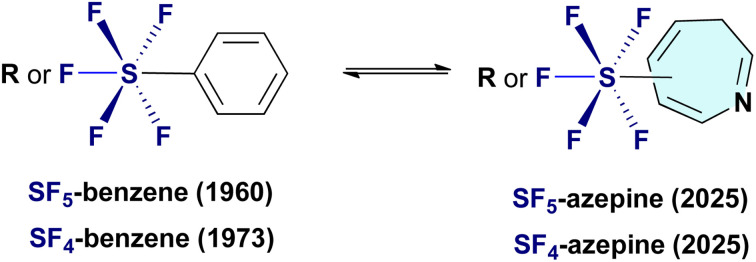

## Introduction

**Fig. 1:**
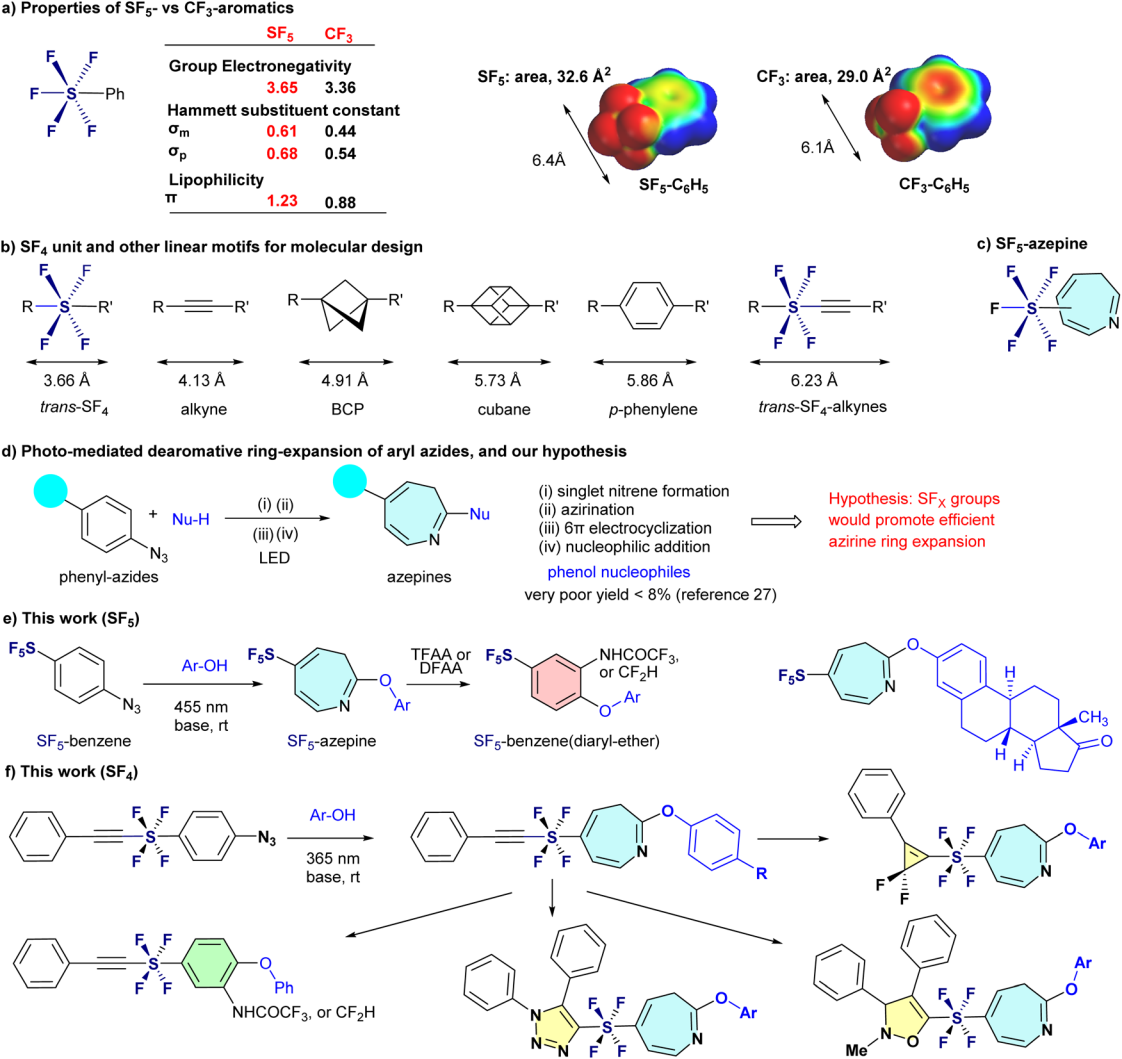
(a) Properties of SF_5_- *vs.* CF_3_-aromatics. (b) SF_4_ unit and other linear motifs for molecular design. (c) SF_5_-azepine. (d) Photo-mediated dearomative ring-expansion of aryl azides, and our hypothesis. (e) This work (SF_5_). (f) This work (SF_4_).

Fluorinated substituents are indispensable in the molecular design of pharmaceuticals and agrochemicals, where subtle modifications in lipophilicity, metabolic stability, and target engagement can critically influence whether a lead compound progresses to a viable product.^1–3^ Among these, the trifluoromethyl (CF_3_) group has long been a widely adopted motif. However, growing concerns have emerged regarding its environmentally persistent degradation product, trifluoroacetic acid (TFA), which is now regulated as part of the PFAS (per- and polyfluoroalkyl substances) class.^4,5^ This issue is particularly pressing in sectors with unavoidable environmental release, such as agrochemicals, outdoor coatings, and high-volume polymers, where new fluorinated motifs are urgently needed that retain the performance advantages of CF_3_ while avoiding its long-term ecological impact. The challenge is especially acute in agrochemistry, where over 40% of fluorinated agrochemicals incorporate CF_3_.^3^ These compounds are vital to ensuring global food security in the face of a rapidly growing population. The high-valent sulfur fluorides, such as pentafluorosulfanyl (SF_5_)^6,7^ and *trans*-tetrafluorosulfanyl (*trans*-SF_4_)^8–10^ groups directly address this challenge. The SF_5_ group is more electronegative than CF_3_, nearly twice more lipophilic, and adopts a compact “umbrella-like” geometry. Importantly, the S–F bonds in SF_5_ are significantly weaker than C–F bonds, offering potential for improved environmental degradability (Fig. 1a). Seminal contributions by Sheppard,^11,12^ and the development of Umemoto's oxidative protocol^13^ have made aryl-SF_5_ compounds synthetically accessible, enabling incorporation into structures such as pyridines^14,15^ and five-membered heterocycles^16^—key motifs in agrochemicals. However, SF_5_-containing seven-membered heterocycles remain entirely unexplored.

A structurally complementary but similarly underutilized motif is the *trans*-SF_4_ group. In contrast to SF_5_, which functions as a terminal substituent analogous to CF_3_, the SF_4_ moiety serves as a linear linker, connecting two carbon units across a C–SF_4_–C axis measuring just 3.66 Å. This makes *trans*-SF_4_ the shortest member of the “linear bioisostere” family, which includes alkynes (4.13 Å), bicyclo[1.1.1]pentane (4.91 Å), cubane (5.73 Å), and *para*-substituted phenylene (5.86 Å).^8–10^ When combined with an alkyne, the *trans*-SF_4_ group forms the longest fully linear connector known to date—6.23 Å—providing medicinal chemists with an exceptional rod-like linker for fragment-based design and spatial tuning (Fig. 1b).

The azepine ring—a seven-membered nitrogen-containing heterocycle—is a privileged scaffold found in numerous antiviral, anticancer, and neuroactive agents.^17,18^ One of the most conceptually elegant strategies for its construction is photoinduced dearomative coupling (PDC) of aryl azides, a transformation that effectively repurposes benzene rings into azepine frameworks.^19–26^ While PDC has been widely investigated, its synthetic utility remains limited by generally moderate yields and poor compatibility with phenolic nucleophiles. In fact, the only reported example involving a phenol—Takeuchi's 1982 study—afforded the desired diaryl ether azepine in a mere 7.7% yield (Fig. 1d).^27^

The PDC sequence involves four mechanistic steps: photoactivation of the aryl azide to generate a singlet nitrene, intramolecular insertion to form an azirine intermediate, electrocyclic ring-opening to a conjugated ketenimine, and final nucleophilic trapping to yield the azepine product. Since the SF_5_ group dramatically alters the physiochemical properties of the connected benzene ring,^6,7,11,12^ we hypothesized that the SF_5_ group would accelerate nitrene formation, promote efficient azirine ring expansion, and significantly enhance the electrophilicity of the ketenimine intermediate. Together, these effects would break through the long-standing yield barrier and unlock phenol-compatible routes to azepine architectures. We report herein a visible-light-driven (blue LED, 455 nm), base-mediated protocol that transforms SF_5_-phenyl azides into SF_5_-azepine diaryl ethers in isolated yields of up to 83%—approximately twice the efficiency achieved with non-SF_5_ analogues. These azepine products undergo efficient skeletal contraction back to SF_5_-aryl amines in yields exceeding 90% upon treatment with acid anhydrides, enabling a reversible and programmable interconversion between benzene and azepine frameworks with broad functional group tolerance (Fig. 1e). This concept also extends to *trans*-SF_4_ alkynyl azides, which, under 365 nm irradiation, furnish the first azepine derivatives incorporating this uniquely linear, rod-like spacer. Beyond their skeletal reversion, *trans*-SF_4_ alkynyl azepines retain the synthetic versatility of alkyne functionality, offering broad opportunities for downstream chemical transformations (Fig. 1f). These findings establish high-valent sulfur fluorides not simply as PFAS-safe sustainable CF_3_ alternatives, but as reactivity-switching motifs for skeletal editing—unlocking novel fluorinated architectures for next-generation drugs and materials.

## Results and discussion

**Scheme 1:**
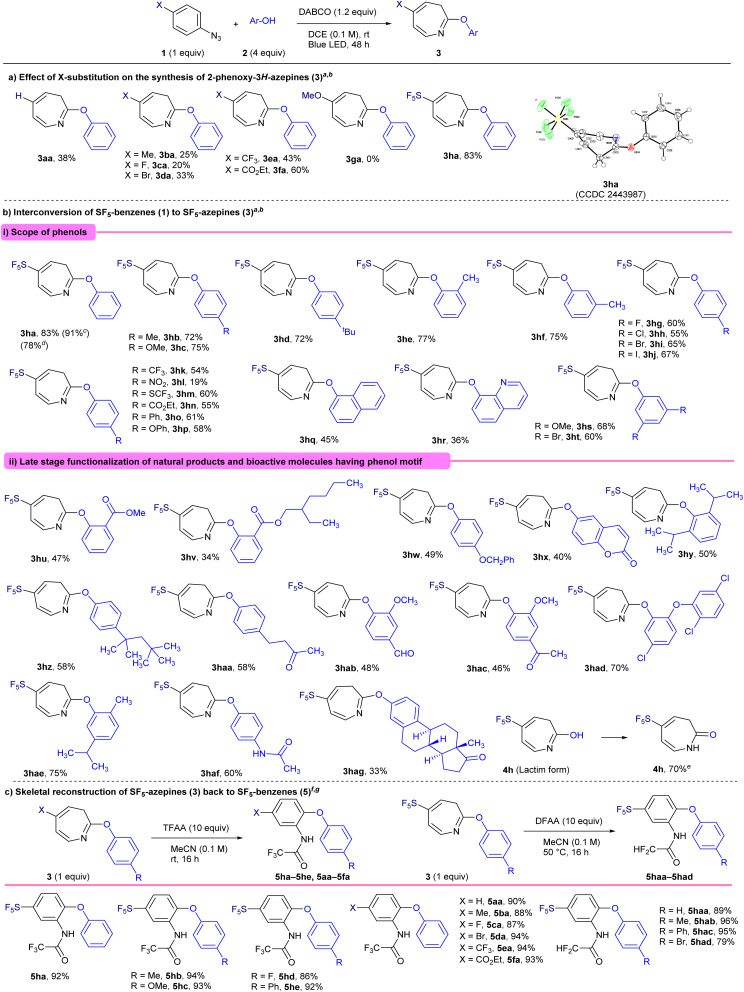
Substrate scope for azepine formation and its reversion to benzenes. ^*a*^Unless otherwise noted, the reactions were carried out with 1 (0.2 mmol), 2 (0.8 mmol), and DABCO (0.24 mmol) in DCE (0.1 M) irradiated under blue LED light at rt for 48 h. ^*b*^Isolated yields of 3. ^*c*^Yield of 3 determined by ^19^F NMR spectroscopy using C_6_H_5_F as the internal standard. ^*d*^Isolated yield of the gram-scale reaction. ^*e*^Reaction was carried out in THF/H_2_O (1/1) in the absence of 2. ^*f*^Unless otherwise noted, the reactions were carried out with 3 (0.1 mmol), TFAA (1 mmol) in MeCN (0.1 M) stirred at rt for 16 h or carried out with 3 (0.1 mmol), DFAA (1 mmol) in MeCN (0.1 M) stirred at 50 °C for 16 h. ^*g*^Isolated yields of 5.

We began our investigation by revisiting the photoinduced dearomative coupling reaction of phenyl azide (1a) with phenol (2a) (Table 1), originally examined by Takeuchi *et al.* in 1982, which yielded azepine-phenyl-ether (3aa) in a modest yield (7.7%)^27^ (entry 1). Their protocol was employed in a quartz tube with a 300 W high-pressure mercury lamp emitting aggressive short-wavelength radiation; thus, we aimed to improve this method by utilizing a milder and more environmentally friendly blue LED (40 W, 455 nm) source. Initially, the reaction did not proceed in EtOH under blue LED irradiation for 24 h (entry 2). To enhance the nucleophilicity of phenol, we subsequently added K_2_CO_3_ and changed the solvent from EtOH to DCE to avoid the competitive insertion of ethanol. The observed yield of 3aa was 27%. We further screened various bases, including Na_2_CO_3_, KOH, Et_3_N, DBU, and DABCO, and found that DABCO was the optimal choice, yielding desired product 3aa in 38% yield. Changing the irradiation source from a standard blue LED (40 W) to a 365 nm LED reduced the yield to 34%. Control experiments confirmed that LED irradiation is essential for the reaction to proceed. Although the maximum yield of 38% represents a substantial improvement over the original value (7.7%), opportunities remain for further optimization. Given the scarcity of the literature on the synthesis of substituted 2-phenoxy-3*H*-azepines, we extended our methodology to a diverse range of substituted aryl azides 1 (Scheme 1a). Methyl-substituted azide 1b afforded the corresponding product, 3ba, in 25% yield. The halogen-substituted derivatives fluoro-(1c) and bromo-(1d) were well tolerated, yielding 3ca and 3da in 20% and 33% yields, respectively. Electron-withdrawing substituents such as CF_3_ (1e) and CO_2_Et (1f) significantly improved the reaction outcomes, affording products (3ea and 3fa) in 43% and 60% yields, respectively. In contrast, MeO-substituted azide 1g failed to react under the tested conditions, providing no detectable product 3ga. Notably, SF_5_-substituted phenyl azide (1h) produced the desired SF_5_-azepine-phenyl-ether 3ha in 83% yield, highlighting the unique enhanced effect of the SF_5_-group in this synthetic approach. The structure of 3ha was confirmed using X-ray crystallography (CCDC 2443987).

**Table 1:** Optimization of the reaction conditions^a^

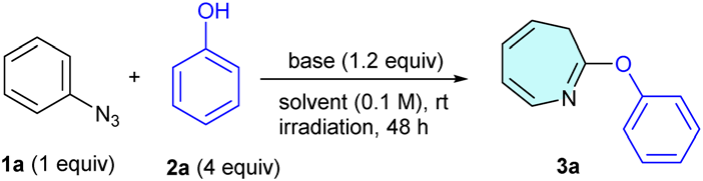				
Entry	Irradiation	Solvent	Base	Yield^b^ (%)
1	UV	EtOH	—	7.7 (ref. 27)
2	Blue LED (40 W)	EtOH	—	0
3	Blue LED (40 W)	DCE	K_2_CO_3_	27
4	Blue LED (40 W)	DCE	Na_2_CO_3_	12
5	Blue LED (40 W)	DCE	KOH	10
6	Blue LED (40 W)	DCE	Et_3_N	8
7	Blue LED (40 W)	DCE	DBU	24
8	Blue LED (40 W)	DCE	DABCO	38
9	365 nm LED (30 W)	DCE	DABCO	34
10	—	DCE	DABCO	0

a Unless otherwise noted, the reactions were carried out with 1a (0.2 mmol), 2a (0.8 mmol), and DABCO (0.24 mmol) in DCE (0.1 M) irradiated under blue LED light (about 455 nm) at rt for 48 h.

b Isolated yields of 3a.

Encouraged by the exceptional performance of the SF_5_ group rather than the others in phenyl under photo-induced dearomative coupling reactions with phenol 2a, we systematically explored the reaction scope using SF_5_-phenyl azide 1h with various phenols 2 featuring diverse electronic properties (Scheme 1b-i). Phenol (2a) initially yielded SF_5_-azepine 3ha in an excellent yield of 83%, which was maintained at 78% upon scaling the reaction to the gram scale. Phenols 2 bearing electron-donating substituents, such as 4-Me (2b), 4-MeO (2c), 4-*tert*-Bu (2d), 2-Me (2e), and 3-Me (2f), were effectively incorporated, affording the corresponding products (3hb–3hf) in yields ranging from 72% to 77%. Halogen-substituted phenols, including 4-F (2g), 4-Cl (2h), 4-Br (2i), and 4-I (2j), also reacted efficiently under the optimized conditions, producing azepines 3hg–3hj with yields of up to 67%, without any loss of halogen, especially the iodo-group.^28^ Thus, phenols containing electron-donating substituents demonstrated enhanced efficiency in the PDC reaction. Phenols bearing electron-withdrawing substituents, such as 4-CF_3_ (2k), 4-NO_2_ (2l), 4-SCF_3_ (2m), 4-CO_2_Et (2n), and 4-phenyl (2o), also reacted effectively, yielding the corresponding azepines (3hk–3ho) in good yields of up to 61%, except for 4-NO_2_-phenol (2l). Moreover, structurally more complex phenols, including 4-phenoxyphenol (2p), naphthol (2q), and 8-hydroxyquinoline (2r), provided the desired products (3hp, 3hq, and 3hr) in yields of 58%, 45%, and 36%, respectively. Notably, disubstituted phenols, such as 3,5-dibromophenol (2s) and 3,5-dimethoxyphenol (2t), also participated smoothly under the optimized reaction conditions without compromising the reaction efficiency to furnish 3hs and 3ht, respectively.

We further explored our synthetic strategy for the late-stage functionalization of diverse natural products and bioactive molecules featuring phenolic motifs (Scheme 1b-ii). Initially, reactions involving 2-methyl salicylate (2u) and 2-ethylhexyl salicylate (2v) proceeded effectively, delivering the corresponding azepines 3hu and 3hv in yields of 47% and 34%, respectively. Subsequently, substrates such as 4-benzyloxyphenol (2w), 6-hydroxycoumarin (2x), 2,6-diisopropylphenol (2y), 4-(1,1,3,3-tetramethylbutyl) phenol (2z), and 4-(4-hydroxyphenyl)-butan-2-one (2aa) were successfully converted into their corresponding SF_5_-azepine derivatives (3hw–3haa) in moderate yields (40–58%). Furthermore, more structurally diverse and bioactive phenols such as vanillin (2ab), acetovanillone (2ac), triclosan (2ad), and carvacrol (2ae) participated in the reaction, producing the desired azepines (3hab–3hae) in good yields (up to 75%). Interestingly, pharmaceuticals such as paracetamol (2af) and estrone (2ag) also underwent efficient transformation under the optimized reaction conditions, affording SF_5_-substituted azepines 3haf and 3hag in yields of 60% and 33%, respectively. Notably, the reaction under aqueous conditions in the absence of phenols furnished SF_5_-azepinone 4h in 70% yield *via* its lactim form 4h. Having successfully demonstrated the skeletal transformation of SF_5_-benzenes into SF_5_-azepine scaffolds (Scheme 1b), we explored the reverse process, namely the skeletal reconstruction of SF_5_-azepines back to SF_5_-benzene derivatives (Scheme 1c). Treatment of 5-(pentafluoro-λ^6^-sulfaneyl)-2-phenoxy-3*H*-azepine (3ha) with trifluoroacetic anhydride (TFAA) in acetonitrile efficiently afforded the corresponding SF_5_-substituted *ortho*-aminophenol (5ha) in excellent yield (92%). Substituted SF_5_-azepines bearing Me (3hb), MeO (3hc), and F (3hg) groups were well tolerated under the reaction conditions, furnishing the corresponding aminophenol derivatives 5hb, 5hc, and 5hd, respectively, in excellent yields (up to 94%). Additionally, phenyl-substituted azepine 3ho underwent smooth skeletal editing to deliver *ortho*-aminophenol 5he in 92% yield. This reverse skeletal transformation was also applicable to non-SF_5_-substituted azepine-aryl ethers, enabling their conversion into the corresponding diaryl ethers, as proven by non-substituted phenoxy-3*H*-azepine (3aa), which actively participated in the reaction to deliver the corresponding *ortho*-aminophenol (5aa) in 90% yield. Likewise, substituted phenoxy-3*H*-azepines bearing electronically varied substituents, such as Me (3ba), F (3ca), Br (3da), CF_3_ (3ea), and CO_2_Et (3fa), smoothly participated in the reaction to furnish the corresponding *ortho*-aminophenol derivatives 5ba, 5ca, 5da, 5ea, and 5fa in high yields ranging from 87–94%. These results indicated that the opposite skeletal transformation from azepines 3 to benzenes 5 proceeded smoothly, independent of the electronic state of the functional groups, which is different from the skeletal transformation from benzenes to azepines 3. It is noteworthy that this reverse skeletal transformation can also be performed using difluoroacetic anhydride (DFAA) in place of the commonly employed TFAA. Under these conditions, the corresponding COCF_2_H-substituted diaryl ethers (5haa–5had) were obtained in excellent yields (79–96%). Importantly, the COCF_2_H group lies outside the OECD PFAS definition, ensuring full PFAS-free compatibility.

### Reaction mechanism and electrophilicity analysis

**Fig. 2:**
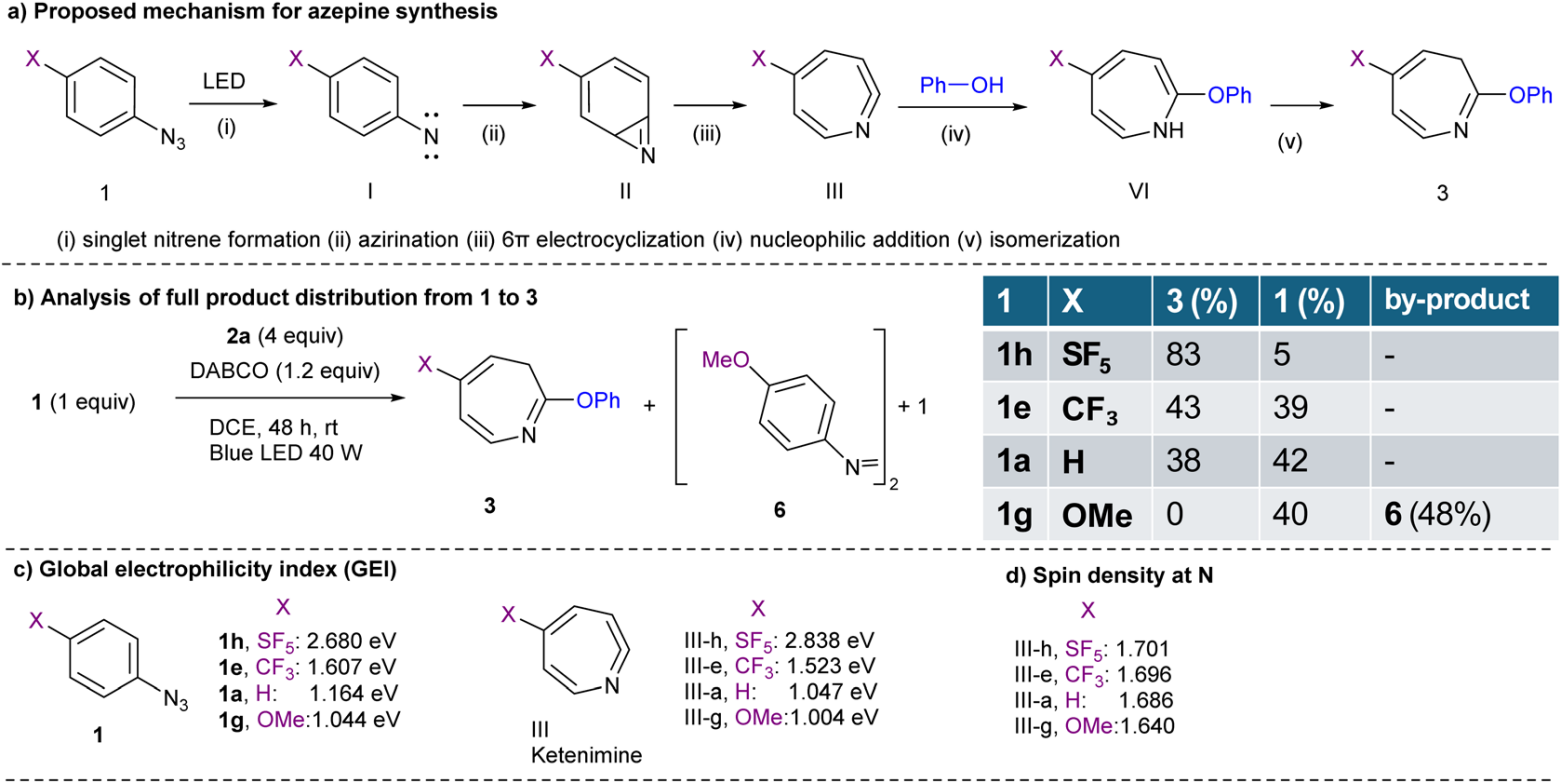
(a) Proposed mechanism. (b) Analysis of full product distribution from 1 to 3. (c) Global electrophilicity index. (d) Spin density on the N atom, DFT, SMD-M06-2X/def2-TZVP.

A plausible reaction mechanism for the photoinduced dearomative coupling of phenyl azide 1 with phenol 2 has been proposed based on previous studies (Fig. 2a). Upon photoirradiation, phenyl azide 1 undergoes photolysis to generate a singlet nitrene intermediate (I). This highly reactive species undergoes regioselective insertion at the *ortho*-position of the aryl ring to form a strained azirine intermediate (II). The azirine subsequently undergoes a thermally allowed 6π-electrocyclic ring-opening reaction to yield a conjugated ketenimine intermediate (III), which acts as a key electrophilic species. Nucleophilic attack by phenol 2 then affords 1*H*-azepine (VI), which undergoes tautomerization or isomerization to the thermodynamically favored 3*H*-azepine product 3. To investigate the role of SF_5_ substitution in this skeletal transformation, we analyzed the full product distribution, including azepine 3, unreacted starting material 1, and the byproduct (Fig. 2b). Notably, SF_5_-phenyl azide (1h) predominantly yielded azepine 3ha with minimal residual starting material 1h (5%). In contrast, phenyl azides bearing CF_3_ (1e), H (1a), and MeO (1g) substituents left similar levels of unreacted starting material 1 (1e: 39%; 1a: 42%; 1g: 40%), regardless of their electronic nature. However, a clear divergence was observed in azepine formation: while 1e (X = CF_3_) and 1a (X = H) provided azepines in ∼43% yield, 1g (X = MeO) failed to produce any azepine (0%), but instead afforded azo-dimer 6 in 48% yield. To rationalize the enhanced reactivity of SF_5_-substituted phenyl azide 1h, we examined their global electrophilicity index (GEI), calculated as the square of the electronegativity divided by the chemical hardness (Fig. 2c). This ground-state molecular descriptor correlates well with electrophilic reactivity, especially toward soft nucleophiles, making it a valuable predictor of reaction outcomes. The GEI values for *para*-substituted phenyl azides 1 (X–C_6_H_4_–N_3_) were as follows: 1h (X = SF_5_), 2.680 eV; 1e (CF_3_), 1.607 eV; 1a (H), 1.164; and 1g (OMe), 1.044 eV. These results indicate that strongly electron-withdrawing substituents like SF_5_ substantially enhance the electrophilicity of azide 1 and its nitrene intermediate I, facilitating smooth formation of azirine II and the ketenimine III. This trend closely matches our experimental findings, where the residual starting material followed the order SF_5_ (5%) <<< CF_3_ (39%) ≈ H (42%) ≈ MeO (40%). We further computed the GEI values for ketenimine intermediates (III) to assess their susceptibility to phenolic nucleophiles 2. The GEI values were as follows: III-h (SF_5_), 2.838 eV; III-e (CF_3_), 1.523 eV; III-a (H), 1.047 eV; and III-g (MeO), 1.004 eV. Again, the electrophilicity of the SF_5_ group was correlated with the experimental yields of azepine-aryl ethers: SF_5_ (83%) >>> CF_3_ (43%) ≈ H (38%), with no product observed for OMe (0%). Density functional theory (DFT) calculations were performed at the SMD-M06-2X/def2-TZVP level of theory for the spin density on the N atom, and it showed a correlation with the experimental yield (Fig. 2d).

#### Mechanistic rationale for divergent behaviour focusing on the electronic landscape of nitrenes

**Fig. 3:**
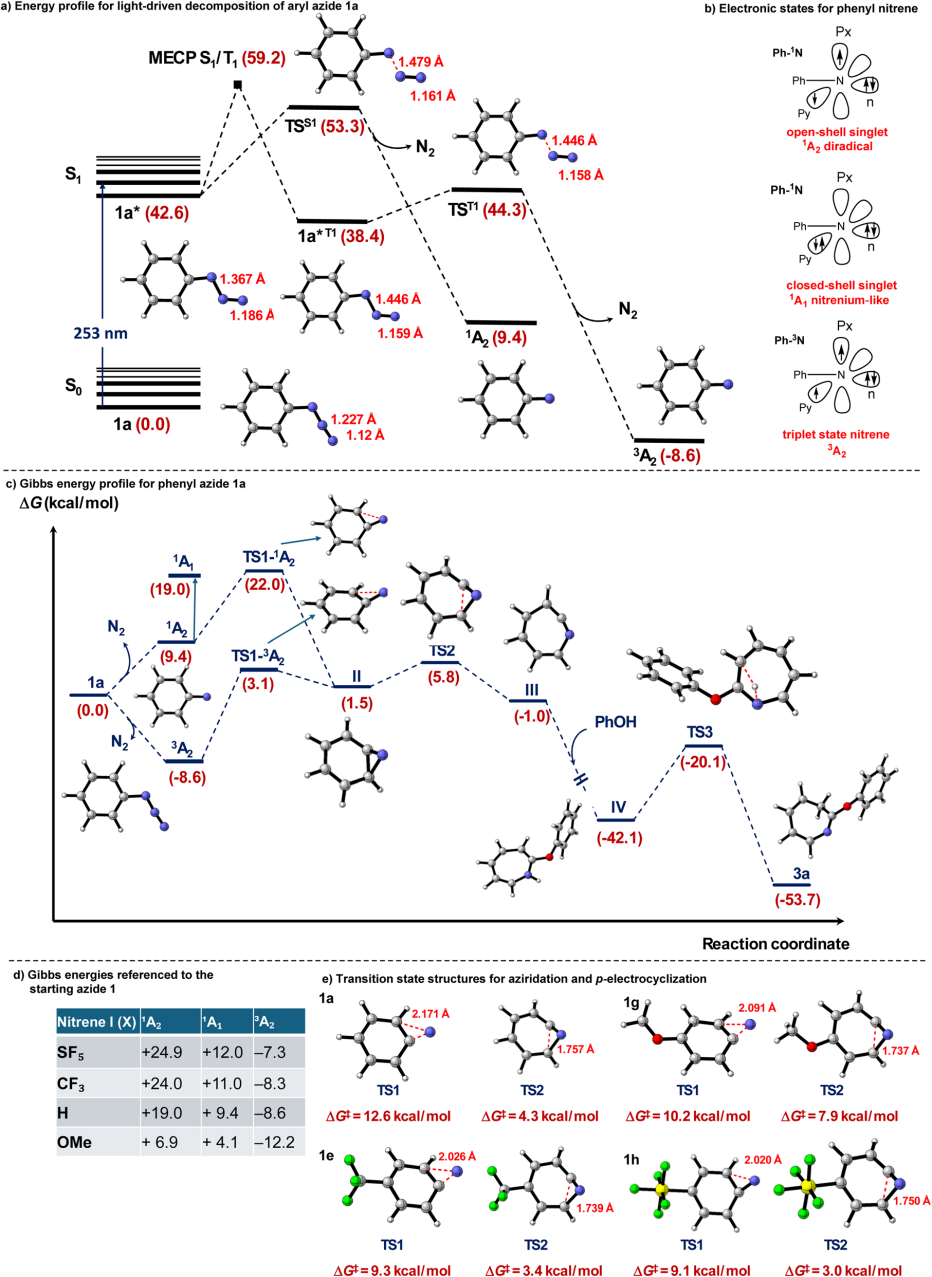
(a) Calculated energy profile for light-driven decomposition of phenyl azide 1a. The relative energies are given in kcal mol^−1^. (b) Electronic states of phenyl nitrene. (c) Free energy profile for phenyl azide 1a calculated at the SMD/M06-2X/def2-TZVP level of theory in DCE. Relative Gibbs energies are given in kcal mol^−1^. (d) DFT, SMD-M06-2X/def2-TZVP; Gibbs energies in the table are referenced to the starting azide 1. (e) Optimized transition state structures for aziridination and 6π-electrocyclization. Gibbs activation barriers are given in kcal mol^−1^ and distances in Å.

We investigated the detailed mechanism of photoactivation of phenyl azide (1a) by means of TD-DFT calculations at the SMD-M06-2X/def2-TZVP level of theory using DCE as solvent (SMD model). TD-DFT calculations showed that the excitation at 253 nm with significant oscillator strength (*f* = 0.1220), mainly corresponds to the transition of an electron from the HOMO to the LUMO of aryl azide 1a to generate the excited state 1a* with a weakened azide as evidenced by the bond lengthening from 1.227 Å in 1a to 1.367 Å in 1a* (Fig. 3a). Phenyl nitrene I, obtained upon N_2_ cleavage can exist in three different electronic states: the open-shell singlet (^1^A_2_ diradical, one electron in each of the two orthogonal N p orbitals (αβ-coupled)), closed-shell singlet (^1^A_1_, nitrenium, similar to two electrons paired in one in-plane p-orbital, with a vacant N π* orbital), and triplet (^3^A_2_, one electron in each of the two orthogonal N p orbitals (αα coupled)) (Fig. 3b).^29^ Starting from 1a*, N_2_ can be cleaved *via* the transition structure TS^S1^ to yield phenyl nitrene I at the open-shell singlet ^1^A_2_. Alternatively, 1a* might also be able to form the corresponding nitrene I at triplet state 1a*^T1^ through intersystem crossing (ISC). To evaluate the possibility of this ISC process, the energy crossing point (MECP) between the singlet and triplet (MECP S_1_/T_1_) was located with a barrier of 16.6 kcal mol^−1^. Spin density analysis of the triplet state 1a*^T1^ reveals that the two unpaired electrons are predominantly localized on the nitrogen atom, with a spin density contribution of 1.686, and the remaining electron density, influenced by π-conjugation, is delocalized over the carbon atoms of the adjacent six-membered ring bonded to the nitrogen. Computational results indicate that TS^S1^ lies significantly lower in energy than MECP S_1_/T_1_, suggesting that formation of the open-shell singlet ^1^A_2_ is kinetically favoured (Fig. 3a). This finding aligns well with previous studies, which have shown that light-induced decomposition of aryl azides predominantly yields open-shell singlet nitrenes.

To get further insights into the reaction mechanism, the Gibbs energy profile for phenyl azide (1a) as the model substrate was investigated at the SMD-M06-2X/def2TZVP level of theory in dichloroethane as solvent (Fig. 3c). The unrestricted open-shell singlet (^1^A_2_) was located 9.4 kcal mol^−1^ higher in the Gibbs energy profile than the phenyl azide (1a), and the corresponding restricted closed-shell singlet (^1^A_1_) was found to be 19.0 kcal mol^−1^ higher in energy than the unrestricted closed-shell singlet ^1^A_1_. However, the triplet state (^3^A_2_) was found to be 8.6. kcal mol^−1^ more stable than the phenyl azide (1a). We further studied the possible transformations of aryl azides 1 with different substituents leading to the formation of nitrenes (Fig. 3d). For the *p*-MeO system (1g), the strong +M effect of MeO stabilizes the empty N π* orbital. Thus, the lone-pair donation both narrows the ^1^A_2_–^1^A_1_ gap (2.8 kcal mol^−1^) (*cf.*, ^1^A_2_–^1^A_1_ gap of SF_5_, 12.9 kcal mol^−1^) and increases spin–orbit coupling, accelerating ISC by >10^2^-fold to the triplet surface, becoming the dominant pathway.^30–34^ The resulting triplet ^3^A_2_ nitrene (−12.2 kcal mol^−1^) from the *p*-MeO system (1g) dimerizes at the diffusion limit to azo-dimer 6 (48%); azepine 3ga is not detected, a well-established outcome for triplet aryl nitrenes in non-protic solvents. Next, the intramolecular aziridination was investigated from ^1^A_2_ and ^3^A_2_ (Fig. 3c). DFT calculations demonstrate that both ^1^A_2_ and ^3^A_2_ intermediates are reactive toward the aziridination, as inferred from the computed Gibbs activation energies for TS1-^1^A_2_ and TS1-^3^A_2_. The ^1^A_2_ intermediate directly gives three-membered azirine II*via* transition structure TS1-^1^A_2_ with a Gibbs activation energy of 12.6 kcal mol^−1^ from intermediate ^1^A_2_.

Alternatively, the barrier from ^3^A_2_ involving the triplet state was computed to be 11.7 kcal mol^−1^*via* transition structure TS1-^3^A_2_, showing that the process is also feasible. Calculations reveal that the endergonicity for transformation of three-membered azirine II from phenyl azide (1a) is 1.5 kcal mol^−1^. After the three-membered ring formation, azirine II is involved in 6π-electrocyclization which is predicted to be fast *via*TS2 with a Gibbs activation barrier of 4.3 kcal mol^−1^, delivering ketenimine intermediate III in a slightly exergonic step (Δ*G* = −1.0 kcal mol^−1^). Next, the phenol nucleophilic attack affords 1*H*-azepine IV in a highly exergonic and irreversible step (Δ*G* = −42.1 kcal mol^−1^). Finally, the formed 1*H*-azepine IV undergoes tautomerization to yield the thermodynamically favored 3*H*-azepine product 3a. This step is computed to be exergonic by about 11.6 kcal mol^−1^. In the case of CF_3_-azepine, the computed activation barriers for aziridination and 6π-electrocyclization were found to be 9.3 and 3.4 kcal mol^−1^, respectively (Fig. 3e). The relatively high activation barriers for phenyl azide (1a) and CF_3_-phenyl azide 1e rationalize why the yield is moderate in the case of products 3aa and 3ea. Finally, the computed activation barriers for aziridination and 6π-electrocyclization of SF_5_-azepine from SF_5_-phenyl azide 1h were found to be 9.1 and 3.0 kcal mol^−1^, respectively. In this case, the computed activation barrier for aziridination is more favorable, rationalizing the higher reactivity of SF_5_-azepines. As inferred from these DFT calculations, it is notable that aziridination, with the highest activation barrier of the entire mechanism, is the rate determining step of the reaction mechanism. The reaction mechanism studied by DFT calculations agrees with the experimental findings as computed barriers are affordable at room temperature. Finally, phenyl azide 1g (OMe case) showed similar barriers for aziridination, however, the computed barrier for 6π-electrocyclization was found to be higher in energy (7.9 kcal mol^−1^). This data, together with the data in Fig. 3d, indicate that the dimerization reaction would be favored for MeO-substituted phenyl azide 1g.

### Extension from SF_5_ to SF_4_

**Scheme 2:**
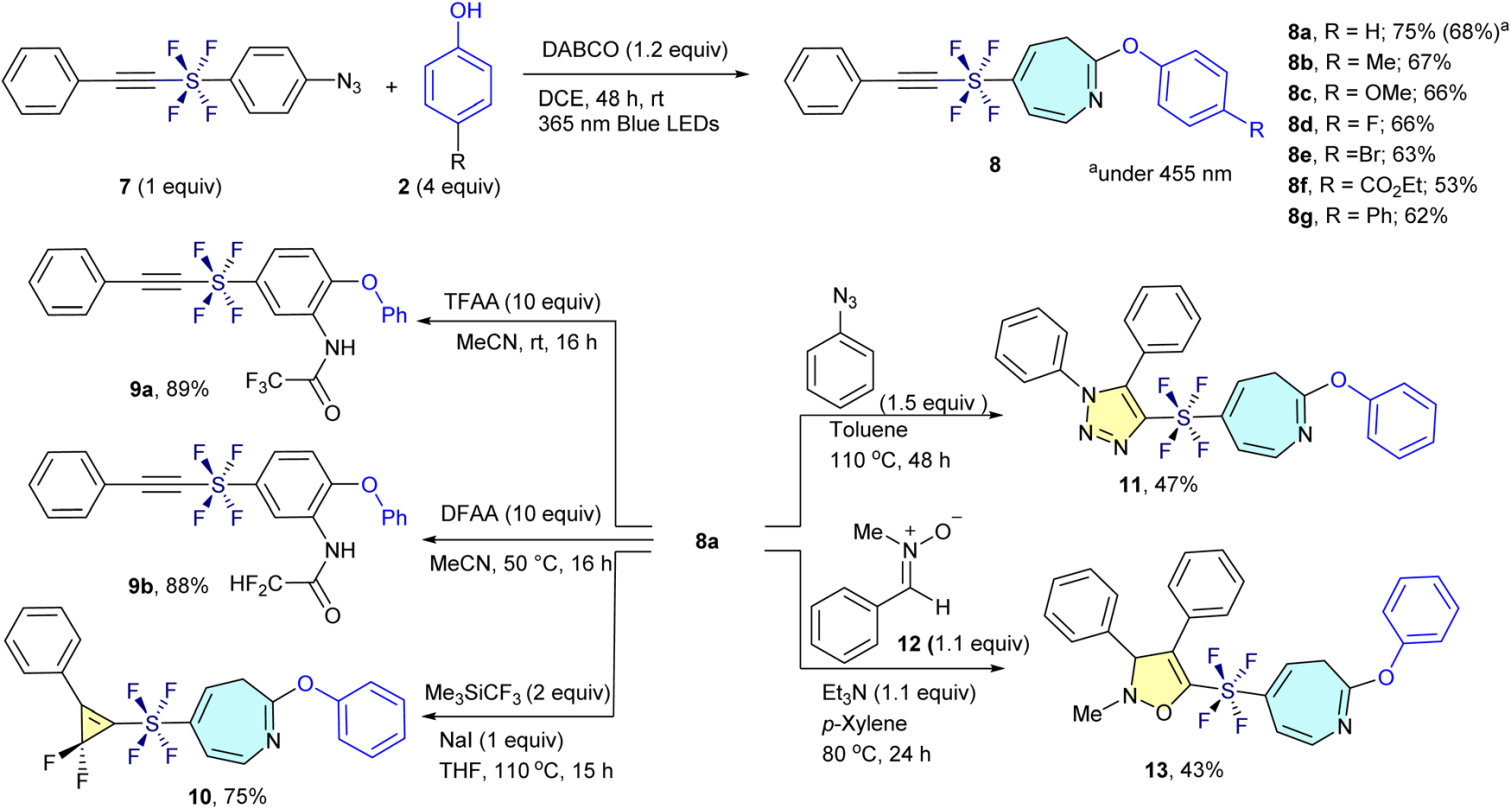
Chemo-selective transformation of alkynylated *trans*-SF_4_-aryl azides (7) to azepines (8), and their further chemo-selective derivatization to 9, 10, 11 and 13.

We extended our dearomative skeletal editing methodology from SF_5_-aryl azides (1) to alkynylated *trans*-SF_4_-aryl azide (7). Given the electron-deficient and highly polarized nature of the alkynyl moiety in compound 7, which is prone to reactions with nucleophiles, electron-rich species, and carbenes, we initially anticipated potential side reactions involving the alkyne. In particular, the presence of both an aryl azide and an alkyne linked *via* the SF_4_ unit raised concerns about competing nitrene insertion pathways. Surprisingly, the photoinduced reaction proceeded with high chemoselectivity at the aryl azide site, leaving the alkyne untouched. This enabled efficient ring expansion to afford the desired alkynyl-*trans*-SF_4_-azepine 8a in 68% yield under the same conditions used for the SF_5_ analogues. The yield further improved to 75% when the reaction was conducted under 365 nm irradiation. The transformation proved broadly applicable across a variety of functionalized *trans*-SF_4_ aryl azides. Substituents including electron-donating (Me and OMe), halogen (F and Br), electron-withdrawing (CO_2_Et), and aryl (Ph) groups were well tolerated, providing the corresponding *trans*-SF_4_ azepines 8b–8g in 53–67% yields (Scheme 2, top).

Further derivatization demonstrated the synthetic utility of these scaffolds. The alkynylated SF_4_-azepine 8a underwent ring re-contraction upon treatment with TFAA and acetonitrile, regenerating the corresponding SF_4_-benzene derivative 9a bearing amino and ether functionalities in 89% yield. The reaction of 8a with DFAA furnished the PFAS-free ring-reconstructed product 9b in similarly high yield (88%). In addition, selective gem-difluorocyclopropanation of 8a was achieved using Me_3_SiCF_3_ and NaI in THF, furnishing the unique SF_4_-azepine–gem-difluorocyclopropene hybrid 10 in 75% yield. Next, the compound 8a participated in a click reaction with azidobenzene 1a, resulting in the formation of the SF_4_-azepine–triazole (11) with 47% yield. After that, the compound 8a smoothly involved in a (3 + 2) cycloaddition reaction with nitrone (12) to afford the SF_4_-azepine–isoxazole (13) with 43% yield (Scheme 2, bottom). These results demonstrate that the *trans*-SF_4_ group functions as a stable, linear, and highly versatile linker under photoinduced skeletal editing conditions. The resulting SF_4_-azepine frameworks represent promising candidates for PFAS-safe agrochemical development, combining structural novelty with tunable electronic properties and synthetic flexibility.

## Conclusions

We have developed a bidirectional skeletal-editing platform centered on high-valent sulfur fluorides—namely, pentafluorosulfanyl (SF_5_) and tetrafluorosulfanyl (SF_4_)—that enables the reversible interconversion between benzenes and azepine-aryl ethers under mild, visible-light irradiation. These motifs function not merely as PFAS-safe perfluoroalkyl alternatives but as reactivity-switching handles that unlock new chemical transformations. The strongly electron-withdrawing nature of SF_5_ selectively stabilizes the open-shell singlet nitrene intermediate, accelerating a 6π-electrocyclization/ring-expansion cascade and delivering azepines in yields significantly exceeding those of CF_3_- or their non-fluorinated counterparts. Computational studies support this unique reactivity enhancement. Importantly, the resulting azepines undergo clean skeletal contraction upon treatment with acid anhydrides, regenerating the original SF_5_- or SF_4_-aryl cores while introducing an aryl-ether functionality—marking the first efficient return pathway for any high-valent sulfur fluoride azepine. While demonstrated using phenolic nucleophiles, the underlying mechanism—diradical nitrene capture followed by skeletal reorganization—should be generalizable to other soft nucleophiles such as amines, thiols, and carboxylates. This work thus establishes high-valent sulfur fluorides as programmable scaffolding elements for visible-light skeletal editing and introduces a sustainable route to structurally diverse, fluorinated heterocycles. Because the SF_5_ group serves as a terminal substituent while the SF_4_ unit functions as a linear connector, this platform offers a versatile and PFAS-conscious entry point for the design of fluorinated agrochemicals and advanced materials.

## Author contributions

CN optimized the reaction conditions, surveyed the substrate scope, analyzed the data and discussed the results with NS. TM, MZB, and SW prepared starting materials. SO and JE conducted DFT studies. CN and NS wrote the manuscript. NS supervised the study. All authors contributed to the manuscript and approved the final version of the manuscript.

## Conflicts of interest

The authors declare no conflicts of interest.

## Supplementary Material

SC-OLF-D5SC08177G-s001

SC-OLF-D5SC08177G-s002

## Data Availability

CCDC 2443987 contains the supplementary crystallographic data for this paper.^35^ The data that support the findings of this study are available within the article and the supplementary information (SI). Supplementary information: materials and methods, experimental procedures, characterization data, and NMR spectra. See DOI: https://doi.org/10.1039/d5sc08177g.
