# Supplementary material for: High-valent sulfur fluorides as reactivity switches for PFAS-free benzene–azepine skeletal editing
Source: Chem Sci. 2025 Nov 19;17(3):1840–9. doi: 10.1039/d5sc08177g (PMC12659759; doi:10.1039/d5sc08177g)

Supplementary Information (SI) for Chemical Science.
This journal is © The Royal Society of Chemistry 2025

Supplementary Information

High-valent sulfur fluorides as reactivity switches for PFAS-free benzene–azepine skeletal editing

Chavakula Nagababu,[1] Takuya Muramatsu,[2] Muhamad Zulfaqar Bacho,[1] Shiwei Wu,[2] Seishu Ochiai,[2] Jorge Escorihuela,[3,4] and Norio Shibata*[1,2]

Affiliations:

[1]Department of Nanopharmaceutical Sciences, Nagoya Institute of Technology, Gokiso, Showa-ku, Nagoya 466-8555, Japan

[2]Department of Engineering, Nagoya Institute of Technology, Gokiso, Showa-ku, Nagoya 466-8555, Japan

[3]Departamento de Química Orgánica, Universitat de València, Avda. Vicente Andrés Estellés s/n, Burjassot 46100, Valencia, Spain

[4]Instituto de Ciencia Molecular (ICMol), Universitat de València, Calle Catedrático José Beltrán 2, Paterna, Valencia, Spain
*Corresponding author Email: nozshiba@nitech.ac.jp

Table of Contents

1. General information………………………………………………………… S2
2. General procedure for the Synthesis of (4-azidophenyl)pentafluoro-λ^6-sulfane and (4-azidophenyl)tetrafluoro(phenylethynyl)-λ^6-sulfane (**1**) [Method A]……………………………………………………………... S3
3. General procedure for the synthesis of substituted 2-phenoxy-3*H*-azepine (**3**) [Method B]………………………………………………………………… S5
4. General procedure for the synthesis of 5-(pentafluoro-λ^6-sulfaneyl)-1,3-dihydro-2*H*-azepin-2-one (**4h**).. S25
5. General procedure for synthesis of substituted 2,2,2-trifluoro-*N*-2-phenoxyphenyl)acetamide and 2,2-difluoro-*N*-(2-phenoxyphenyl)acetamide (**5**) [Method-C]………………………………………………………………… S26
6. General procedure for the synthesis of substituted 2-phenoxy-5-(tetrafluoro(phenylethynyl)-λ^6-sulfaneyl)-3*H*-azepine (**8**) [Method D]……… S34
7. Synthetic transformations of 2-phenoxy-5-(tetrafluoro(phenylethynyl)-λ^6-sulfaneyl)-3*H*-azepine (**8a**)…………………………………………………... S38
8. Computational details... S42
9. References………………………………………………………………… S65
10. X-ray crystallography data (**3ha**)…………………………………………… S66

11. ^{1}H NMR, ^{13}C NMR and ^{19}F NMR spectral copies of compounds………….. S68

1. General information and materials

All reactions were performed using oven-dried glassware and were performed under positive pressure of nitrogen unless otherwise mentioned. All the reactions were monitored by thin-layer chromatography (TLC) carried out on 0.25 mm Merck silica gel (60-F254) or measurement of ^{19}F NMR. The TLC plates were visualized with UV light. Products were purified by column chromatography carried out on columns packed with silica gel (60N spherical neutral size 63–210 µm). The ^{1}H NMR (500 MHz & 700 MHz), ^{13}C NMR (126 MHz) and ^{19}F NMR (282 MHz, 376 MHz & 658 MHz), spectra for solution in $CDCl_3$ and DMSO-d_6 were recorded on a Bruker Avance 500, a Varian 300, a Bruker Avance 400, and JEOL700. Chemical shifts (δ) are expressed in ppm downfield from TMS (δ = 0.00 ppm) and $CDCl_3$ (δ = 7.26 ppm) or DMSO-d_6 (δ = 2.50 ppm) for ^{1}H NMR, $CDCl_3$ (δ = 77.16 ppm) or DMSO-d_6 (δ = 39.5 ppm) for ^{13}C NMR and internal standard, C_6F_6 (δ = −162.20 ppm) for ^{19}F NMR. High-resolution mass spectrometry was recorded on a SHIMADZU GCMS-QP5050A (EI-MS) and SHIMADZU LCMS-2020 (ESI-MS and APCI-MS). High-resolution mass spectrometry (HRMS) was carried out on an electron impact ionization mass spectrometer with a micro-TOF analyzer and recorded on a Waters, GCT Premier (EI-MS) with a TOF analyzer. Infrared spectra were recorded on a JASCO FT/IR-4100 spectrometer. Melting points were recorded on a BUCHI M-565. The EvoluChem PhotoRedOx Box™ was used as the photoreactor, and Kessil A160WE Tuna Blue, LED lighting (40W) was used as the light source for irradiation. The distance of the light source to the irradiation vessel was fixed by using the PhotoRedOx Box™ (as shown in Figure S1). Chemicals and solvents were purchased and used without further purification unless otherwise noted.

2. General procedure for the Synthesis of (4-azidophenyl)pentafluoro-λ^6-sulfane and (4-azidophenyl)tetrafluoro(phenylethynyl)-λ^6-sulfane (1) [Method A]

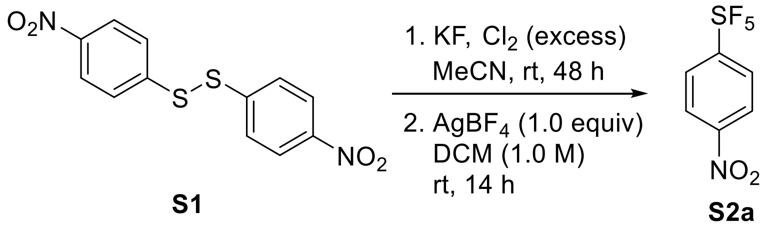

An oven-dried 250 mL PFA bottle with a magnetic stirring bar was charged with disulfide (**S1**, 6.17 g, 20 mmol), spray-dried KF (18.59 g, 320 mmol), and anhydrous acetonitrile (120 mL) inside the glove box. The bottle was then capped and sealed with parafilm and taken out from the glovebox. The bottle was cooled in an ice/water bath while chlorine gas was bubbled through the stirred reaction mixture for approximately 8 minutes. The bottle was sealed, and the reaction mixture was stirred at 0 °C for 3 hours. Stirring then continued at room temperature for 48 hours. After the reaction was completed, the solution was filtered under a nitrogen atmosphere to another 250 ml PFA bottle using a PP/ETFE filter. The residue was washed with MeCN (2.5 ml × 2). MeCN was evaporated in vacuo to give crude arylsulfur chlorotetrafluoride (9.17 g) 86% yield. The crude was directly used for the next step without further purification.

Crude arylsulfur chlorotetrafluoride (9.17 g, 34.5 mmol, 1.0 equiv) and $AgBF_4$ (6.72 g, 34.5 mmol, 1.0 equiv) were weighed into a PFA tube containing a magnetic stirrer bar in a glove box. Anhydrous dichloromethane (34.5 mL, 1.0 M) was added in the glovebox. The mixture was stirred at room temperature for 14 h outside the glovebox. After complete conversion, the reaction mixture was filtered through a pad of silica and washed with dichloromethane. The solvent was removed in vacuo to give the crude product, which was purified by chromatography on silica gel (eluted with pentane/DCM: 3/1) to afford the corresponding product **S2a** as a white or pale yellow solid (7.36 g, 86% yield). The data of compound **S2a** were in accordance with previous literature.[1]

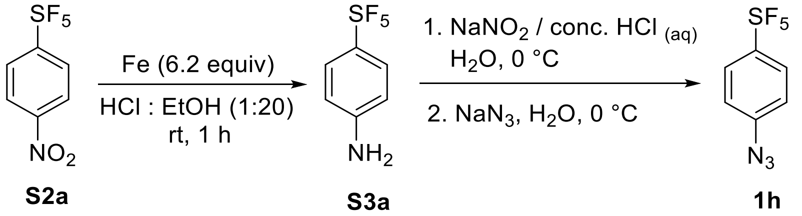

To a stirred solution of nitro compound **S2a** (2.0 g, 8 mmol) in ethanol (80 mL, 0.1 M) was added Fe powder (2.77 g, 6.2 equiv, 49.6 mmol), followed by the slow addition of concentrated hydrochloric acid (4 mL, 2.0 M) at 0 °C. After the reaction mixture was stirred for 1 h at room temperature, the reaction was poured into ice water and neutralized with sodium carbonate until alkaline, then extracted with dichloromethane (3 × 20 mL). The combined extracts were dried over anhydrous Na_2SO_4, filtered, concentrated in vacuo to give the crude 4-(pentafluoro-λ^6-sulfanyl) aniline (1.4 g, 82% yield). The crude was directly used for the next step without further purification.[2]

Crude 4-(pentafluoro-λ^6-sulfanyl) aniline (**S3a**) (1.5 g, 6.84 mmol) was then mixed with conc. HCl aqueous solution (8.55 mL) and the obtained mixture were stirred vigorously at 0 °C (ice bath) for 15 min. Note 1: the aniline was not dissolved. A solution of sodium nitrite (0.71 g, 10.26 mmol, 1.5 equiv) in water (6.45 mL, 1.06 M) was added in a dropwise manner over 5 min. Note 2: formation of a yellow solution was observed. The mixture was stirred at 0 °C for an additional 30 min. A solution of sodium azide (0.89 g, 13.68 mmol, 2.0 equiv) in water (6.45 mL, 1.06 M) was added dropwise at 0 °C within 5 min, and the resulting reaction mixture was stirred vigorously at room temperature for a further 3 h. Note 3: The addition of the sodium azide solution resulted in the disappearance of the yellow colour. The mixture was extracted with ethyl acetate (3 × 35 mL), the extracts were combined, washed with water (1 × 50 mL), dried over anhydrous sodium sulphate and concentrated under reduced pressure. The crude product was purified by flash chromatography with hexane to afford the (4-azidophenyl)pentafluoro-λ^6-sulfane (**1h**) (1.5 g, 89% yield) as an orange oil. The data of compound **S3** were in accordance with previous literature.[3]

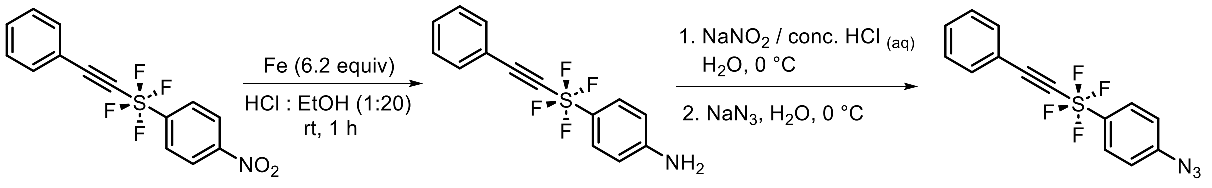

S2b **S3b** **7**

To a stirred solution of nitro compound **S2b** [prepared by reported literature[4]] (2.57 g, 8 mmol) in ethanol (80 mL, 0.1 M) was added Fe powder (2.77 g, 49.6 mmol), followed by the slow addition of concentrated hydrochloric acid (4 mL, 2.0 M) at 0 °C. After the reaction mixture was stirred for 1 h at room temperature, the reaction was poured into ice water and neutralized with sodium carbonate until alkaline, then extracted with dichloromethane (3 × 20 mL). The combined extracts were dried over anhydrous Na_2SO_4, filtered, concentrated in vacuo

to give the crude 4-(tetrafluoro(phenylethynyl)-λ^6-sulfaneyl) aniline. The crude was directly used for the next step without further purification. Crude 4-(tetrafluoro(phenylethynyl)-λ^6-sulfaneyl)aniline (**S3b**) was then mixed with conc. HCl aqueous solution (8.55 mL) and the obtained mixture were stirred vigorously at 0 °C (ice bath) for 15 min. A solution of sodium nitrite (0.71 g, 10.26 mmol, 1.5 equiv) in water (6.45 mL, 1.06 M) was added in a dropwise manner over 5 min. The mixture was stirred at 0 °C for an additional 30 min. A solution of sodium azide (0.89 g, 13.68 mmol, 2.0 equiv) in water (6.45 mL, 1.06 M) was added dropwise at 0 °C within 5 min, and the resulting reaction mixture was stirred vigorously at room temperature for a further 3 h. The mixture was extracted with ethyl acetate (3 × 35 mL), the extracts were combined, washed with water (1 × 50 mL), dried over anhydrous sodium sulphate and concentrated under reduced pressure. The crude product was purified by column chromatography with hexane to afford the (4-azidophenyl)tetrafluoro(phenylethynyl)-λ^6-sulfane (**7**) as pale yellow solid, yield 65% (1.70 g), mp: 164.3 – 166.4 °C; **^{1}H NMR** (700 MHz, $CDCl_3$) δ 7.77-7.75 (m, 2H), 7.57 (dd, J = 5.2, 3.2 Hz, 2H), 7.45-7.42 (m, 1H), 7.39-7.36 (m, 2H), 7.03 (d, J = 8.7 Hz, 2H); **^{13}C NMR** (126 MHz, $CDCl_3$) δ 156.2-155.4 (m), 142.5, 132.6, 130.4, 128.7, 127.8-127.6 (m, J = 9.7, 4.8 Hz), 118.9, 118.6, 96.1-94.4 (m), 72.8 (p, J = 9.6 Hz); **^{19}F NMR** (658 MHz, $CDCl_3$) δ 88.46 (s, 4F); **IR (KBr)**: 3092, 2923, 1636, 1492, 1324, 1247, 782, 764, 728 cm^{-1}.

3. General procedure for the synthesis of substituted 2-phenoxy-3*H*-azepine (3) [Method B]

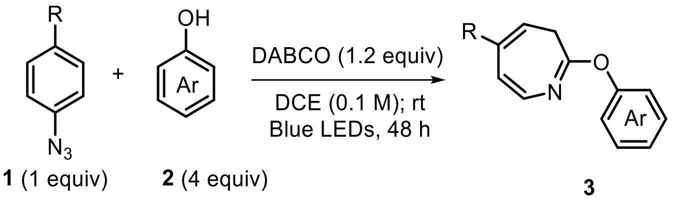

In an oven-dried screw-cap vial equipped with a magnetic stir bar, substituted azido benzene (**1**) (0.20 mmol, 1 equiv), phenol (**2**) (0.80 mmol, 4 equiv), and DABCO (0.24 mmol, 1.2 equiv) were dissolved in DCE (2 mL, 0.1 M). The reaction mixture was stirred and irradiated with blue LED light (Kessil A160WE Tuna Blue, LED lighting (40 W)) for 48 hours. Afterward, the solvent was removed under reduced pressure, and the crude was purified by column chromatography using *n*-hexane/ethyl acetate to afford Pure compound (**3**).

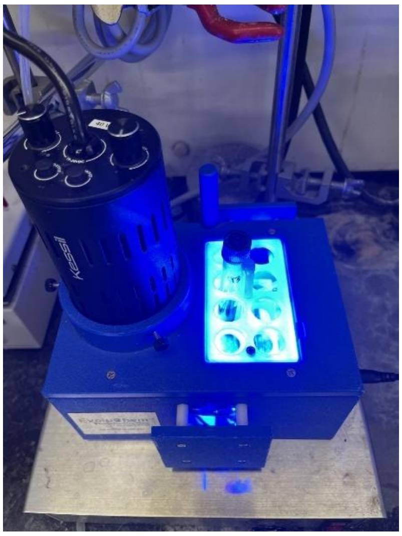

Figure S1: Photoreactor setup

2-Phenoxy-3*H*-azepine (3aa)

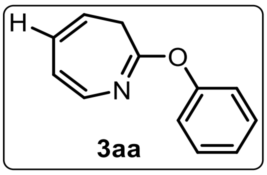

Following the general method **B** (azidobenzene **1a** (23.8 mg, 0.20 mmol), phenol **2a** (75.3 mg, 0.80 mmol), and DABCO (26.9 mg, 0.24 mmol) in 2 mL anhydrous DCE were used) compound **3aa** was obtained as a white solid, yield 38% (14.0 mg), mp: 57.9 – 59.4 °C; **^{1}H NMR** (500 MHz, $CDCl_3$) δ 7.36-7.31 (m, 2H), 7.18-7.13 (m, 1H), 7.0-6.96 (m, 2H), 6.90 (d, *J* = 8.1 Hz, 1H), 6.36 (dd, *J* = 8.9, 5.7 Hz, 1H), 6.06 (dd, *J* = 8.1, 5.7 Hz, 1H), 5.44 (dt, *J* = 8.8, 6.7 Hz, 1H), 2.84 (d, *J* = 6.7 Hz, 2H); **^{13}C NMR** (126 MHz, $CDCl_3$) δ 153.3, 148.2, 137.4, 129.5, 128.3, 125.3, 121.4, 116.5, 115.8, 33.6; **HRMS (ESI)**: calcd for $C_{12}H_{12}NO$ $[M + H]^+$: 186.0919, found: 186.0921; **IR (KBr)**: 3023, 2992, 1628, 1589, 1490, 1313, 1247, 1204, 881, 808, 796 cm^{-1}.

5-Methyl-2-phenoxy-3*H*-azepine (3ba)

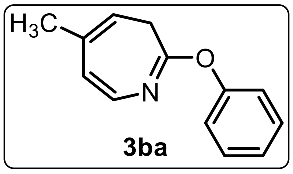

Following the general method **B** (1-azido-4-methylbenzene **1b** (26.6 mg, 0.20 mmol), phenol **2a** (75.3 mg, 0.80 mmol), and DABCO (26.9 mg, 0.24 mmol) in 2 mL anhydrous DCE were used) compound **3ba** was obtained as a yellow oil, yield 25% (9.9 mg), **^{1}H NMR** (500 MHz,

$CDCl_3$) δ 7.36-7.31 (m, 2H), 7.18-7.13 (m, 1H), 7.04-7.00 (m, 2H), 6.80 (d, *J* = 8.3 Hz, 1H), 5.94 (d, *J* = 8.3 Hz, 1H), 5.21 (td, *J* = 6.7, 0.9 Hz, 1H), 2.75 (d, *J* = 6.5 Hz, 2H), 1.94 (s, 3H); **^{13}C NMR** (126 MHz, $CDCl_3$) δ 153.3, 150.9, 136.7, 136.6, 129.5, 125.2, 121.5, 119.0, 113.1, 32.8, 21.3; **HRMS (ESI)**: calcd for $C_{13}H_{14}NO$ [M + H]$^+$: 200.1075, found: 200.1082; **IR (KBr)**: 3054, 2968, 1630, 1590, 1490, 1322, 1246, 1203, 888, 800, 787 cm^{-1}.

5-Fluoro-2-phenoxy-3*H*-azepine (3ca)

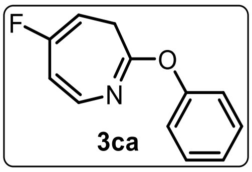

Following the general method **B** (1-azido-4-fluorobenzene **1c** (27.4 mg, 0.20 mmol), phenol **2a** (75.3 mg, 0.80 mmol), and DABCO (26.9 mg, 0.24 mmol) in 2 mL anhydrous DCE were used) compound **3ca** was obtained as a white solid, yield 20% (8.1 mg), mp: 59.4 – 60.9 °C; **^{1}H NMR** (500 MHz, $CDCl_3$) δ 7.39-7.34 (m, 2H), 7.22-7.17 (m, 1H), 7.03 (ddd, *J* = 4.5, 3.3, 1.8 Hz, 2H), 6.95 (dd, *J* = 8.6, 5.6 Hz, 1H), 5.95 (ddd, *J* = 8.6, 7.0, 1.7 Hz, 1H), 5.12 -5.05 (m, 1H), 2.71 (dd, *J* = 7.3, 1.9 Hz, 2H); **^{13}C NMR** (126 MHz, $CDCl_3$) δ 159.8 (d, *J* = 244.3 Hz), 152.9, 152.3 (d, *J* = 5.7 Hz), 139.8 (d, *J* = 10.1 Hz), 129.6, 125.7, 121.4, 107.8 (d, *J* = 30.3 Hz), 94.8 (d, *J* = 26.1 Hz), 28.2 (d, *J* = 10.0 Hz); **^{19}F NMR** (282 MHz, $CDCl_3$) δ -113.07 (s, 1F); **HRMS (ESI)**: calcd for $C_{12}H_{11}NOF$ [M + H]$^+$: 204.0825, found: 204.0822; **IR (KBr)**: 3056, 2958, 1641, 1589, 1490, 1322, 1254, 1132, 890, 804, 780 cm^{-1}.

5-Bromo-2-phenoxy-3*H*-azepine (3da)

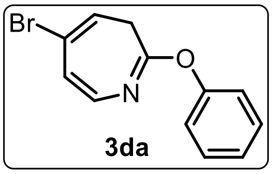

Following the general method **B** (1-azido-4-bromobenzene **1d** (39.6 mg, 0.20 mmol), phenol **2a** (75.3 mg, 0.80 mmol), and DABCO (26.9 mg, 0.24 mmol) in 2 mL anhydrous DCE were used) compound **3da** was obtained as a yellow solid, yield 33% (17.4 mg), mp: 44.6 – 46.3 °C; **^{1}H NMR** (500 MHz, $CDCl_3$) δ 7.38-7.33 (m, 2H), 7.22 -7.17 (m, 1H), 7.05-7.01 (m, 2H), 6.81 (d, *J* = 8.3 Hz, 1H), 6.13 (dd, *J* = 8.3, 1.0 Hz, 1H), 5.69 (t, *J* = 7.2 Hz, 1H), 2.82 (d, *J* = 7.0 Hz, 2H); **^{13}C NMR** (126 MHz, $CDCl_3$) δ 152.9, 150.3, 138.7, 129.6, 125.7, 121.4, 120.5, 117.8, 116.8, 33.8; **HRMS (ESI)**: calcd for $C_{12}H_{11}NOBr$ [M + H]$^+$: 264.0024, found: 264.0027; **IR (KBr)**: 3055, 2995, 1631, 1528, 1317, 1247, 1196, 1160, 882, 805, 786 cm^{-1}.

2-Phenoxy-5-(trifluoromethyl)-3*H*-azepine (3ea)

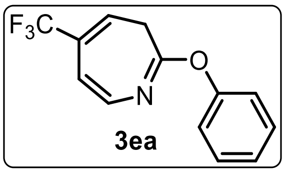

Following the general method **B** (1-azido-4-(trifluoromethyl)benzene **1e** (37.4 mg, 0.20 mmol), phenol **2a** (75.3 mg, 0.80 mmol), and DABCO (26.9 mg, 0.24 mmol) in 2 mL anhydrous DCE were used) compound **3ea** was obtained as a white solid, yield 43% (21.7 mg), mp: 69.5 – 71.2 °C; **^{1}H NMR** (500 MHz, $CDCl_3$) δ 7.38-7.33 (m, 2H), 7.20 (ddd, *J* = 8.5, 2.2, 1.1 Hz, 1H), 7.06 (d, *J* = 8.3 Hz, 1H), 7.03-6.99 (m, 2H), 6.17 (d, *J* = 8.3 Hz, 1H), 5.97 (td, *J* = 7.1, 0.8 Hz, 1H), 2.92 (d, *J* = 6.7 Hz, 2H); **^{13}C NMR** (126 MHz, $CDCl_3$) δ 152.9, 149.8, 140.4, 131.2 (q, *J* = 30.9 Hz), 129.6, 125.8, 123.4 (q, *J* = 273.2 Hz), 121.3, 117.9 (q, *J* = 4.9 Hz), 109.7 (d, *J* = 1.9 Hz), 32.7; **^{19}F NMR** (282 MHz, $CDCl_3$) δ -65.76 (s, 3F); **HRMS (ESI)**: calcd for $C_{13}H_{11}NOF_3$ $[M + H]^+$: 254.0793, found: 254.0800; **IR (KBr)**: 3042, 1633, 1590, 1490, 1329, 1250, 1198, 896, 808, 791cm^{-1}.

Ethyl 2-phenoxy-3*H*-azepine-5-carboxylate (3fa)

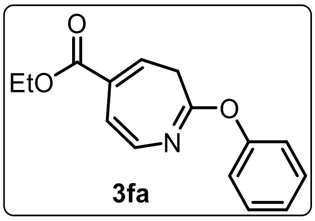

Following the general method **B** (ethyl 4-azidobenzoate **1f** (38.2 mg, 0.20 mmol), phenol **2a** (75.3 mg, 0.80 mmol), and DABCO (26.9 mg, 0.24 mmol) in 2 mL anhydrous DCE were used) compound **3fa** was obtained as a white solid, yield 60% (30.8 mg), mp: 49.7 – 51.1 °C; **^{1}H NMR** (500 MHz, $CDCl_3$) δ 7.37-7.31 (m, 2H), 7.20-7.15 (m, 1H), 7.03-6.98 (m, 3H), 6.57 (dd, *J* = 8.4, 1.0 Hz, 1H), 6.51 (ddd, *J* = 7.2, 4.0, 0.8 Hz, 1H), 4.28 (q, *J* = 7.1 Hz, 2H), 2.94 (d, *J* = 7.0 Hz, 2H), 1.34 (t, *J* = 7.1 Hz, 3H); **^{13}C NMR** (126 MHz, $CDCl_3$) δ 166.3, 153.0, 149.1, 138.6, 132.2, 129.5, 125.6, 124.5, 121.4, 113.1, 61.1, 33.4, 14.4; **HRMS (ESI)**: calcd for $C_{15}H_{16}NO_3$ $[M + H]^+$: 258.1130, found: 258.1139; **IR (KBr)**: 3056, 2984, 1714, 1632, 1590, 1488, 1318, 1197, 1071, 1038, 905, 806, 755 cm^{-1}.

5-(Pentafluoro-λ^6-sulfaneyl)-2-phenoxy-3*H*-azepine (3ha)

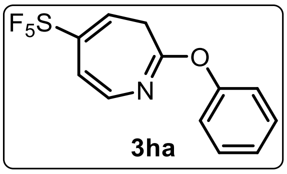

Following the general method **B** ((4-azidophenyl)pentafluoro-λ^6-sulfane **1h** (49.0 mg, 0.20 mmol), phenol **2a** (75.3 mg, 0.80 mmol), and DABCO (26.9 mg, 0.24 mmol) in 2 mL anhydrous DCE were used) compound **3ha** was obtained as a white solid, yield 83% (51.6 mg), mp: 95.6 – 97.8 °C; **^{1}H NMR** (500 MHz, $CDCl_3$) δ 7.40-7.35 (m, 2H), 7.24-7.19 (m, 1H)7.06 (d, *J* = 8.7 Hz, 1H), 7.03-6.99 (m, 2H), 6.38 (dd, *J* = 8.7, 1.1 Hz, 1H), 6.13 (t, *J* = 7.5 Hz, 1H), 2.90 (s, 2H); **^{13}C NMR** (126 MHz, $CDCl_3$) δ 154.4 (p, *J* = 15.4 Hz), 152.7, 150.7, 140.2, 129.7, 125.9, 121.3, 118.5-118.3 (m), 110.4-110.3 (m), 31.9; **^{19}F NMR** (282 MHz, $CDCl_3$) *δ* 85.05-82.79 (m, 1F), 61.60 (d, *J* = 149.0 Hz, 4F); **HRMS (ESI)**: calcd for $C_{12}H_{11}F_5NOS$ $[M + H]^+$: 312.0482, found: 312.0481; **IR (KBr)**: 3078, 2925, 1631, 1540, 1490, 1419, 1179, 1012, 954, 889, 805 cm^{-1}.

5-(Pentafluoro-λ^6-sulfaneyl)-2-(*p*-tolyloxy)-3*H*-azepine (3hb)

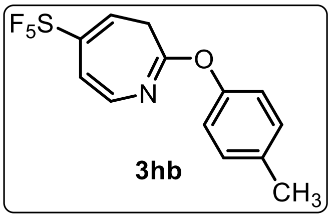

Following the general method **B** ((4-azidophenyl)pentafluoro-λ^6-sulfane **1h** (49.0 mg, 0.20 mmol), 4-methylphenol **2b** (86.5 mg, 0.80 mmol), and DABCO (26.9 mg, 0.24 mmol) in 2 mL anhydrous DCE were used) compound **3hb** was obtained as a white solid, yield 72% (46.8 mg), mp: 76.4 – 78.6 °C; **^{1}H NMR** (500 MHz, $CDCl_3$) δ 7.16 (d, *J* = 8.4 Hz, 2H), 7.06 (d, *J* = 8.6 Hz, 1H), 6.91-6.87 (m, 2H), 6.36 (dd, *J* = 8.7, 1.2 Hz, 1H), 6.11 (t, *J* = 7.5 Hz, 1H), 2.88 (s, 2H), 2.33 (s, 3H); **^{13}C NMR** (126 MHz, $CDCl_3$) δ 154.4 (p, *J* = 15.4 Hz), 151.1, 150.5, 140.4, 135.7, 130.2, 120.9, 118.5-118.3 (m), 110.3-110.1 (m), 31.9, 21.0; **^{19}F NMR** (282 MHz, $CDCl_3$) δ 84.64-82.38 (m, 1F) 61.58 (d, *J* = 149.0 Hz, 4F); **HRMS (ESI)**: calcd for $C_{13}H_{13}F_5NOS$ $[M + H]^+$: 326.0638, found: 326.0644; **IR (KBr):** 3073, 2930, 1632, 1602, 1531,1505, 1016, 1150, 1004, 940, 900, 886 cm^{-1}.

2-(4-Methoxyphenoxy)-5-(pentafluoro-λ^6-sulfaneyl)-3*H*-azepine (3hc)

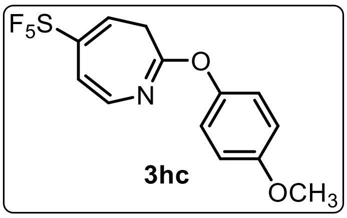

Following the general method **B** ((4-azidophenyl)pentafluoro-λ⁶-sulfane **1h** (49.0 mg, 0.20 mmol), 4-methoxyphenol **2c** (99.3 mg, 0.80 mmol), and DABCO (26.9 mg, 0.24 mmol) in 2 mL anhydrous DCE were used) compound **3hc** was obtained as a white solid, yield 75% (51.1 mg), mp: 73.2 – 74.9 °C; **^{1}H NMR** (300 MHz, $CDCl_3$) δ 7.06 (d, J = 8.7 Hz, 1H), 6.99-6.82 (m, 4H), 6.36 (d, J = 8.6 Hz, 1H), 6.11 (t, J = 7.4 Hz, 1H), 3.79 (s, 3H), 2.88 (d, J = 4.5 Hz, 2H); **^{13}C NMR** (126 MHz, $CDCl_3$) δ 157.3, 154.4 (p, J = 15.4 Hz), 151.4, 146.3, 140.4, 122.1, 118.6-118.4 (m), 114.7, 110.3-110.2 (m), 55.7, 31.9; **^{19}F NMR** (282 MHz, $CDCl_3$) δ 84.72-82.20 (m, 1F), 61.55 (d, J = 148.9 Hz, 4F); **HRMS (ESI)**: calcd for $C_{13}H_{13}F_5NO_2S$ $[M + H]^+$: 342.0587, found: 342.0588; **IR (KBr):** 3082, 2967, 1631, 1535, 1596, 1332, 1244, 1199, 903, 837, 812, 784 cm^{-1}.

2-(4-(*Tert*-butyl)phenoxy)-5-(pentafluoro-λ^6-sulfaneyl)-3*H*-azepine (3hd)

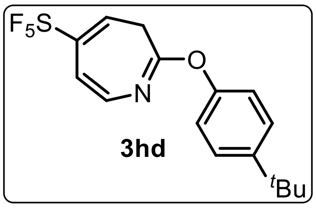

Following the general method **B** ((4-azidophenyl)pentafluoro-λ^6-sulfane **1h** (49.0 mg, 0.20 mmol), 4-*tert*-butylphenol **2d** (120.2 mg, 0.80 mmol), and DABCO (26.9 mg, 0.24 mmol) in 2 mL anhydrous DCE were used) compound **3hd** was obtained as a white solid, yield 72% (52.9 mg), mp: 66.0 – 68.2 °C; **^{1}H NMR** (500 MHz, $CDCl_3$) δ 7.39-7.35 (m, 2H), 7.08 (d, J = 8.6 Hz, 1H), 6.96-6.92 (m, 2H), 6.37 (dd, J = 8.7, 1.1 Hz, 1H), 6.12 (t, J = 7.5 Hz, 1H), 2.89 (s, 2H), 1.30 (s, 9H); **^{13}C NMR** (126 MHz, $CDCl_3$) δ 154.4 (p, J = 15.6 Hz), 150.8, 150.4, 148.7, 140.3, 126.6, 120.5, 118.5-118.3 (m), 110.4-110.2 (m), 34.6, 32.0, 31.5; **^{19}F NMR** (282 MHz, $CDCl_3$) δ 84.64-82.34 (m, 1F), 61.63 (d, J = 149.0 Hz, 4F); **HRMS (ESI)**: calcd for $C_{16}H_{19}F_5NOS$ $[M + H]^+$: 368.1108, found: 368.1122; **IR (KBr)**: 3087, 3040, 2963, 2910, 1634, 1598, 1536, 1330, 1254, 1181, 902, 813, 792 cm^{-1}.

5-(Pentafluoro-λ^6-sulfaneyl)-2-(*o*-tolyloxy)-3*H*-azepine (3he)

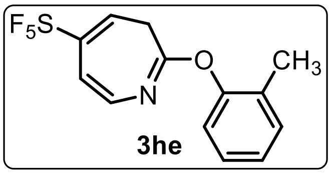

Following the general method **B** ((4-azidophenyl)pentafluoro-λ^6-sulfane **1h** (49.0 mg, 0.20 mmol), 2-methylphenol **2e** (86.5 mg, 0.80 mmol), and DABCO (26.9 mg, 0.24 mmol) in 2 mL anhydrous DCE were used) compound **3he** was obtained as a white solid, yield 77% (50.0 mg), mp: 85.0 – 86.6 °C; **^{1}H NMR** (500 MHz, $CDCl_3$) δ 7.24-7.18 (m, 2H), 7.14 (t, *J* = 7.4 Hz, 1H), 7.05 (d, *J* = 8.6 Hz, 1H), 6.97 (d, *J* = 7.9 Hz, 1H), 6.35 (d, *J* = 8.6 Hz, 1H), 6.13 (t, *J* = 7.5 Hz, 1H), 2.93 (s, 2H), 2.03 (s, 3H); **^{13}C NMR** (126 MHz, $CDCl_3$) δ 154.4 (p, *J* = 15.5 Hz), 151.2, 150.3, 140.4, 131.5, 129.9, 127.2, 126.4, 121.6, 118.7-118.4 (m), 110.0-109.8 (m), 31.8, 16.28; **^{19}F NMR** (282 MHz, $CDCl_3$) δ 84.54-82.29 (m, 1F), 61.51 (d, *J* = 148.9 Hz, 4F); **HRMS (ESI)**: calcd for $C_{13}H_{13}F_5NOS$ $[M + H]^+$: 326.0638, found: 326.0651; **IR (KBr)**: 3082, 2911, 1630, 1537, 1490, 1331, 1249, 1182, 889, 838, 775 cm^{-1}.

5-(Pentafluoro-λ^6-sulfaneyl)-2-(*m*-tolyloxy)-3*H*-azepine (3hf)

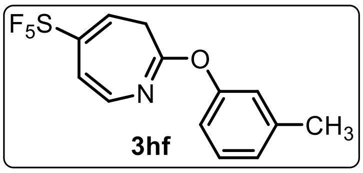

Following the general method **B** ((4-azidophenyl)pentafluoro-λ^6-sulfane **1h** (49.0 mg, 0.20 mmol), 3-methylphenol **2f** (86.5 mg, 0.80 mmol), and DABCO (26.9 mg, 0.24 mmol) in 2 mL anhydrous DCE were used) compound **3hf** was obtained as a white solid, yield 75% (48.7 mg), mp: 103.6 – 105.2 °C; **^{1}H NMR** (500 MHz, $CDCl_3$) δ 7.24 (t, *J* = 5.3 Hz, 1H), 7.07 (d, *J* = 8.7 Hz, 1H), 7.04-7.01(m, 1H), 6.83-6.78 (m, 2H), 6.37 (dd, *J* = 8.7, 1.3 Hz, 1H), 6.12 (t, *J* = 7.5 Hz, 1H), 2.89 (s, 2H), 2.35 (s, 3H); **^{13}C NMR** (126 MHz, $CDCl_3$) δ 154.5 (p, *J* = 15.4 Hz), 152.7, 150.8, 140.4, 139.9, 129.4, 126.8, 121.8, 118.5-118.3 (m), 118.3, 110.4-110.2 (m), 31.9, 21.5; **^{19}F NMR** (282 MHz, $CDCl_3$) δ 84.64-82.34 (m, 1F), 61.59 (d, *J* = 149.0 Hz, 4F); **HRMS (ESI)**: calcd for $C_{13}H_{13}F_5NOS$ $[M + H]^+$: 326.0638, found: 326.0643; **IR (KBr)**: 3089, 2930, 1632, 1613, 1539, 1330, 1255, 1178, 979, 877, 813 cm^{-1}.

2-(4-Fluorophenoxy)-5-(pentafluoro-λ^6-sulfaneyl)-3*H*-azepine (3hg)

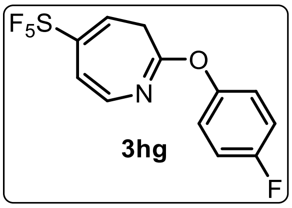

3hg

Following the general method **B** ((4-azidophenyl)pentafluoro-λ^6-sulfane **1h** (49.0 mg, 0.20 mmol), 4-fluorophenol **2g** (89.7 mg, 0.80 mmol), and DABCO (26.9 mg, 0.24 mmol) in 2 mL anhydrous DCE were used) compound **3hg** was obtained as a white solid, yield 60% (39.5 mg), mp: 43.8 – 45.6 °C; **^{1}H NMR** (500 MHz, $CDCl_3$) δ 7.08-7.02 (m, 3H), 7.01-6.96 (m, 2H), 6.39 (dd, *J* = 8.7, 1.2 Hz, 1H), 6.12 (t, *J* = 7.5 Hz, 1H), 2.90 (s, 2H); **^{13}C NMR** (126 MHz, $CDCl_3$) δ 160.2 (d, *J* = 244.6 Hz), 154.4 (p, *J* = 15.6 Hz), 150.9, 148.5 (d, *J* = 2.7 Hz), 140.1, 122.8 (d, *J* = 8.5 Hz), 118.7-118.5 (m), 116.3 (d, *J* = 23.6 Hz), 110.7-110.5 (m), 31.8; **^{19}F NMR** (282 MHz, $CDCl_3$) δ 84.47-82.23 (m, 1F), 61.58 (d, *J* = 149.0 Hz, 4F), -117.16 to -117.29 (m, 1F); **HRMS (ESI)**: calcd for $C_{12}H_{10}F_6NOS$ $[M + H]^+$: 330.0387, found: 330.0388; **IR (KBr)**: 3114, 3066, 2913, 1630, 1539, 1334, 1252, 1189, 906, 836, 805, 780 cm^{-1}.

2-(4-Chlorophenoxy)-5-(pentafluoro-λ^6-sulfaneyl)-3*H*-azepine (3hh)

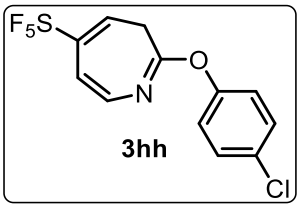

3hh

Following the general method **B** ((4-azidophenyl)pentafluoro-λ^6-sulfane **1h** (49.0 mg, 0.20 mmol), 4-chlorophenol **2h** (123.7 mg, 0.80 mmol), and DABCO (26.9 mg, 0.24 mmol) in 2 mL anhydrous DCE were used) compound **3hh** was obtained as a white solid, yield 55% (38.0 mg), mp: 71.4 – 73.1 °C; **^{1}H NMR (**500 MHz, $CDCl_3$) δ 7.35-7.31 (m, 2H), 7.05 (d, *J* = 8.6 Hz, 1H), 6.99-6.95 (m, 2H), 6.40 (dd, *J* = 8.7, 1.2 Hz, 1H), 6.12 (t, *J* = 7.5 Hz, 1H), 2.90 (s, 2H); **^{13}C NMR** (126 MHz, $CDCl_3$) δ 154.5 (p, *J* = 15.6 Hz), 151.2, 150.5, 139.9, 131.3, 129.7, 122.7, 118.7-118.5 (m), 110.9-110.6 (m), 31.8; **^{19}F NMR** (282 MHz, $CDCl_3$) δ 84.37-82.13 (m, 1F), 61.57 (d, *J* = 149.0 Hz, 4F); **HRMS (ESI)**: calcd for $C_{12}H_{10}F_5ClNOS$ $[M + H]^+$: 346.0092, found: 346.0088; **IR (KBr)**: 3086, 2927, 1634, 1541, 1485, 1330, 1201, 940, 904, 886, 844, 819 cm^{-1}.

2-(4-Bromophenoxy)-5-(pentafluoro-λ^6-sulfaneyl)-3*H*-azepine (3hi)

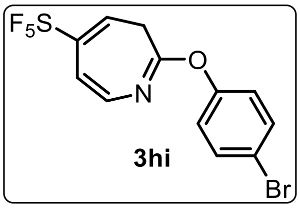

Following the general method **B** ((4-azidophenyl)pentafluoro-λ^6-sulfane **1h** (49.0 mg, 0.20 mmol), 4-bromophenol **2i** (138.4 mg, 0.80 mmol), and DABCO (26.9 mg, 0.24 mmol) in 2 mL anhydrous DCE (0.1 M) were used) compound **3hi** was obtained as a pale yellow solid, yield 65% (50.7 mg), mp: 83.3 – 85.6 °C; **^{1}H NMR** (500 MHz, $CDCl_3$) δ 7.52-7.46 (m, 2H), 7.05 (d, J = 8.5 Hz, 1H), 6.95-6.89 (m, 2H), 6.40 (dd, J = 8.6, 1.4 Hz, 1H), 6.13 (t, J = 7.4 Hz, 1H), 2.90 (s, 2H); **^{13}C NMR** (126 MHz, $CDCl_3$) δ 154.4 (p, J = 15.6 Hz), 151.7, 150.4, 139.9, 132.7, 123.2, 119.1, 118.6, 110.8, 31.8; **^{19}F NMR** (282 MHz, $CDCl_3$) δ 84.34-82.10 (m, 1F), 61.58 (d, J = 149.5 Hz, 4F); **HRMS (ESI)**: calcd for $C_{12}H_{10}BrF_5NOS$ $[M + H]^+$: 389.9587, found: 389.9595; **IR (KBr)**: 3090, 2899, 1632, 1536, 1482, 1321, 1246, 1198, 1011, 906, 889, 827, 792 cm^{-1}.

2-(4-Iodophenoxy)-5-(pentafluoro-λ^6-sulfaneyl)-3*H*-azepine (3hj)

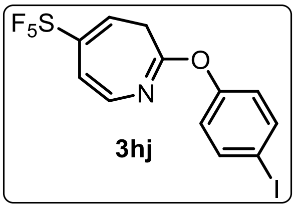

Following the general method **B** ((4-azidophenyl)pentafluoro-λ^6-sulfane **1h** (49.0 mg, 0.20 mmol), 4-iodophenol **2j** (176.0 mg, 0.80 mmol), and DABCO (26.9 mg 0.24 mmol) in 2 mL anhydrous DCE were used) compound **3hj** was obtained as a white solid, yield 67% (58.5 mg), mp: 114.3 – 116.3 °C; **^{1}H NMR** (500 MHz, $CDCl_3$) δ 7.68 (dd, J = 8.8, 2.1 Hz, 2H), 7.06 (d, J = 8.6 Hz, 1H), 6.82-6.78 (m, 2H), 6.40 (d, J = 8.7 Hz, 1H), 6.13 (t, J = 7.5 Hz, 1H), 2.90 (s, 2H); **^{13}C NMR** (126 MHz, $CDCl_3$) δ 154.4 (p, J = 15.6 Hz), 152.6, 150.3, 139.9, 138.7, 123.6, 118.6, 110.9-110.5 (m), 90.1, 31.8; **^{19}F NMR** (282 MHz, $CDCl_3$) δ 85.44-83.02 (m, 1F), 62.57 (d, J = 149.1 Hz, 4F); **HRMS (ESI)**: calcd for $C_{12}H_{10}IF_5NOS$ $[M + H]^+$: 437.9448, found: 437.9453; **IR (KBr)**: 3087, 2925, 1630, 1574, 1535, 1479, 1421, 1320, 1269, 1173, 1008, 904, 826, 789 cm^{-1}.

5-(Pentafluoro-λ^6-sulfaneyl)-2-(4-(trifluoromethyl)phenoxy)-3*H*-azepine (3hk)

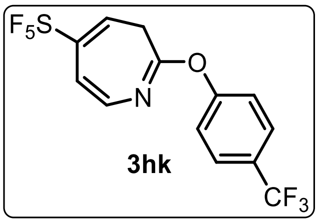

Following the general method **B** ((4-azidophenyl)pentafluoro-λ^{6}-sulfane **1h** (49.0 mg, 0.20 mmol), 4-trifluoromethylphenol **2k** (1292.7 mg, 0.80 mmol), and DABCO (26.9 mg, 0.24 mmol) in 2 mL anhydrous DCE were used) compound **3hk** was obtained as a white solid, yield 54% (40.9 mg), mp: 72.1 – 73.7 °C; **^{1}H NMR** (500 MHz, $CDCl_3$) δ 7.64 (d, *J* = 8.5 Hz, 2H), 7.15 (d, *J* = 8.4 Hz, 2H), 7.06 (d, *J* = 8.7 Hz, 1H), 6.43 (dd, *J* = 8.7, 1.2 Hz, 1H), 6.15 (t, *J* = 7.5 Hz, 1H), 2.93 (s, 2H); **^{13}C NMR** (126 MHz, $CDCl_3$) δ 155.1, 154.5 (p, *J* = 15.7 Hz), 149.9, 139.8, 128.2 (q, *J* = 32.9 Hz), 127.1 (d, *J* = 3.6 Hz), 123.9 (q, *J* = 272.0 Hz), 121.9, 118.8-118.5 (m), 111.2-110.9 (m), 31.8; **^{19}F NMR** (282 MHz, $CDCl_3$) δ 84.26-81.93 (m, 1F), 61.61 (d, *J* = 149.1 Hz, 4F), -62.75 (s, 3F); **HRMS (ESI)**: calcd for $C_{13}H_{10}F_8NOS$ $[M + H]^+$: 380.0355, found: 380.0358; **IR (KBr)**: 3089, 2906, 1636, 1465, 1323, 1125, 1063, 905, 821, 783 cm^{-1}.

2-(4-Nitrophenoxy)-5-(pentafluoro-λ^{6}-sulfaneyl)-3*H*-azepine (3hl)

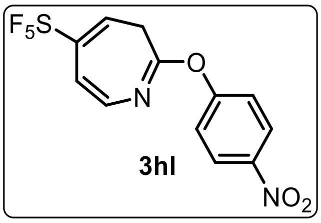

Following the general method **B** ((4-azidophenyl)pentafluoro-λ^{6}-sulfane **1h** (49.0 mg, 0.20 mmol), 4-nitrophenol **2l** (111.3 mg, 0.80 mmol), and DABCO (26.9 mg, 0.24 mmol) in 2 mL anhydrous DCE were used) compound **3hl** was obtained as a white solid, yield 19% (13.5 mg), mp: 81.4 – 82.9 °C; **^{1}H NMR** (500 MHz, $CDCl_3$) δ 8.28-8.25 (m, 2H), 7.23-7.19 (m, 2H), 7.05 (d, *J* = 8.7 Hz, 1H), 6.47 (dd, *J* = 8.7, 1.2 Hz, 1H), 6.16 (t, *J* = 7.4 Hz, 1H), 2.95 (s, 2H); **^{13}C NMR** (126 MHz, $CDCl_3$) δ 157.3, 154.5 (p, *J* = 15.6 Hz), 149.3, 145.3, 139.5, 125.5, 122.3, 118.9-118.5 (m), 111.6-111.3 (m), 31.6; **^{19}F NMR** (282 MHz, $CDCl_3$) δ 84.07-81.83 (m, 1F), 61.62 (d, J = 149.1 Hz, 4F); **HRMS (ESI)**: calcd for $C_{12}H_{10}F_5N_2O_3S$ $[M + H]^+$: 357.0332, found: 357.0334; **IR (KBr)**: 3097, 2920, 1631, 1522, 1312, 1246, 1213, 904, 873, 865, 798 cm^{-1}.

5-(pentafluoro-λ^{6}-sulfaneyl)-2-(4-((trifluoromethyl)thio)phenoxy)-3*H*-azepine (3hm)

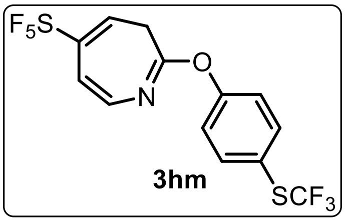

Following the general method **B** ((4-azidophenyl)pentafluoro-λ^6-sulfane **1h** (49.0 mg, 0.20 mmol), 4-(trifluoromethylthio)phenol **2m** (155.3 mg, 0.80 mmol), and DABCO (26.9 mg, 0.24 mmol) in 2 mL anhydrous DCE were used) compound **3hm** was obtained as a yellow oil, yield 60% (49.3 mg), **^{1}H NMR** (500 MHz, $CDCl_3$) δ 7.61-7.56 (m, 2H), 7.05-7.00 (m, 2H), 6.98 (d, *J* = 8.7 Hz, 1H), 6.35 (dd, *J* = 8.7, 1.1 Hz, 1H), 6.06 (t, *J* = 7.5 Hz, 1H), 2.84 (s, 2H); **^{13}C NMR** (126 MHz, $CDCl_3$) δ 154.8, 154.5 (p, *J* = 15.8 Hz), 149.8, 139.8, 137.9, 129.6 (q, *J* = 308.2 Hz), 122.5, 121.5 (d, *J* = 1.8 Hz), 118.7-118.5 (m), 111.1-110.9 (m), 31.8; **^{19}F NMR** (376 MHz, $CDCl_3$) δ 84.02-82.38 (m, 1F), 61.69 (d, *J* = 149.0 Hz, 4F), -43.31 (s, 3F); **HRMS (ESI)**: calcd for $C_{13}H_{10}F_8NOS$ [M + H]$^+$: 412.0076, found: 412.0081; **IR (KBr)**: 3077, 3023, 2960, 2929, 1637, 1586, 1487, 1328, 1248, 1118, 901, 847, 819 cm^{-1}.

Ethyl 4-((5-(pentafluoro-λ^6-sulfaneyl)-3*H*-azepin-2-yl)oxy)benzoate (3hn)

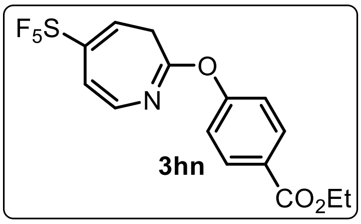

Following the general method **B** ((4-azidophenyl)pentafluoro-λ^6-sulfane **1h** (49.0 mg, 0.20 mmol), ethyl 4-hydroxybenzoate **2n** (132.9 mg, 0.80 mmol), and DABCO (26.9 mg, 0.24 mmol) in 2 mL anhydrous DCE were used) compound **3hn** was obtained as a white solid, yield 55% (42.1 mg), mp: 69.6 – 71.0 °C; **^{1}H NMR** (500 MHz, $CDCl_3$) δ 8.12-8.03 (m, 2H), 7.13-7.02 (m, 3H), 6.42 (dd, *J* = 8.7, 1.2 Hz, 1H), 6.14 (t, *J* = 7.4 Hz, 1H), 4.36 (q, *J* = 7.1 Hz, 2H), 2.92 (s, 2H), 1.38 (t, *J* = 7.1 Hz, 3H); **^{13}C NMR** (126 MHz, $CDCl_3$) δ 165.8, 154.5 (p, *J* = 15.6 Hz), 156.2, 149.9, 139.9, 131.7, 128.1, 121.3, 118.7-118.4 (m), 110.9-110.7 (m), 61.2, 31.8, 14.4; **^{19}F NMR** (282 MHz, $CDCl_3$) δ 84.47-82.00 (m, 1F), 61.60 (d, *J* = 149.1 Hz, 4F); **HRMS (ESI)**: calcd for $C_{15}H_{15}F_5NO_3S$ [M + H]$^+$: 384.0693, found: 384.0704; **IR (KBr)**: 3067, 2991, 2944, 1632, 1538, 1333, 1246, 1202, 1173, 1162, 892, 833, 780 cm^{-1}.

2-([1,1'-Biphenyl]-4-yloxy)-5-(pentafluoro-λ^6-sulfaneyl)-3*H*-azepine (3ho)

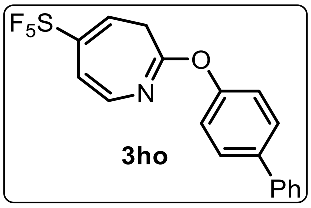

3ho

Following the general method **B** ((4-azidophenyl)pentafluoro-λ^6-sulfane **1h** (49.0 mg, 0.20 mmol), 4-phenylphenol **2o** (136.2 mg, 0.80 mmol), and DABCO (26.9 mg, 0.24 mmol) in 2 mL anhydrous DCE were used) compound **3ho** was obtained as a white solid, yield 61% (47.2 mg), mp: 102.4 – 104.1 °C; **^{1}H NMR** (500 MHz, $CDCl_3$) δ 7.60-7.52 (m, 4H), 7.43 (dd, *J* = 10.6, 4.9 Hz, 2H), 7.37-7.31 (m, 1H), 7.12-7.06 (m, 3H), 6.40 (dd, *J* = 8.7, 1.0 Hz, 1H), 6.15 (t, *J* = 7.5 Hz, 1H), 2.93 (s, 2H); **^{13}C NMR** (126 MHz, $CDCl_3$) δ 154.5 (p, *J* = 15.7 Hz), 152.1, 150.8, 140.4, 140.2, 139.1, 128.9, 128.4, 127.5, 127.2, 121.5, 118.7-118.4 (m), 110.6-110.4 (m), 31.9; **^{19}F NMR** (282 MHz, $CDCl_3$) δ 84.51-82.27 (m, 1F), 61.62 (d, *J* = 149.1 Hz, 4F); **HRMS (ESI)**: calcd for $C_{18}H_{15}F_5NOS$ $[M + H]^+$: 388.0795, found: 388.0799; **IR (KBr)**: 3087, 3068, 2959, 1633, 1596, 1537, 1485, 1320, 1252, 1172, 902, 817 cm^{-1}.

5-(Pentafluoro-λ^6-sulfaneyl)-2-(4-phenoxyphenoxy)-3*H*-azepine (3hp)

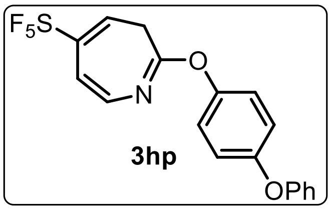

3hp

Following the general method **B** ((4-azidophenyl)pentafluoro-λ^6-sulfane **1h** (49.0 mg, 0.20 mmol), 4-phenoxyphenol **2p** (148.9 mg, 0.80 mmol), and DABCO (26.9 mg, 0.24 mmol) in 2 mL anhydrous DCE were used) compound **3hp** was obtained as a white solid, yield 58% (46.7 mg), mp: 58.4 – 60.1 °C; **^{1}H NMR** (500 MHz, $CDCl_3$) δ 7.36-7.31 (m, 2H), 7.13-7.06 (m, 2H), 7.03-6.99 (m, 2H), 6.98 (s, 4H), 6.38 (dd, *J* = 8.7, 1.2 Hz, 1H), 6.12 (t, *J* = 7.5 Hz, 1H), 2.89 (s, 2H); **^{13}C NMR** (126 MHz, $CDCl_3$) δ 154.4 (p, *J* = 15.6 Hz), 157.1, 154.9, 150.9, 148.1, 140.2, 129.9, 123.7, 122.4, 119.5, 119.2, 118.6-118.4 (m), 110.6-110.3 (m), 31.9; **^{19}F NMR** (282 MHz, $CDCl_3$) δ 84.59-82.20 (m, 1F), 61.58 (d, *J* = 149.0 Hz, 4F); **HRMS (ESI)**: calcd for $C_{18}H_{15}F_5NO_2S$ $[M + H]^+$: 404.0744, found: 404.0750; **IR (KBr)**: 3087, 2961, 2946, 2097, 1613, 1587, 1538, 1421, 1322, 1274, 1174, 1081, 1009, 906, 887, 806 cm^{-1}.

2-(Naphthalen-1-yloxy)-5-(pentafluoro-λ^6-sulfaneyl)-3*H*-azepine (3hq)

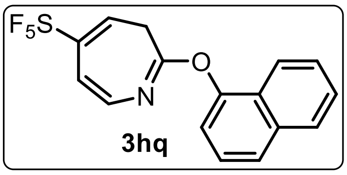

3hq

Following the general method **B** ((4-azidophenyl)pentafluoro-λ^6-sulfane **1h** (49.0 mg, 0.20 mmol), 1-napthol **2q** (115.3 mg, 0.80 mmol), and DABCO (26.9 mg, 0.24 mmol) in 2 mL anhydrous DCE were used) compound **3hq** was obtained as a white solid, yield 45% (32.5 mg), mp: 131.3 – 133.5 °C; ^{1}**H NMR** (500 MHz, $CDCl_3$) δ 7.88-7.85 (m, 1H), 7.74 (d, *J* = 8.3 Hz, 1H), 7.68 (dd, *J* = 8.2, 0.8 Hz, 1H), 7.51-7.43 (m, 3H), 7.17 (dd, *J* = 7.5, 0.9 Hz, 1H), 6.99 (d, *J* = 8.6 Hz, 1H), 6.35 (dd, *J* = 8.6, 1.2 Hz, 1H), 6.27 (t, *J* = 7.5 Hz, 1H), 3.06 (s, 2H); ^{13}**C NMR** (126 MHz, $CDCl_3$) δ 154.6 (p, *J* = 15.4 Hz), 150.7, 148.6, 140.2, 134.9, 128.3, 126.6, 126.6, 126.4, 126.3, 125.5, 121.4, 118.7-118.5 (m), 110.3-110.1 (m), 31.9; ^{19}**F NMR** (282 MHz, $CDCl_3$) δ 85.48-83.28 (m, 1F), 62.62 (d, *J* = 149.0 Hz, 4F); **HRMS (ESI)**: calcd for $C_{16}H_{13}F_5NOS$ $[M + H]^+$: 362.0638, found: 362.0641; **IR (KBr)**: 3073, 2922, 1628, 1599, 1533, 1325, 1314, 1245, 1224, 1172, 1006, 972, 948, 889, 819 cm^{-1}.

8-((5-(Pentafluoro-λ^6-sulfaneyl)-3*H*-azepin-2-yl)oxy)quinoline (3hr)

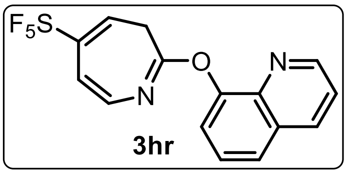

3hr

Following the general method **B** ((4-azidophenyl)pentafluoro-λ^6-sulfane **1h** (49.0 mg, 0.20 mmol), 8-hydroxyquinoline **2r** (116.1 mg, 0.80 mmol), and DABCO (26.9 mg, 0.24 mmol) in 2 mL anhydrous DCE were used) compound **3hr** was obtained as a brown solid, yield 36% (26.0 mg), mp: 136.2 – 138.5 °C; ^{1}**H NMR** (500 MHz, $CDCl_3$) δ 8.79 (dd, *J* = 4.1, 1.5 Hz, 1H), 8.14 (dd, *J* = 8.3, 1.5 Hz, 1H), 7.70 (dd, *J* = 8.2, 1.0 Hz, 1H), 7.53 (t, *J* = 7.9 Hz, 1H), 7.40 (m, 2H), 6.91 (d, *J* = 8.7 Hz, 1H), 6.30 (t, *J* = 7.8 Hz, 2H), 3.11 (s, 2H); ^{13}**C NMR** (126 MHz, $CDCl_3$) δ 153.8 (p, *J* = 15.2 Hz), 151.3, 150.3, 149.1, 140.6, 139.8, 136.0, 129.8, 126.3, 126.1, 121.9, 121.0, 119.6-119.4 (m), 110.2-109.9 (m), 31.9; ^{19}**F NMR** (376 MHz, CDCl3) δ 84.89 – 83.21 (m, 1F), 61.72 (d, J = 148.9 Hz, 4F); **HRMS (ESI)**: calcd for $C_{15}H_{12}F_5N_2OS$ $[M + H]^+$: 363.0590, found: 363.0602; **IR (KBr)**: 3086, 2917, 1631, 1325, 1309, 1240, 1171, 849, 829, 813, 779 cm^{-1}.

2-(3,5-Bibromophenoxy)-5-(pentafluoro-λ^6-sulfaneyl)-3*H*-azepine (3hs)

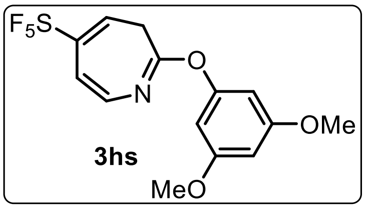

Following the general method **B** ((4-azidophenyl)pentafluoro-λ^6-sulfane **1h** (49.0 mg, 0.20 mmol), 3,5-dimethoxyphenol **2s** (123.3 mg, 0.80 mmol), and DABCO (26.9 mg, 0.24 mmol) in 2 mL anhydrous DCE were used) compound **3hs** was obtained as a white solid, yield 68% (50.4 mg), mp: 92.9 – 94.9 °C; ^{1}**H NMR** (500 MHz, $CDCl_3$) δ 7.10 (d, J = 8.6 Hz, 1H), 6.39 (dd, J = 8.7, 1.2 Hz, 1H), 6.31 (t, J = 2.2 Hz, 1H), 6.16 (d, J = 2.2 Hz, 2H), 6.11 (t, J = 7.5 Hz, 1H), 3.75 (s, 6H), 2.87 (s, 2H); ^{13}**C NMR** (126 MHz, $CDCl_3$) δ 161.3, 154.4, 154.3, 150.1, 140.2, 118.4-118.2 (m), 110.5-110.2 (m), 99.9, 98.1, 55.5, 31.9; ^{19}**F NMR** (282 MHz, $CDCl_3$) δ 84.63-82.19 (m, 1F), 61.62 (d, J = 149.0 Hz, 4F); **HRMS (ESI)**: calcd for $C_{14}H_{15}F_5NO_3S$ [M + H]$^+$: 372.0693, found: 372.0702; **IR (KBr)**: 3421, 3004, 2966, 2943, 1630, 1614, 1588, 1333, 1255, 1157, 886, 805 cm^{-1}.

2-(3,5-Dibromophenoxy)-5-(pentafluoro-λ^6-sulfaneyl)-3*H*-azepine (3ht)

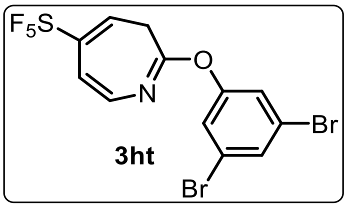

Following the general method **B** ((4-azidophenyl)pentafluoro-λ^6-sulfane **1h** (49.0 mg, 0.20 mmol), 3,5-dibromophenol **2t** (123.3 mg, 0.80 mmol), and DABCO (26.9 mg, 0.24 mmol) in 2 mL anhydrous DCE were used) compound **3ht** was obtained as a white solid, yield 60% (56.2 mg), mp: 60.5 – 62.1 °C; ^{1}**H NMR** (500 MHz, $CDCl_3$) δ 7.52 (t, J = 1.6 Hz, 1H), 7.18 (d, J = 1.6 Hz, 2H), 7.05 (d, J = 8.7 Hz, 1H), 6.43 (dd, J = 8.7, 1.0 Hz, 1H), 6.12 (t, J = 7.4 Hz, 1H), 2.88 (d, J = 3.1 Hz, 2H); ^{13}**C NMR** (126 MHz, $CDCl_3$) δ 154.5 (p, J = 15.7 Hz), 153.3, 149.8, 139.7, 131.8, 123.9, 122.9, 118.8-118.5 (m), 111.4-111.1 (m), 31.5; ^{19}**F NMR** (376 MHz, $CDCl_3$) δ 83.86-82.19 (m, 1F), 61.62 (d, J = 149.0 Hz, 4F); **HRMS (ESI)**: calcd for $C_{12}H_9Br_2F_5NOS$ [M + H]$^+$: 467.8692, found: 467.8695; **IR (KBr):** 3095, 2966, 2943, 1630, 1614, 1588, 1334, 1195, 887, 805, 725 cm^{-1}.

Methyl 2-((5-(pentafluoro-λ^6-sulfaneyl)-3*H*-azepin-2-yl)oxy)benzoate (3hu)

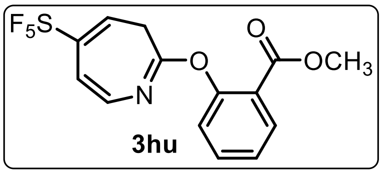

Following the general method **B** ((4-azidophenyl)pentafluoro-λ^6-sulfane **1h** (49.0 mg, 0.20 mmol), methyl salicylate **2u** (121.7 mg, 0.80 mmol), and DABCO (26.9 mg, 0.24 mmol) in 2 mL anhydrous DCE were used) compound **3hu** was obtained as a colourless oil, yield 47% (34.7 mg), **^{1}H NMR** (500 MHz, $CDCl_3$) δ 7.97 (dd, *J* = 7.8, 1.7 Hz, 1H), 7.57 (ddd, *J* = 8.1, 7.6, 1.7 Hz, 1H), 7.31 (td, *J* = 7.7, 1.1 Hz, 1H), 7.11 (dd, *J* = 8.1, 1.0 Hz, 1H), 6.96 (d, *J* = 8.7 Hz, 1H), 6.33 (dd, *J* = 8.7, 1.1 Hz, 1H), 6.18 (t, *J* = 7.4 Hz, 1H), 3.78 (s, 3H), 2.97 (d, *J* = 3.4 Hz, 2H); **^{13}C NMR** (126 MHz, $CDCl_3$) δ 164.7, 153.9 (p, *J* = 15.6 Hz), 152.2, 151.5, 139.9, 133.9, 131.8, 126.2, 123.6, 123.4, 119.4-119.2 (m), 110.8-110.5 (m), 52.2, 31.5; **^{19}F NMR** (282 MHz, $CDCl_3$) δ 85.86-83.64 (m, 1F), 62.47 (d, *J* = 149.1 Hz, 4F); **HRMS (ESI)**: calcd for $C_{14}H_{13}F_5NO_3S$ $[M + H]^+$: 370.0536, found: 370.0551; **IR (KBr):** 3081, 2955, 2928, 1729, 1637, 1331, 1249, 1083, 903, 885, 844, 811, 782.cm^{-1}.

2-Ethylhexyl 2-((5-(pentafluoro-λ^6-sulfaneyl)-3*H*-azepin-2-yl)oxy)benzoate (3hv)

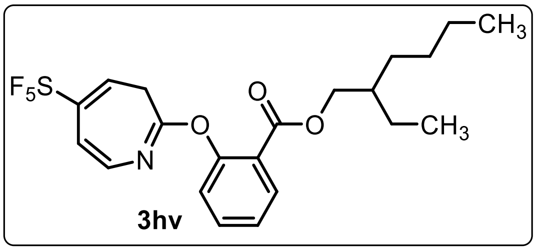

Following the general method **B** ((4-azidophenyl)pentafluoro-λ^6-sulfane **1h** (49.0 mg, 0.20 mmol), ethylhexyl salicylate **2v** (200.3 mg, 0.80 mmol), and DABCO (26.9 mg, 0.24 mmol) in 2 mL anhydrous DCE were used) compound **3hv** was obtained as a colourless oil, yield 34% (31.7 mg), **^{1}H NMR** (500 MHz, $CDCl_3$) δ 7.95 (dd, *J* = 7.8, 1.7 Hz, 1H), 7.58-7.53 (m, 1H), 7.30 (td, *J* = 7.7, 1.1 Hz, 1H), 7.06 (dd, *J* = 8.1, 1.0 Hz, 1H), 6.96 (d, *J* = 8.7 Hz, 1H), 6.33 (dd, *J* = 8.7, 1.1 Hz, 1H), 6.18 (t, *J* = 7.4 Hz, 1H), 4.11 (qd, *J* = 11.0, 5.8 Hz, 2H), 2.96 (d, *J* = 4.3 Hz, 2H), 1.69-1.61 (m, 1H), 1.46-1.38 (m, 2H), 1.37-1.32 (m, 2H), 1.32-1.25 (m, 4H), 0.93-0.87 (m, 6H); **^{13}C NMR** (126 MHz, $CDCl_3$) δ 164.2, 153.9 (p, *J* = 15.4 Hz), 152.3, 151.3, 139.9, 133.8, 131.6, 126.1, 123.8, 123.6, 119.5-119.2 (m), 110.6, 67.4, 38.9, 31.6, 30.6, 29.0, 23.9, 23.1, 14.2, 11.1; **^{19}F NMR** (282 MHz, $CDCl_3$) δ 84.92-82.57 (m, 1F), 61.52 (d, *J* = 149.1 Hz, 4F); **HRMS (ESI)**: calcd for $C_{21}H_{27}F_5NO_3S$ $[M + H]^+$: 468.1632, found: 468.1647; **IR (KBr):** 3082, 2962, 2932, 1725, 1637, 1330, 1250, 1206, 1080, 903, 847, 812, 781 cm^{-1}.

2-(4-(Benzyloxy)phenoxy)-5-(pentafluoro-λ^6-sulfaneyl)-3*H*-azepine (3hw)

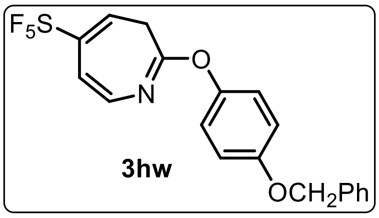

Following the general method **B** ((4-azidophenyl)pentafluoro-λ^6-sulfane **1h** (49.0 mg, 0.20 mmol), 4-(benzyloxy)phenol **2w** (160.2 mg, 0.80 mmol), and DABCO (26.9 mg, 0.24 mmol) in 2 mL anhydrous DCE were used) compound **3hw** was obtained as a white solid, yield 49% (41.1 mg), mp: 89.7 – 91.5 °C; **^{1}H NMR** (500 MHz, $CDCl_3$) δ 7.44-7.36 (m, 4H), 7.36-7.30 (m, 1H), 7.06 (d, *J* = 8.6 Hz, 1H), 6.99-6.90 (m, 4H), 6.36 (dd, *J* = 8.7, 1.2 Hz, 1H), 6.11 (t, *J* = 7.4 Hz, 1H), 5.03 (s, 2H), 2.88 (s, 2H); **^{13}C NMR** (126 MHz, $CDCl_3$) δ 156.5, 154.4 (p, *J* = 15.3 Hz), 151.3, 146.5, 140.3, 136.9, 128.7, 128.2, 127.6, 122.1, 118.6-118.3 (m), 115.6, 110.5-109.4 (m), 70.5, 31.9; **^{19}F NMR** (282 MHz, $CDCl_3$) δ 84.72-82.20 (m, 1F), 61.56 (d, *J* = 148.9 Hz, 4F); **HRMS (ESI)**: calcd for $C_{19}H_{17}F_5NO_2S$ $[M + H]^+$: 418.0900, found: 418.0908; **IR (KBr):** 3081, 2943, 2373, 1629, 1594, 1504, 1420, 1387, 1236, 1195, 902, 810, 782 cm^{-1}.

6-((5-(Pentafluoro-λ^6-sulfaneyl)-3*H*-azepin-2-yl)oxy)-2*H*-chromen-2-one (3hx)

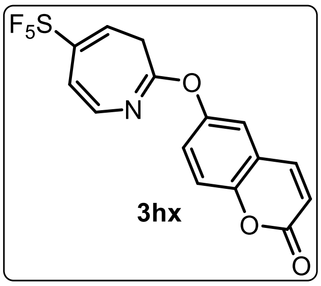

Following the general method **B** ((4-azidophenyl)pentafluoro-λ^6-sulfane **1h** (49.0 mg, 0.20 mmol), 6-hydroxycoumarin **2x** (129.7 mg, 0.80 mmol), and DABCO (26.9 mg, 0.24 mmol) in 2 mL anhydrous DCE were used) compound **3hx** was obtained as a white solid, yield 40% (30.3 mg), mp: 137.2 – 139.9 °C; **^{1}H NMR** (500 MHz, $CDCl_3$) δ 7.66 (d, *J* = 9.6 Hz, 1H), 7.34 (dd, *J* = 8.0, 1.9 Hz, 1H), 7.20 (dd, *J* = 8.0, 2.3 Hz, 2H), 7.04 (d, *J* = 8.7 Hz, 1H), 6.46 (d, *J* = 9.6 Hz, 1H), 6.42 (dd, *J* = 8.7, 1.2 Hz, 1H), 6.16 (t, *J* = 7.4 Hz, 1H), 2.94 (s, 2H); **^{13}C NMR** (126 MHz, $CDCl_3$) δ 160.5, 154.5 (p, *J* = 15.8 Hz), 151.6, 150.7, 148.7, 142.9, 139.8, 125.3, 119.8, 119.4, 118.9-118.6 (m), 118.2, 117.6, 111.1-110.9 (m), 31.7; **^{19}F NMR** (282 MHz, $CDCl_3$) δ 84.29-82.04 (m, 1F), 61.58 (d, *J* = 149.1 Hz, 4F); **HRMS (ESI)**: calcd for

$C_{15}H_{11}F_5NO_3S$ [M + H]$^+$: 380.0380, found: 380.0388; **IR (KBr):** 3092, 3046, 2925, 2905, 1732, 1695, 1630, 1323, 1253, 1174, 1103, 889, 846 cm^{-1}.

2-(2,6-Diisopropylphenoxy)-5-(pentafluoro-λ^6-sulfaneyl)-3*H*-azepine (3hy)

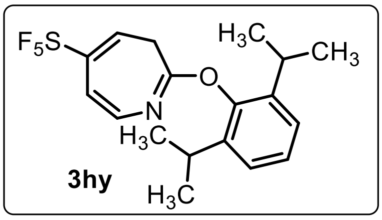

Following the general method **B** ((4-azidophenyl)pentafluoro-λ^6-sulfane **1h** (49.0 mg, 0.20 mmol), 2,6-diisopropylphenol **2y** (142.6 mg, 0.80 mmol), and DABCO (26.9 mg, 0.24 mmol) in 2 mL anhydrous DCE were used) compound **3hy** was obtained as a white solid, yield 50% (39.5 mg), mp: 131.2 – 132.0 °C; **^{1}H NMR** (500 MHz, $CDCl_3$) δ 7.22 (dd, *J* = 8.3, 7.0 Hz, 1H), 7.16-7.12 (m, 2H), 7.03 (d, *J* = 8.6 Hz, 1H), 6.34 (dd, *J* = 8.6, 1.2 Hz, 1H), 6.15 (t, *J* = 7.5 Hz, 1H), 2.97 (s, 2H), 2.84 (dt, *J* = 13.4, 6.6 Hz, 2H), 1.13 (s, 12H); **^{13}C NMR** (126 MHz, $CDCl_3$) δ 154.5 (p, *J* = 15.6 Hz), 150.3, 147.0, 140.5, 140.1, 126.9, 124.3, 118.4-118.0 (m), 109.8-109.6 (m), 31.7, 27.3, 23.3 (d, *J* = 119.9 Hz); **^{19}F NMR** (282 MHz, $CDCl_3$) δ 85.86-83.34 (m, 1F) 62.54 (d, *J* = 148.8, 4F); **HRMS (ESI)**: calcd for $C_{18}H_{23}F_5NOS$ [M + H]$^+$: 396.1421, found: 396.1431; **IR (KBr):** 3083, 2974, 2933, 1622, 1585, 1532, 1322, 1270, 1181, 888, 839, 816, 788 cm^{-1}.

5-(Pentafluoro-λ^6-sulfaneyl)-2-(4-(2,4,4-trimethylpentan-2-yl)phenoxy)-3*H*-azepine (3hz)

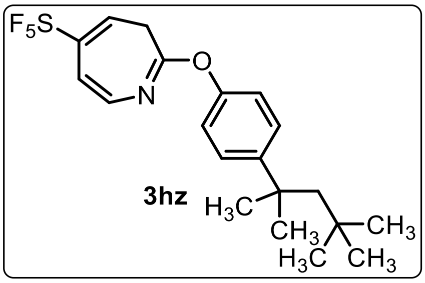

Following the general method **B** ((4-azidophenyl)pentafluoro-λ^6-sulfane **1h** (49.0 mg, 0.20 mmol), 4-(2,4,4-trimethylpentan-2-yl)phenol **2z** (165.1 mg, 0.80 mmol), and DABCO (26.91 mg, 0.24 mmol) in 2 mL anhydrous DCE were used) compound **3hz** was obtained as a colourless oil, yield 58% (49.1 mg), **^{1}H NMR** (500 MHz, $CDCl_3$) δ 7.39-7.32 (m, 2H), 7.08 (d, *J* = 8.6 Hz, 1H), 6.94-6.90 (m, 2H), 6.37 (dd, *J* = 8.7, 1.1 Hz, 1H), 6.10 (t, *J* = 7.5 Hz, 1H), 2.87 (s, 2H), 1.71 (s, 2H), 1.35 (s, 6H), 0.72 (s, 9H); **^{13}C NMR** (126 MHz, $CDCl_3$) δ 154.4 (p, *J* = 15.6 Hz), 150.6, 150.4, 147.8, 140.4, 127.3, 120.2, 118.5-118.2 (m), 110.3-110.1 (m), 57.2,

38.5, 32.50, 32.04, 31.91, 31.56; **^{19}F NMR** (282 MHz, $CDCl_3$) δ 84.73-82.31 (m, 1F), 61.62 (d, *J* = 149.0 Hz, 4F); **HRMS (ESI)**: calcd for $C_{20}H_{27}F_5NOS$ $[M + H]^+$: 424.1734, found: 424.1750; **IR (KBr):** 3040, 2957, 2905, 1632, 1534, 1359, 1250, 1180, 900, 886, 781 cm^{-1}.

4-(4-((5-(Pentafluoro-λ^6-sulfaneyl)-3*H*-azepin-2-yl)oxy)phenyl)butan-2-one (3haa)

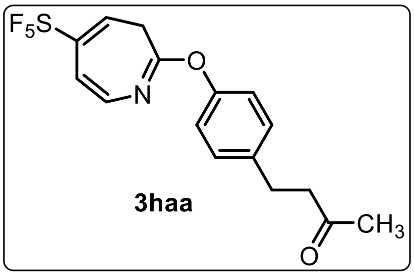

Following the general method **B** ((4-azidophenyl)pentafluoro-λ^6-sulfane **1h** (49.0 mg, 0.20 mmol), 4-(4-hydroxyphenyl)butan-2-one **2aa** (131.4 mg, 0.80 mmol), and DABCO (26.9 mg, 0.24 mmol) in 2 mL anhydrous DCE were used) compound **3haa** was obtained as a white solid, yield 58% (44.2 mg), mp: 67.1 – 69.2 °C; **^{1}H NMR** (500 MHz, $CDCl_3$) δ 7.21-7.15 (m, 2H), 7.06 (d, *J* = 8.7 Hz, 1H), 6.96-6.89 (m, 2H), 6.37 (dd, *J* = 8.7, 1.2 Hz, 1H), 6.12 (t, *J* = 7.5 Hz, 1H), 2.94-2.83 (m, 4H), 2.75 (t, *J* = 7.5 Hz, 2H), 2.14 (s, 3H); **^{13}C NMR** (126 MHz, $CDCl_3$) δ 154.4 (p, *J* = 15.6 Hz), 207.8, 151.0, 150.9, 140.2, 138.6, 129.5, 121.2, 118.6-118.3 (m), 110.5-110.2 (m), 45.1, 31.9, 30.2, 29.1; **^{19}F NMR** (282 MHz, cdcl3) δ 85.70-83.14 (m, 1F), 62.59 (d, *J* = 149.0 Hz, 4F); **HRMS (ESI)**: calcd for $C_{16}H_{16}F_5NO_2SNa$ $[M + Na]^+$: 404.0720, found: 404.0731; **IR (KBr)**: 3078, 2996, 1712, 1633, 1506, 1327, 1255, 1202, 1169, 901, 859, 818 cm^{-1}.

3-Methoxy-4-((5-(pentafluoro-λ^6-sulfaneyl)-3*H*-azepin-2-yl)oxy)benzaldehyde (3hab)

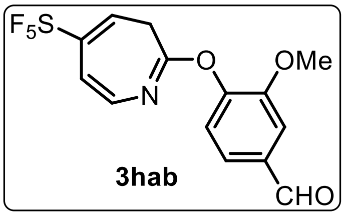

Following the general method **B** ((4-azidophenyl)pentafluoro-λ^6-sulfane **1h** (49.0 mg, 0.20 mmol), 4-hydroxy-3-methoxybenzaldehyde **2ab** (121.7 mg, 0.80 mmol), and DABCO (26.9 mg, 0.24 mmol) in 2 mL anhydrous DCE were used) compound **3hab** was obtained as a white solid, yield 48% (35.4 mg), mp: 133.6 – 135.7 °C; **^{1}H NMR** (500 MHz, $CDCl_3$) δ 9.93 (s, 1H), 7.49 (dd, *J* = 8.0, 1.8 Hz, 1H), 7.45 (d, *J* = 1.7 Hz, 1H), 7.24 (d, *J* = 8.0 Hz, 1H), 7.00 (d, *J* = 8.6 Hz, 1H), 6.39 (dd, *J* = 8.7, 1.2 Hz, 1H), 6.15 (t, *J* = 7.5 Hz, 1H), 3.74 (s, 3H), 2.94 (s, 2H);

^{13}**C NMR** (126 MHz, $CDCl_3$) δ 191.1, 154.0 (p, J = 15.6 Hz), 151.6, 149.1, 146.7, 139.6, 135.4, 124.9, 122.8, 119.0-118.7 (m), 111.3, 110.6-110.4 (m), 55.7, 31.5; ^{19}**F NMR** (282 MHz, $CDCl_3$) δ 84.77-82.55 (m, 1F), 61.61 (d, J = 149.0 Hz, 4F); **HRMS (ESI)**: calcd for $C_{14}H_{13}F_5NO_3S$ $[M + H]^+$: 370.0536, found: 370.0541; **IR (KBr)**: 3038, 2985, 2949, 2923, 1698, 1636, 1501, 1424, 1325, 1200, 890, 830, 792 cm^{-1}.

1-(3-Methoxy-4-((5-(pentafluoro-λ^6-sulfaneyl)-3*H*-azepin-2-yl)oxy)phenyl)ethan-1-one (3hac)

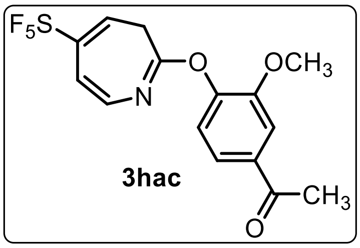

Following the general method **B** ((4-azidophenyl)pentafluoro-λ^6-sulfane **1h** (49.0 mg, 0.20 mmol), 1-(4-hydroxy-3-methoxyphenyl)ethan-1-one **2ac** (132.9 mg, 0.80 mmol), and DABCO (26.9 mg, 0.24 mmol) in 2 mL anhydrous DCE were used) compound **3hac** was obtained as a white solid, yield 46% (35.2 mg), mp: 89.5 – 91.1 °C; ^{1}**H NMR** (500 MHz, $CDCl_3$) δ 7.58-7.54 (m, 2H), 7.15 (d, J = 8.1 Hz, 1H), 7.00 (d, J = 8.6 Hz, 1H), 6.37 (dd, J = 8.7, 1.0 Hz, 1H), 6.14 (t, J = 7.5 Hz, 1H), 3.72 (s, 3H), 2.93 (s, 1H), 2.58 (s, 3H); ^{13}**C NMR** (126 MHz, $CDCl_3$) δ 196.9, 154.0 (p, J = 15.3 Hz), 150.9, 149.3, 145.6, 139.7, 136.1, 122.2, 122.2, 118.9-118.7 (m), 111.9, 110.5-110.2 (m), 55.7, 31.5, 26.6; ^{19}**F NMR** (282 MHz, $CDCl_3$) δ 84.86-82.58 (m, 1F), 61.62 (d, J = 148.9 Hz, 4F); **HRMS (ESI)**: calcd for $C_{15}H_{14}F_5NO_3SNa$ $[M + Na]^+$: 406.0512, found: 406.0514; **IR (KBr)**: 3080, 2968, 2921, 2347, 1740, 1682, 1644, 1501, 1325, 1280, 1176, 887, 835, 809 cm^{-1}.

2-(5-Chloro-2-(2,5-dichlorophenoxy)phenoxy)-5-(pentafluoro-λ^6-sulfaneyl)-3*H*-azepine (3had)

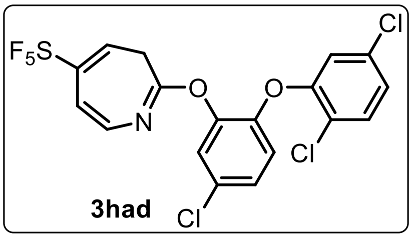

Following the general method **B** ((4-azidophenyl)pentafluoro-λ^6-sulfane **1h** (49.0 mg, 0.20 mmol), 5-chloro-2-(2,5-dichlorophenoxy)phenol **2ad** (231.6 mg, 0.80 mmol), and DABCO

(26.9 mg, 0.24 mmol) in 2 mL anhydrous DCE were used) compound **3had** was obtained as a white solid, yield 70% (70.9 mg), mp: 126.6 – 128.8 °C; **^{1}H NMR** (500 MHz, $CDCl_3$) δ 7.41 (d, J = 2.5 Hz, 1H), 7.22 (d, J = 2.5 Hz, 1H), 7.15-7.10 (m, 2H), 6.99 (d, J = 8.7 Hz, 1H), 6.75 (d, J = 8.8 Hz, 1H), 6.67 (d, J = 8.8 Hz, 1H), 6.34 (dd, J = 8.7, 1.2 Hz, 1H), 6.03 (t, J = 7.4 Hz, 1H), 2.88 (s, 2H); **^{13}C NMR** (126 MHz, $CDCl_3$) δ 154.0 (p, J = 15.6 Hz), 150.3, 149.8, 147.1 143.2, 139.4, 130.5, 130.1, 128.9, 128.2, 127.1, 126.6, 123.8, 121.4, 119.3, 119.0-118.7 (m), 111.1, 31.2; **^{19}F NMR** (282 MHz, $CDCl_3$) δ 84.26-82.00 (m, 1F), 61.43 (d, J = 149.2 Hz, 4F); **HRMS (ESI)**: calcd for $C_{18}H_{12}F_5Cl_3NO_2S$ [M + H]$^+$: 505.9574, found: 505.9576; **IR (KBr)**: 3078, 2996, 1640, 1582, 1475, 1334, 1286, 1181, 877, 841, 790 cm^{-1}.

2-(5-isopropyl-2-methylphenoxy)-5-(pentafluoro-λ^6--sulfaneyl)-3*H*-azepine (3hae)

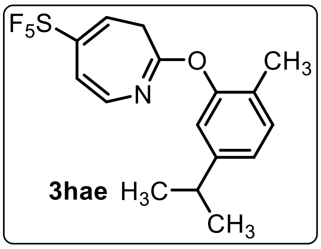

Following the general method **B** ((4-azidophenyl)pentafluoro-λ^6-sulfane **1h** (49.0 mg, 0.20 mmol), 5-isopropyl-2-methylphenol **2ae** (120.2 mg, 0.80 mmol), and DABCO (26.9 mg, 0.24 mmol) in 2 mL anhydrous DCE were used) compound **3hae** was obtained as a white solid, yield 75% (55.1 mg), mp: 94.1 – 96.2 °C; **^{1}H NMR** (500 MHz, $CDCl_3$) δ 7.11 (d, J = 7.8 Hz, 1H), 7.06 (d, J = 8.6 Hz, 1H), 7.01 (dd, J = 7.8, 1.8 Hz, 1H), 6.82 (d, J = 1.7 Hz, 1H), 6.34 (dd, J = 8.6, 1.2 Hz, 1H), 6.13 (t, J = 7.5 Hz, 1H), 2.97 – 2.83 (m, 3H), 1.97 (s, 3H), 1.22 (d, J = 6.9 Hz, 6H); **^{13}C NMR** (126 MHz, $CDCl_3$) δ 154.8-154.1 (m), 151.1, 150.2, 148.3, 140.5, 1313, 126.9, 124.5, 119.5, 118.7-118.4 (m), 109.9-109.7 (m), 33.6, 31.9, 23.9, 15.9; **^{19}F NMR** (282 MHz, $CDCl_3$) δ 84.83-82.52 (m, 1F), 61.68 (d, J = 148.8 Hz, 4F); **HRMS (ESI)**: calcd for $C_{16}H_{19}F_5NOS$ [M + H]$^+$: 368.1108, found: 368.1119; **IR (KBr)**: 3052, 2964, 1629, 1528, 1415, 1331, 1267, 1176, 1003, 891, 834, 783 cm^{-1}.

***N*-(4-((5-(Pentafluoro-λ^6-sulfaneyl)-3*H*-azepin-2-yl)oxy)phenyl)acetamide (3haf)**

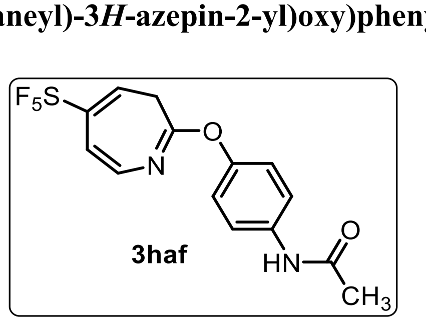

Following the general method **B** ((4-azidophenyl)pentafluoro-λ^6-sulfane **1h** (49.0 mg, 0.20 mmol), *N*-(4-hydroxyphenyl)acetamide **2af** (120.9 mg, 0.80 mmol), and DABCO (26.9 mg, 0.24 mmol) in 2 mL anhydrous DCE were used) compound **3haf** was obtained as a pale yellow solid, yield 60% (44.1 mg), mp: 159.7 – 162.4 °C; **^{1}H NMR** (500 MHz, DMSO-d$_6$) δ 10.00 (s, 1H), 7.57 (d, *J* = 8.9 Hz, 2H), 7.09 (d, *J* = 8.6 Hz, 1H), 6.96 (d, *J* = 8.9 Hz, 2H), 6.57-6.24 (m, 2H), 2.95 (s, 2H), 2.03 (s, 3H); **^{13}C NMR** (126 MHz, DMSO-d$_6$) δ 168.2, 153.2-152.6 (m), 151.2, 147.6, 140.4, 136.9, 121.4, 120.8, 119.9, 109.2, 31.3, 23.9; **^{19}F NMR** (282 MHz, DMSO-d$_6$) δ 86.84-84.57 (m, 1F), 61.61 (d, *J* = 149.7 Hz, 4F); **HRMS (ESI)**: calcd for $C_{14}H_{14}F_5N_2O_2S$ [M + H]$^+$: 369.0696, found: 369.0706; **IR (KBr)**: 3303, 3274, 3206, 2927, 1667, 1634, 1504, 1332, 1250, 1196, 874, 849, 833, 810 cm^{-1}.

(8R,9S,13S,14S)-13-Methyl-3-((5-(pentafluoro-λ^6-sulfaneyl)-3*H*-azepin-2-yl)oxy)-6,7,8,9,11,12,13,14,15,16-decahydro-17*H*-cyclopenta[a]phenanthren-17-one (3hag)

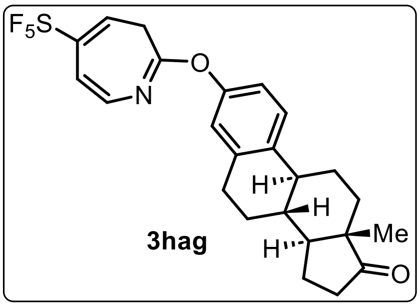

Following the general method **B** ((4-azidophenyl)pentafluoro-λ^6-sulfane **1h** (49.0 mg, 0.20 mmol), estrone **2ag** (216.3 mg, 0.80 mmol), and DABCO (26.9 mg, 0.24 mmol) in 2 mL anhydrous DCE were used) compound **3hag** was obtained as a white solid, yield 33% (32.1 mg), mp: 95.6 – 97.8 °C; **^{1}H NMR** (500 MHz, CDCl$_3$) δ 7.28 (d, *J* = 8.5 Hz, 1H), 7.08 (d, *J* = 8.6 Hz, 1H), 6.81-6.73 (m, 2H), 6.37 (dd, *J* = 8.7, 1.2 Hz, 1H), 6.12 (t, *J* = 7.4 Hz, 1H), 2.95-2.82 (m, 4H), 2.50 (dd, *J* = 19.1, 8.6 Hz, 1H), 2.43-2.35 (m, 1H), 2.27 (td, *J* = 10.8, 3.8 Hz, 1H), 2.19-2.10 (m, 1H), 2.09-1.92 (m, 3H), 1.68-1.54 (m, 2H), 1.54-1.39 (m, 4H), 0.90 (s, 3H); **^{13}C NMR** (126 MHz, CDCl$_3$) δ 154.4 (p, *J* = 15.4 Hz), 151.1, 150.6, 140.4, 138.3, 137.5, 126.6, 121.1, 118.5, 118.4, 110.3, 50.5, 48.0, 44.3, 38.0, 35.9, 31.9, 31.6, 29.5, 26.4, 25.8, 21.7, 13.9; **^{19}F NMR** (282 MHz, CDCl$_3$) δ 84.68-82.31 (m, 1F), 61.58 (d, *J* = 149.1 Hz, 4F); **HRMS (ESI)**: calcd for $C_{24}H_{27}F_5NO_2S$ [M + H]$^+$: 488.1683, found: 488.1697; **IR (KBr)**: 3074, 2933, 2890, 2373, 1736, 1632, 1493, 1328, 1248, 1132, 888, 816 cm^{-1}.

(E)-1,2-bis(4-methoxyphenyl)diazene (6)

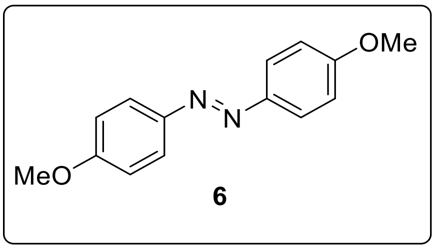

Following the general method **B** (1-azido-4-methoxybenzene **1g** (29.8 mg, 0.20 mmol), phenol **2a** (75.3 mg, 0.80 mmol) and DABCO (26.9 mg, 0.24 mmol) in 2 mL anhydrous DCE were used) compound **6** was obtained as an orange solid, yield 48% (23.3 mg); **^{1}H NMR** (500 MHz, $CDCl_3$) δ 7.88 (d, *J* = 9.0 Hz, 4H), 7.00 (d, *J* = 9.0 Hz, 4H), 3.88 (s, 6H); **^{13}C NMR** (126 MHz, $CDCl_3$) δ 161.68, 147.19, 124.48, 114.29, 55.69. The data of compound **6** were in accordance with previous literature.[5]

4. General procedure for the synthesis of 5-(pentafluoro-λ^6-sulfaneyl)-1,3-dihydro-2*H*-azepin-2-one (4h)

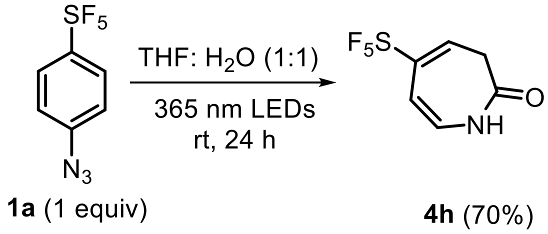

In an oven-dried screw-cap vial equipped with a magnetic stir bar, (4-azidophenyl)pentafluoro-λ^6-sulfane **1h** (49.0 mg, 0.20 mmol) was dissolved in 2 mL of THF: H_2O (1:1). The reaction mixture was stirred and irradiated with blue LED light (Kessil A160WE Tuna Blue, LED lighting (40 W)) for 24 hours. After that, the organic layer was extracted with ethyl acetate, dried over anhydrous Na_2SO_4, and concentrated under reduced pressure. The crude product was purified by column chromatography using *n*-hexane/ethyl acetate to afford Pure compound **4h** as a white solid, yield 70% (32.9 mg), mp: 93.3 – 95.4 °C; **^{1}H NMR** (500 MHz, $CDCl_3$) δ 8.62 (s, 1H), 6.42 (dd, *J* = 9.2, 5.0 Hz, 1H), 6.33 (t, *J* = 7.6 Hz, 1H), 6.17 (d, *J* = 9.2 Hz, 1H), 2.99 (d, *J* = 7.5 Hz, 2H); **^{13}C NMR** (126 MHz, $CDCl_3$) δ 168.7, 154.08 (p, *J* = 16.4 Hz), 128.7, 123.5-123.3 (m), 109.5-109.3 (m), 35.7; **^{19}F NMR** (376 MHz, $CDCl_3$) δ 83.53-81.89 (m, 1F), 60.62 (d, *J* = 149.2 Hz, 4F); **HRMS (ESI)**: calcd for $C_6H_7F_5NOS$ $[M + H]^+$: 236.0169, found: 236.0169; **IR (KBr)**: 3208, 3078, 2952, 1701, 1638, 1601, 1324, 876, 810, 766 cm^{-1}.

5. General procedure for synthesis of substituted 2,2,2-trifluoro-*N*-(2-phenoxyphenyl)acetamide (5ha–5he, 5aa–5fa) [Method-1C]

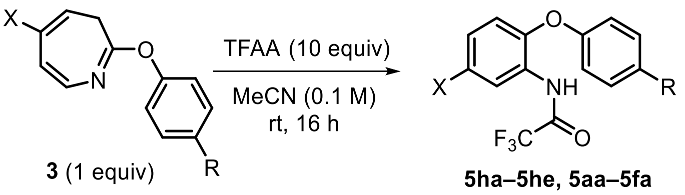

3 (1 equiv) **5ha–5he, 5aa–5fa**

In an oven-dried screw-cap vial equipped with a magnetic stir bar, substituted 2-phenoxy-3*H*-azepine (**3**) (0.10 mmol, 1 equiv), trifluoroacetic anhydride (1 mmol, 10 equiv) were dissolved in acetonitrile (0.1 M). The reaction mixture was stirred at rt for 16 hours. Afterward, the solvent was removed under reduced pressure, and the residue was purified by column chromatography using *n*-hexane/ethyl acetate to afford pure compound (**5**).

2,2,2-Trifluoro-*N*-(5-(pentafluoro-λ^6-sulfaneyl)-2-phenoxyphenyl)acetamide (5ha)

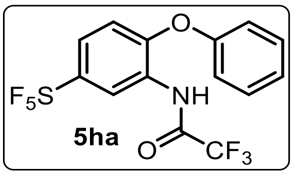

Following the general method **1C** (5-(pentafluoro-λ^6-sulfaneyl)-2-phenoxy-3*H*-azepine **3ha** (31.1 mg, 0.10 mmol), TFAA (0.14 mL, 1.0 mmol) in 1 mL anhydrous acetonitrile were used) compound **5ha** was obtained as a white solid, yield 92% (37.4 mg), mp: 100.3 – 102.3 °C; **^{1}H NMR** (500 MHz, $CDCl_3$) δ 8.92 (d, *J* = 2.6 Hz, 1H), 8.65 (s, 1H), 7.51-7.45 (m, 3H), 7.34-7.30 (m, 1H), 7.14-7.10 (m, 2H), 6.80 (d, *J* = 9.1 Hz, 1H); **^{13}C NMR** (126 MHz, $CDCl_3$) δ 154.9 (q, *J* = 38.2 Hz), 153.9, 149.3, 148.2 (p, *J* = 18.9 Hz), 130.7, 126.3, 125.7, 124.2-123.9 (m), 120.6, 119.2, 115.5 (q, *J* = 288.5 Hz), 114.7; **^{19}F NMR** (376 MHz, $CDCl_3$) δ 84.37-82.66 (m, 1F), 63.24 (d, *J* = 150.5 Hz, 4F), -76.21 (s, 3F); **HRMS (ESI)**: calcd for $C_{14}H_9F_8NO_2SNa$ [M + Na]$^+$: 430.0124, found: 430.0136; **IR (KBr)**: 3303, 1668, 1633, 1548, 1504, 1332, 1318, 1250, 1196, 873, 849, 833, 781 cm^{-1}.

2,2,2-Trifluoro-*N*-(5-(pentafluoro-λ^6-sulfaneyl)-2-(*p*-tolyloxy)phenyl)acetamide (5hb)

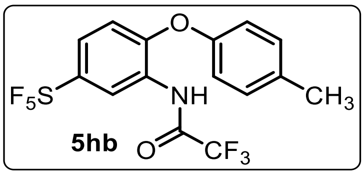

Following the general method **1C** (5-(pentafluoro-λ^6-sulfaneyl)-2-(*p*-tolyloxy)-3*H*-azepine **3hb** (32.5 mg, 0.10 mmol), TFAA (0.14 mL, 1.0 mmol) in 1 mL anhydrous acetonitrile were used) compound **5hb** was obtained as a white solid, yield 94% (39.6 mg), mp: 69.3 – 71.3 °C;

^{1}**H NMR** (500 MHz, $CDCl_3$) δ 8.90 (d, *J* = 2.6 Hz, 1H), 8.68 (s, 1H), 7.47 (dd, *J* = 9.2, 2.7 Hz, 1H), 7.28-7.24 (m, 2H), 7.02-6.98 (m, 2H), 6.77 (d, *J* = 9.1 Hz, 1H), 2.40 (s, 3H); ^{13}**C NMR** (126 MHz, $CDCl_3$) δ 154.9 (q, *J* = 38.1 Hz), 151.6, 149.7, 147.9 (p, *J* = 18.7 Hz), 136.2, 131.1, 125.5, 124.2-123.9 (m), 120.5, 119.2-118.9 (m, *J* = 9.0, 4.6 Hz), 115.5 (q, *J* = 288.5 Hz), 114.3, 20.9; ^{19}**F NMR** (376 MHz, $CDCl_3$) δ 84.53-82.82 (m, 1F), 63.30 (d, *J* = 150.5 Hz, 4F), -76.20 (s, 3F); **HRMS (ESI)**: calcd for $C_{15}H_{11}F_8NO_2SNa$ $[M + Na]^+$: 444.0280, found: 440.0298; **IR (KBr)**: 3378, 1733, 1608, 1548, 1482, 1269, 1229, 1166, 860, 842, 809 cm^{-1}.

2,2,2-Trifluoro-*N*-(2-(4-methoxyphenoxy)-5-(pentafluoro-λ^6-sulfaneyl)phenyl)acetamide (5hc)

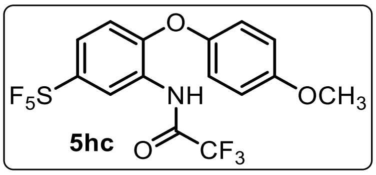

Following the general method **1C** (2-(4-methoxyphenoxy)-5-(pentafluoro-λ^6-sulfaneyl)-3*H*-azepine **3hc** (34.1 mg, 0.10 mmol), TFAA (0.14 mL, 1.0 mmol) in 1 mL anhydrous acetonitrile were used) compound **5hc** was obtained as a white solid, yield 93% (40.6 mg), mp: 94.9 – 96.8 °C; ^{1}**H NMR** (500 MHz, $CDCl_3$) δ 8.89 (d, *J* = 2.6 Hz, 1H), 8.69 (s, 1H), 7.46 (dd, *J* = 9.2, 2.6 Hz, 1H), 7.07-7.03 (m, 2H), 7.00-6.95 (m, 2H), 6.73 (d, *J* = 9.1 Hz, 1H), 3.85 (s, 3H); ^{13}**C NMR** (126 MHz, $CDCl_3$) δ 157.8, 154.9 (q, *J* = 38.2 Hz), 150.1, 147.8 (p, *J* = 18.7 Hz), 146.9, 125.3, 124.2-123.9 (m), 121.9, 119.0, 115.6, 115.5 (q, *J* = 288.4 Hz), 113.8, 55.8; ^{19}**F NMR** (376 MHz, $CDCl_3$) δ 84.67-82.80 (m, 1F), 63.33 (d, *J* = 150.5 Hz, 4F), -76.18 (s, 3F); **HRMS (ESI)**: calcd for $C_{15}H_{11}F_8NO_3SNa$ $[M + Na]^+$: 460.0230, found: 460.0238; **IR (KBr)**: 3396, 1734, 1609, 1551, 1509, 1481, 1226, 1186, 908, 843, 813 cm^{-1}.

2,2,2-Trifluoro-*N*-(2-(4-fluorophenoxy)-5-(pentafluoro-λ^6-sulfaneyl)phenyl)acetamide (5hd)

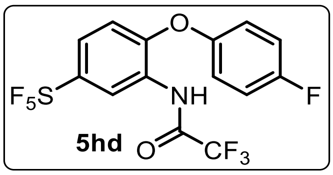

Following the general method **1C** (2-(4-fluorophenoxy)-5-(pentafluoro-λ^6-sulfaneyl)-3*H*-azepine **3hg** (32.9 mg, 0.10 mmol), TFAA (0.14 mL, 1.0 mmol) in 1 mL anhydrous acetonitrile were used) compound **5hd** was obtained as a white solid, yield 86% (36.5 mg), mp: 102.5 – 104.4 °C; ^{1}**H NMR** (500 MHz, $CDCl_3$) δ 8.90 (d, J = 2.6 Hz, 1H), 8.62 (s, 1H), 7.50 (dd, J =

9.1, 2.6 Hz, 1H), 7.19-7.14 (m, 2H), 7.13-7.08 (m, 2H), 6.75 (d, J = 9.1 Hz, 1H); **^{13}C NMR** (126 MHz, $CDCl_3$) δ 160.5 (d, *J* = 245.9 Hz), 154.9 (q, *J* = 38.2 Hz), 149.6 (d, *J* = 2.6 Hz), 149.5, 148.2 (p, *J* = 18.9 Hz), 125.6, 124.3-124.0 (m), 122.3 (d, *J* = 8.5 Hz), 119.4-119.2 (m), 117.5 (d, *J* = 23.7 Hz), 115.5 (q, *J* = 288.5 Hz), 114.2; **^{19}F NMR** (376 MHz, $CDCl_3$) δ 84.22-82.52 (m, 1F), 63.22 (d, *J* = 150.6 Hz, 4F), -115.98 to -116.10 (m, 1F), -76.18 (s, 3F); **HRMS (ESI)**: calcd for $C_{14}H_8F_9NO_2SNa$ [M + Na]$^+$: 448.0030, found: 448.0034; I**R (KBr)**: 3396, 1734, 1549, 1504, 1482, 1219, 1186, 1156, 909, 857, 812 cm^{-1}.

***N*-(2-([1,1'-Biphenyl]-4-yloxy)-5-(pentafluoro-λ^6-sulfaneyl)phenyl)-2,2,2-Trifluoroacetamide (5he)**

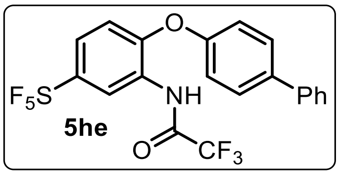

Following the general method **1C** (2-([1,1'-biphenyl]-4-yloxy)-5-(pentafluoro-λ^6-sulfaneyl)-3*H*-azepine **3ho** (38.7 mg, 0.10 mmol), TFAA (0.14 mL, 1.0 mmol) in 1 mL anhydrous acetonitrile were used) compound **5he** was obtained as a white solid, yield 92% (44.4 mg), mp: 128.6 – 130.1 °C; **^{1}H NMR** (500 MHz, $CDCl_3$) δ 8.93 (d, *J* = 2.6 Hz, 1H), 8.66 (s, 1H), 7.69-7.66 (m, 2H), 7.61-7.58 (m, 2H), 7.52 (dd, *J* = 9.1, 2.7 Hz, 1H), 7.50-7.46 (m, 2H), 7.41-7.37 (m, 1H), 7.20-7.17 (m, 2H), 6.89 (d, *J* = 9.1 Hz, 1H); **^{13}C NMR** (126 MHz, $CDCl_3$) δ 154.9 (q, *J* = 38.2 Hz), 153.3, 149.2, 148.6-147.9 (m), 139.9, 139.5, 129.3, 129.1, 127.8, 127.2, 125.8, 124.3-124.0 (m), 120.8, 119.3, 115.5 (q, *J* = 288.5 Hz), 114.8; **^{19}F NMR** (376 MHz, $CDCl_3$) δ 84.3-82.6 (m, 1F), 63.22 (d, *J* = 150.5 Hz, 4F), -76.20 (s, 3F); **HRMS (ESI)**: calcd for $C_{20}H_{13}F_8NO_2SNa$ [M + Na]$^+$: 506.0437, found: 506.0444; **IR (KBr)**: 3401, 1733, 1546, 1514, 1478, 1221, 1177, 906, 853, 812 cm^{-1}.

2,2,2-Trifluoro-*N*-(2-phenoxyphenyl)acetamide (5aa)

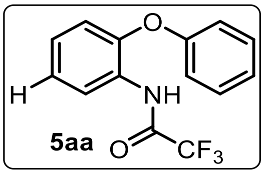

Following the general method **1C** (2-phenoxy-3*H*-azepine **3aa** (18.5 mg, 0.10 mmol), TFAA (0.14 mL, 1.0 mmol) in 1 mL anhydrous acetonitrile were used) compound **5aa** was obtained as a colourless oil, yield 90% (25.3 mg), **^{1}H NMR** (500 MHz, $CDCl_3$) δ 8.50 (s, 1H), 8.30 (dd, *J* = 7.7, 2.0 Hz, 1H), 7.34-7.29 (m, 2H), 7.15-7.10 (m, 1H), 7.08-7.01 (m, 2H), 7.00-6.97 (m,

2H), 6.79-6.76 (m, 1H); **13C NMR** (126 MHz, $CDCl_3$) δ 155.6, 154.7 (q, *J* = 37.5 Hz), 146.9, 130.3, 126.7, 126.3, 124.9, 123.9, 121.1, 119.5, 117.1, 115.7 (q, *J* = 288.6 Hz); **19F NMR** (376 MHz, $CDCl_3$) δ -76.05 (s, 3F); **HRMS (ESI)**: calcd for $C_{14}H_{11}F_3NO_2$ $[M + H]^+$: 282.0742, found: 282.0746; **IR (KBr)**: 3403, 1737, 1541, 1484, 1220, 1155, 904, 876, 824 cm^{-1}.

2,2,2-Trifluoro-*N*-(5-methyl-2-phenoxyphenyl)acetamide (5ba)

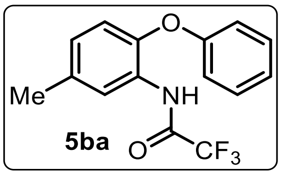

Following the general method **1C** (5-methyl-2-phenoxy-3*H*-azepine **3ba** (19.9 mg, 0.10 mmol), TFAA (0.14 mL, 1.0 mmol) in 1 mL anhydrous acetonitrile were used) compound **5ba** was obtained as a white solid, yield 88% (25.9 mg), mp: 41.2 – 43.5 °C; **1H NMR** (500 MHz, $CDCl_3$) δ 8.49 (s, 1H), 8.21 (d, *J* = 1.8 Hz, 1H), 7.40-7.35 (m, 2H), 7.19-7.15 (m, 1H), 7.04-7.01 (m, 2H), 6.93 (ddd, *J* = 8.3, 2.0, 0.6 Hz, 1H), 6.78 (d, *J* = 8.3 Hz, 1H), 2.36 (s, 3H); **13C NMR** (126 MHz, $CDCl_3$) δ 156.1, 154.6 (q, *J* = 37.4 Hz), 144.4, 134.1, 130.2, 126.8, 126.7, 124.5, 121.6, 118.9, 117.6, 115.7 (q, *J* = 288.6 Hz), 21.2; **19F NMR** (376 MHz, $CDCl_3$) δ -76.30 (s, 3F); **HRMS (ESI)**: calcd for $C_{15}H_{13}F_3NO_2$ $[M + H]^+$: 296.0898, found: 296.0900; **IR (KBr)**: 3413, 1735, 1546, 1483, 1221, 1161, 904, 853, 817 cm^{-1}.

2,2,2-Trifluoro-*N*-(5-fluoro-2-phenoxyphenyl)acetamide (5ca)

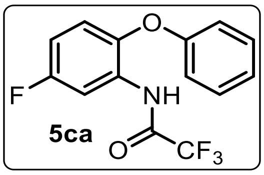

Following the general method **1C** (5-fluoro-2-phenoxy-3*H*-azepine **3ca** (20.3 mg, 0.10 mmol), TFAA (0.14 mL, 1.0 mmol) in 1 mL anhydrous acetonitrile were used) compound **5ca** was obtained as a yellow oil, yield 87% (26.0 mg), **1H NMR** (500 MHz, $CDCl_3$) δ 8.57 (s, 1H), 8.23-8.20 (m, 1H), 7.42-7.37 (m, 2H), 7.23-7.18 (m, 1H), 7.03 (ddd, *J* = 4.6, 3.4, 1.8 Hz, 2H), 6.86-6.83 (m, 2H); **13C NMR** (126 MHz, $CDCl_3$) δ 158.4 (d, *J* = 242.4 Hz), 155.8, 154.8 (q, *J* = 37.9 Hz), 142.6 (d, *J* = 2.8 Hz), 130.4, 127.7 (d, *J* = 11.7 Hz), 124.9, 118.9, 118.4 (d, *J* = 9.2 Hz), 115.5 (q, *J* = 288.5 Hz), 112.5 (d, *J* = 23.6 Hz), 108.7 (d, *J* = 29.8 Hz); **19F NMR** (376 MHz, $CDCl_3$) δ -76.30 (s, 3F), -116.43 (s, 1F); **HRMS (ESI)**: calcd for $C_{14}H_{10}F_4NO_2$ $[M + H]^+$: 300.0648, found: 300.0636; **IR (KBr)**: 3411, 1738, 1547, 1479, 1218, 1166, 1137, 906, 857, 814 cm^{-1}.

N-(5-Bromo-2-phenoxyphenyl)-2,2,2-trifluoroacetamide (5da)

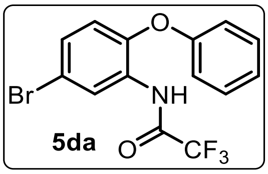

Following the general method **1C** (5-bromo-2-phenoxy-3*H*-azepine **3da** (26.4 mg, 0.10 mmol), TFAA (0.14 mL, 1.0 mmol) in 1 mL anhydrous acetonitrile were used) compound **5da** was obtained as a yellow oil, yield 94% (33.8 mg), **^{1}H NMR** (500 MHz, $CDCl_3$) δ 8.58 (d, J = 2.4 Hz, 1H), 8.56 (s, 1H), 7.44-7.39 (m, 2H), 7.25-7.20 (m, 2H), 7.08-7.05 (m, 2H), 6.71 (d, J = 8.8 Hz, 1H); **^{13}C NMR** (126 MHz, $CDCl_3$) δ 155.0, 154.7 (q, J = 37.9 Hz),146.1, 130.4, 129.0, 127.7, 125.3, 123.8, 119.6, 118.0, 116.0, 115.5 (q, J = 288.6 Hz); **^{19}F NMR** (376 MHz, $CDCl_3$) δ -76.21 (s, 3F); **HRMS (ESI)**: calcd for $C_{14}H_{10}F_3BrNO_2$ [M + H]$^+$: 359.9847, found: 359.9848; **IR (KBr)**: 3408, 1734, 1536, 1474, 1256, 1218, 1149, 908, 877, 811 cm^{-1}.

2,2,2-Trifluoro-N-(2-phenoxy-5-(trifluoromethyl)phenyl)acetamide (5ea)

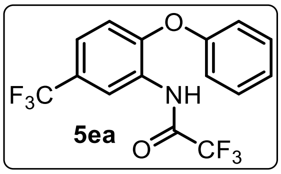

Following the general method **1C** (2-phenoxy-5-(trifluoromethyl)-3*H*-azepine **3ea** (25.3 mg, 0.10 mmol), TFAA (0.14 mL, 1.0 mmol) in 1 mL anhydrous acetonitrile were used) compound **5ea** was obtained as a white solid, yield 94% (32.8 mg), mp: 37.5 – 39.0 °C; **^{1}H NMR** (500 MHz, $CDCl_3$) δ 8.73 (d, J = 2.0 Hz, 1H), 8.67 (s, 1H), 7.49-7.44 (m, 2H), 7.38-7.34 (m, 1H), 7.32-7.28 (m, 1H), 7.13-7.10 (m, 2H), 6.86 (d, J = 8.6 Hz, 1H); **^{13}C NMR** (126 MHz, $CDCl_3$) δ 154.9 (q, J = 38.0 Hz), 154.3, 149.7, 130.6, 126.5, 126.0, 125.6 (q, J = 33.3 Hz), 123.8 (q, J = 272.0 Hz), 123.3 (dd, J = 7.4, 3.6 Hz), 120.4, 118.3 (d, J = 3.3 Hz), 115.7, 115.6 (q, J = 288.5 Hz); **^{19}F NMR** (376 MHz, $CDCl_3$) δ -62.51 (s, 3F), -76.21 (s, 3F); **HRMS (ESI)**: calcd for $C_{15}H_9F_6NO_2Na$ [M + Na]$^+$: 372.0435, found: 372.0443; **IR (KBr)**: 3399, 1730, 1554, 1486, 1330, 1216, 1178, 1117, 898, 853, 728 cm^{-1}.

Ethyl 4-phenoxy-3-(2,2,2-trifluoroacetamido)benzoate (5fa)

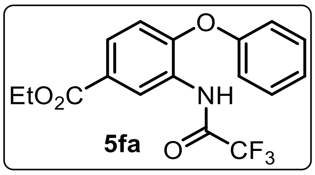

Following the general method **1C** (ethyl 2-phenoxy-3*H*-azepine-5-carboxylate **3fa** (25.7 mg, 0.10 mmol), TFAA (0.14 mL, 1.0 mmol) in 1 mL anhydrous acetonitrile were used) compound **5fa** was obtained as a white solid, yield 93% (32.8 mg), mp: 52.9 – 54.6 °C; **^{1}H NMR** (500 MHz, $CDCl_3$) δ 9.03 (d, *J* = 2.0 Hz, 1H), 8.61 (s, 1H), 7.82 (dd, *J* = 8.7, 2.1 Hz, 1H), 7.48-7.43 (m, 2H), 7.31-7.27 (m, 1H), 7.13-7.09 (m, 2H), 6.81 (d, *J* = 8.7 Hz, 1H), 4.38 (q, *J* = 7.1 Hz, 2H), 1.40 (t, *J* = 7.1 Hz, 3H); **^{13}C NMR** (126 MHz, $CDCl_3$) δ 165.6, 154.8 (q, *J* = 37.8 Hz), 154.3, 150.9, 130.5, 128.2, 125.8, 125.8, 125.7, 122.4, 120.5, 115.7 (q, *J* = 288.6 Hz), 115.2, 61.4, 14.4; **^{19}F NMR** (376 MHz, $CDCl_3$) δ -76.20 (s, 3F); **HRMS (ESI)**: calcd for $C_{17}H_{15}F_3NO_4$ $[M + H]^+$: 354.0953, found: 354.0960; **IR (KBr)**: 3407, 1735, 1713, 1551, 1484, 1302, 1214, 1131, 905, 865, 767 cm^{-1}.

General procedure for synthesis of substituted 2,2-difluoro-*N*-(5-(pentafluoro-λ^6-sulfaneyl)-2-phenoxyphenyl)acetamide (5haa–5had) [Method-2C]

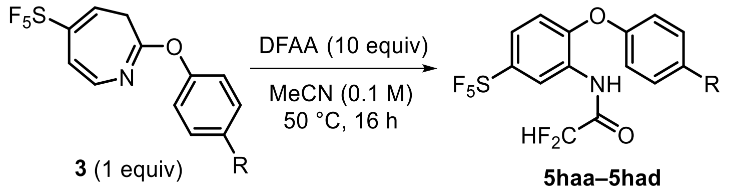

In an oven-dried screw-cap vial equipped with a magnetic stir bar, substituted 2-phenoxy-3*H*-azepine (**3**) (0.10 mmol, 1 equiv), difluoroacetic anhydride (1 mmol, 10 equiv) were dissolved in acetonitrile (0.1 M). The reaction mixture was stirred at 50 °C for 16 hours. Afterward, the solvent was removed under reduced pressure, and the residue was purified by column chromatography using *n*-hexane/ethyl acetate/acetone/Et_3N to afford pure compound (**5haa**–**5had**).

2,2-difluoro-*N*-(5-(pentafluoro-λ^6-sulfaneyl)-2-phenoxyphenyl)acetamide (5haa)

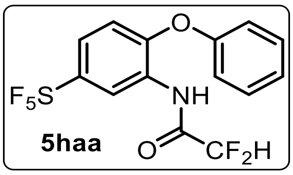

Following the general method **2C** (5-(pentafluoro-λ^6-sulfaneyl)-2-phenoxy-3*H*-azepine **3ha** (31.1 mg, 0.10 mmol), difluoroacetic anhydride (0.11 mL, 1.0 mmol) in 1 mL anhydrous acetonitrile were used) compound **5haa** was obtained as a yellow oil, yield 89% (34.8 mg), **^{1}H NMR** (500 MHz, $CDCl_3$) δ 8.97 (d, *J* = 2.7 Hz, 1H), 8.69 (s, 1H), 7.51 – 7.43 (m, 3H), 7.32 – 7.27 (m, 1H), 7.11 (dd, *J* = 8.7, 1.2 Hz, 2H), 6.79 (d, *J* = 9.1 Hz, 1H), 6.06 (t, *J* = 54.2 Hz, 1H);

^{13}C NMR (126 MHz, $CDCl_3$) δ 160.6 (t, J = 25.1 Hz), 154.2, 149.1, 148.3 (p, J = 18.0 Hz), 130.7, 126.5, 126.1, 123.5, 120.5, 119.1, 114.9, 108.4; (t, J = 254.5 Hz); **^{19}F NMR** (658 MHz, $CDCl_3$) δ 83.77 (p, J = 150.7 Hz, 1F), 63.23 (d, J = 150.5 Hz, 4F), -126.19 (dd, J = 54.2, 2.5 Hz, 2F); **HRMS (ESI)**: calcd for $C_{14}H_{11}F_7NO_2S$ $[M + H]^+$: 390.0399, found: 390.0392; **IR (KBr)**: 3307, 1718, 1608, 1543, 1480, 1219, 1091, 929, 844 cm^{-1}.

2,2-difluoro-*N*-(5-(pentafluoro-λ^6-sulfaneyl)-2-(*p*-tolyloxy)phenyl)acetamide (5hab)

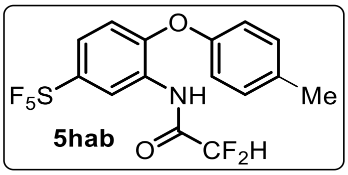

Following the general method **2C** (5-(Pentafluoro-λ^6-sulfaneyl)-2-(*p*-tolyloxy)-3*H*-azepine **3hb** (32.5 mg, 0.10 mmol), difluoroacetic anhydride (0.11 mL, 1.0 mmol) in 1 mL anhydrous acetonitrile were used) compound **5hab** was obtained as a white solid, yield 96% (38.7 mg), mp: 84.2–85.1 °C; **^{1}H NMR** (500 MHz, $CDCl_3$) δ 8.95 (d, J = 2.7 Hz, 1H), 8.71 (s, 1H), 7.44 (dd, J = 9.1, 2.7 Hz, 1H), 7.25 (dd, J = 8.7, 0.9 Hz, 2H), 7.07 – 6.87 (m, 2H), 6.76 (d, J = 9.1 Hz, 1H), 6.06 (t, J = 54.2 Hz, 1H), 2.39 (s, 3H); **^{13}C NMR** (126 MHz, $CDCl_3$) δ 160.6 (t, J = 24.9 Hz), 151.8, 149.5, 148.0 (p, J = 18.0 Hz), 135.9, 131.1, 126.2, 123.4, 120.40 118.9, 114.4, 108.4 (t, J = 254.3 Hz), 21.0; **^{19}F NMR** (658 MHz, $CDCl_3$) δ 83.89 (p, J = 150.7 Hz, 1F), 63.26 (d, J = 150.6 Hz, 4F), -126.18 (dd, J = 54.5, 1.9 Hz, 2F); **HRMS (ESI)**: calcd for $C_{15}H_{13}F_7NO_2S$ $[M + H]^+$: 404.0555, found: 404.0558; **IR (KBr)**: 3406, 1720, 1607, 1541, 1479, 1218, 1095, 900 cm^{-1}.

***N*-(2-([1,1'-biphenyl]-4-yloxy)-5-(pentafluoro-λ^6-sulfaneyl)phenyl)-2,2-difluoroacetamide (5hac)**

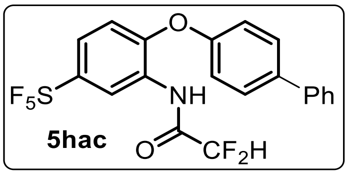

Following the general method **2C** (2-([1,1'-Biphenyl]-4-yloxy)-5-(pentafluoro-λ^6-sulfaneyl)-3*H*-azepine **3ho** (38.7 mg, 0.10 mmol), difluoroacetic anhydride (0.11 mL, 1.0 mmol) in 1 mL anhydrous acetonitrile were used) compound **5hac** was obtained as a white solid, yield 95% (44.3 mg), mp: 121.8 – 122.7 °C; **^{1}H NMR** (500 MHz, $CDCl_3$) δ 8.98 (d, J = 2.6 Hz, 1H), 8.71 (s, 1H), 7.69 – 7.64 (m, 2H), 7.61 – 7.57 (m, 2H), 7.50 – 7.45 (m, 3H), 7.41 – 7.35 (m, 1H), 7.20 – 7.14 (m, 2H), 6.88 (d, J = 9.1 Hz, 1H), 6.07 (t, J = 54.1 Hz, 1H); **^{13}C NMR** (126 MHz,

CDCl3) δ 160.6 (t, J = 25.0 Hz), 153.6, 149.1, 148.3 (p, J = 18.2 Hz), 140.0, 139.2, 129.2 (d, J = 18.8 Hz), 127.8, 127.2, 126.5, 123.5, 120.7, 119.1, 115.0, 108.4 (t, J = 254.4 Hz); **^{19}F NMR** (658 MHz, $CDCl_3$) δ 83.75 (p, J = 150.7 Hz, 1F), 63.24 (d, J = 150.5 Hz, 4F), -126.16 (dd, J = 54.2, 2.2 Hz, 2F); **HRMS (ESI)**: calcd for $C_{20}H_{14}F_7NO_2S$ $[M + H]^+$:466.0712, found: 466.0720; **IR (KBr)**: 3327, 3093, 3031, 1883, 1696, 1549, 1481,1255, 1093, 936 cm^{-1}.

***N*-(2-(4-bromophenoxy)-5-(pentafluoro-λ^6-sulfaneyl)phenyl)-2,2-difluoroacetamide (5had)**

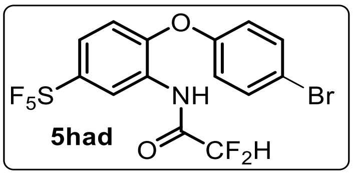

Following the general method **2C** (2-(4-Bromophenoxy)-5-(pentafluoro-λ^6-sulfaneyl)-3*H*-azepine **3hi** (39.0 mg, 0.10 mmol), difluoroacetic anhydride (0.11 mL, 1.0 mmol) in 1 mL anhydrous acetonitrile were used) compound **5had** was obtained as a yellow oil, yield 79% (37.0 mg), **^{1}H NMR** (500 MHz, $CDCl_3$) δ 8.96 (d, J = 2.6 Hz, 1H), 8.62 (s, 1H), 7.57 (d, J = 8.9 Hz, 2H), 7.48 (dd, J = 9.1, 2.6 Hz, 1H), 7.00 (d, J = 9.0 Hz, 2H), 6.80 (d, J = 9.1 Hz, 1H), 6.06 (t, J = 54.2 Hz, 1H); **^{13}C NMR** (126 MHz, $CDCl_3$) δ 160.6 (t, J = 25.1 Hz), 153.4, 148.7, 133.7, 126.59, 123.6, 122.1, 120.3, 119.3, 119.0, 115.0, 108.3 (t, J = 254.3 Hz); **^{19}F NMR** (658 MHz, $CDCl_3$) δ 83.45 (p, J = 150.8 Hz, 1F), 63.14 (d, J = 150.5 Hz, 4F), -126.15 (dd, J = 55.1, 2.4 Hz, 2F); **HRMS (ESI)**: calcd for $C_{14}H_8BrF_7NO_2S$ $[M - H]^-$:465.9347, found: 465.9348; **IR (KBr)**: 3410, 1719, 1542, 1478, 1222, 1092, 928, 842, 596 cm^{-1}.

6. General procedure for the synthesis of substituted 2-phenoxy-5-(tetrafluoro(phenylethynyl)-λ^6-sulfaneyl)-3*H*-azepine (8) [Method D]

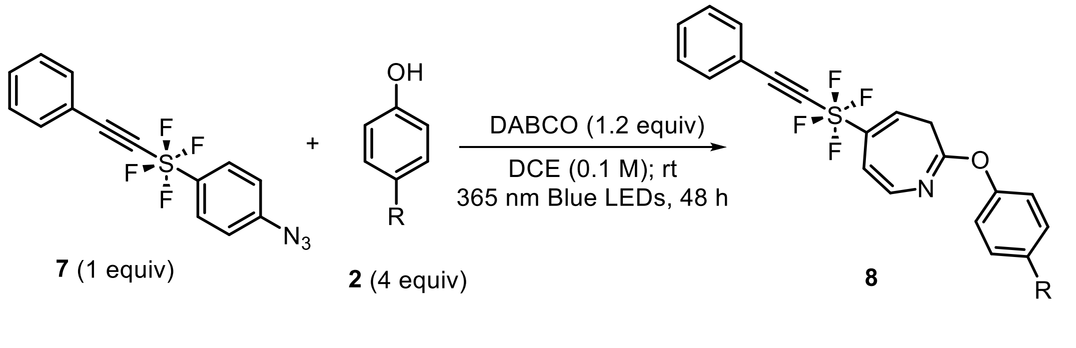

In an oven-dried screw-cap vial equipped with a magnetic stir bar, substituted azido benzene (**7**) (0.20 mmol, 1 equiv), phenol (**2**) (0.80 mmol, 4 equiv), and DABCO (0.24 mmol, 1.2 equiv) were dissolved in anhydrous DCE (2 mL, 0.1 M). The reaction mixture was stirred and irradiated with blue LED light (365 nm) for 48 hours. Afterward, the solvent was removed

under reduced pressure, and the crude was purified by column chromatography using *n*-hexane/ethyl acetate to afford pure compound (**8**).

2-Phenoxy-5-(tetrafluoro(phenylethynyl)-λ^6-sulfaneyl)-3*H*-azepine (8a)

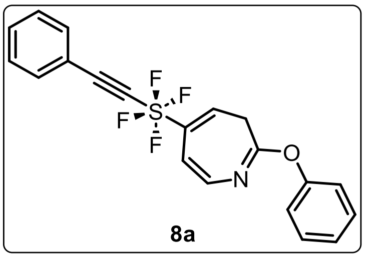

Following the general method **D** ((4-azidophenyl)tetrafluoro(phenylethynyl)-λ^6-sulfane **7** (65.4 mg, 0.20 mmol), phenol **2a** (75.3 mg, 0.80 mmol), and DABCO (26.9 mg, 0.24 mmol) in 2 mL anhydrous DCE were used) compound **8a** was obtained as a white solid, yield 75% (59.0 mg), mp: 118.6 – 120.6 °C; **^{1}H NMR** (500 MHz, $CDCl_3$) δ 7.56 (d, *J* = 7.9 Hz, 2H), 7.45-7.41 (m, 1H), 7.40-7.34 (m, 4H), 7.21 (ddd, *J* = 7.5, 2.6, 1.3 Hz, 1H), 7.05-7.00 (m, 3H), 6.47 (dd, *J* = 8.7, 2.2 Hz, 1H), 6.12 (td, *J* = 7.5, 1.3 Hz, 1H), 2.87 (s, 2H); **^{13}C NMR** (126 MHz, $CDCl_3$) δ 159.3 (p, *J* = 20.9 Hz), 152.9, 150.8, 139.3, 132.6, 130.3, 129.6, 128.6, 125.8, 121.3, 118.9, 111.48-111.29 (m), 116.8, 95.2 (p, *J* = 54.4 Hz), 72.8-72.4 (m), 31.6; **^{19}F NMR** (282 MHz, $CDCl_3$) δ 86.80 (s, 4F); **HRMS (ESI)**: calcd for $C_{20}H_{16}F_4NOS$ $[M + H]^+$: 394.0889, found: 394.0895; **IR (KBr)**: 3082, 2921, 2217, 1632, 1488, 1327, 1251, 1200, 883, 854, 776, 728 cm^{-1}.

5-(Tetrafluoro(phenylethynyl)-λ^6-sulfaneyl)-2-(*p*-tolyloxy)-3*H*-azepine (8b)

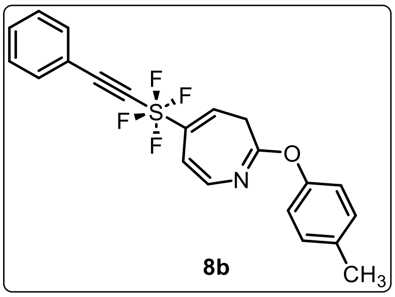

Following the general method **D** ((4-azidophenyl)tetrafluoro(phenylethynyl)-λ^6-sulfane **7** (65.4 mg, 0.20 mmol), 4-methylphenol **2b** (86.5 mg, 0.80 mmol), and DABCO (26.9 mg, 0.24 mmol) in 2 mL anhydrous DCE were used) compound **8b** was obtained as a white solid, yield 67% (54.5 mg), mp: 119.8 – 121.7 °C; **^{1}H NMR** (500 MHz, $CDCl_3$) δ 7.58-7.54 (m, 2H), 7.45-7.41 (m, 1H), 7.39-7.35 (m, 2H), 7.16 (d, *J* = 8.2 Hz, 2H), 7.02 (d, *J* = 8.7 Hz, 1H), 6.92-6.88 (m, 2H), 6.45 (dd, *J* = 8.7, 0.8 Hz, 1H), 6.11 (td, *J* = 7.4, 0.7 Hz, 1H), 2.86 (s, 2H), 2.33 (s, 3H); **^{13}C NMR** (126 MHz, $CDCl_3$) δ 159.3 (p, *J* = 20.9 Hz), 151.2, 150.7, 139.4, 135.5, 132.6, 130.3,

130.2, 128.6, 121.0, 118.9, 116.9-116.7 (m), 111.2, 95.2 (p, J = 54.5 Hz), 72.5 (p, J = 10.3 Hz), 31.6, 21.0; **^{19}F NMR** (658 MHz, $CDCl_3$) δ 86.78 (s, 4F); **HRMS (ESI)**: calcd for $C_{21}H_{18}F_4NOS$ $[M + H]^+$: 408.1045, found: 408.1051; **IR (KBr)**: 3074, 2924, 2214, 1631, 1503, 1326, 1252, 1202, 883, 853, 774 cm^{-1}.

2-(4-methoxyphenoxy)-5-(tetrafluoro(phenylethynyl)-λ^6-sulfaneyl)-3*H*-azepine (8c)

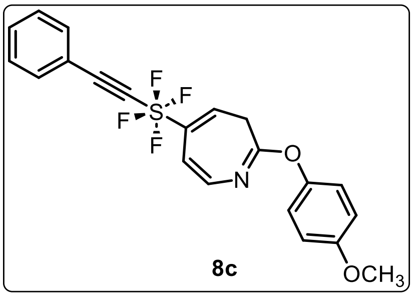

8c

Following the general method **D** ((4-azidophenyl)tetrafluoro(phenylethynyl)-λ^6-sulfane **7** (65.4 mg, 0.20 mmol), 4-methoxyphenol **2c** (99.3 mg, 0.80 mmol), and DABCO (26.9 mg, 0.24 mmol) in 2 mL anhydrous DCE were used) compound **8c** was obtained as a white solid, yield 66% (55.8 mg), mp: 105.0 – 107.9 °C; **^{1}H NMR** (500 MHz, $CDCl_3$) δ 7.58-7.54 (m, 2H), 7.45-7.40 (m, 1H), 7.39-7.34 (m, 2H), 7.02 (d, J = 8.7 Hz, 1H), 6.97-6.92 (m, 2H), 6.90-6.85 (m, 2H), 6.45 (dd, J = 8.7, 1.0 Hz, 1H), 6.10 (td, J = 7.5, 0.8 Hz, 1H), 3.78 (s, 3H), 2.85 (s, 2H); **^{13}C NMR** (126 MHz, $CDCl_3$) δ 159.3 (p, J = 21.0 Hz), 157.2, 151.5, 146.4, 139.4, 132.6, 130.3, 128.6, 122.1, 118.9, 116.9-116.8 (m), 114.6, 111.3-111.1 (m), 95.2 (p, J = 54.5 Hz), 72.5 (p, J = 9.8 Hz), 55.7, 31.6; **^{19}F NMR** (282 MHz, $CDCl_3$) δ 86.78 (s, 4F); **HRMS (ESI)**: calcd for $C_{21}H_{18}F_4NO_2S$ $[M + H]^+$: 424.0994, found: 424.1006; **IR (KBr)**: 3088, 2937, 2226, 1633, 1503, 1327, 1242, 883, 859, 775, 732 cm^{-1}.

2-(4-fluorophenoxy)-5-(tetrafluoro(phenylethynyl)-λ^6-sulfaneyl)-3*H*-azepine (8d)

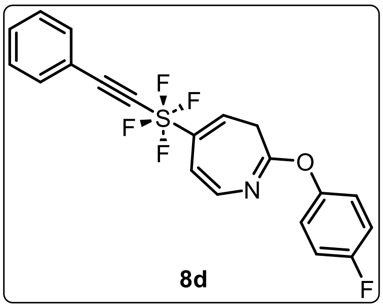

8d

Following the general method **D** ((4-azidophenyl)tetrafluoro(phenylethynyl)-λ^6-sulfane **7** (65.4 mg, 0.20 mmol), 4-fluorophenol **2g** (89.7 mg, 0.80 mmol), and DABCO (26.9 mg, 0.24 mmol) in 2 mL anhydrous DCE were used) compound **8d** was obtained as a white solid, yield 66%

(54.3 mg), mp: 115.4 – 117.6 °C; **^{1}H NMR** (500 MHz, $CDCl_3$) δ 7.57-7.54 (m, 2H), 7.45-7.40 (m, 1H), 7.39-7.34 (m, 2H), 7.07-7.03 (m, 2H), 7.02-6.97 (m, 3H), 6.47 (dd, J = 8.7, 1.0 Hz, 1H), 6.11 (td, J = 7.5, 0.8 Hz, 1H), 2.86 (s, 2H); **^{13}C NMR** (126 MHz, $CDCl_3$) δ 160.1 (d, J = 244.3 Hz), 159.7-158.9 (m), 151.0, 148.7 (d, J = 2.7 Hz), 139.1, 132.6, 130.3, 128.6, 122.8 (d, J = 8.5 Hz), 117.0-116.8 (m), 116.3 (d, J = 23.5 Hz), 118.9, 111.7-111.5 (m), 95.2 (p, J = 54.3 Hz), 72.8-72.5 (m), 31.5; **^{19}F NMR** (282 MHz, $CDCl_3$) δ 86.81 (s, 4F), -117.52 (s, 1F); **HRMS (ESI)**: calcd for $C_{20}H_{15}F_5NOS$ $[M + H]^+$: 412.0795, found: 412.0809; **IR (KBr)**: 3082, 2920, 2219, 1636, 1501, 1325, 1254, 1190, 883, 854, 775, 730 cm^{-1}.

2-(4-bromophenoxy)-5-(tetrafluoro(phenylethynyl)-λ^6-sulfaneyl)-3*H*-azepine (8e)

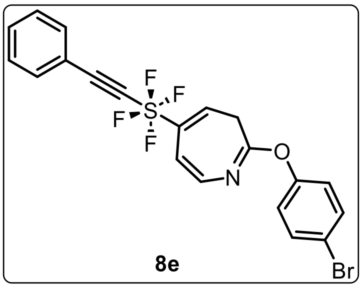

Following the general method **D** ((4-azidophenyl)tetrafluoro(phenylethynyl)-λ^6-sulfane **7** (65.4 mg, 0.20 mmol), 4-bromophenol **2i** (138.4 mg, 0.80 mmol), and DABCO (26.9 mg, 0.24 mmol) in 2 mL anhydrous DCE were used) compound **8e** was obtained as a pale yellow solid, yield 63% (59.5 mg), mp: 164.3 – 166.4 °C; **^{1}H NMR** (500 MHz, $CDCl_3$) δ 7.57-7.54 (m, 2H), 7.49-7.45 (m, 2H), 7.45-7.40 (m, 1H), 7.39-7.34 (m, 2H), 7.00 (d, J = 8.7 Hz, 1H), 6.94-6.90 (m, 2H), 6.48 (dd, J = 8.7, 1.0 Hz, 1H), 6.11 (td, J = 7.5, 0.8 Hz, 1H), 2.85 (s, 2H); **^{13}C NMR** (126 MHz, $CDCl_3$) δ 159.3 (p, J = 21.0 Hz), 151.8, 150.5, 138.9, 132.7, 132.6, 130.3, 128.6, 123.2, 118.9, 117.1-116.8 (m), 111.8, 95.1 (p, J = 54.3 Hz), 72.7 (p, J = 9.8 Hz), 31.4; **^{19}F NMR** (658 MHz, $CDCl_3$) δ 86.82 (s, 4F); **HRMS (ESI)**: calcd for $C_{20}H_{15}F_4BrNOS$ $[M + H]^+$: 471.9994, found: 471.9994; **IR (KBr)**: 3089, 2925, 2218, 1634, 1538, 1323, 1243, 1207, 888, 856, 772 cm^{-1}.

Ethyl 4-((5-(tetrafluoro(phenylethynyl)-λ^6-sulfaneyl)-3*H*-azepin-2-yl)oxy)benzoate (8f)

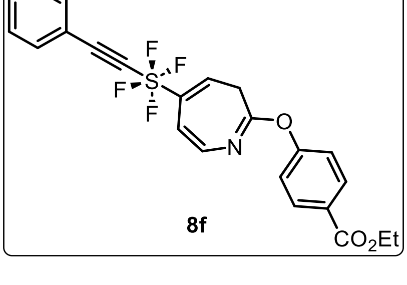

Following the general method **D** ((4-azidophenyl)tetrafluoro(phenylethynyl)-λ^6-sulfane **7** (65.4 mg, 0.20 mmol), ethyl 4-hydroxybenzoate **2n** (132.9 mg, 0.80 mmol), and DABCO (26.9 mg, 0.24 mmol) in 2 mL anhydrous DCE were used) compound **8f** was obtained as a white solid, yield 53% (49.3 mg), mp: 95.8 – 97.6 °C; **^{1}H NMR** (500 MHz, $CDCl_3$) δ 8.09-8.04 (m, 2H), 7.59-7.55 (m, 2H), 7.46-7.41 (m, 1H), 7.40-7.35 (m, 2H), 7.11-7.07 (m, 2H), 7.01 (d, *J* = 8.7 Hz, 1H), 6.51 (dd, *J* = 8.7, 0.9 Hz, 1H), 6.15-6.11 (m, 1H), 4.36 (q, *J* = 7.1 Hz, 2H), 2.88 (s, 2H), 1.38 (t, *J* = 7.1 Hz, 3H); **^{13}C NMR** (126 MHz, $CDCl_3$) δ 165.9, 159.3 (p, *J* = 21.3 Hz), 156.3, 149.9, 138.9, 132.6, 131.3, 130.3, 128.6, 127.9, 121.3, 118.9, 117.0-116.8 (m), 111.9, 95.1 (p, *J* = 54.3 Hz), 72.7 (p, *J* = 9.7 Hz), 61.2, 31.5, 14.5; **^{19}F NMR** (282 MHz, $CDCl_3$) δ 86.89 (s, 4F); **HRMS (ESI)**: calcd for $C_{23}H_{20}F_4NO_3S$ $[M + H]^+$: 466.1100, found: 466.1102; **IR (KBr)**: 3434, 3068, 2978, 2216, 1724, 1637, 1542, 1328, 1247, 1201, 882, 854, 709 cm^{-1}.

2-([1,1'-Biphenyl]-4-yloxy)-5-(tetrafluoro(phenylethynyl)-λ^6-sulfaneyl)-3*H*-azepine (8g)

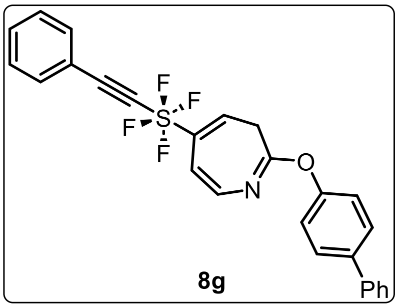

Following the general method **D** ((4-azidophenyl)tetrafluoro(phenylethynyl)-λ^6-sulfane **7** (65.4 mg, 0.20 mmol), 4-phenylphenol **2o** (136.2 mg, 0.80 mmol), and DABCO (26.9 mg, 0.24 mmol) in 2 mL anhydrous DCE were used) compound **8g** was obtained as a white solid, yield 62% (58.2 mg), mp: 122.2 – 124.3 °C; **^{1}H NMR** (500 MHz, $CDCl_3$) δ 7.59-7.56 (m, 3H), 7.56-7.53 (m, 3H), 7.45-7.40 (m, 3H), 7.39-7.31 (m, 3H), 7.12-7.08 (m, 2H), 7.05 (d, *J* = 8.7 Hz, 1H), 6.49 (dd, *J* = 8.7, 1.0 Hz, 1H), 6.14 (td, *J* = 7.4, 0.7 Hz, 1H), 2.89 (s, 2H); **^{13}C NMR** (126 MHz, $CDCl_3$) δ 159.3 (p, *J* = 21.0 Hz), 152.3, 150.9, 140.5, 139.3, 138.9, 132.6, 130.3, 128.9, 128.6, 128.4, 127.4, 127.2, 121.6, 118.9, 117.0-116.8 (m), 111.5, 95.2 (p, *J* = 54.3 Hz), 72.6 (p, *J* = 9.8 Hz), 31.6; **^{19}F NMR** (282 MHz, $CDCl_3$) δ 86.85 (s, 4F); **HRMS (ESI)**: calcd for $C_{26}H_{20}F_4NOS$ $[M + H]^+$: 470.1202, found: 470.1203; **IR (KBr)**: 3034, 2925, 2213, 1632, 1539, 1326, 1247, 1203, 883, 853, 727 cm^{-1}.

7. Synthetic transformations of 2-phenoxy-5-(tetrafluoro(phenylethynyl)-λ^6-sulfaneyl)-3*H*-azepine (8a)

2,2,2-Trifluoro-*N*-(2-phenoxy-4-(tetrafluoro(phenylethynyl)-λ^6-sulfaneyl)phenyl)acetamide (9a)

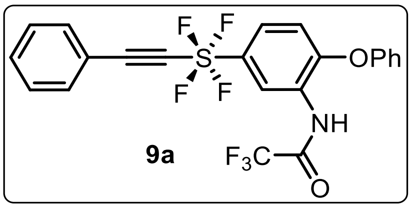

Following the general method **1C** (2-phenoxy-5-(tetrafluoro(phenylethynyl)-λ^6-sulfaneyl)-3*H*-azepine **8a** (39.3 mg, 0.10 mmol), TFAA (0.14 mL, 1.0 mmol) in 1 mL anhydrous acetonitrile were used) compound **9a** was obtained as a white solid, yield 89% (43.5 mg), mp: 130.9 – 132.5 °C; **^{1}H NMR** (500 MHz, $CDCl_3$) δ 8.93 (d, *J* = 2.6 Hz, 1H), 8.62 (s, 1H), 7.59-7.56 (m, 2H), 7.53 (dd, *J* = 9.1, 2.7 Hz, 1H), 7.48-7.42 (m, 3H), 7.40-7.36 (m, 2H), 7.31-7.27 (m, 1H), 7.13-7.09 (m, 2H), 6.77 (d, *J* = 9.1 Hz, 1H); **^{13}C NMR** (126 MHz, $CDCl_3$) δ 154.8 (q, *J* = 38.0 Hz), 154.3, 153.7 (p, *J* = 24.6 Hz), 148.5, 132.6, 130.6, 130.4, 128.6, 125.9, 125.4, 124.1-123.9 (m), 120.4, 119.4-119.1 (m), 118.8, 115.6 (q, *J* = 288.6 Hz), 114.7, 95.2 (p, *J* = 54.2 Hz), 72.9 (p, *J* = 9.9 Hz); **^{19}F NMR** (282 MHz, $CDCl_3$) δ 88.66 (s, 4F), -76.20 (s, 3F); **HRMS (ESI)**: calcd for $C_{22}H_{14}F_7NO_2SNa$ [M + Na]$^+$: 512.0531, found: 512.0533; **IR (KBr)**: 3348, 2219, 1722, 1545, 1480, 1224, 1173, 1156, 908, 847, 790, 757 cm^{-1}.

2,2-difluoro-*N*-(2-phenoxy-5-(tetrafluoro(phenylethynyl)- λ^6-sulfaneyl)phenyl)acetamide (9b)

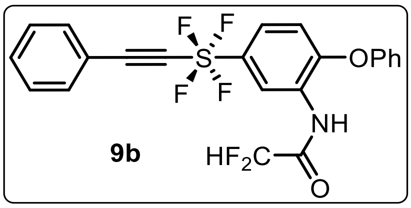

Following the general method **2C** (2-phenoxy-5-(tetrafluoro(phenylethynyl)-λ^6-sulfaneyl)-3*H*-azepine **8a** (39.3 mg, 0.10 mmol), difluoroacetic anhydride (0.11 mL, 1.0 mmol) in 1 mL anhydrous acetonitrile were used) compound **9b** was obtained as white solid, yield 88% (41.5 mg), mp 118.7–119.6 °C; **^{1}H NMR** (500 MHz, $CDCl_3$) δ 8.98 (d, *J* = 2.7 Hz, 1H), 8.65 (s, 1H), 7.59 – 7.55 (m, 2H), 7.50 (dd, *J* = 9.1, 2.7 Hz, 1H), 7.44 (ddd, *J* = 9.0, 5.4, 1.9 Hz, 3H), 7.38 (t, *J* = 7.4 Hz, 2H), 7.30 – 7.21 (m, 1H), 7.15 – 7.06 (m, 2H), 6.77 (d, *J* = 9.0 Hz, 1H), 6.05 (t, *J* = 54.2 Hz, 1H); **^{13}C NMR** (126 MHz, $CDCl_3$) δ 160.5 (t, *J* = 24.8 Hz), 154.6, 153.8 (p, *J* = 23.9 Hz), 148.3, 132.6, 130.4 (d, *J* = 21.5 Hz), 128.6, 126.1, 125.7, 123.4, 120.3, 119.0 (d, *J* = 23.3 Hz), 114.8, 108.4 (t, *J* = 254.3 Hz), 95.3 (p, *J* = 54.4 Hz); **^{19}F NMR** (658 MHz, $CDCl_3$) δ 88.62 (s, 4F), -126.17 (dd, *J* = 54.1, 2.3 Hz, 2F); **HRMS (ESI)**: calcd for $C_{22}H_{15}F_6NO_2SNa$ [M + Na]$^+$:494.0625, found: 494.0620; **IR (KBr)**: 3343, 2217, 1707, 1480, 1218, 1092, 855, 789, 730 cm^{-1}.

General procedure for synthesis of 5-((3,3-difluoro-2-phenylcycloprop-1-en-1-yl)tetrafluoro-λ[6]-sulfaneyl)-2-phenoxy-3*H*-azepine (10)

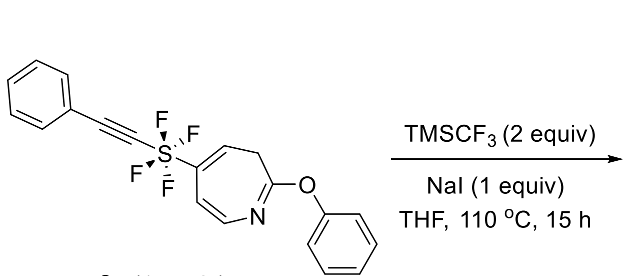

8a (1 equiv) **10**

An oven dried 4 mL pressure tube was charged with 2-phenoxy-5-(tetrafluoro(phenylethynyl)-λ[6]-sulfaneyl)-3*H*-azepine **8a** (39.3 mg, 0.1 mmol), $TMSCF_3$ (0.029 mL, 0.2 mmol), sodium iodide (14.9 mg, 0.1 mmol), and THF (0.5 mL) at room temperature. Then, the reaction mixture was heated at 110 °C with oil bath for 15 h.[6] The reaction was quenched by adding $Na_2S_2O_4$ aq. solution and extracted with diethyl ether (3 times). The organic layer was washed with brine and dried over with Na_2SO_4. After filtrated and transferred to round-bottom flask, evaporated the solvent in vacuo to obtain the crude product. The crude product was purified by column chromatography using *n*-hexane/ethyl acetate to afford pure compound **10** as a white solid, yield 75% (33.2 mg), mp: 68.4 – 70.7 °C; **^{1}H NMR** (500 MHz, $CDCl_3$) δ 7.73-7.69 (m, 2H), 7.53-7.48 (m, 1H), 7.46-7.42 (m, 2H), 7.33-7.28 (m, 2H), 7.16-7.12 (m, 1H), 7.00 (d, *J* = 8.7 Hz, 1H), 6.97- 6.94 (m, 2H), 6.46 (dd, *J* = 8.7, 0.9 Hz, 1H), 6.15 (td, *J* = 7.5, 0.8 Hz, 1H), 2.84 (s, 2H); **^{13}C NMR** (126 MHz, $CDCl_3$) δ 158.3 (p, *J* = 19.1 Hz), 152.8, 150.8, 133.9-32.5 (m), 139.7, 133.1, 131.4, 129.7, 129.4, 126.9-126.6 (m), 125.9, 121.4, 121.3, 117.6-117.4 (m), 111.0-110.8 (m), 99.9 (t, *J* = 279.7 Hz), 31.7; **^{19}F NMR** (282 MHz, $CDCl_3$) δ 72.37 (s, 4F), -103.33 (s, 2F); **HRMS (ESI)**: calcd for $C_{21}H_{16}F_6NOS$ $[M + H]^+$: 444.0857, found: 444.0864; **IR (KBr)**: 3064, 2925, 1738, 1321, 1244, 1196, 1064, 763 cm^{-1}.

General procedure for synthesis of 5-((1,5-diphenyl-1*H*-1,2,3-triazol-4-yl)tetrafluoro-λ[6]-sulfaneyl)-2-phenoxy-3*H*-azepine (11)

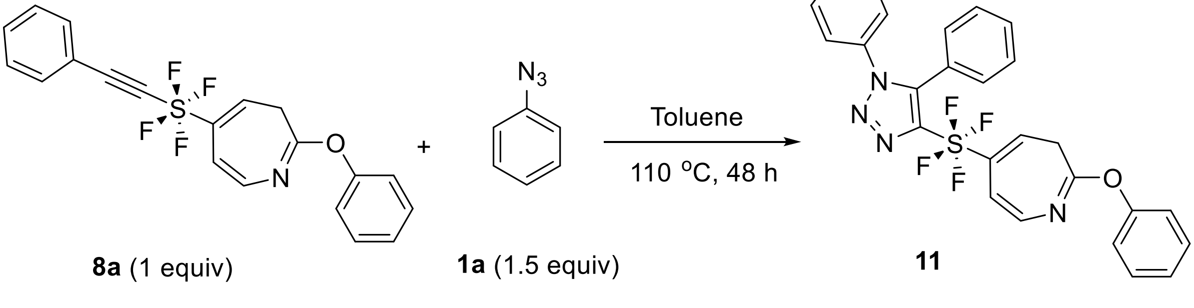

8a (1 equiv) **1a** (1.5 equiv) **11**

An oven-dried test tube was charged with 2-phenoxy-5-(tetrafluoro(phenylethynyl)-λ^6-sulfaneyl)-3*H*-azepine **8a** (39.3 mg, 0.1 mmol), phenyl azide **1a** (17.8 mg, 0.15 mmol), and toluene (0.5 mL) and allowed to stir at 110 °C for 48 h.[7] The reaction was allowed to cool to room temperature and the solvent was evaporated in vacuo to give the crude products. The crude product was purified by column chromatography using *n*-hexane/ethyl acetate to afford pure compound **11** as a pale yellow solid, yield 47% (24.0 mg), mp: 164.3 – 166.4 °C; **^{1}H NMR** (500 MHz, $CDCl_3$) δ 7.43-7.39 (m, 1H), 7.39-7.34 (m, 5H), 7.34-7.31 (m, 2H), 7.30-7.23 (m, 4H), 7.21-7.16 (m, 1H), 7.01-6.96 (m, 3H), 6.48 (dd, *J* = 8.7, 0.8 Hz, 1H), 6.12 (td, *J* = 7.4, 0.6 Hz, 1H), 2.83 (s, 2H); **^{13}C NMR** (126 MHz, $CDCl_3$) δ 160.7 (p, *J* = 34.9 Hz), 159.7 (p, *J* = 21.5 Hz), 152.9, 150.7, 139.0, 136.2, 133.7, 130.6, 129.8, 129.6, 129.6, 129.3, 128.5, 126.8, 125.7, 125.5, 121.3, 116.9-116.6 (m), 111.7, 31.6; **^{19}F NMR** (282 MHz, $CDCl_3$) δ 70.55 (s, 4F); **HRMS (ESI)**: calcd for $C_{26}H_{21}F_4N_4OS$ $[M + H]^+$ 513.1372, found: 513.1380; **IR (KBr)**: 3092, 2923, 1636, 1589, 1492, 1324, 1247, 1199, 782, 764, 729 cm^{-1}.

General procedure for synthesis of 2-methyl-3,4-diphenyl-5-(tetrafluoro(2-phenoxy-3*H*-azepin-5-yl)-λ^6-sulfaneyl)-2,3-dihydroisoxazole (13)

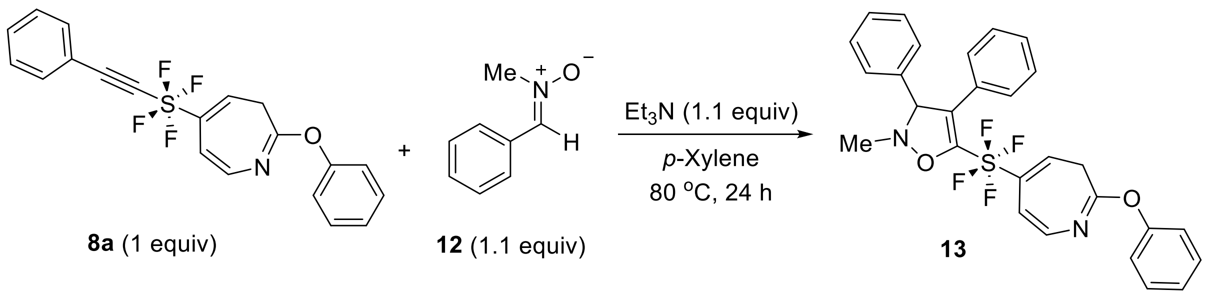

An oven-dried test tube was charged with 2-phenoxy-5-(tetrafluoro(phenylethynyl)-λ^6-sulfaneyl)-3*H*-azepine **8a** (39.3 mg, 0.1 mmol), nitrone **12** (14.8 mg, 0.11 mmol), Et_3N (0.015 mL, 0.11 mmol) and *p*-xylene (0.5 mL) and allowed to stir at 80 °C for 24 h.[8] The reaction mixture was evaporated under reduced pressure to give the crude product, which was purified by neutral silica column chromatography using *n*-hexane/ethyl acetate/Et_3N to afford pure compound **13** as a white solid, yield 43% (22.7 mg), mp: 113.4 – 115.3 °C; **^{1}H NMR** (500 MHz, $CDCl_3$) δ 7.75-7.72 (m, 2H), 7.49-7.43 (m, 5H), 7.40 (t, *J* = 7.6 Hz, 2H), 7.36-7.30 (m, 3H), 7.19-7.14 (m, 1H), 6.97-6.94 (m, 2H), 6.87 (d, *J* = 8.8 Hz, 1H), 6.32 (dd, *J* = 8.8, 0.9 Hz, 1H), 5.91 (td, *J* = 7.5, 0.8 Hz, 1H), 5.14 (s, 1H), 3.08 (s, 3H), 2.72 (s, 2H); **^{13}C NMR** (126 MHz, $CDCl_3$) δ 161.2 (p, *J* = 23.0 Hz), 152.9, 152.8, 150.9, 140.7, 138.6, 134.2 (t, *J* = 31.3 Hz), 130.6, 129.9, 129.5, 128.8, 128.3, 128.2, 127.4, 125.7, 121.3, 116.1, 112.0, 77.9, 46.9, 31.3; **^{19}F NMR** (658 MHz, $CDCl_3$) δ 74.92 (s, 4F); **HRMS (ESI)**: calcd for $C_{28}H_{24}F_4N_2O_2SNa$

[M + Na]$^+$: 551.1392, found: 551.1415; **IR (KBr)**: 3027, 2923, 1633, 1489, 1321, 1246, 1199, 778, 715 cm^{-1}.

8. Computational details

Calculation of global electrophilicity index values

In this section, all the DFT calculations were carried out using the Rowan platform.[9] Initial grounds-state structures of *para*-substituted phenyl azides (**1a**, **1e**, **1g**, and **1h**) and their corresponding ketenimine intermediates (**III-a**, **III-e**, **III-g**, and **III-h**) were drawn in the platform, followed by geometry optimization and frequencies calculations of them using AIMNet2[10] (putting "rapid" mode) level of theory. Then, all the optimized structures had no imaginary frequencies. After that, Fukui index calculation was conducted by GFN2-xTB[11] (putting "rapid" mode), giving their global electrophilicity index (GEI)[12] values which were shown below

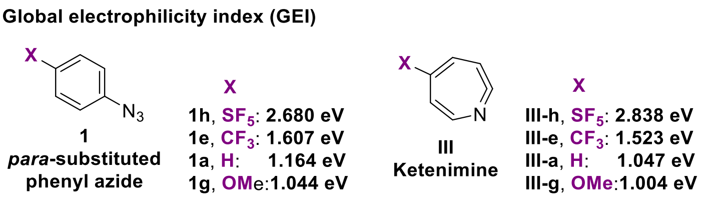

Cartesian coordinates of optimized structures and their energies data

1a

Electronic Energy = -396.1123160 Eh
Zero-point Energy = 0.1042650 Eh
Thermal Energy Correction = 0.1112570 Eh
Enthalpy Correction = 0.1122010 Eh
Gibbs Free Energy Correction = 0.0718620 Eh
Total Thermal Energy = -396.0010590 Eh
Total Enthalpy = -396.0001150 Eh
Total Gibbs Free Energy = -396.0404540 Eh

H	0.61783214	-1.07868892	-0.61860903
C	0.58580406	-1.11668231	-1.69763831
C	1.34891798	-0.23599720	-2.45281059
C	1.31072635	-0.27774982	-3.83501491
C	0.50439725	-1.21572724	-4.46900821
C	-0.26165654	-2.10352135	-3.72475104
C	-0.21853684	-2.04638430	-2.34007864
H	1.97860124	0.48890809	-1.95747372
H	1.89458309	0.40439824	-4.43536902
N	0.52659081	-1.19285432	-5.89134380
H	-0.88496897	-2.83307297	-4.21859772
H	-0.81962853	-2.73537901	-1.76520543
N	-0.18090166	-2.03093451	-6.45202333
N	-0.78761074	-2.75029338	-7.05805731

1e

Electronic Energy = -733.3774290 Eh
Zero-point Energy = 0.1088520 Eh
Thermal Energy Correction = 0.1195360 Eh
Enthalpy Correction = 0.1204800 Eh
Gibbs Free Energy Correction = 0.0716170 Eh
Total Thermal Energy = -733.2578930 Eh
Total Enthalpy = -733.2569490 Eh
Total Gibbs Free Energy = -733.3058120 Eh

C	0.58569477	-1.06561531	-1.65823321
C	1.72504255	-1.32007817	-2.41600730
C	1.64564459	-1.33303890	-3.79131361
C	0.42195519	-1.09095086	-4.41845330
C	-0.71890825	-0.83421134	-3.65273808
C	-0.63356435	-0.82221521	-2.27588155
H	2.66520662	-1.50654654	-1.92041367
H	2.51675512	-1.52850124	-4.39551582
N	0.39507433	-1.11704763	-5.78847245
H	-1.66473523	-0.64608956	-4.13965508
H	-1.51129788	-0.62493229	-1.68038083
N	-0.49558528	-0.95356831	-6.55971936
N	-1.17433827	-0.83678366	-7.44553906
C	0.70990901	-1.06166490	-0.16298927
F	-0.44611192	-0.82010952	0.46851374
F	1.16288091	-2.23835442	0.31051274
F	1.58158293	-0.13076472	0.27050425

1g

Electronic Energy = -510.7088700 Eh
Zero-point Energy = 0.1361550 Eh
Thermal Energy Correction = 0.1459170 Eh
Enthalpy Correction = 0.1468610 Eh
Gibbs Free Energy Correction = 0.1004500 Eh
Total Thermal Energy = -510.5629530 Eh
Total Enthalpy = -510.5620090 Eh
Total Gibbs Free Energy = -510.6084200 Eh

C	0.49249429	-1.04504258	-1.72163578
C	-0.39615751	-0.32123341	-2.51112661
C	-0.35667807	-0.44027429	-3.88747610
C	0.56753597	-1.28109475	-4.49702376
C	1.46141455	-2.00959382	-3.70734581
C	1.41958181	-1.88896472	-2.33793203
H	-1.12413882	0.33879135	-2.06729541
H	-1.04404430	0.11956304	-4.50200281
N	0.56112704	-1.36002535	-5.87246431
H	2.18230412	-2.66502477	-4.17522677
H	2.10125204	-2.44320192	-1.71274639
N	1.23599353	-1.98954924	-6.61904290
N	1.74662685	-2.48448395	-7.49135389
O	0.54351820	-1.00557535	-0.36249335
C	-0.36271710	-0.17540648	0.32181396
H	-1.39987404	-0.47117001	0.13354782
H	-0.22807540	0.87661691	0.05027687
H	-0.13852087	-0.30499066	1.37927659

1h

Electronic Energy = -1293.2285620 Eh
Zero-point Energy = 0.1127260 Eh
Thermal Energy Correction = 0.1252590 Eh
Enthalpy Correction = 0.1262030 Eh
Gibbs Free Energy Correction = 0.0734200 Eh
Total Thermal Energy = -1293.1033030 Eh
Total Enthalpy = -1293.1023590 Eh
Total Gibbs Free Energy = -1293.1551420 Eh
C -2.56334591 -1.88671590 -0.16682591
C -2.18550525 -1.23402216 -1.34726926
C -1.49518444 -0.03868188 -1.27858845
C -1.19623305 0.48052789 -0.03909501
C -1.55348505 -0.13651884 1.13967889
C -2.24364342 -1.33157135 1.07613803
N -3.24688628 -3.07023412 -0.17225009
H -2.43221559 -1.66242065 -2.30733230
H -1.19274393 0.48383081 -2.17005570
H -1.29504870 0.31216188 2.08343309
H -2.54122110 -1.84565427 1.97539650
S -0.27874580 2.06921259 0.04340286
F 0.79990785 1.59133812 -1.12660267
F -1.23042701 2.76396070 -1.12809131
F -1.28211072 2.67488219 1.22022173
F 0.52620114 3.46302257 0.11247535
F 0.74599542 1.50354453 1.22170868
N -3.63998953 -3.75202240 -1.06590836
N -4.08864794 -4.52971602 -1.73730982

III-a
Electronic Energy = -286.4845000 Eh
Zero-point Energy = 0.0931090 Eh
Thermal Energy Correction = 0.0983900 Eh
Enthalpy Correction = 0.0993330 Eh
Gibbs Free Energy Correction = 0.0636130 Eh
Total Thermal Energy = -286.3861100 Eh
Total Enthalpy = -286.3851670 Eh
Total Gibbs Free Energy = -286.4208870 Eh
H 0.85906480 -1.44185517 -0.90349603
C 0.71028421 -1.21110571 -1.95145312
C 1.49911354 -0.26947579 -2.50910244
C 1.26224108 0.15973567 -3.88871690
C 1.04504760 -0.91756731 -4.59836832
C -0.41413780 -2.33675835 -3.87710549
C -0.36616550 -1.94257194 -2.58604235
H 2.29810430 0.18695829 -1.93884404
H 1.38404156 1.16736289 -4.23350397
N 0.66507190 -2.09523842 -4.72350191
H -1.22440014 -2.93279044 -4.27535709
H -1.13567699 -2.30957756 -1.92018656

III-e
Electronic Energy = -623.7512090 Eh
Zero-point Energy = 0.0977280 Eh
Thermal Energy Correction = 0.1065100 Eh
Enthalpy Correction = 0.1074530 Eh
Gibbs Free Energy Correction = 0.0630800 Eh
Total Thermal Energy = -623.6446990 Eh

Total Enthalpy = -623.6437560 Eh
Total Gibbs Free Energy = -623.6881290 Eh
C 0.53548070 -1.20731934 -1.90680888
C 1.58740727 -0.50084846 -2.62517922
C 1.52215013 -0.06621757 -3.90021628
C -0.08175478 -1.37272564 -4.49927993
C -0.30179338 -2.46620460 -3.81161135
C -0.35398367 -2.10093275 -2.40090970
H 2.43763861 -0.20536774 -2.02617987
H 2.32361456 0.48422490 -4.37131207
N 0.33709580 -0.21320017 -4.61939100
H -0.53478799 -3.44503795 -4.17920179
H -1.08445098 -2.56572259 -1.75217895
C 0.53974223 -0.91563707 -0.42752197
F -0.49121293 -1.45883750 0.23223237
F 1.65515170 -1.37726813 0.17159961
F 0.50283321 0.40321500 -0.17005737

III-g
Electronic Energy = -401.0840430 Eh
Zero-point Energy = 0.1259990 Eh
Thermal Energy Correction = 0.1337690 Eh
Enthalpy Correction = 0.1347130 Eh
Gibbs Free Energy Correction = 0.0930840 Eh
Total Thermal Energy = -400.9502740 Eh
Total Enthalpy = -400.9493300 Eh
Total Gibbs Free Energy = -400.9909590 Eh
C 0.39958188 -0.97913164 -1.93063219
C -0.20468571 0.03924410 -2.59538467
C 0.25504713 0.39195915 -3.93825055
C 0.39710642 -0.71420630 -4.62389391
C 1.60972938 -2.26854343 -3.71205062
C 1.46630261 -1.83448142 -2.44325065
H -1.01888118 0.59840115 -2.15780187
H 0.29230021 1.39964498 -4.30333388
N 0.68640274 -1.92436876 -4.68649176
H 2.38362208 -2.96851842 -3.99676844
H 2.08106899 -2.27243660 -1.67029374
O 0.11675498 -1.32123789 -0.63996478
C -0.90698338 -0.63785213 0.03920213
H -1.87152187 -0.75677967 -0.46503200
H -0.67992323 0.42820376 0.14293297
H -0.95845722 -1.09328357 1.02679124

III-h
Electronic Energy = -1183.5978460 Eh
Zero-point Energy = 0.1008180 Eh
Thermal Energy Correction = 0.1115170 Eh
Enthalpy Correction = 0.1124610 Eh
Gibbs Free Energy Correction = 0.0640400 Eh
Total Thermal Energy = -1183.4863290 Eh
Total Enthalpy = -1183.4853850 Eh
Total Gibbs Free Energy = -1183.5338060 Eh
S 0.68959057 -0.10147438 -0.36988380
F 2.25205858 0.28753362 -0.79031781
F -0.85355102 -0.40653730 0.17948422
F 0.15214767 1.31592231 -1.05980139

F	1.23656479	-1.44561343	0.44755993
F	0.92690617	0.73419187	0.99047806
C	0.41928183	-1.07662544	-1.94464717
C	1.01243557	-0.45591297	-3.09115111
C	1.55294413	-1.12486273	-4.13966698
C	0.53328588	-3.00078261	-3.74105760
C	-0.54901475	-3.00816666	-3.00191225
C	-0.25445615	-2.22591807	-1.79574460
H	1.13592614	0.61415085	-3.03521854
H	2.02988084	-0.62034566	-4.96728303
N	1.60648242	-2.50795273	-4.11351180
H	-1.45691928	-3.55724782	-3.14456381
H	-0.58781156	-2.55513626	-0.82316608

Calculation of Gibbs energies which are relative to nitrene (I)

In this section, geometry optimization and frequency calculations were carried out with the Gaussian package.[13] All the structures were optimized using the long-range corrected hybrid M06-2X density functional[14] in combination with the Def2TZVP basis set.[15] The effect of the solvent was mimicked by applying the SMD model using dichloroethane as solvent.[16] The full intrinsic reaction coordinate (IRC) was computed at the same level of theory to identify the transition states.[17] Frequency calculations of reactants and transition states were performed at the same level of theory. The optimized geometries were confirmed to be minima (no imaginary frequencies) or transition structures (one imaginary frequency) by frequency calculations. 3D structures of optimized stationary points were represented using the CYLview 1.0 program.[18]

Energies and cartesian coordinates of stationary points

1a

E(electronic) = -395.828891294
Zero-point correction= 0.104399 (Hartree/Particle)
Thermal correction to Energy= 0.111346
Thermal correction to Enthalpy= 0.112290
Thermal correction to Gibbs Free Energy= 0.072225
Sum of electronic and zero-point Energies= -395.724492
Sum of electronic and thermal Energies= -395.717546
Sum of electronic and thermal Enthalpies= -395.716601
Sum of electronic and thermal Free Energies= -395.756666

6	0.151365000	-0.379002000	0.000013000
6	-0.122218000	0.985839000	-0.000040000
6	-0.885093000	-1.307069000	0.000086000
6	-1.441965000	1.414667000	-0.000007000
6	-2.198228000	-0.864851000	0.000116000
6	-2.483404000	0.495881000	0.000073000
1	0.684623000	1.709601000	-0.000106000
1	-0.647574000	-2.363189000	0.000123000
1	-1.653860000	2.476405000	-0.000043000
1	-3.003353000	-1.588701000	0.000177000
1	-3.510153000	0.837493000	0.000101000
7	1.468740000	-0.913032000	0.000015000
7	2.388586000	-0.099647000	0.000029000

7 3.286612000 0.563480000 -0.000285000

1e

E(electronic) = -732.921722413
Zero-point correction= 0.109461 (Hartree/Particle)
Thermal correction to Energy= 0.119883
Thermal correction to Enthalpy= 0.120827
Thermal correction to Gibbs Free Energy= 0.070709
Sum of electronic and zero-point Energies= -732.812261
Sum of electronic and thermal Energies= -732.801839
Sum of electronic and thermal Enthalpies= -732.800895
Sum of electronic and thermal Free Energies= -732.851013

6	-1.722941000	0.455176000	-0.003073000
6	-1.225276000	-0.844704000	-0.011997000
6	-0.854028000	1.545430000	-0.004245000
6	0.145565000	-1.050611000	-0.022003000
6	0.509561000	1.333264000	-0.014819000
6	1.008862000	0.034087000	-0.025673000
1	-1.893394000	-1.697548000	-0.012820000
1	-1.263031000	2.546949000	0.001585000
1	0.533937000	-2.060248000	-0.029884000
1	1.187426000	2.177789000	-0.016759000
7	-3.101949000	0.770317000	0.005937000
7	-3.884721000	-0.180171000	0.007458000
7	-4.672832000	-0.968181000	0.010432000
6	2.491500000	-0.167451000	-0.000232000
9	3.014647000	0.077341000	1.215402000
9	2.849490000	-1.415293000	-0.323746000
9	3.128320000	0.654412000	-0.849062000

1g

E(electronic) = -510.354558154
Zero-point correction= 0.137570 (Hartree/Particle)
Thermal correction to Energy= 0.146923
Thermal correction to Enthalpy= 0.147867
Thermal correction to Gibbs Free Energy= 0.102396
Sum of electronic and zero-point Energies= -510.216988
Sum of electronic and thermal Energies= -510.207635
Sum of electronic and thermal Enthalpies= -510.206691
Sum of electronic and thermal Free Energies= -510.252162

6	1.084927000	0.422594000	-0.000012000
6	0.096205000	1.393573000	-0.000009000
6	0.731561000	-0.925976000	-0.000008000
6	-1.245484000	1.034181000	-0.000001000
6	-0.601394000	-1.286988000	0.000000000
6	-1.600153000	-0.312320000	0.000004000
1	0.380577000	2.438116000	-0.000013000
1	1.493429000	-1.696723000	-0.000011000
1	-1.997988000	1.809684000	0.000001000
1	-0.890169000	-2.330270000	0.000004000
7	2.432849000	0.883028000	-0.000021000
7	3.305284000	0.021974000	-0.000021000
7	4.165495000	-0.692254000	-0.000003000
8	-2.876716000	-0.765536000	0.000009000
6	-3.916573000	0.196310000	0.000045000
1	-3.872105000	0.825444000	0.892620000
1	-3.872154000	0.825459000	-0.892523000

1	-4.847794000	-0.364913000	0.000065000

1h

E(electronic) = -1292.63106224
Zero-point correction= 0.112927 (Hartree/Particle)
Thermal correction to Energy= 0.125223
Thermal correction to Enthalpy= 0.126167
Thermal correction to Gibbs Free Energy= 0.073273
Sum of electronic and zero-point Energies= -1292.518136
Sum of electronic and thermal Energies= -1292.505840
Sum of electronic and thermal Enthalpies= -1292.504895
Sum of electronic and thermal Free Energies= -1292.557789

6	-2.475501000	0.464433000	-0.000047000
6	-1.939250000	-0.821355000	-0.000095000
6	-1.637388000	1.576686000	-0.000030000
6	-0.565193000	-0.990469000	-0.000142000
6	-0.266286000	1.408541000	0.000019000
6	0.258202000	0.123820000	-0.000014000
1	-2.578215000	-1.695867000	-0.000173000
1	-2.070969000	2.567659000	0.000006000
1	-0.153047000	-1.990030000	-0.000192000
1	0.378882000	2.275944000	0.000051000
7	-3.860392000	0.740690000	-0.000064000
7	-4.618476000	-0.231128000	0.000055000
7	-5.386973000	-1.037661000	0.000163000
16	2.039861000	-0.097573000	0.000016000
9	3.615169000	-0.292284000	0.000063000
9	2.250544000	1.007989000	1.123236000
9	1.975086000	-1.221232000	-1.123332000
9	1.975066000	-1.221958000	1.122664000
9	2.250685000	1.008621000	-1.122539000

1a- open-shell singlet nitrene ($^1A_2$)

E(electronic) = -286.247521675
Zero-point correction= 0.092505 (Hartree/Particle)
Thermal correction to Energy= 0.097930
Thermal correction to Enthalpy= 0.098874
Thermal correction to Gibbs Free Energy= 0.064104
Sum of electronic and zero-point Energies= -286.155017
Sum of electronic and thermal Energies= -286.149592
Sum of electronic and thermal Enthalpies= -286.148648
Sum of electronic and thermal Free Energies= -286.183418

6	0.000000000	0.000000000	1.080840000
6	0.000000000	1.231587000	0.326319000
6	0.000000000	-1.231587000	0.326319000
6	0.000000000	1.227712000	-1.045284000
6	0.000000000	-1.227712000	-1.045284000
6	0.000000000	0.000000000	-1.717356000
1	0.000000000	2.154735000	0.893627000
1	0.000000000	-2.154735000	0.893627000
1	0.000000000	2.149198000	-1.611698000
1	0.000000000	-2.149198000	-1.611698000
1	0.000000000	0.000000000	-2.801684000
7	0.000000000	0.000000000	2.383500000

1e- open-shell singlet nitrene ($^1A_2$)

E(electronic) = -623.331710809

Zero-point correction= 0.096791 (Hartree/Particle)
Thermal correction to Energy= 0.105197
Thermal correction to Enthalpy= 0.106141
Thermal correction to Gibbs Free Energy= 0.061964
Sum of electronic and zero-point Energies= -623.234919
Sum of electronic and thermal Energies= -623.226514
Sum of electronic and thermal Enthalpies= -623.225570
Sum of electronic and thermal Free Energies= -623.269746

6	2.532416000	-0.007229000	0.000057000
6	1.802089000	1.230172000	0.000407000
6	1.770586000	-1.228383000	-0.000285000
6	0.425865000	1.249783000	-0.000404000
6	0.398632000	-1.208287000	0.000331000
6	-0.249195000	0.030986000	-0.000065000
1	2.378006000	2.147278000	0.000828000
1	2.322711000	-2.159874000	-0.000646000
1	-0.124781000	2.179665000	-0.000738000
1	-0.182876000	-2.121333000	0.000547000
7	3.842003000	-0.020592000	-0.000112000
6	-1.763201000	0.008608000	-0.000017000
9	-2.228765000	-0.632805000	1.076399000
9	-2.296834000	1.228019000	-0.001083000
9	-2.228872000	-0.634713000	-1.075244000

1g- open-shell singlet nitrene ($^1A_2$)
E(electronic) = -400.791827175
Zero-point correction= 0.126158 (Hartree/Particle)
Thermal correction to Energy= 0.133955
Thermal correction to Enthalpy= 0.134899
Thermal correction to Gibbs Free Energy= 0.093699
Sum of electronic and zero-point Energies= -400.665669
Sum of electronic and thermal Energies= -400.657872
Sum of electronic and thermal Enthalpies= -400.656928
Sum of electronic and thermal Free Energies= -400.698128

6	-1.986596000	-0.160958000	-0.000019000
6	-1.017183000	-1.250935000	-0.000011000
6	-1.411068000	1.182953000	-0.000015000
6	0.327139000	-1.049655000	0.000001000
6	-0.074975000	1.399630000	-0.000004000
6	0.808476000	0.284923000	0.000005000
1	-1.422791000	-2.256279000	-0.000015000
1	-2.110443000	2.011070000	-0.000021000
1	1.018220000	-1.879629000	0.000005000
1	0.361829000	2.389828000	-0.000001000
7	-3.252834000	-0.373248000	-0.000029000
8	2.080660000	0.585748000	0.000017000
6	3.071308000	-0.451599000	0.000038000
1	2.974151000	-1.063708000	0.896008000
1	2.974189000	-1.063708000	-0.895936000
1	4.026790000	0.063027000	0.000059000

1h- open-shell singlet nitrene ($^1A_2$)
E(electronic) = -1183.03860526
Zero-point correction= 0.100224 (Hartree/Particle)
Thermal correction to Energy= 0.110492
Thermal correction to Enthalpy= 0.111437
Thermal correction to Gibbs Free Energy= 0.063557

Sum of electronic and zero-point Energies= -1182.938381
Sum of electronic and thermal Energies= -1182.928113
Sum of electronic and thermal Enthalpies= -1182.927169
Sum of electronic and thermal Free Energies= -1182.975048

6	3.182899000	-0.000072000	-0.000052000
6	2.438237000	1.227020000	-0.000178000
6	2.438217000	-1.227032000	0.000097000
6	1.062708000	1.231104000	-0.000207000
6	1.062736000	-1.230849000	0.000172000
6	0.410130000	0.000133000	-0.000006000
1	2.998626000	2.153549000	-0.000284000
1	2.998417000	-2.153686000	0.000183000
1	0.506775000	2.157599000	-0.000336000
1	0.506832000	-2.157369000	0.000318000
7	4.494573000	-0.000019000	-0.000073000
16	-1.411085000	0.000024000	0.000023000
9	-2.987175000	-0.000168000	0.000049000
9	-1.460430000	-1.117862000	1.121105000
9	-1.460697000	1.117824000	-1.121130000
9	-1.460662000	1.118055000	1.120948000
9	-1.460464000	-1.118091000	-1.120827000

1a- closed-shell singlet nitrene ($^1A_1$)
E(electronic) = -286.262206452
Zero-point correction= 0.092323 (Hartree/Particle)
Thermal correction to Energy= 0.097547
Thermal correction to Enthalpy= 0.098491
Thermal correction to Gibbs Free Energy= 0.063558
Sum of electronic and zero-point Energies= -286.169883
Sum of electronic and thermal Energies= -286.164659
Sum of electronic and thermal Enthalpies= -286.163715
Sum of electronic and thermal Free Energies= -286.198649

6	1.057245000	-0.000074000	-0.000024000
6	0.336448000	-1.223666000	0.000006000
6	0.336465000	1.223601000	-0.000022000
6	-1.042494000	-1.211584000	0.000041000
6	-1.042451000	1.211658000	0.000009000
6	-1.731050000	0.000012000	0.000041000
1	0.894455000	-2.151006000	0.000008000
1	0.894785000	2.150761000	-0.000051000
1	-1.590968000	-2.144673000	0.000070000
1	-1.590964000	2.144691000	0.000005000
7	2.388787000	0.000049000	-0.000057000
1	-2.813803000	0.000194000	0.000067000

1e- closed-shell singlet nitrene ($^1A_1$)
E(electronic) = -623.351534379
Zero-point correction= 0.097175 (Hartree/Particle)
Thermal correction to Energy= 0.105934
Thermal correction to Enthalpy= 0.106878
Thermal correction to Gibbs Free Energy= 0.061029
Sum of electronic and zero-point Energies= -623.254359
Sum of electronic and thermal Energies= -623.245600
Sum of electronic and thermal Enthalpies= -623.244656
Sum of electronic and thermal Free Energies= -623.290505

6	2.514511000	-0.006720000	0.000017000
6	1.815700000	1.223292000	-0.000026000

6	1.785619000	-1.222951000	0.000023000
6	0.434891000	1.232666000	-0.000044000
6	0.409491000	-1.195418000	-0.000020000
6	-0.257335000	0.028581000	-0.000052000
1	2.380989000	2.145383000	-0.000028000
1	2.329056000	-2.158048000	0.000042000
1	-0.100323000	2.172231000	-0.000073000
1	-0.154539000	-2.120047000	-0.000026000
7	3.850365000	-0.021955000	0.000042000
6	-1.760155000	0.008300000	-0.000011000
9	-2.245768000	-0.631448000	1.074422000
9	-2.293321000	1.231997000	-0.000896000
9	-2.245808000	-0.633030000	-1.073474000

1g- closed-shell singlet nitrene ($^1A_1$)

E(electronic) = -400.796131693
Zero-point correction= 0.125676 (Hartree/Particle)
Thermal correction to Energy= 0.133318
Thermal correction to Enthalpy= 0.134262
Thermal correction to Gibbs Free Energy= 0.093501
Sum of electronic and zero-point Energies= -400.670455
Sum of electronic and thermal Energies= -400.662814
Sum of electronic and thermal Enthalpies= -400.661869
Sum of electronic and thermal Free Energies= -400.702631

6	-1.966090000	-0.157179000	-0.000022000
6	-1.032410000	-1.242699000	-0.000011000
6	-1.421025000	1.172796000	-0.000017000
6	0.322570000	-1.032761000	0.000006000
6	-0.074310000	1.384945000	-0.000004000
6	0.810965000	0.286223000	0.000008000
1	-1.431192000	-2.249509000	-0.000017000
1	-2.115112000	2.003912000	-0.000022000
1	1.001502000	-1.872956000	0.000013000
1	0.346517000	2.382164000	0.000001000
7	-3.259365000	-0.371134000	-0.000035000
8	2.102004000	0.592391000	0.000022000
6	3.066473000	-0.459080000	0.000036000
1	2.962526000	-1.074544000	0.894549000
1	2.962555000	-1.074541000	-0.894483000
1	4.035691000	0.030815000	0.000052000

1h- closed-shell singlet nitrene ($^1A_1$)

E(electronic) = -1183.05987280
Zero-point correction= 0.100776 (Hartree/Particle)
Thermal correction to Energy= 0.111335
Thermal correction to Enthalpy= 0.112279
Thermal correction to Gibbs Free Energy= 0.064135
Sum of electronic and zero-point Energies= -1182.959096
Sum of electronic and thermal Energies= -1182.948538
Sum of electronic and thermal Enthalpies= -1182.947594
Sum of electronic and thermal Free Energies= -1182.995738

6	3.162921000	0.000000000	-0.000052000
6	2.449688000	1.221584000	-0.000172000
6	2.449691000	-1.221585000	0.000092000
6	1.070619000	1.216517000	-0.000157000
6	1.070622000	-1.216525000	0.000124000
6	0.400361000	-0.000005000	-0.000006000

1	3.000546000	2.152187000	-0.000292000
1	3.000554000	-2.152186000	0.000194000
1	0.530071000	2.152730000	-0.000260000
1	0.530078000	-2.152740000	0.000245000
7	4.499921000	0.000002000	-0.000075000
16	-1.403603000	-0.000004000	0.000023000
9	-2.985943000	0.000012000	0.000048000
9	-1.468130000	-1.121426000	1.122412000
9	-1.468145000	1.121429000	-1.122352000
9	-1.468110000	1.121749000	1.122082000
9	-1.468166000	-1.121747000	-1.122045000

1a- triplet nitrene ($^3A_2$)

E(electronic) = -286.290163389

Zero-point correction= 0.091928 (Hartree/Particle)

Thermal correction to Energy= 0.097173

Thermal correction to Enthalpy= 0.098117

Thermal correction to Gibbs Free Energy= 0.062780

Sum of electronic and zero-point Energies= -286.198236

Sum of electronic and thermal Energies= -286.192990

Sum of electronic and thermal Enthalpies= -286.192046

Sum of electronic and thermal Free Energies= -286.227383

6	0.000000000	0.000000000	1.057110000
6	0.000000000	1.227554000	0.337120000
6	0.000000000	-1.227554000	0.337120000
6	0.000000000	1.211708000	-1.038980000
6	0.000000000	-1.211708000	-1.038980000
6	0.000000000	0.000000000	-1.732909000
1	0.000000000	2.155924000	0.893474000
1	0.000000000	-2.155924000	0.893474000
1	0.000000000	2.145530000	-1.586345000
1	0.000000000	-2.145530000	-1.586345000
1	0.000000000	0.000000000	-2.815071000
7	0.000000000	0.000000000	2.382560000

1e- triplet nitrene ($^3A_2$)

E(electronic) = -623.380572857

Zero-point correction= 0.096791 (Hartree/Particle)

Thermal correction to Energy= 0.105582

Thermal correction to Enthalpy= 0.106527

Thermal correction to Gibbs Free Energy= 0.059259

Sum of electronic and zero-point Energies= -623.283782

Sum of electronic and thermal Energies= -623.274991

Sum of electronic and thermal Enthalpies= -623.274046

Sum of electronic and thermal Free Energies= -623.321314

6	2.516047000	-0.006731000	0.000394000
6	1.815166000	1.228332000	-0.000304000
6	1.785059000	-1.227751000	-0.000501000
6	0.437921000	1.234889000	-0.002019000
6	0.412938000	-1.197600000	-0.002005000
6	-0.257764000	0.028996000	-0.003058000
1	2.379924000	2.150900000	0.000390000
1	2.328115000	-2.163259000	-0.000171000
1	-0.098365000	2.173814000	-0.002910000
1	-0.151992000	-2.121634000	-0.002672000
7	3.842815000	-0.022720000	0.001737000
6	-1.758318000	0.008469000	-0.000232000

9 -2.244789000 -0.595831000 1.095426000
9 -2.292159000 1.231713000 -0.040311000
9 -2.247906000 -0.668371000 -1.050720000

1g- triplet nitrene ($^3A_2$)

E(electronic) = -400.820665283
Zero-point correction= 0.125341 (Hartree/Particle)
Thermal correction to Energy= 0.133002
Thermal correction to Enthalpy= 0.133946
Thermal correction to Gibbs Free Energy= 0.092136
Sum of electronic and zero-point Energies= -400.695325
Sum of electronic and thermal Energies= -400.687664
Sum of electronic and thermal Enthalpies= -400.686720
Sum of electronic and thermal Free Energies= -400.728530
6 -1.957826000 -0.156655000 -0.000020000
6 -1.040177000 -1.244938000 -0.000012000
6 -1.428167000 1.171294000 -0.000015000
6 0.317712000 -1.025737000 0.000001000
6 -0.077817000 1.376113000 -0.000002000
6 0.812694000 0.285612000 0.000006000
1 -1.433047000 -2.253483000 -0.000016000
1 -2.117641000 2.005632000 -0.000021000
1 0.991145000 -1.870777000 0.000007000
1 0.334973000 2.376984000 0.000002000
7 -3.260283000 -0.367100000 -0.000032000
8 2.116580000 0.597738000 0.000018000
6 3.065835000 -0.460419000 0.000038000
1 2.958702000 -1.078514000 0.893559000
1 2.958734000 -1.078521000 -0.893483000
1 4.042953000 0.014861000 0.000054000

1h- triplet nitrene ($^3A_2$)

E(electronic) = -1183.08913080
Zero-point correction= 0.100397 (Hartree/Particle)
Thermal correction to Energy= 0.110984
Thermal correction to Enthalpy= 0.111928
Thermal correction to Gibbs Free Energy= 0.062690
Sum of electronic and zero-point Energies= -1182.988734
Sum of electronic and thermal Energies= -1182.978147
Sum of electronic and thermal Enthalpies= -1182.977203
Sum of electronic and thermal Free Energies= -1183.026441
6 3.163406000 0.000063000 -0.000054000
6 2.448508000 1.226602000 -0.000204000
6 2.447998000 -1.227136000 0.000126000
6 1.072914000 1.219201000 -0.000166000
6 1.072912000 -1.219686000 0.000135000
6 0.399957000 0.000084000 -0.000009000
1 2.998748000 2.157698000 -0.000342000
1 2.998526000 -2.158088000 0.000246000
1 0.530855000 2.154410000 -0.000289000
1 0.530401000 -2.154636000 0.000275000
7 4.491663000 0.000308000 -0.000070000
16 -1.401454000 0.000044000 0.000020000
9 -2.985283000 0.000016000 0.000049000
9 -1.467882000 -1.121562000 1.122659000
9 -1.467856000 1.121724000 -1.122472000
9 -1.467820000 1.121942000 1.122294000

9 -1.467945000 -1.121787000 -1.122385000

1a- TS1

E(electronic) = -286.241657428

Zero-point correction= 0.091460 (Hartree/Particle)

Thermal correction to Energy= 0.096314

Thermal correction to Enthalpy= 0.097258

Thermal correction to Gibbs Free Energy= 0.063009

Sum of electronic and zero-point Energies= -286.150198

Sum of electronic and thermal Energies= -286.145343

Sum of electronic and thermal Enthalpies= -286.144399

Sum of electronic and thermal Free Energies= -286.178648

6	-0.496904000	-1.123893000	-0.270828000
6	-1.065455000	0.194792000	-0.140142000
6	0.852232000	-1.309691000	0.058856000
6	-0.176892000	1.343576000	-0.125163000
6	1.661912000	-0.203698000	0.147940000
1	1.267226000	-2.305814000	0.141321000
6	1.155075000	1.120135000	-0.019109000
1	-0.592708000	2.340685000	-0.197820000
1	2.723849000	-0.330710000	0.319682000
1	1.850849000	1.949326000	-0.004788000
7	-2.238483000	0.023480000	0.332808000
1	-1.159635000	-1.945166000	-0.497374000

1a- II

E(electronic) = -286.275589461

Zero-point correction= 0.092835 (Hartree/Particle)

Thermal correction to Energy= 0.097945

Thermal correction to Enthalpy= 0.098889

Thermal correction to Gibbs Free Energy= 0.064345

Sum of electronic and zero-point Energies= -286.182755

Sum of electronic and thermal Energies= -286.177645

Sum of electronic and thermal Enthalpies= -286.176700

Sum of electronic and thermal Free Energies= -286.211244

6	0.693165000	-0.866496000	0.110847000
6	1.156208000	0.507223000	0.411769000
6	-0.632454000	-1.391226000	0.214311000
6	0.104403000	1.451501000	0.067691000
1	1.876604000	0.722913000	1.188647000
6	-1.525170000	-0.404822000	-0.035125000
1	-0.880690000	-2.416571000	0.443856000
6	-1.137379000	0.981734000	-0.219825000
1	0.271318000	2.519557000	0.154443000
1	-2.581585000	-0.642357000	-0.071314000
1	-1.925469000	1.681927000	-0.466447000
7	1.612456000	-0.504708000	-0.649598000

1a- TS2

E(electronic) = -286.267147858

Zero-point correction= 0.091028 (Hartree/Particle)

Thermal correction to Energy= 0.095932

Thermal correction to Enthalpy= 0.096876

Thermal correction to Gibbs Free Energy= 0.062701

Sum of electronic and zero-point Energies= -286.176120

Sum of electronic and thermal Energies= -286.171216

Sum of electronic and thermal Enthalpies= -286.170271

Sum of electronic and thermal Free Energies= -286.204447

6	-0.502941000	-1.071637000	-0.025094000
6	-1.314118000	0.458503000	-0.322910000
6	0.811683000	-1.291587000	-0.312836000
6	-0.313828000	1.391992000	-0.140446000
1	-2.123477000	0.580981000	-1.029800000
6	1.560589000	-0.176053000	0.081987000
1	1.199871000	-2.173202000	-0.797461000
6	0.994772000	1.084782000	0.270252000
1	-0.501711000	2.395266000	-0.510359000
1	2.637913000	-0.276379000	0.149762000
1	1.663170000	1.905314000	0.495983000
7	-1.470387000	-0.686855000	0.626593000

1a- III

E(electronic) = -286.237204172

Zero-point correction= 0.089787 (Hartree/Particle)
Thermal correction to Energy= 0.095233
Thermal correction to Enthalpy= 0.096177
Thermal correction to Gibbs Free Energy= 0.060932
Sum of electronic and zero-point Energies= -286.147417
Sum of electronic and thermal Energies= -286.141972
Sum of electronic and thermal Enthalpies= -286.141027
Sum of electronic and thermal Free Energies= -286.176272

6	0.000000000	-1.332127000	0.000000000
6	1.318824000	-1.207865000	0.000000000
6	-1.674796000	0.295753000	0.000000000
6	1.548912000	0.265480000	0.000000000
6	-0.825025000	1.314828000	0.000000000
6	0.677904000	1.289781000	0.000000000
1	2.085832000	-1.957998000	0.000000000
1	-2.749251000	0.378057000	0.000000000
1	2.602510000	0.526946000	0.000000000
1	-1.269672000	2.302428000	0.000000000
1	1.119205000	2.277701000	0.000000000
7	-1.151934000	-1.040320000	0.000000000

1a- IV

E(electronic) = -593.800567479

Zero-point correction= 0.202015 (Hartree/Particle)
Thermal correction to Energy= 0.213100
Thermal correction to Enthalpy= 0.214045
Thermal correction to Gibbs Free Energy= 0.163448
Sum of electronic and zero-point Energies= -593.598552
Sum of electronic and thermal Energies= -593.587467
Sum of electronic and thermal Enthalpies= -593.586523
Sum of electronic and thermal Free Energies= -593.637119

6	-0.051025000	-0.988640000	0.000000000
6	1.175442000	-0.443583000	0.000000000
6	0.391641000	-3.450565000	0.000000000
6	2.466912000	-1.143123000	0.000000000
6	1.721436000	-3.542567000	0.000000000
6	2.714345000	-2.454253000	0.000000000
1	1.217391000	0.635251000	0.000000000
1	-0.196793000	-4.359133000	0.000000000
1	3.324672000	-0.479460000	0.000000000
1	2.113157000	-4.551315000	0.000000000

1	3.750776000	-2.770218000	0.000000000
7	-0.423722000	-2.320524000	0.000000000
8	-1.202067000	-0.264707000	0.000000000
6	-1.139684000	1.119128000	0.000000000
6	-1.139450000	1.792197000	1.209691000
6	-1.139450000	1.792197000	-1.209691000
6	-1.139450000	3.180973000	1.203614000
6	-1.139450000	3.180973000	-1.203614000
6	-1.138420000	3.875377000	0.000000000
1	-1.139979000	1.228940000	2.134304000
1	-1.139979000	1.228940000	-2.134304000
1	-1.142469000	3.719989000	2.142326000
1	-1.142469000	3.719989000	-2.142326000
1	-1.140186000	4.957788000	0.000000000
1	-1.418627000	-2.478138000	0.000000000

1a- 3

E(electronic) = -593.820703158

Zero-point correction= 0.203281 (Hartree/Particle)
Thermal correction to Energy= 0.214208
Thermal correction to Enthalpy= 0.215152
Thermal correction to Gibbs Free Energy= 0.165223
Sum of electronic and zero-point Energies= -593.617422
Sum of electronic and thermal Energies= -593.606495
Sum of electronic and thermal Enthalpies= -593.605551
Sum of electronic and thermal Free Energies= -593.655481

6	0.920851000	-0.375037000	-0.330757000
6	1.068001000	0.417186000	0.941931000
6	3.095082000	-1.185136000	-0.276493000
6	2.049671000	1.500099000	0.609445000
6	3.821051000	-0.141557000	0.191116000
6	3.328779000	1.206767000	0.328379000
1	0.120021000	0.821177000	1.283036000
1	3.586481000	-2.142243000	-0.411101000
1	1.703097000	2.526514000	0.604811000
1	4.875934000	-0.319362000	0.364918000
1	4.038773000	2.015572000	0.191316000
7	1.820256000	-1.139329000	-0.797958000
8	-0.184246000	-0.196564000	-1.098900000
6	-1.426080000	-0.115592000	-0.501890000
6	-1.780391000	-0.960791000	0.539829000
6	-2.326953000	0.795777000	-1.029397000
6	-3.060283000	-0.874252000	1.070529000
6	-3.606521000	0.864433000	-0.496397000
6	-3.975641000	0.035021000	0.555625000
1	-1.064670000	-1.678372000	0.923551000
1	-2.018728000	1.434555000	-1.846982000
1	-3.342795000	-1.528571000	1.885382000
1	-4.315888000	1.572925000	-0.904818000
1	-4.973674000	0.094186000	0.969719000
1	1.478225000	-0.250078000	1.705555000

1g- TS1

E(electronic) = -400.779597779

Zero-point correction= 0.124925 (Hartree/Particle)
Thermal correction to Energy= 0.132176
Thermal correction to Enthalpy= 0.133120
Thermal correction to Gibbs Free Energy= 0.093227

Sum of electronic and zero-point Energies= -400.654673
Sum of electronic and thermal Energies= -400.647422
Sum of electronic and thermal Enthalpies= -400.646478
Sum of electronic and thermal Free Energies= -400.686371
Frequency -306.4432

1	-1.550157000	-2.093032000	-0.717623000
6	-1.108560000	-1.160040000	-0.407690000
6	-1.978864000	-0.051080000	-0.092099000
6	0.263986000	-1.026911000	-0.153894000
6	-1.437933000	1.306854000	-0.123090000
6	0.756851000	0.258196000	-0.042839000
1	0.917662000	-1.886835000	-0.151075000
6	-0.100915000	1.426820000	-0.115120000
1	-2.094425000	2.167845000	-0.119219000
1	0.388864000	2.392389000	-0.102953000
7	-2.855712000	-0.627616000	0.610774000
8	2.038402000	0.563657000	0.105012000
6	2.989835000	-0.496484000	0.169857000
1	2.770028000	-1.153761000	1.012384000
1	2.989103000	-1.066536000	-0.760239000
1	3.955282000	-0.020144000	0.312469000

1g- II
E(electronic) = -400.803643375
Zero-point correction= 0.126076 (Hartree/Particle)
Thermal correction to Energy= 0.133554
Thermal correction to Enthalpy= 0.134498
Thermal correction to Gibbs Free Energy= 0.094227
Sum of electronic and zero-point Energies= -400.677568
Sum of electronic and thermal Energies= -400.670089
Sum of electronic and thermal Enthalpies= -400.669145
Sum of electronic and thermal Free Energies= -400.709416

1	-1.639251000	-1.838837000	-1.188261000
6	-1.251368000	-1.173394000	-0.428943000
6	-1.966199000	0.032288000	-0.014455000
6	0.189533000	-1.000922000	-0.252610000
6	-1.534534000	1.395256000	-0.129028000
6	0.656028000	0.258513000	-0.036640000
1	0.867915000	-1.828636000	-0.408895000
6	-0.189274000	1.450980000	-0.087125000
1	-2.183021000	2.254155000	-0.217786000
1	0.324967000	2.404427000	-0.092912000
7	-2.188815000	-0.917860000	0.767194000
8	1.957209000	0.584169000	0.144687000
6	2.900831000	-0.471202000	0.120090000
1	2.672539000	-1.215056000	0.887871000
1	2.922523000	-0.954202000	-0.859945000
1	3.868258000	-0.019303000	0.324349000

1g- TS2
E(electronic) = -400.788921837
Zero-point correction= 0.123957 (Hartree/Particle)
Thermal correction to Energy= 0.131297

Thermal correction to Enthalpy= 0.132241
Thermal correction to Gibbs Free Energy= 0.092133
Sum of electronic and zero-point Energies= -400.664965
Sum of electronic and thermal Energies= -400.657625
Sum of electronic and thermal Enthalpies= -400.656681
Sum of electronic and thermal Free Energies= -400.696789
Frequency -503.5889

6	1.875242000	0.646105000	0.104724000
6	1.706011000	-1.036896000	-0.289291000
6	0.974820000	1.584025000	-0.296265000
6	0.341147000	-1.248309000	-0.319911000
1	2.395485000	-1.561357000	-0.937291000
6	-0.331286000	1.100975000	-0.126986000
1	1.226035000	2.539617000	-0.728698000
6	-0.614654000	-0.262974000	-0.008075000
1	-0.033756000	-2.149496000	-0.793695000
1	-1.144359000	1.811507000	-0.189479000
7	2.334522000	-0.252726000	0.812204000
8	-1.883479000	-0.740729000	0.144831000
6	-2.950515000	0.187203000	0.155797000
1	-2.848630000	0.899762000	0.978656000
1	-3.016319000	0.731159000	-0.790667000
1	-3.856870000	-0.397054000	0.297144000

1g- III

E(electronic) =-400.761315209
Zero-point correction= 0.123100 (Hartree/Particle)
Thermal correction to Energy= 0.130863
Thermal correction to Enthalpy= 0.131807
Thermal correction to Gibbs Free Energy= 0.090918
Sum of electronic and zero-point Energies= -400.638215
Sum of electronic and thermal Energies= -400.630452
Sum of electronic and thermal Enthalpies= -400.629508
Sum of electronic and thermal Free Energies= -400.670397

6	0.224722000	-2.081042000	0.000000000
6	1.406934000	-1.521124000	0.000000000
6	-1.868949000	-1.122571000	0.000000000
6	1.167312000	-0.024522000	0.000000000
6	-1.422947000	0.130530000	0.000000000
6	0.000000000	0.635656000	0.000000000
1	2.383567000	-1.966957000	0.000000000
1	-2.917191000	-1.378328000	0.000000000
1	2.086734000	0.543477000	0.000000000
1	-2.161959000	0.920694000	0.000000000
7	-0.969707000	-2.212095000	0.000000000
8	-0.079752000	1.998460000	0.000000000
6	1.121317000	2.739564000	0.000000000
1	1.716413000	2.525730000	0.892233000
1	1.716413000	2.525730000	-0.892233000
1	0.831654000	3.787707000	0.000000000

3e

E(electronic) =--708.348323578

6	0.151320000	-0.896696000	-0.310946000
6	0.495484000	-0.056767000	0.891731000

6	2.107946000	-2.129817000	-0.192577000
6	1.672753000	0.783375000	0.492963000
6	3.042129000	-1.236900000	0.197210000
6	2.858176000	0.199704000	0.229248000
1	-0.338323000	0.567868000	1.196920000
1	2.381686000	-3.178203000	-0.228990000
1	1.540369000	1.853949000	0.429558000
1	4.040644000	-1.603403000	0.404207000
7	0.863840000	-1.862294000	-0.724772000
8	-0.911571000	-0.558924000	-1.082283000
6	-2.085135000	-0.136386000	-0.491150000
6	-2.595033000	-0.772786000	0.631250000
6	-2.767079000	0.902717000	-1.103841000
6	-3.806373000	-0.342900000	1.155668000
6	-3.982066000	1.315655000	-0.575259000
6	-4.502686000	0.698946000	0.555892000
1	-2.050348000	-1.593722000	1.082838000
1	-2.342700000	1.371562000	-1.982290000
1	-4.209277000	-0.832461000	2.033134000
1	-4.520905000	2.126002000	-1.049468000
1	-5.448979000	1.026371000	0.966256000
1	0.749714000	-0.734407000	1.711602000
8	4.013756000	0.866737000	-0.023757000
6	3.975194000	2.278988000	0.047858000
1	3.316062000	2.694108000	-0.718759000
1	3.637655000	2.610524000	1.033748000
1	4.992270000	2.622561000	-0.125314000

1h- TS1

E(electronic) = -623.336409319

Zero-point correction= 0.095328 (Hartree/Particle)

Thermal correction to Energy= 0.103814

Thermal correction to Enthalpy= 0.104759

Thermal correction to Gibbs Free Energy= 0.060767

Sum of electronic and zero-point Energies= -623.241081

Sum of electronic and thermal Energies= -623.232595

Sum of electronic and thermal Enthalpies= -623.231651

Sum of electronic and thermal Free Energies= -623.275643

Frequency -783.2702

1	2.488394000	-1.957717000	0.686528000
6	1.891045000	-1.117064000	0.378158000
6	2.508953000	0.165864000	0.073596000
6	0.502006000	-1.193649000	0.172105000
6	1.755227000	1.399952000	0.199503000
6	-0.207268000	-0.026578000	0.058757000
1	-0.004536000	-2.148225000	0.228710000
6	0.413617000	1.265870000	0.170570000
1	2.253743000	2.355720000	0.277193000
1	-0.220941000	2.143275000	0.212898000
7	3.425130000	-0.288687000	-0.654642000
6	-1.700944000	-0.047307000	-0.062266000
9	-2.117739000	0.633437000	-1.141864000
9	-2.197536000	-1.281521000	-0.161814000
9	-2.292323000	0.530888000	0.996414000

1h- II

E(electronic) = -623.368179582

Zero-point correction= 0.097871 (Hartree/Particle)

Thermal correction to Energy= 0.106390

Thermal correction to Enthalpy= 0.107334

Thermal correction to Gibbs Free Energy= 0.063445

Sum of electronic and zero-point Energies= -623.270309

Sum of electronic and thermal Energies= -623.261790

Sum of electronic and thermal Enthalpies= -623.260846

Sum of electronic and thermal Free Energies= -623.304735

1	-2.553770000	-1.678812000	-1.116233000
6	-2.015498000	-1.070754000	-0.403537000
6	-2.461100000	0.284869000	0.003322000
6	-0.571964000	-1.156167000	-0.287234000
6	-1.778553000	1.526203000	-0.185327000
6	0.130370000	-0.014555000	-0.070104000
1	-0.053636000	-2.085649000	-0.490472000
6	-0.445127000	1.318655000	-0.136632000
1	-2.253712000	2.484067000	-0.332977000
1	0.231897000	2.163916000	-0.180099000
7	-2.822365000	-0.590906000	0.809868000
6	1.624272000	-0.062338000	0.053371000
9	2.230024000	0.608881000	-0.941623000
9	2.045367000	0.506503000	1.195639000
9	2.112540000	-1.304569000	0.035711000

1h- TS2

E(electronic) = -623.360543300

Zero-point correction= 0.095980 (Hartree/Particle)

Thermal correction to Energy= 0.104323

Thermal correction to Enthalpy= 0.105267

Thermal correction to Gibbs Free Energy= 0.061216

Sum of electronic and zero-point Energies= -623.264564

Sum of electronic and thermal Energies= -623.256220

Sum of electronic and thermal Enthalpies= -623.255276

Sum of electronic and thermal Free Energies= -623.299327

Frequency -419.8234

6	2.487516000	0.372772000	0.109977000
6	1.946827000	-1.221076000	-0.327625000
6	1.836465000	1.517496000	-0.262138000
6	0.574251000	-1.105086000	-0.370343000
1	2.506736000	-1.884726000	-0.970989000
6	0.464830000	1.341222000	-0.105223000
1	2.315567000	2.407970000	-0.635862000
6	-0.119109000	0.072755000	-0.038371000
1	0.020647000	-1.899622000	-0.861235000
1	-0.176601000	2.213269000	-0.129553000
7	2.732726000	-0.612128000	0.796041000
6	-1.610551000	-0.033414000	0.060630000
9	-2.150458000	-0.677501000	-0.990300000
9	-1.995792000	-0.725449000	1.148743000
9	-2.217840000	1.156283000	0.133103000

1h- III

E(electronic) =-623.371340197

Zero-point correction= 0.097529 (Hartree/Particle)

Thermal correction to Energy= 0.106198
Thermal correction to Enthalpy= 0.107142
Thermal correction to Gibbs Free Energy= 0.063061
Sum of electronic and zero-point Energies= -623.273811
Sum of electronic and thermal Energies= -623.265143
Sum of electronic and thermal Enthalpies= -623.264198
Sum of electronic and thermal Free Energies= -623.308280

6	2.502491000	-0.530237000	0.183312000
6	1.872174000	-1.570908000	-0.309732000
6	1.838528000	1.467628000	-0.168693000
6	0.438162000	-1.381442000	-0.074649000
6	0.580898000	1.114864000	-0.474822000
6	-0.098651000	-0.143884000	-0.141048000
1	2.303930000	-2.379959000	-0.876635000
1	2.267888000	2.422353000	-0.441486000
1	-0.192005000	-2.239624000	0.127961000
1	-0.025400000	1.864738000	-0.971465000
7	2.640511000	0.599753000	0.649260000
6	-1.580331000	0.014835000	0.056692000
9	-2.173148000	0.513392000	-1.042695000
9	-2.204323000	-1.129576000	0.345232000
9	-1.862264000	0.872749000	1.051956000

3e

E(electronic) =--930.914522393
Zero-point correction= 0.208318 (Hartree/Particle)
Thermal correction to Energy= 0.222746
Thermal correction to Enthalpy= 0.223690
Thermal correction to Gibbs Free Energy= 0.164623
Sum of electronic and zero-point Energies= -930.706204
Sum of electronic and thermal Energies= -930.691777
Sum of electronic and thermal Enthalpies= -930.690833
Sum of electronic and thermal Free Energies= -930.749900

6	0.482038000	0.985756000	-0.304112000
6	0.126642000	0.202164000	0.936903000
6	-1.428581000	2.301779000	-0.212263000
6	-1.082189000	-0.592844000	0.562503000
6	-2.396876000	1.458398000	0.212839000
6	-2.241884000	0.024790000	0.295278000
1	0.937405000	-0.438766000	1.266503000
1	-1.662564000	3.357244000	-0.290908000
1	-1.001725000	-1.670962000	0.511463000
1	-3.376364000	1.879929000	0.405539000
7	-0.202983000	1.962910000	-0.736075000
8	1.516077000	0.581893000	-1.074613000
6	2.674061000	0.103401000	-0.489508000
6	3.249238000	0.750087000	0.594123000
6	3.269473000	-1.002848000	-1.072824000
6	4.441046000	0.261074000	1.111468000
6	4.466034000	-1.475054000	-0.551594000
6	5.052066000	-0.848943000	0.541662000
1	2.770347000	1.623328000	1.021584000
1	2.794876000	-1.476781000	-1.922335000
1	4.896052000	0.757636000	1.958946000
1	4.939024000	-2.338371000	-1.001887000

1 5.983767000 -1.222229000 0.946155000
1 -0.120670000 0.919589000 1.724347000
6 -3.483587000 -0.781768000 0.048913000
9 -4.461081000 -0.450224000 0.910518000
9 -3.983414000 -0.573687000 -1.180567000
9 -3.292479000 -2.098543000 0.168902000

1e- TS1

E(electronic) = -1183.04398968
Zero-point correction= 0.098945 (Hartree/Particle)
Thermal correction to Energy= 0.109253
Thermal correction to Enthalpy= 0.110198
Thermal correction to Gibbs Free Energy= 0.062721
Sum of electronic and zero-point Energies= -1182.945044
Sum of electronic and thermal Energies= -1182.934736
Sum of electronic and thermal Enthalpies= -1182.933792
Sum of electronic and thermal Free Energies= -1182.981269
Frequency -793.9334
1 -3.090687000 -1.973437000 -0.685258000
6 -2.508995000 -1.119950000 -0.382411000
6 -3.146712000 0.149248000 -0.059139000
6 -1.116444000 -1.169621000 -0.208718000
6 -2.420764000 1.395979000 -0.196014000
6 -0.434754000 0.014278000 -0.101206000
1 -0.598524000 -2.116333000 -0.286885000
6 -1.074825000 1.295157000 -0.192381000
1 -2.937148000 2.342934000 -0.262170000
1 -0.471238000 2.191060000 -0.239827000
7 -4.034377000 -0.334806000 0.683585000
16 1.359534000 -0.021620000 0.035854000
9 2.937948000 -0.037576000 0.167060000
9 1.506895000 -1.161548000 -1.059521000
9 1.348270000 1.118655000 1.142878000
9 1.532677000 1.080021000 -1.096480000
9 1.318727000 -1.126797000 1.174353000

1e- II

E(electronic) = -1183.07566244
Zero-point correction= 0.101348 (Hartree/Particle)
Thermal correction to Energy= 0.111736
Thermal correction to Enthalpy= 0.112680
Thermal correction to Gibbs Free Energy= 0.065160
Sum of electronic and zero-point Energies= -1182.974315
Sum of electronic and thermal Energies= -1182.963926
Sum of electronic and thermal Enthalpies= -1182.962982
Sum of electronic and thermal Free Energies= -1183.010502
1 -3.143296000 -1.705032000 -1.110534000
6 -2.610490000 -1.084637000 -0.404557000
6 -3.085641000 0.255132000 0.021816000
6 -1.163472000 -1.133535000 -0.322819000
6 -2.439707000 1.512172000 -0.177173000
6 -0.495446000 0.027787000 -0.114707000
1 -0.633786000 -2.052280000 -0.542291000
6 -1.099232000 1.347026000 -0.158501000
1 -2.939968000 2.459564000 -0.308173000
1 -0.458780000 2.216901000 -0.204473000
7 -3.403000000 -0.636228000 0.828760000

16 1.303992000 -0.028314000 0.030053000
9 2.883437000 -0.055716000 0.174647000
9 1.495813000 1.132423000 -1.041260000
9 1.254891000 -1.194059000 1.106506000
9 1.290844000 1.051405000 1.198272000
9 1.463558000 -1.106964000 -1.124949000

1e- TS2

E(electronic) = -1183.06882983
Zero-point correction= 0.099477 (Hartree/Particle)
Thermal correction to Energy= 0.109721
Thermal correction to Enthalpy= 0.110666
Thermal correction to Gibbs Free Energy= 0.063163
Sum of electronic and zero-point Energies= -1182.969353
Sum of electronic and thermal Energies= -1182.959108
Sum of electronic and thermal Enthalpies= -1182.958164
Sum of electronic and thermal Free Energies= -1183.005667
Frequency -410.0164
6 3.096725000 0.374616000 0.132503000
6 2.562738000 -1.229318000 -0.319714000
6 2.455929000 1.515121000 -0.263649000
6 1.194129000 -1.111278000 -0.408929000
1 3.137065000 -1.897523000 -0.945431000
6 1.080520000 1.342074000 -0.140314000
1 2.942228000 2.408516000 -0.620655000
6 0.504461000 0.071431000 -0.093336000
1 0.664147000 -1.909752000 -0.914344000
1 0.448467000 2.220340000 -0.163119000
7 3.317804000 -0.614496000 0.818735000
16 -1.289561000 -0.022097000 0.034353000
9 -2.870941000 -0.111943000 0.173800000
9 -1.215482000 -1.295305000 0.984176000
9 -1.527369000 1.237532000 -0.909018000
9 -1.310197000 0.932688000 1.306484000
9 -1.426076000 -0.978798000 -1.230621000

1e- III

E(electronic) =-1183.07864176
Zero-point correction= 0.101015 (Hartree/Particle)
Thermal correction to Energy= 0.111563
Thermal correction to Enthalpy= 0.112507
Thermal correction to Gibbs Free Energy= 0.064793
Sum of electronic and zero-point Energies= -1182.977627
Sum of electronic and thermal Energies= -1182.967079
Sum of electronic and thermal Enthalpies= -1182.966135
Sum of electronic and thermal Free Energies= -1183.013849
6 3.098274000 0.528910000 -0.212138000
6 2.485372000 1.568026000 0.307051000
6 2.453050000 -1.463719000 0.165944000
6 1.046438000 1.380964000 0.119426000
6 1.205532000 -1.118147000 0.519740000
6 0.522039000 0.140691000 0.206042000
1 2.934299000 2.379828000 0.856236000
1 2.897326000 -2.412288000 0.436330000
1 0.416882000 2.242618000 -0.068739000
1 0.633345000 -1.868740000 1.049079000
7 3.219593000 -0.598117000 -0.686093000

16 -1.270705000 -0.011860000 -0.031912000
9 -2.836399000 -0.170314000 -0.238283000
9 -1.440648000 -0.834205000 1.320025000
9 -1.244840000 0.802049000 -1.397297000
9 -1.588853000 1.330710000 0.757168000
9 -1.106142000 -1.371039000 -0.841174000

3e

E(electronic) =-1490.62304565
Zero-point correction= 0.211776 (Hartree/Particle)
Thermal correction to Energy= 0.228126
Thermal correction to Enthalpy= 0.229070
Thermal correction to Gibbs Free Energy= 0.166284
Sum of electronic and zero-point Energies= -1490.411270
Sum of electronic and thermal Energies= -1490.394920
Sum of electronic and thermal Enthalpies= -1490.393975
Sum of electronic and thermal Free Energies= -1490.456762
6 1.292998000 1.109284000 -0.311154000
6 0.883428000 0.371501000 0.942546000
6 -0.501901000 2.574557000 -0.209776000
6 -0.384238000 -0.328174000 0.574034000
6 -1.538102000 1.823182000 0.231172000
6 -1.486845000 0.386111000 0.318198000
1 1.639028000 -0.333161000 1.272928000
1 -0.644822000 3.646813000 -0.278900000
1 -0.378777000 -1.408457000 0.510534000
1 -2.467427000 2.333416000 0.443619000
7 0.682170000 2.133299000 -0.745874000
8 2.284397000 0.616098000 -1.081803000
6 3.402777000 0.050634000 -0.493686000
6 4.041733000 0.673339000 0.567835000
6 3.892810000 -1.118344000 -1.051276000
6 5.190819000 0.095609000 1.090155000
6 5.048453000 -1.679439000 -0.525445000
6 5.696732000 -1.078445000 0.546496000
1 3.647569000 1.597726000 0.973584000
1 3.370875000 -1.570763000 -1.884555000
1 5.695445000 0.572798000 1.920426000
1 5.440210000 -2.592306000 -0.955472000
1 6.595830000 -1.521373000 0.954478000
1 0.698039000 1.113171000 1.723889000
16 -3.036560000 -0.505161000 0.028635000
9 -4.408254000 -1.264684000 -0.220603000
9 -2.762587000 -1.548625000 1.197526000
9 -3.445249000 0.460946000 -1.166495000
9 -3.821434000 0.426439000 1.053641000
9 -2.372848000 -1.506973000 -1.012705000

9. References

1. K. Tanagawa, Z. Zhao, N. Saito, N. Shibata, *Bull. Chem. Soc. Jpn*. **2021**, *94*, 1682–1684.
2. C. Wang, Y. Yu, S. Fan, X. Zhang, *Org. Lett*. **2013**, *15*, 5004–5007.
3. I. C. Barral, M. Mielczarek, D. A. Carrillo, V. Capurro, V. S. Cerrato, R. P. Tomas, E. Caci, M. G. Valverde, R. Quesada, *Chem. Commun*. **2020**, *56*, 3218–3221.
4. L. Zhong, P. R. Savoie, A. S. Filatov, J. T. Welch, *Angew. Chem. Int. Ed.* **2014**, *53*, 526–529.
5. L. Hu, X. Cao, L. Shi, F. Qi, Z. Guo, J. Lu, H. Gu, *Org. Lett.* **2011**, *13*, 5640–5643.
6. K. Maruno, K. Niina, O. Nagata, N. Shibata, *Org. Lett.* **2022**, *24*, 1722–1726.
7. P. Das, K. Niina, T. Hiromura, E. Tokunaga, N. Saito, N. Shibata, *Chem. Sci.* **2018**, *9*, 4931–4936.
8. K. Maruno, K. Hada, Y. Sumii, O. Nagata, N. Shibata, *Org. Lett.* **2022**, *24*, 3755–3759.
9. *Rowan Scientific* (https://www.rowansci.com).
10. D. M. Anstine, R. Zubatyuk, O. Isayev, *Chem Rxiv,* **2023**, doi:10.26434/chemrxiv-2023-296ch.
11. C. Bannwarth, S. Ehlert, S. Grimme, *J. Chem. Theory Comput.* **2019**, *15*, 1652–1671.
12. A. T. Maynard, M. Huang, W. G. Rice, D. G. Covel, *Proc. Natl. Acad. Sci. U.S.A.* **1998**, *95*, 11578–11583 (doi.org/10.1073/pnas.95.20.11578).
13. Gaussian 16, Revision B.01, M. J. Frisch, G. W. Trucks, H. B. Schlegel, G. E. Scuseria, M. A. Robb, J. R. Cheeseman, G. Scalmani, V. Barone, G. A. Petersson, H. Nakatsuji, X. Li, M. Caricato, A. V. Marenich, J. Bloino, B. G. Janesko, R. Gomperts, B. Mennucci, H. P. Hratchian, J. V. Ortiz, A. F. Izmaylov, J. L. Sonnenberg, D. Williams-Young, F. Ding, F. Lipparini, F. Egidi, J. Goings, B. Peng, A. Petrone, T. Henderson, D. Ranasinghe, V. G. Zakrzewski, J. Gao, N. Rega, G. Zheng, W. Liang, M. Hada, M. Ehara, K. Toyota, R. Fukuda, J. Hasegawa, M. Ishida, T. Nakajima, Y. Honda, O. Kitao, H. Nakai, T. Vreven, K. Throssell, J. A. Montgomery, Jr., J. E. Peralta, F. Ogliaro, M. J. Bearpark, J. J. Heyd, E. N. Brothers, K. N. Kudin, V. N. Staroverov, T. A. Keith, R. Kobayashi, J. Normand, K. Raghavachari, A. P. Rendell, J. C. Burant, S. S. Iyengar, J. Tomasi, M. Cossi, J. M. Millam, M. Klene, C. Adamo, R. Cammi, J. W. Ochterski, R. L. Martin, K. Morokuma, O. Farkas, J. B. Foresman, D. J. Fox, Gaussian, Inc., Wallingford CT, 2016.
14. J.-D. Chai and M. Head-Gordon, *Phys. Chem. Chem. Phys*. **2008**, *10*, 6615–6620.
15. (a) F. Weigend and R. Ahlrichs, *Phys. Chem. Chem. Phys*. **2005**, *7*, 3297–3305; (b) F. Weigend, *Phys. Chem. Chem. Phys*. **2006**, *8*, 1057–1065.
16. A. V. Marenich, C. J. Cramer and D. G. Truhlar, *J. Phys. Chem. B*. **2009**, *113*, 6378–6396.
17. (a) C. Gonzalez and H. B. Schlegel, *J. Phys. Chem*. **1990**, *94*, 5523–5527. (b) K. Fukui, *Acc. Chem. Res*. **1981**, *14*, 363–368.
18. CYLview20; Legault, C. Y., Université de Sherbrooke, 2020 (http://www.cylview.org).

10. X-ray crystallography data (3ha)

Data Collection

A colourless block crystal of **3ha** (**CCDC 2443987**) was prepared in *n*-hexane/chloroform, having approximate dimensions of 0.500 x 0.500 x 0.500 mm was mounted on a glass fiber. All measurements were made on a Rigaku R-AXIS RAPID diffractometer using graphite monochromated Mo-Kα radiation. The data were collected at a temperature of -100 + 1 °C to a maximum 2θ value of 55.0°. A total of 44 oscillation images were collected. A sweep of data was done using ω scans from 130.0 to 190.0° in 5.0° step, at χ=45.0° and ϕ = 0.0°. The exposure rate was 10.0 [sec./°]. A second sweep was performed using ω scans from 0.0 to 160.0° in 5.0° step, at χ=45.0° and ϕ = 180.0°. The exposure rate was 10.0 [sec./°]. The crystal-to-detector distance was 127.40 mm. Readout was performed in the 0.100 mm pixel mode.

X-Ray crystallographic structure of Product 3ha

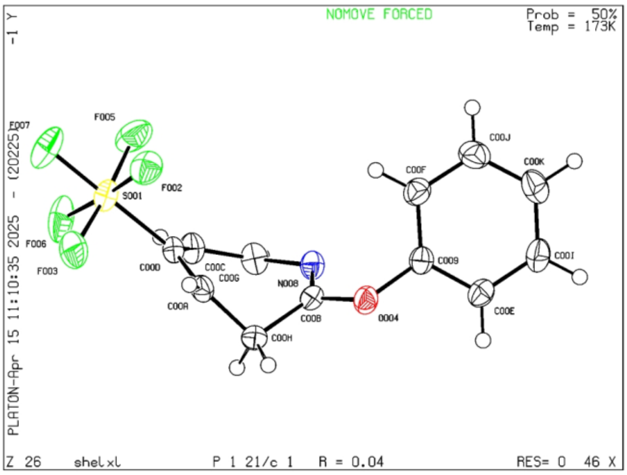

Figure S2: Ortep diagram of **3ha**

Table 1: Crystal data, data collection and structure refinement details of compound 3ha

Bond precision:	C-C = 0.0020 A		Wavelength=0.71075
Cell:	a=12.2626(5)	b=9.5598(3)	c=11.2744(4)
	alpha=90	beta=100.372(7)	gamma=90
Temperature:	173 K		

	Calculated	Reported
Volume	1300.08(9)	1300.08(9)
Space group	P 21/c	P 1 21/c 1
Hall group	-P 2ybc	-P 2ybc
Moiety formula	C12 H10 F5 N O S	C12 H10 F5 N O S
Sum formula	C12 H10 F5 N O S	C12 H10 F5 N O S
Mr	311.27	311.27
Dx,g cm-3	1.590	1.590
Z	4	4
Mu (mm-1)	0.303	0.303
F000	632.0	632.0
F000'	632.99	
h,k,lmax	15,12,14	15,12,14
Nref	2985	2972
Tmin,Tmax	0.859,0.859	1.000,1.000
Tmin'	0.859	

Correction method= # Reported T Limits: Tmin=1.000 Tmax=1.000
AbsCorr = MULTI-SCAN

Data completeness= 0.996 Theta(max)= 27.482

R(reflections)= 0.0381(2747) wR2(reflections)= 0.1031(2972)

S = 1.050 Npar= 190

11. ^{1}H NMR, ^{13}C NMR and ^{19}F NMR spectral copies of compounds

^{1}H NMR (700 MHz, $CDCl_3$) : **7**

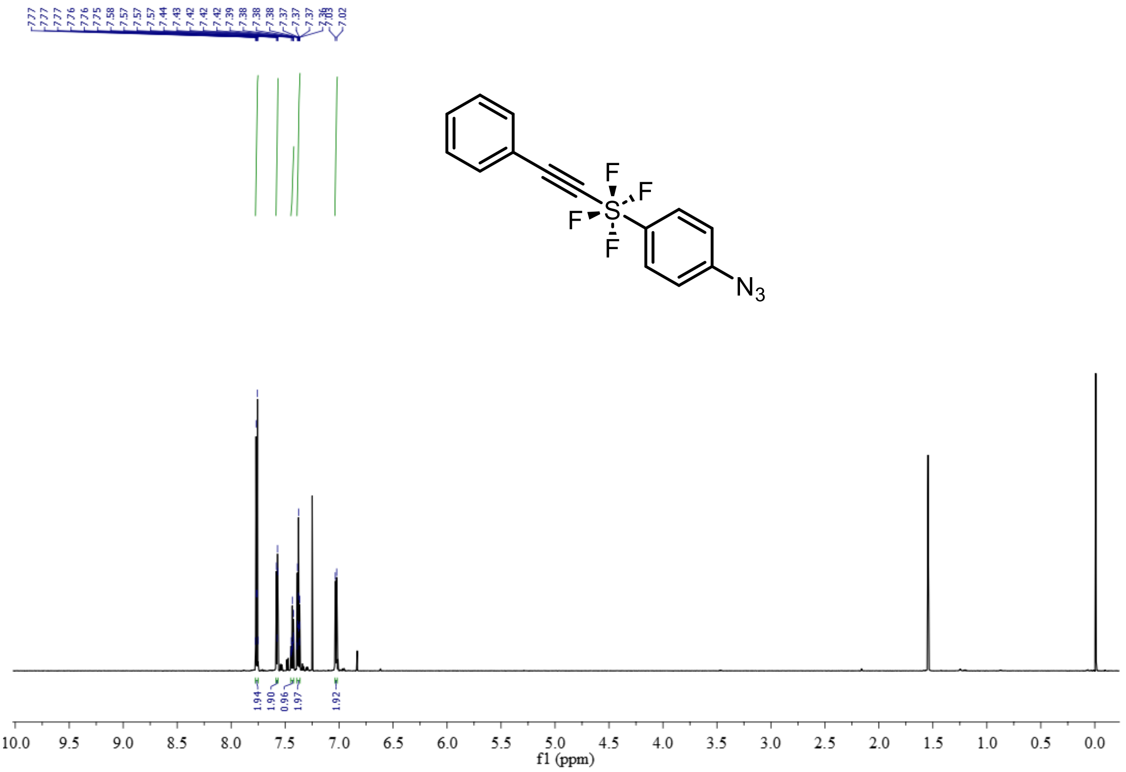

^{13}C NMR (126 MHz, $CDCl_3$) : **7**

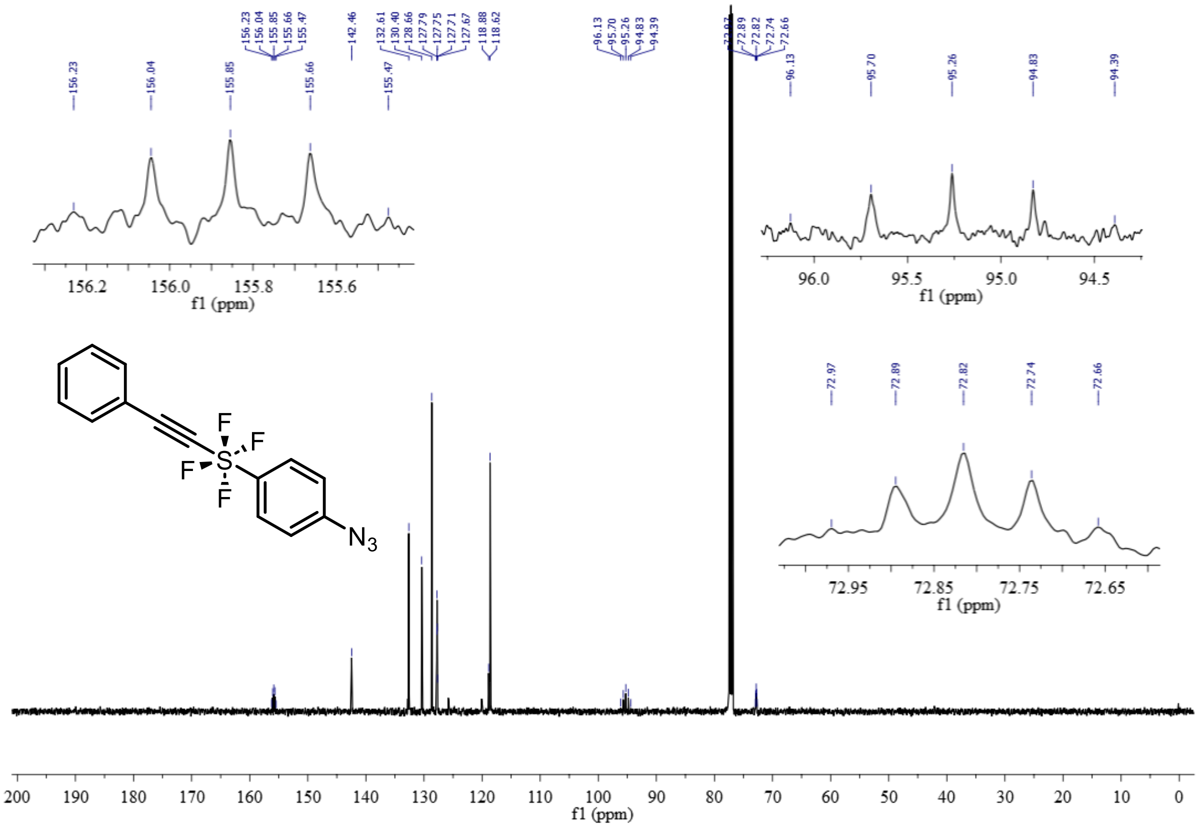

^{19}F NMR (658 MHz, $CDCl_3$) : **7**

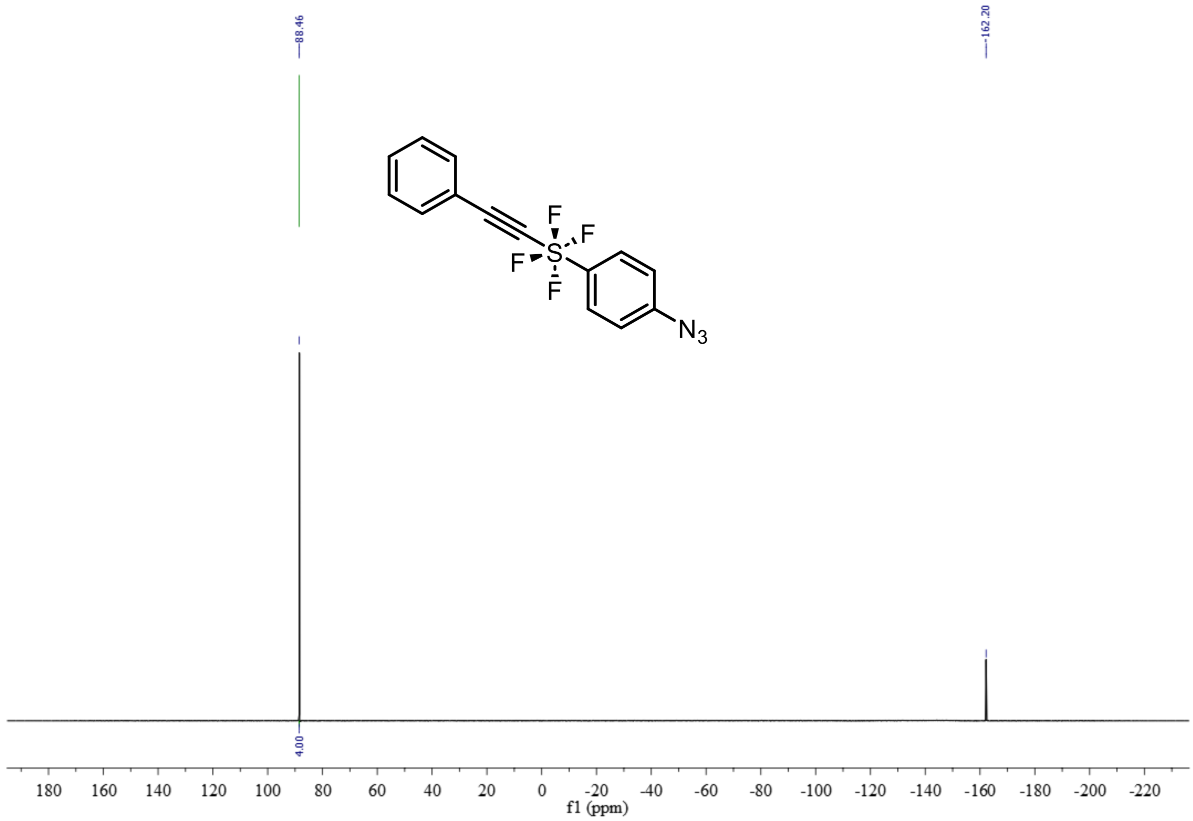

^{1}H NMR (500 MHz, $CDCl_3$) : **3aa**

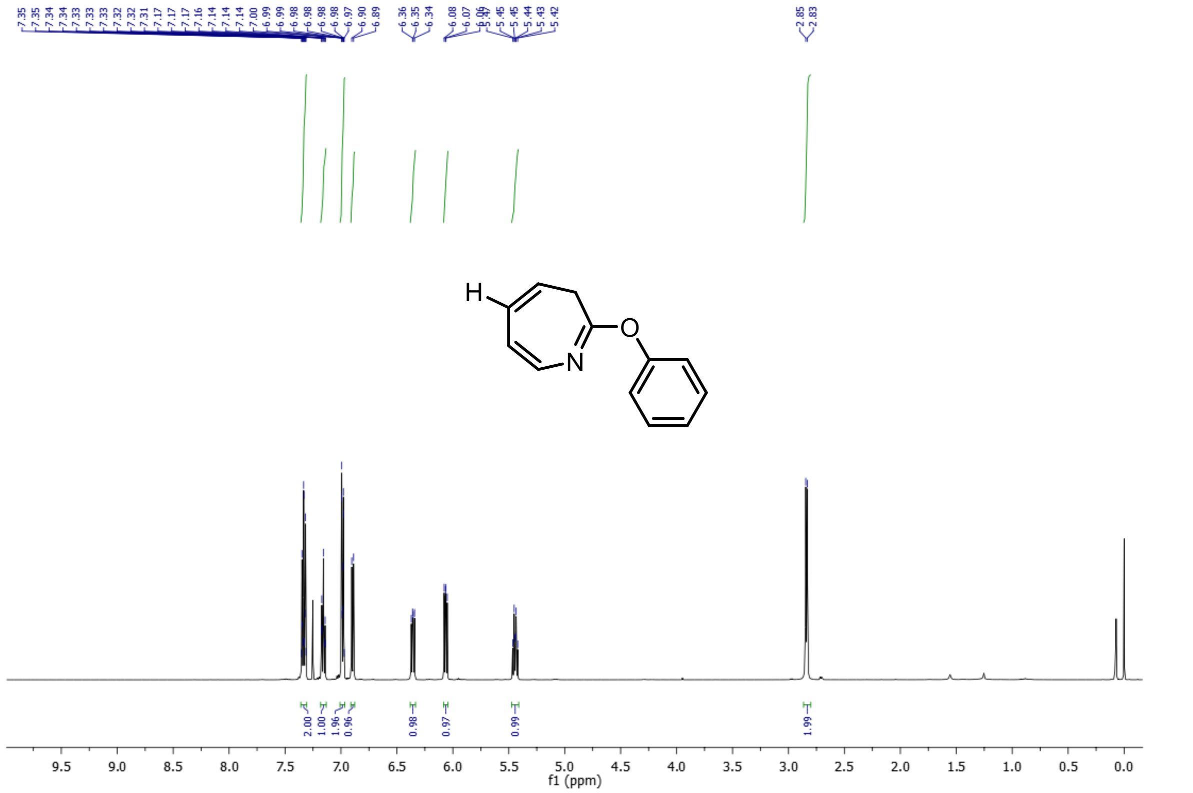

^{13}C NMR (126 MHz, $CDCl_3$) : **3aa**

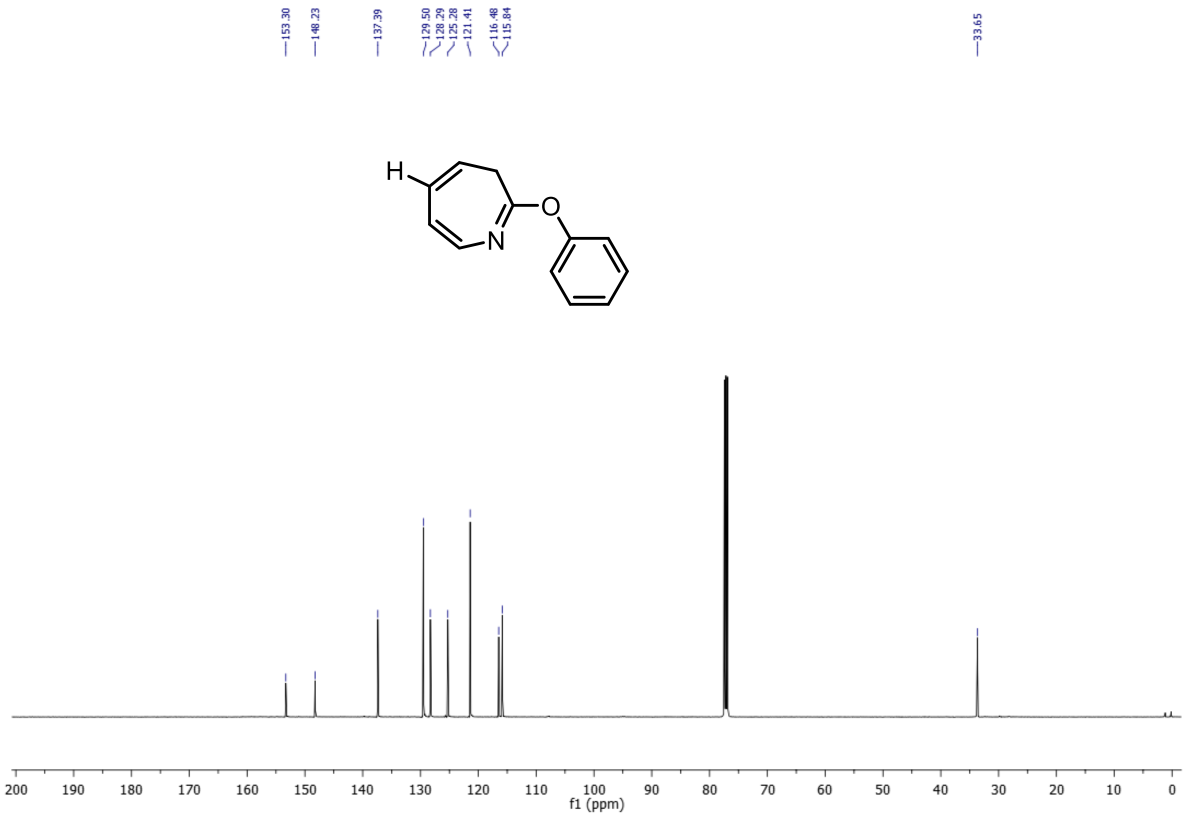

^{1}H NMR (500 MHz, $CDCl_3$) : **3ba**

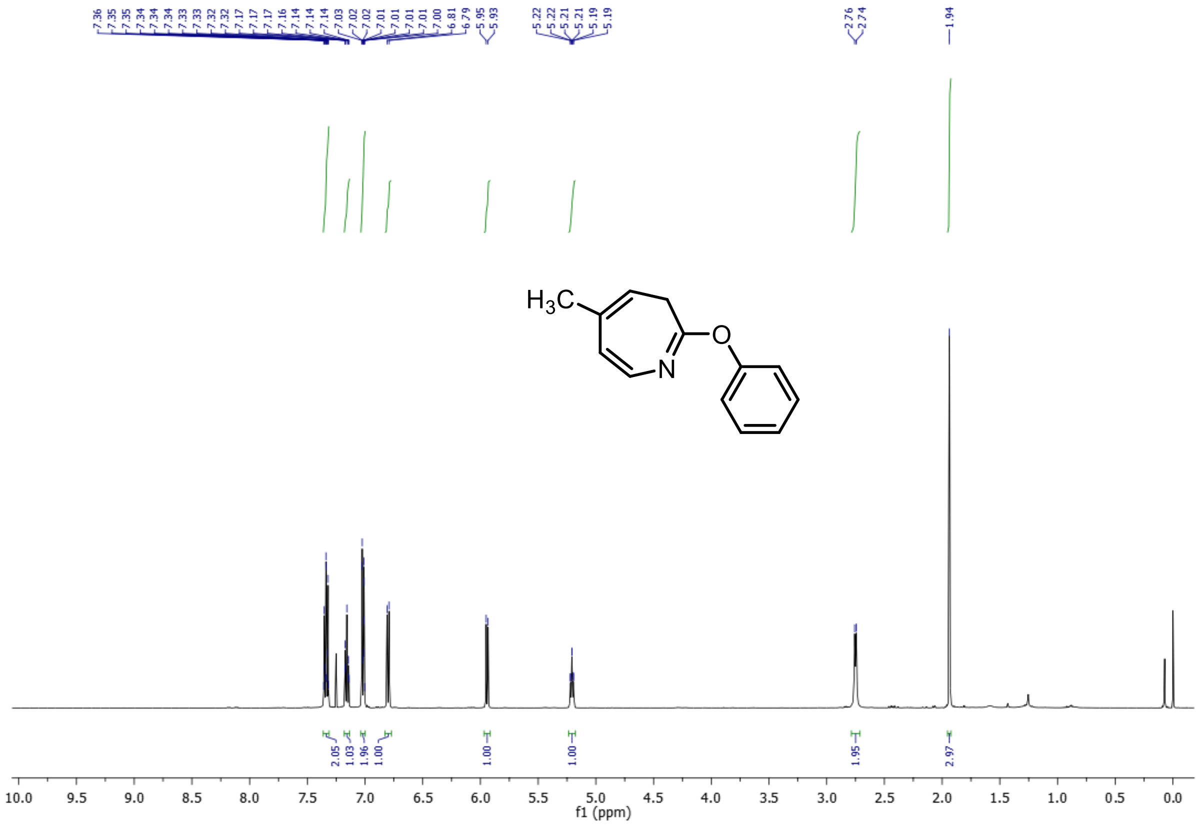

^{13}C NMR (126 MHz, $CDCl_3$) : **3ba**

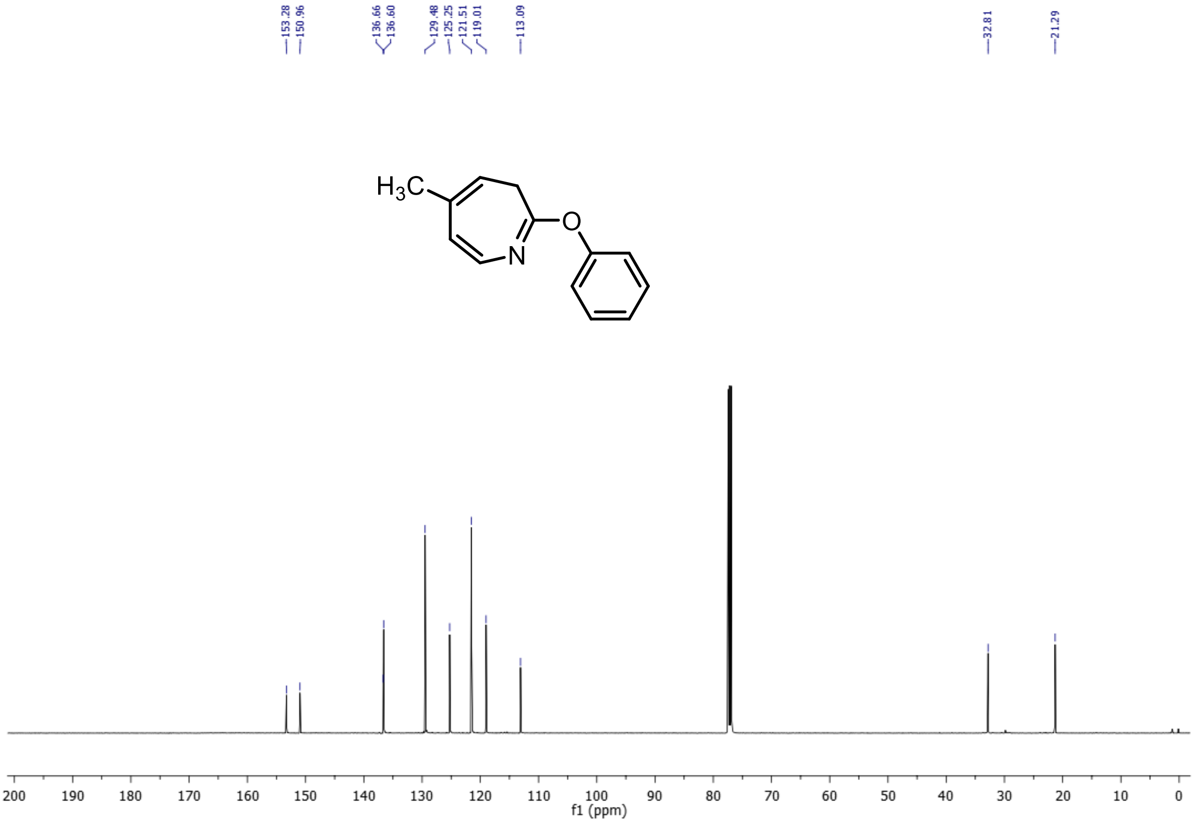

^{1}H NMR (500 MHz, $CDCl_3$) : **3ca**

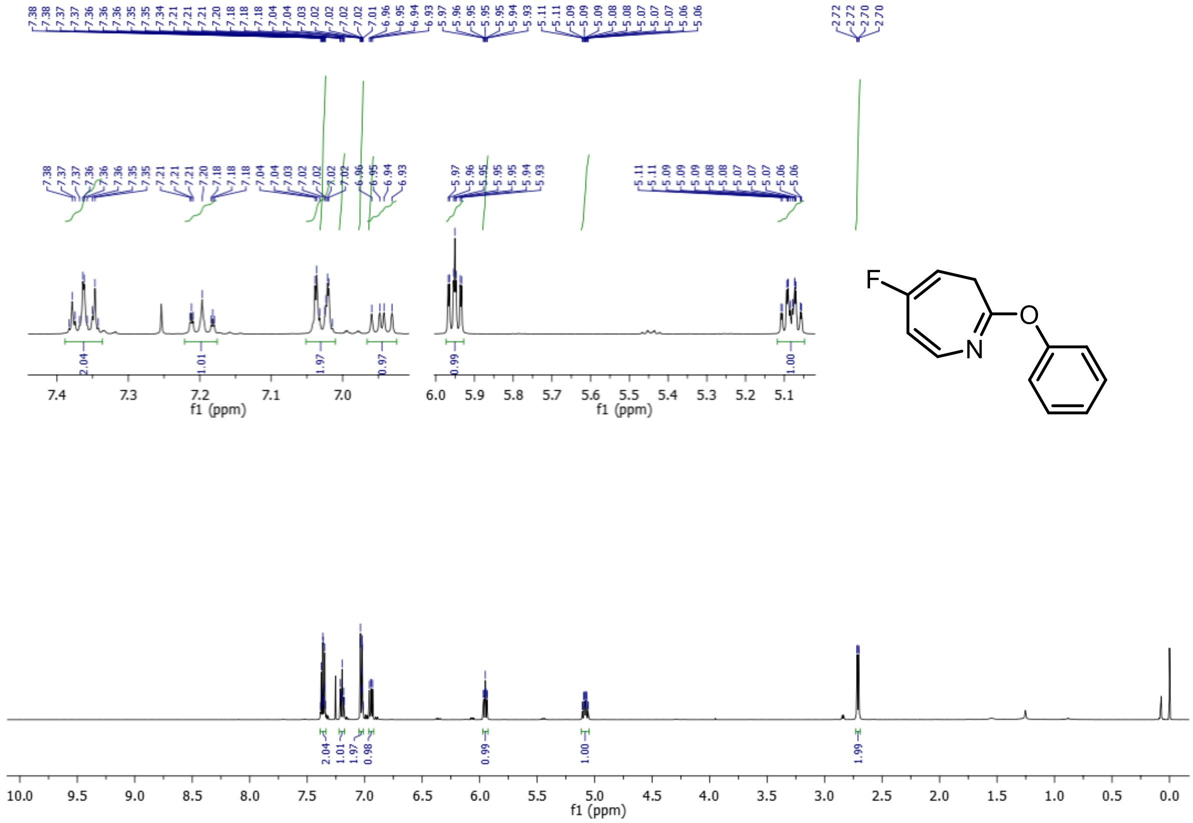

^{13}C NMR (126 MHz, $CDCl_3$) : **3ca**

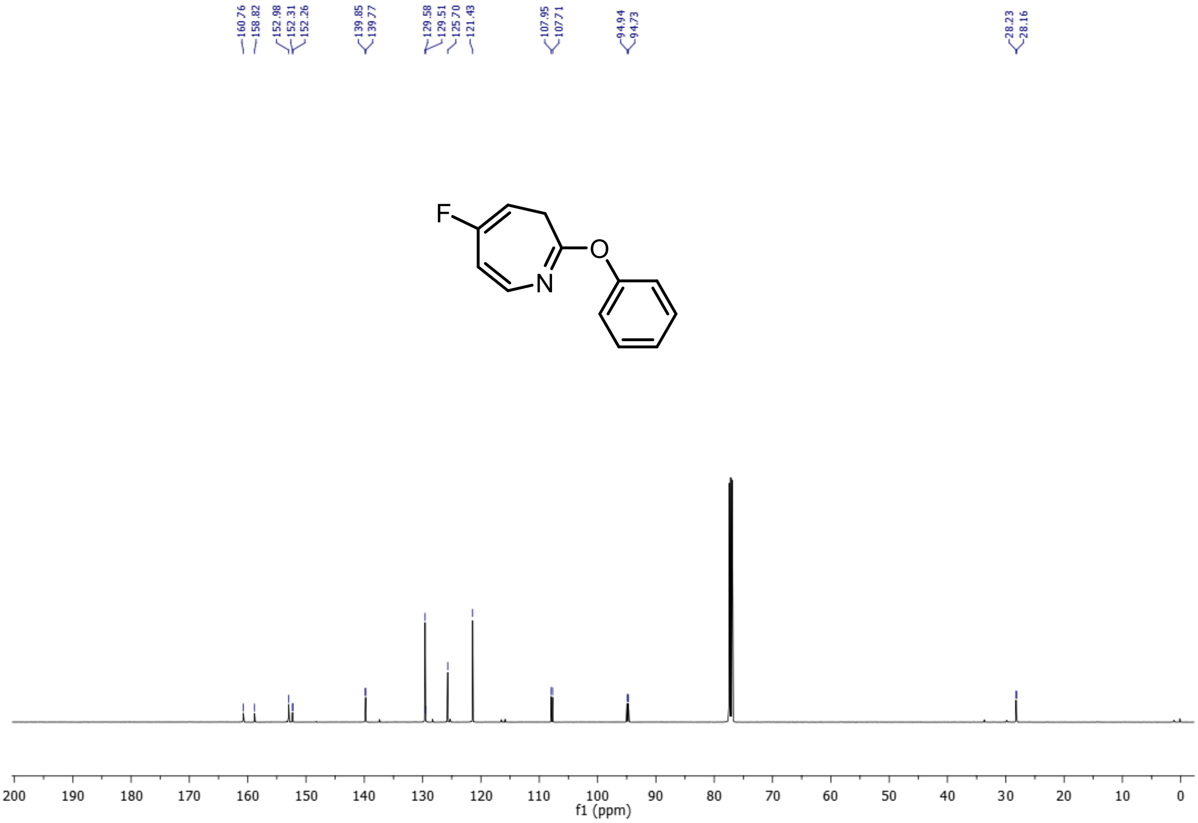

^{19}F NMR (282 MHz, $CDCl_3$) : **3ca**

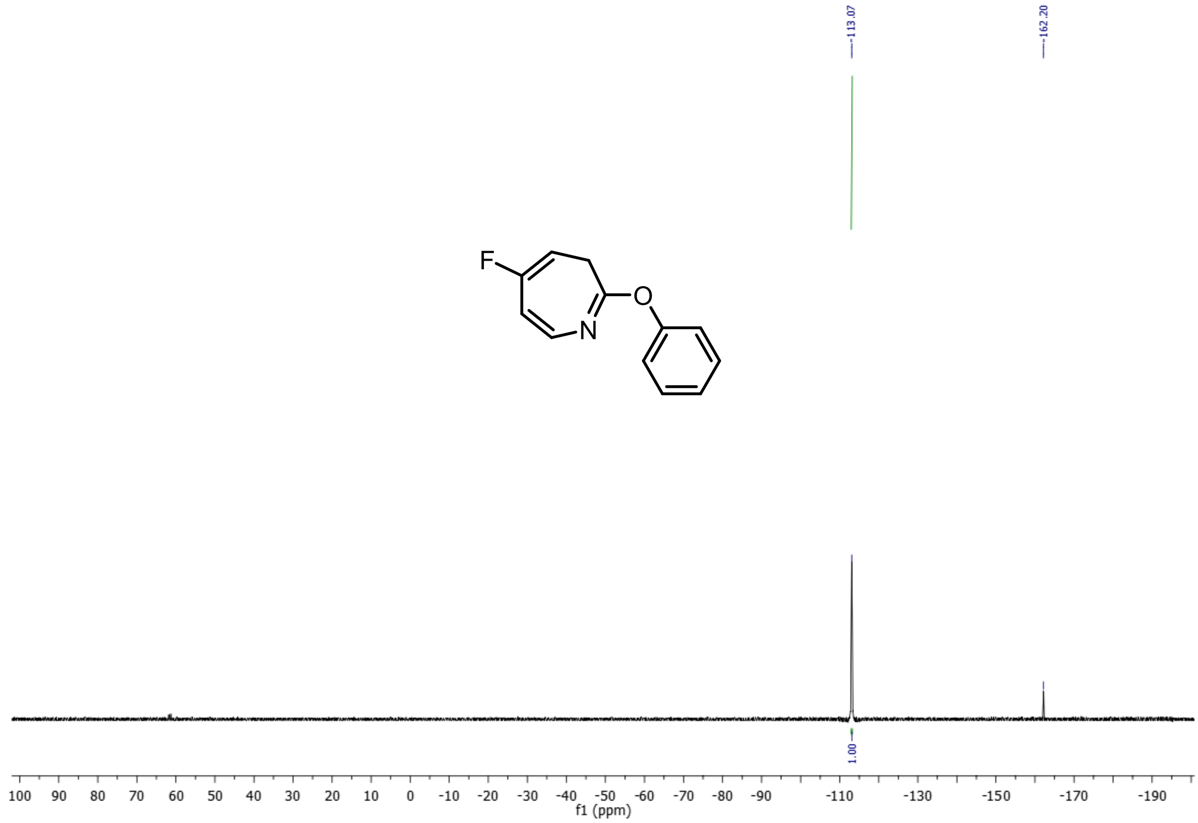

^{1}H NMR (500 MHz, $CDCl_3$) : **3da**

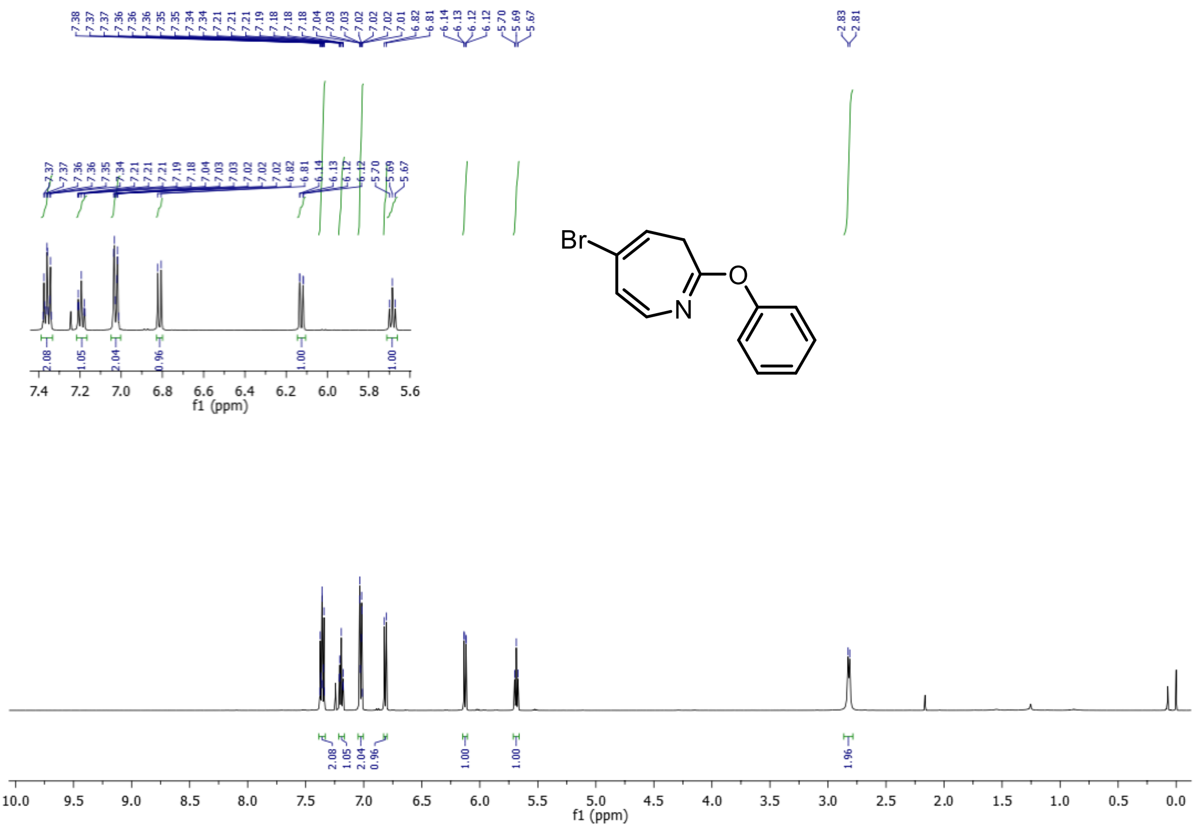

^{13}C NMR (126 MHz, $CDCl_3$) : **3da**

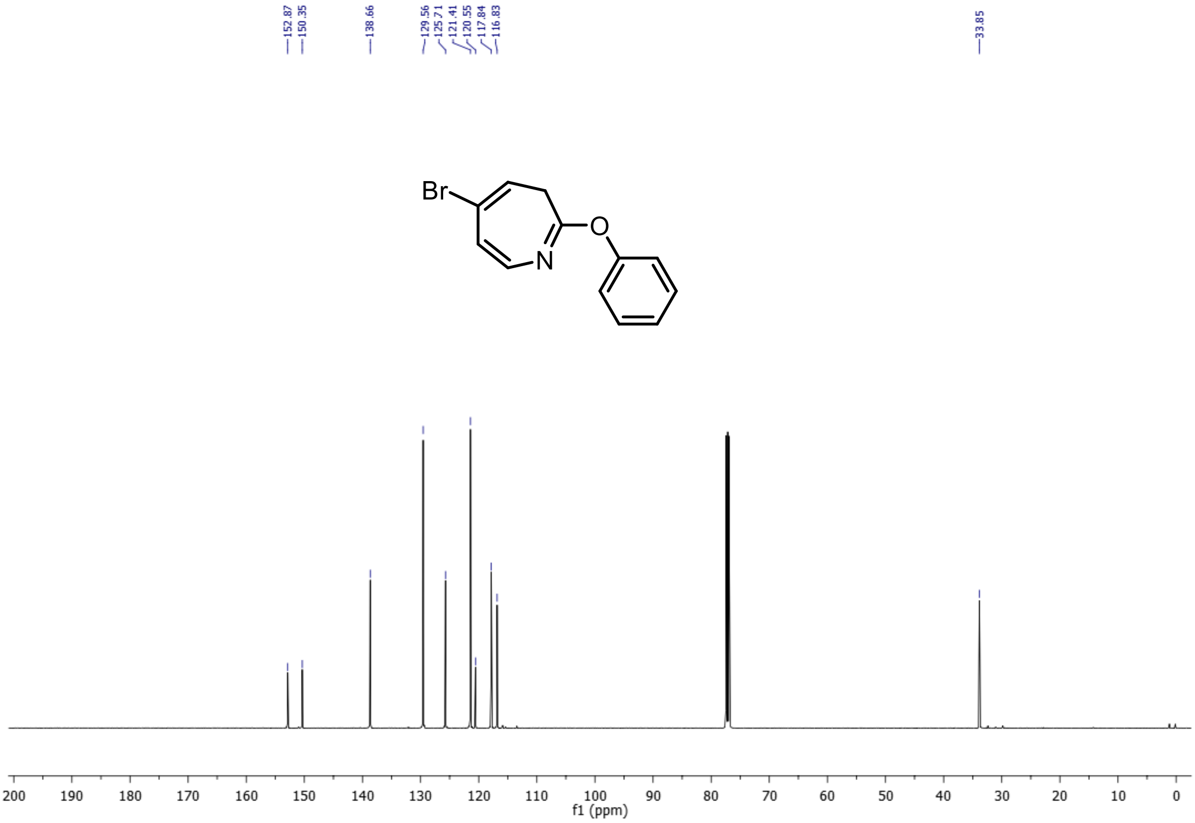

^{1}H NMR (500 MHz, $CDCl_3$) : **3ea**

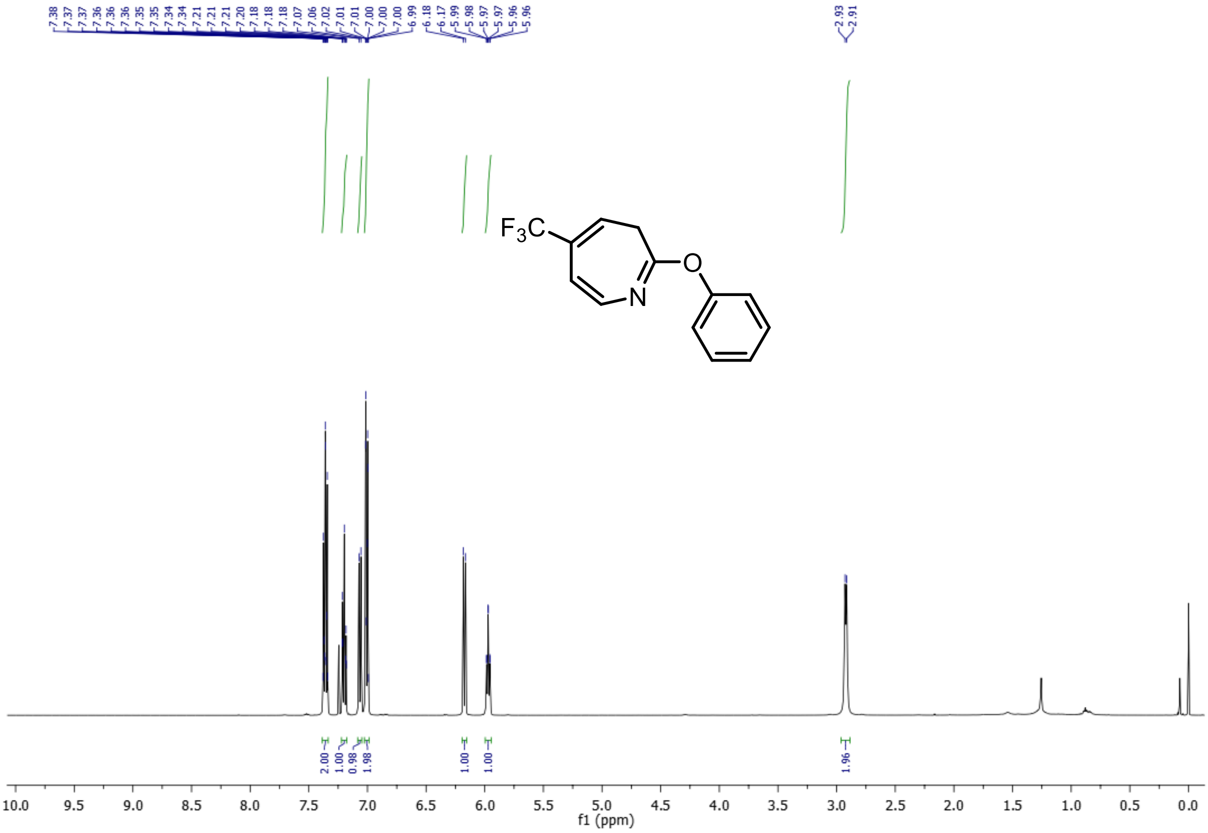

^{13}C NMR (126 MHz, $CDCl_3$) : **3ea**

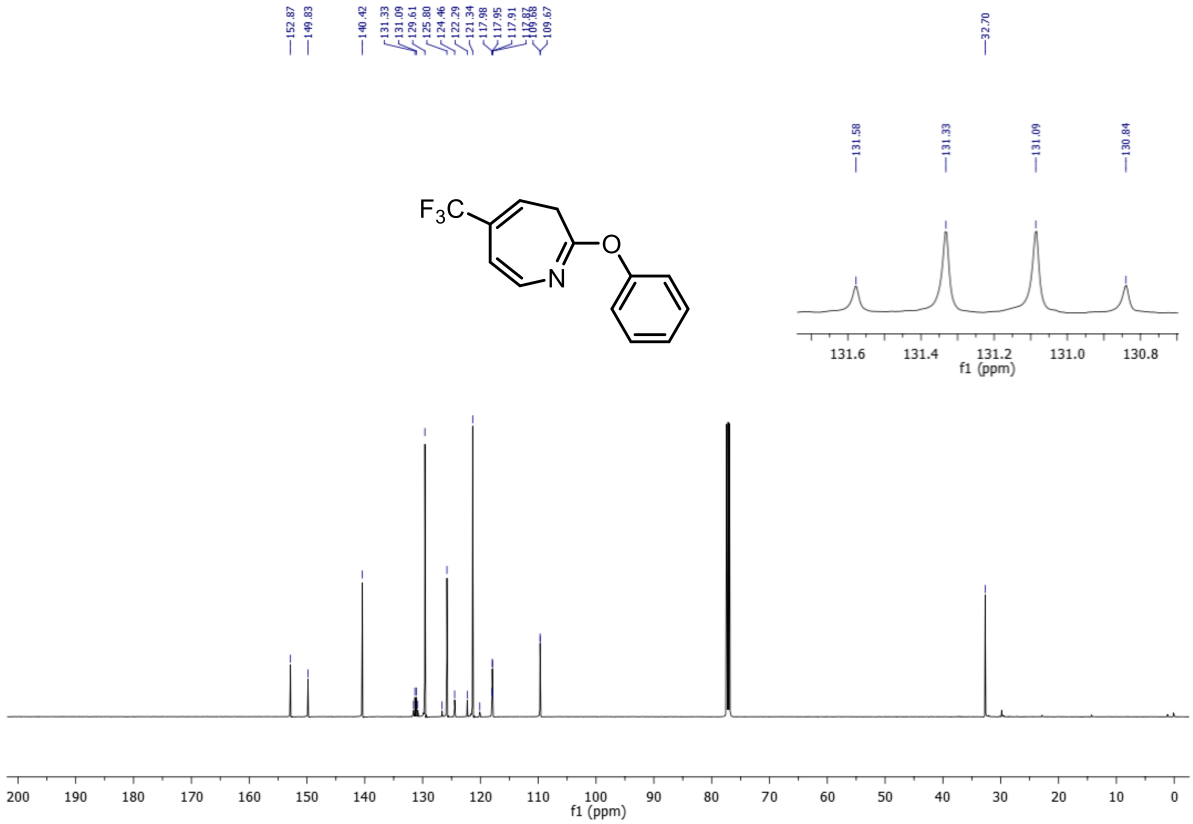

^{19}**F NMR** (282 MHz, $CDCl_3$) : **3ea**

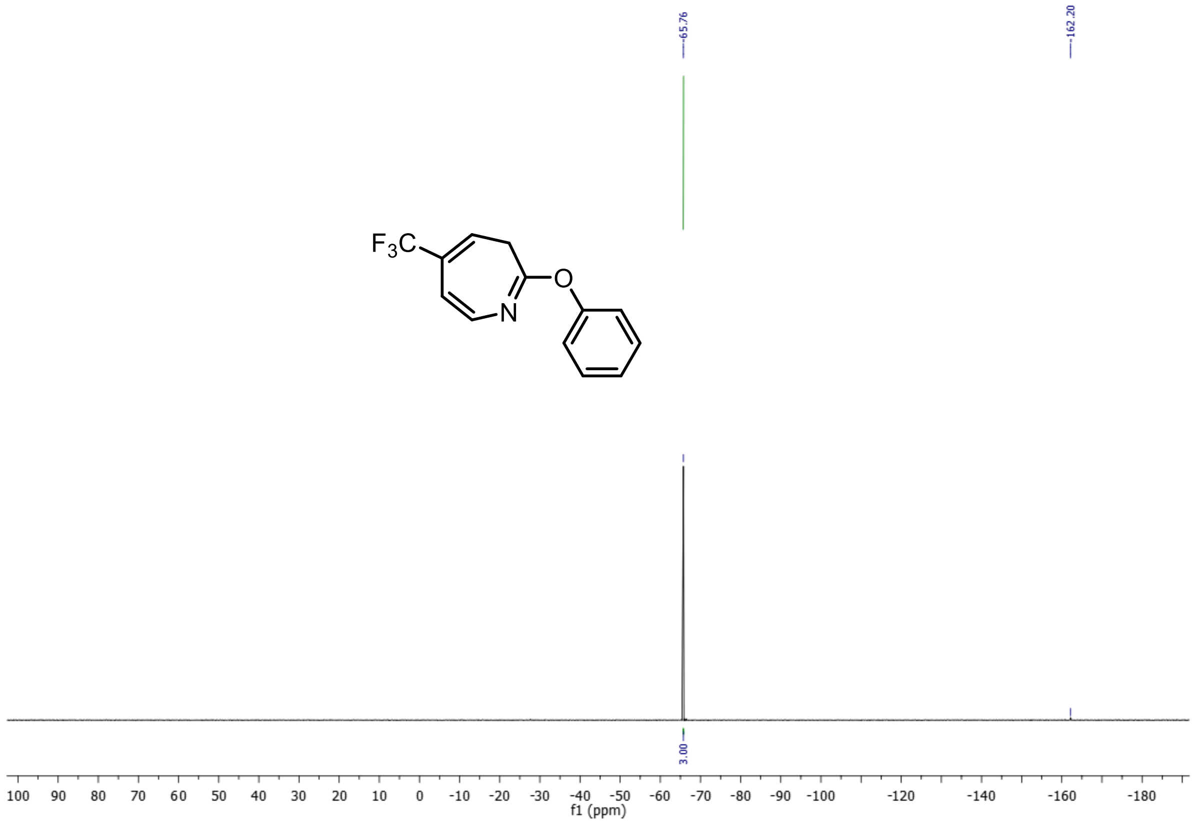

^{1}**H NMR** (500 MHz, $CDCl_3$) : **3fa**

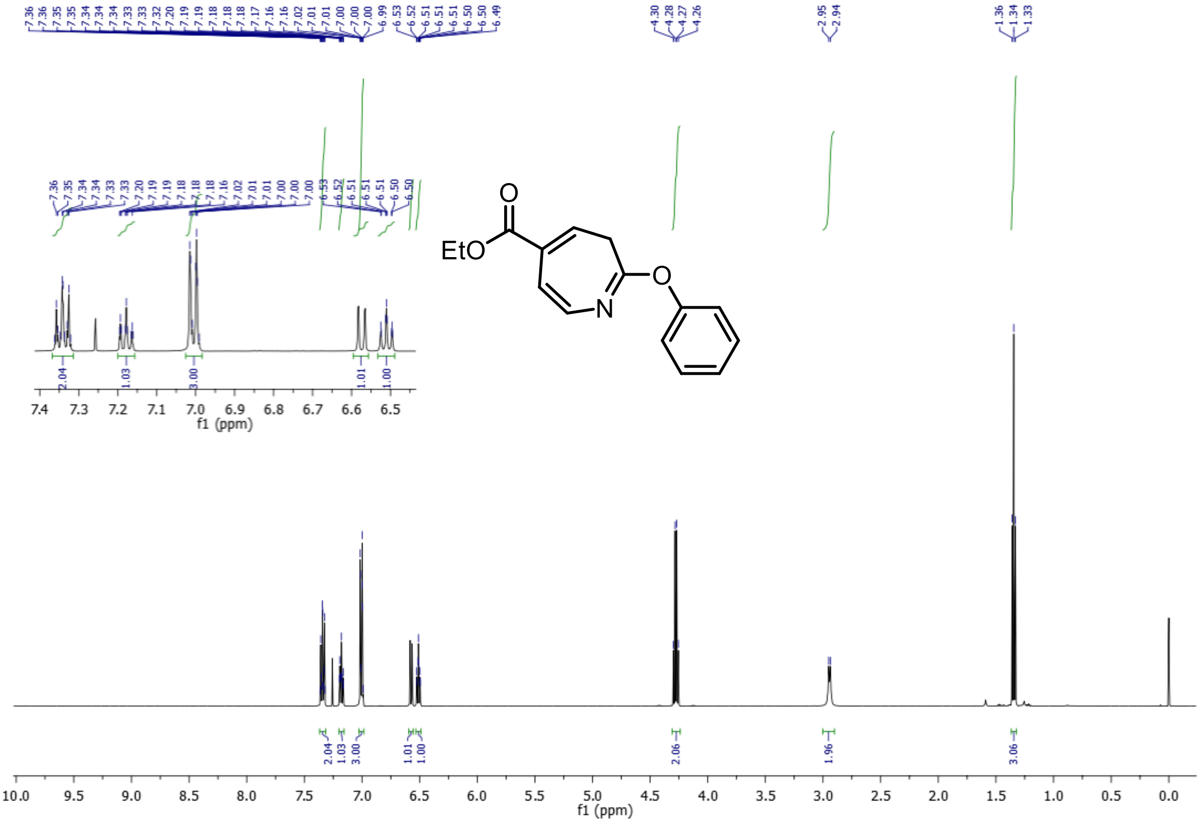

^{13}C NMR (126 MHz, CDCl$_3$) : **3fa**

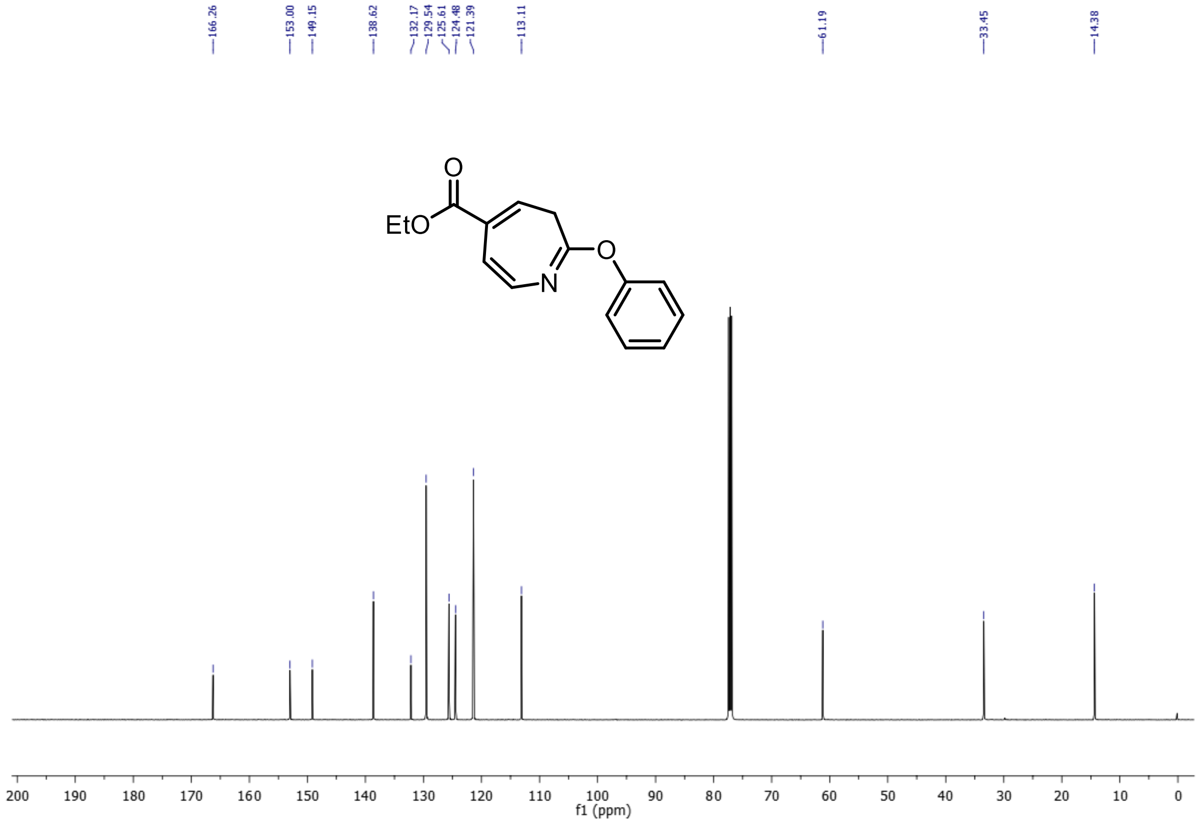

^{1}H NMR (500 MHz, CDCl$_3$) : **3ha**

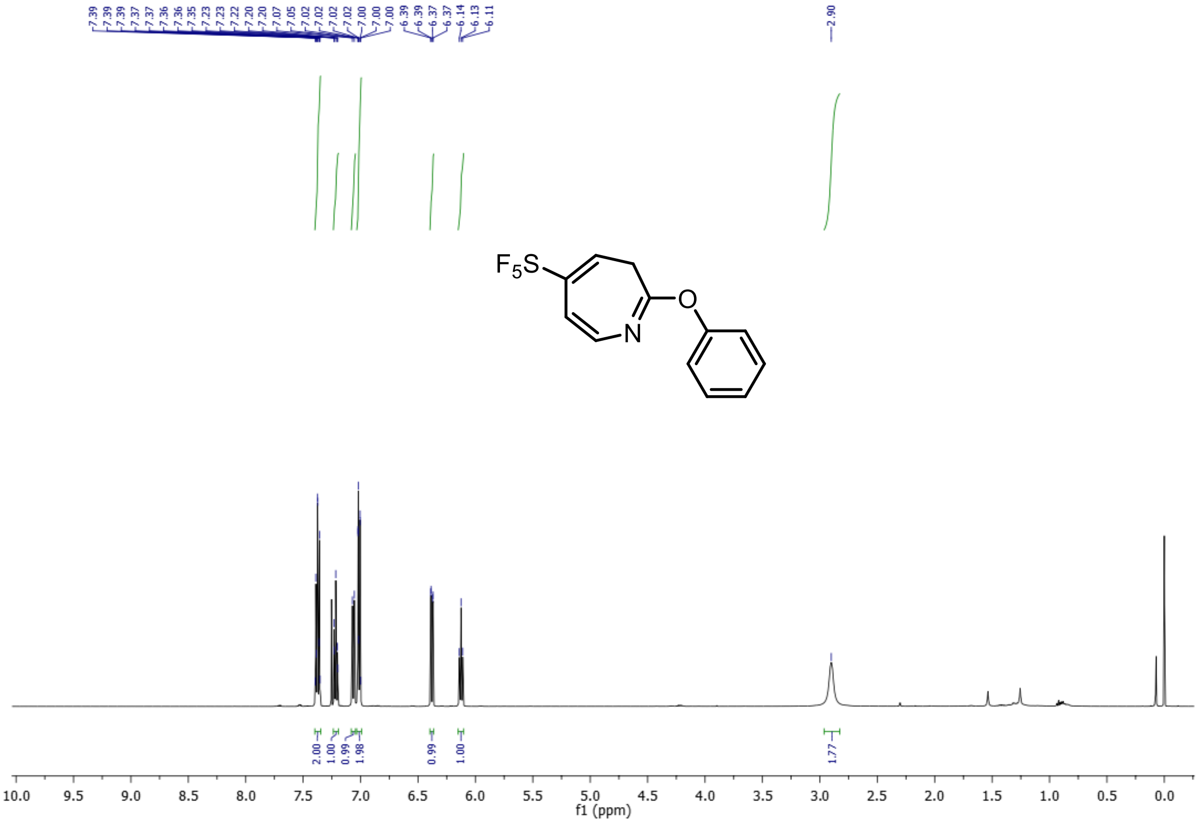

^{13}C NMR (126 MHz, $CDCl_3$) : **3ha**

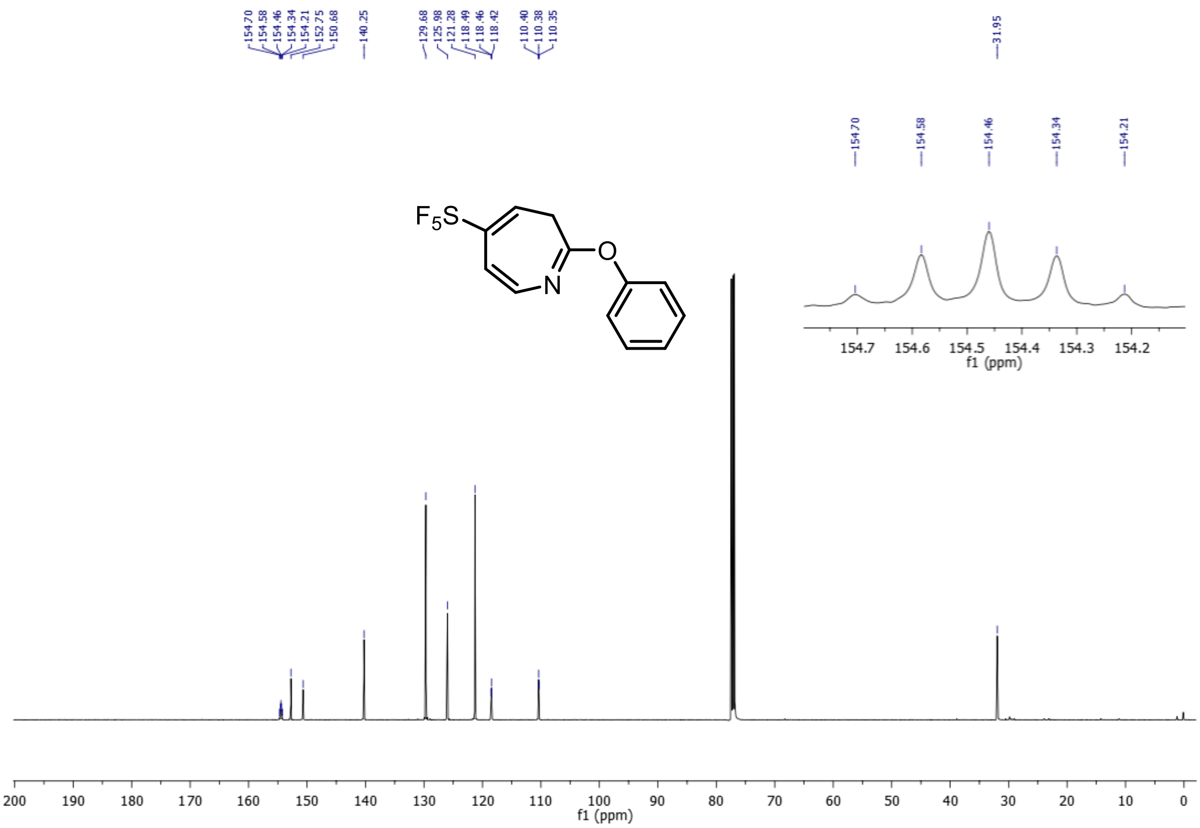

^{19}F NMR (282 MHz, $CDCl_3$) : **3ha**

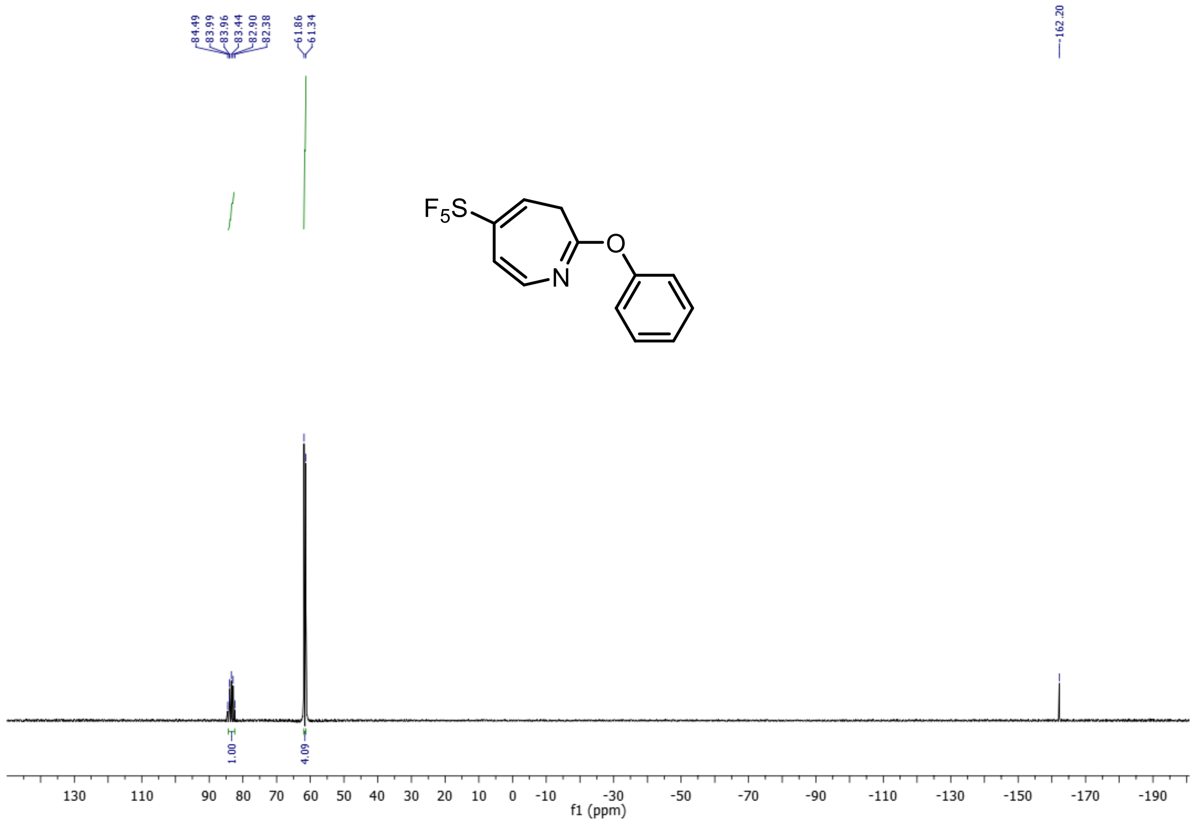

^{1}H NMR (500 MHz, CDCl$_3$) : **3hb**

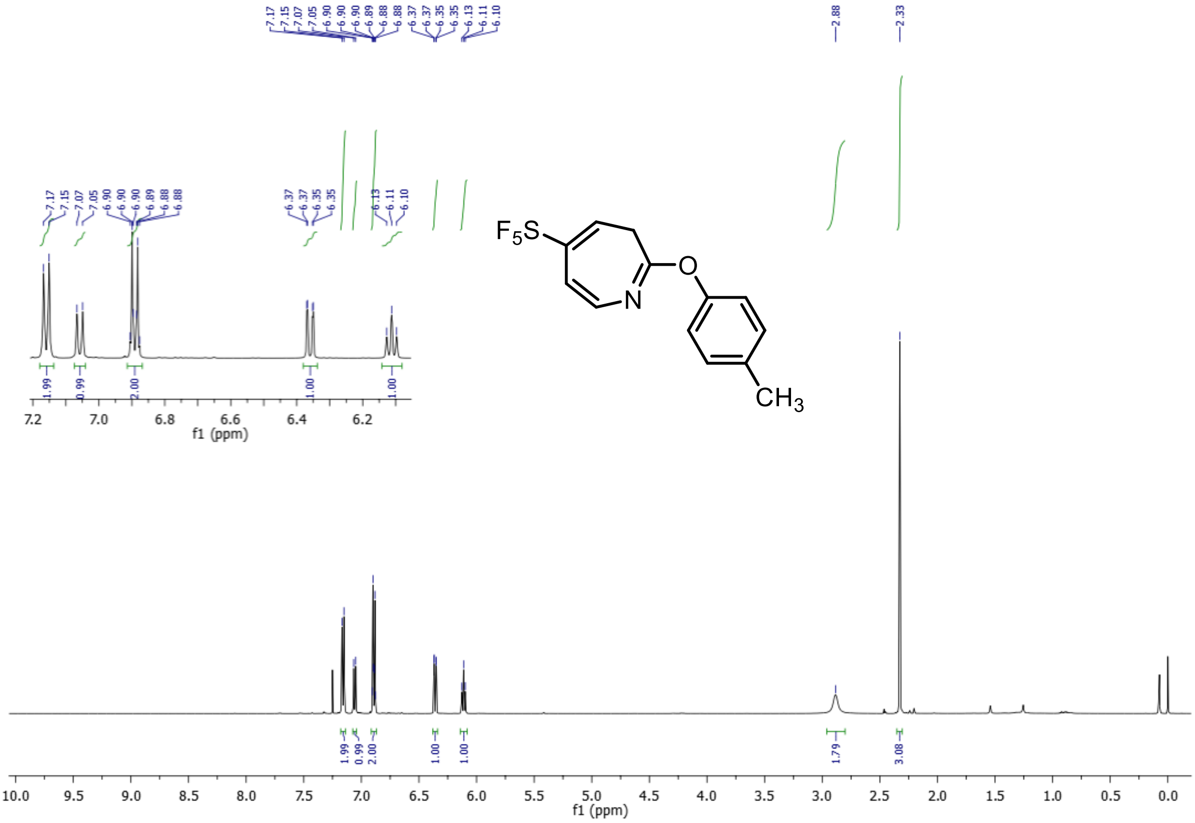

^{13}C NMR (126 MHz, CDCl$_3$) : **3hb**

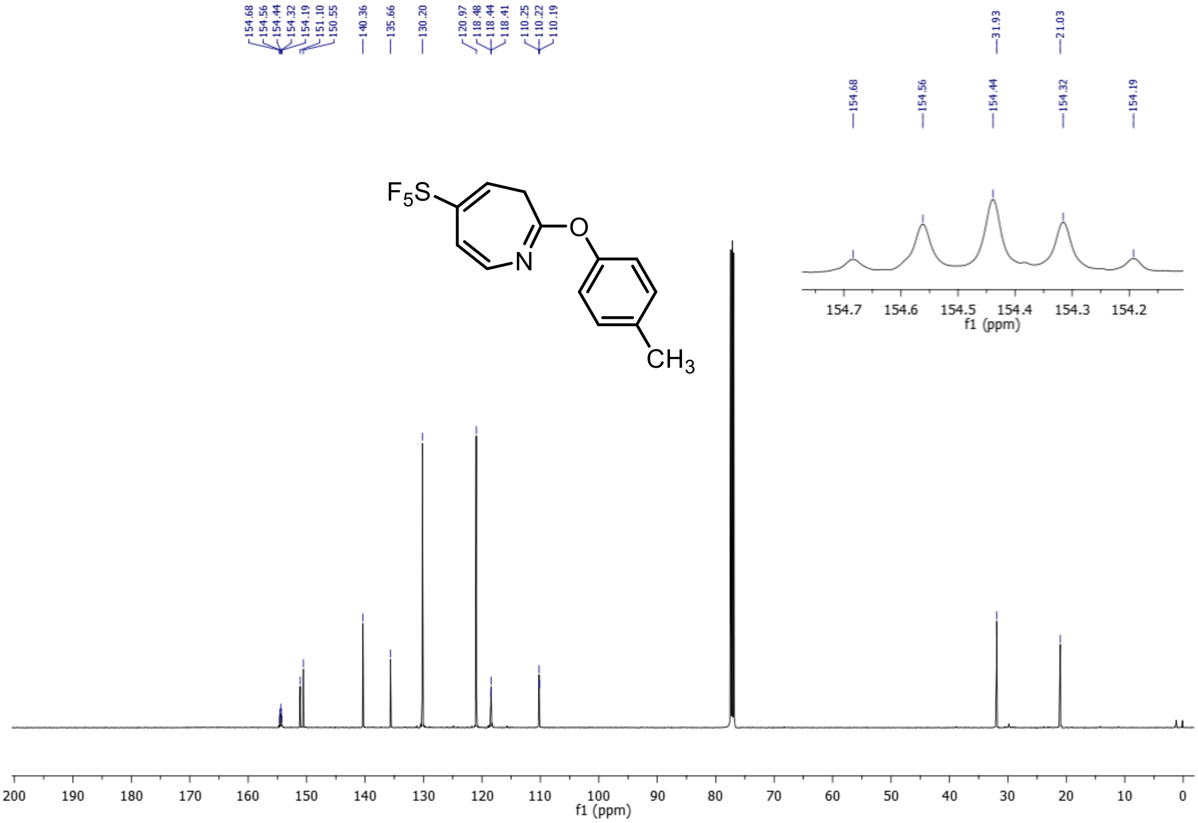

^{19}F NMR (282 MHz, $CDCl_3$) : **3hb**

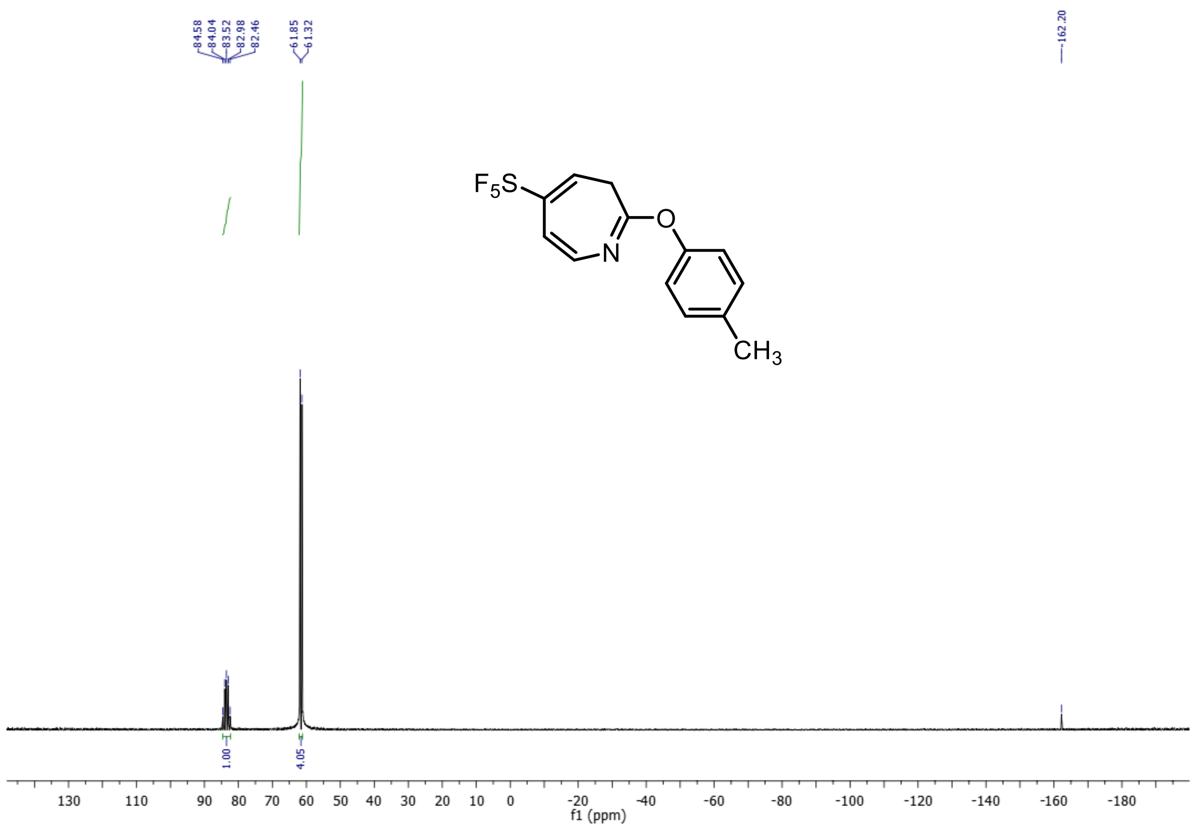

^{1}H NMR (500 MHz, $CDCl_3$) : **3hc**

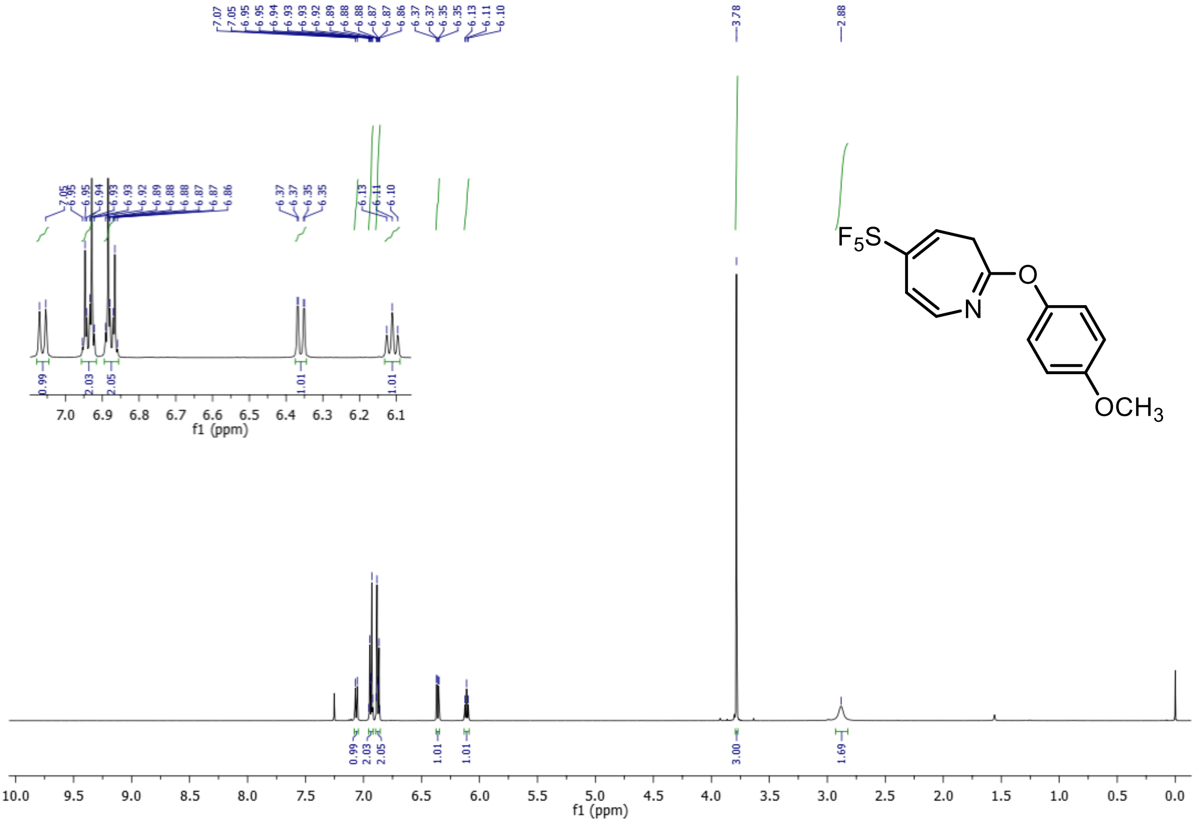

^{13}C NMR (126 MHz, $CDCl_3$) : **3hc**

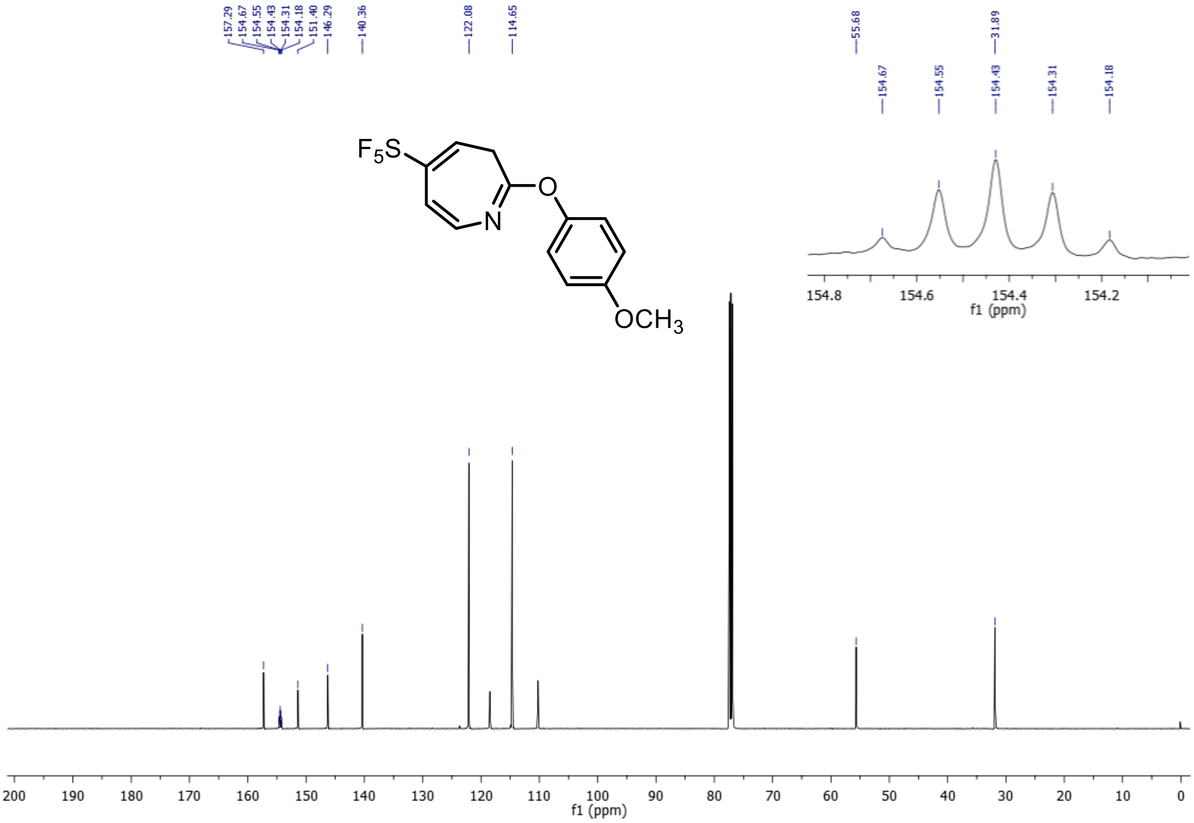

^{19}F NMR (282 MHz, $CDCl_3$) : **3hc**

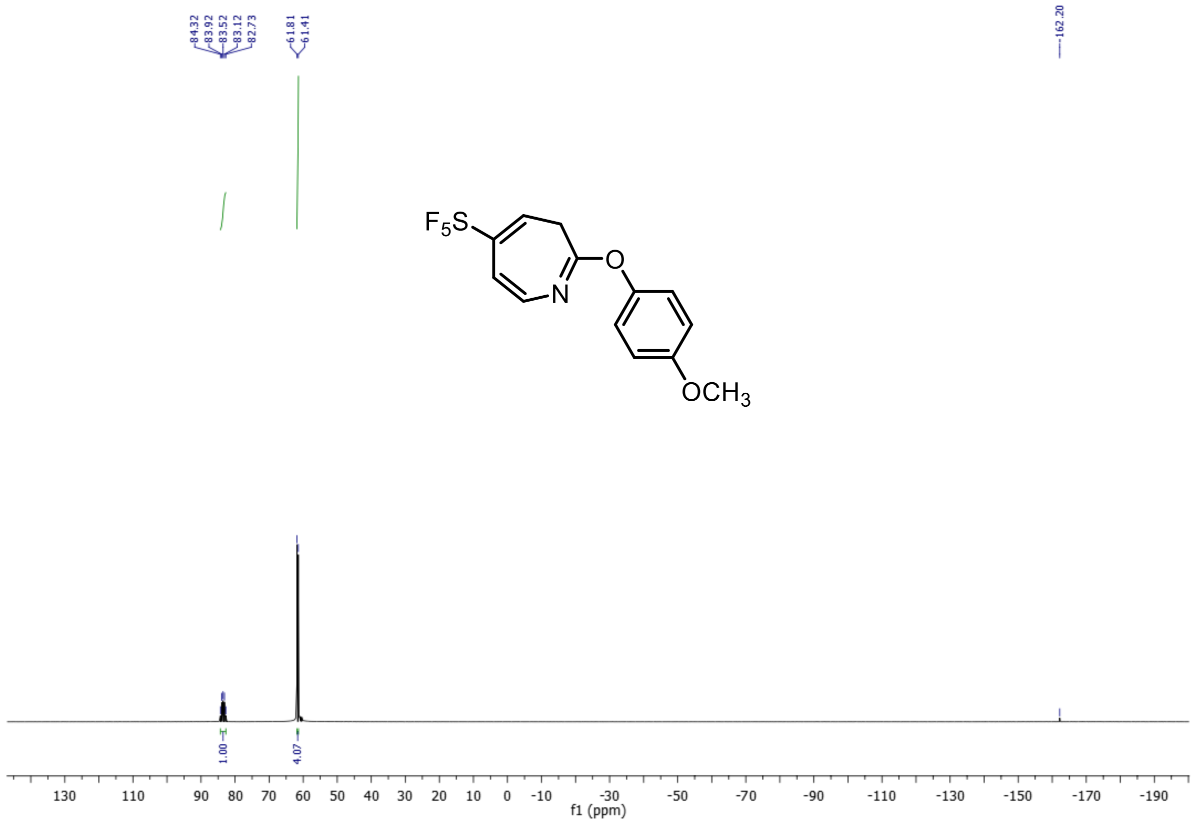

^{1}**H NMR** (500 MHz, $CDCl_3$) : **3hd**

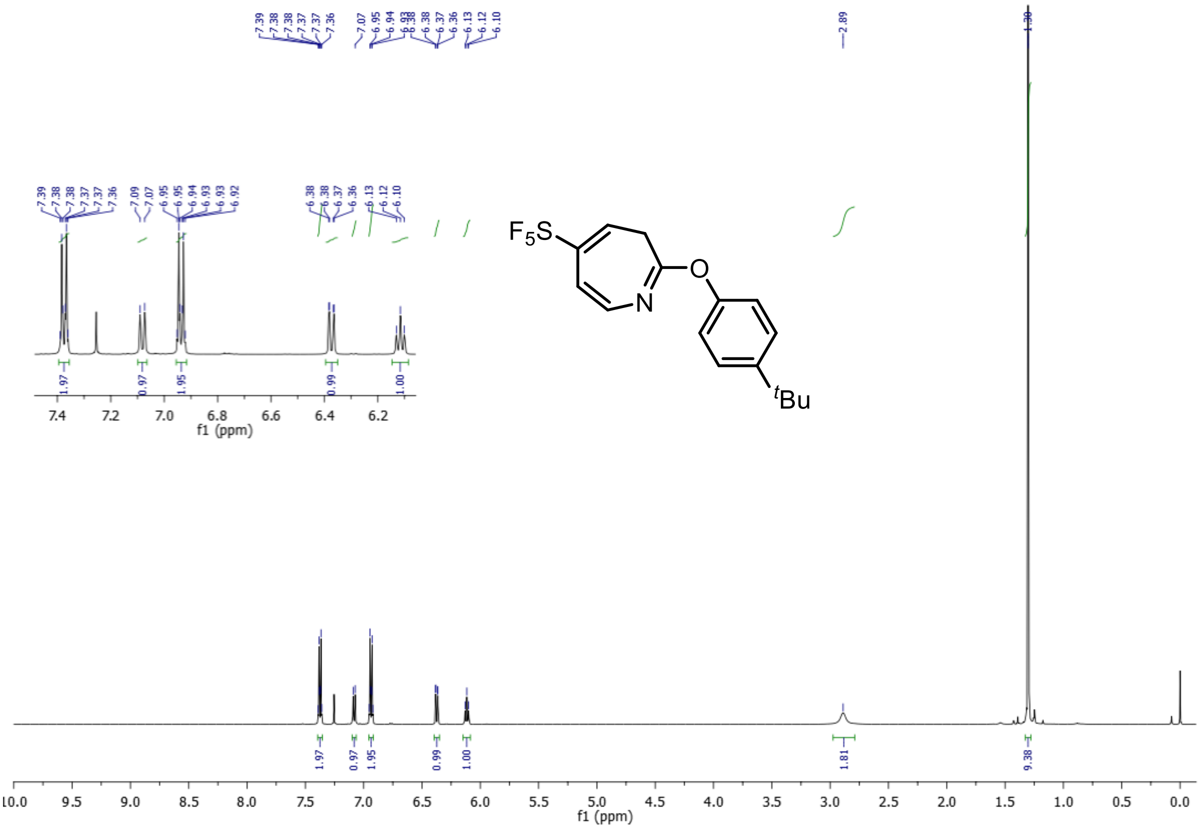

^{13}**C NMR** (126 MHz, $CDCl_3$) : **3hd**

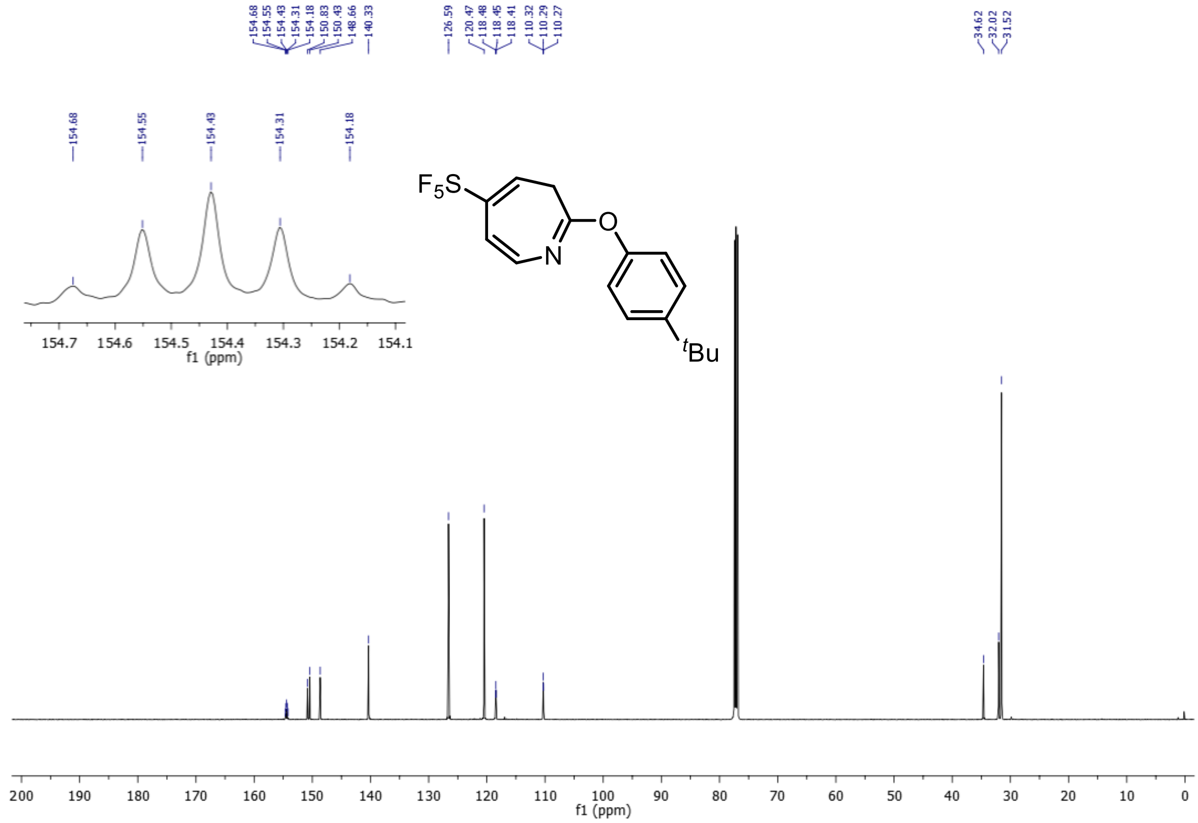

^{19}F NMR (282 MHz, $CDCl_3$) : **3hd**

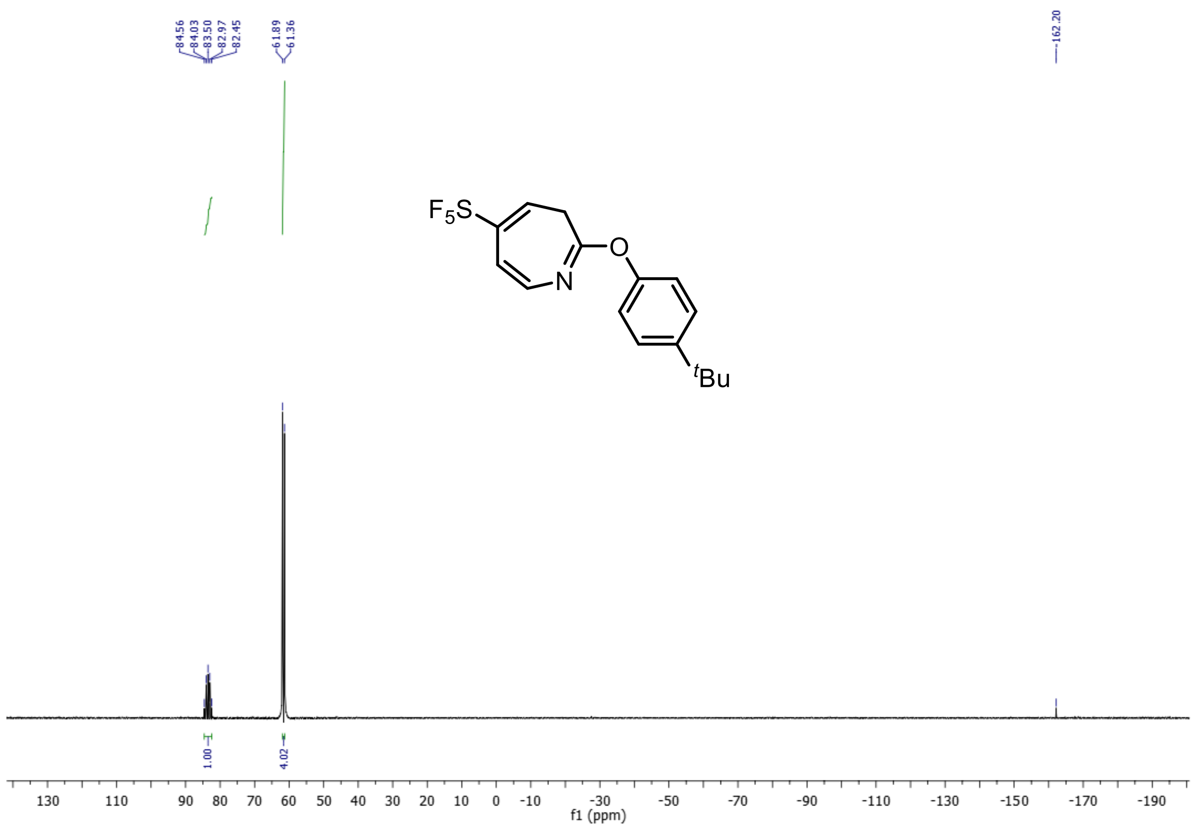

^{1}H NMR (500 MHz, $CDCl_3$) : **3he**

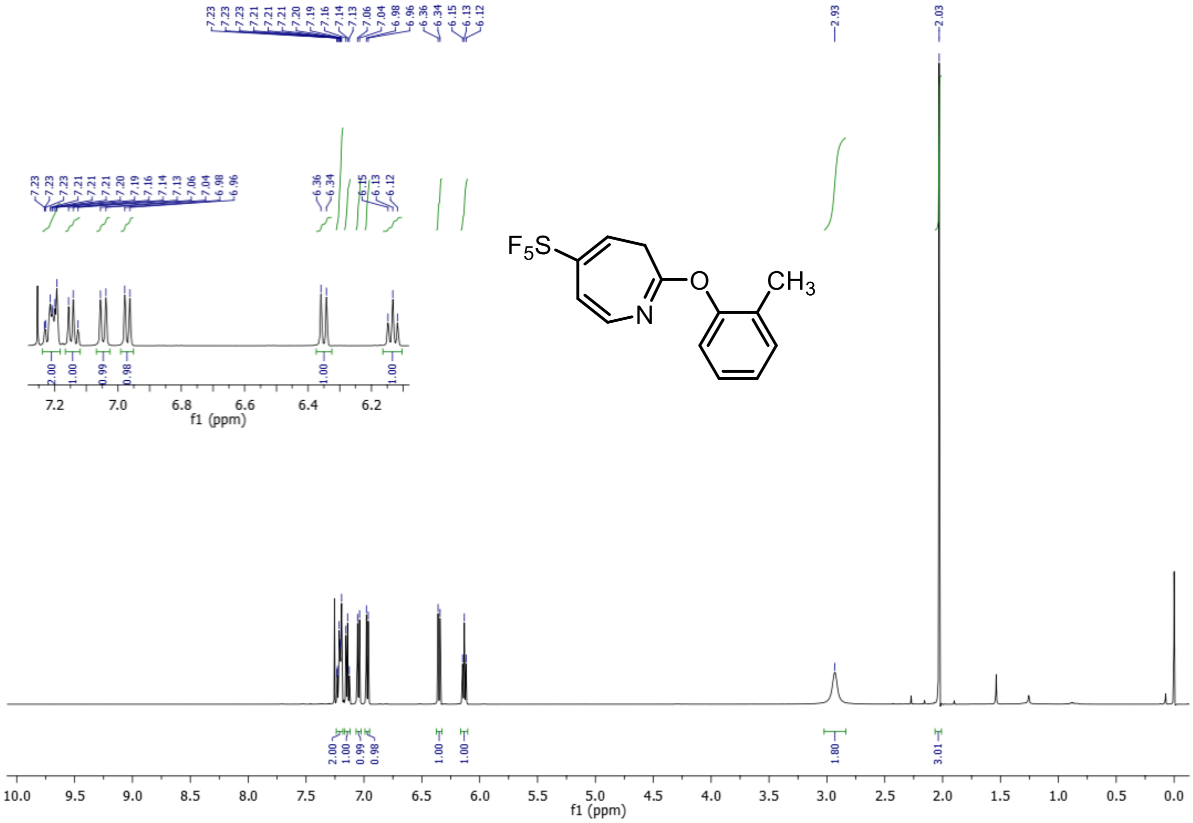

^{13}C NMR (126 MHz, $CDCl_3$) : **3he**

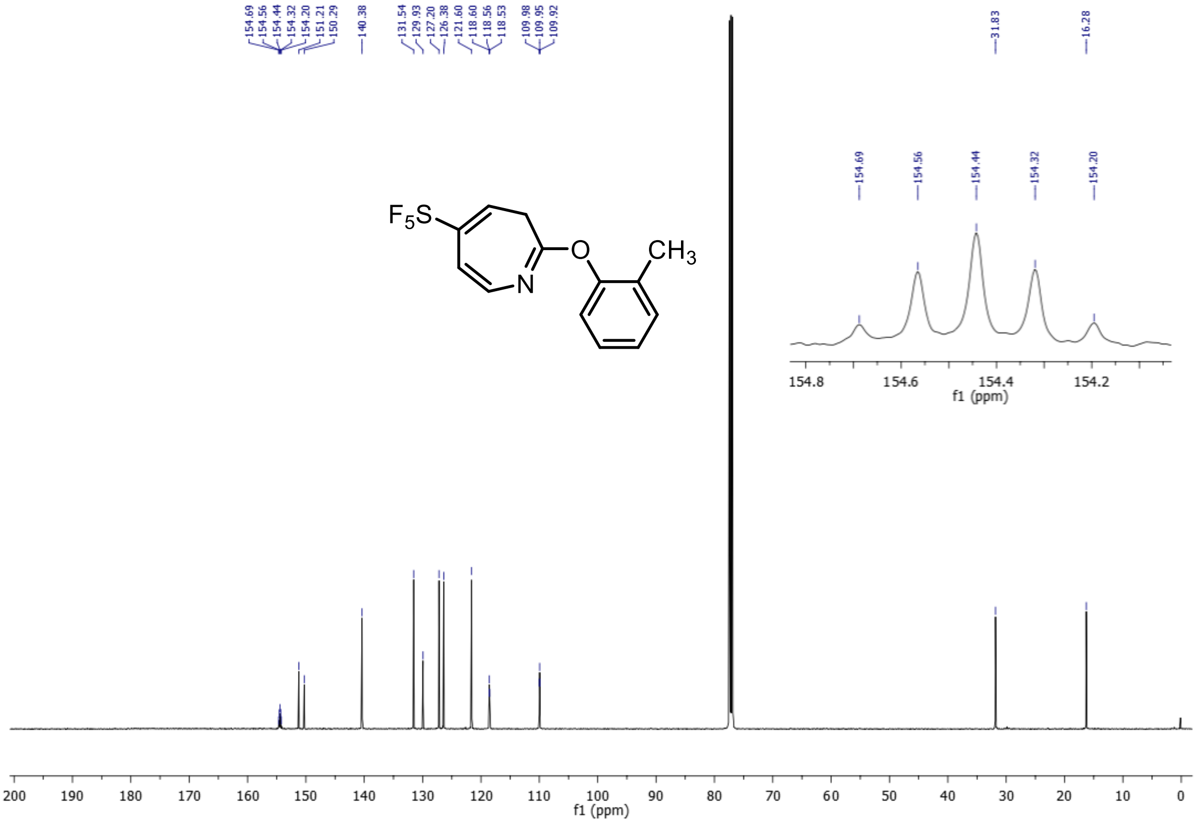

^{19}F NMR (282 MHz, $CDCl_3$) : **3he**

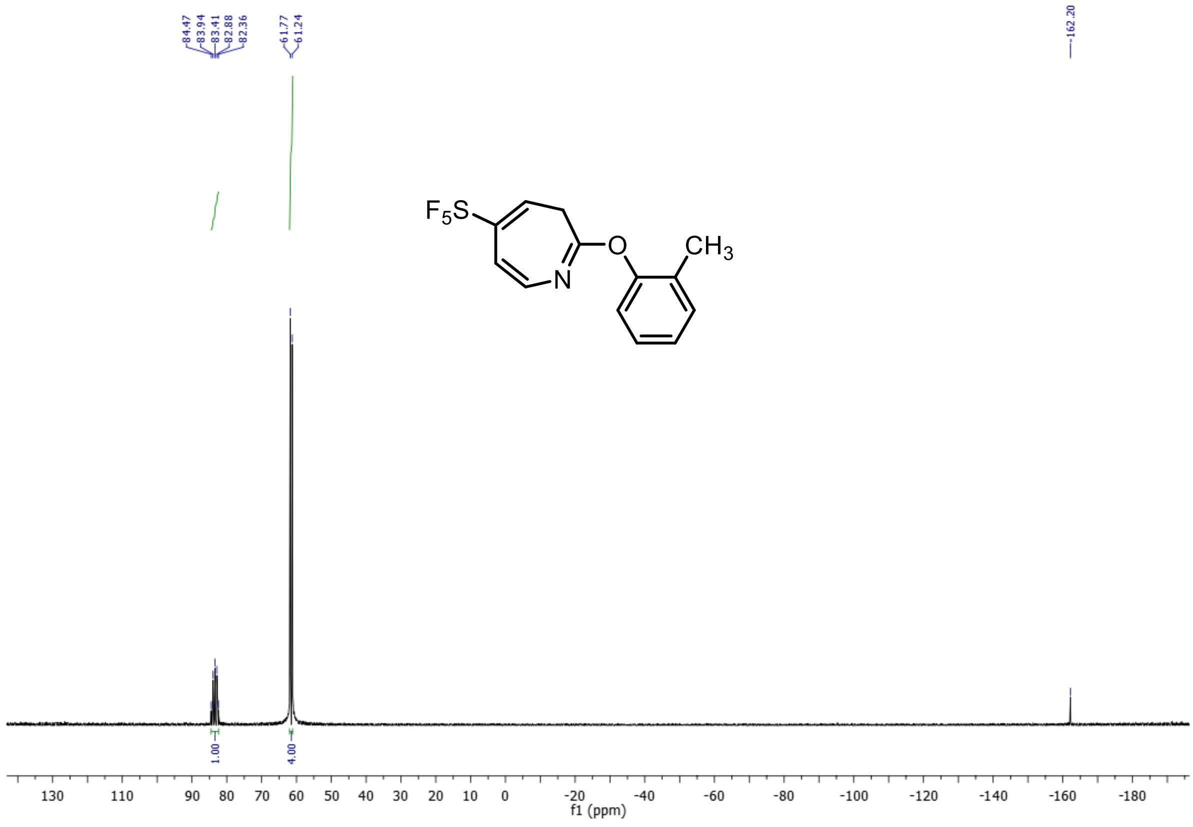

^{1}H NMR (500 MHz, $CDCl_3$) : **3hf**

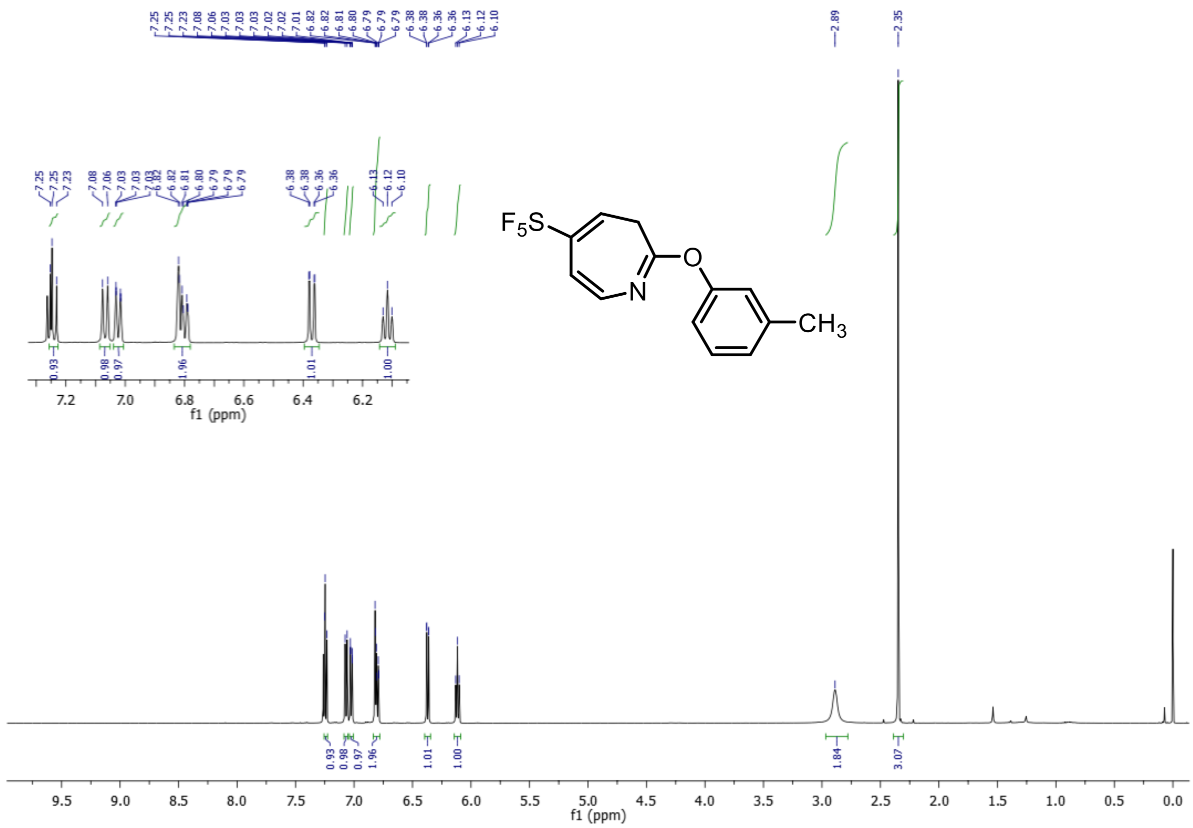

^{13}C NMR (126 MHz, $CDCl_3$) : **3hf**

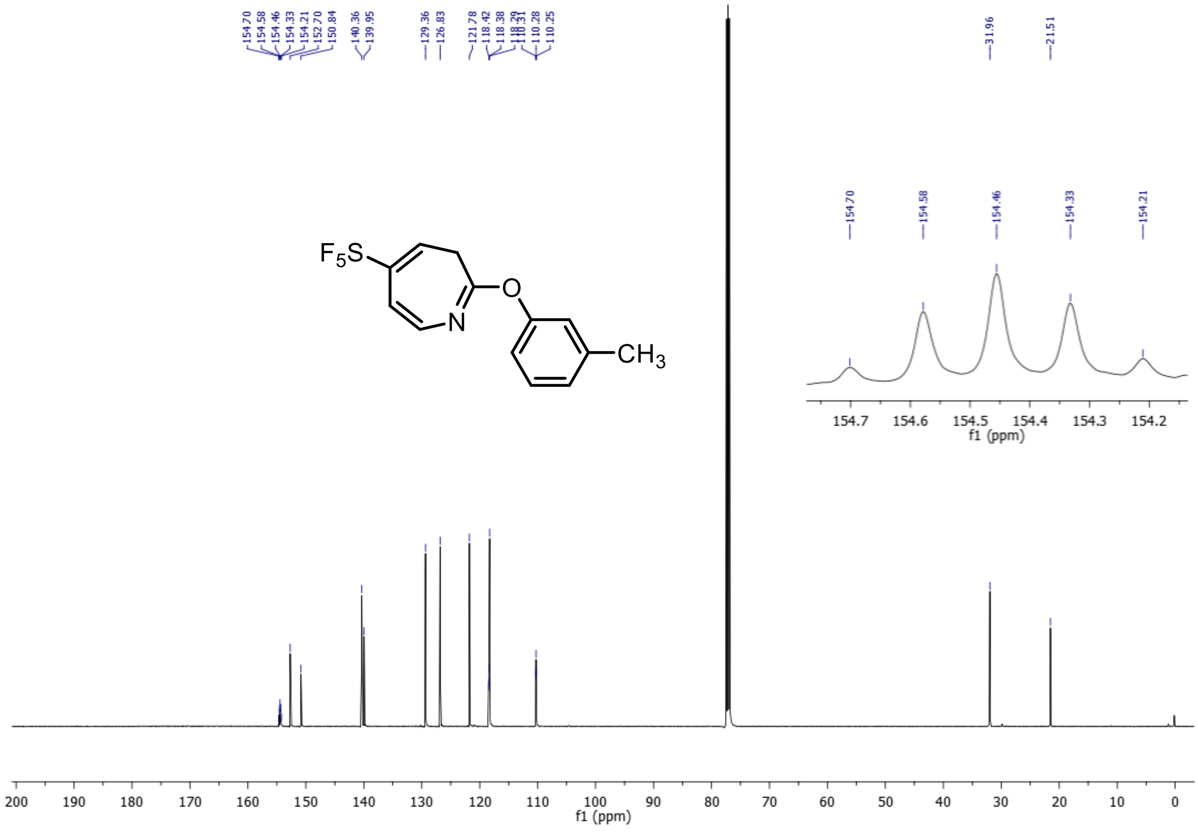

^{19}F NMR (282 MHz, CDCl$_3$) : **3hf**

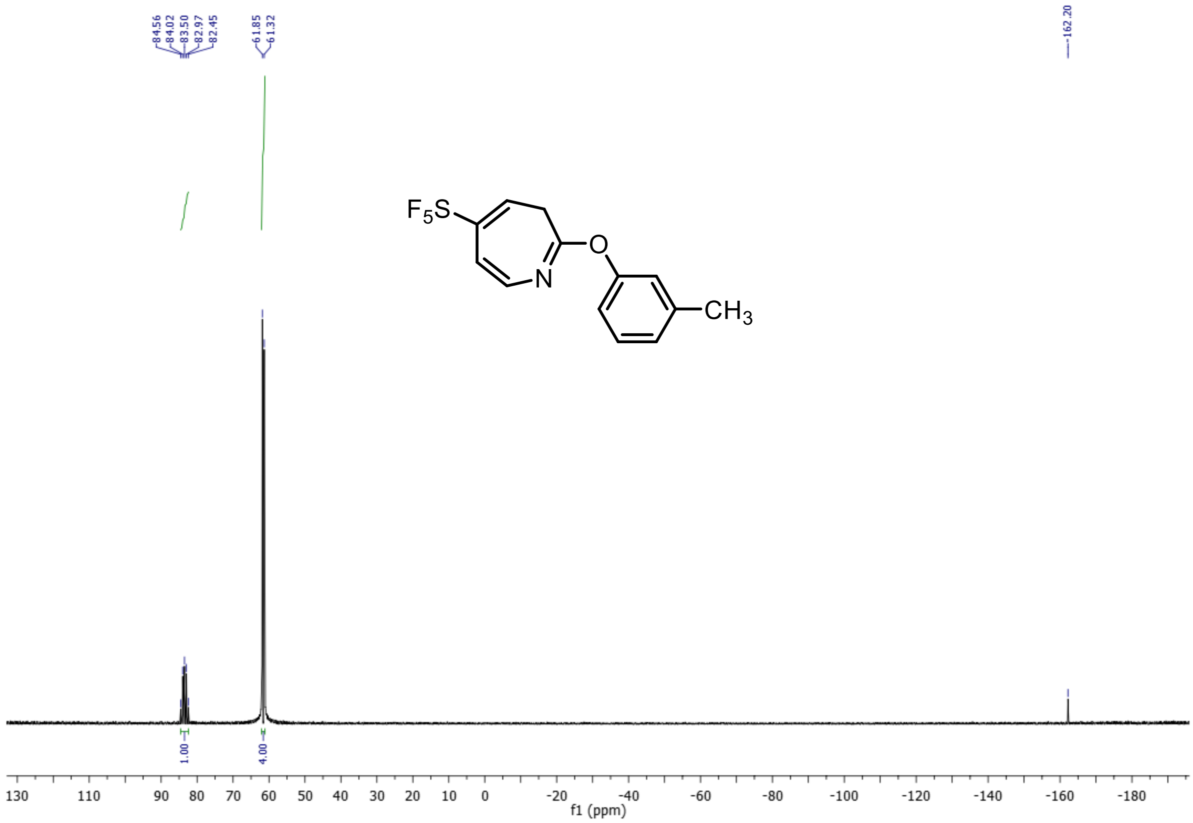

^{1}H NMR (500 MHz, CDCl$_3$) : **3hg**

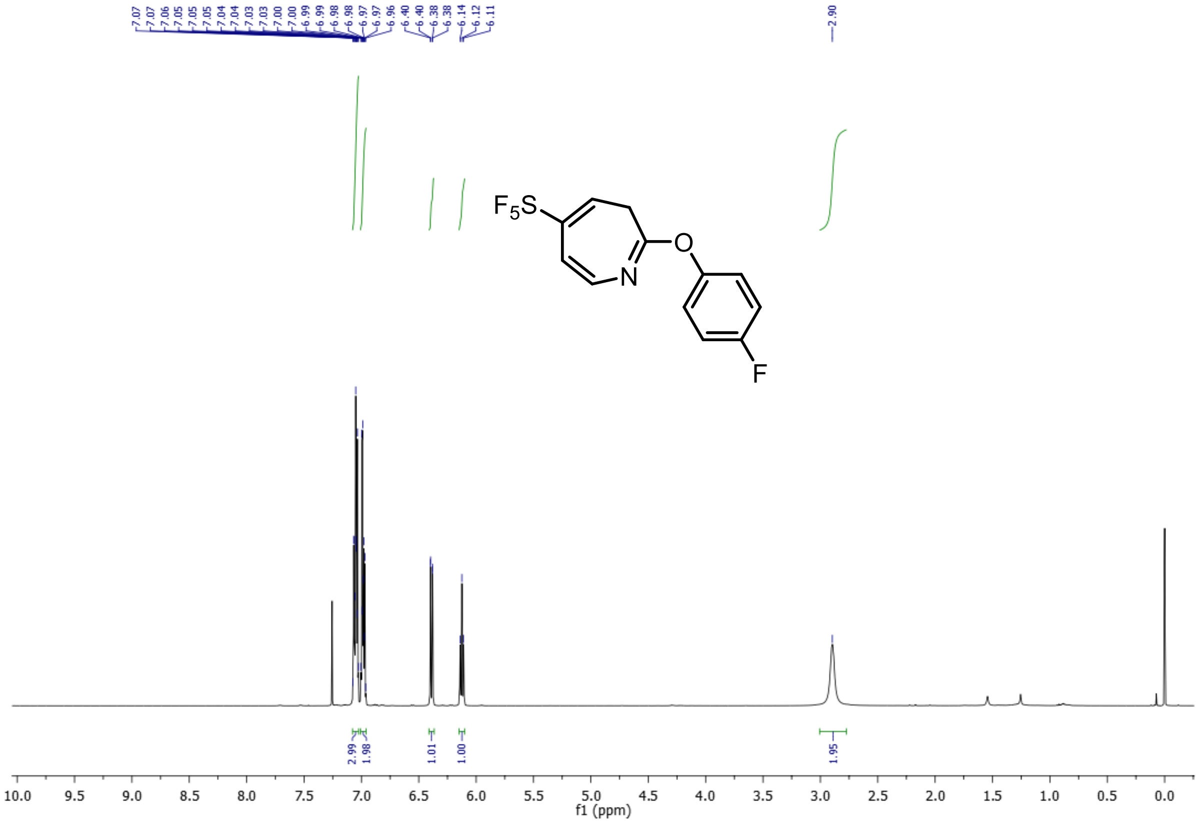

^{13}C NMR (126 MHz, $CDCl_3$) : **3hg**

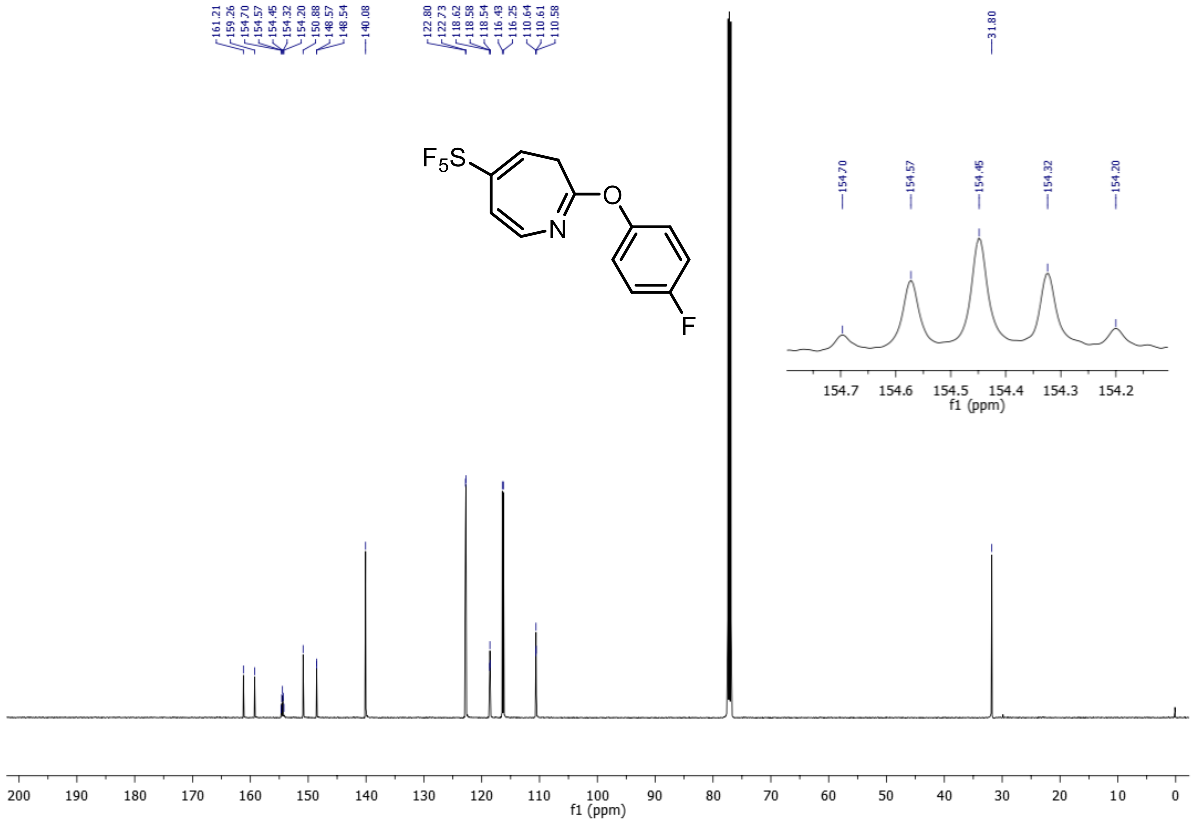

^{19}F NMR (282 MHz, $CDCl_3$) : **3hg**

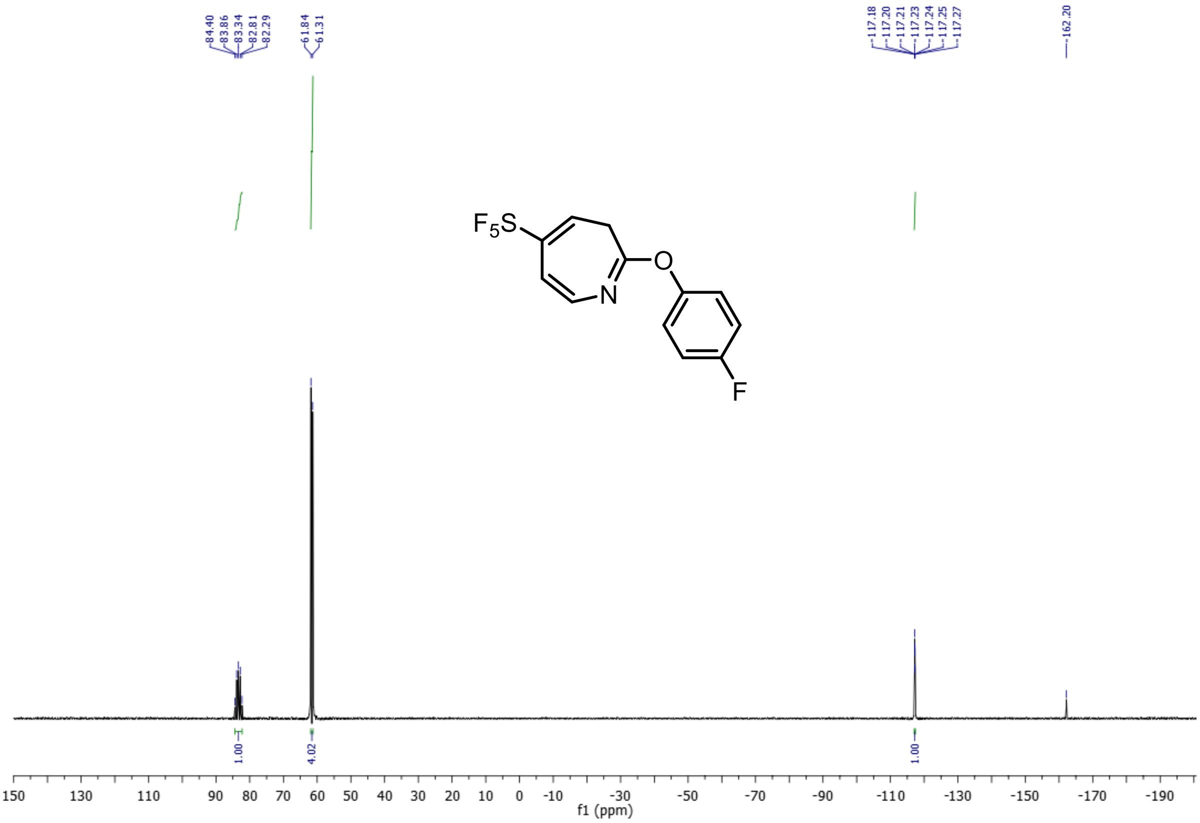

^{1}H NMR (500 MHz, CDCl3) : **3hh**

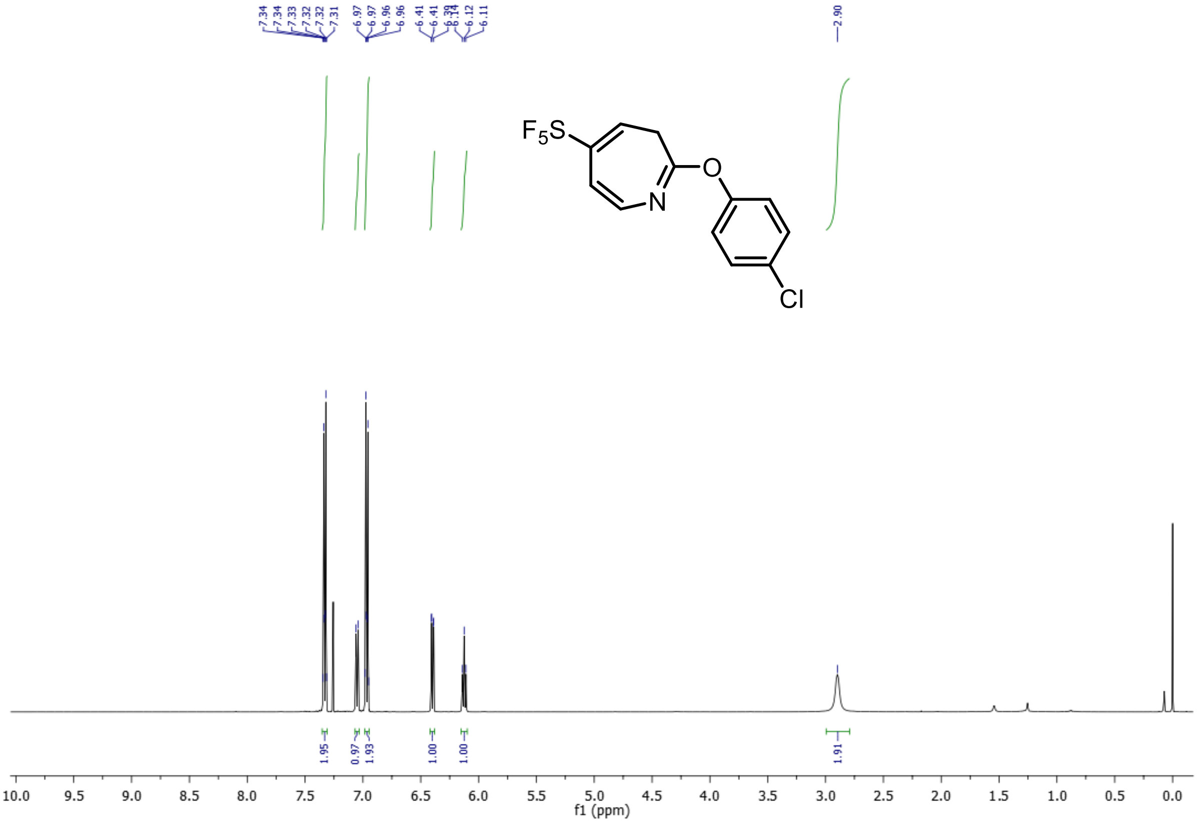

^{13}C NMR (126 MHz, CDCl3) : **3hh**

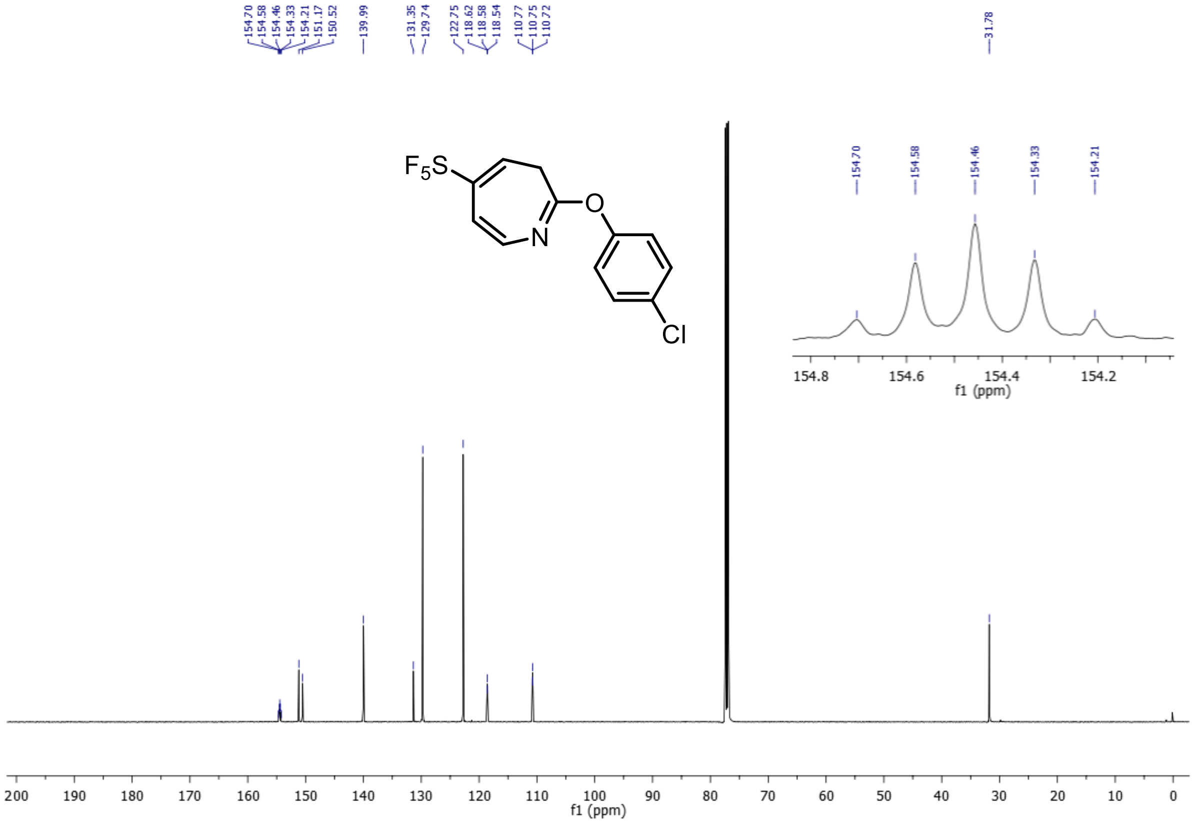

^{19}F NMR (282 MHz, CDCl$_3$) : **3hh**

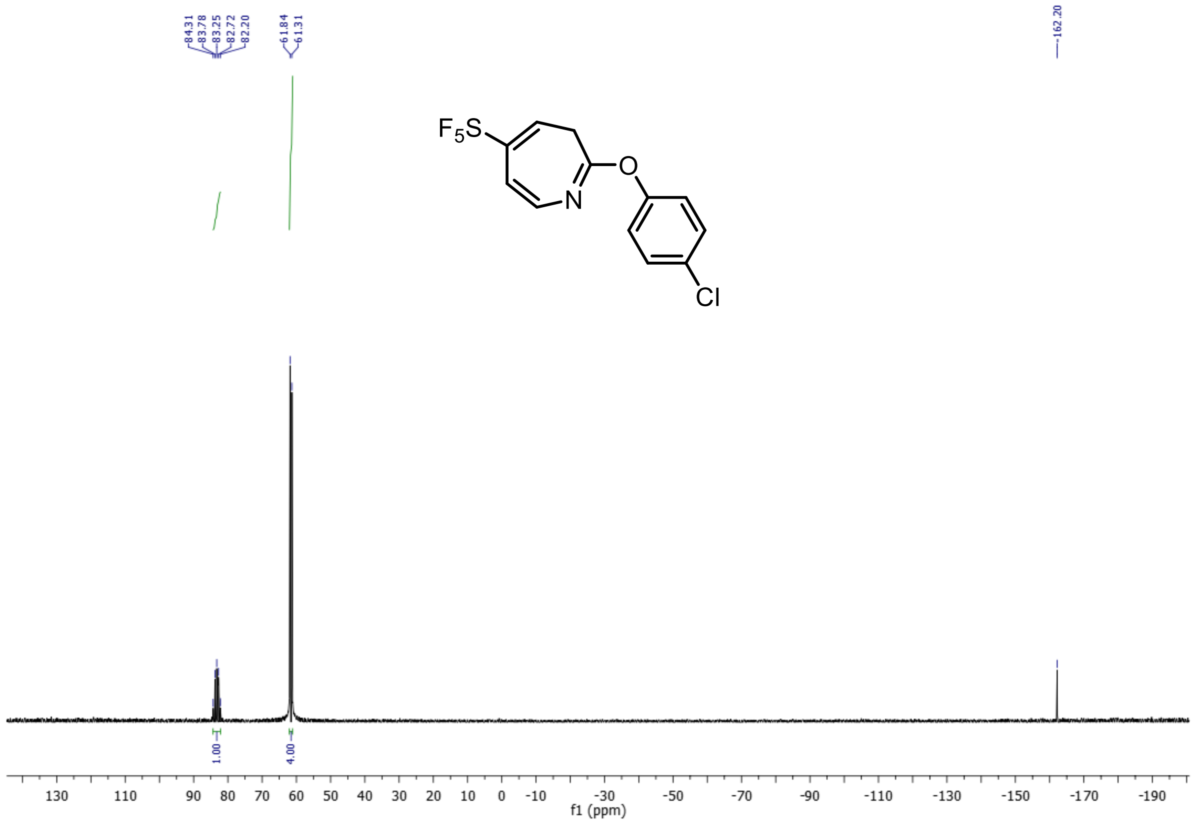

^{1}H NMR (500 MHz, CDCl$_3$) : **3hi**

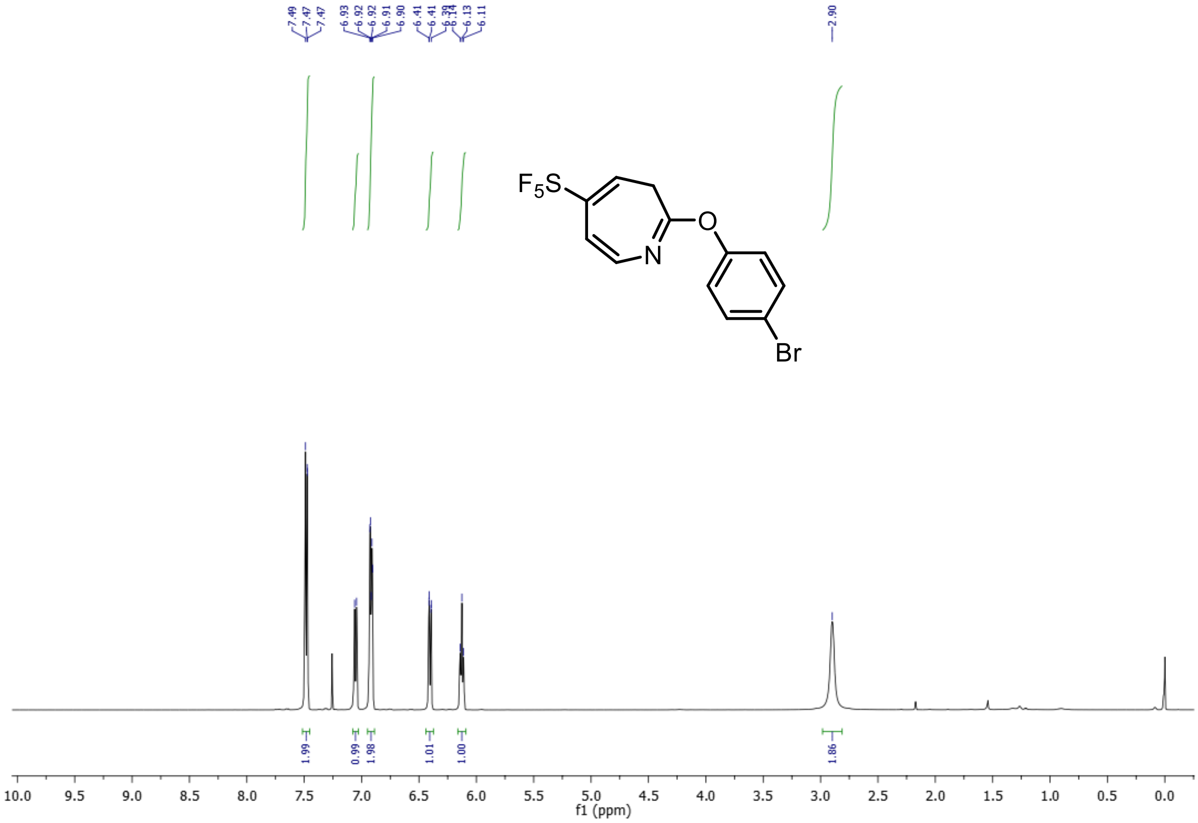

^{13}C NMR (126 MHz, $CDCl_3$) : **3hi**

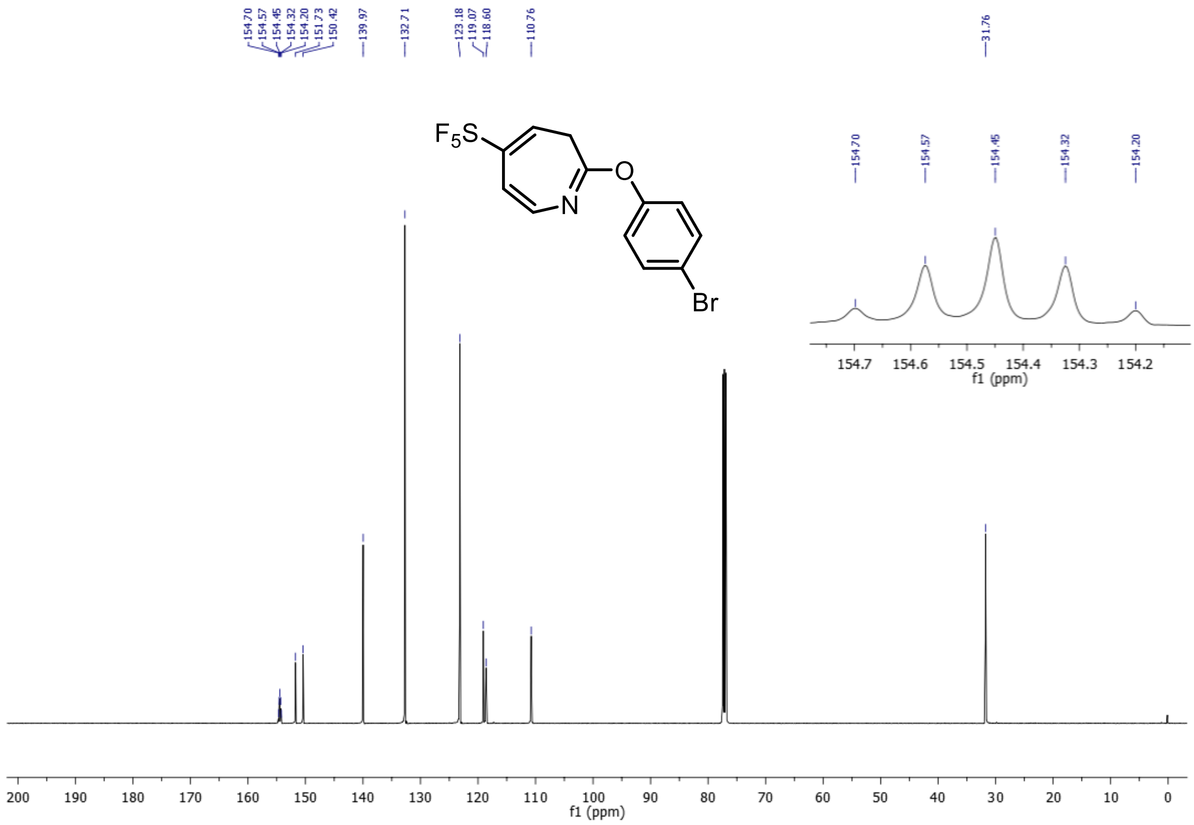

^{19}F NMR (282 MHz, $CDCl_3$) : **3hi**

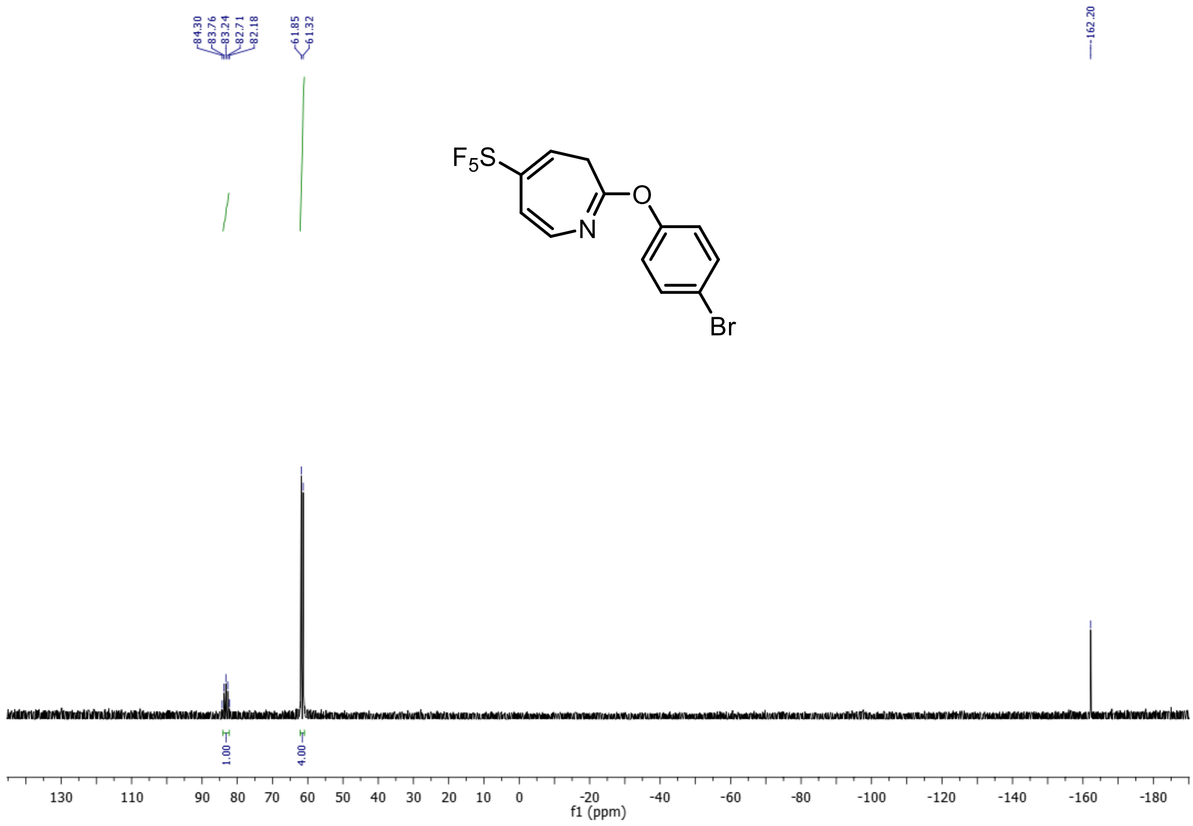

^{1}H NMR (500 MHz, $CDCl_3$) : **3hj**

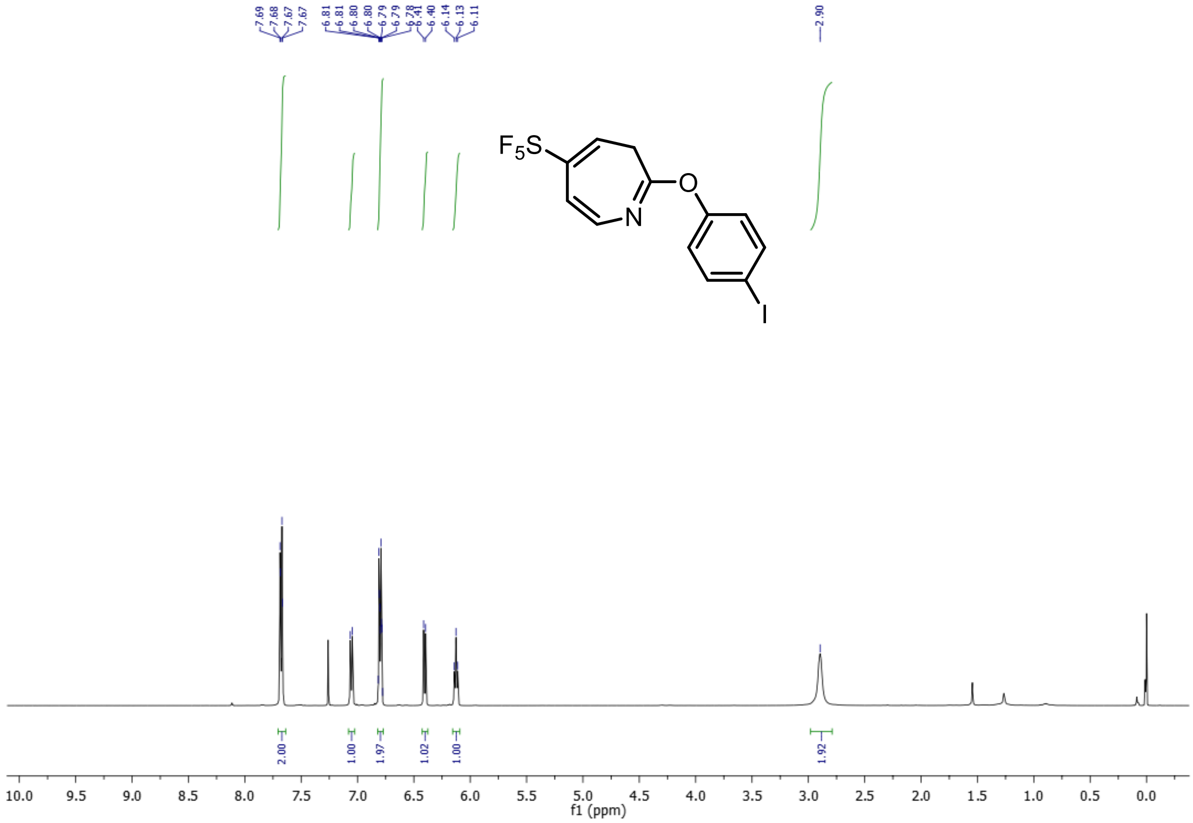

^{13}C NMR (126 MHz, $CDCl_3$) : **3hj**

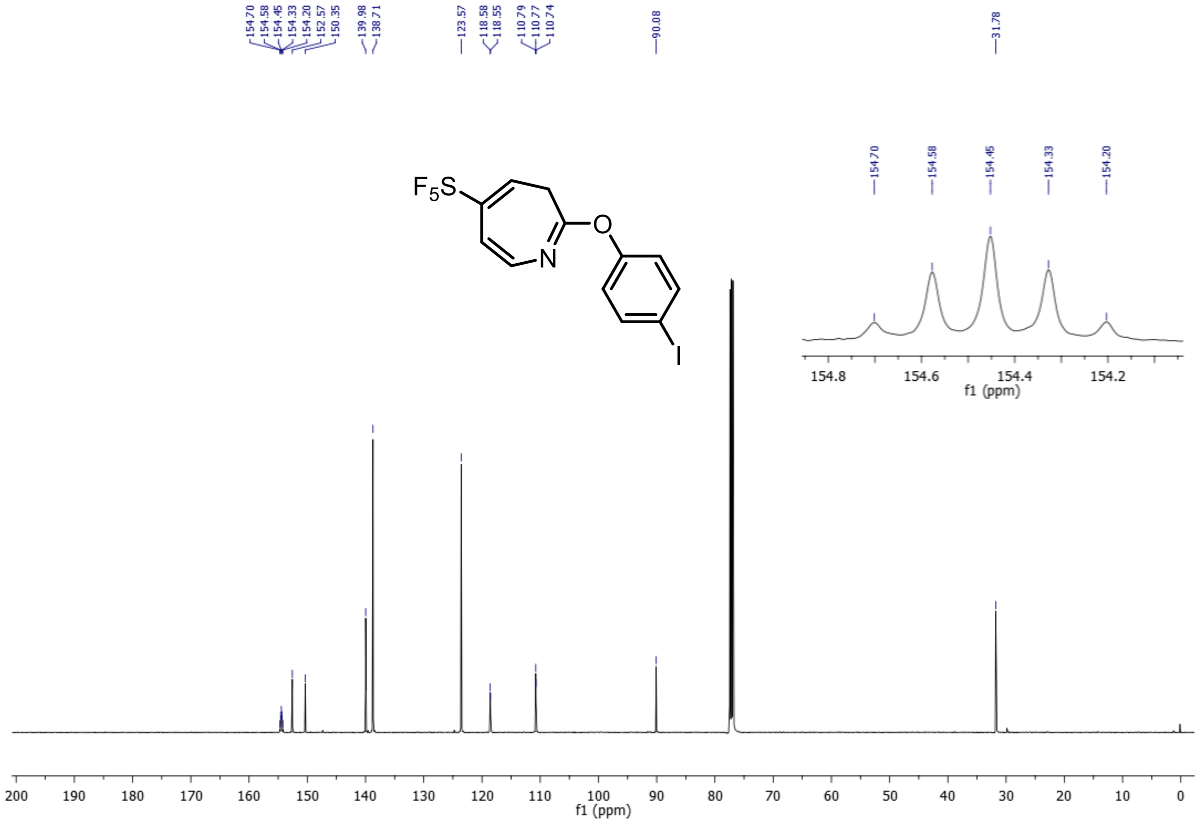

^{19}F NMR (282 MHz, $CDCl_3$) : **3hj**

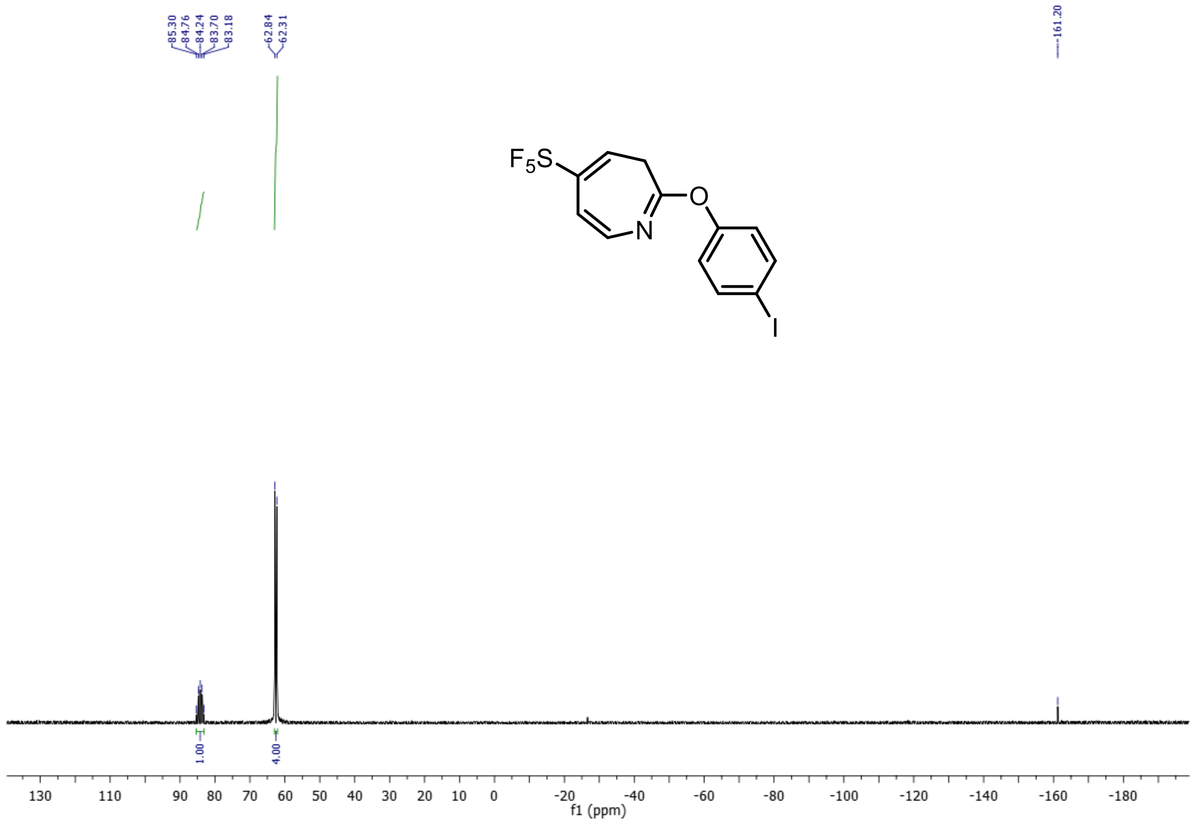

^{1}H NMR (500 MHz, $CDCl_3$) : **3hk**

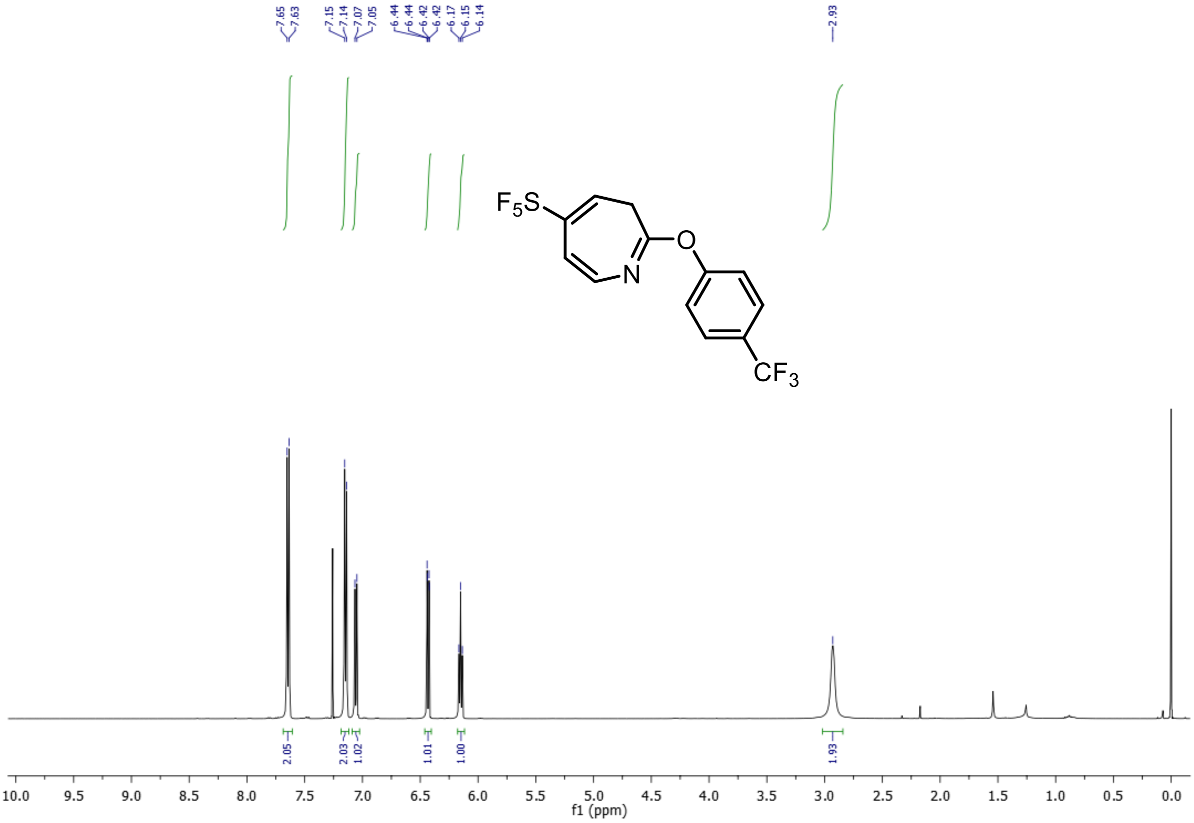

^{13}C NMR (126 MHz, $CDCl_3$) : **3hk**

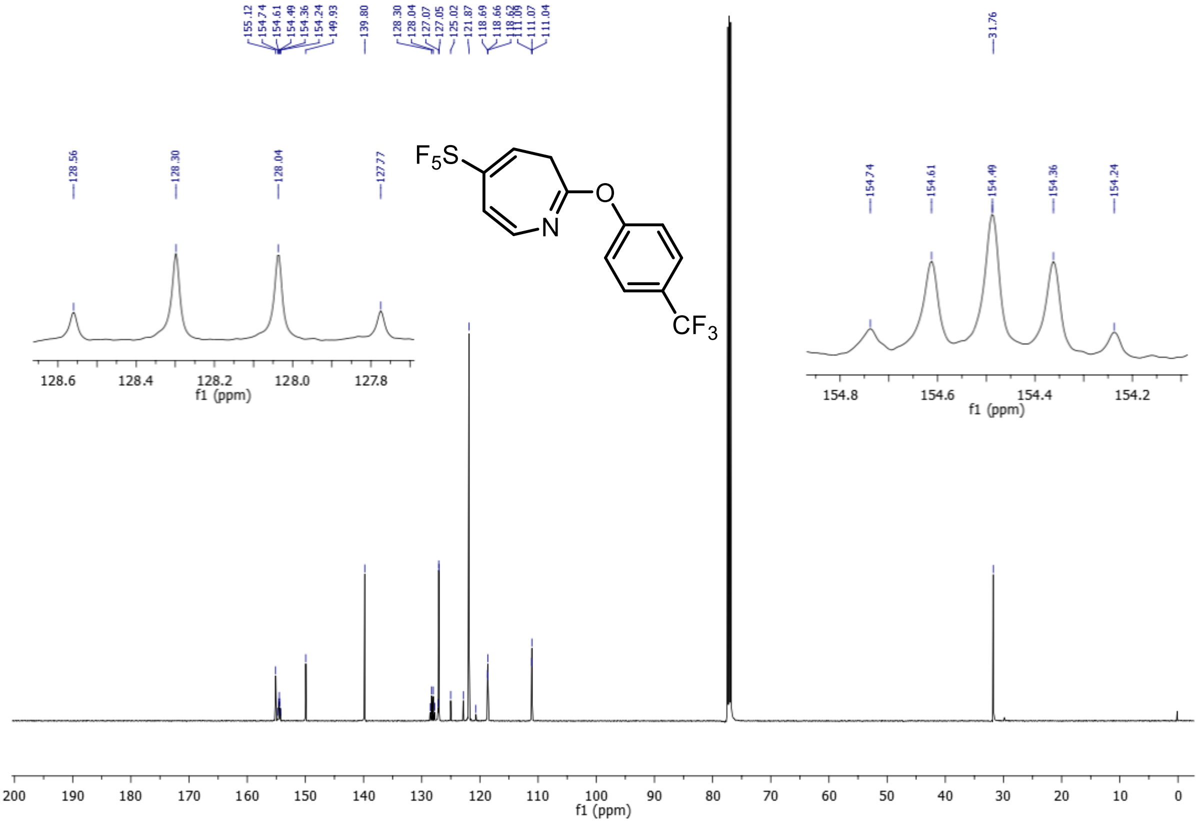

^{19}F NMR (282 MHz, $CDCl_3$) : **3hk**

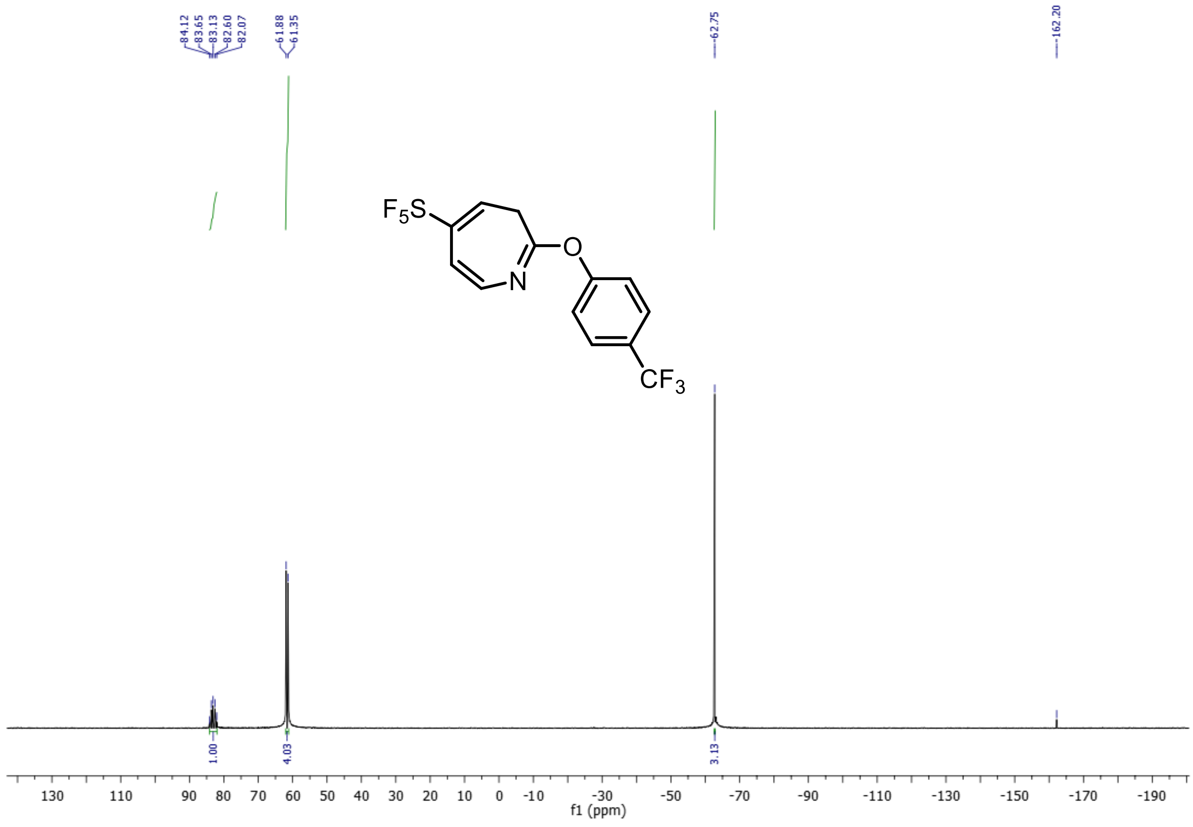

^{1}H NMR (500 MHz, $CDCl_3$) : **3hl**

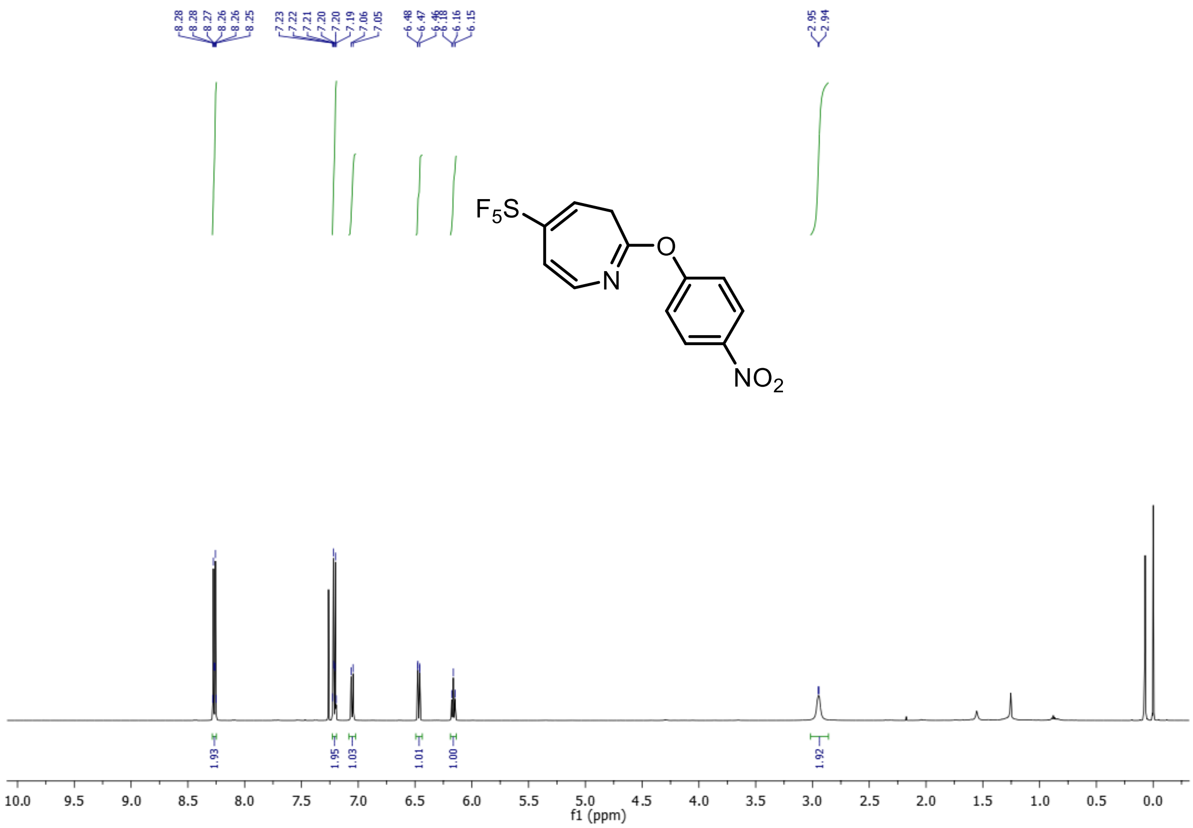

^{13}C NMR (126 MHz, $CDCl_3$) : **3hl**

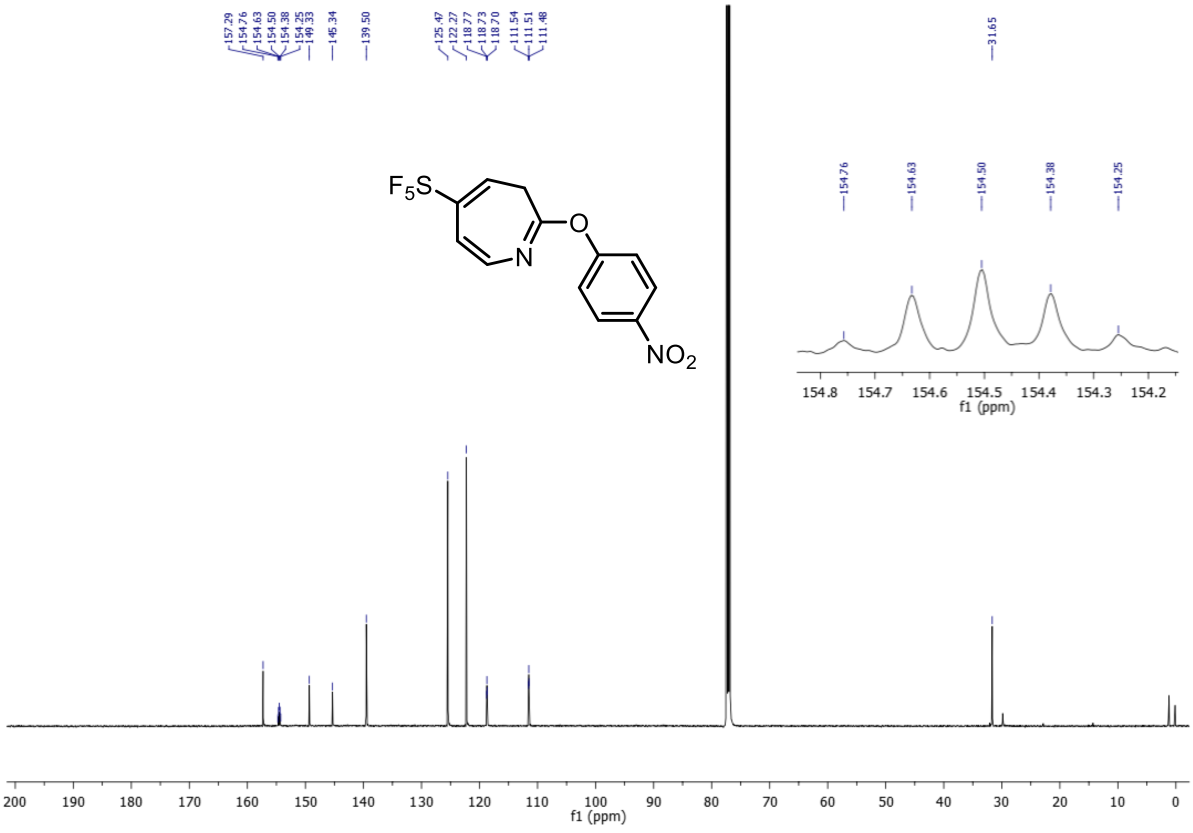

^{19}F NMR (282 MHz, $CDCl_3$) : **3hl**

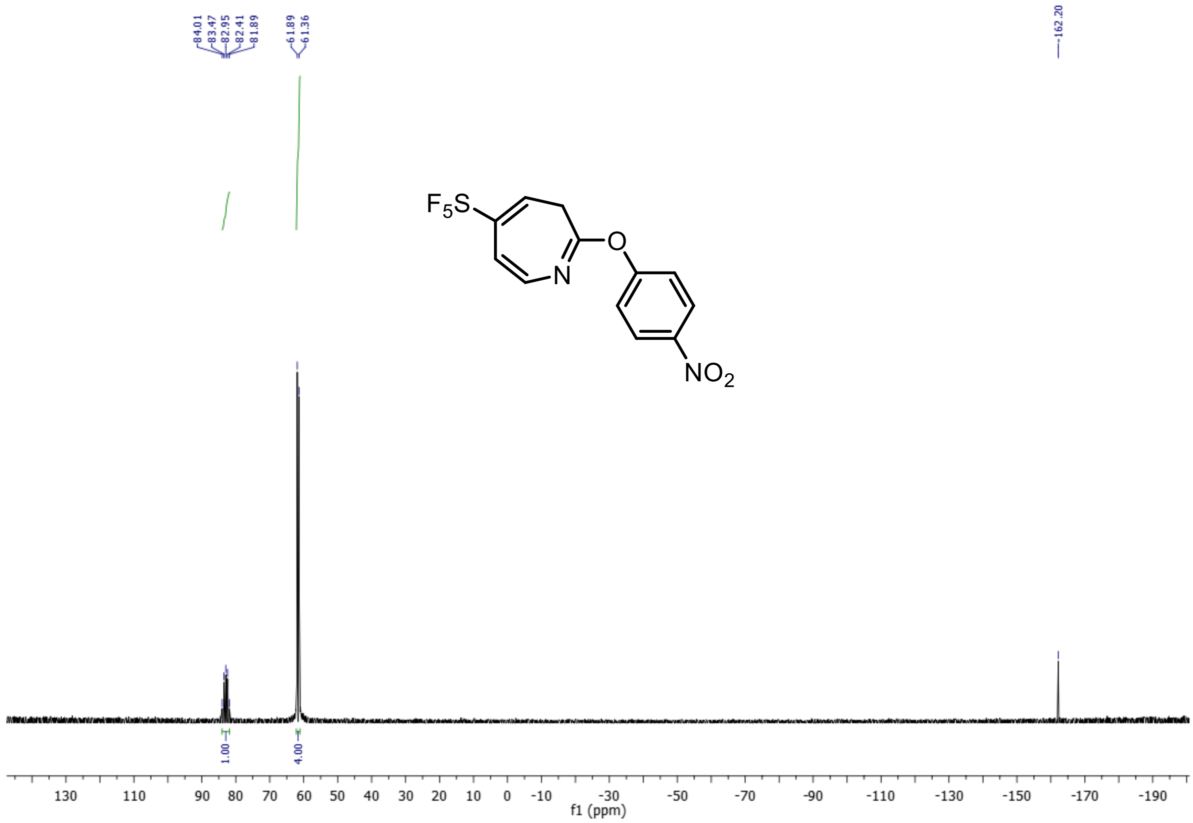

^{1}H NMR (500 MHz, $CDCl_3$) : **3hm**

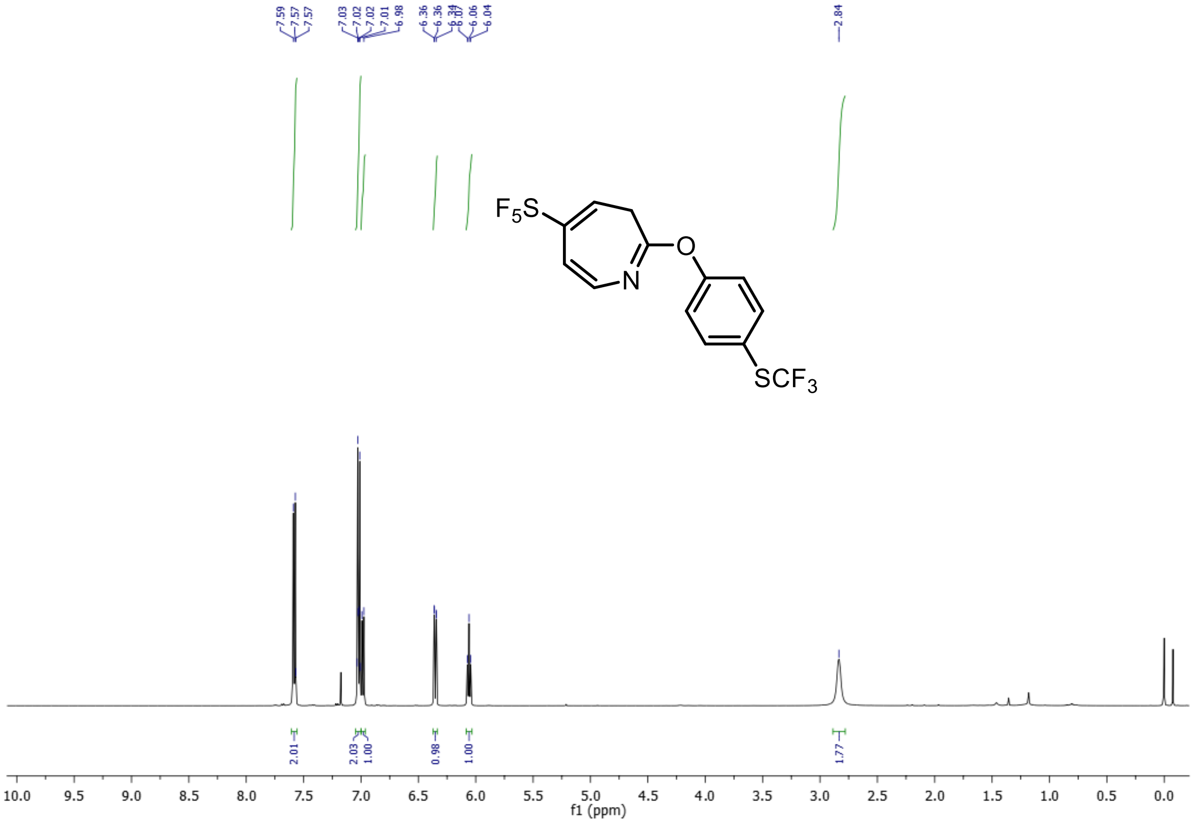

^{13}C NMR (126 MHz, $CDCl_3$) : **3hm**

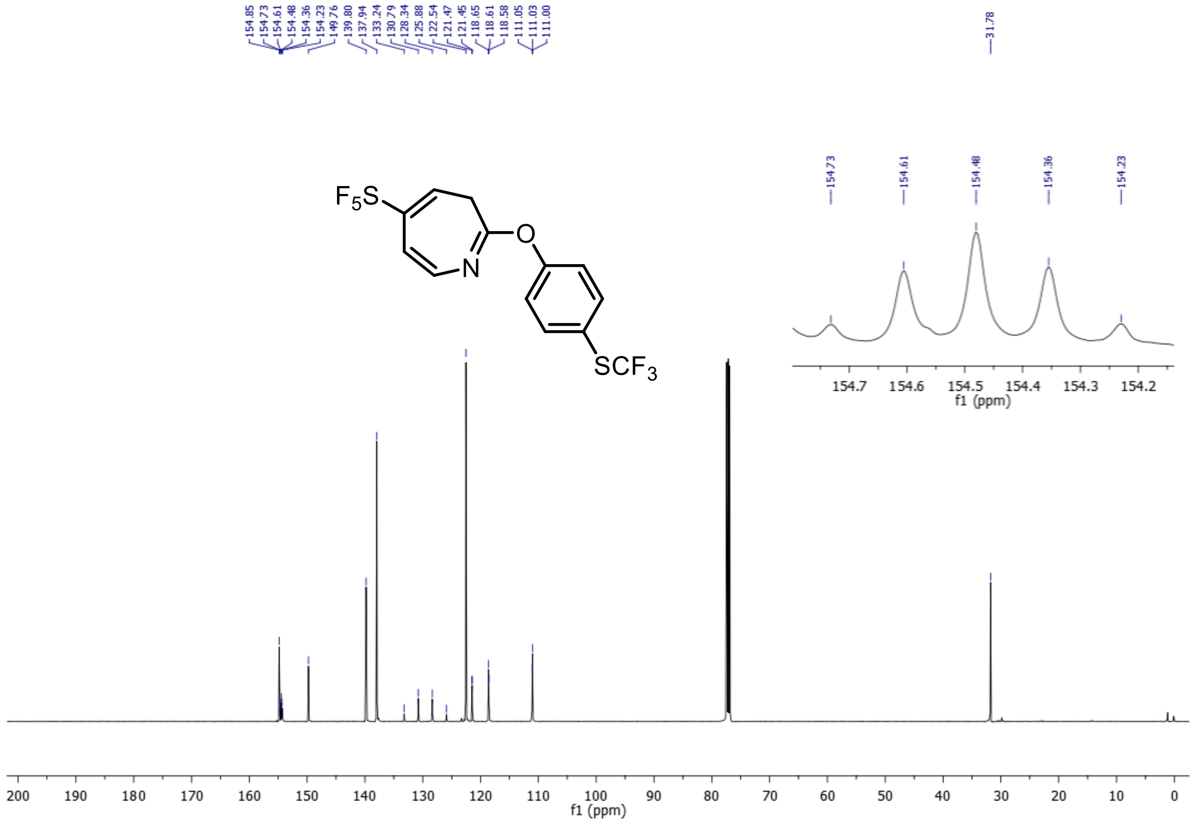

^{19}F NMR (282 MHz, $CDCl_3$) : **3hm**

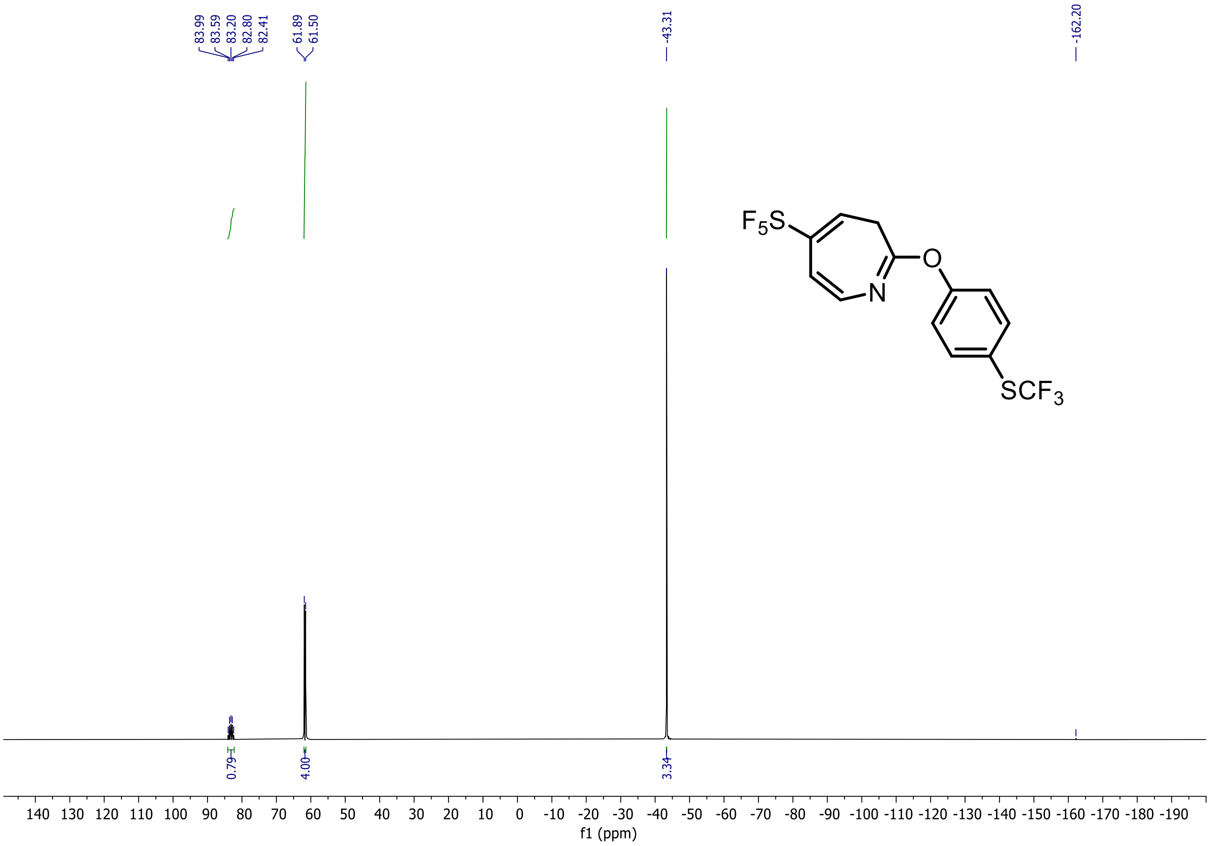

^{1}H NMR (500 MHz, $CDCl_3$) : **3hn**

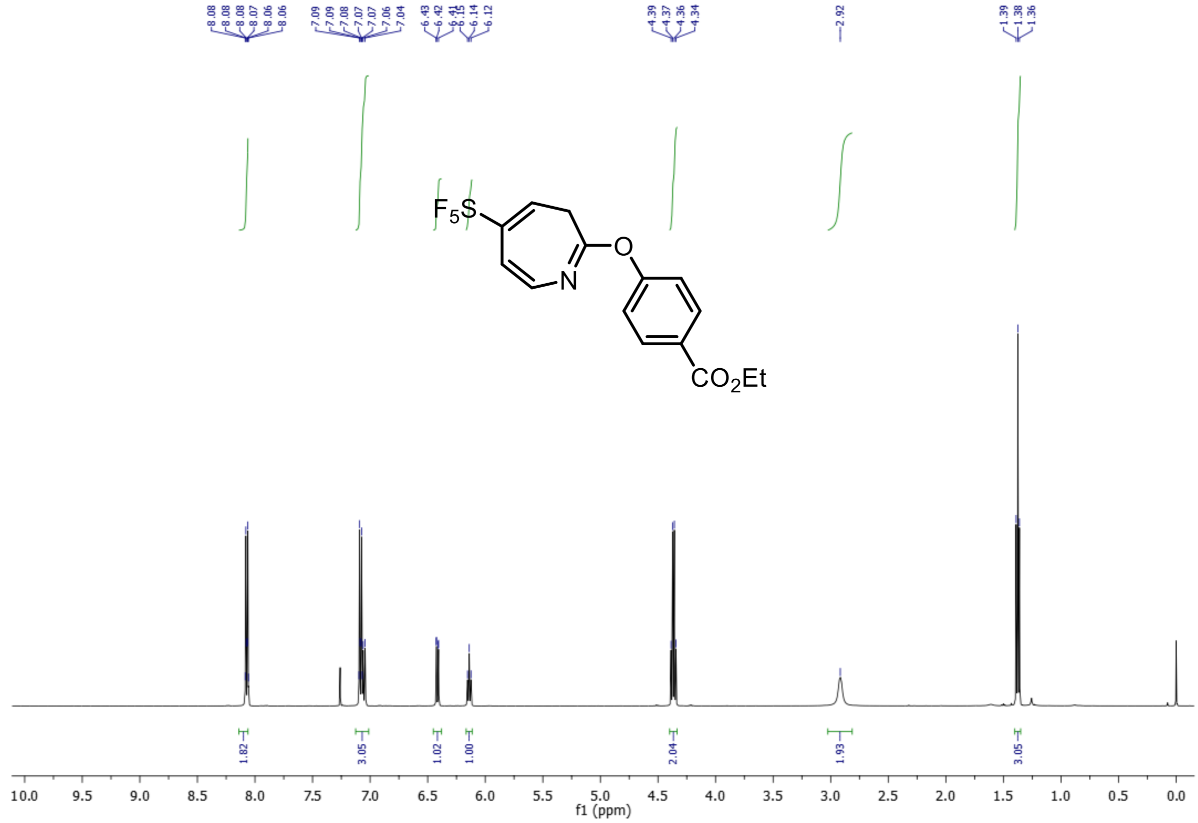

^{13}C NMR (126 MHz, $CDCl_3$) : **3hn**

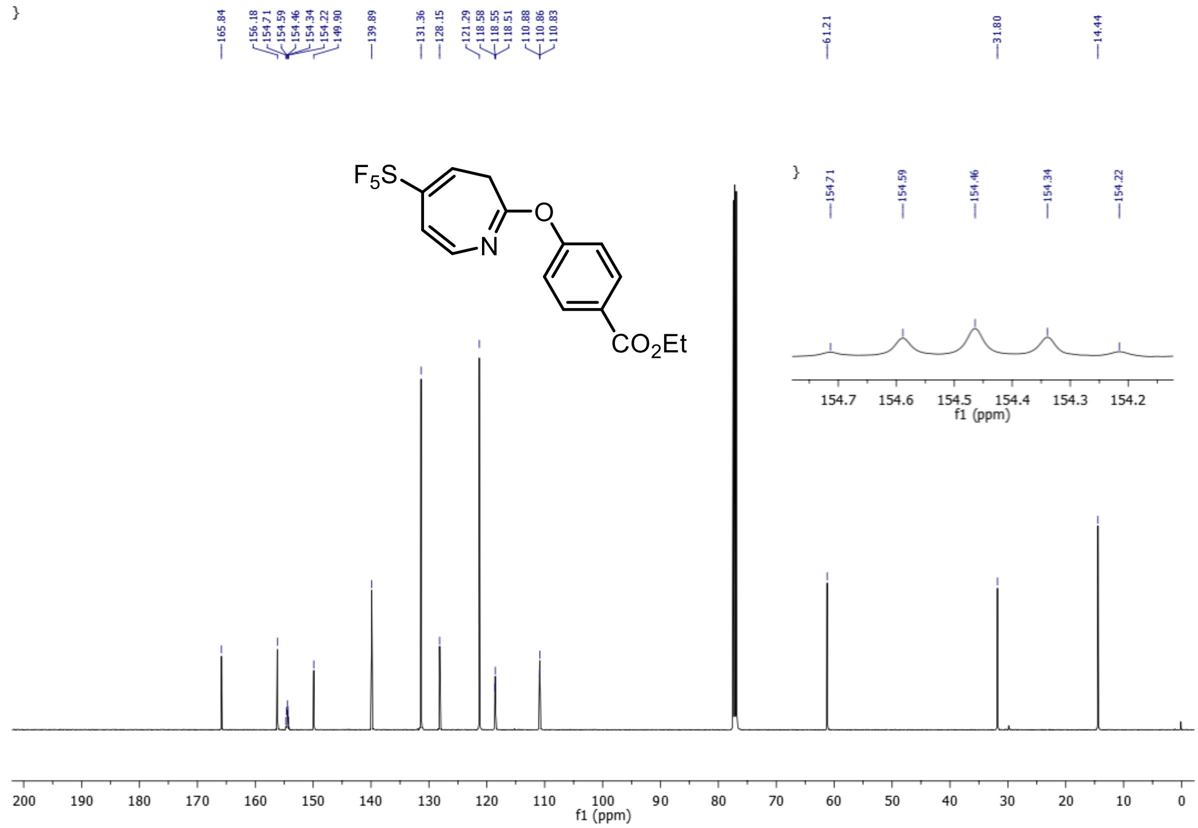

^{19}F NMR (282 MHz, $CDCl_3$) : **3hn**

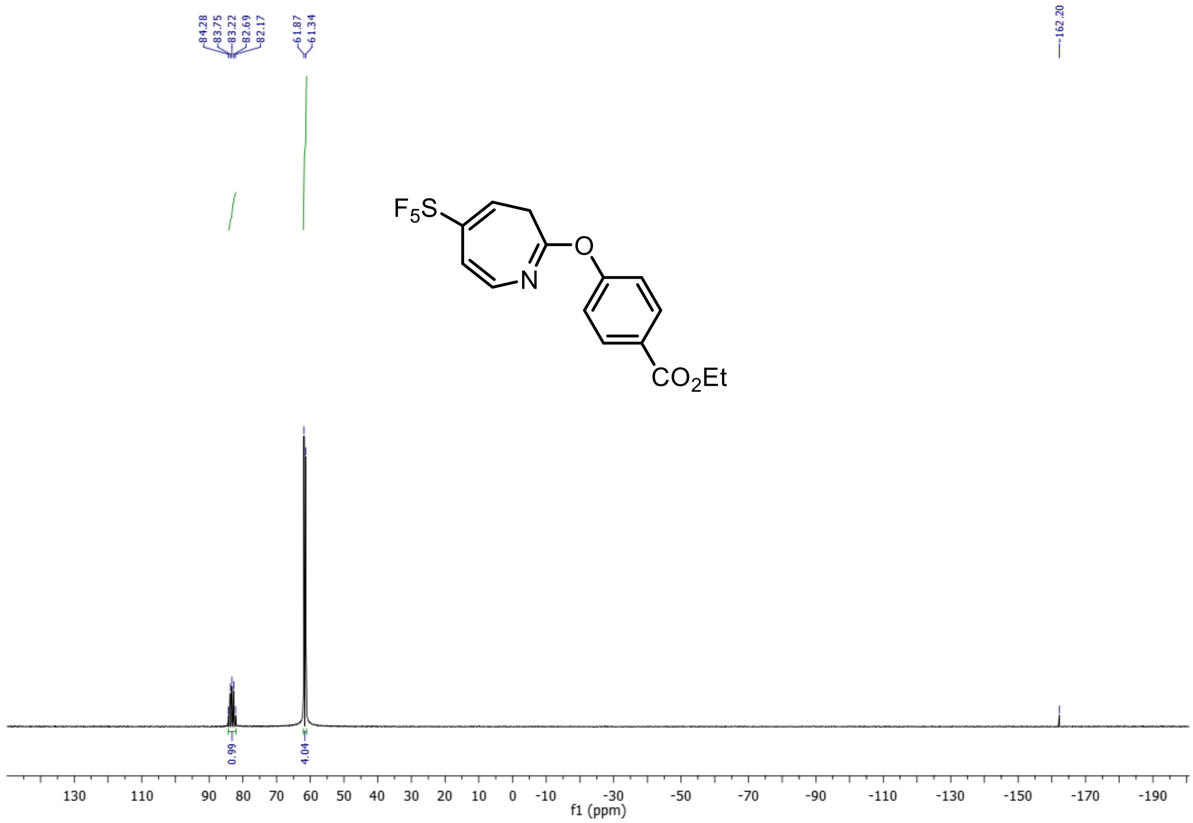

^{1}H NMR (500 MHz, $CDCl_3$) : **3ho**

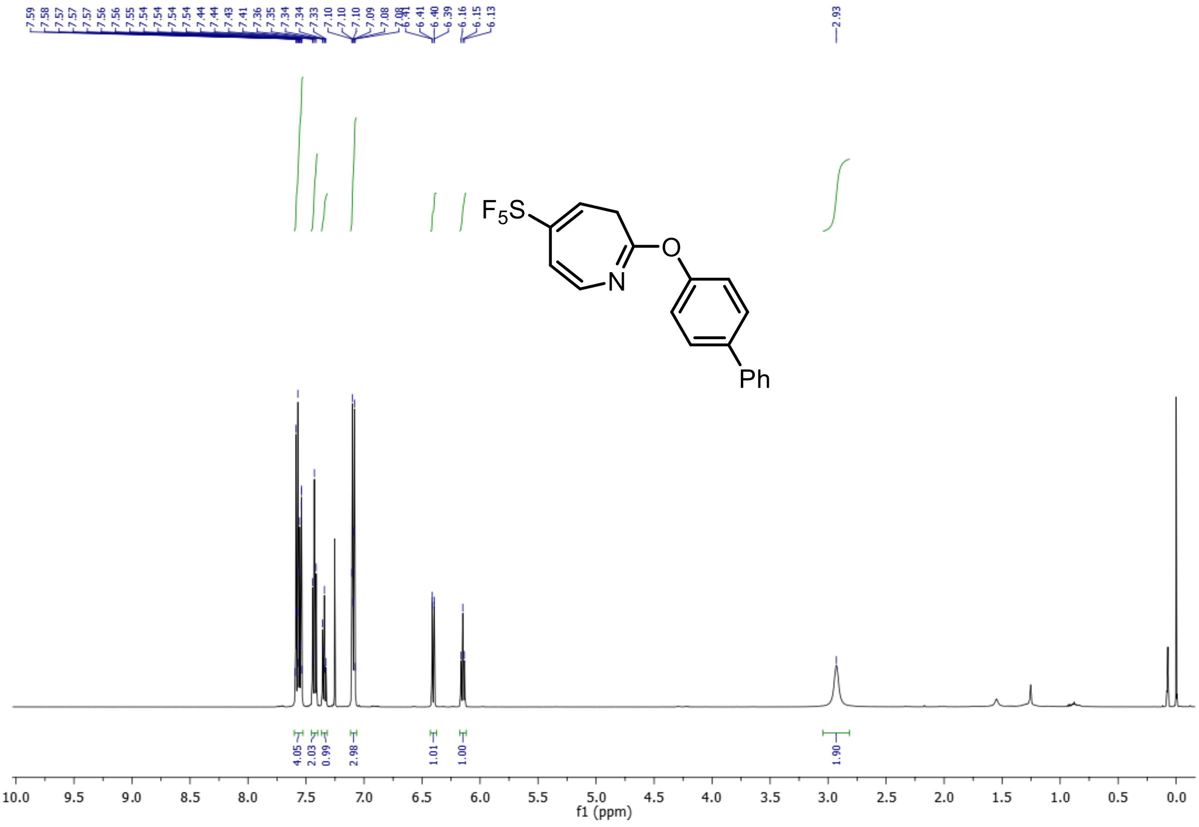

^{13}**C NMR** (126 MHz, $CDCl_3$) : **3ho**

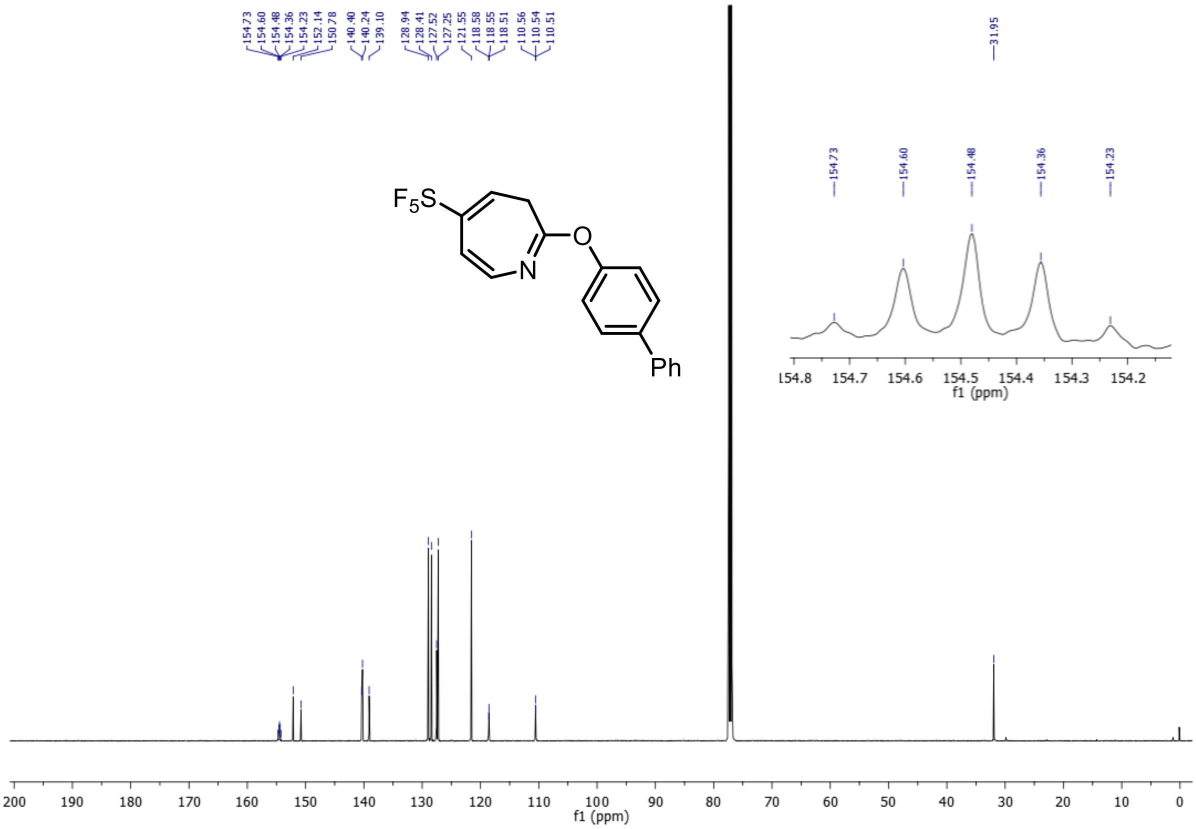

^{19}**F NMR** (282 MHz, $CDCl_3$) : **3ho**

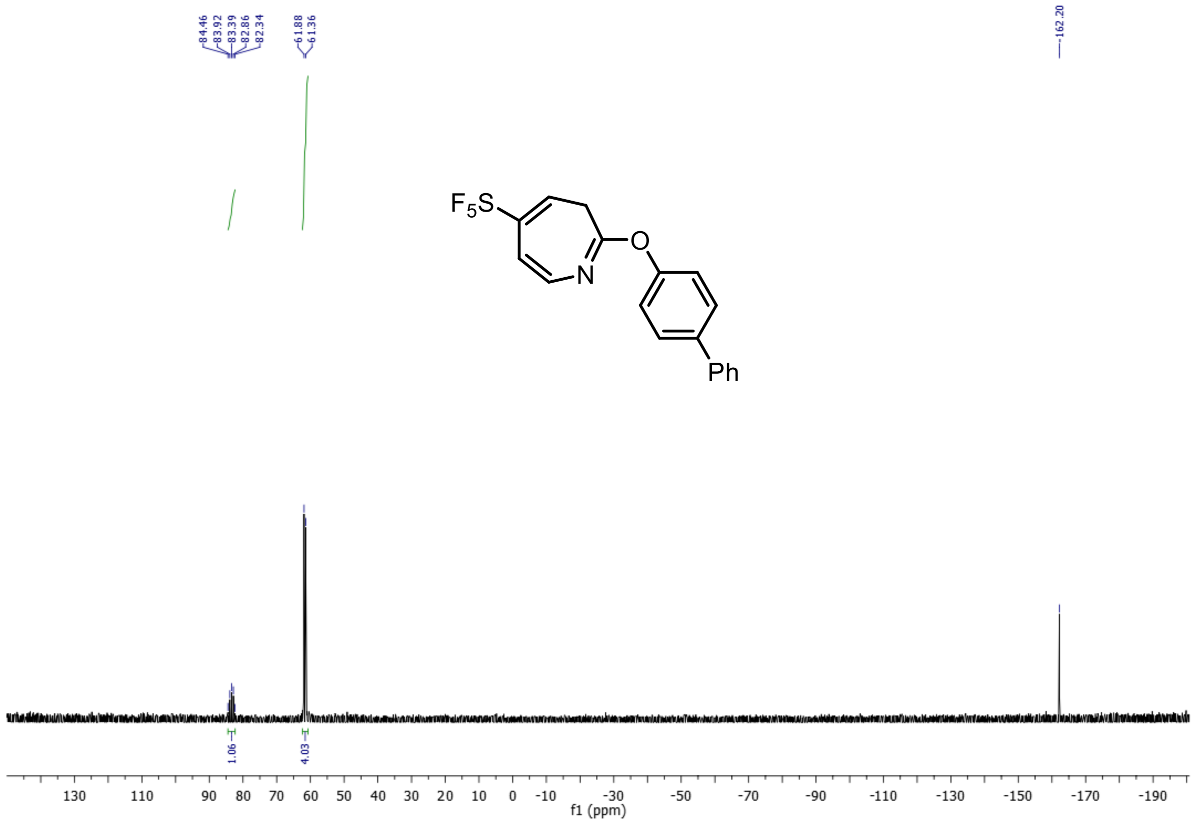

^{1}H NMR (500 MHz, $CDCl_3$) : **3hp**

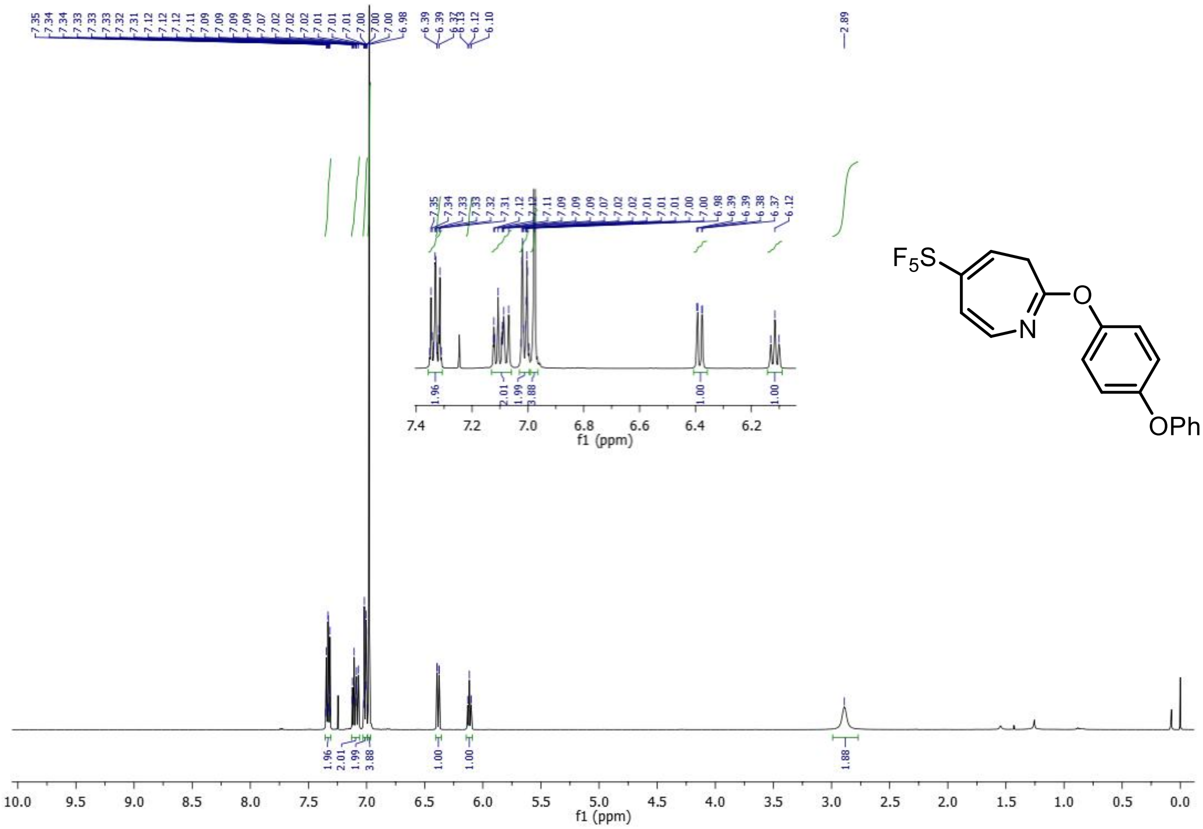

^{13}C NMR (126 MHz, $CDCl_3$) : **3hp**

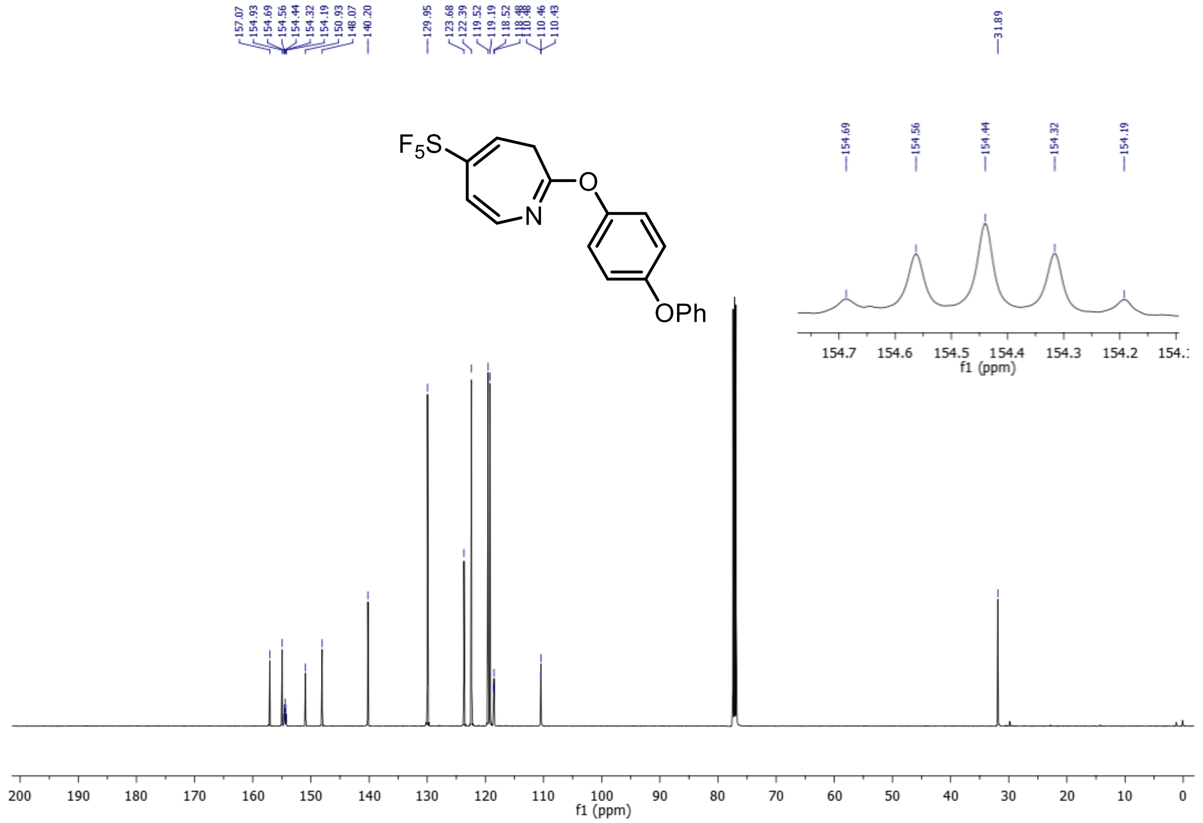

^{19}F NMR (282 MHz, $CDCl_3$) : **3hp**

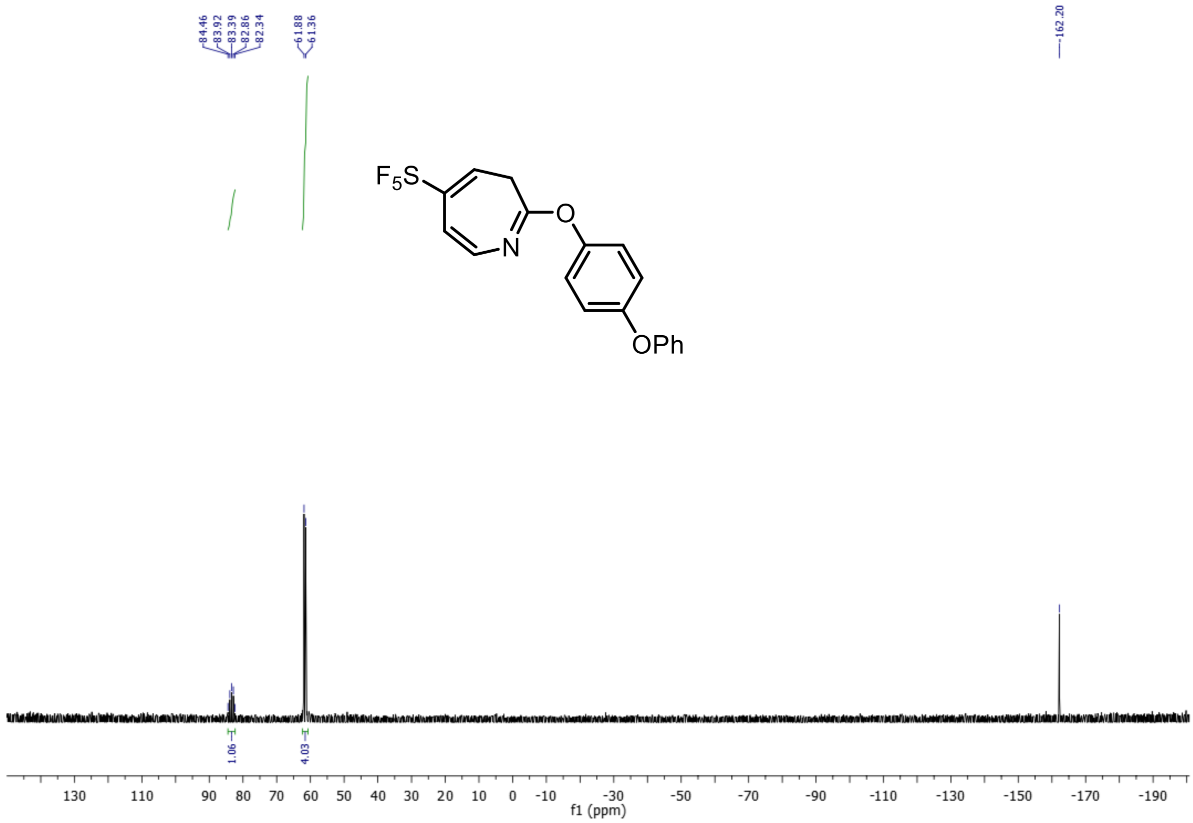

^{1}H NMR (500 MHz, $CDCl_3$) : **3hq**

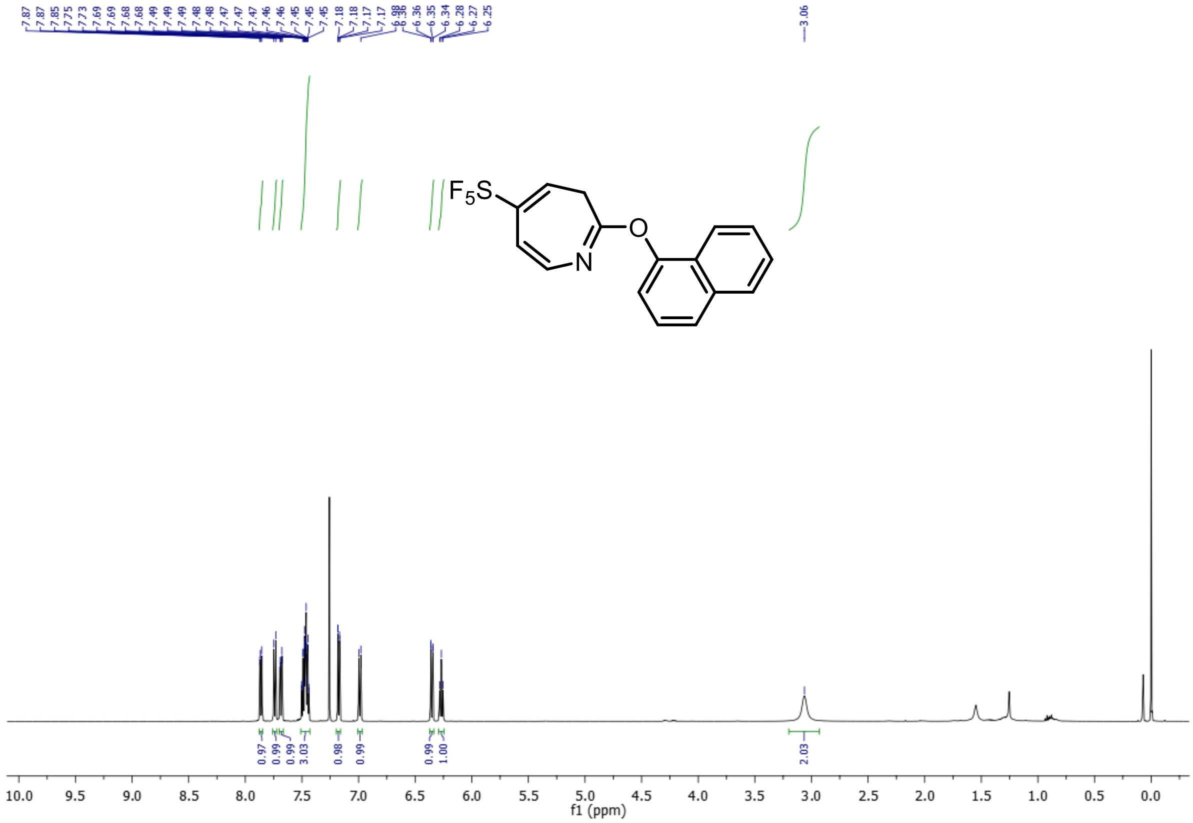

^{13}C NMR (126 MHz, $CDCl_3$) : **3hq**

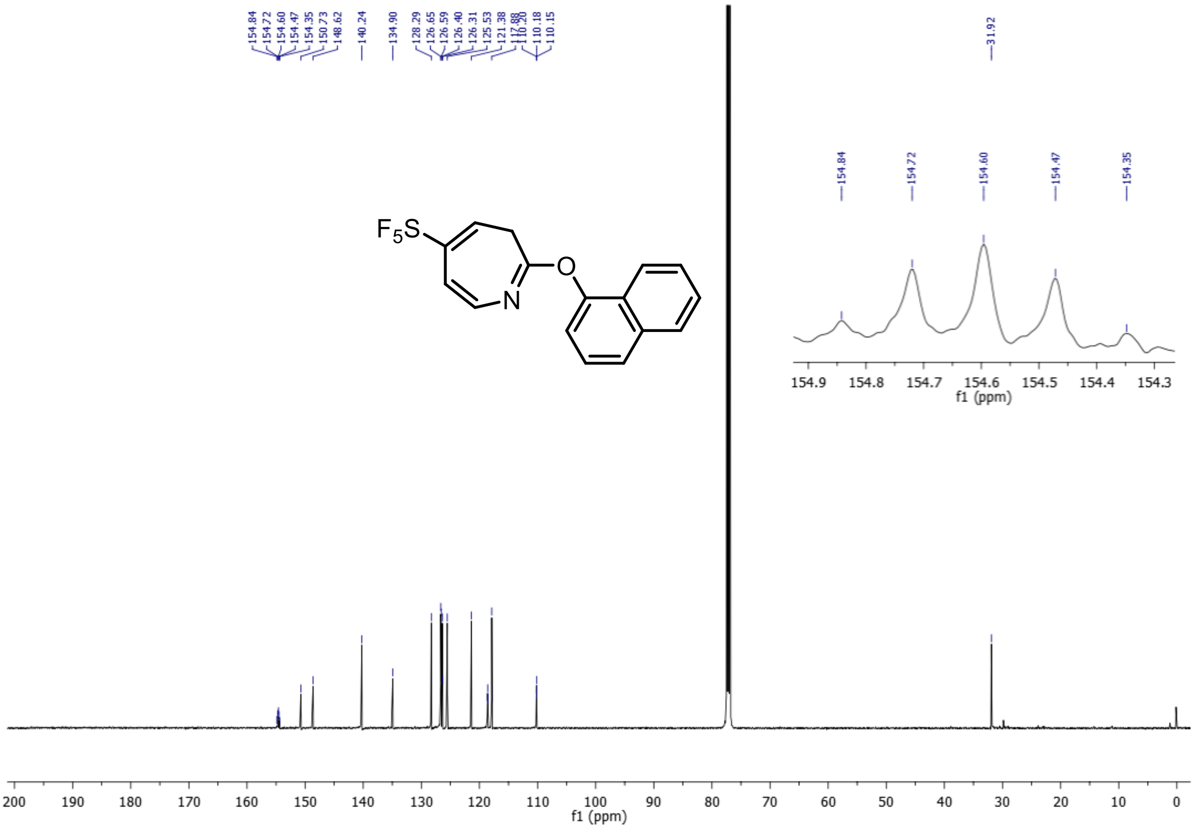

^{19}F NMR (282 MHz, $CDCl_3$) : **3hq**

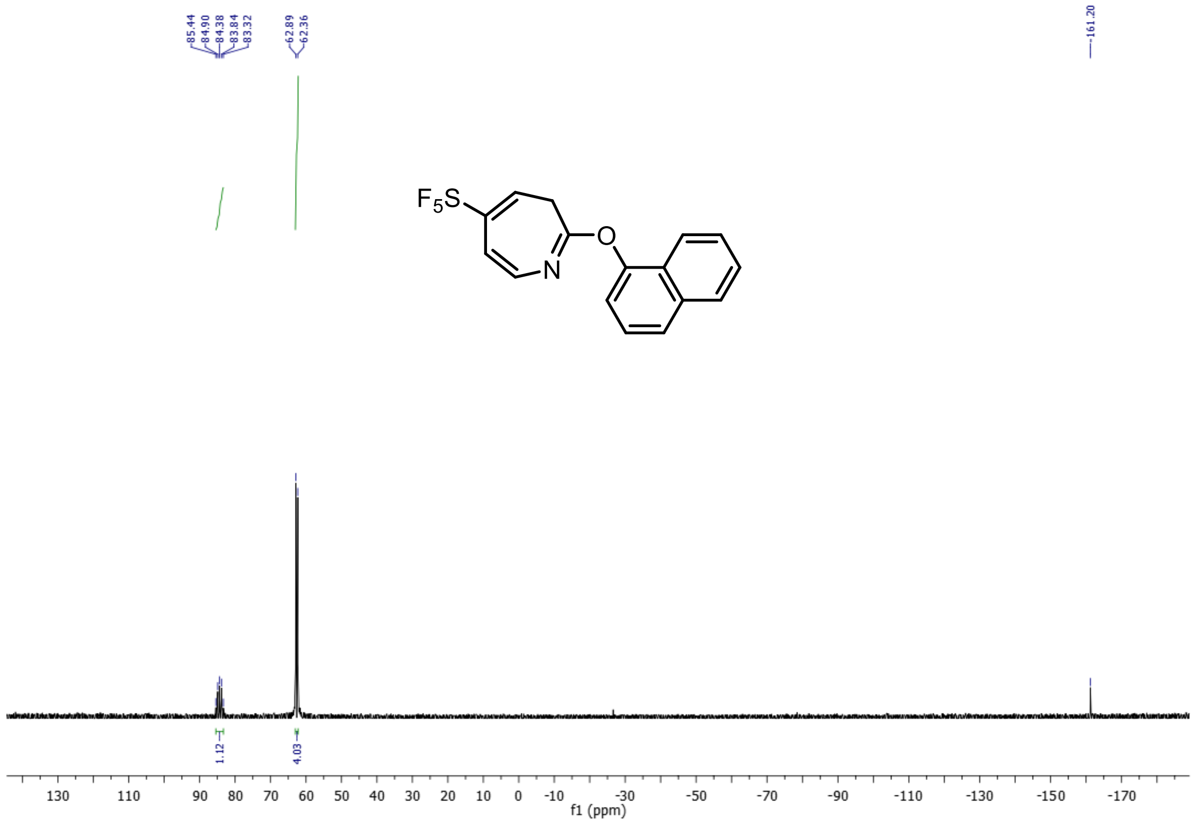

^{1}H NMR (500 MHz, $CDCl_3$) : **3hr**

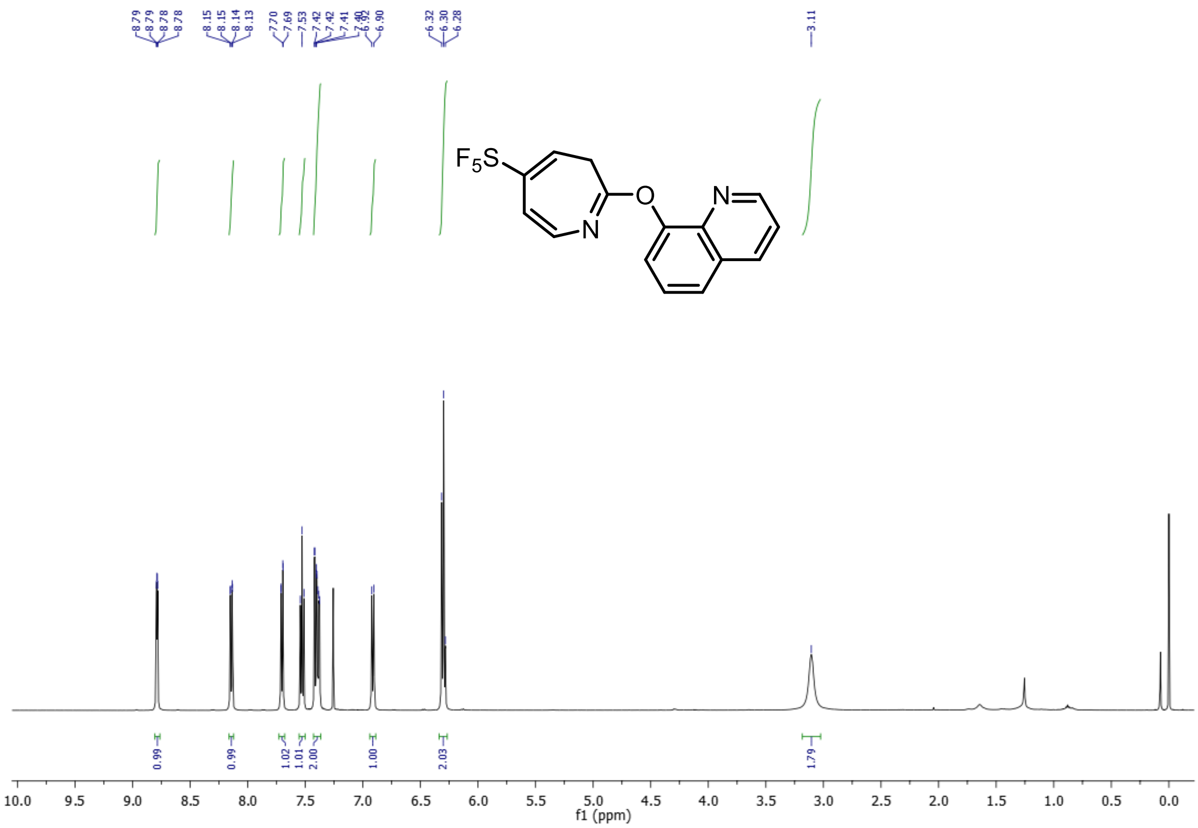

^{13}C NMR (126 MHz, $CDCl_3$) : **3hr**

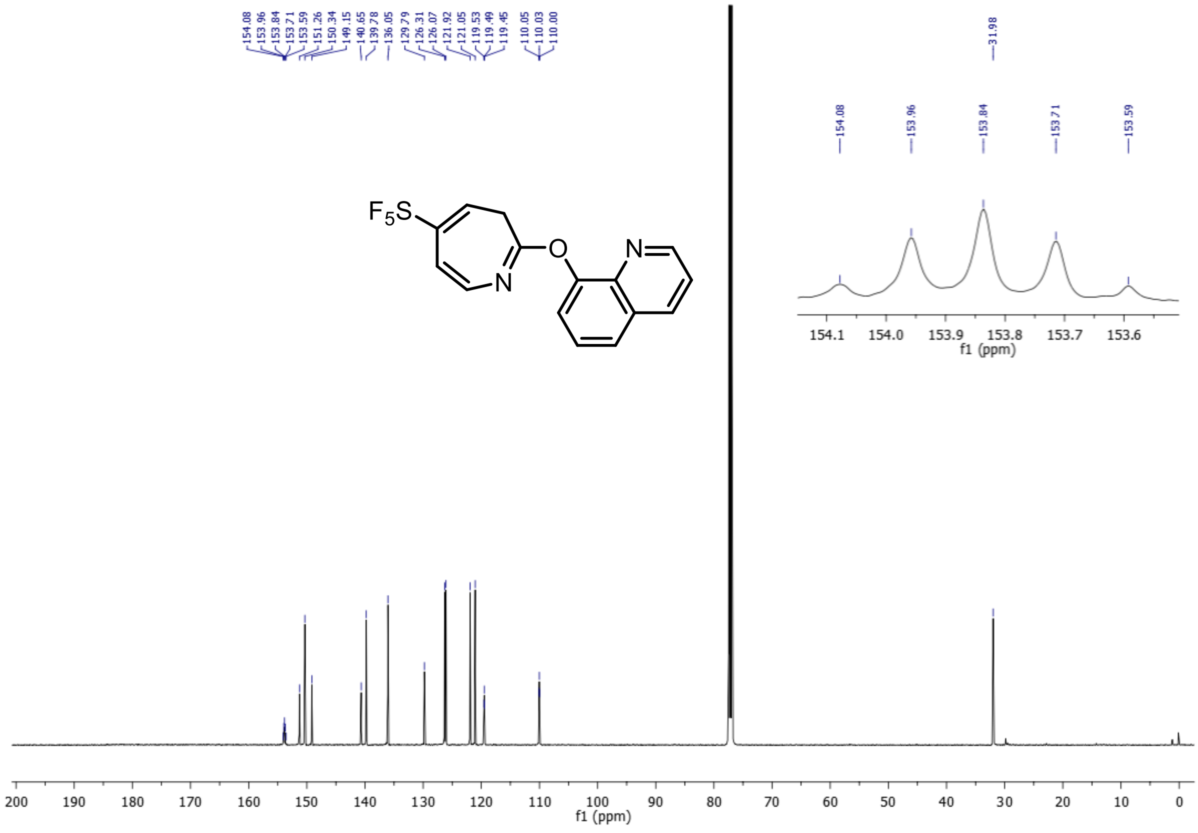

^{19}F NMR (376 MHz, $CDCl_3$) : **3hr**

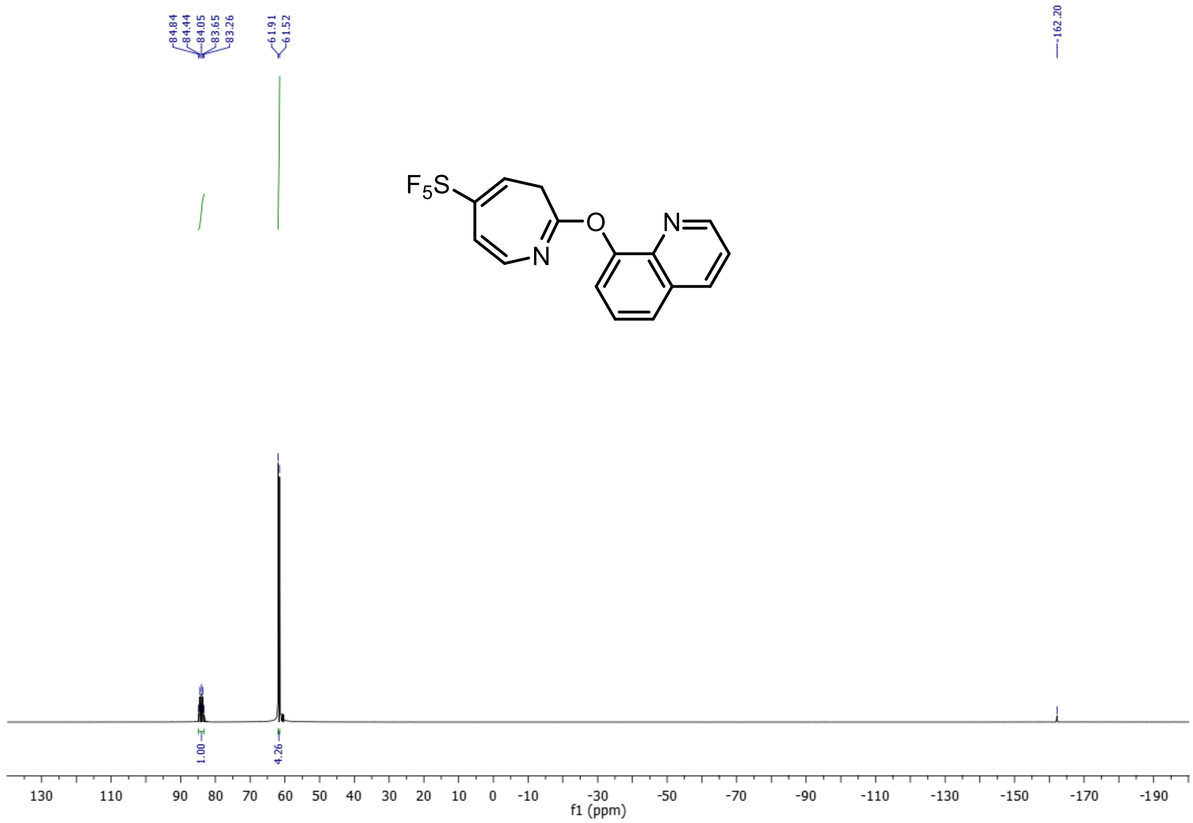

^{1}H NMR (500 MHz, $CDCl_3$) : **3hs**

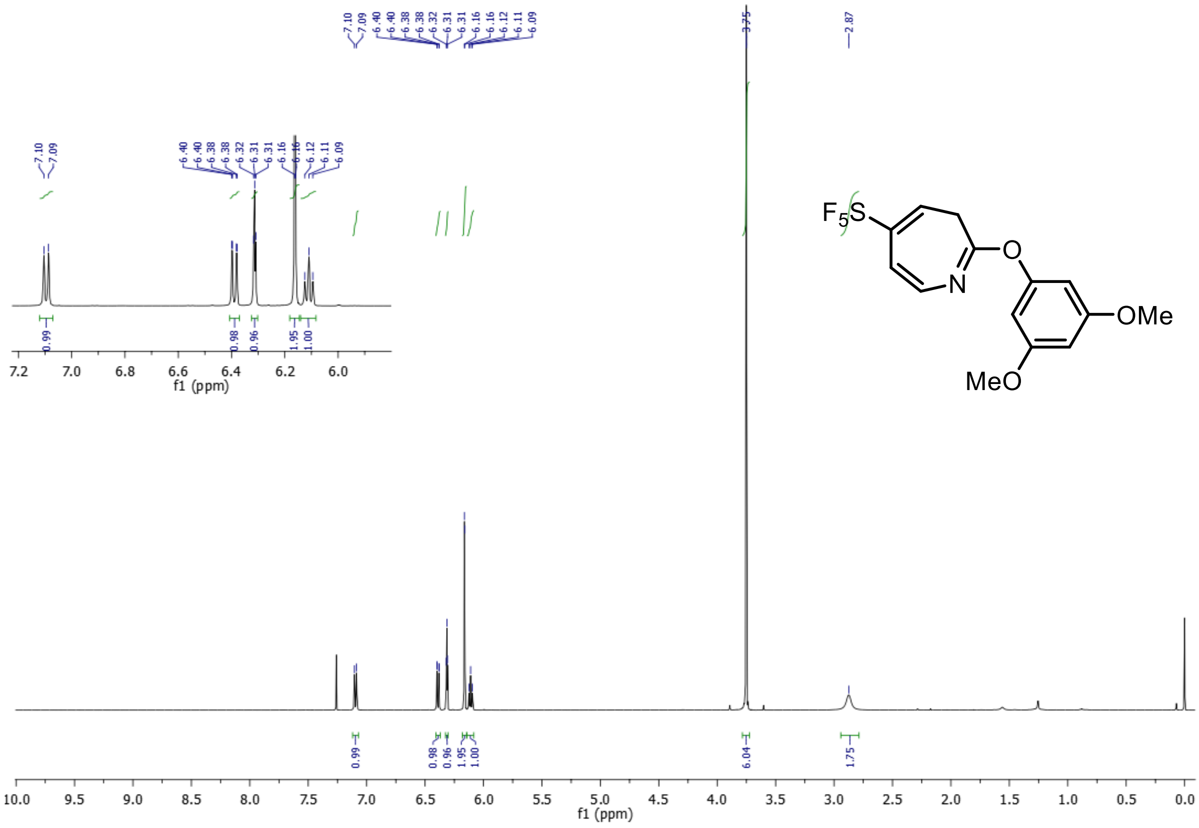

^{13}**C NMR** (126 MHz, $CDCl_3$) : **3hs**

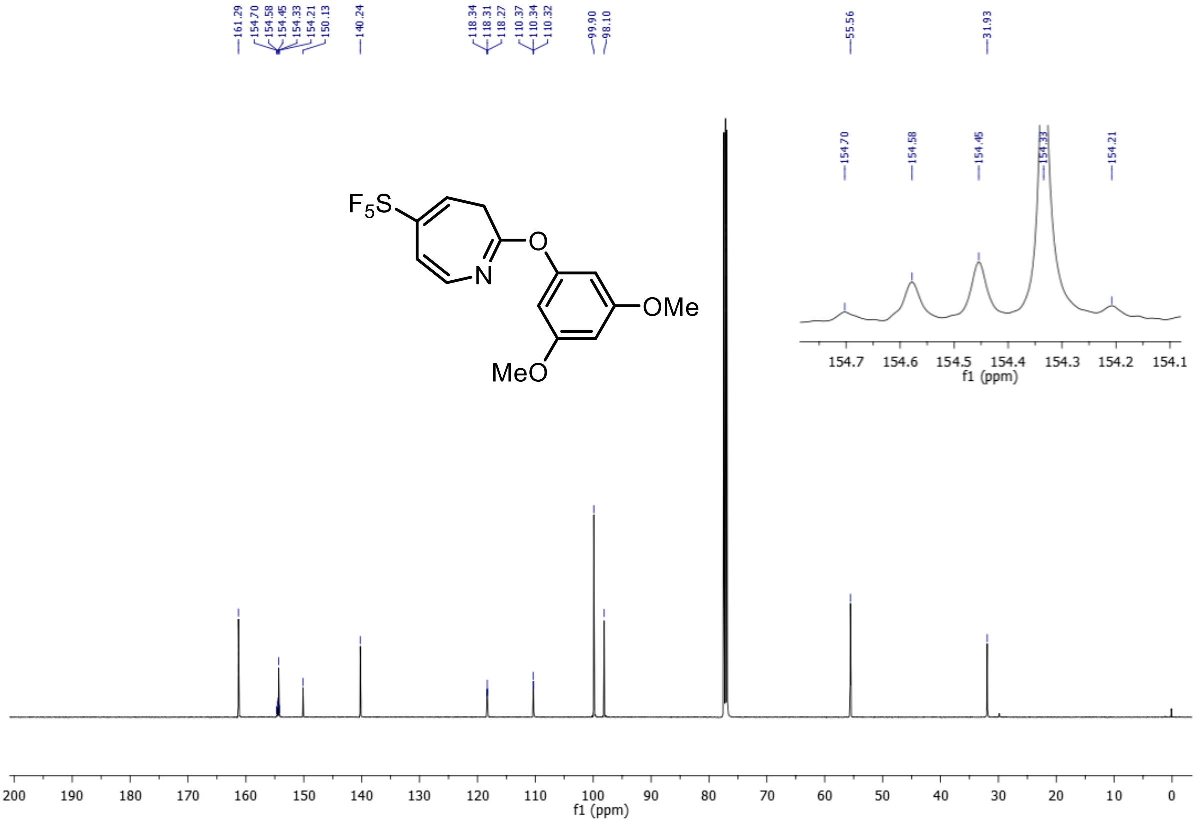

^{19}**F NMR** (282 MHz, $CDCl_3$) : **3hs**

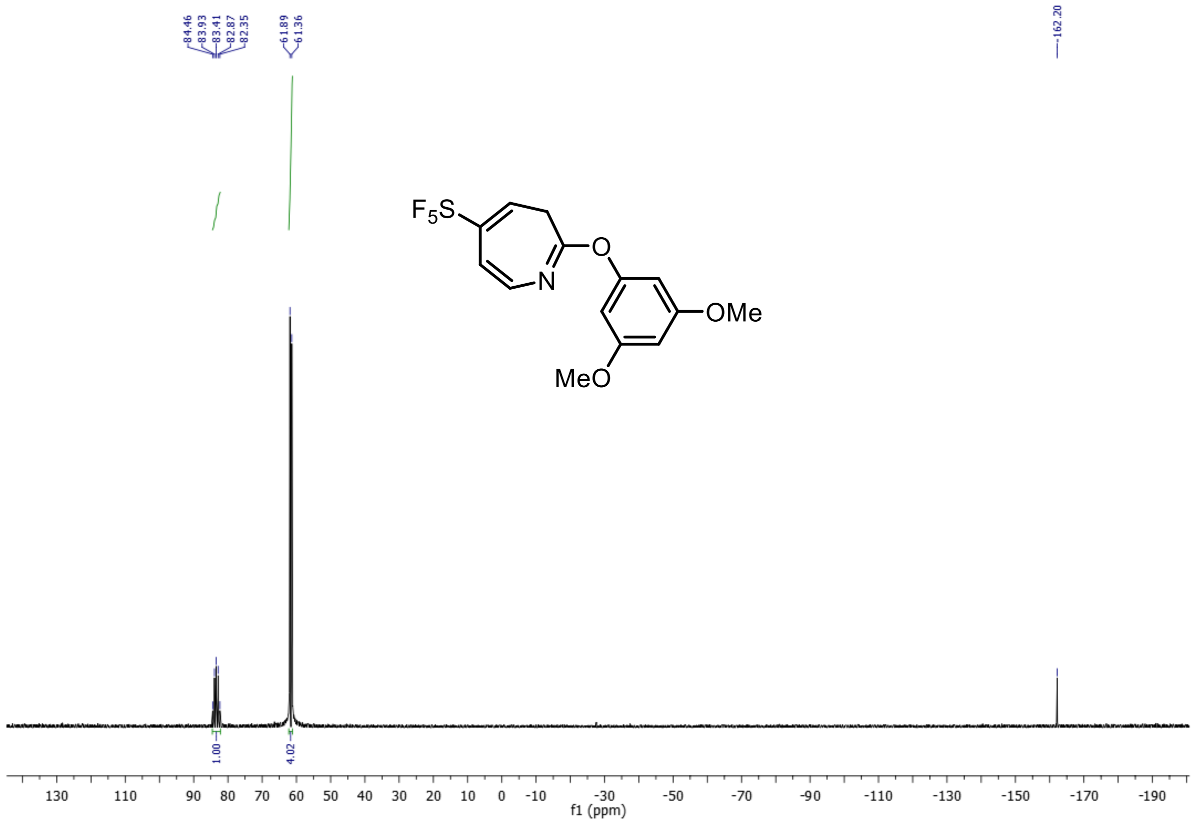

^{1}H NMR (500 MHz, $CDCl_3$) : **3ht**

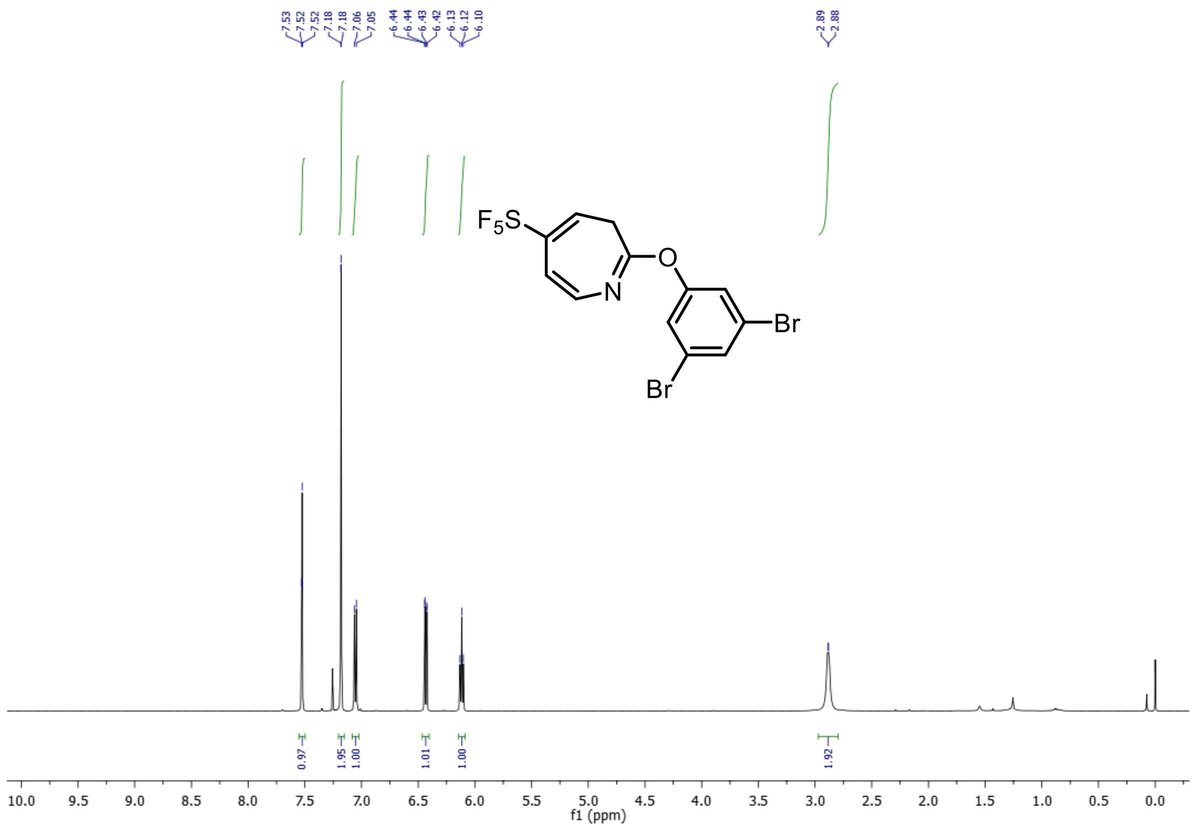

^{13}C NMR (126 MHz, $CDCl_3$) : **3ht**

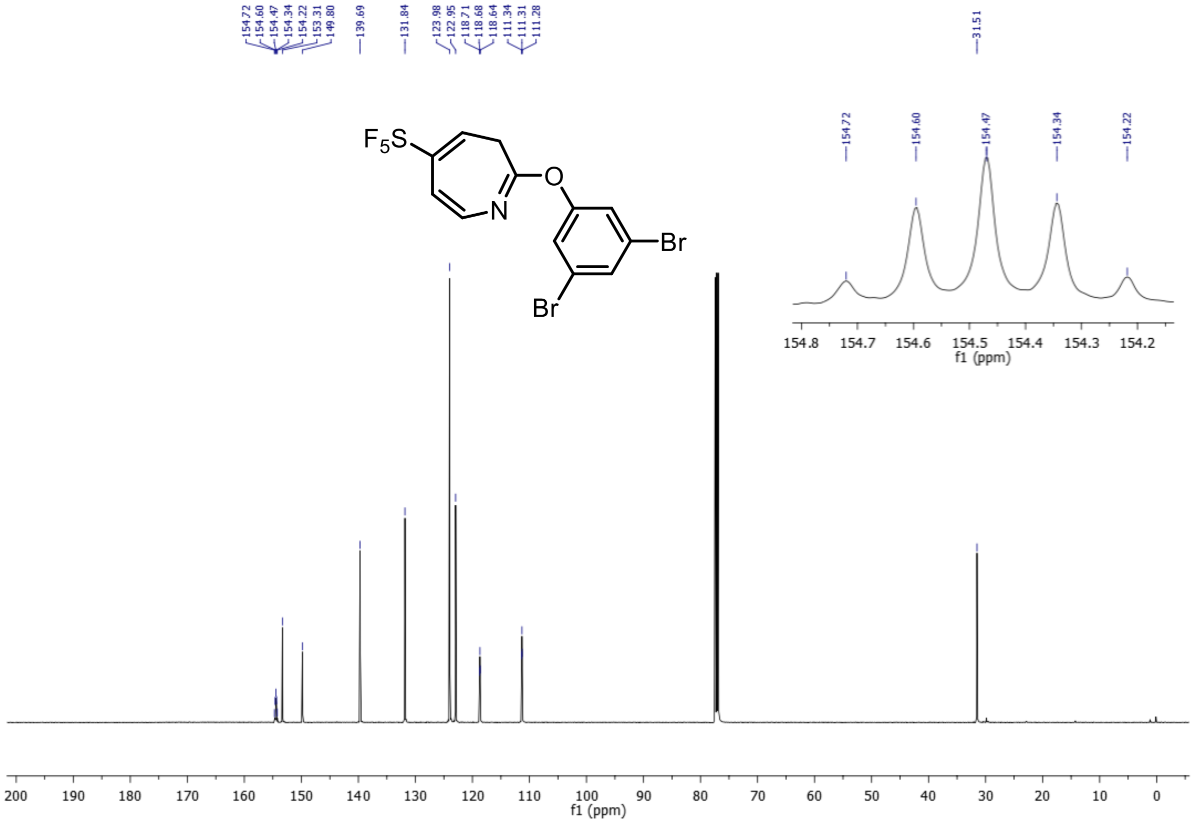

^{19}F NMR (376 MHz, $CDCl_3$) : **3ht**

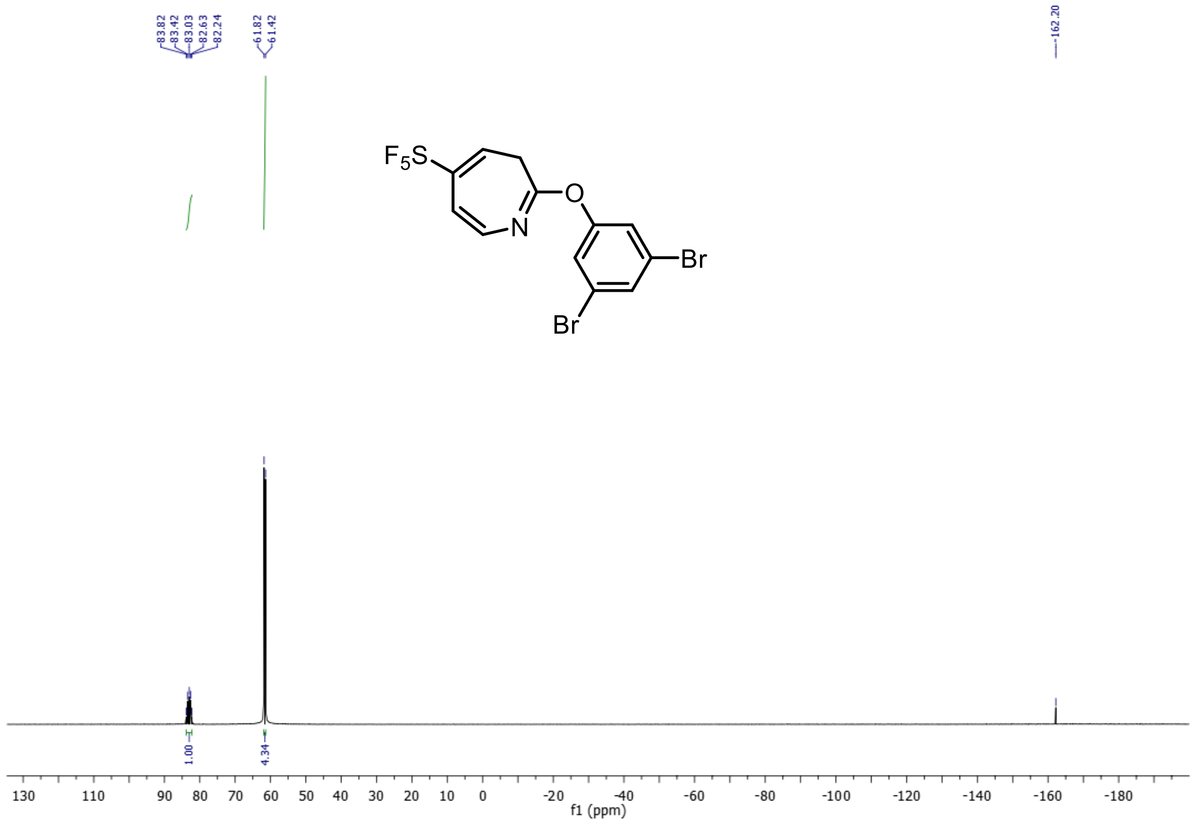

^{1}H NMR (500 MHz, $CDCl_3$) : **3hu**

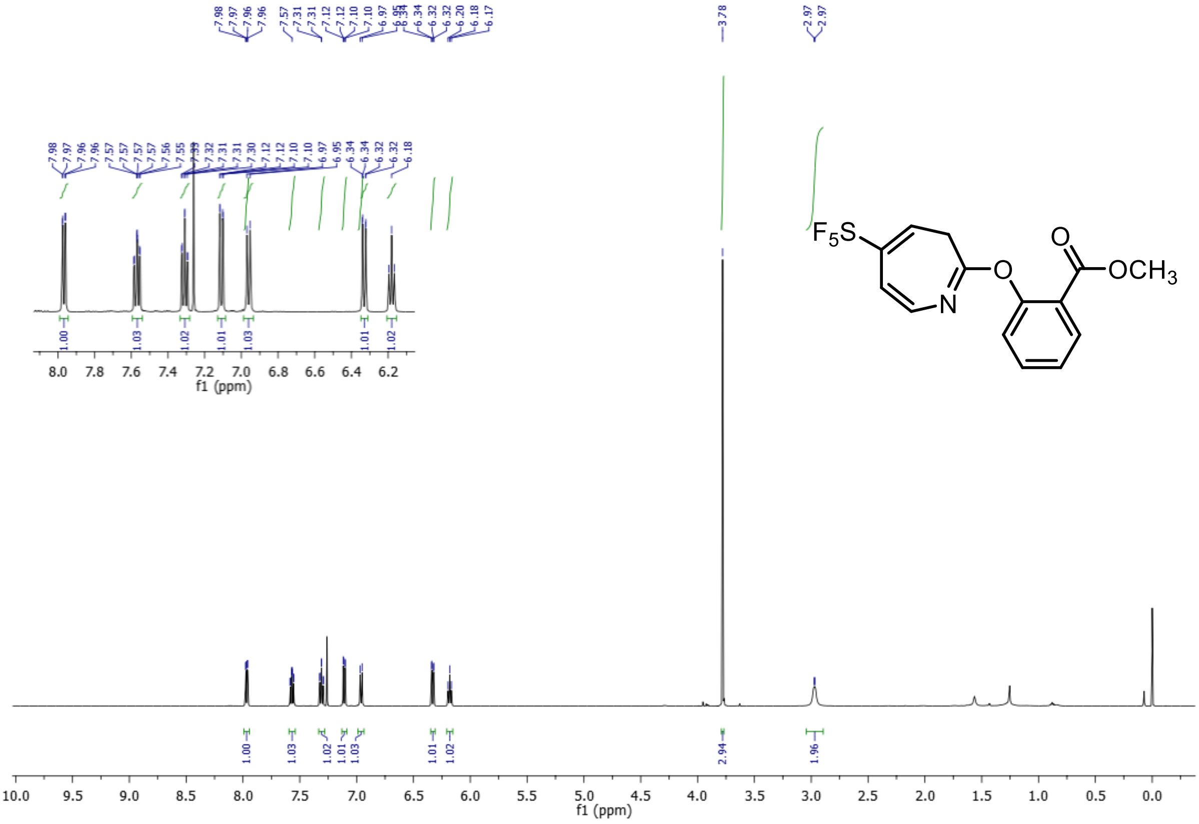

^{13}C NMR (126 MHz, CDCl3) : **3hu**

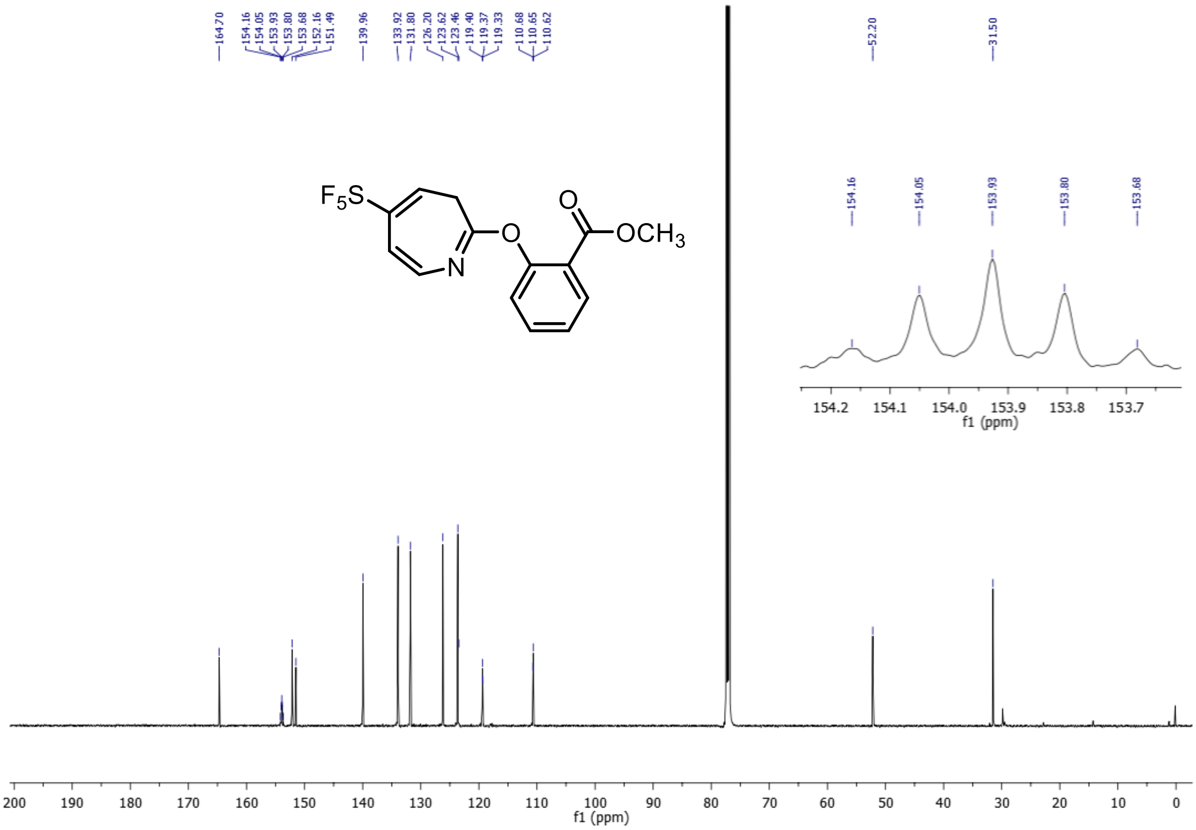

^{19}F NMR (282 MHz, CDCl3) : **3hu**

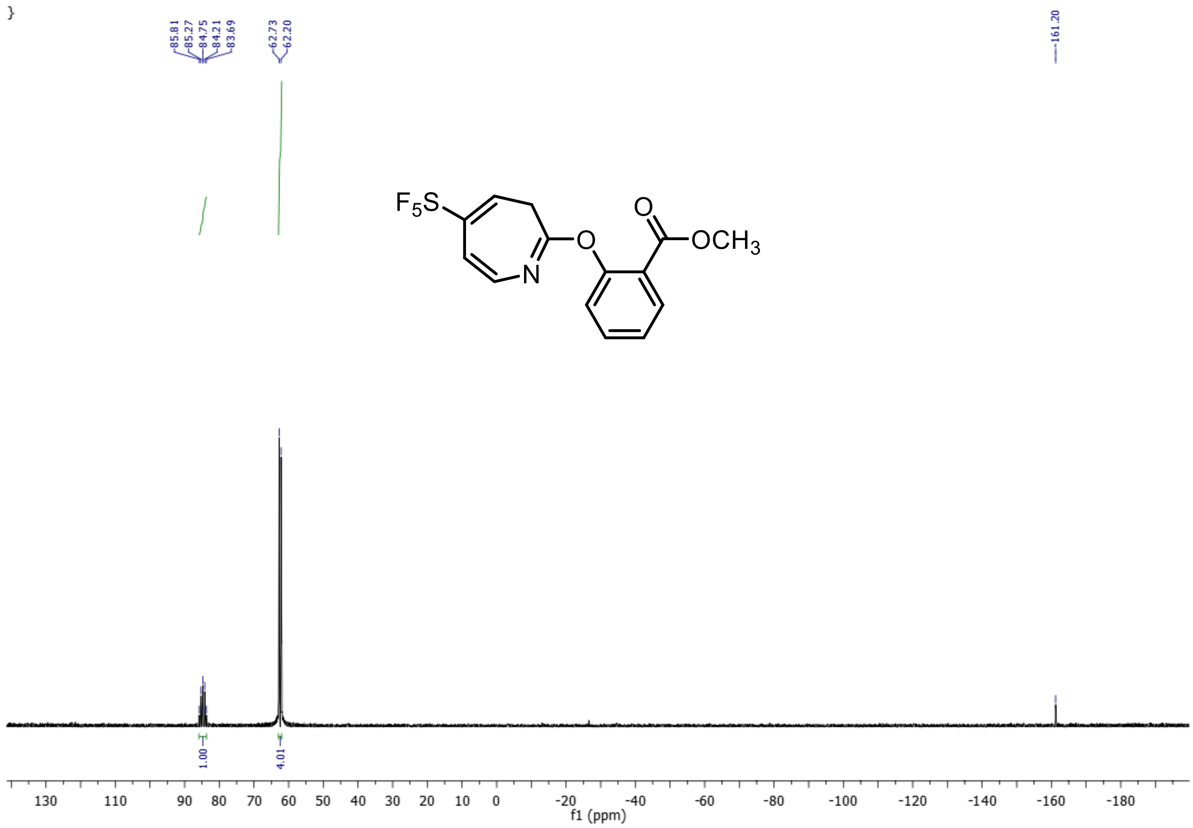

^{1}H NMR (500 MHz, CDCl$_3$) : **3hv**

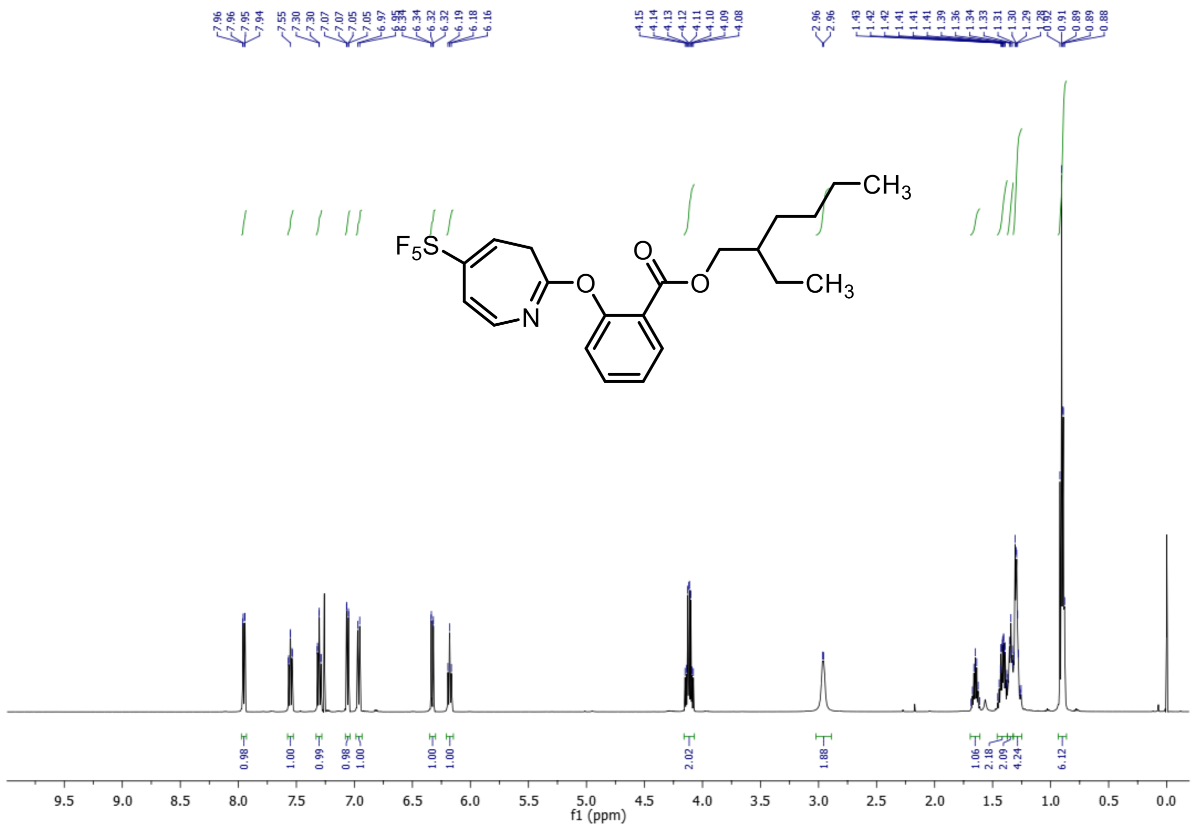

^{13}C NMR (126 MHz, CDCl$_3$) : **3hv**

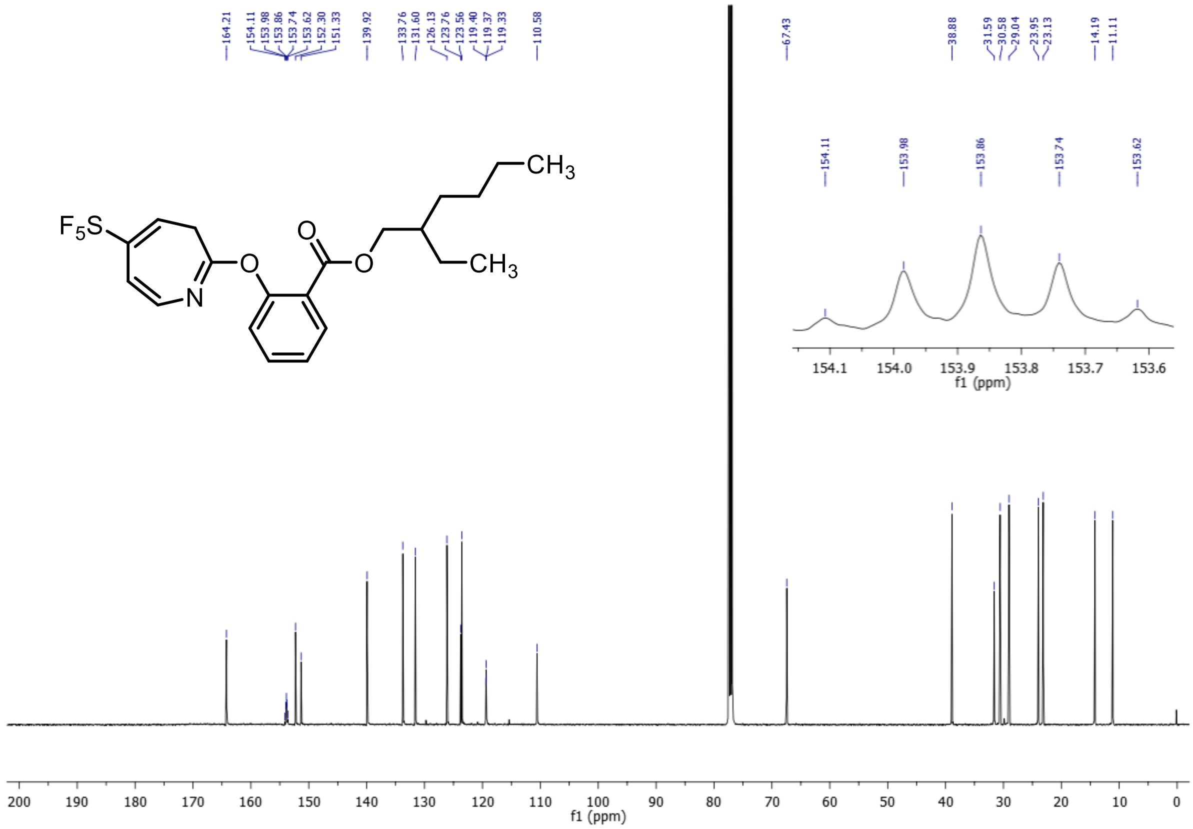

^{19}**F NMR** (282 MHz, $CDCl_3$) : **3hv**

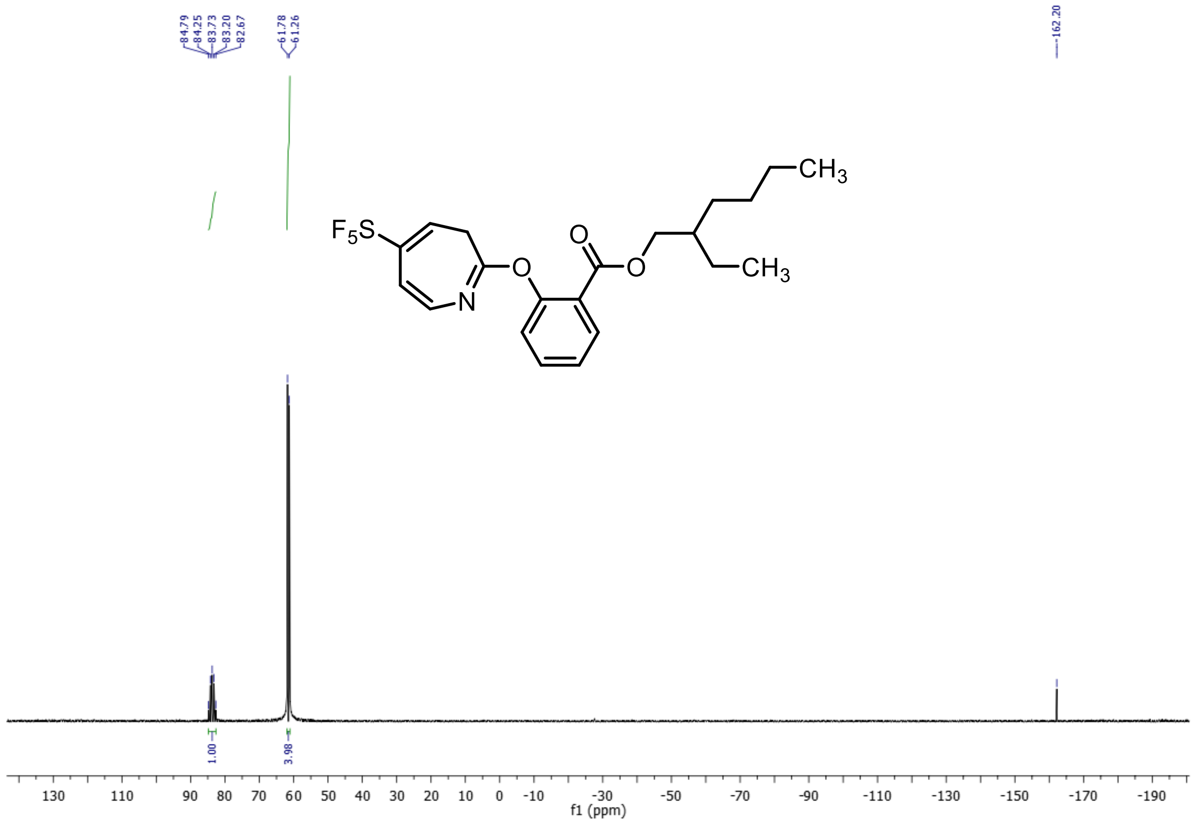

^{1}**H NMR** (500 MHz, $CDCl_3$) : **3hw**

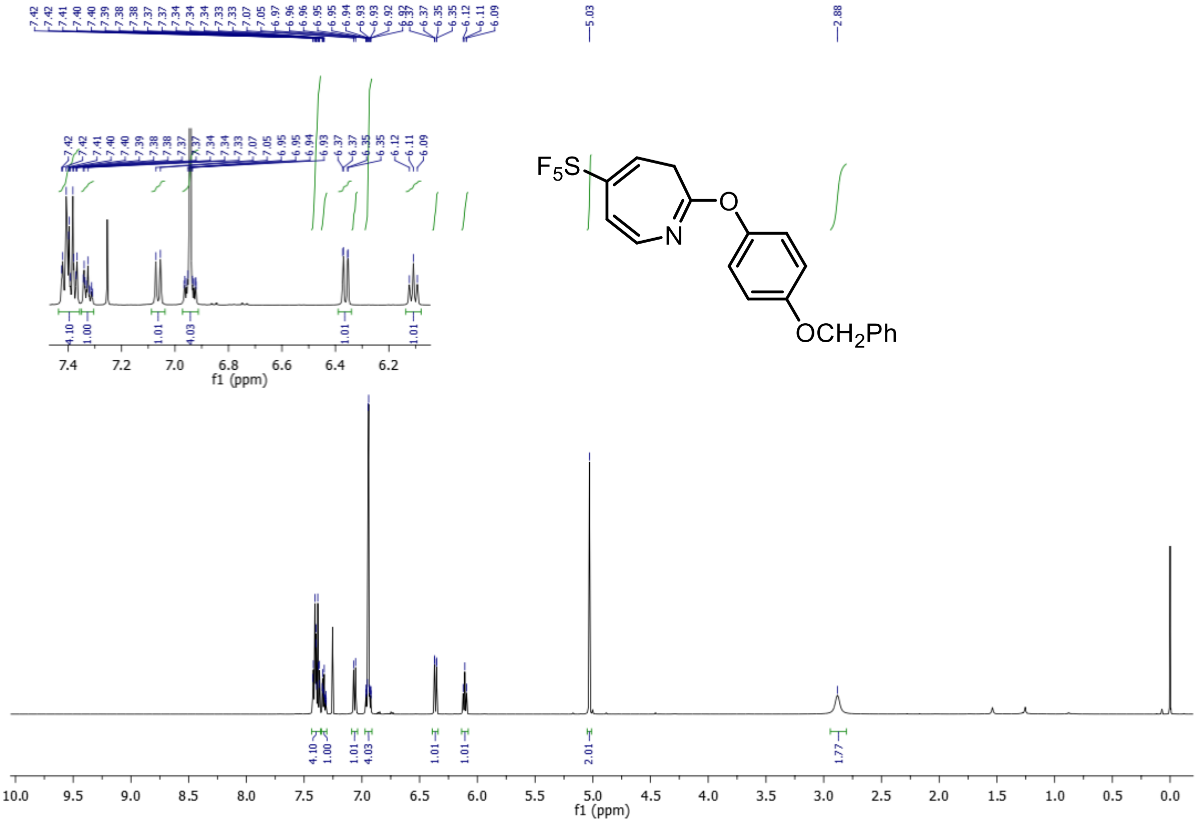

^{13}C NMR (126 MHz, $CDCl_3$) : **3hw**

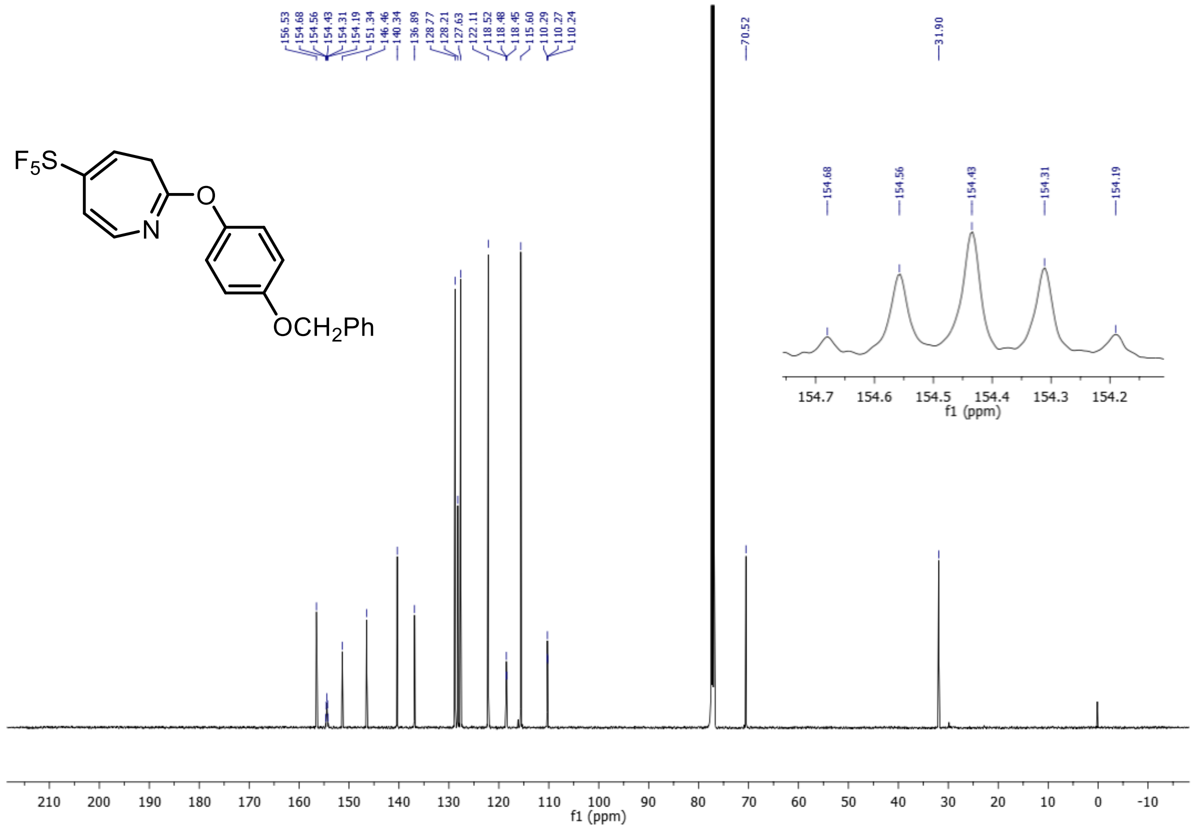

^{19}F NMR (282 MHz, $CDCl_3$) : **3hw**

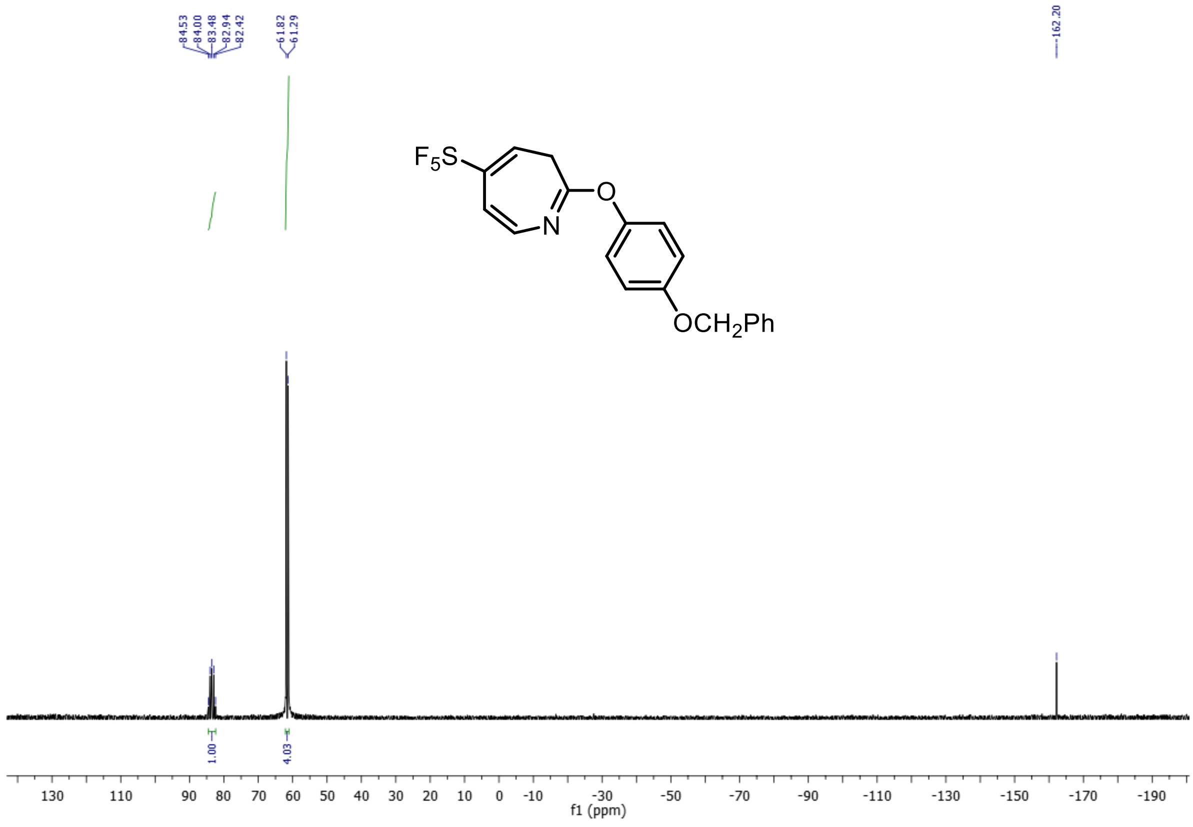

^{1}H NMR (500 MHz, $CDCl_3$) : **3hx**

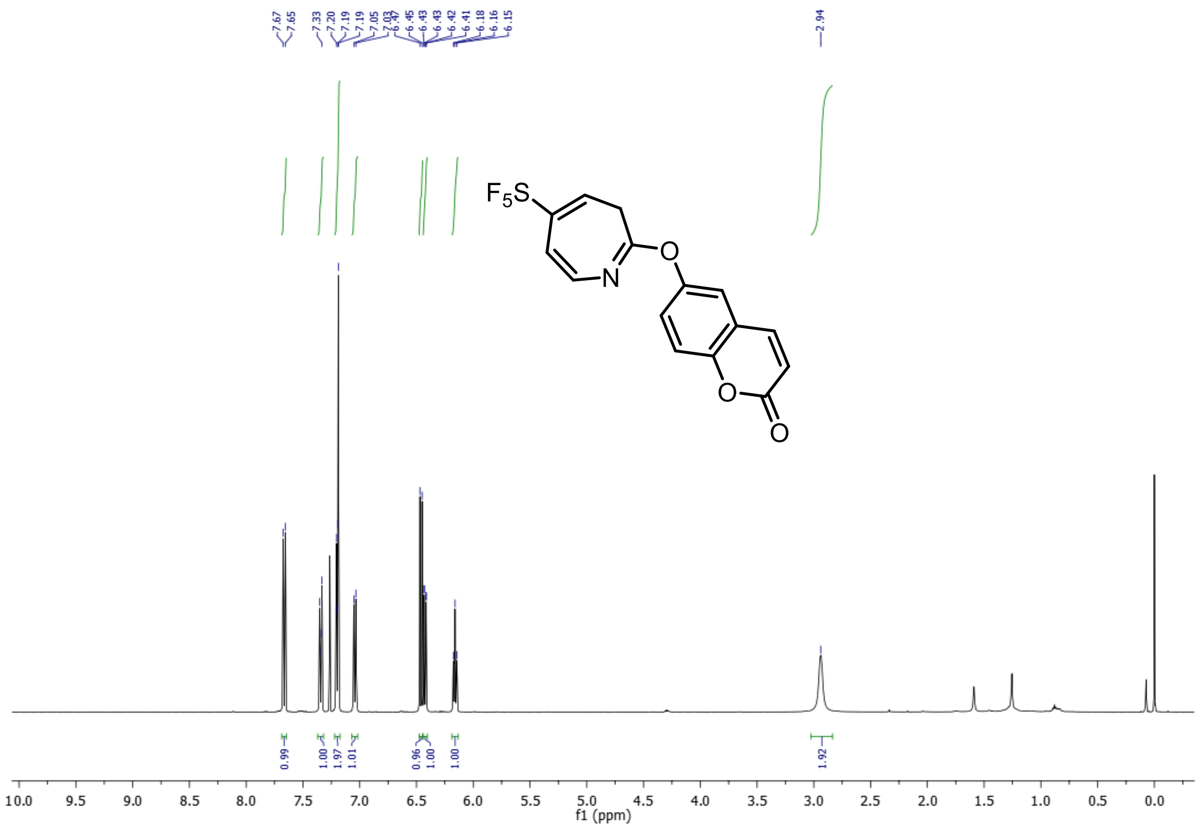

^{13}C NMR (126 MHz, $CDCl_3$) : **3hx**

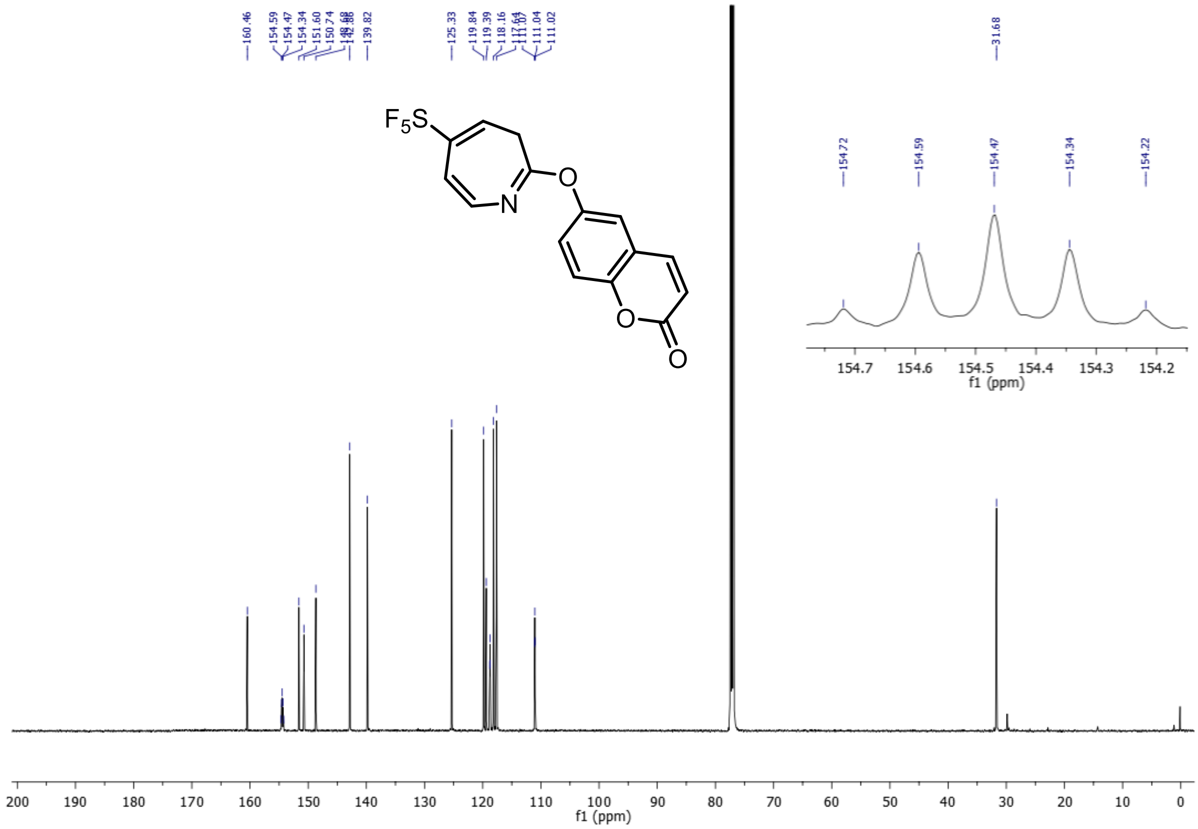

^{19}F NMR (282 MHz, $CDCl_3$) : **3hx**

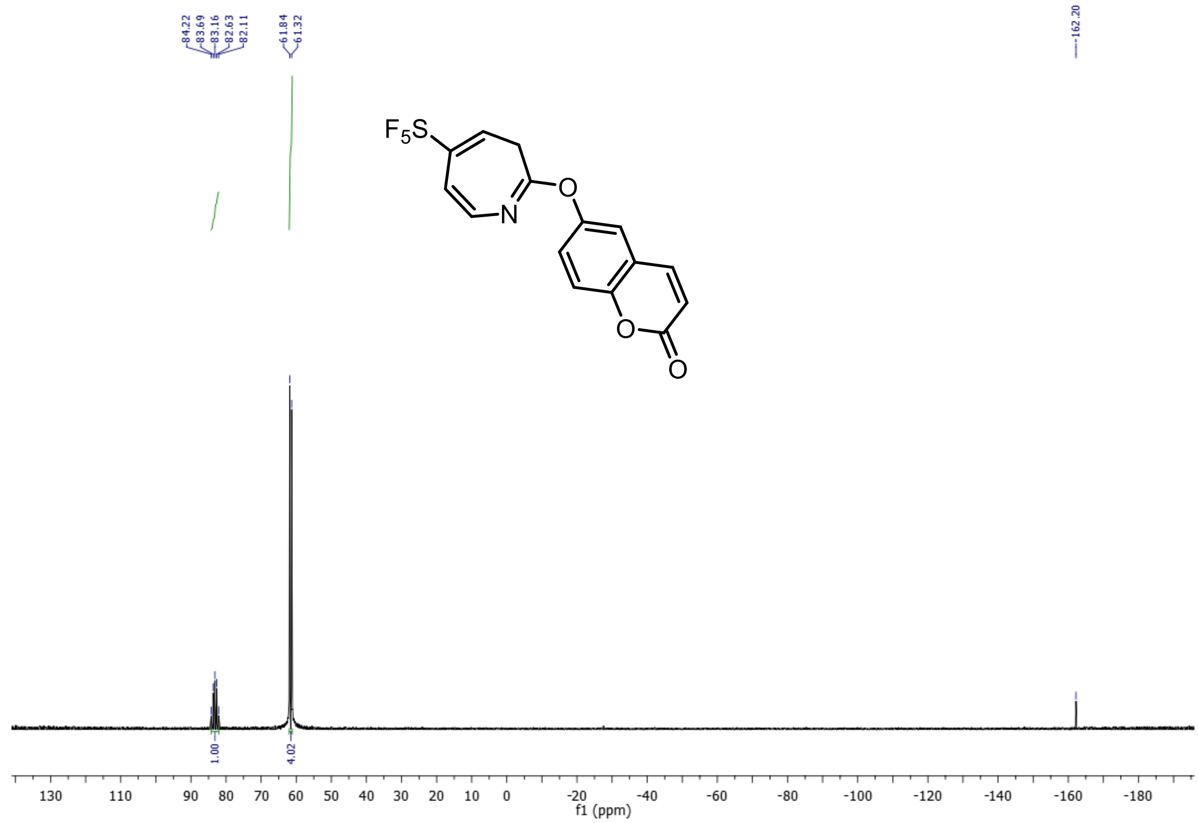

^{1}H NMR (500 MHz, $CDCl_3$) : **3hy**

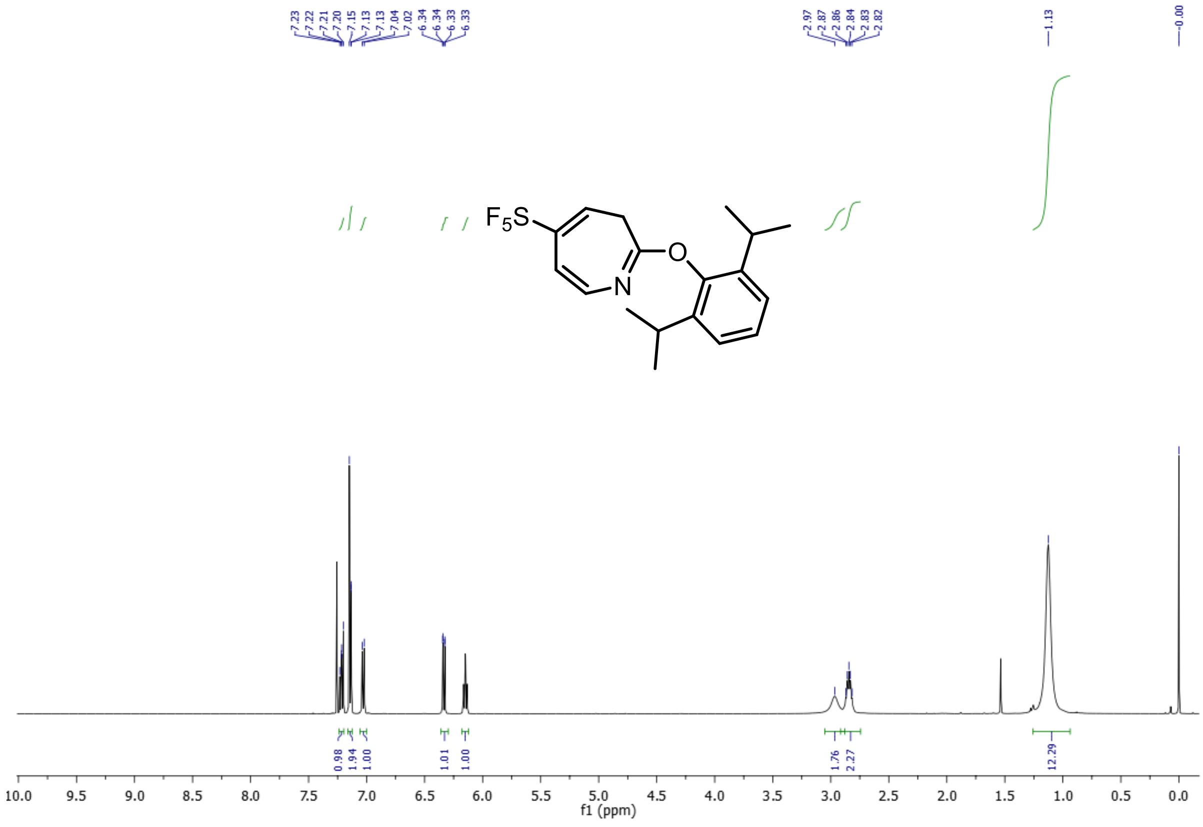

^{13}C NMR (126 MHz, $CDCl_3$) : **3hy**

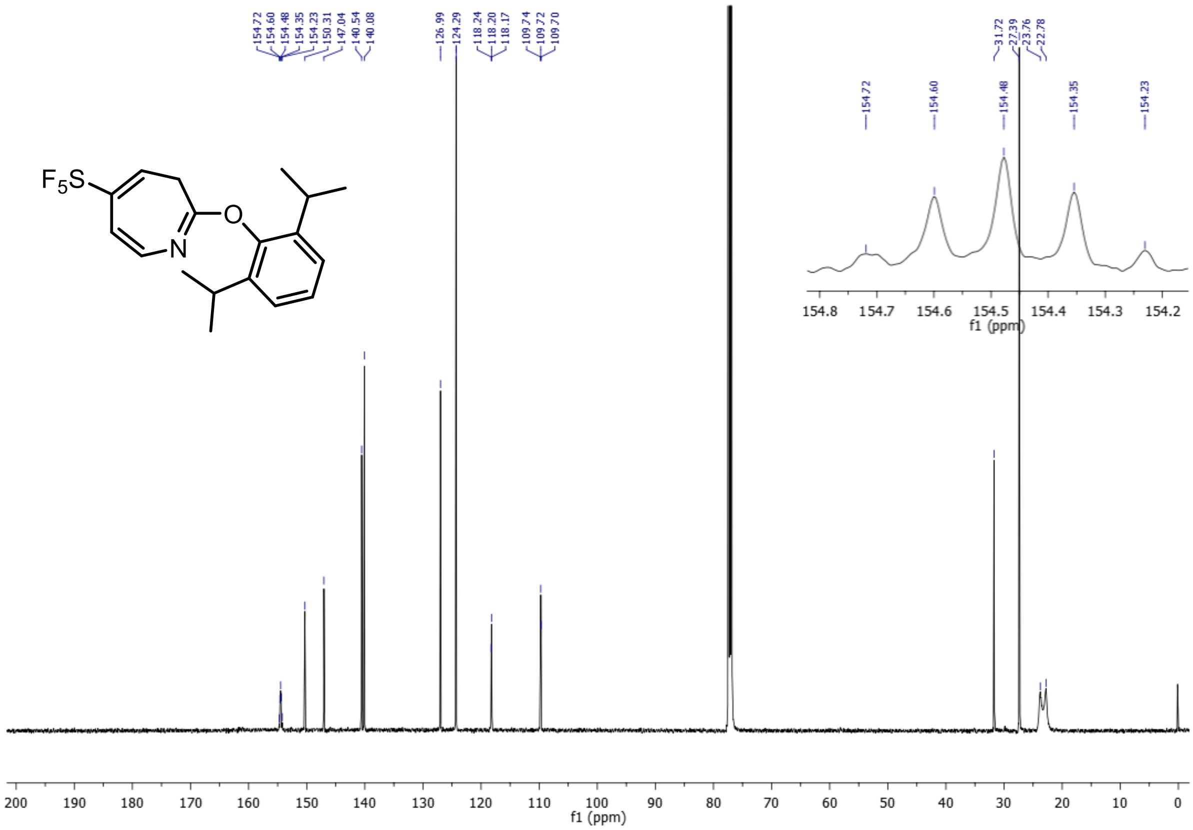

^{19}F NMR (282 MHz, $CDCl_3$) : **3hy**

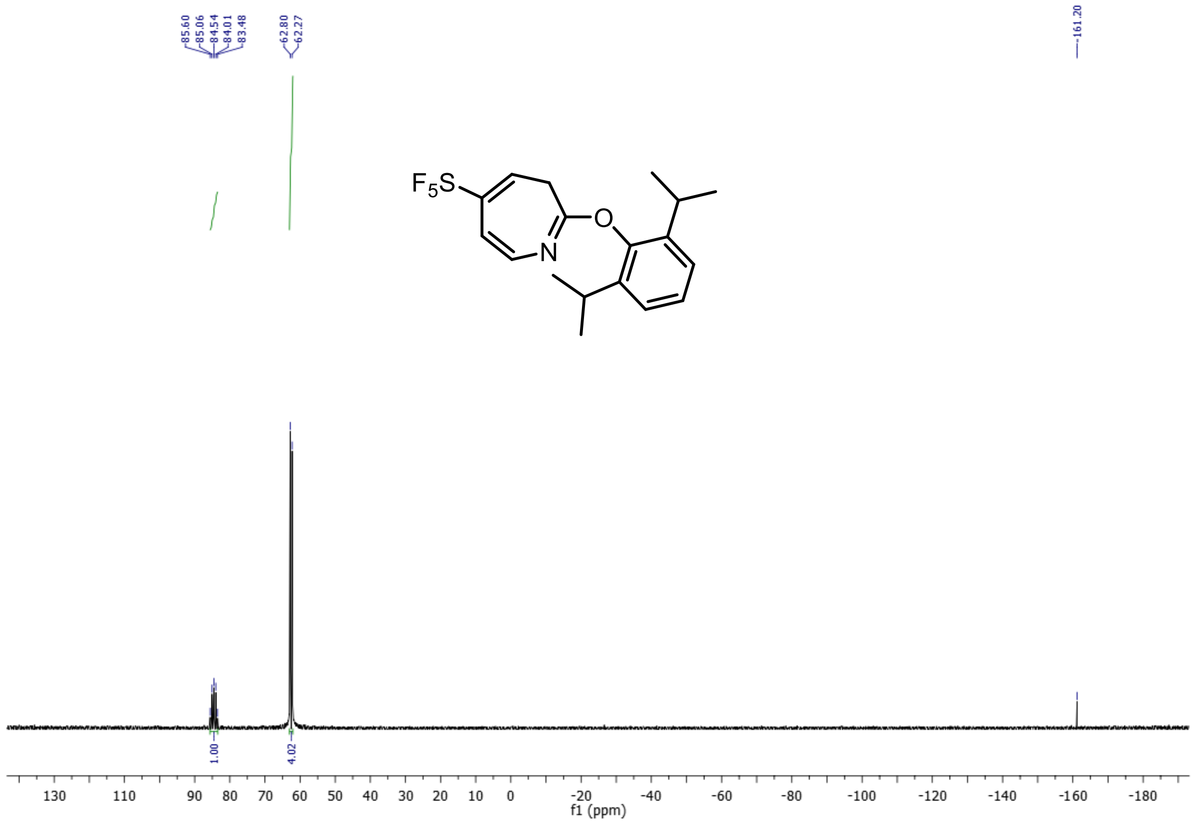

^{1}H NMR (500 MHz, $CDCl_3$) : **3hz**

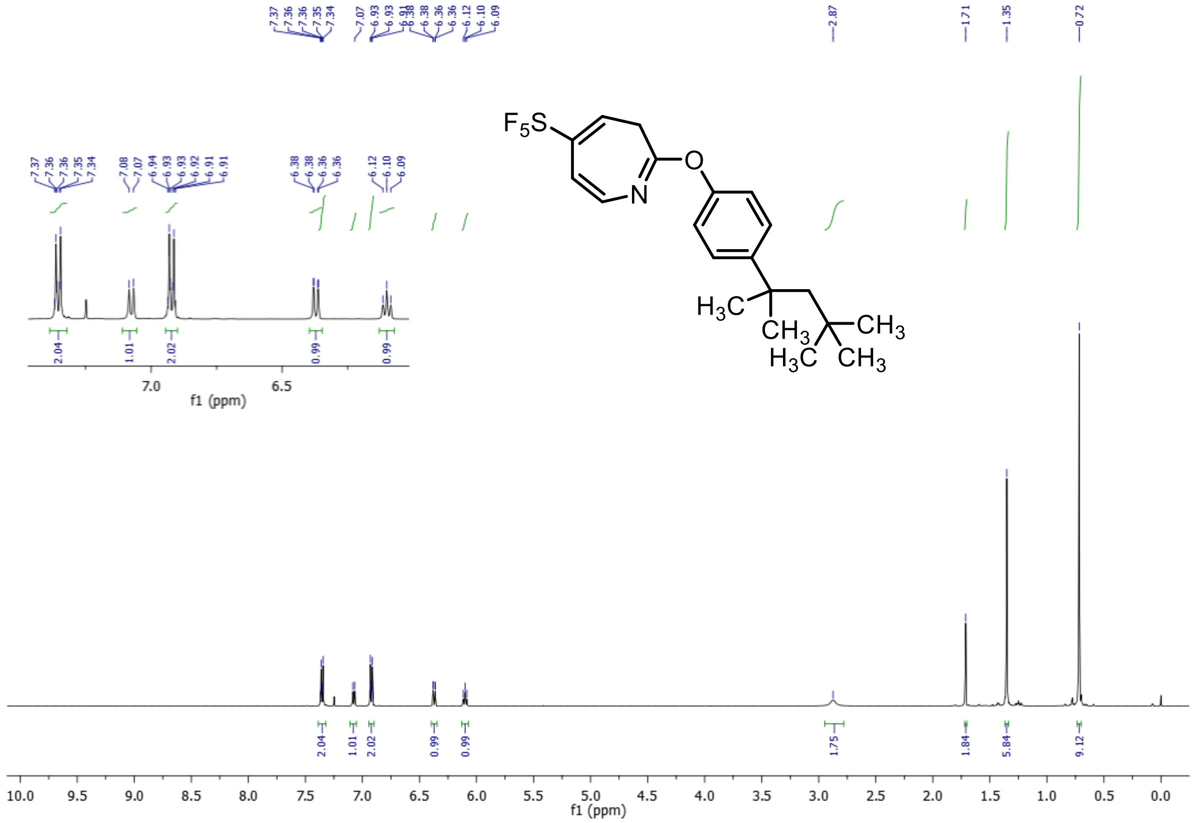

^{13}C NMR (126 MHz, $CDCl_3$) : **3hz**

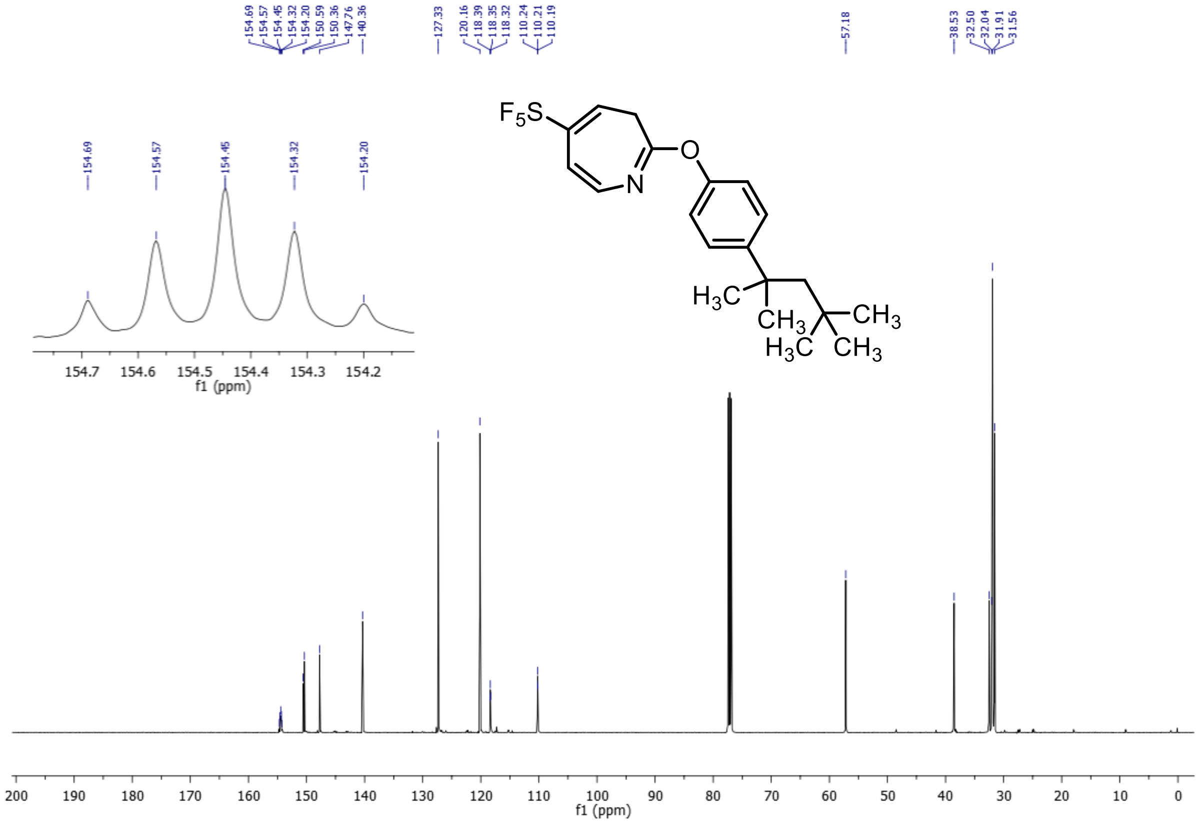

^{19}**F NMR** (282 MHz, $CDCl_3$) : **3hz**

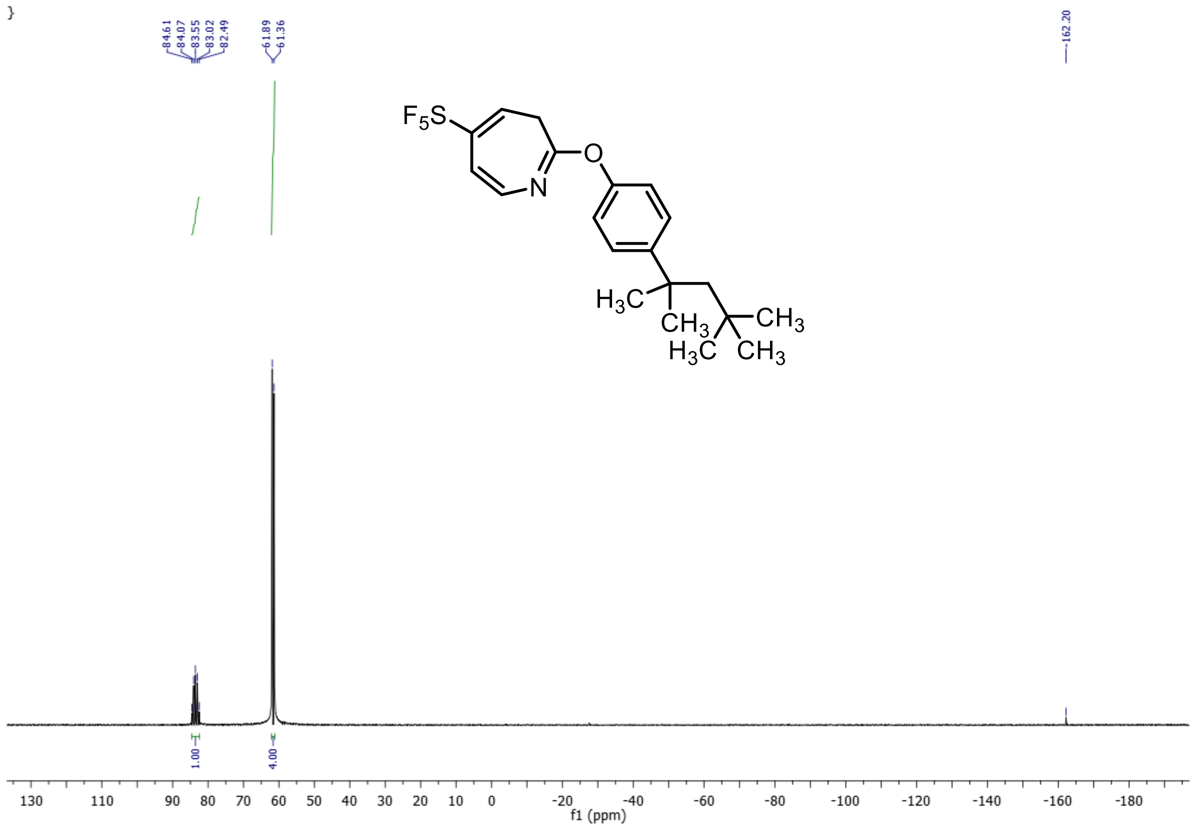

^{1}**H NMR** (500 MHz, $CDCl_3$) : **3haa**

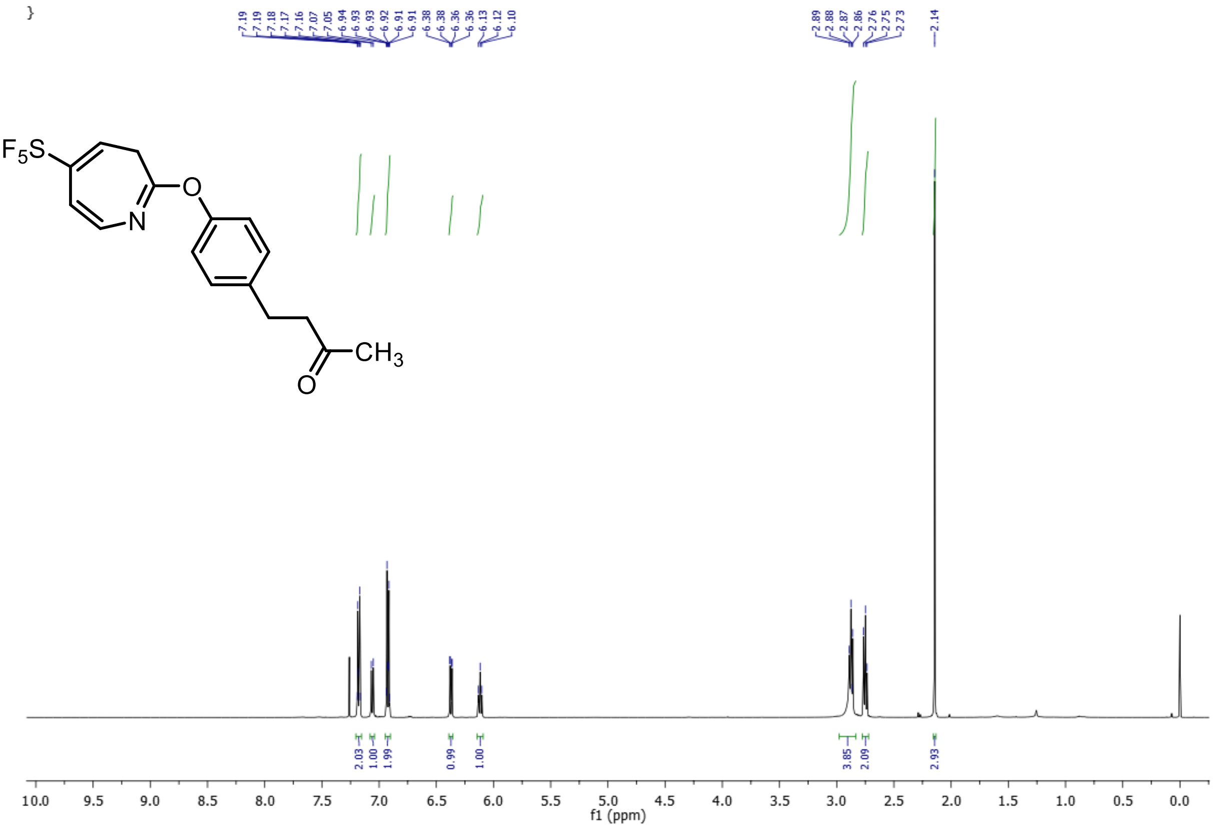

^{13}C NMR (126 MHz, CDCl$_3$) : **3haa**

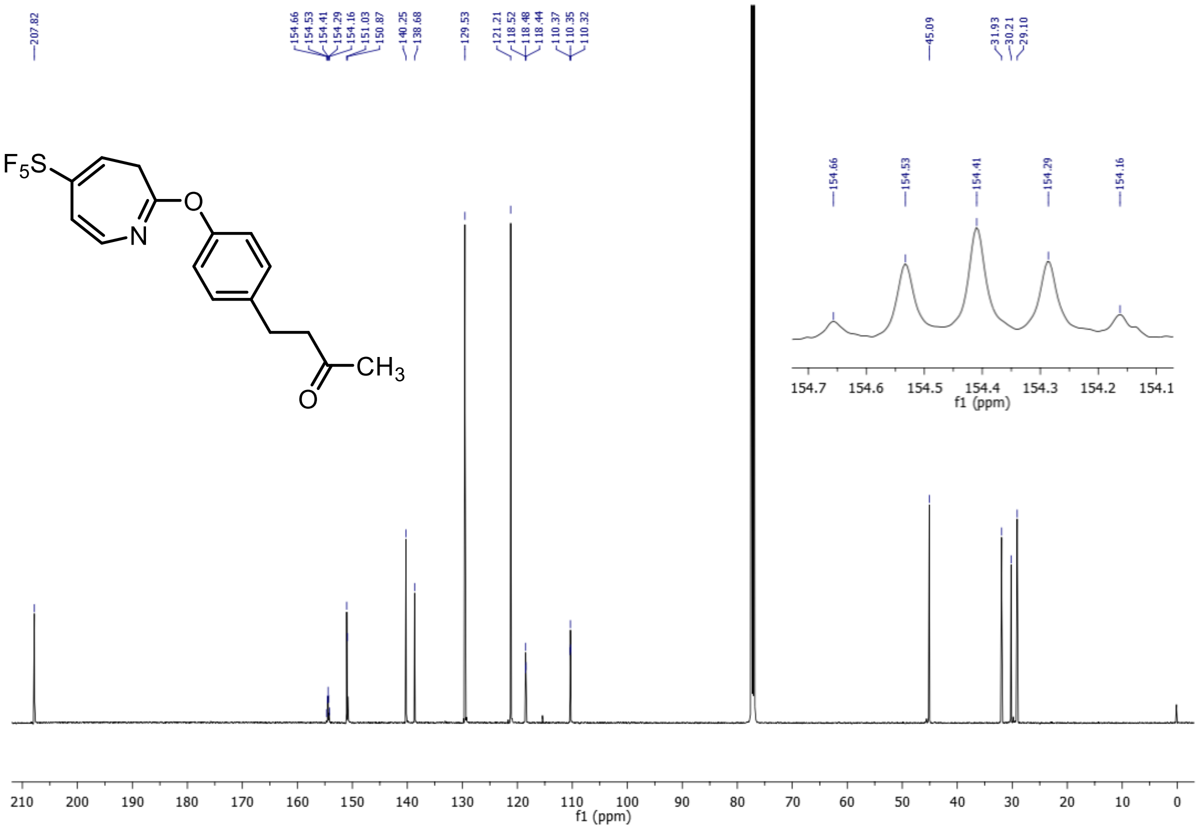

^{19}F NMR (282 MHz, CDCl$_3$) : **3haa**

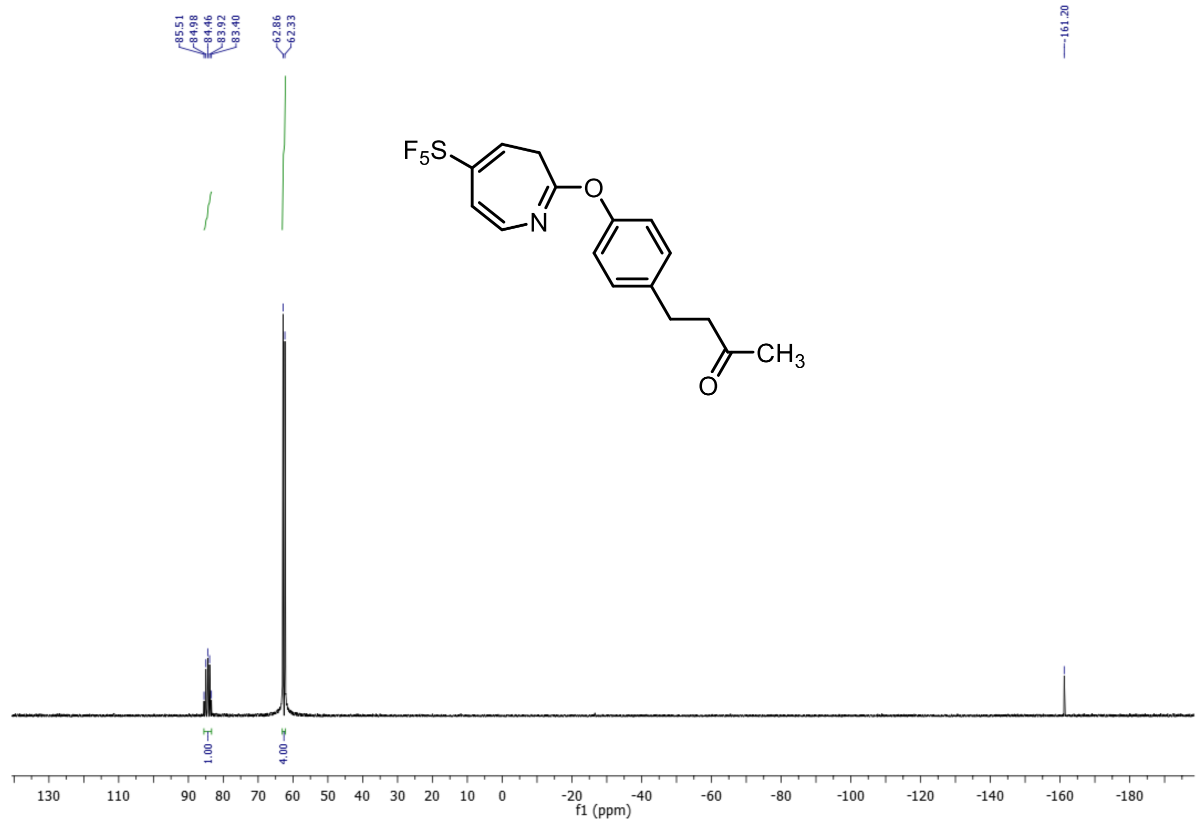

^{1}H NMR (500 MHz, CDCl$_3$) : **3hab**

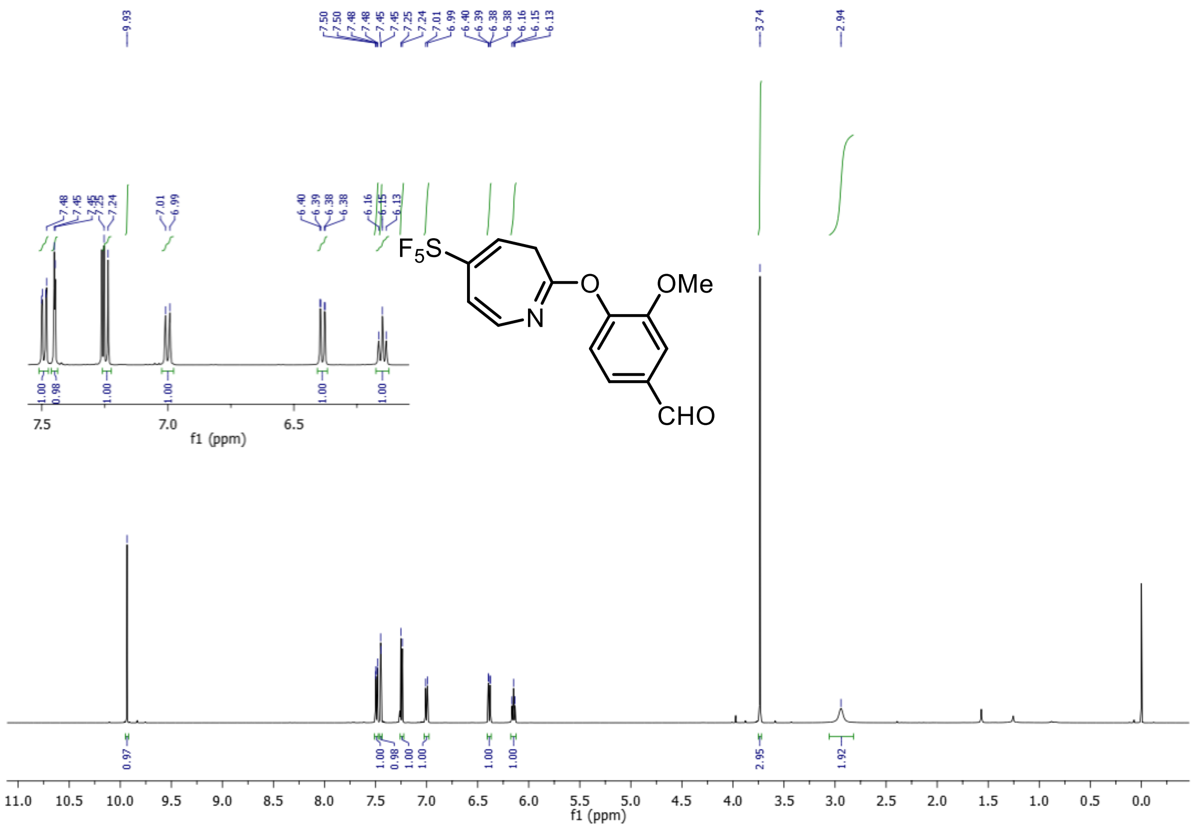

^{13}C NMR (126 MHz, CDCl$_3$) : **3hab**

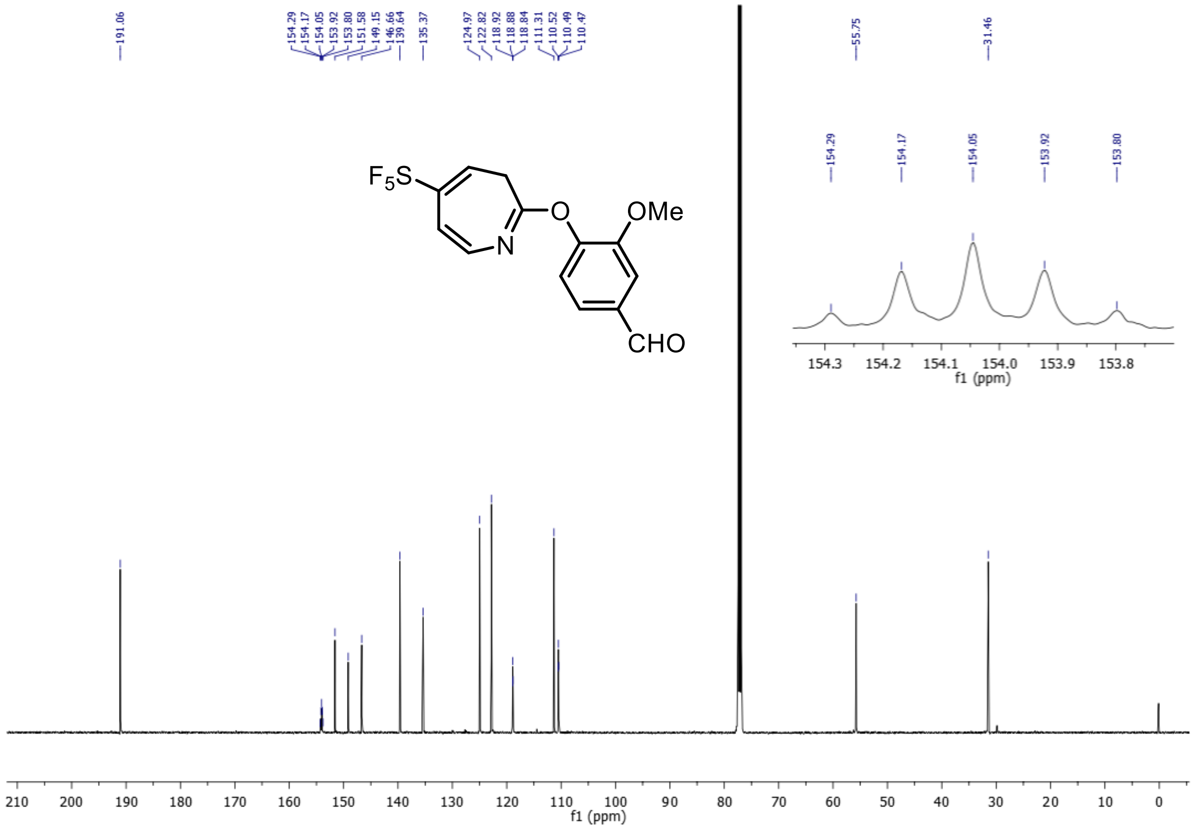

^{19}F NMR (282 MHz, $CDCl_3$) : **3hab**

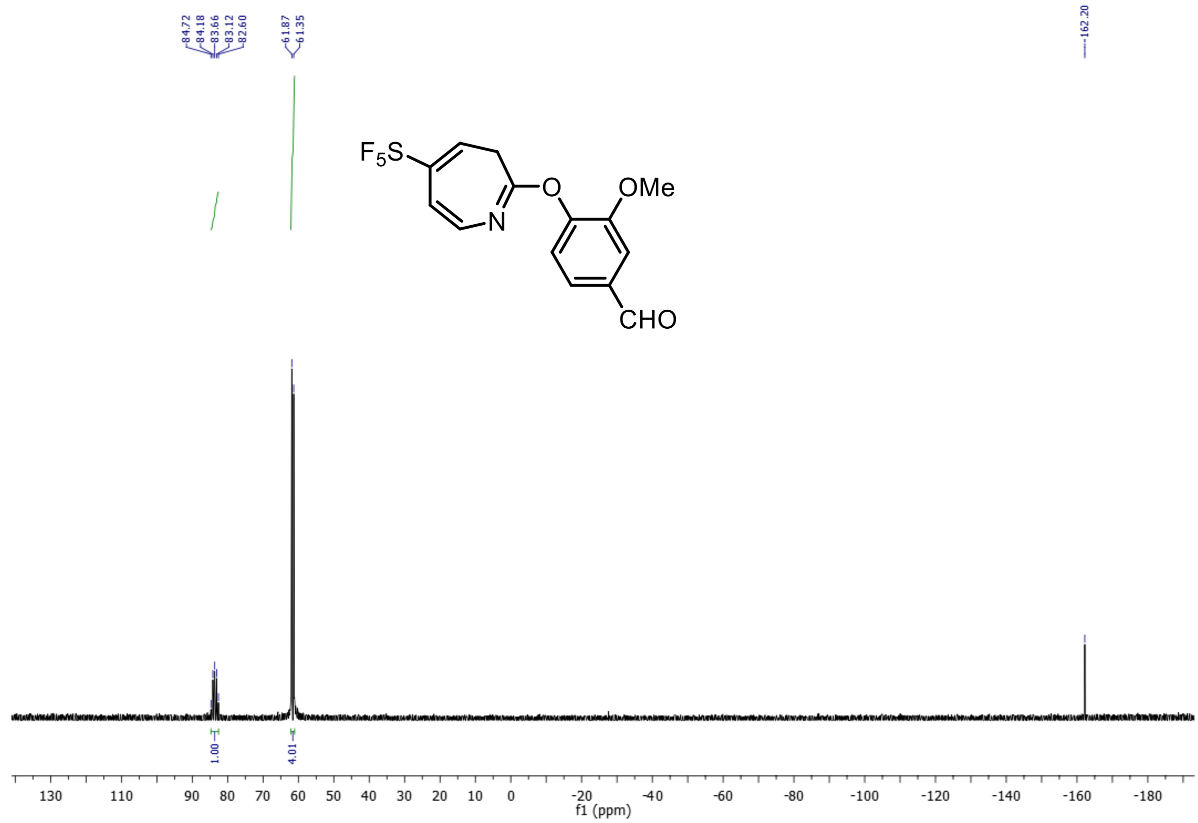

^{1}H NMR (500 MHz, $CDCl_3$) : **3hac**

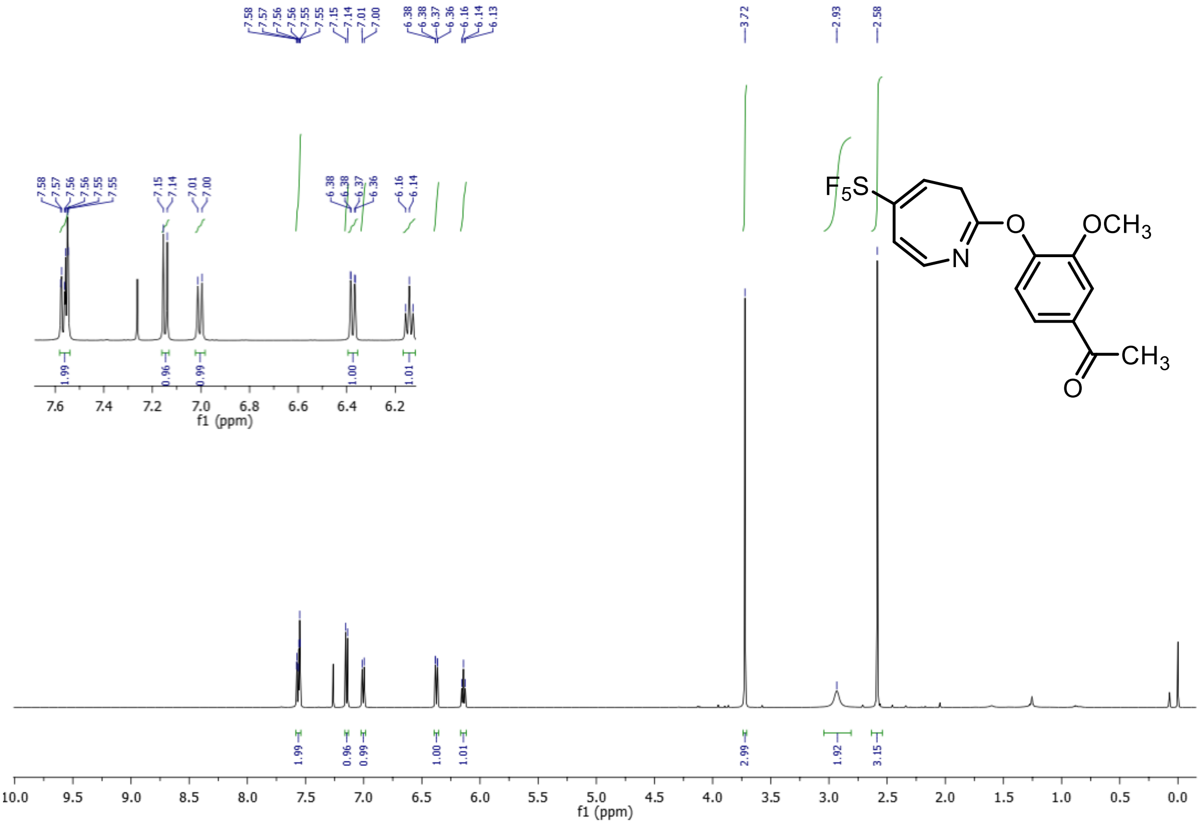

^{13}C NMR (126 MHz, $CDCl_3$) : **3hac**

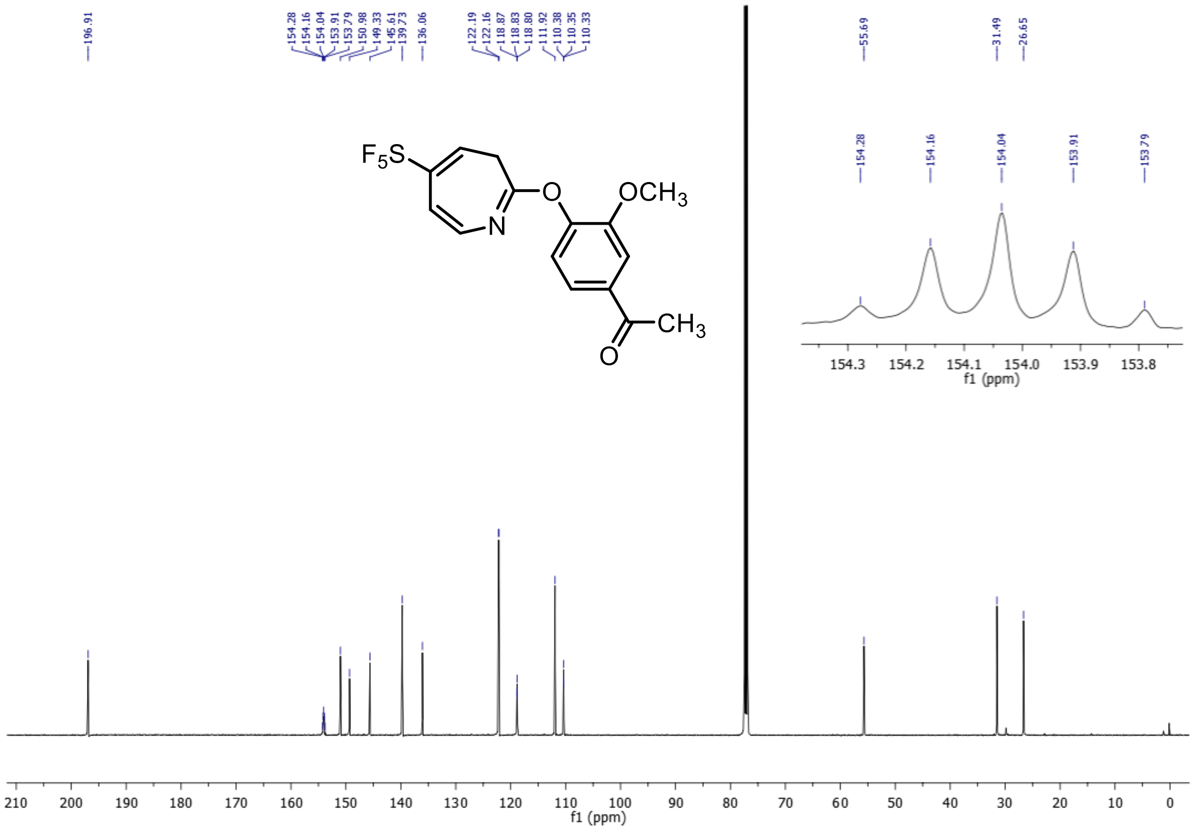

^{19}F NMR (282 MHz, $CDCl_3$) : **3hac**

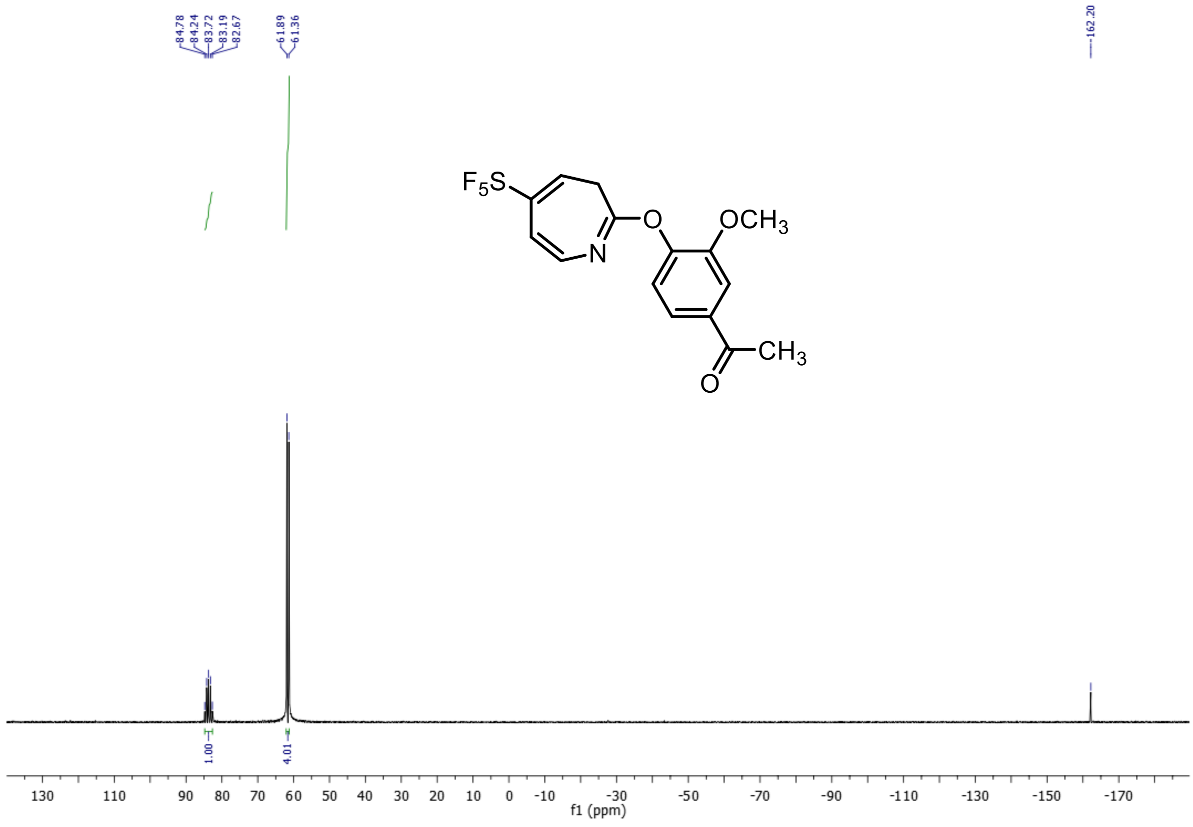

^{1}H NMR (500 MHz, $CDCl_3$) : **3had**

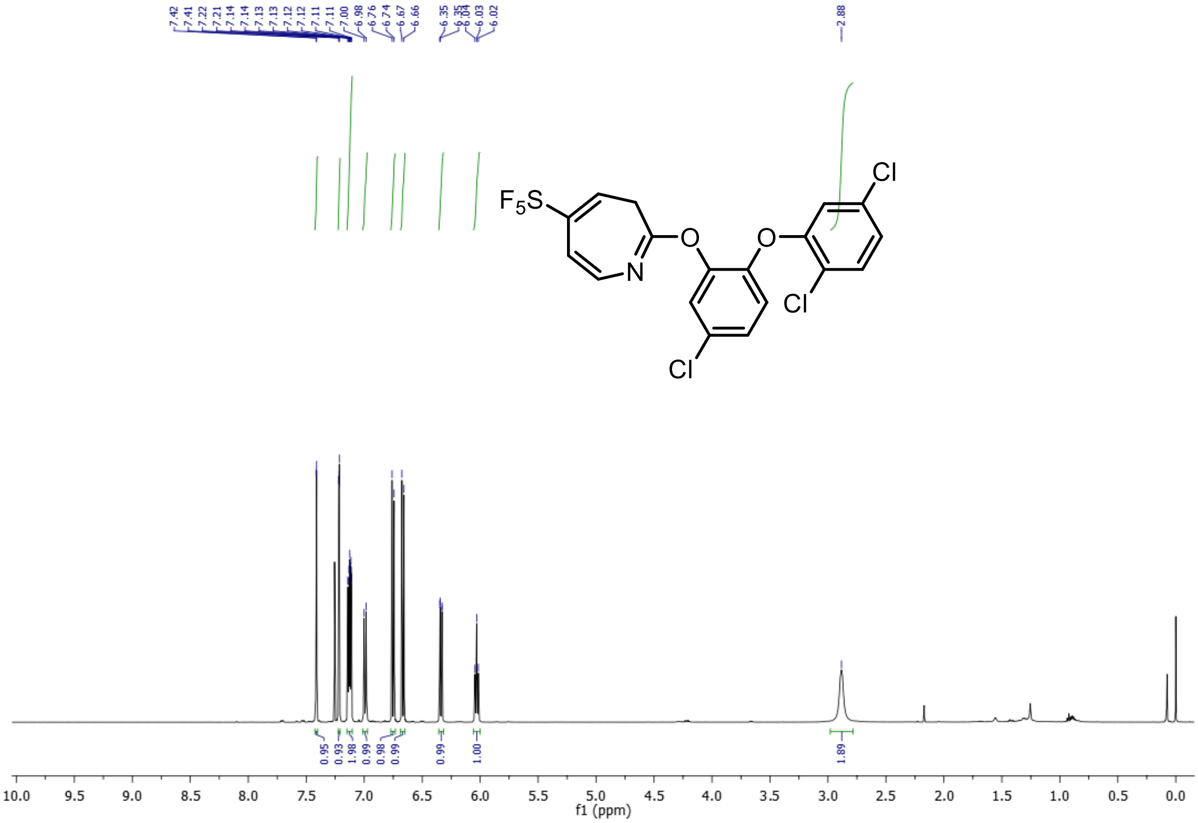

^{13}C NMR (126 MHz, $CDCl_3$) : **3had**

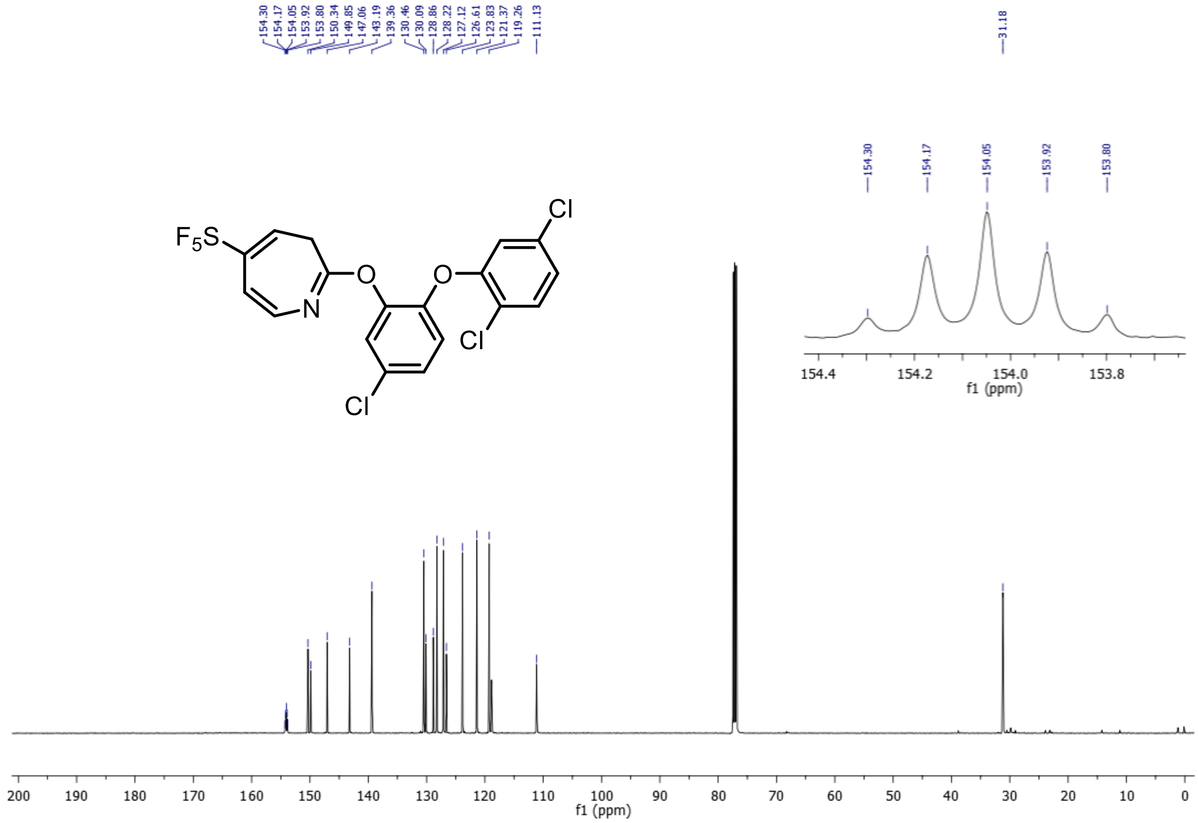

^{19}F NMR (282 MHz, $CDCl_3$) : **3had**

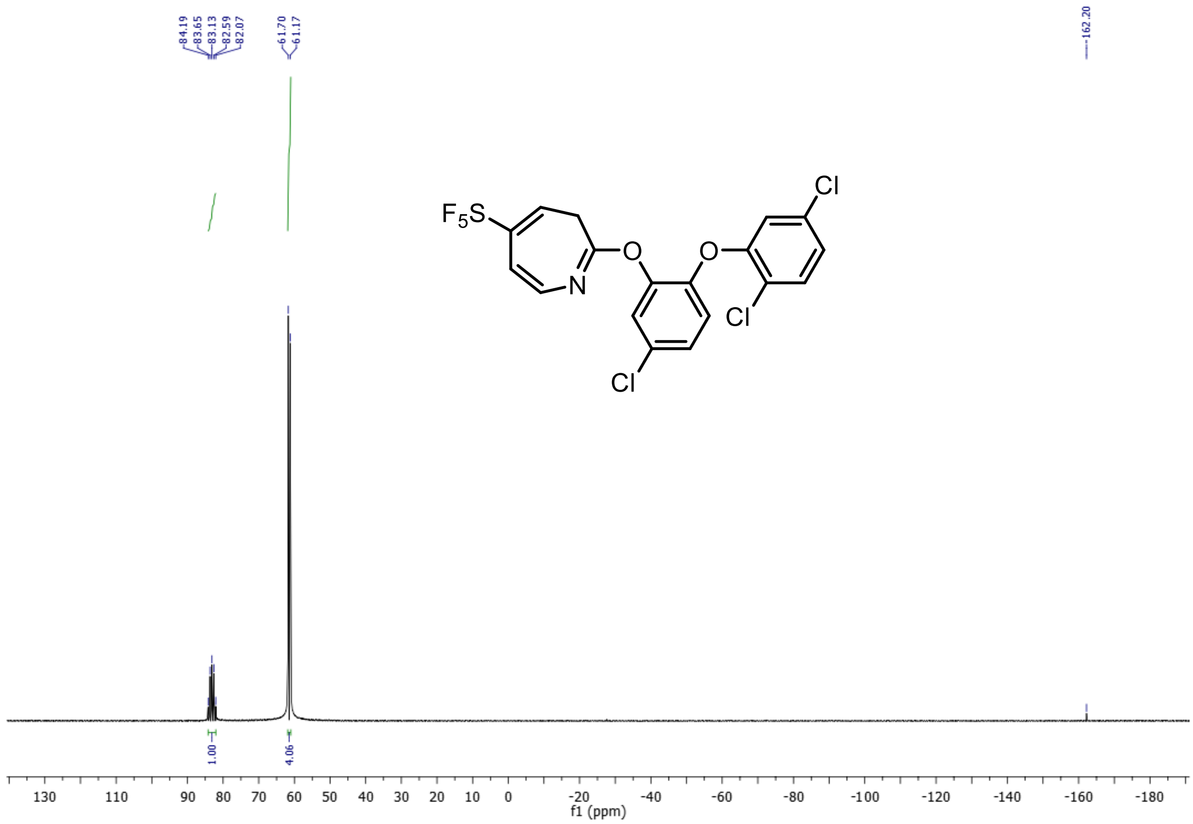

^{1}H NMR (500 MHz, $CDCl_3$) : **3hae**

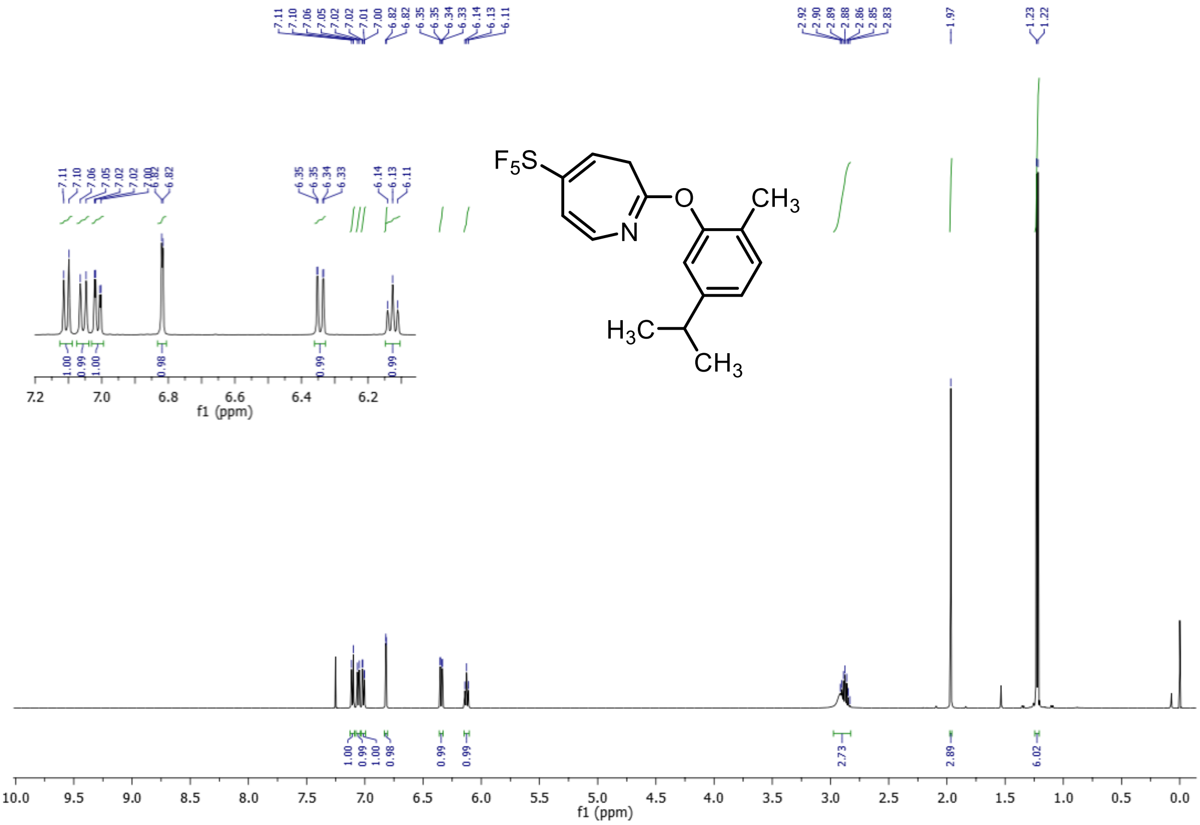

^{13}C NMR (126 MHz, $CDCl_3$) : **3hae**

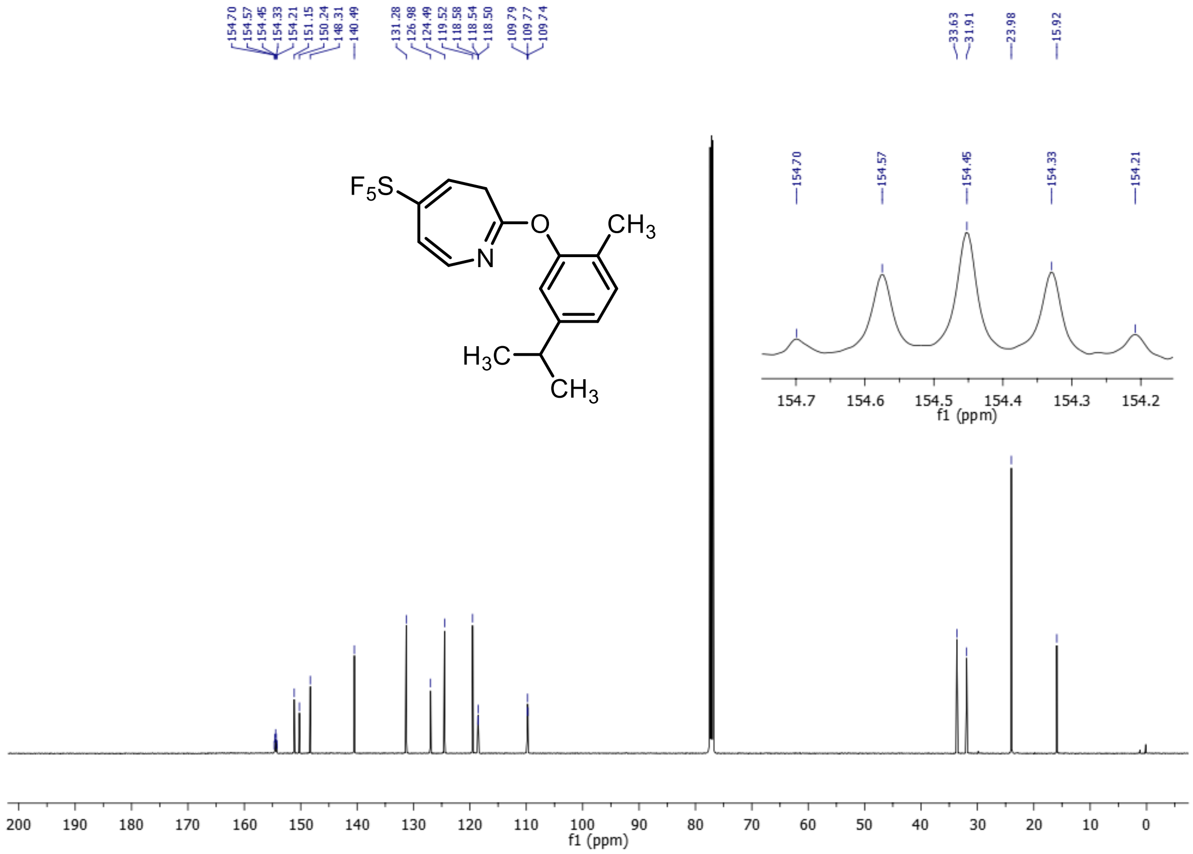

^{19}F NMR (282 MHz, $CDCl_3$) : **3hae**

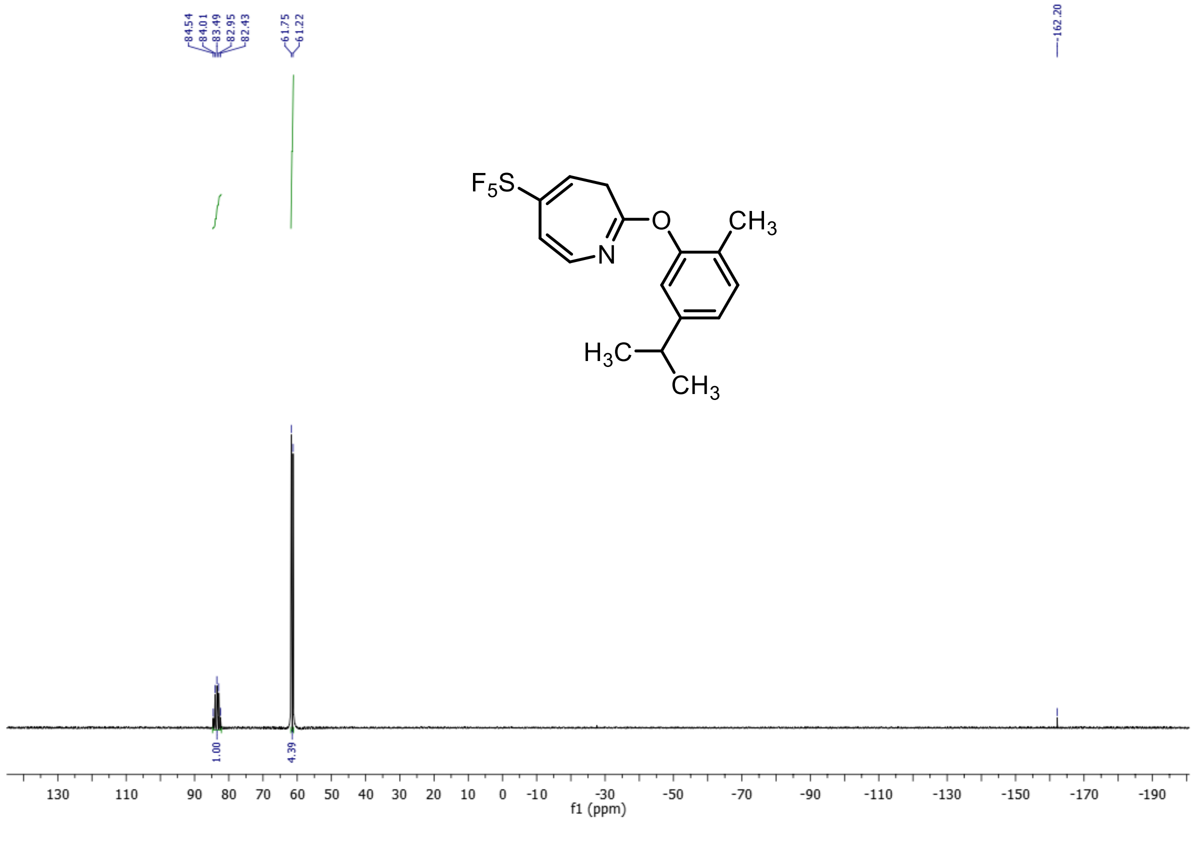

^{1}H NMR (500 MHz, DMSO-d$_6$) : **3haf**

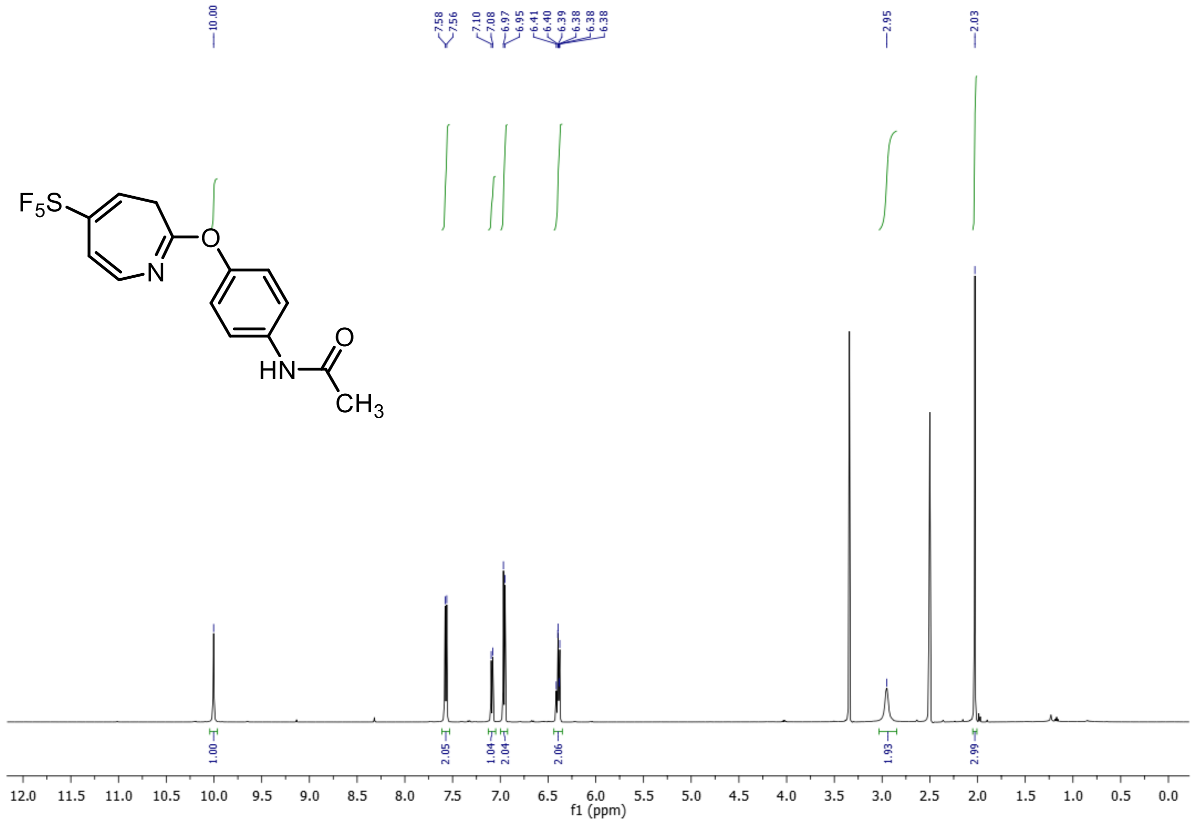

^{13}C NMR (126 MHz, DMSO-d$_6$) : **3haf**

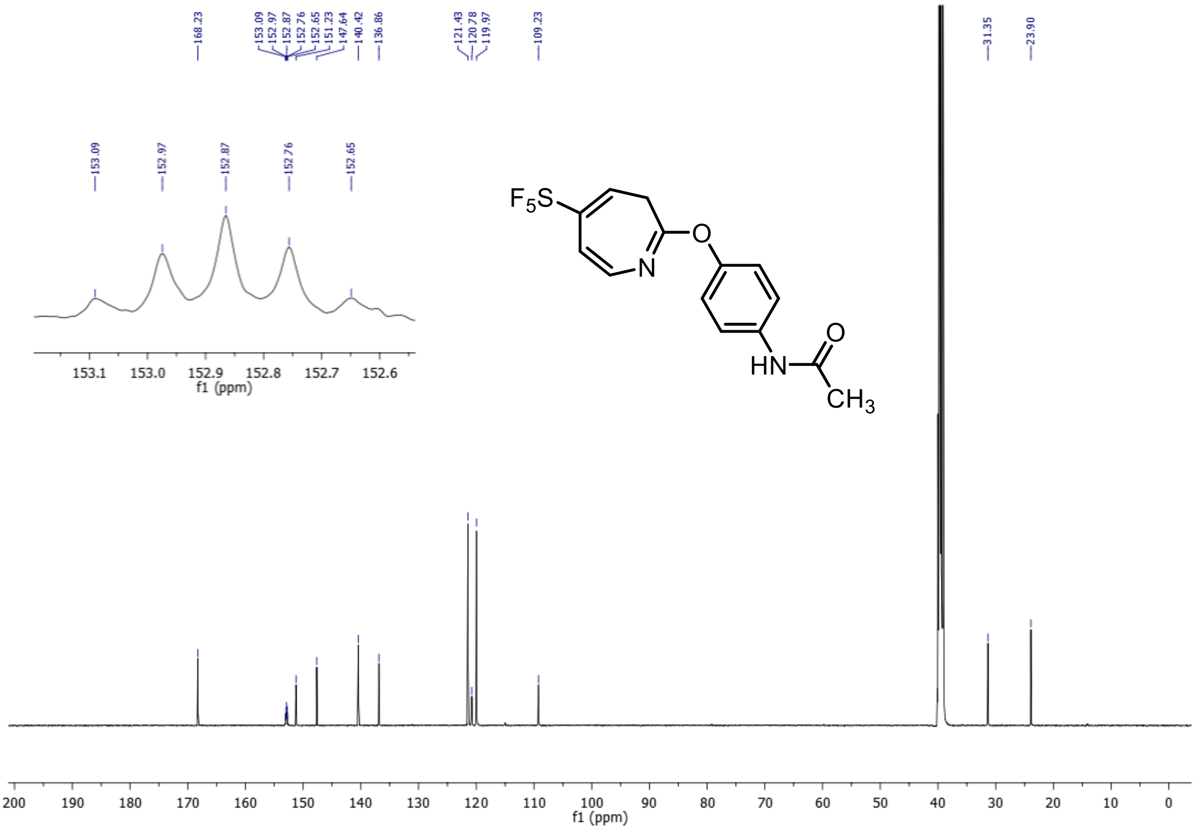

^{19}F NMR (282 MHz, DMSO-d$_6$) : **3haf**

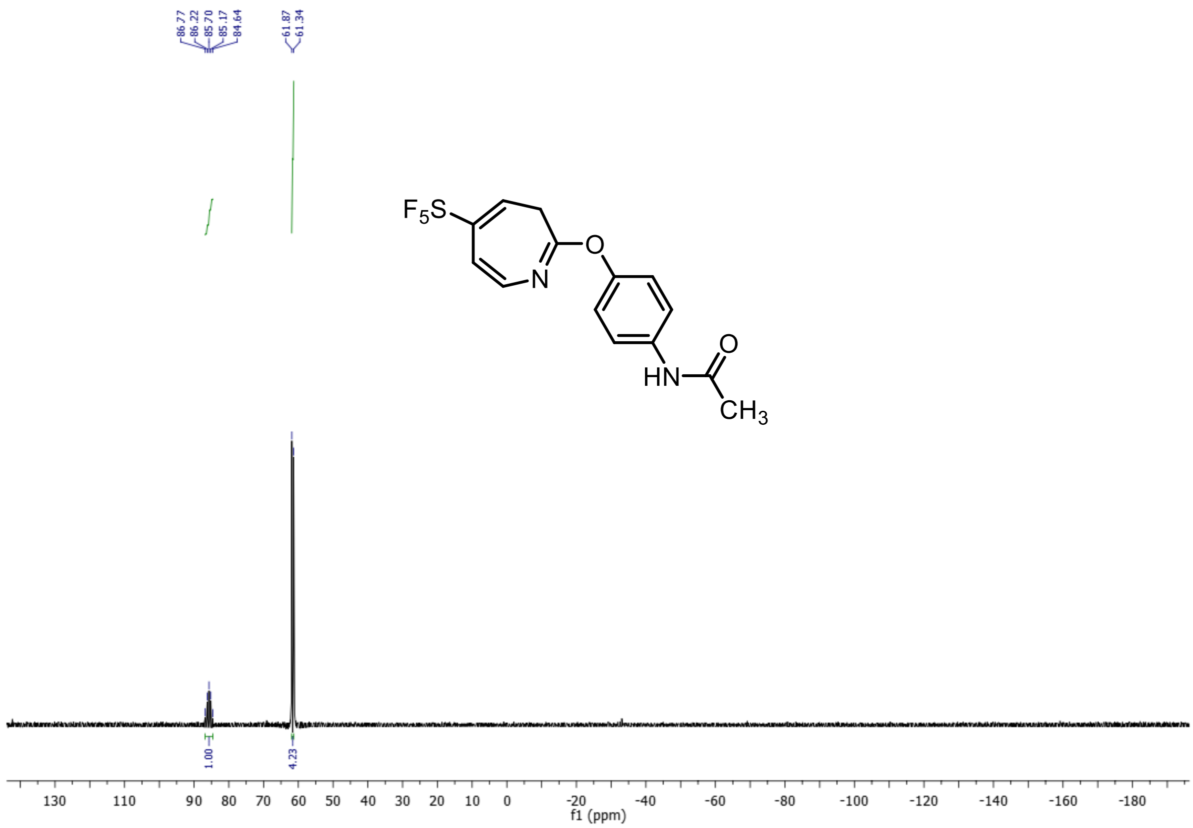

^{1}H NMR (500 MHz, CDCl$_3$) : **3hag**

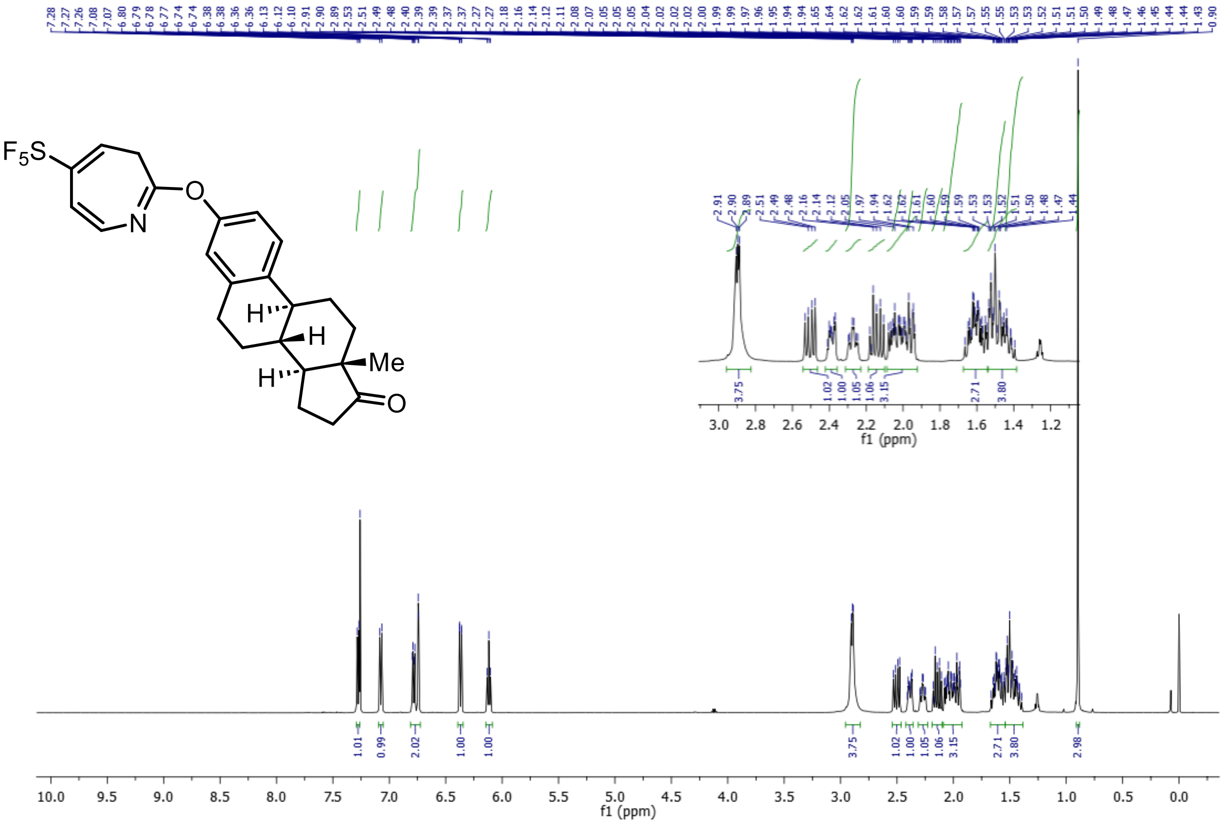

^{13}C NMR (126 MHz, $CDCl_3$) : **3hag**

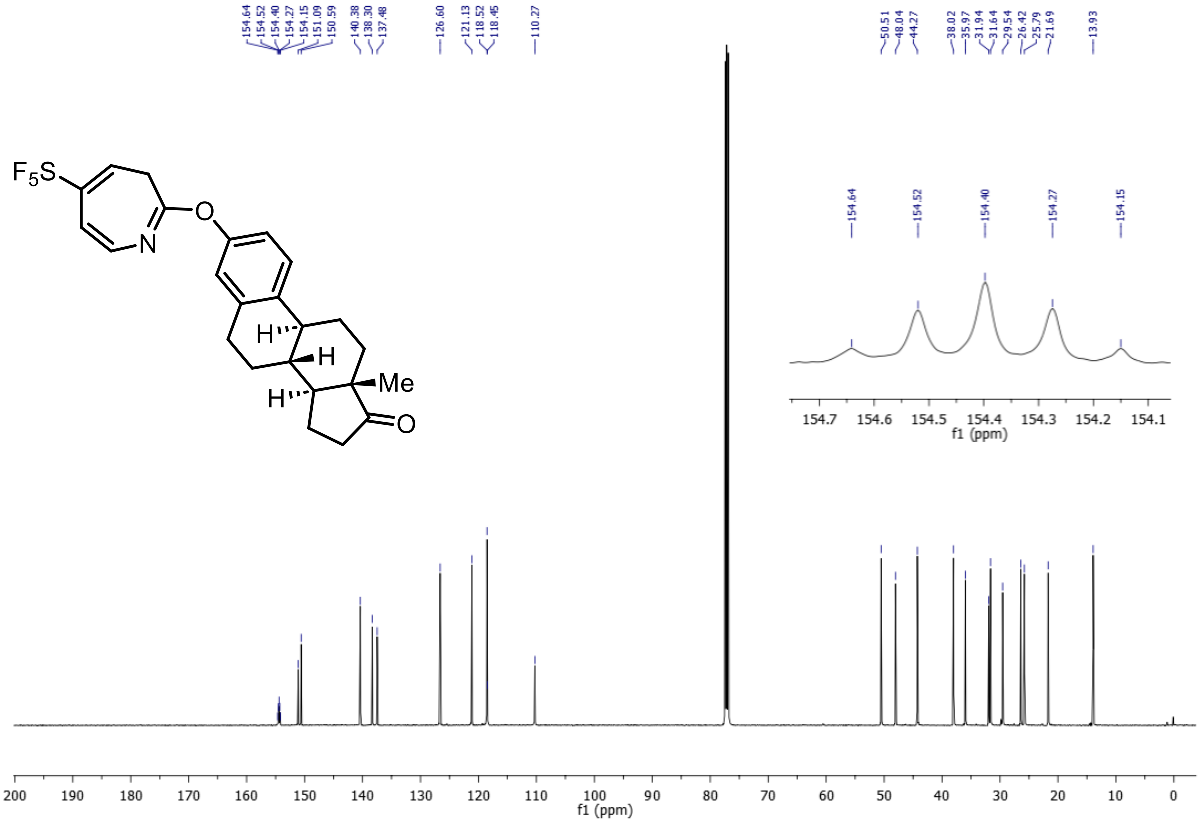

^{19}F NMR (282 MHz, $CDCl_3$) : **3hag**

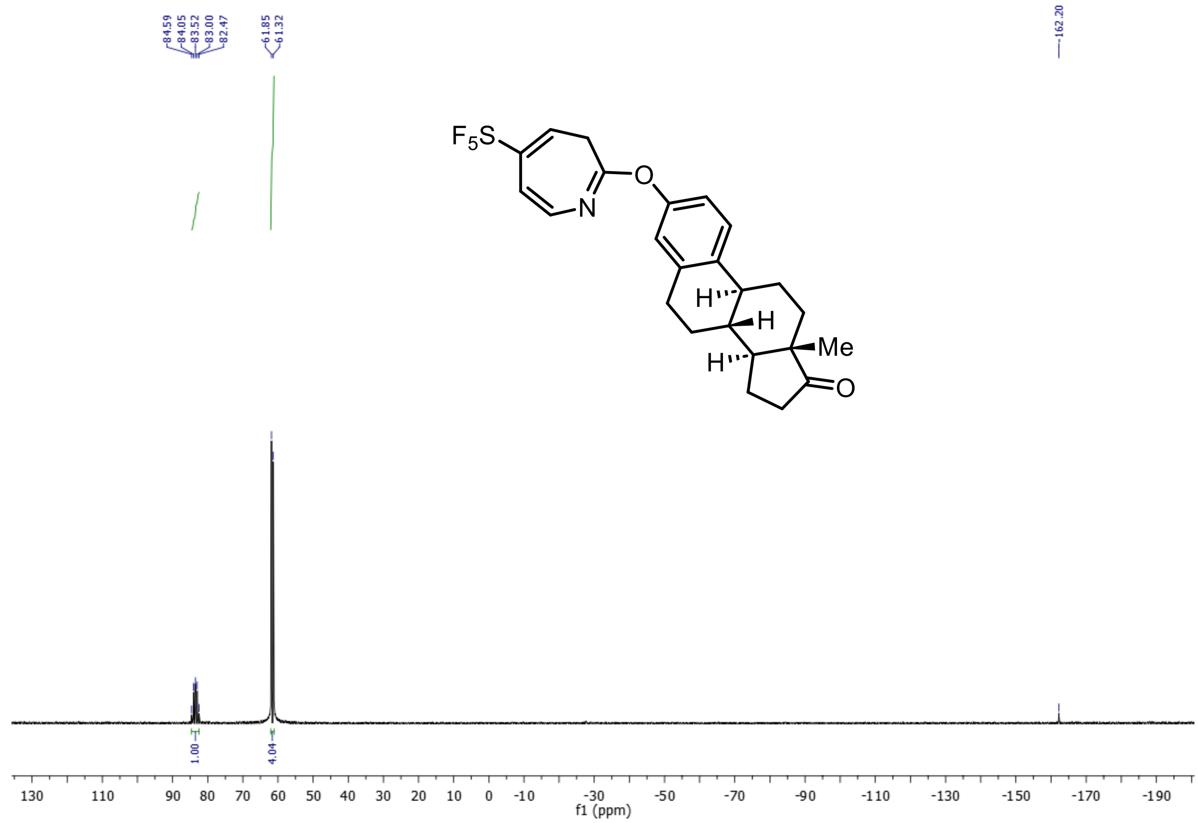

^{1}H NMR (500 MHz, $CDCl_3$) : **6**

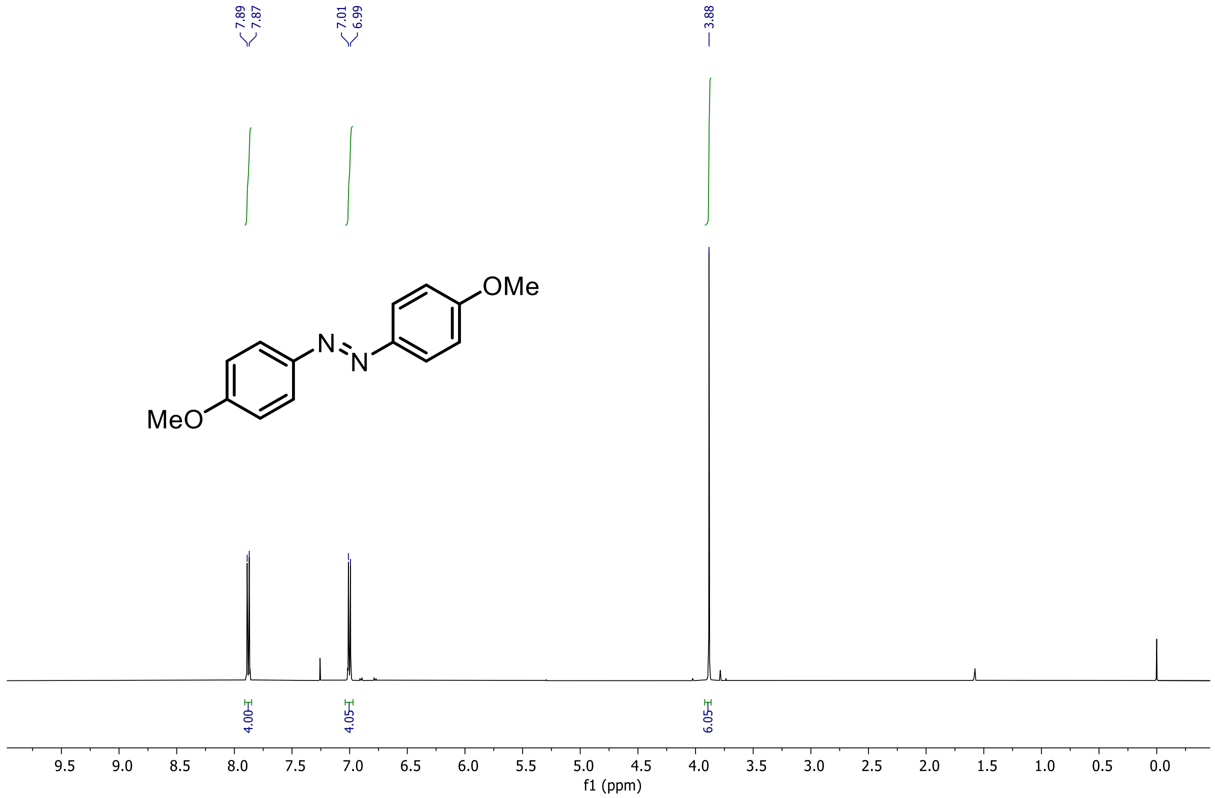

^{13}C NMR (126 MHz, $CDCl_3$) : **6**

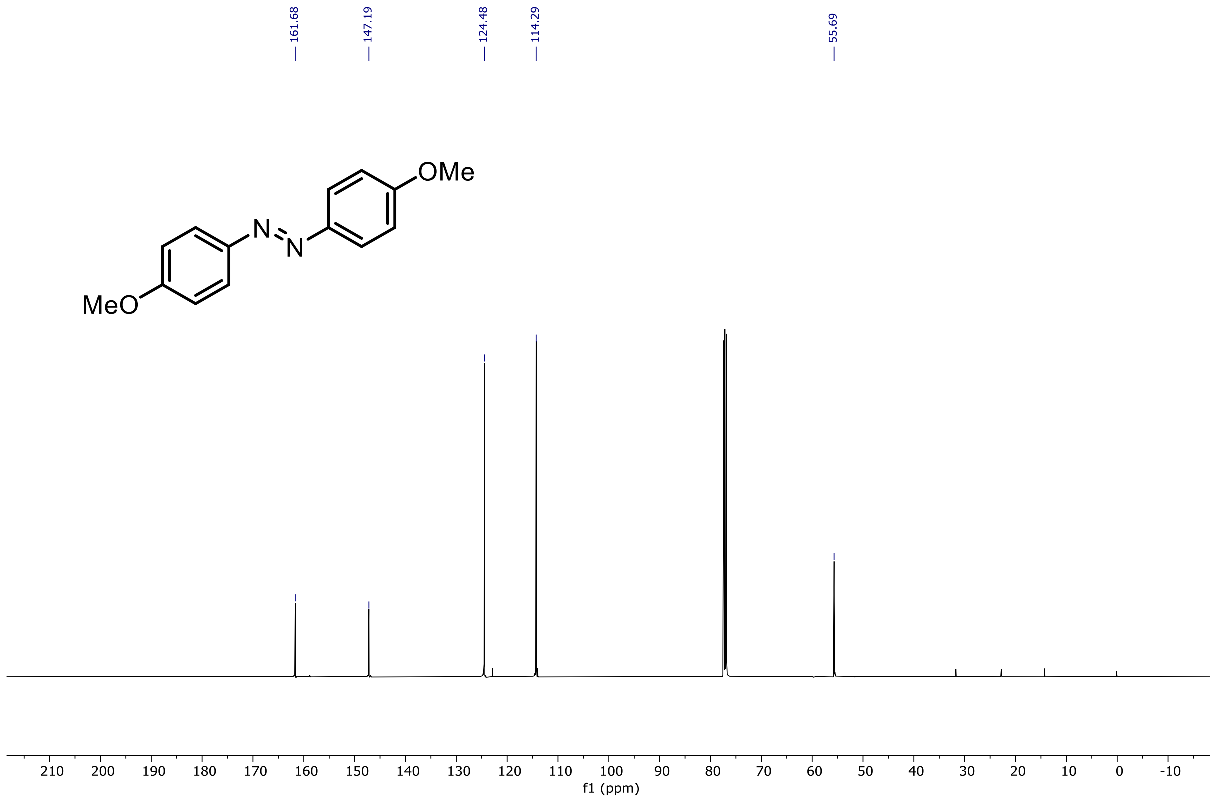

^{1}**H NMR** (500 MHz, $CDCl_3$) : **4h**

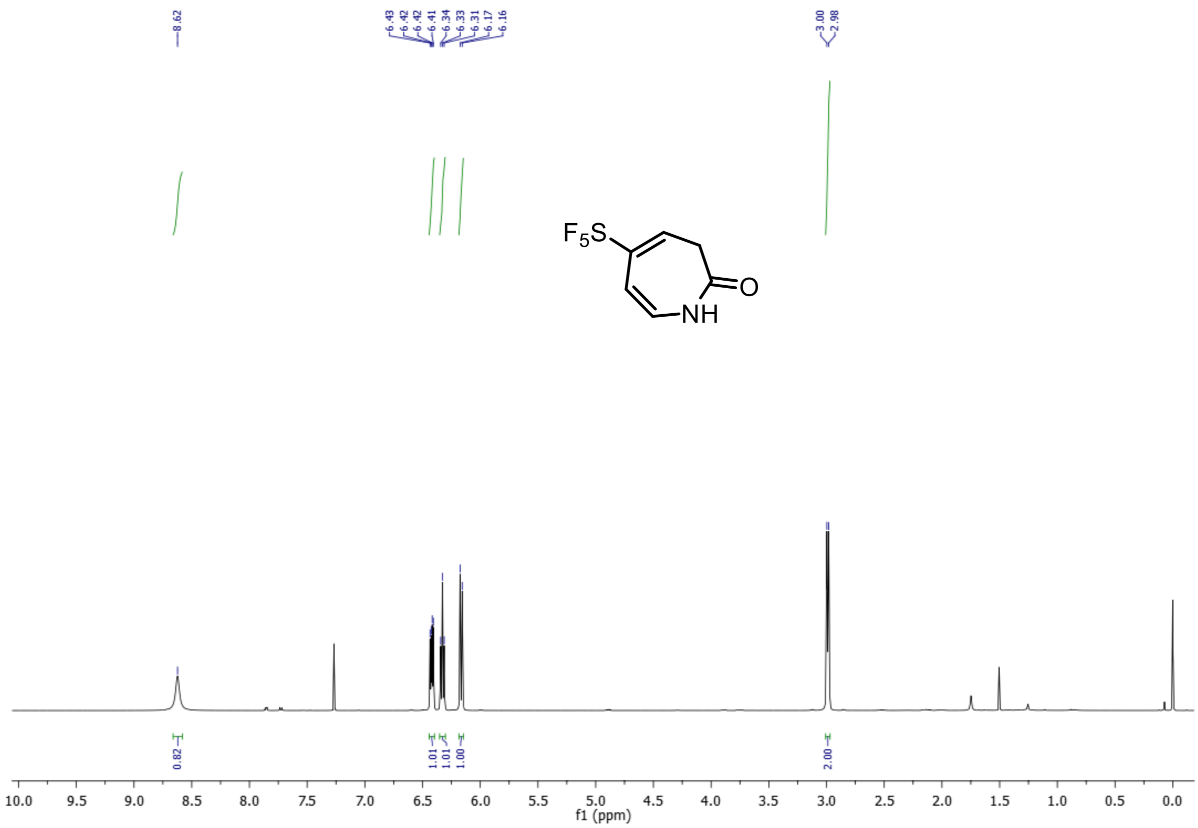

^{13}**C NMR** (126 MHz, $CDCl_3$) : **4h**

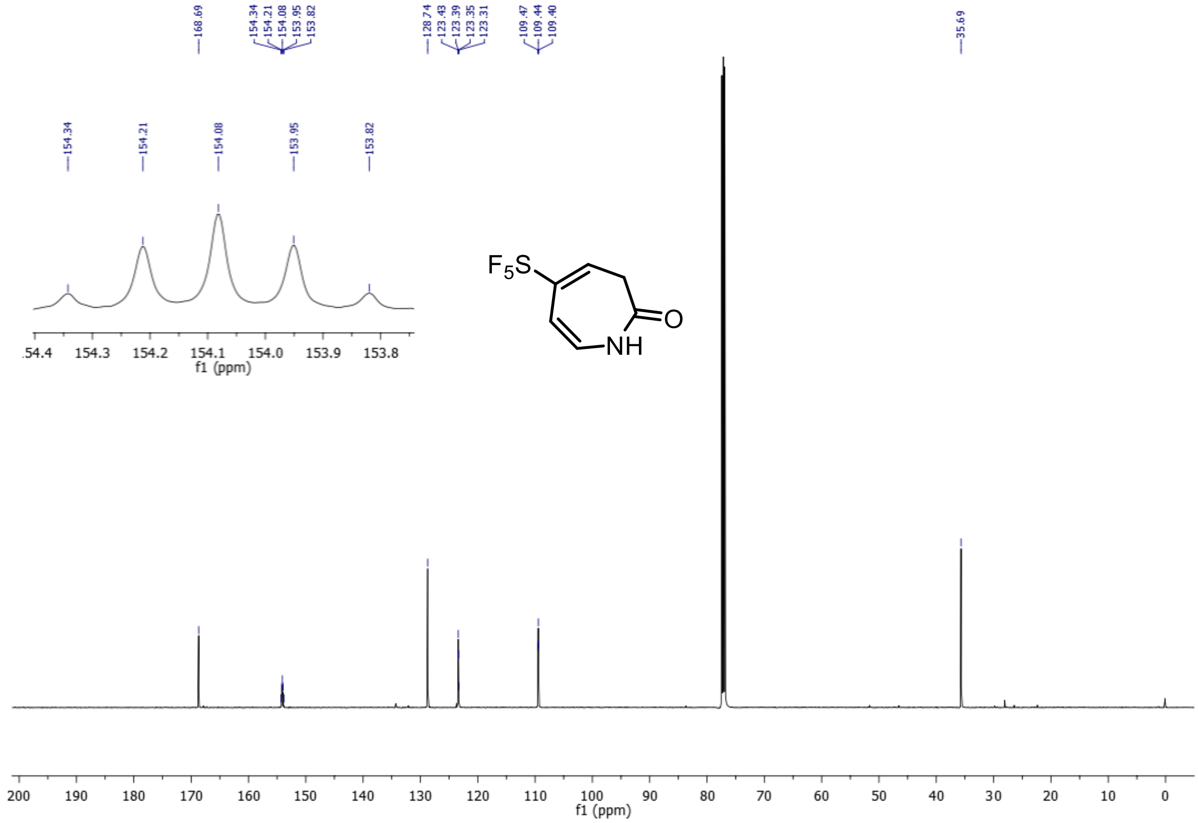

^{19}F NMR (376 MHz, CDCl$_3$) : **4h**

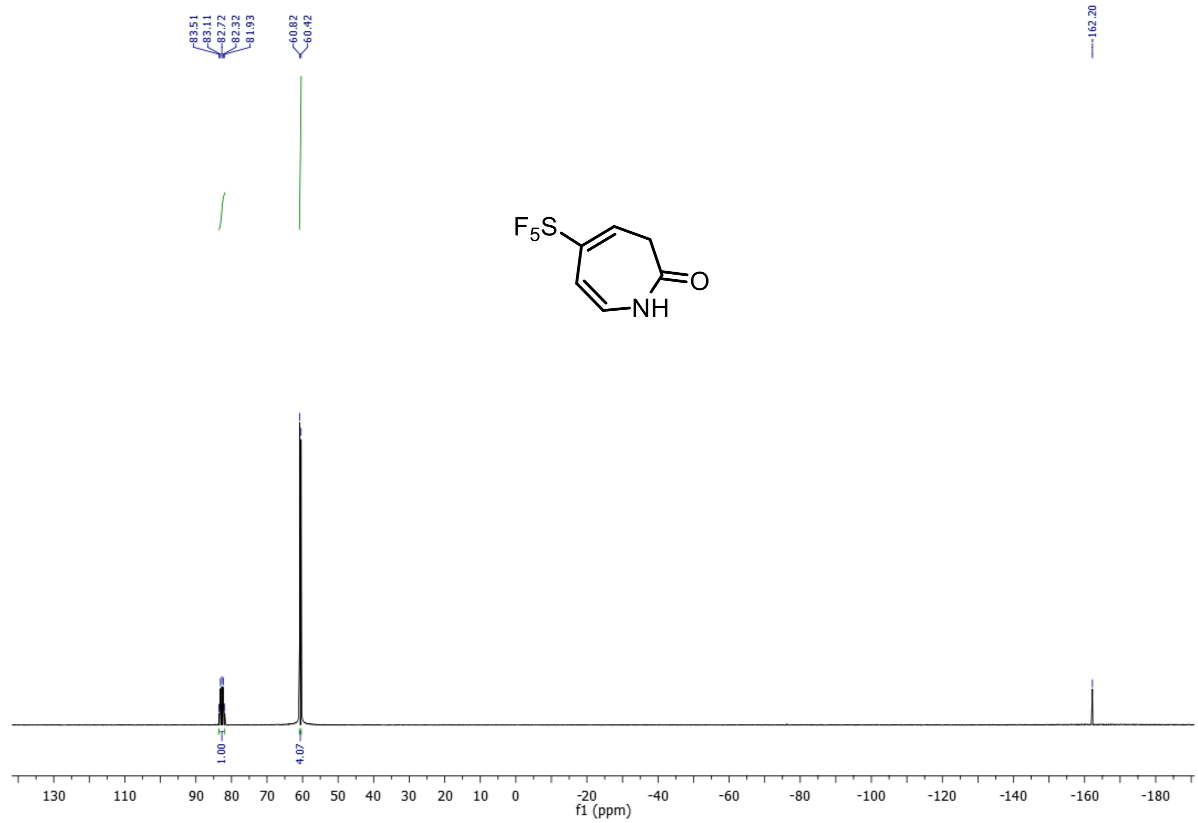

^{1}H NMR (500 MHz, CDCl$_3$) : **5ha**

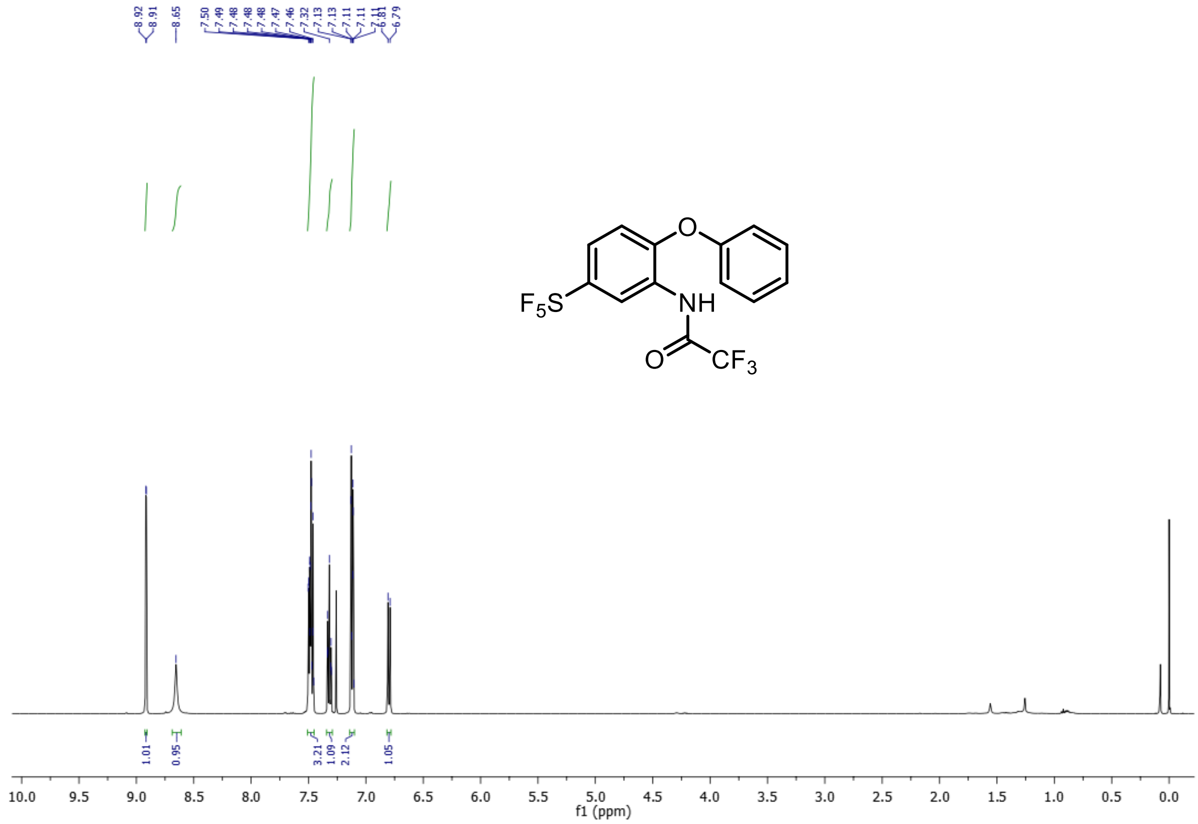

^{13}C NMR (126 MHz, $CDCl_3$) : **5ha**

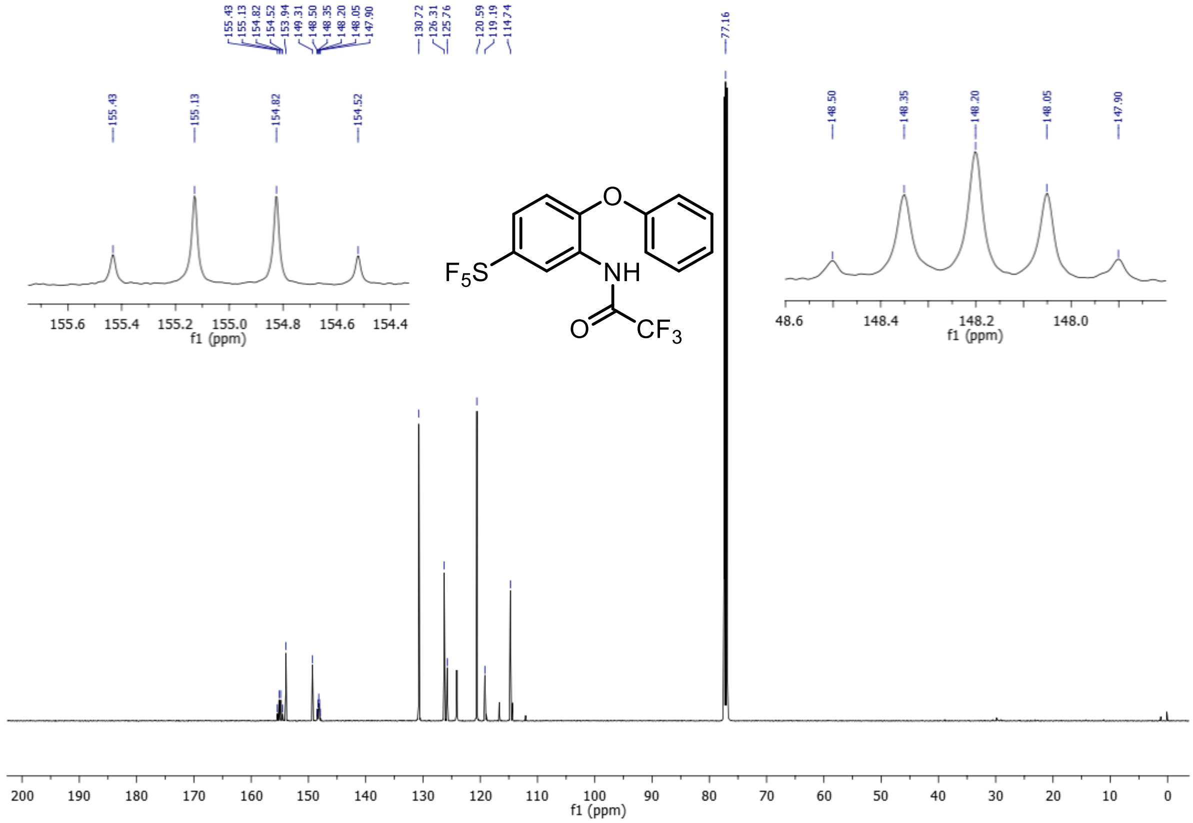

^{19}F NMR (376 MHz, $CDCl_3$) : **5ha**

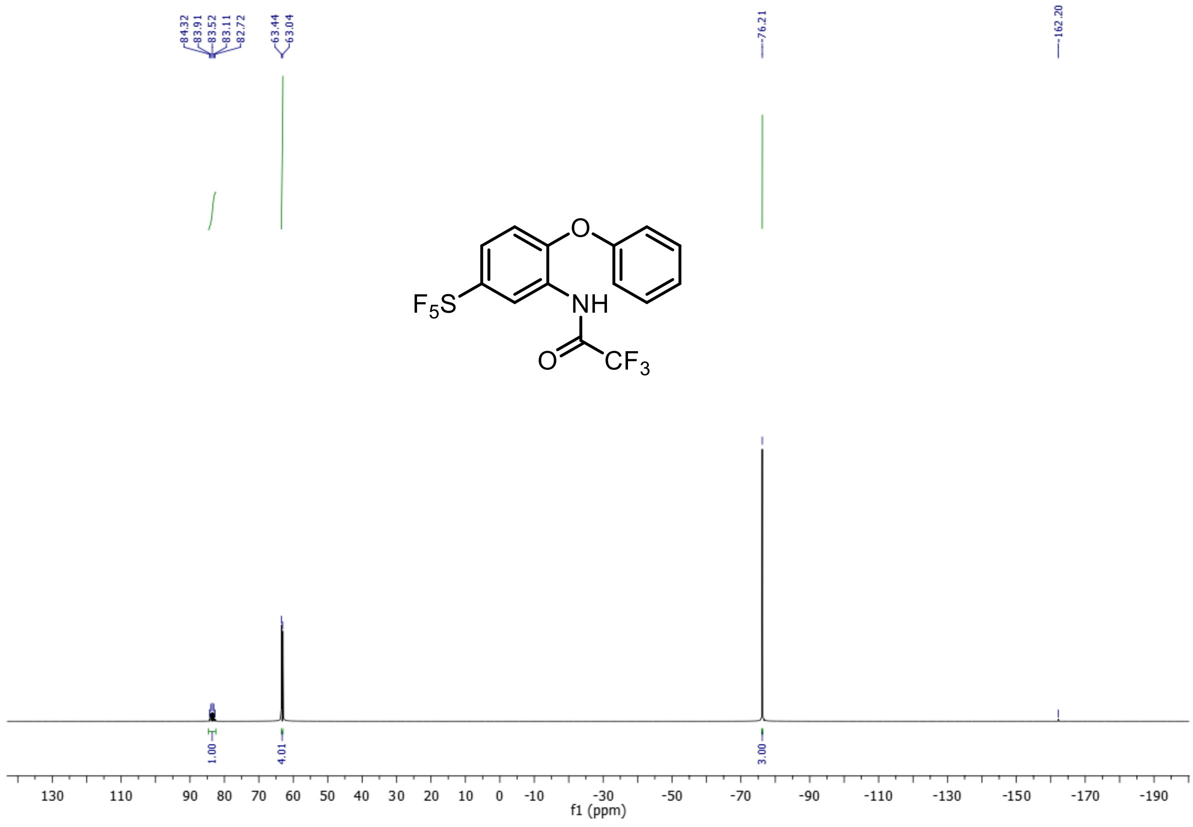

^{1}H NMR (500 MHz, $CDCl_3$) : **5hb**

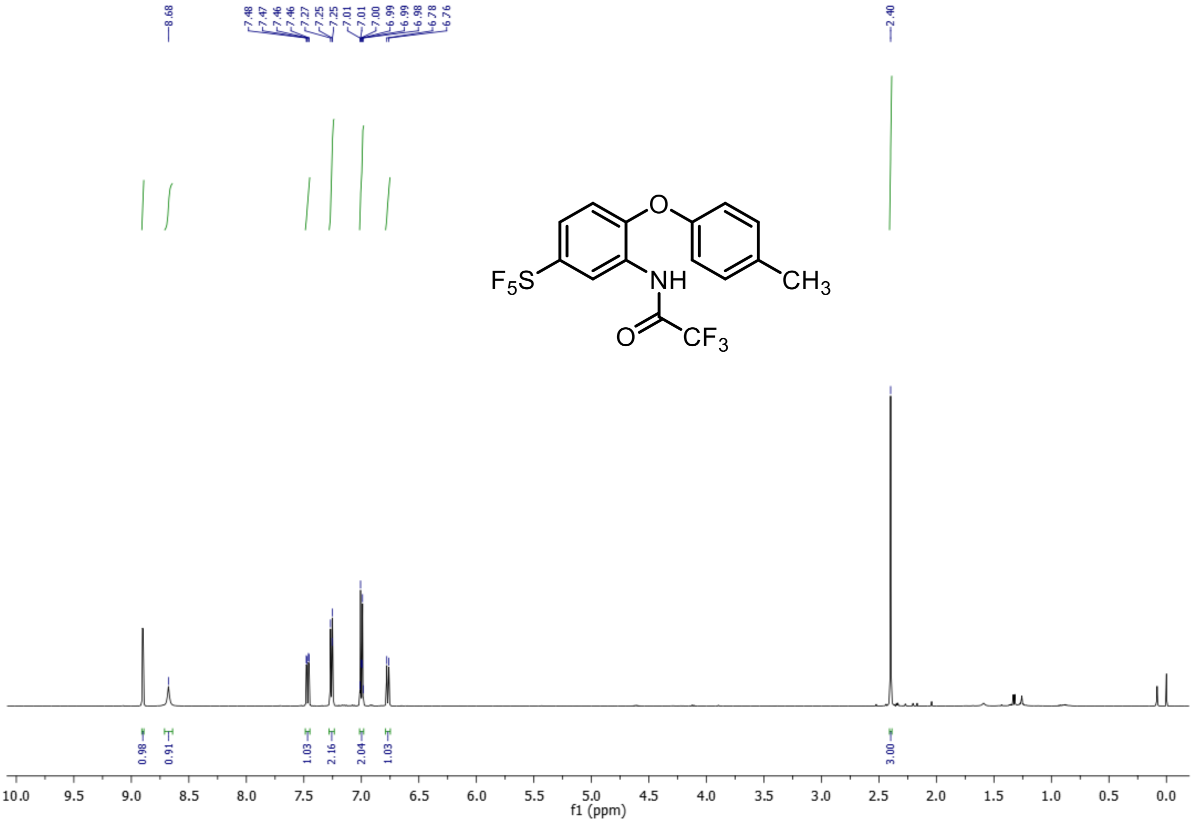

^{13}C NMR (126 MHz, $CDCl_3$) : **5hb**

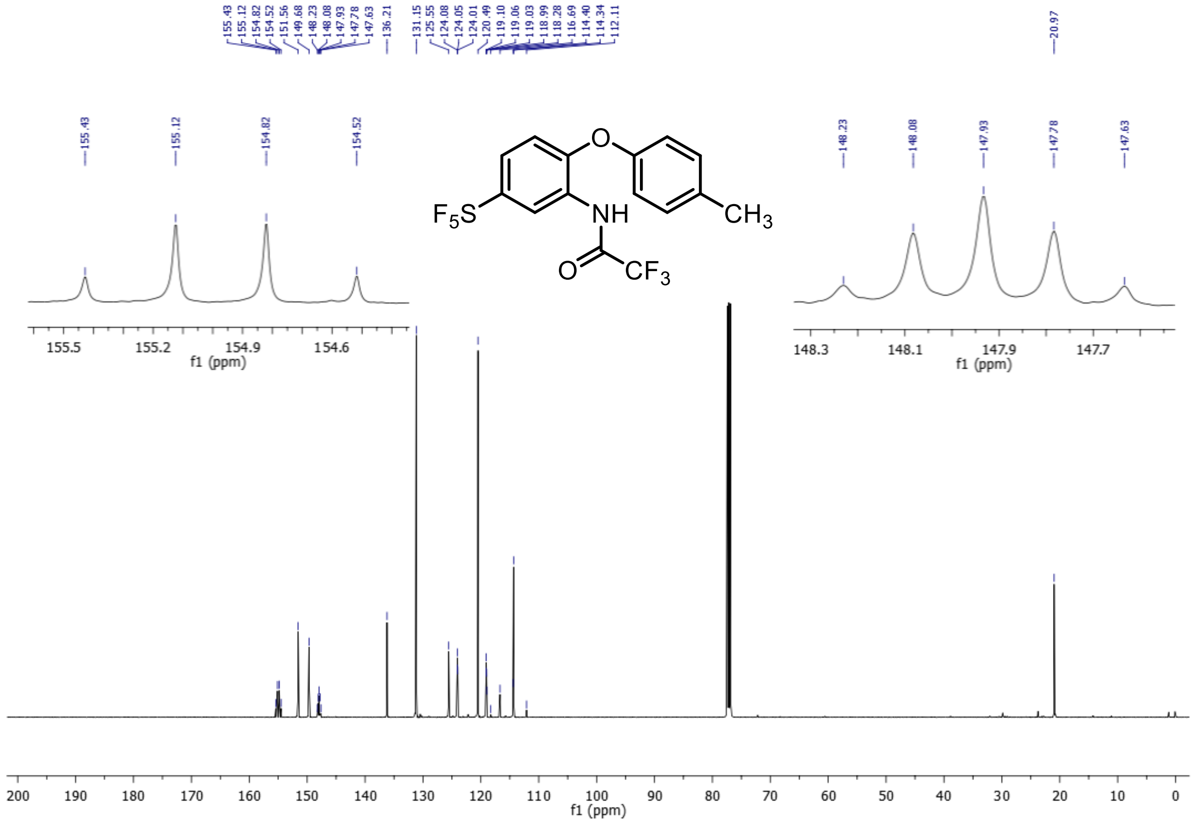

^{19}F NMR (376 MHz, CDCl3) : **5hb**

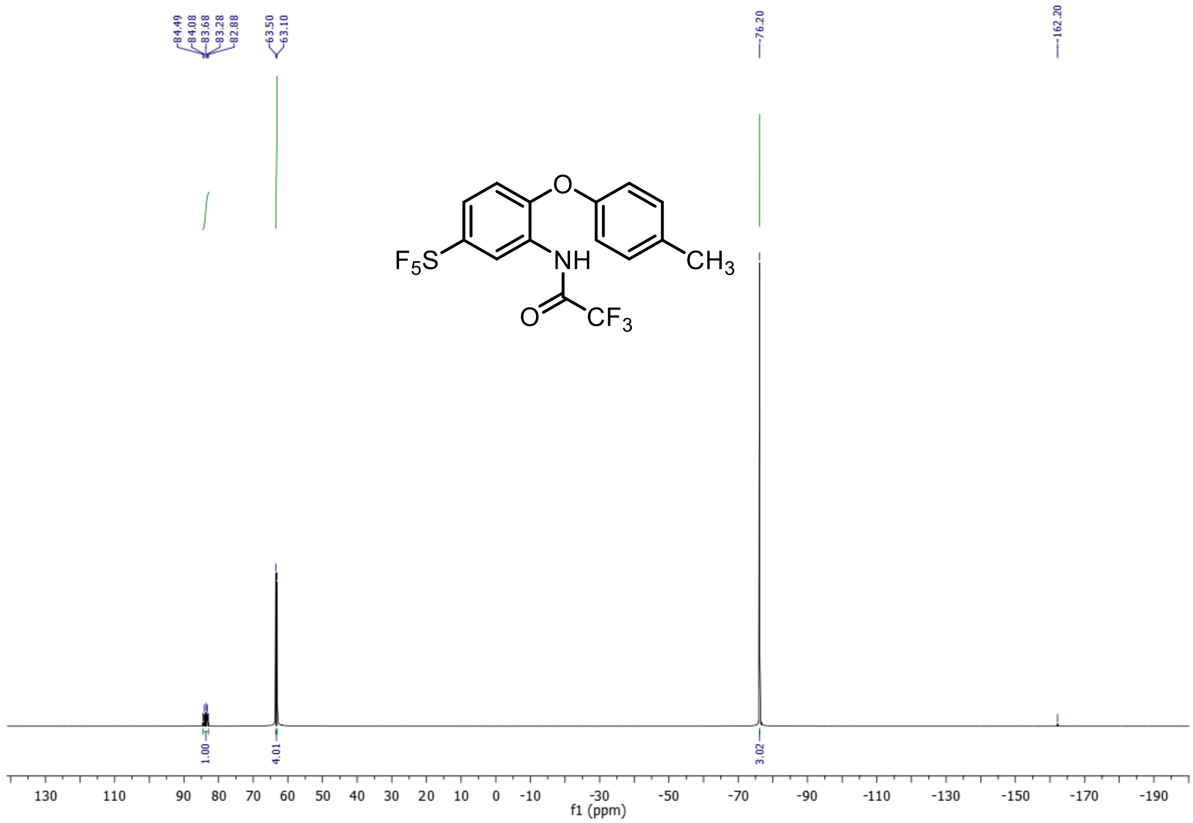

^{1}H NMR (500 MHz, CDCl3) : **5hc**

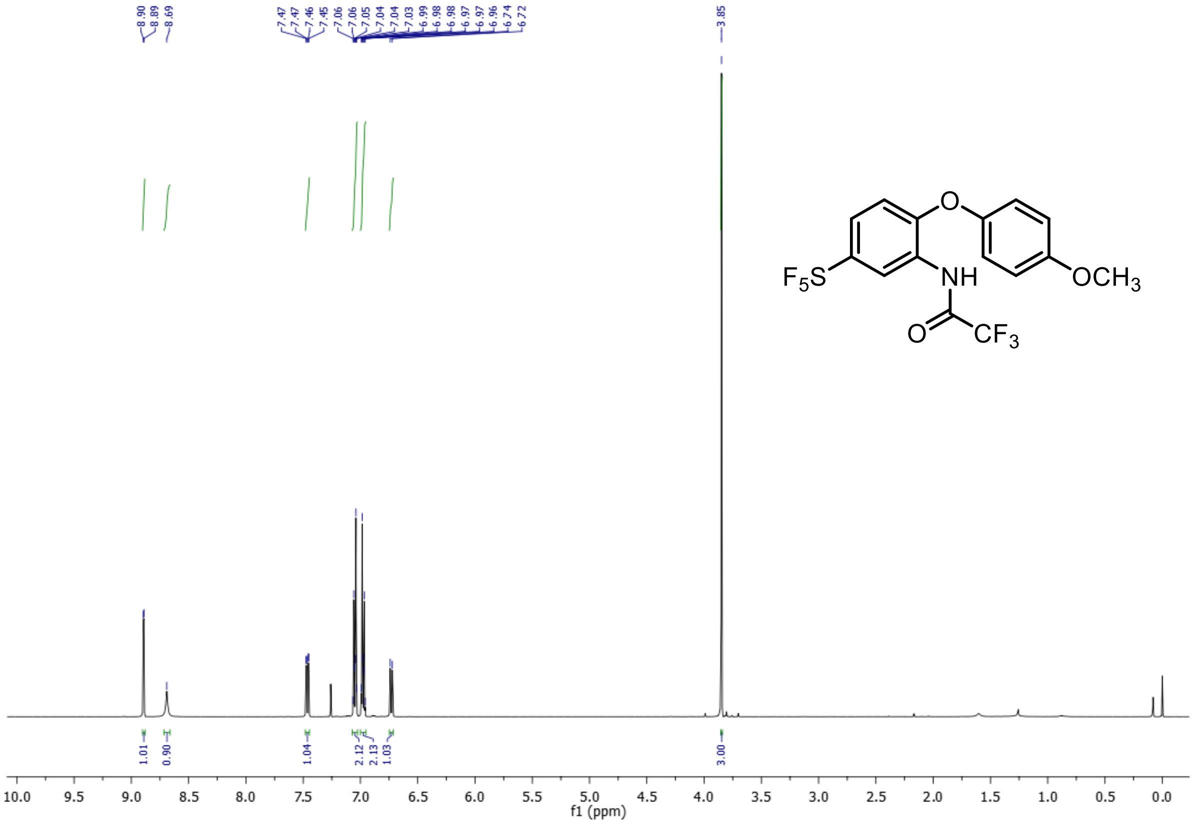

^{13}C NMR (126 MHz, $CDCl_3$) : **5hc**

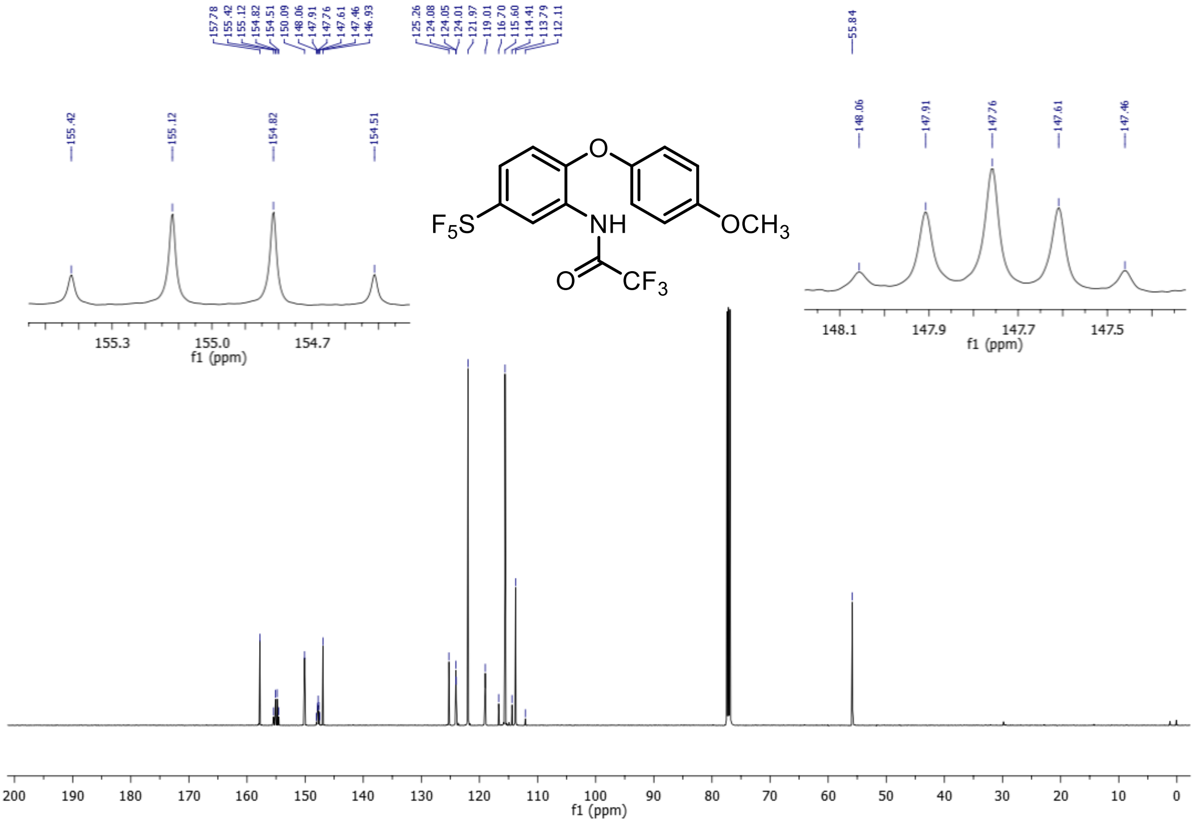

^{19}F NMR (376 MHz, $CDCl_3$) : **5hc**

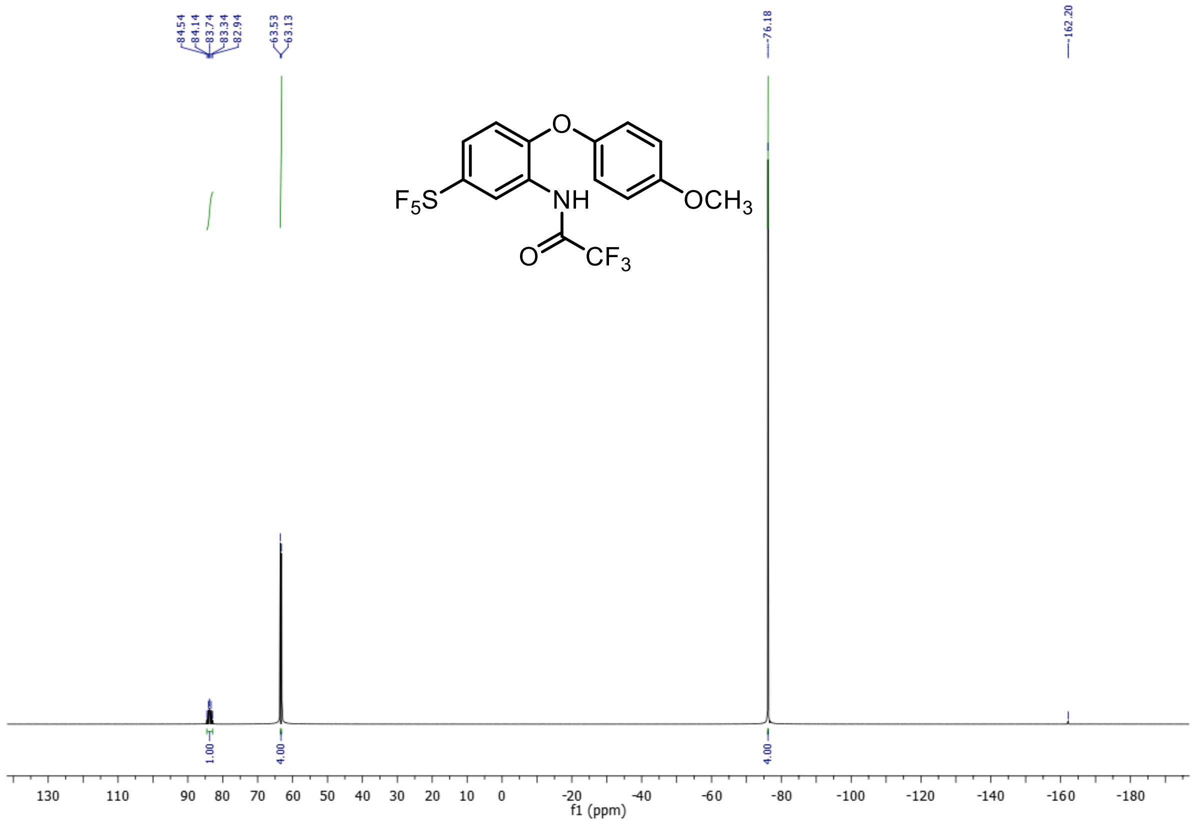

^{1}H NMR (500 MHz, $CDCl_3$) : **5hd**

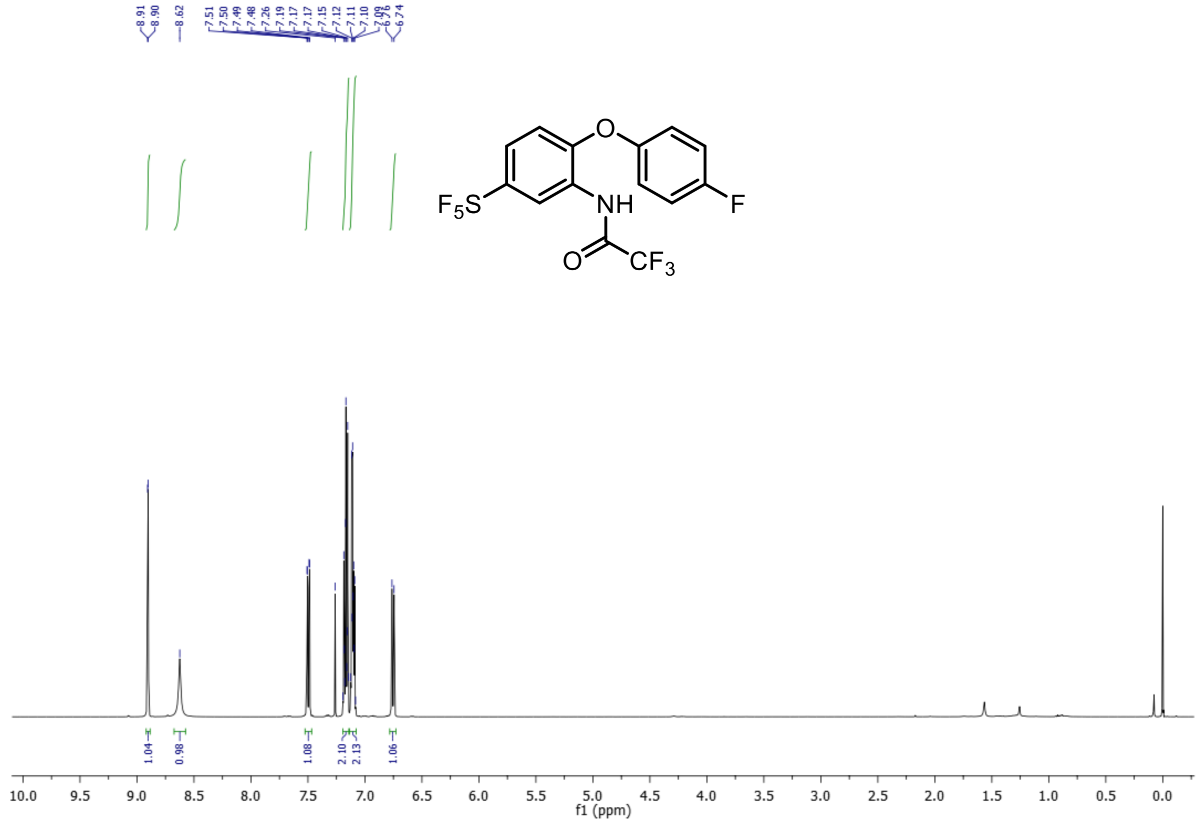

^{13}C NMR (126 MHz, $CDCl_3$) : **5hd**

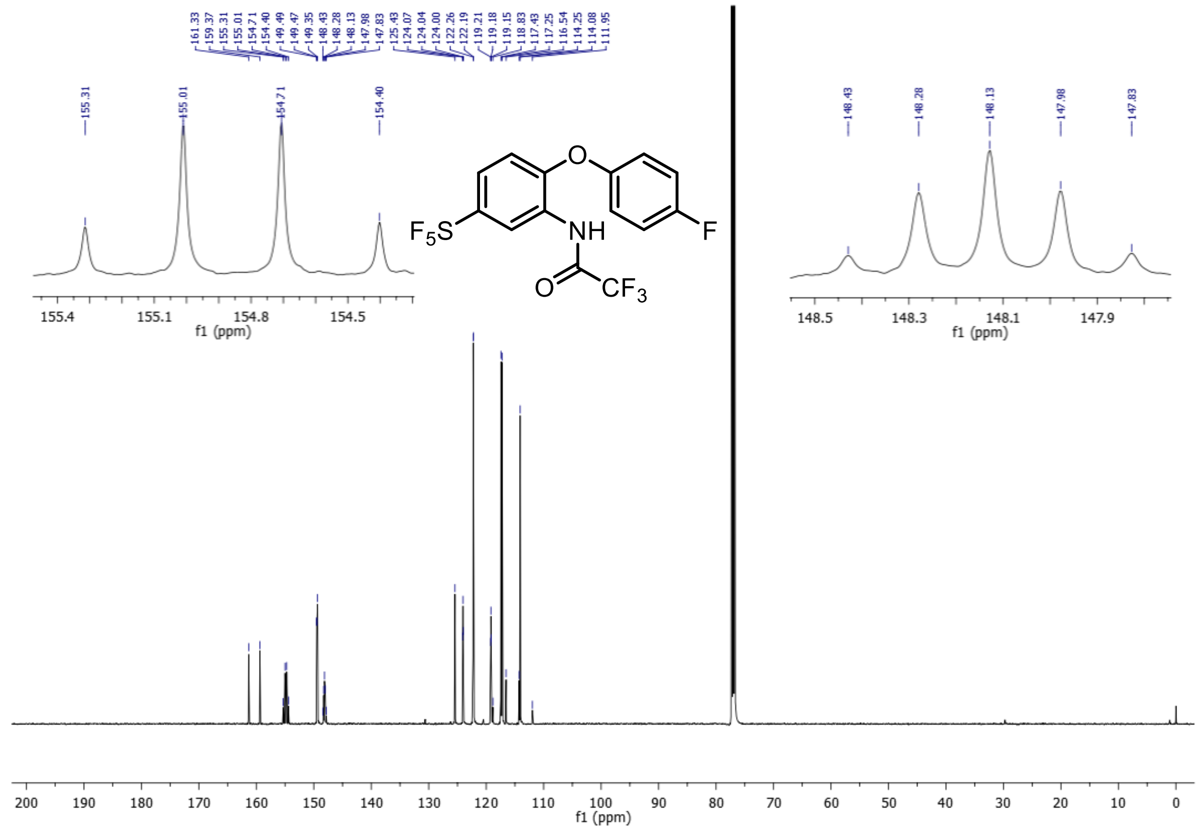

^{19}F NMR (376 MHz, $CDCl_3$) : **5hd**

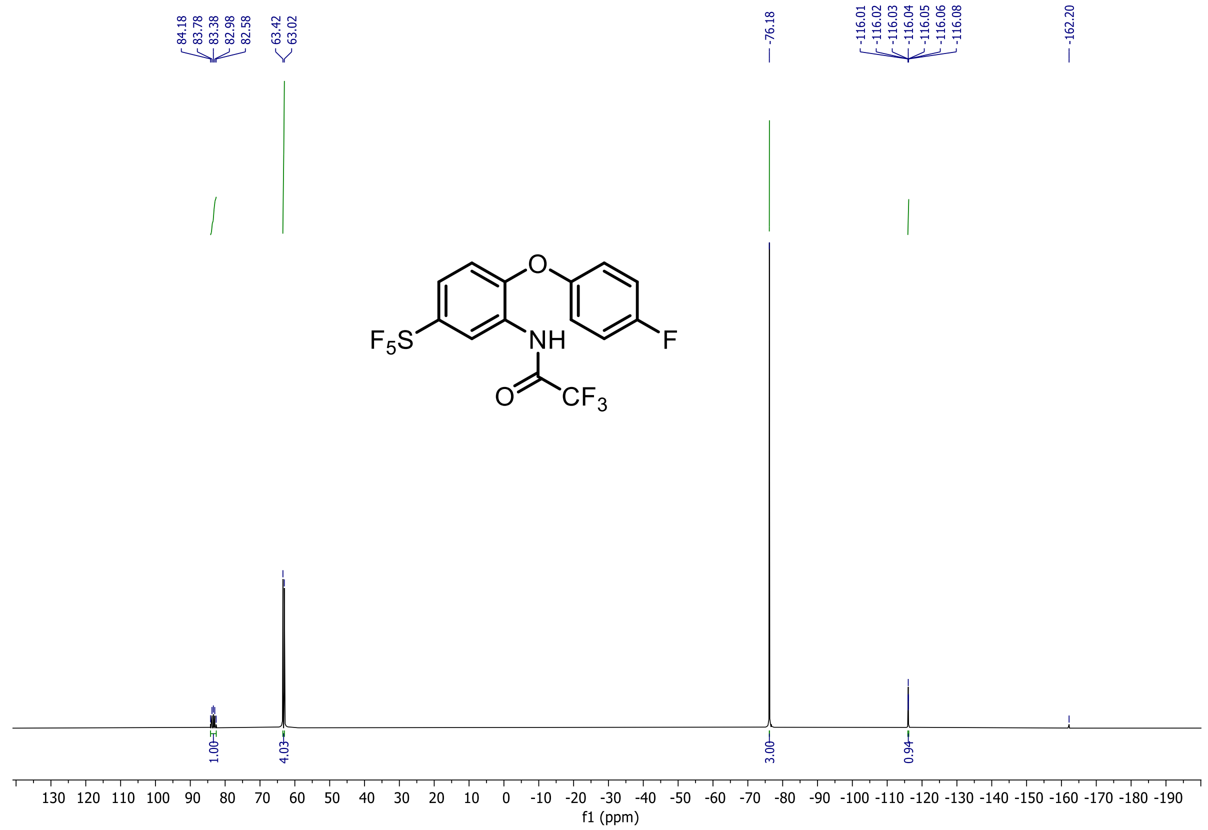

^{1}H NMR (500 MHz, $CDCl_3$) : **5he**

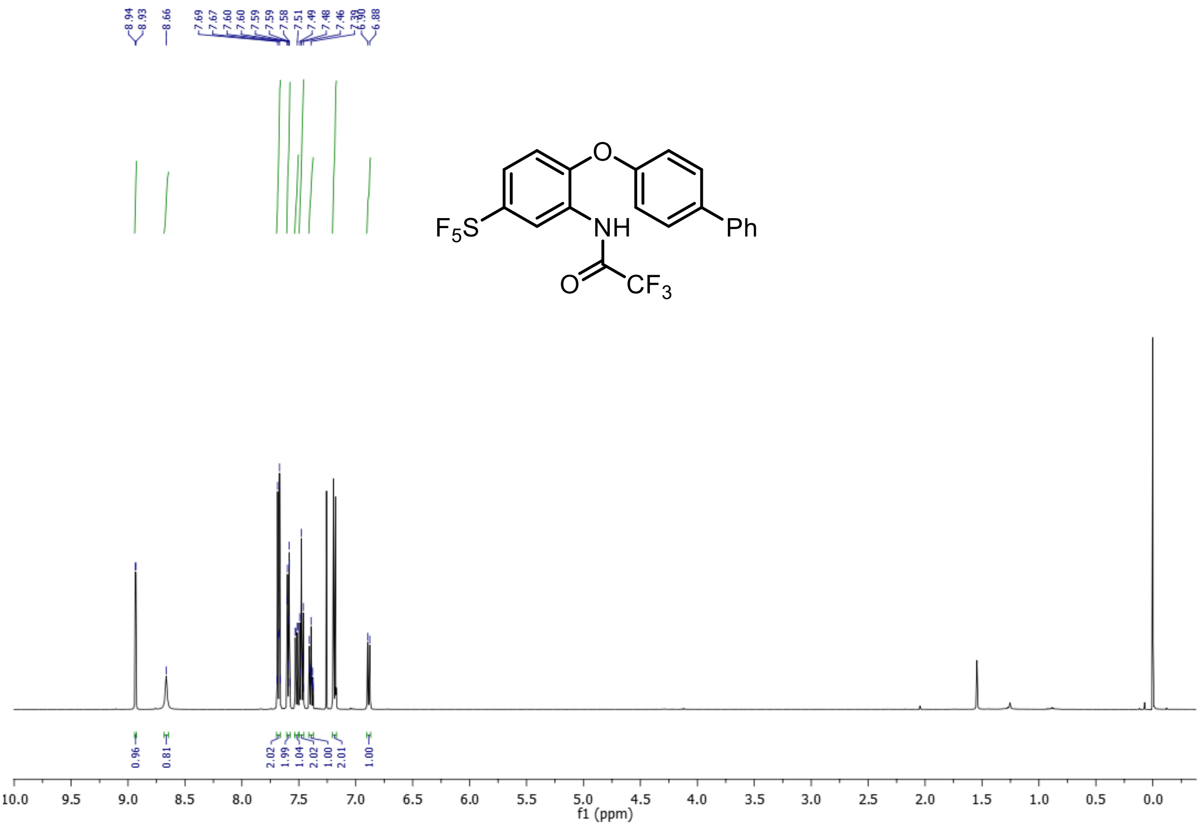

^{13}C NMR (126 MHz, $CDCl_3$) : **5he**

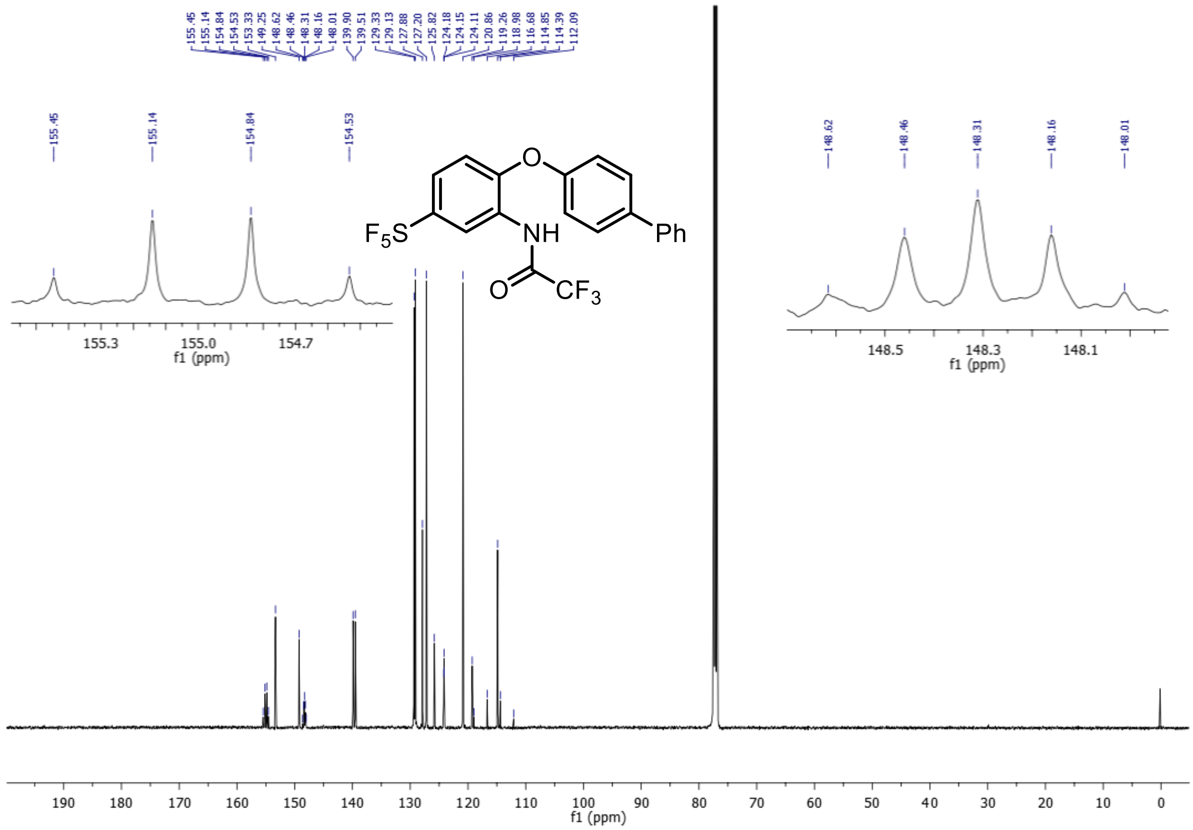

^{19}F NMR (376 MHz, $CDCl_3$) : **5he**

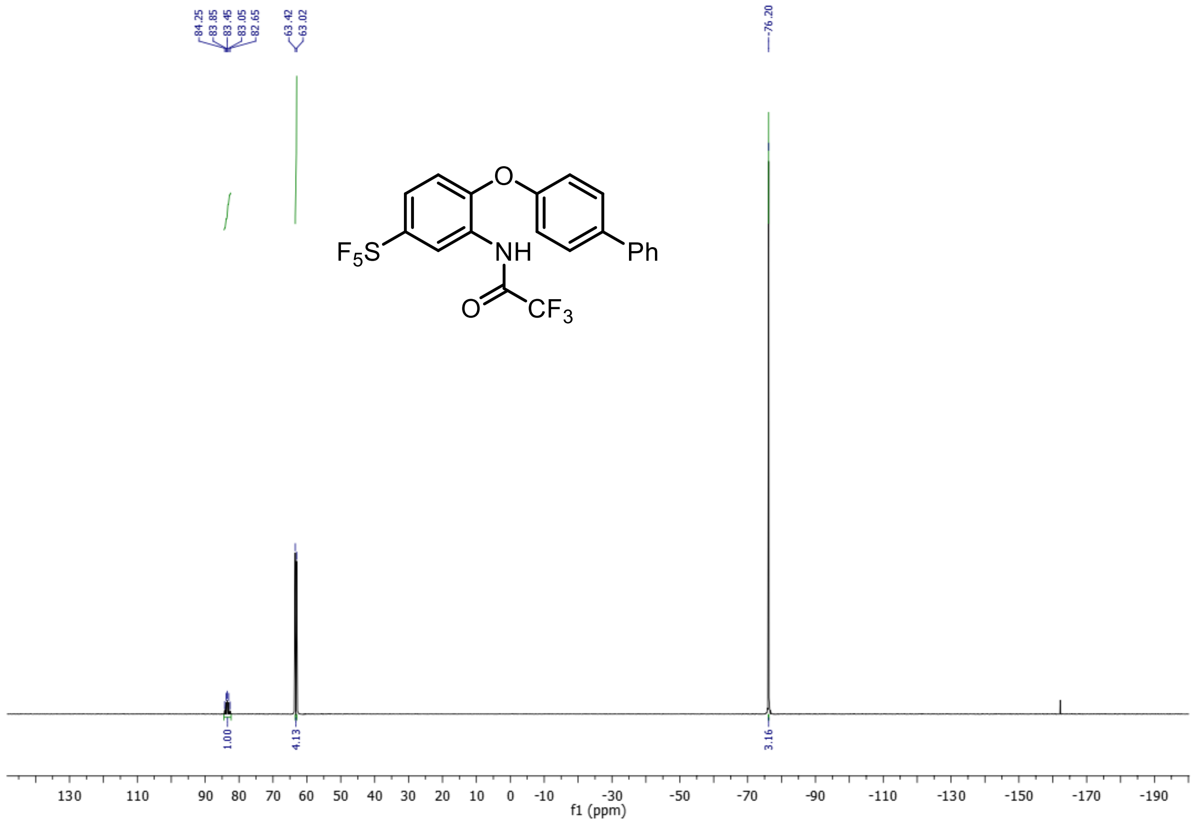

^{1}H NMR (500 MHz, $CDCl_3$) : **5aa**

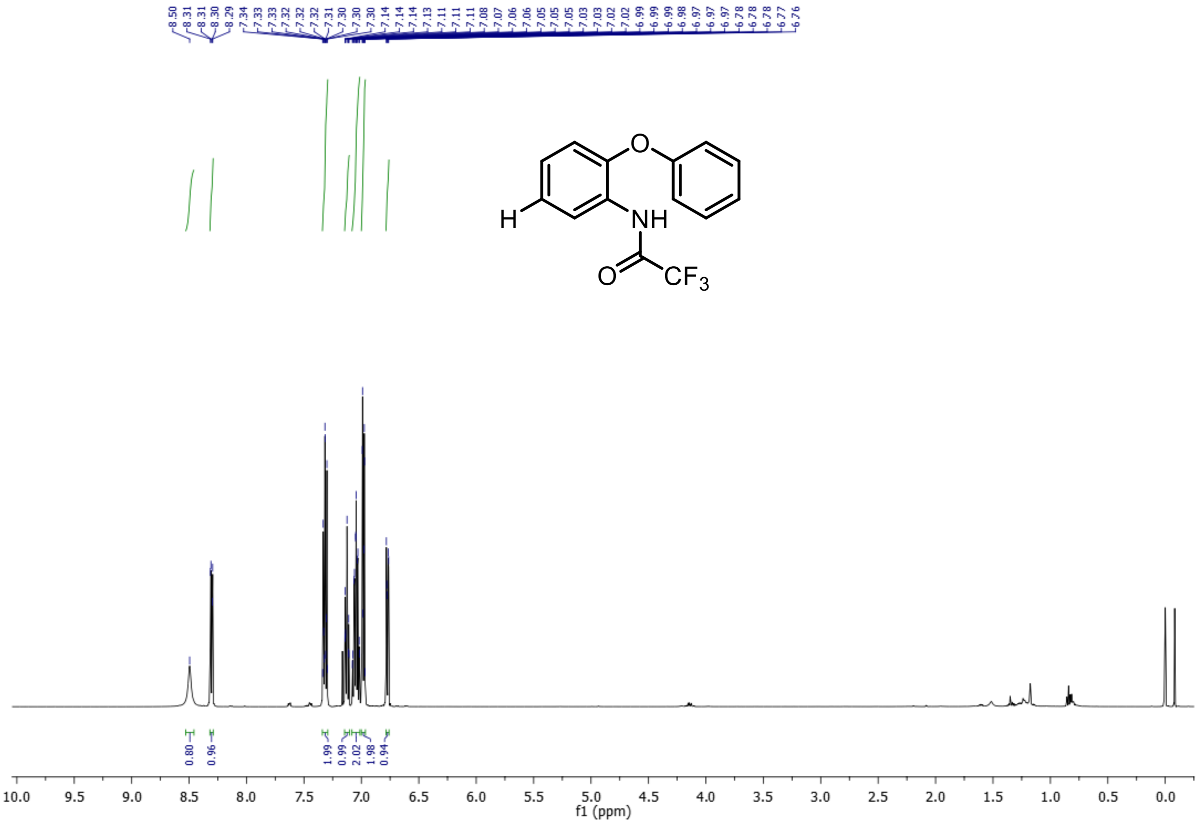

^{13}C NMR (126 MHz, $CDCl_3$) : **5aa**

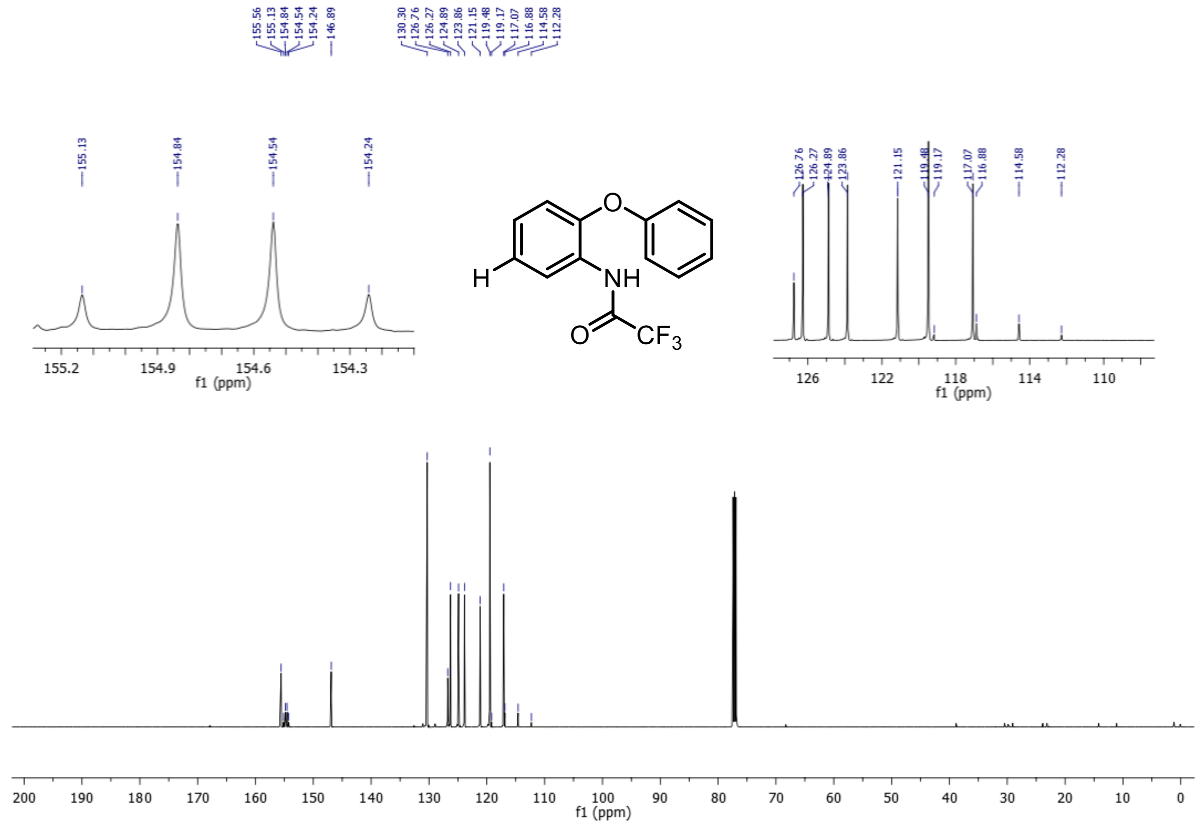

^{19}**F NMR** (376 MHz, $CDCl_3$) : **5aa**

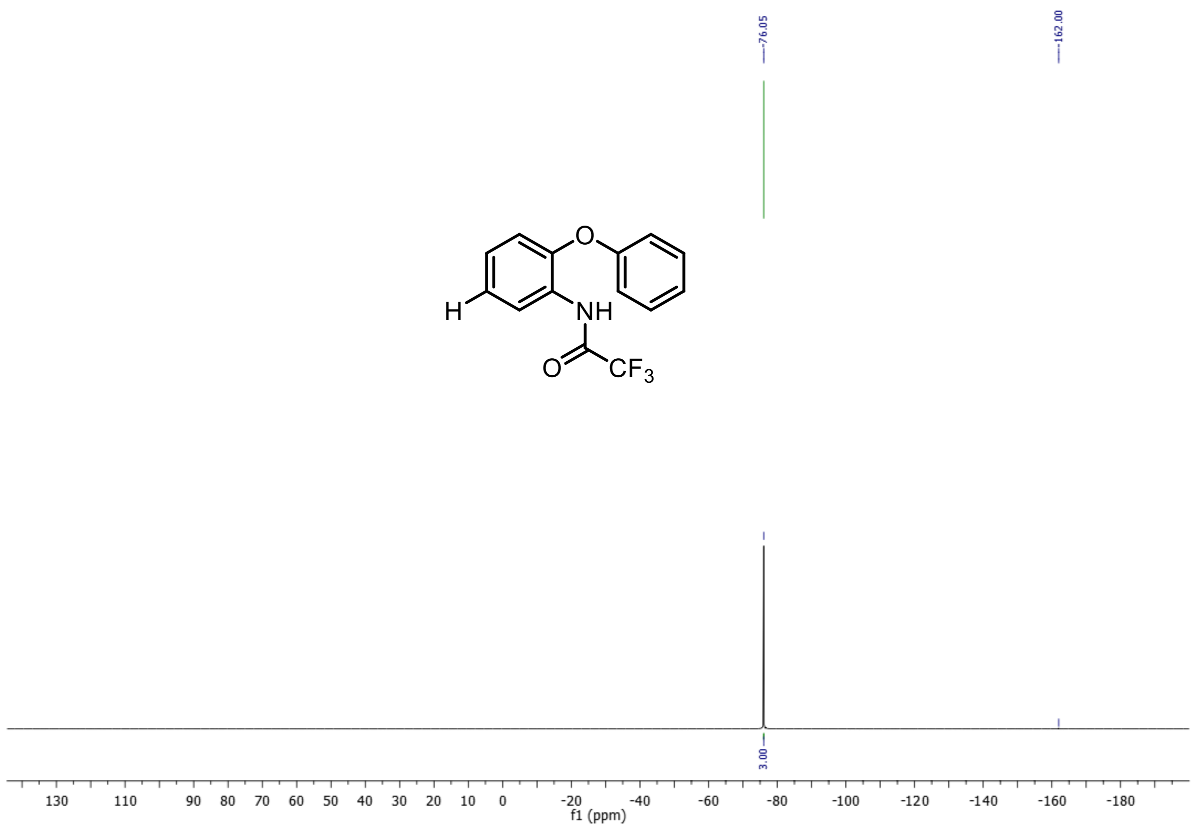

^{1}**H NMR** (500 MHz, $CDCl_3$) : **5ba**

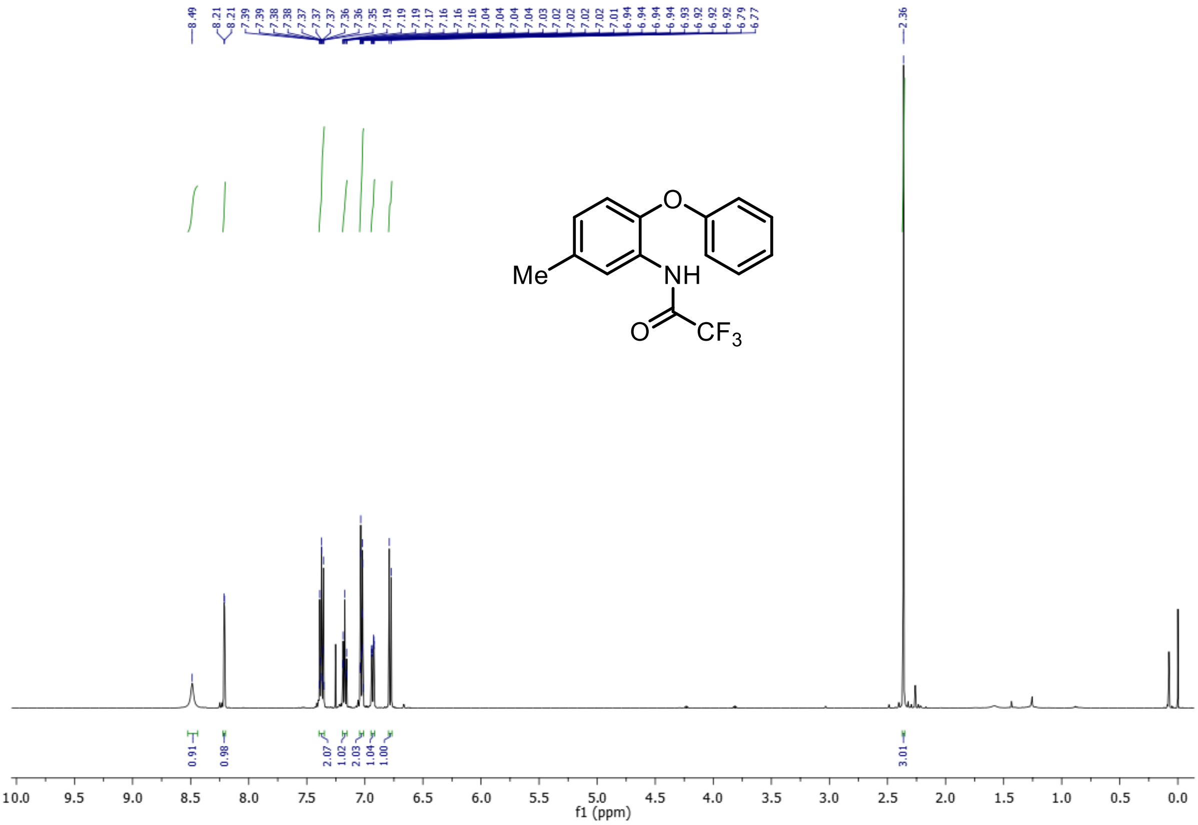

^{13}C NMR (126 MHz, $CDCl_3$) : **5ba**

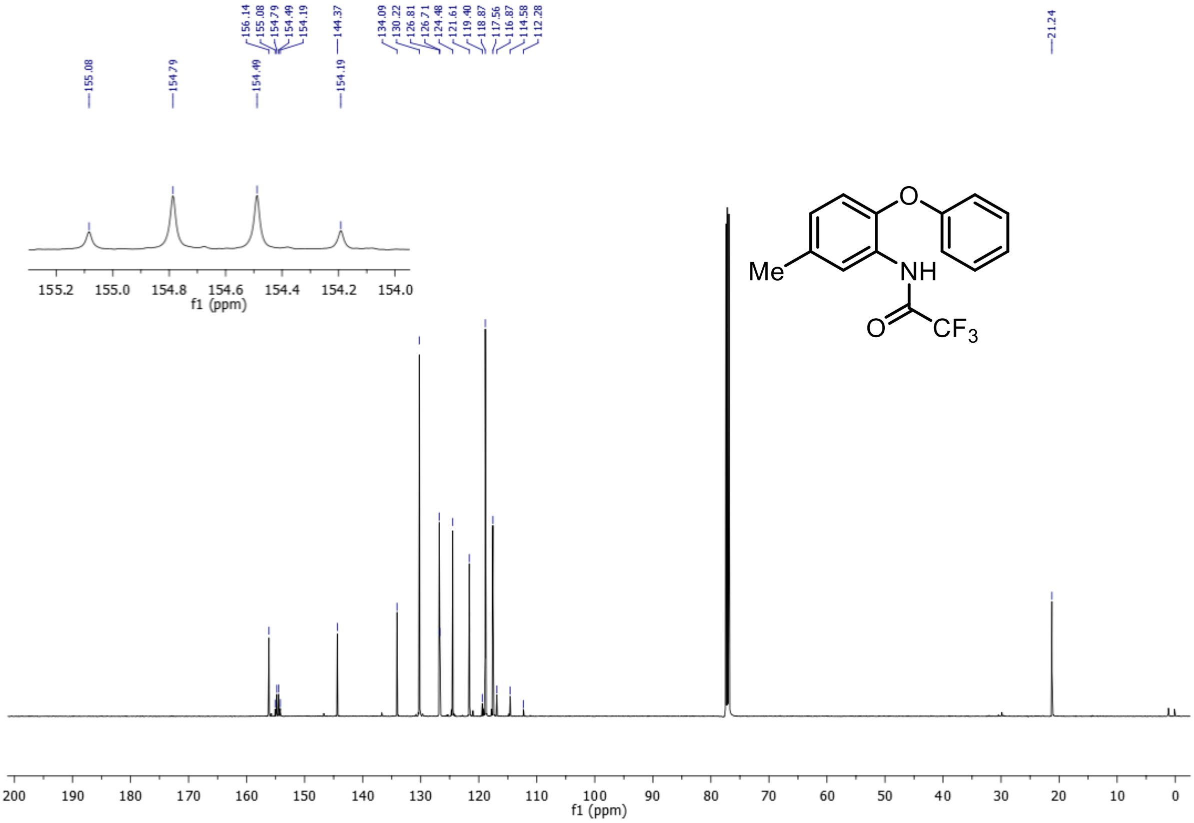

^{19}F NMR (376 MHz, $CDCl_3$) : **5ba**

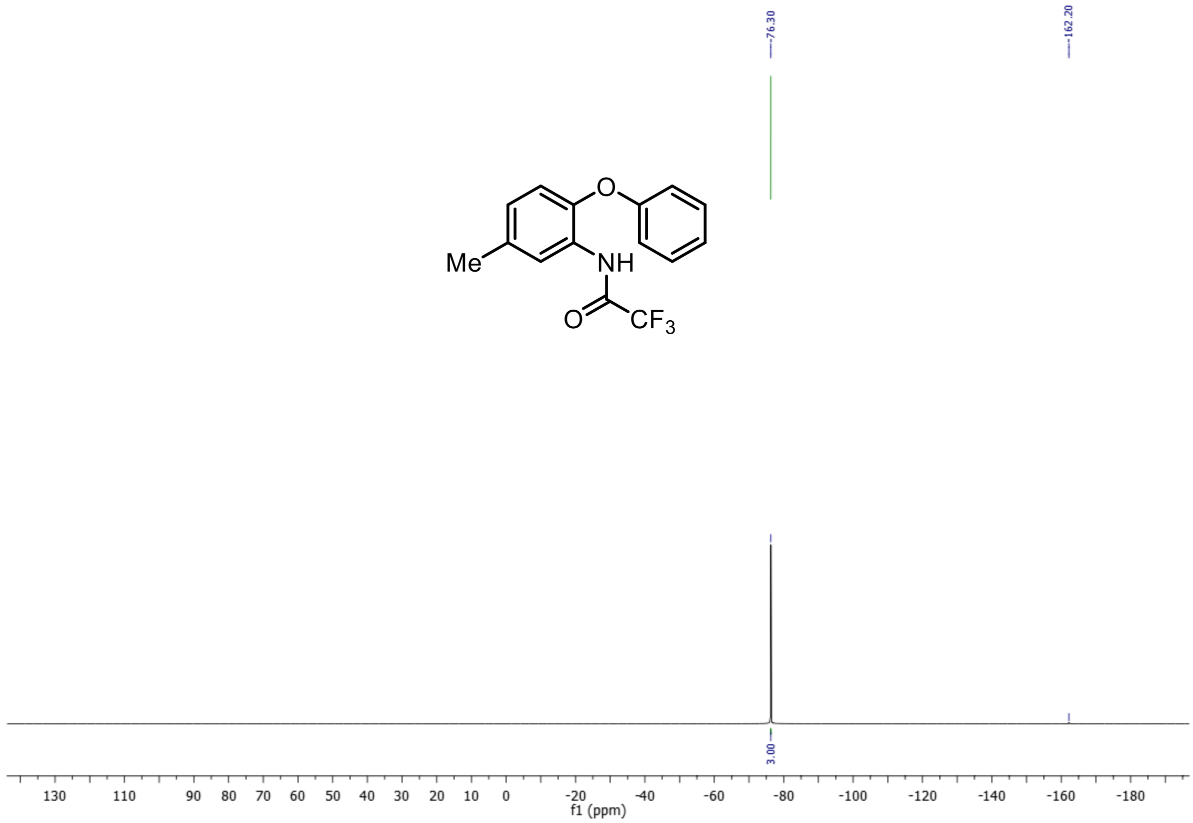

^{1}H NMR (500 MHz, $CDCl_3$) : **5ca**

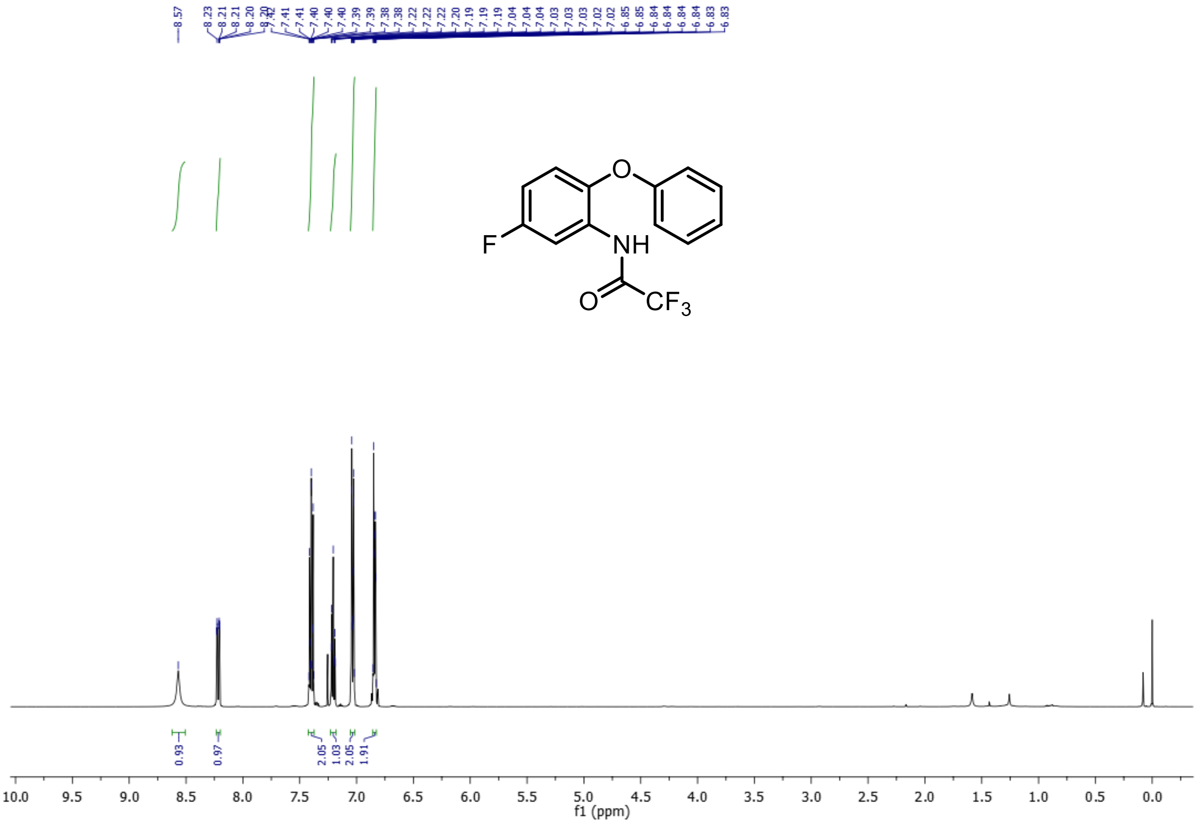

^{13}C NMR (126 MHz, $CDCl_3$) : **5ca**

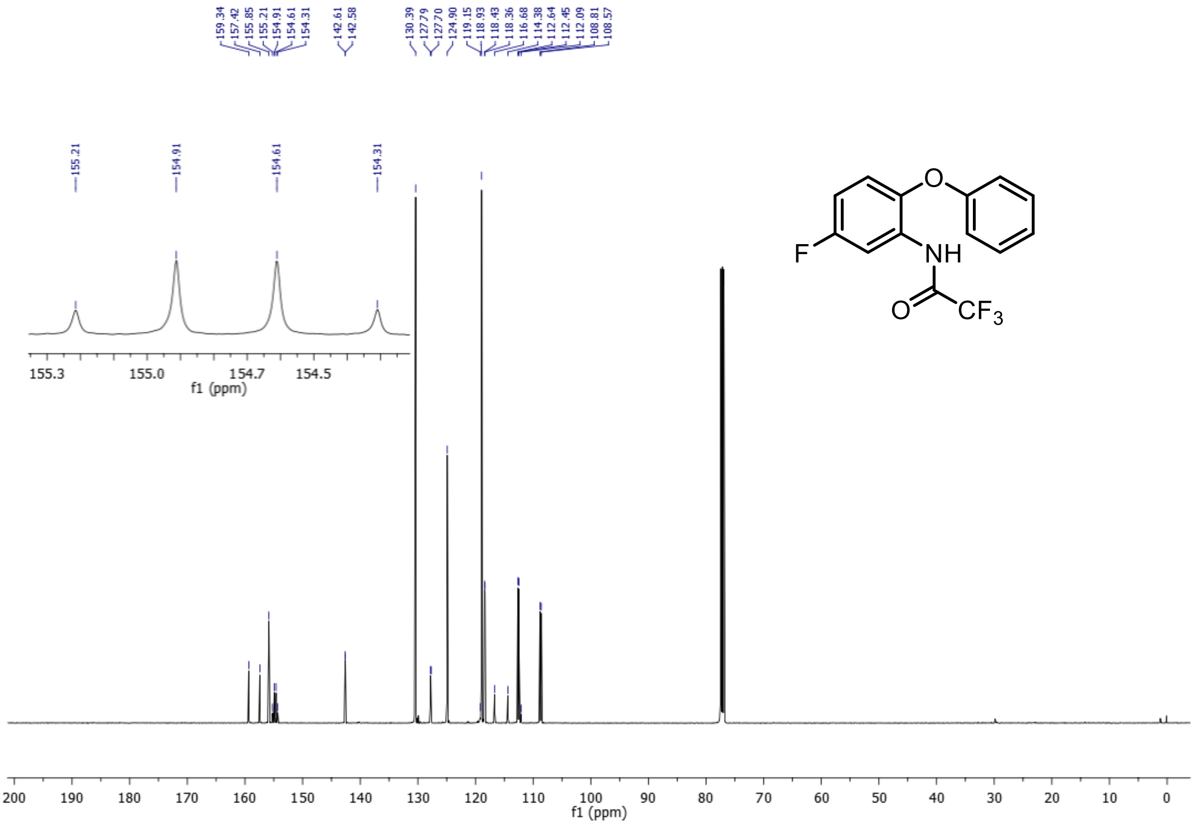

^{19}F NMR (376 MHz, $CDCl_3$) : **5ca**

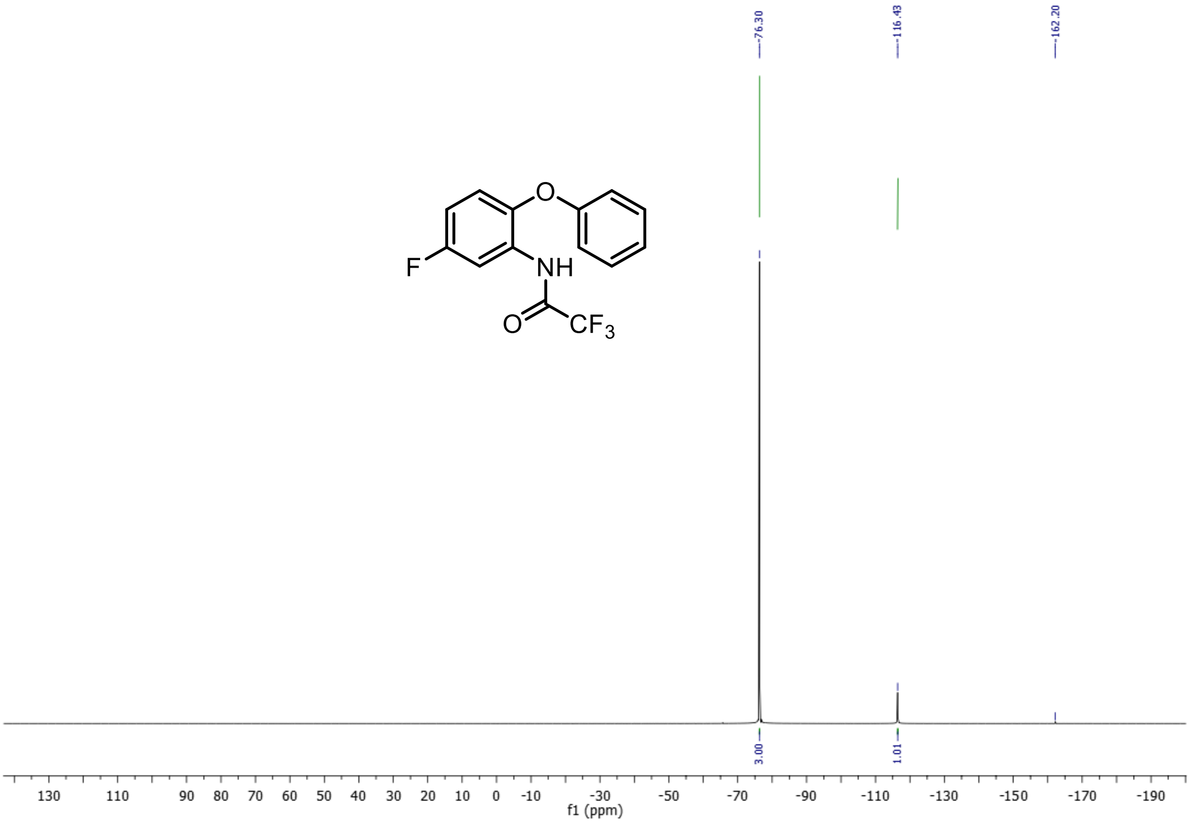

^{1}H NMR (500 MHz, $CDCl_3$) : **5da**

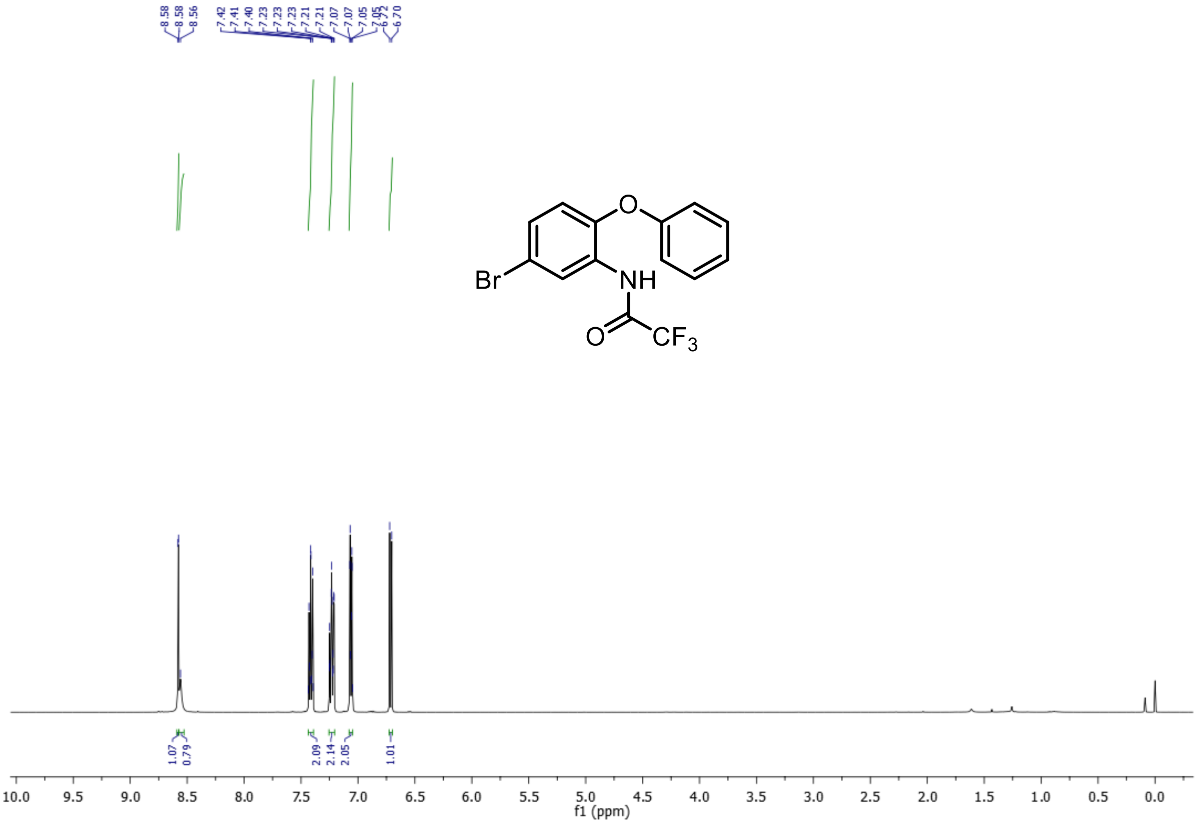

^{13}C NMR (126 MHz, $CDCl_3$) : **5da**

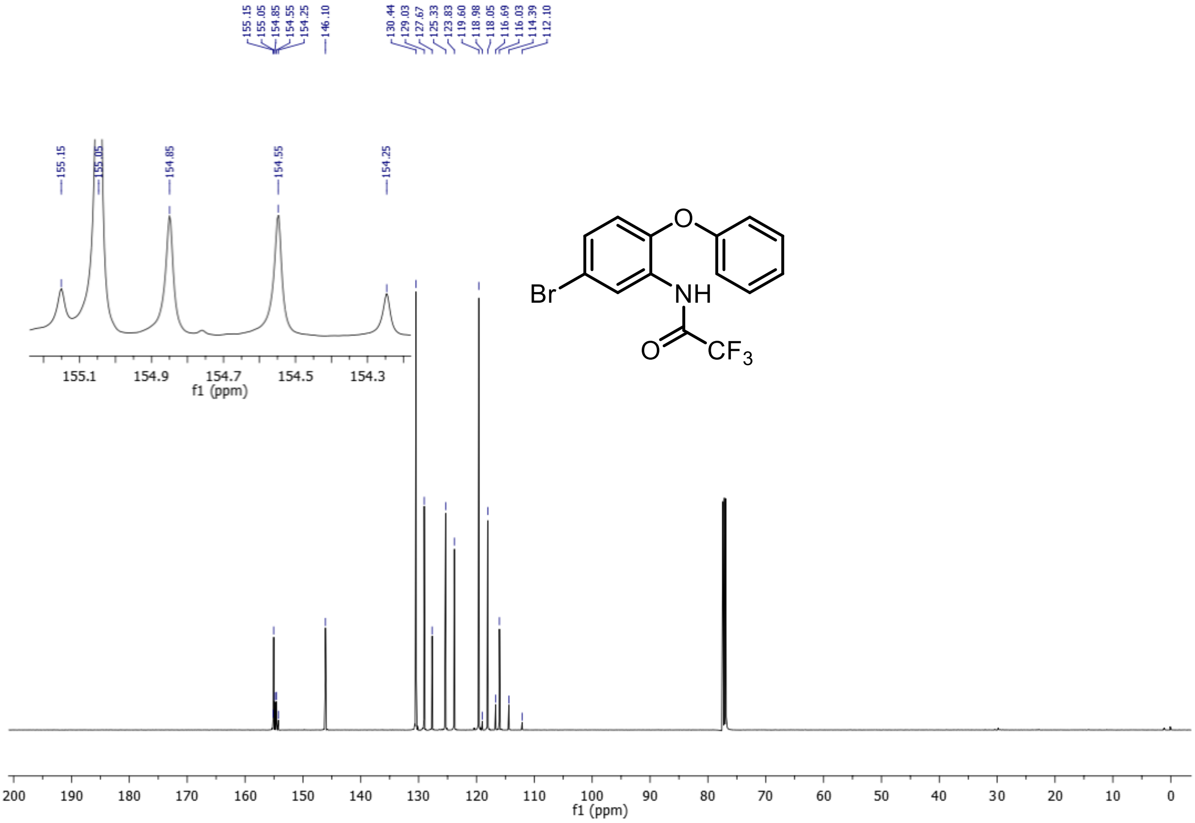

^{19}F NMR (376 MHz, $CDCl_3$) : **5da**

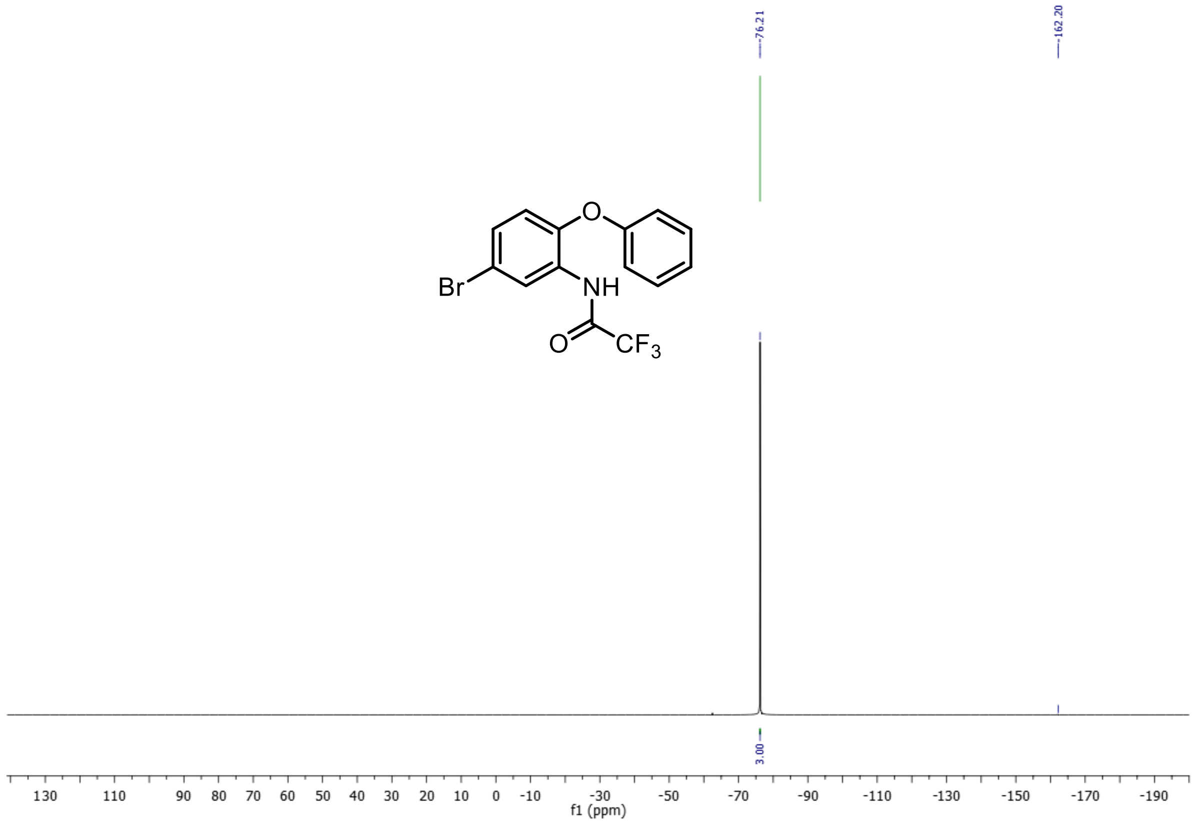

^{1}**H NMR** (500 MHz, $CDCl_3$) : **5ea**

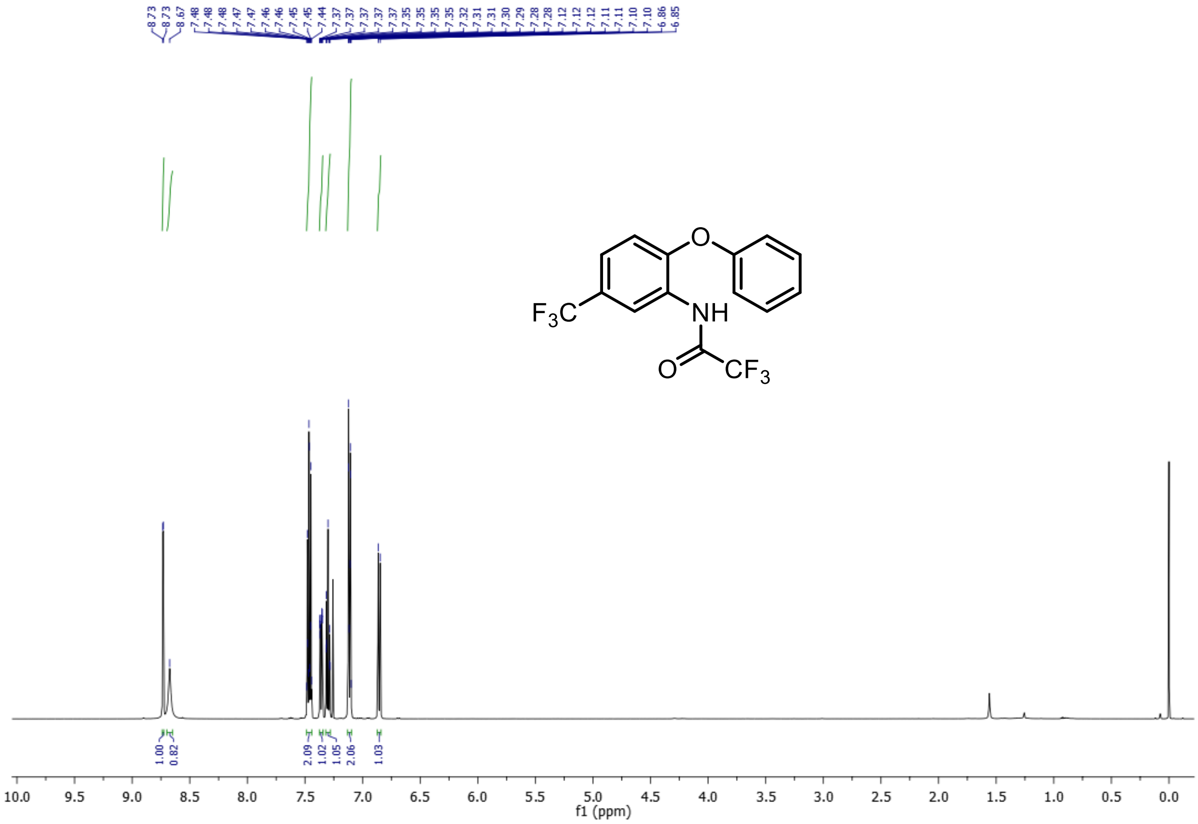

^{13}**C NMR** (126 MHz, $CDCl_3$) : **5ea**

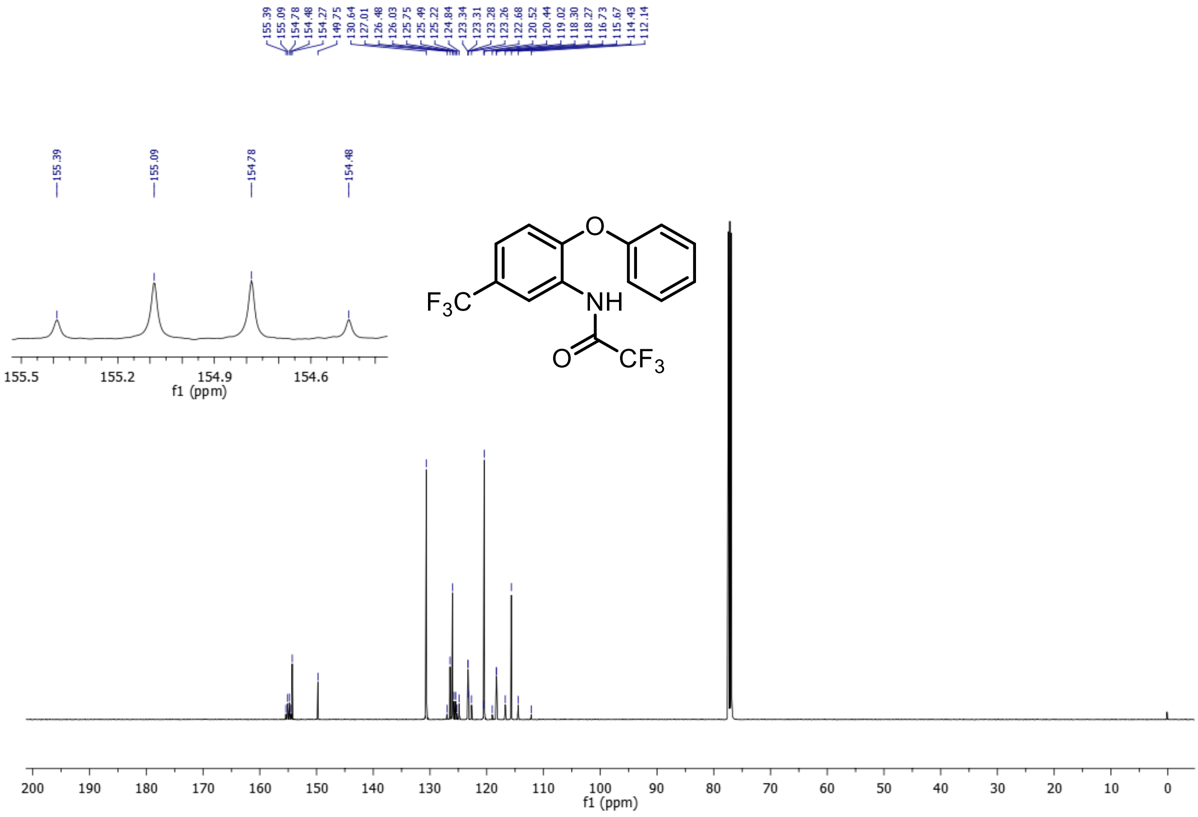

^{19}F NMR (376 MHz, $CDCl_3$) : **5ea**

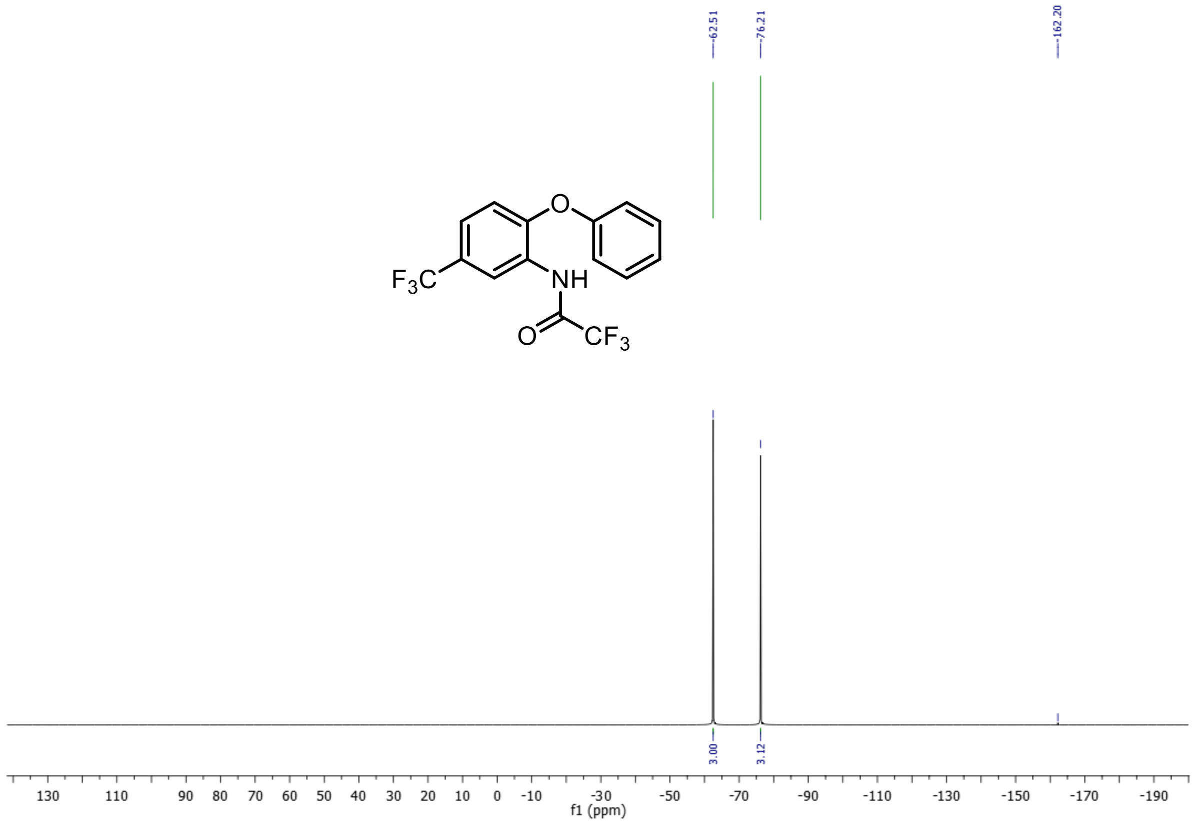

^{1}H NMR (500 MHz, $CDCl_3$) : **5fa**

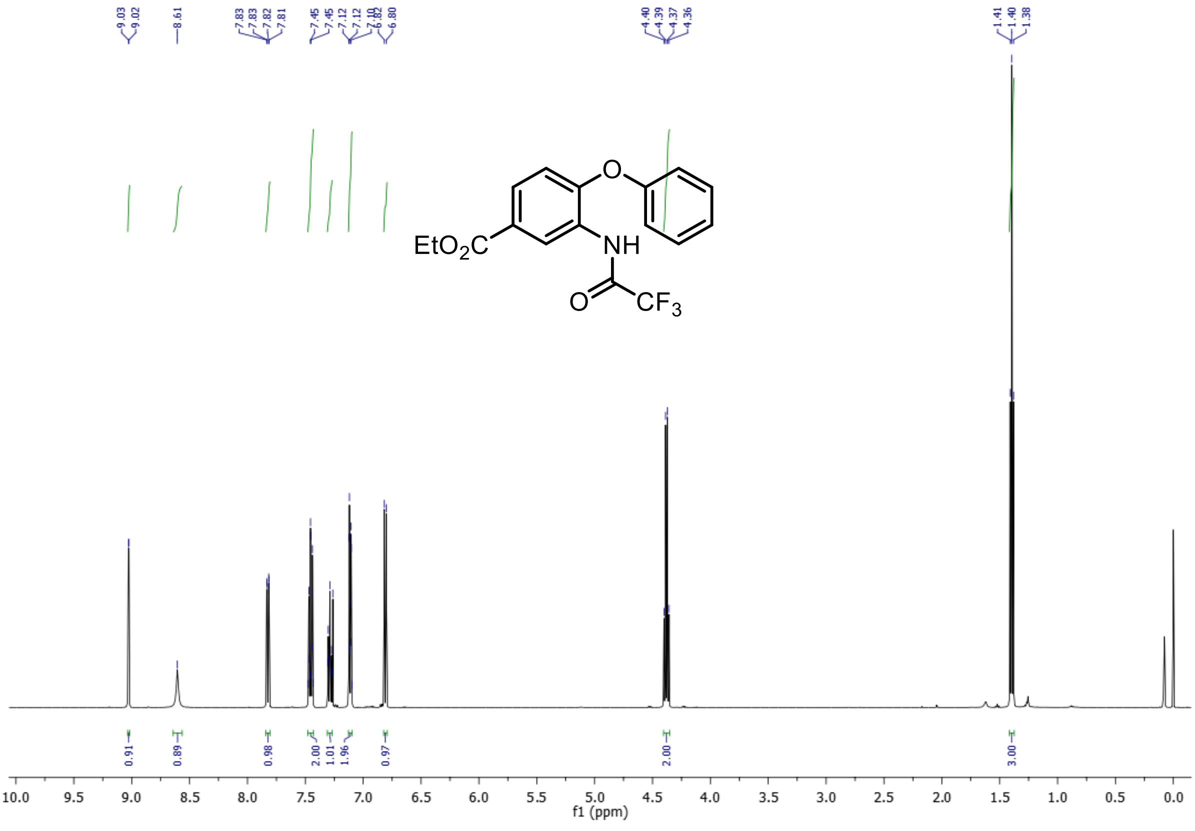

^{13}C NMR (126 MHz, $CDCl_3$) : **5fa**

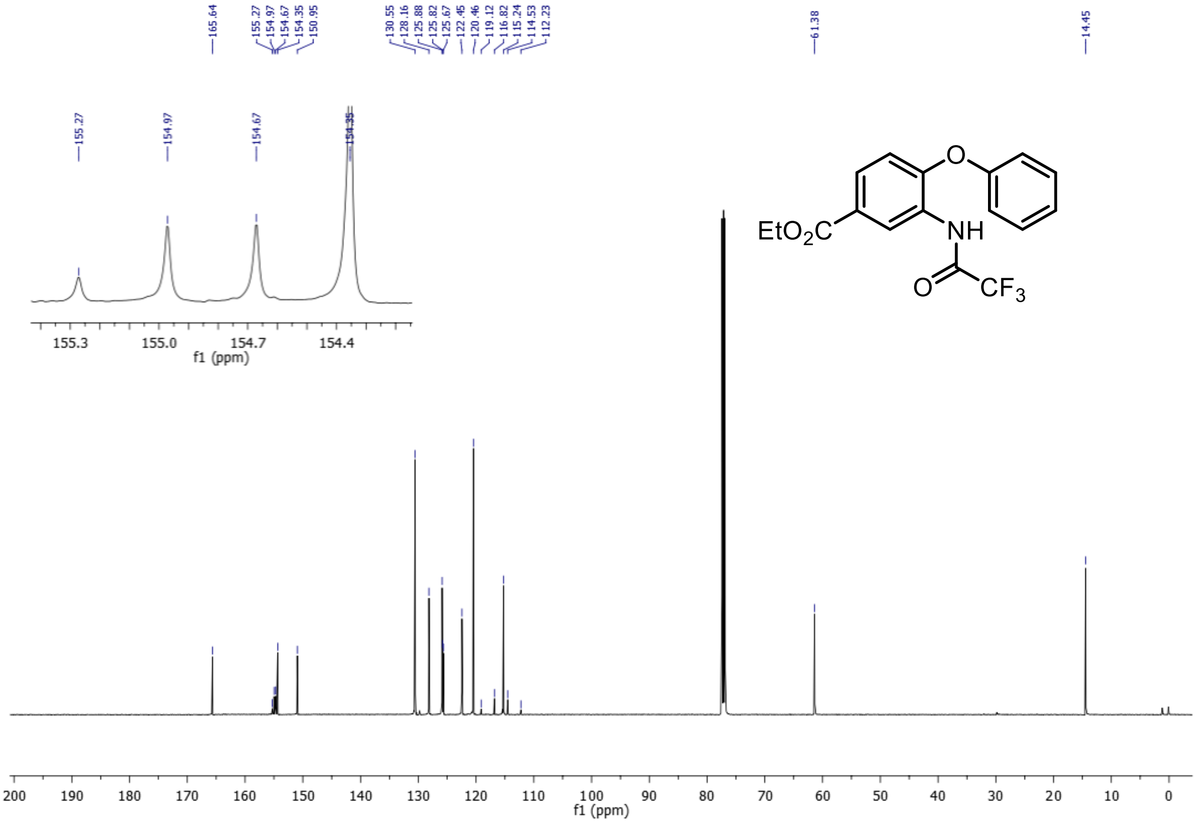

^{19}F NMR (376 MHz, $CDCl_3$) : **5fa**

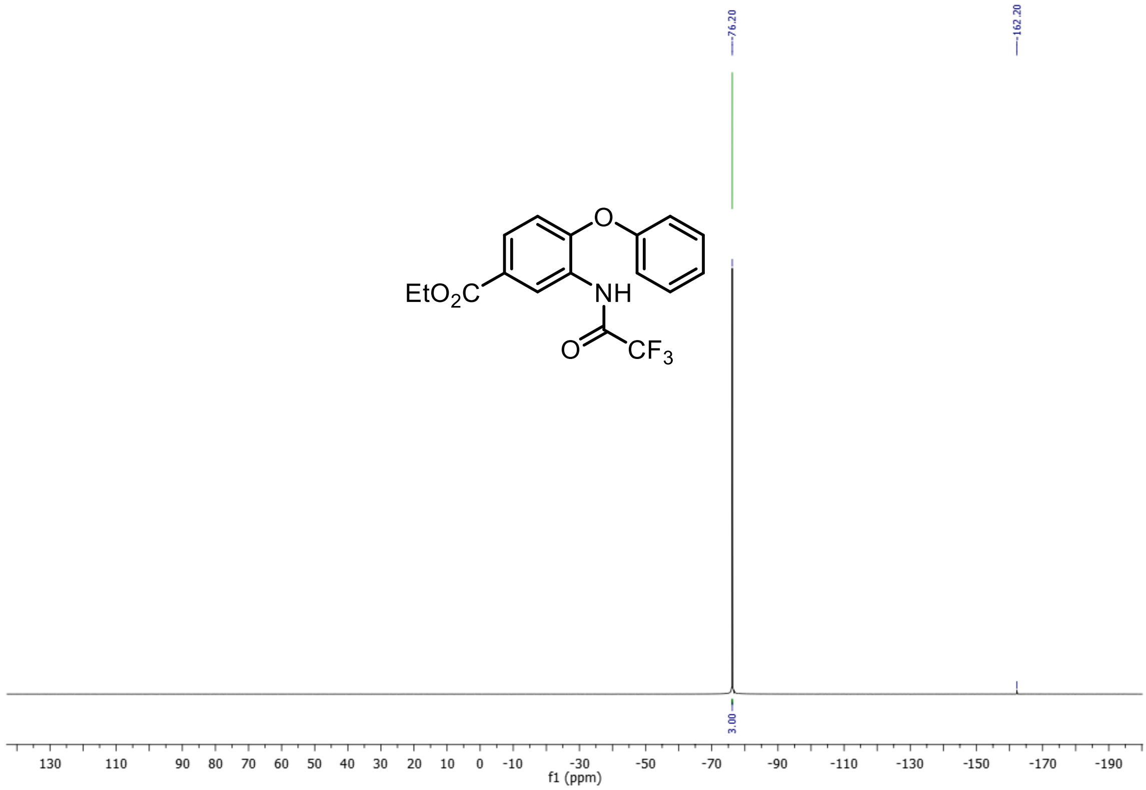

^{1}H NMR (500 MHz, $CDCl_3$) : **5haa**

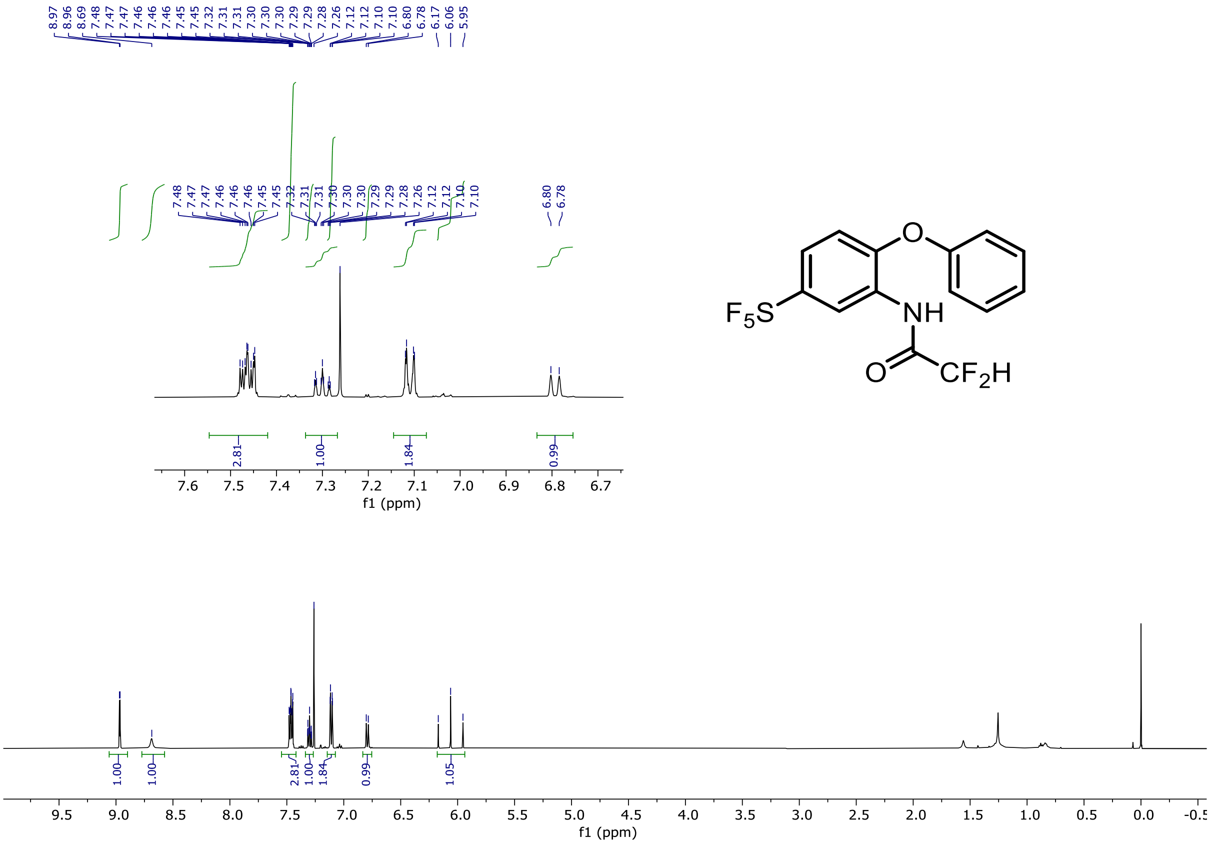

^{13}C NMR (126 MHz, $CDCl_3$) : **5haa**

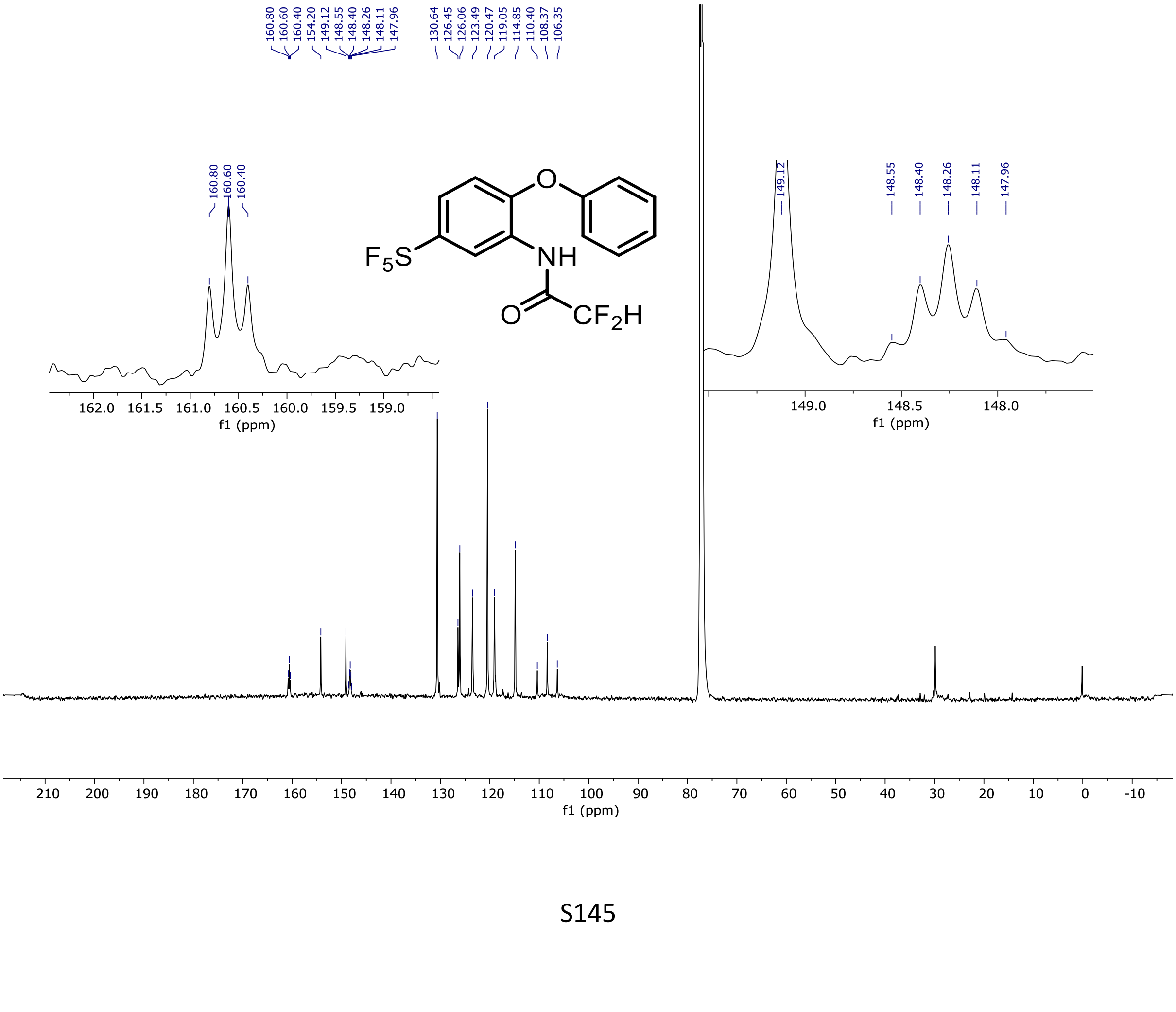

^{19}F NMR (658 MHz, $CDCl_3$) : **5haa**

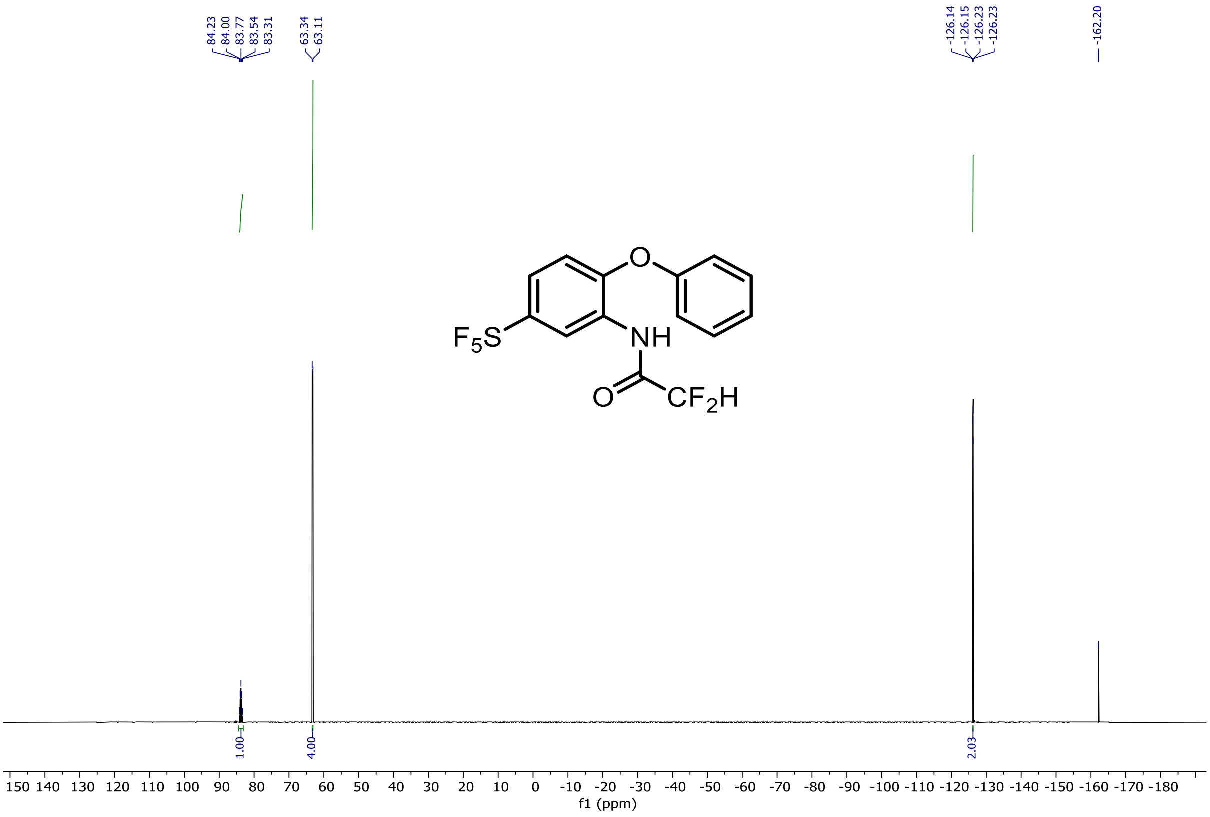

^{1}H NMR (500 MHz, $CDCl_3$) : **5hab**

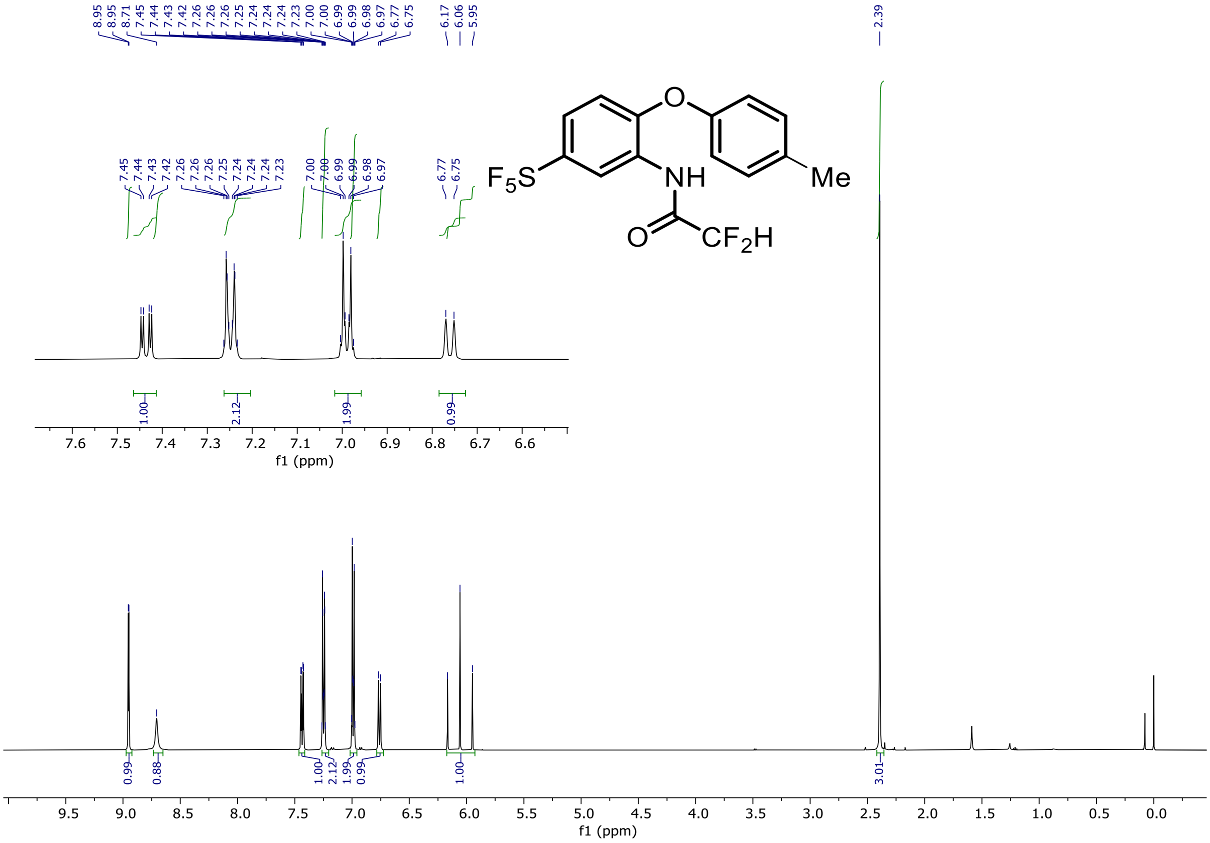

^{13}C NMR (126 MHz, $CDCl_3$) : **5hab**

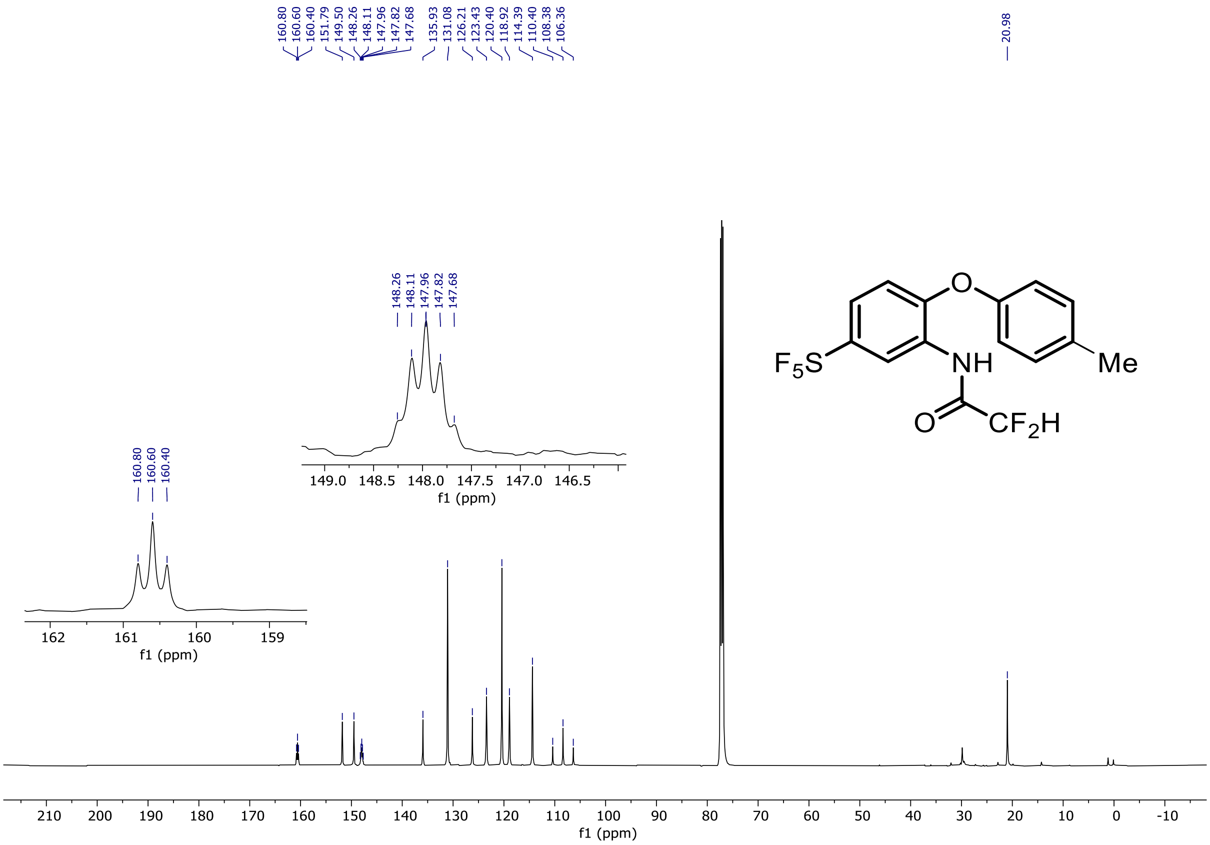

^{19}F NMR (658 MHz, $CDCl_3$) : **5hab**

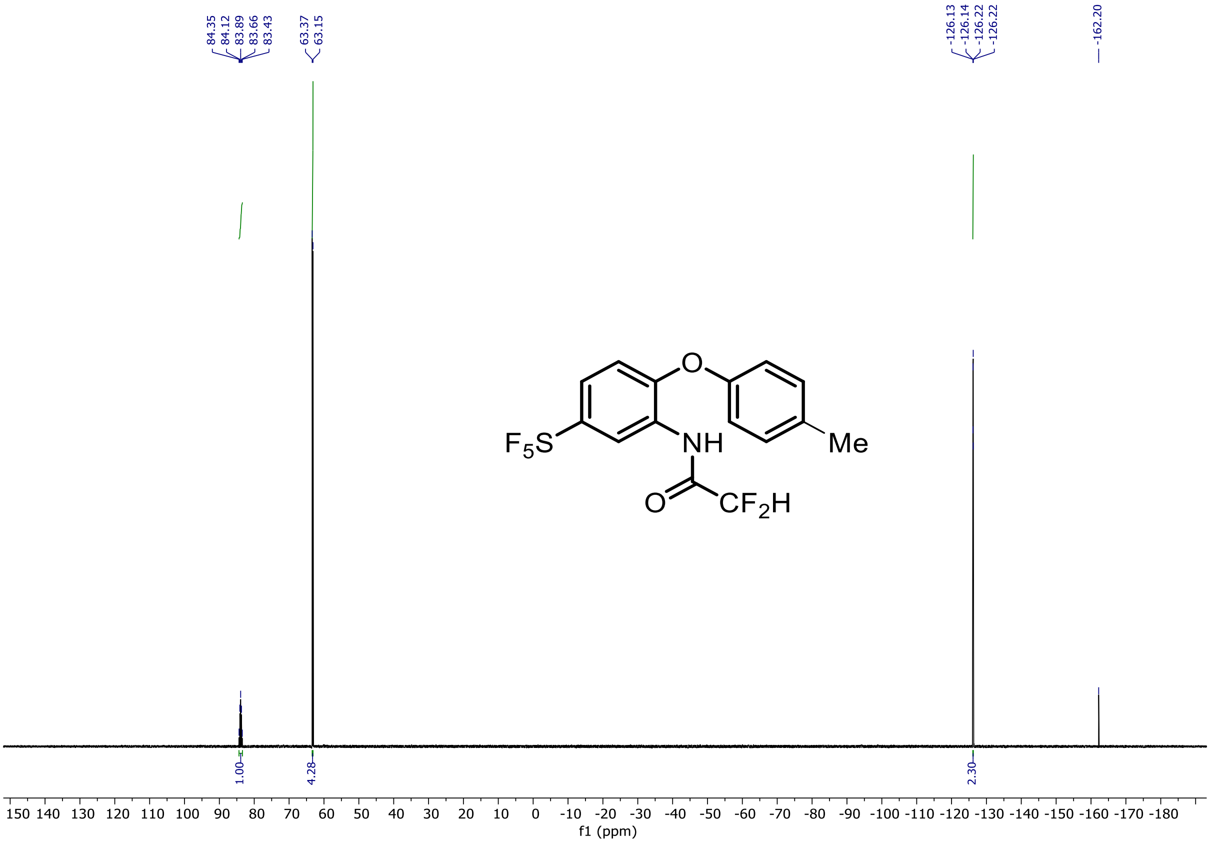

^{1}H NMR (500 MHz, $CDCl_3$) : **5hac**

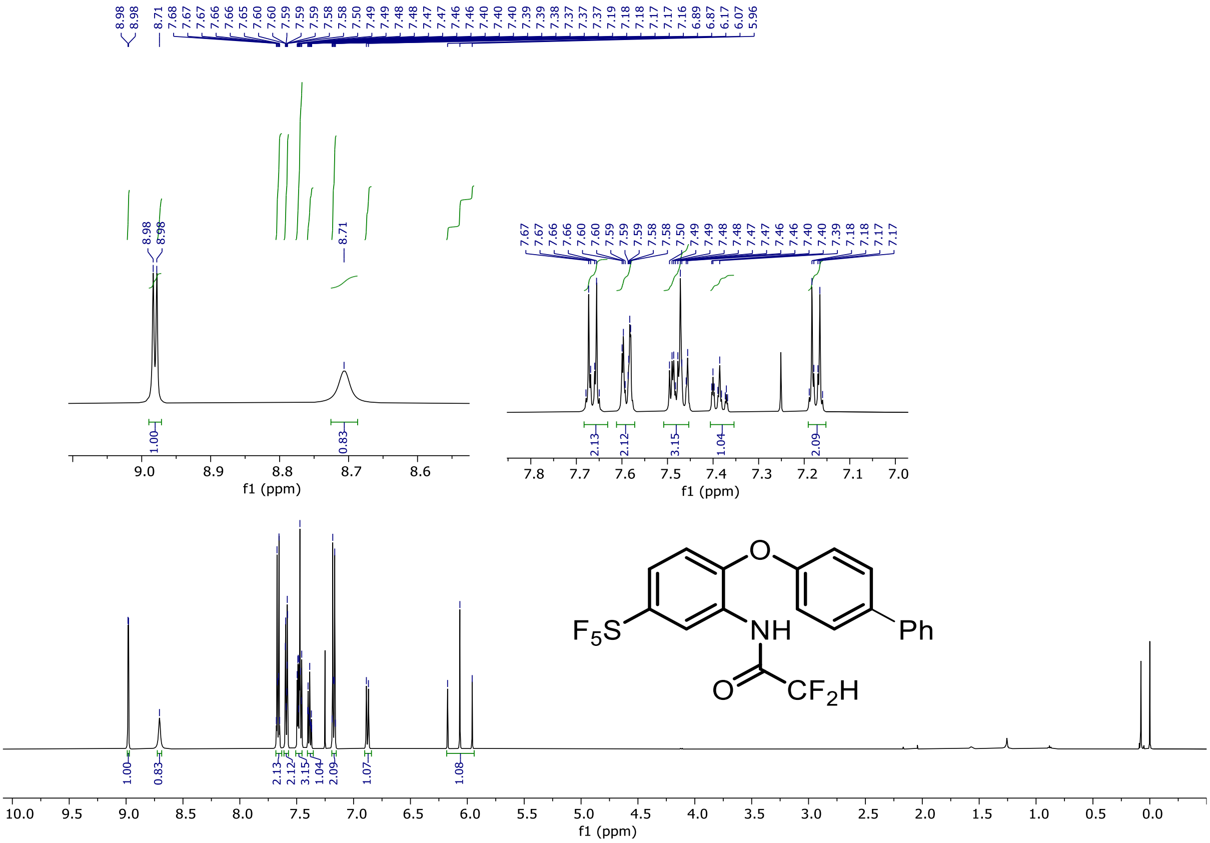

^{13}C NMR (126 MHz, $CDCl_3$) : **5hac**

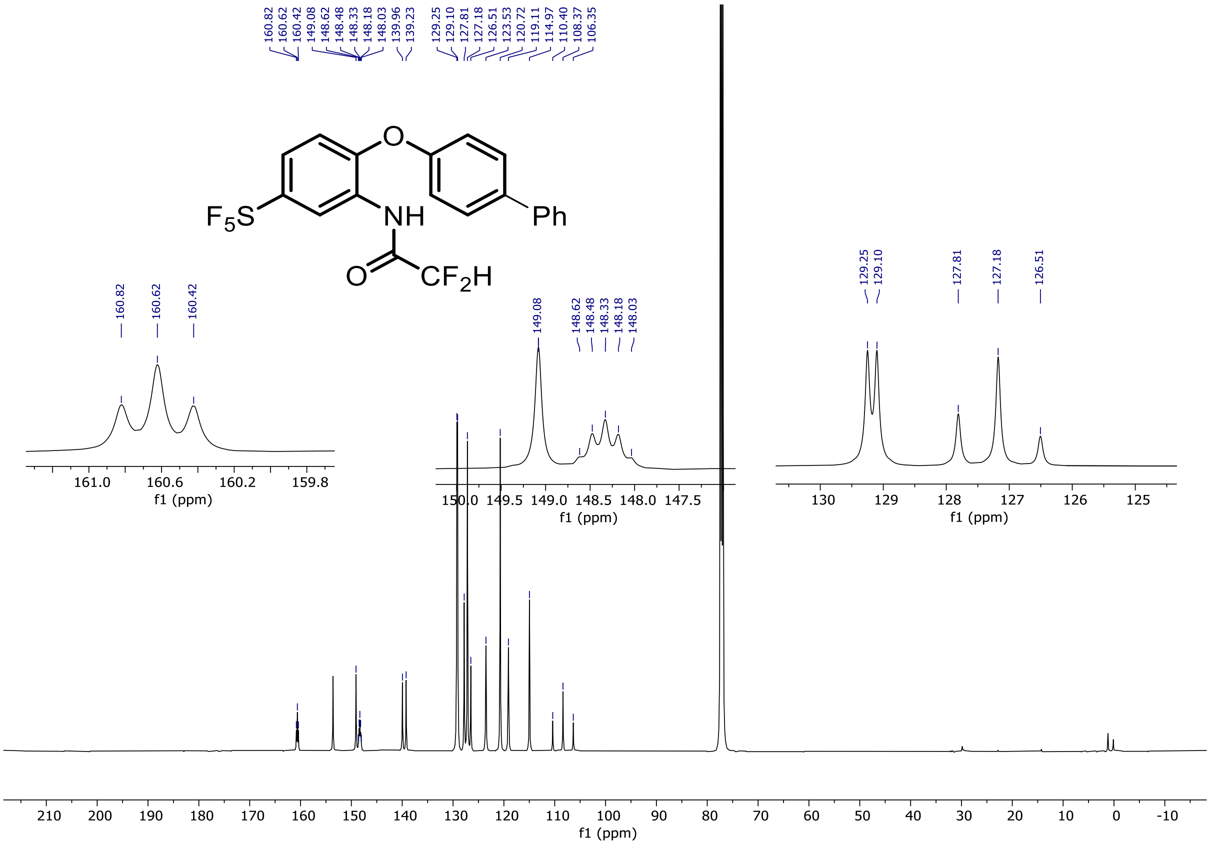

^{19}**F NMR** (658 MHz, CDCl$_3$) : **5hac**

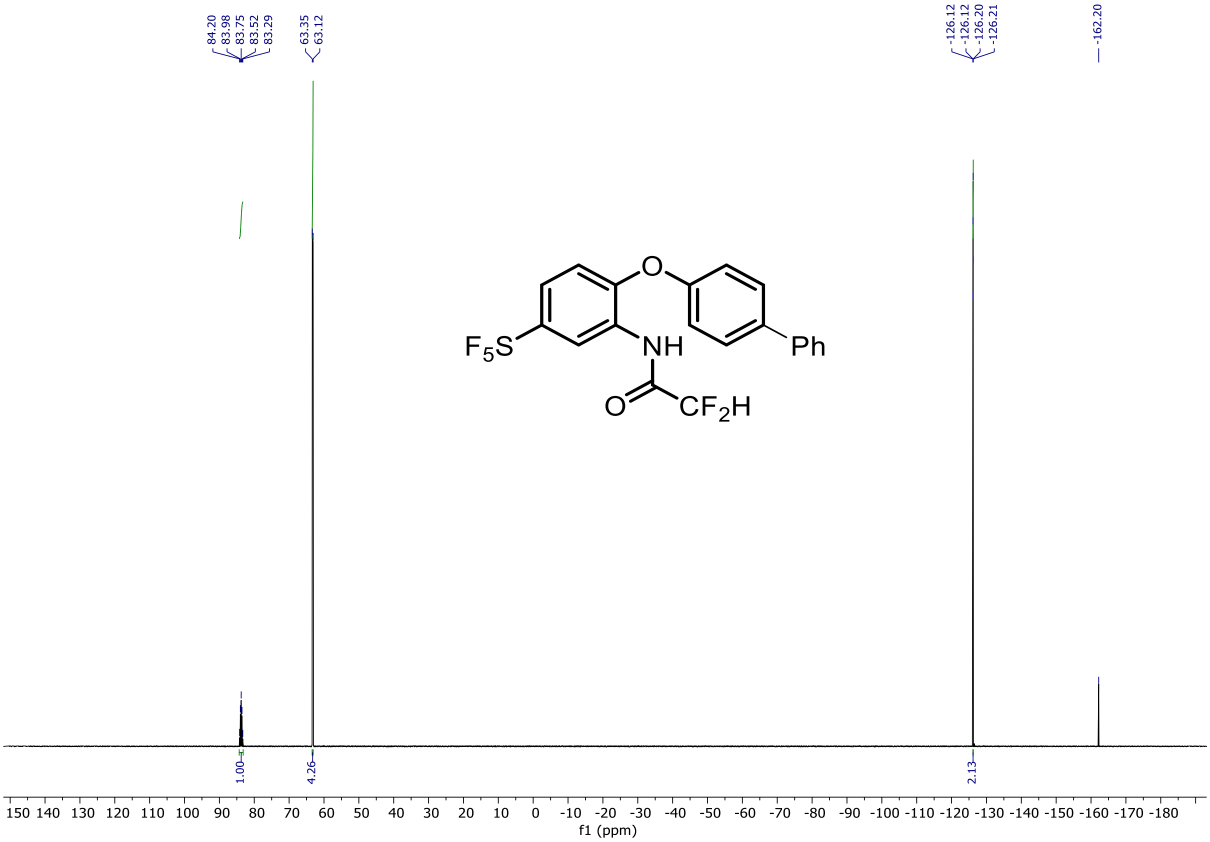

^{1}**H NMR** (500 MHz, CDCl$_3$) : **5had**

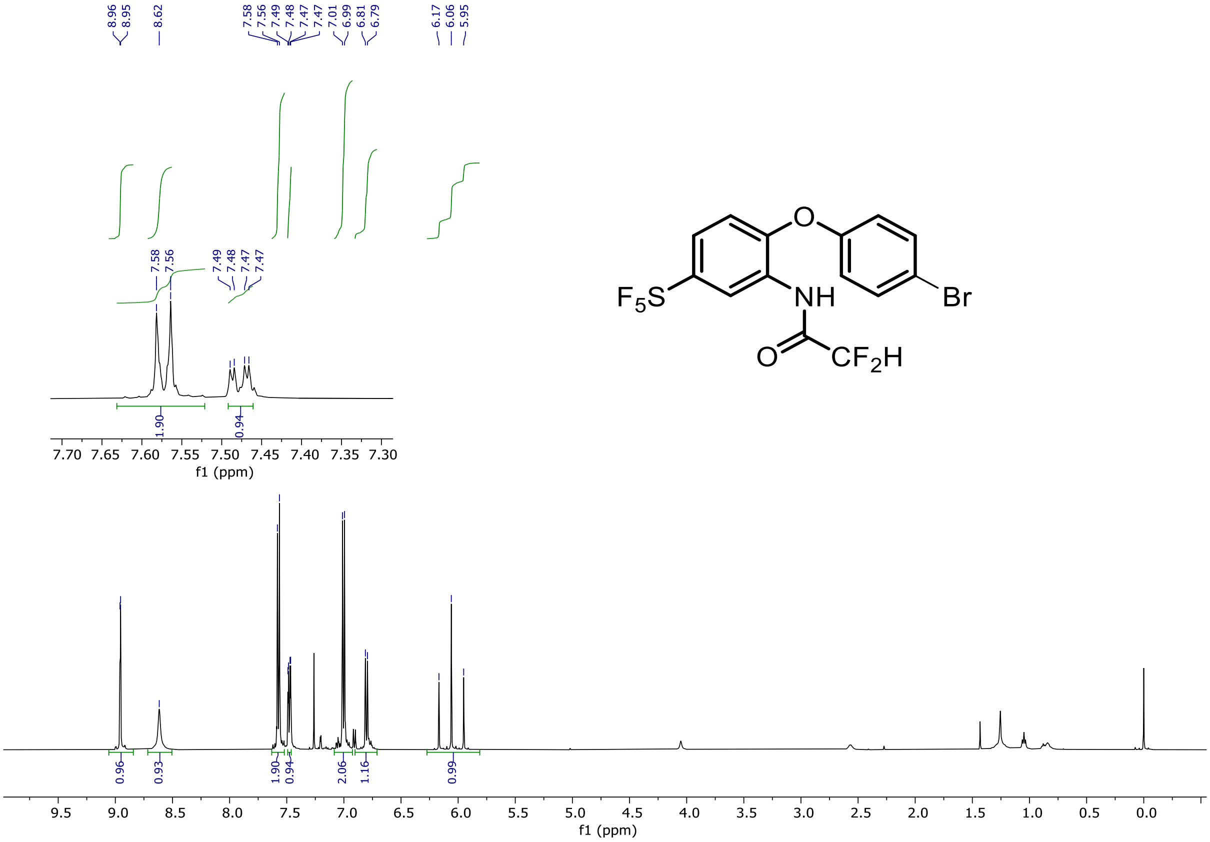

^{13}C NMR (126 MHz, $CDCl_3$) : **5had**

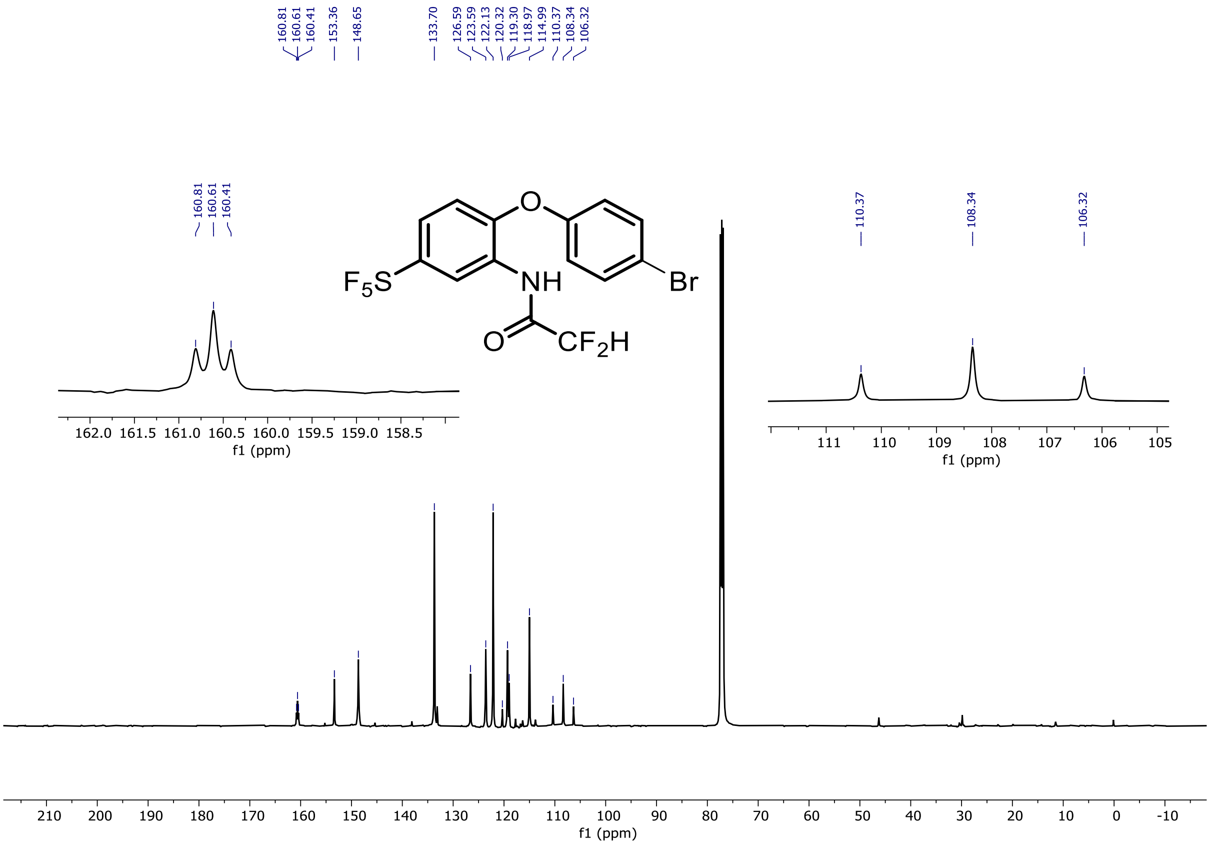

^{19}F NMR (658 MHz, $CDCl_3$) : **5had**

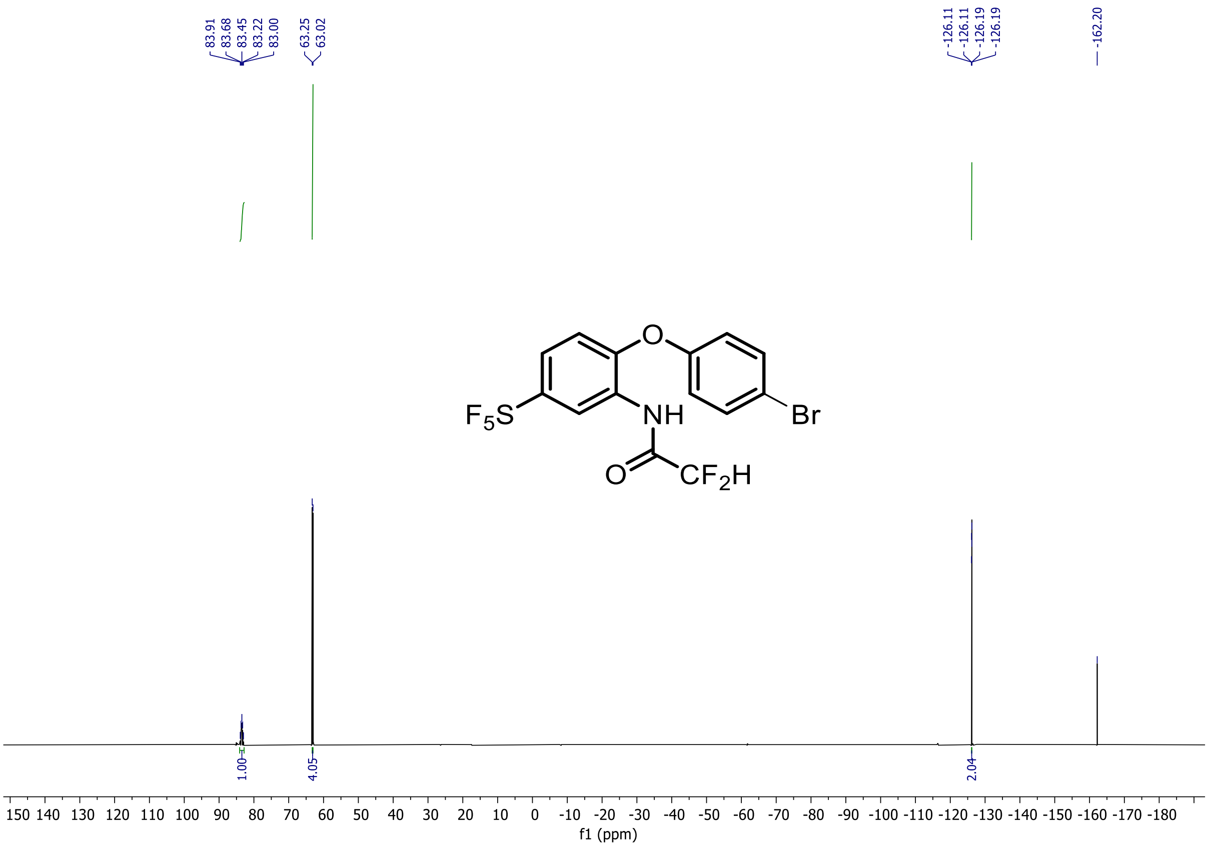

^{1}H NMR (500 MHz, $CDCl_3$) : **8a**

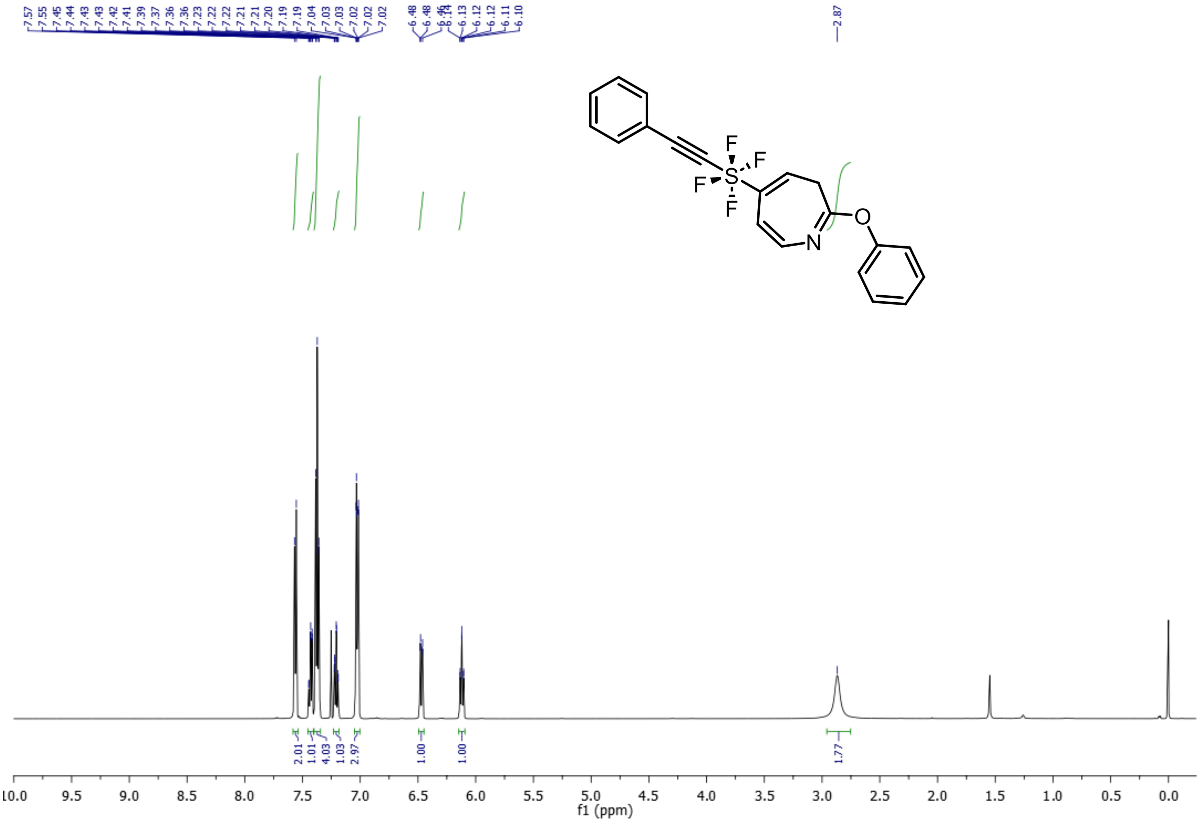

^{13}C NMR (126 MHz, $CDCl_3$) : **8a**

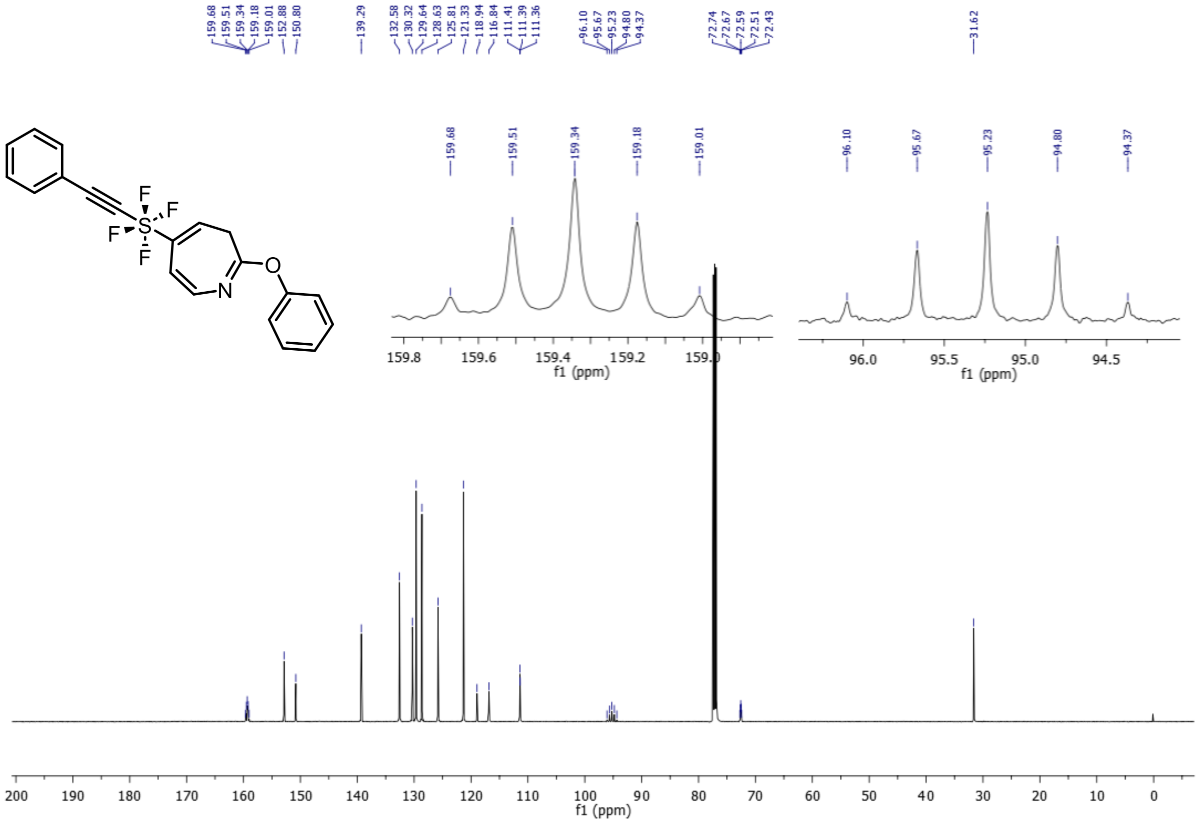

^{19}F NMR (282 MHz, $CDCl_3$) : **8a**

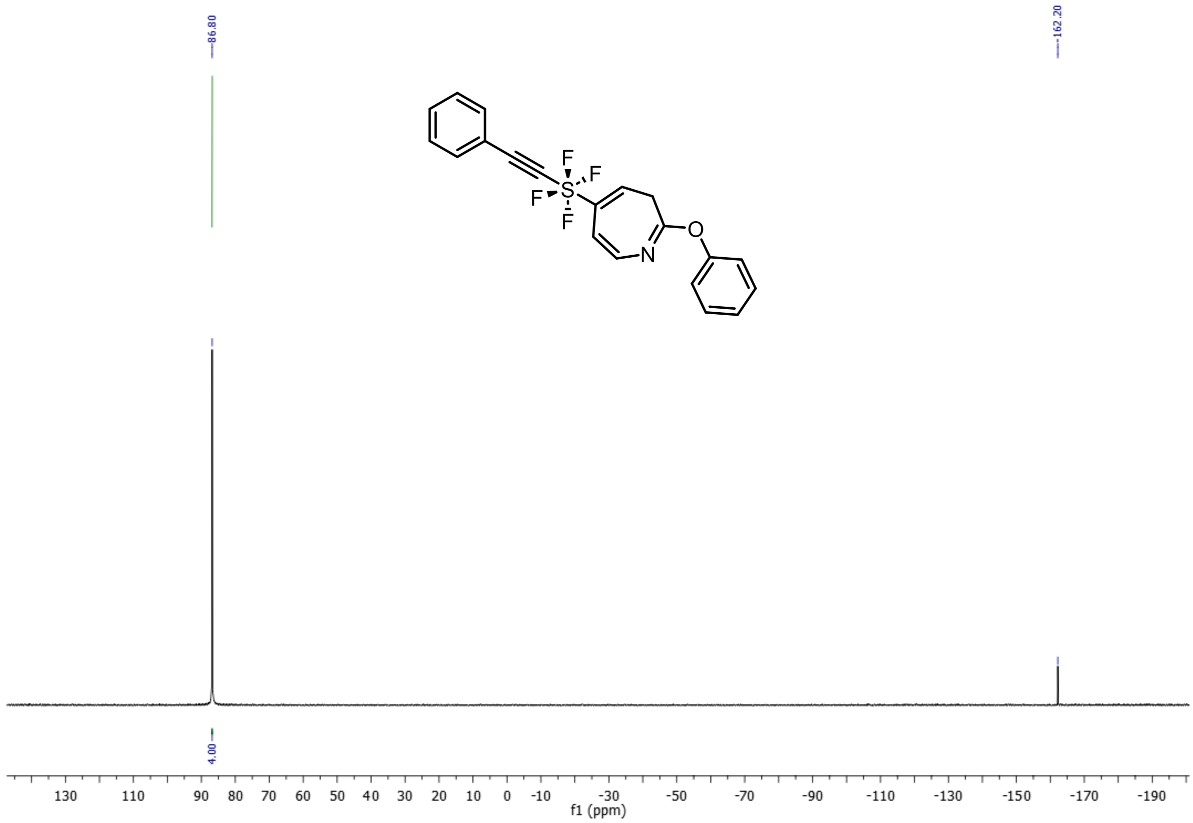

^{1}H NMR (500 MHz, $CDCl_3$) : **8b**

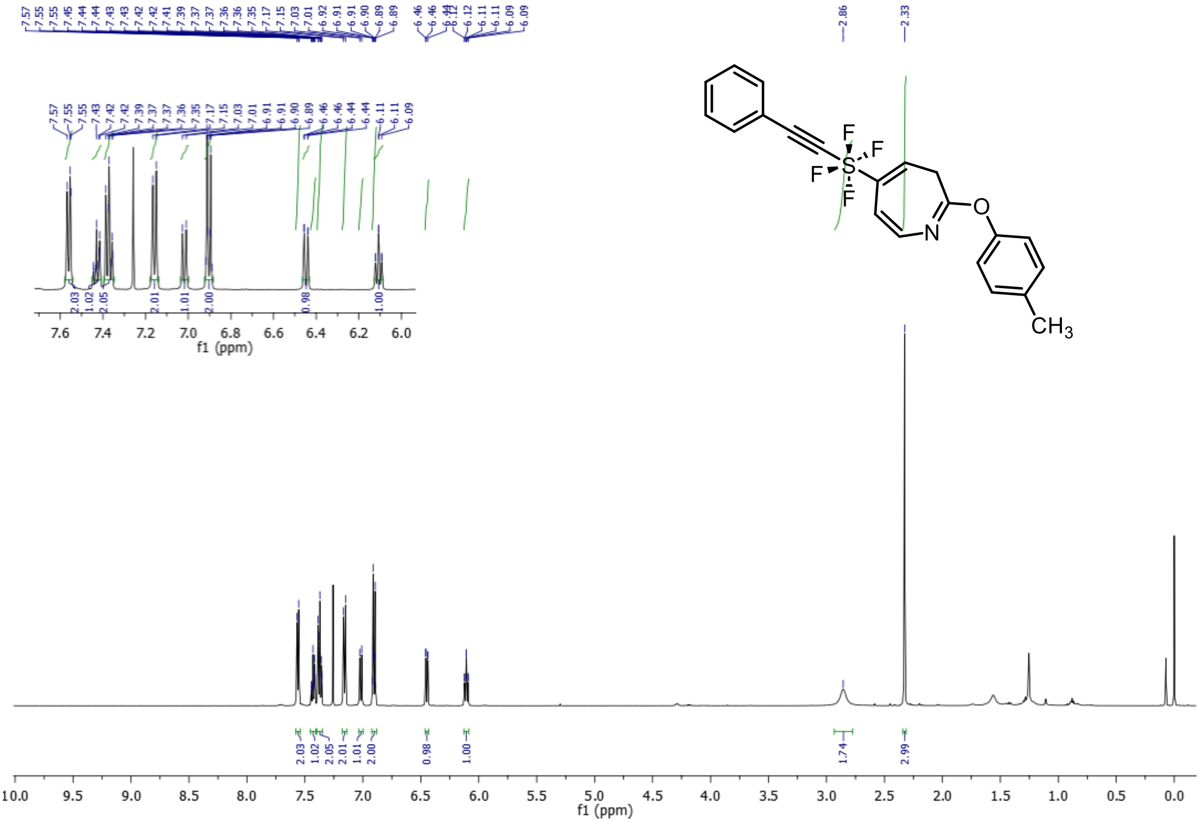

^{13}C NMR (126 MHz, $CDCl_3$) : **8b**

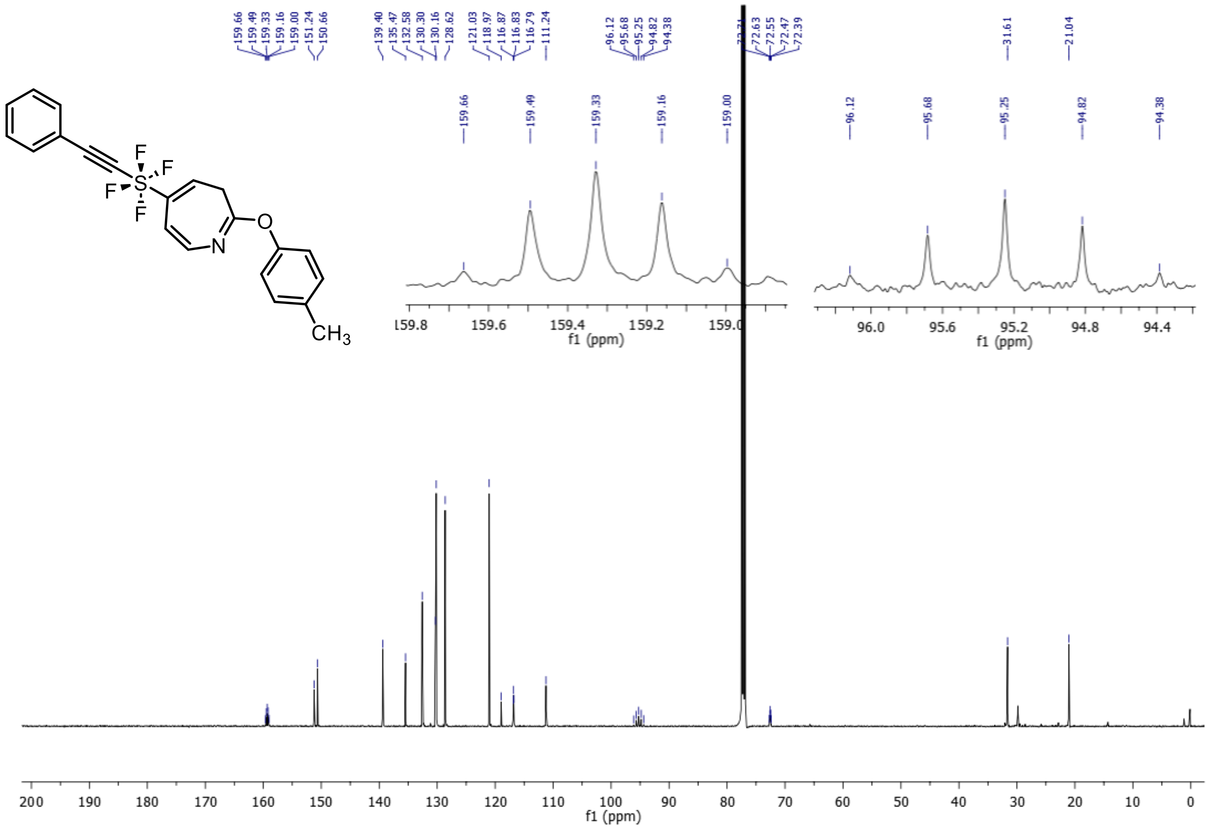

^{19}F NMR (658 MHz, $CDCl_3$) : **8b**

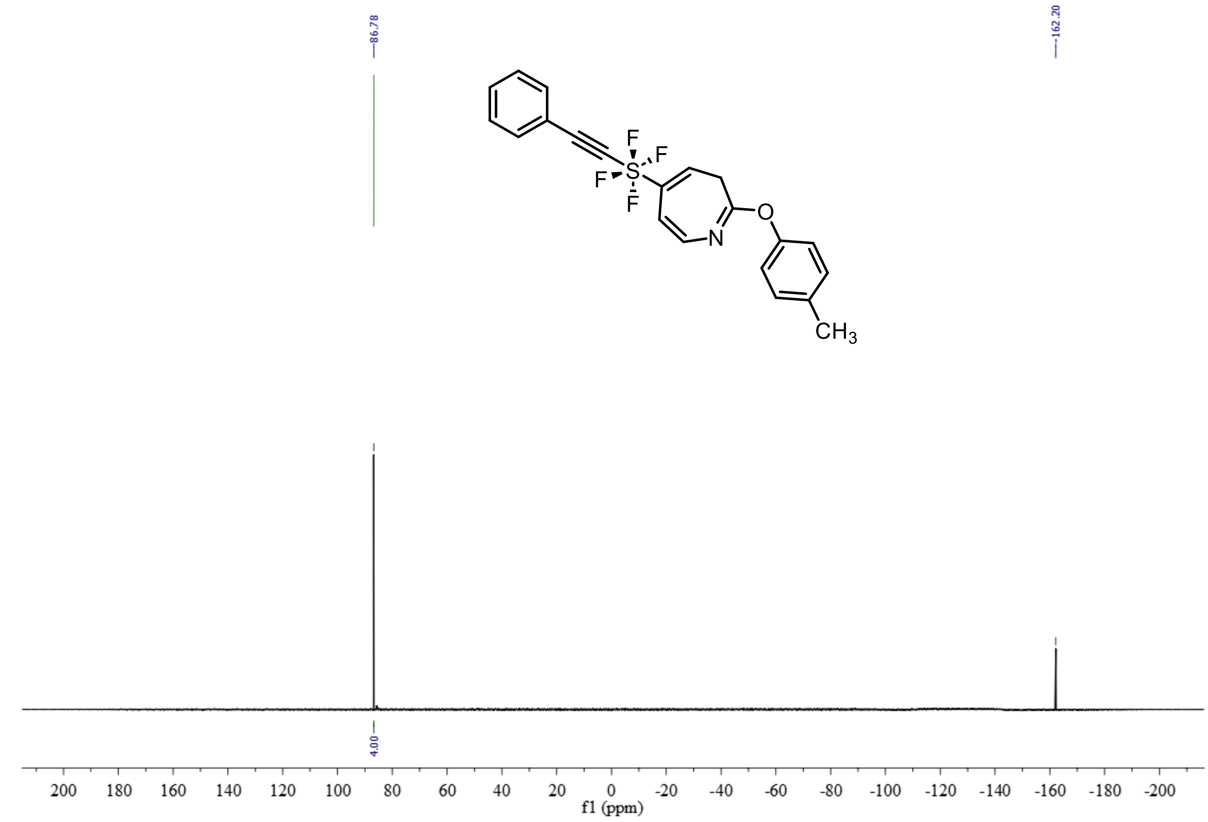

¹H NMR (500 MHz, $CDCl_3$) : **8c**

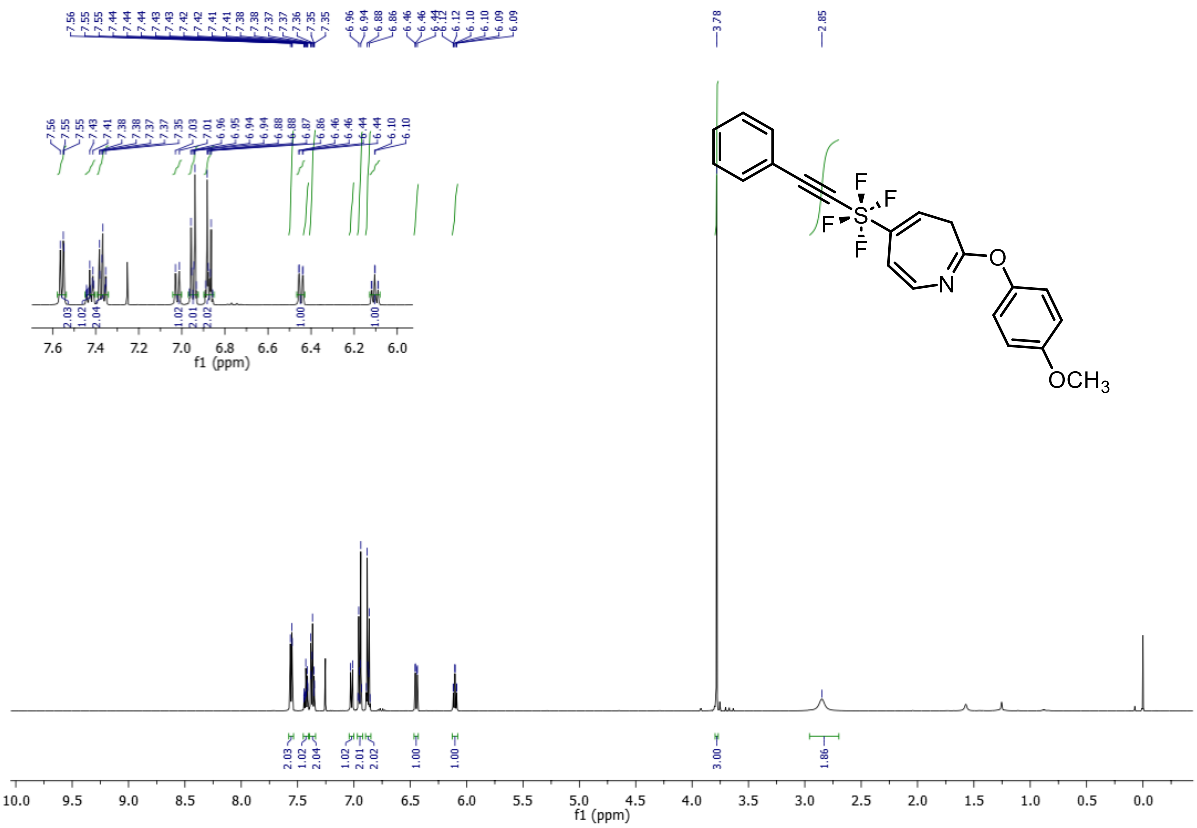

¹³C NMR (126 MHz, $CDCl_3$) : **8c**

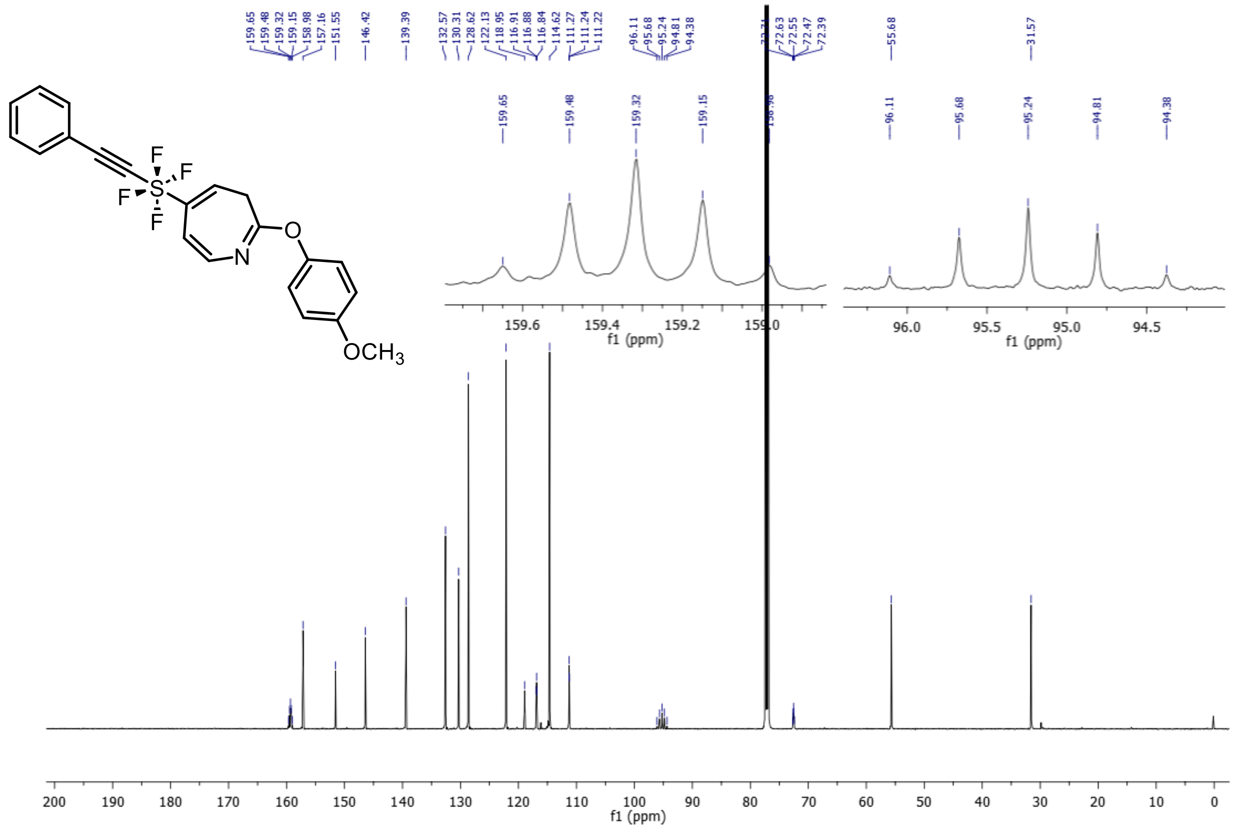

^{19}F NMR (282 MHz, $CDCl_3$) : **8c**

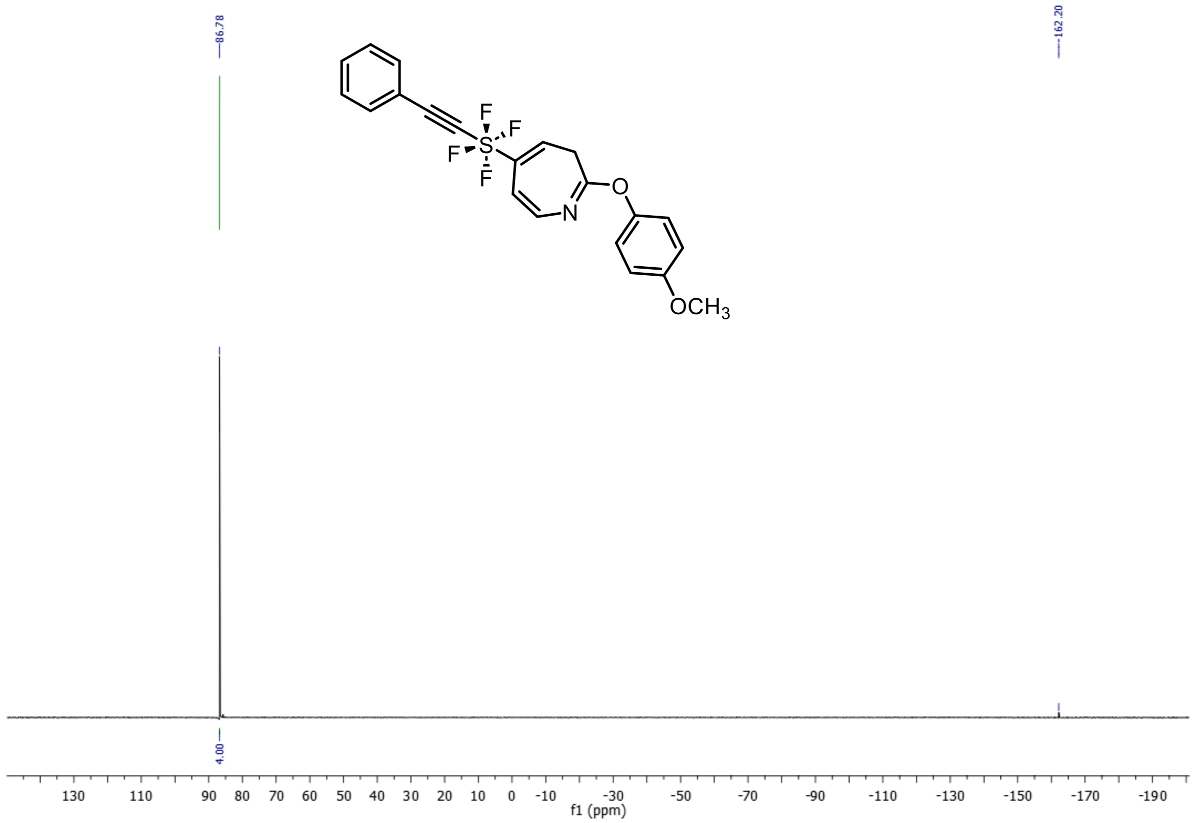

^{1}H NMR (500 MHz, $CDCl_3$) : **8d**

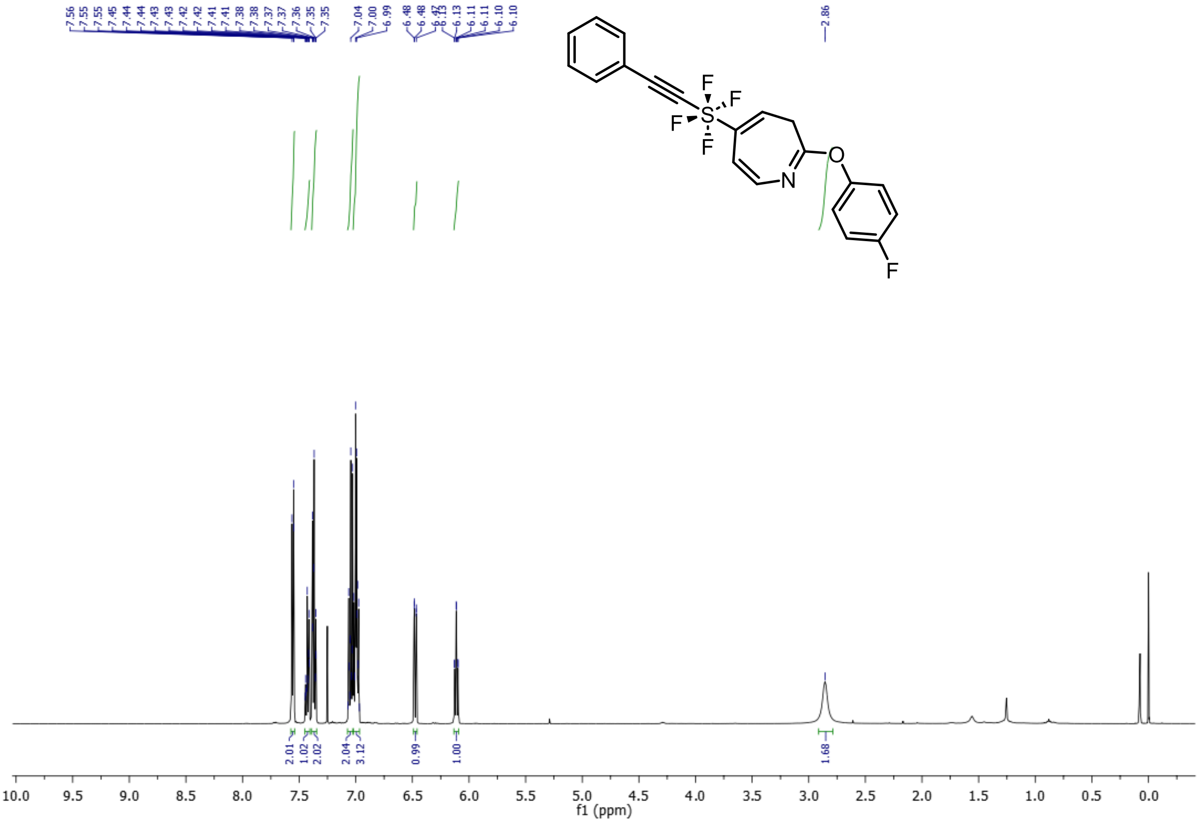

^{13}C **NMR** (126 MHz, $CDCl_3$) : **8d**

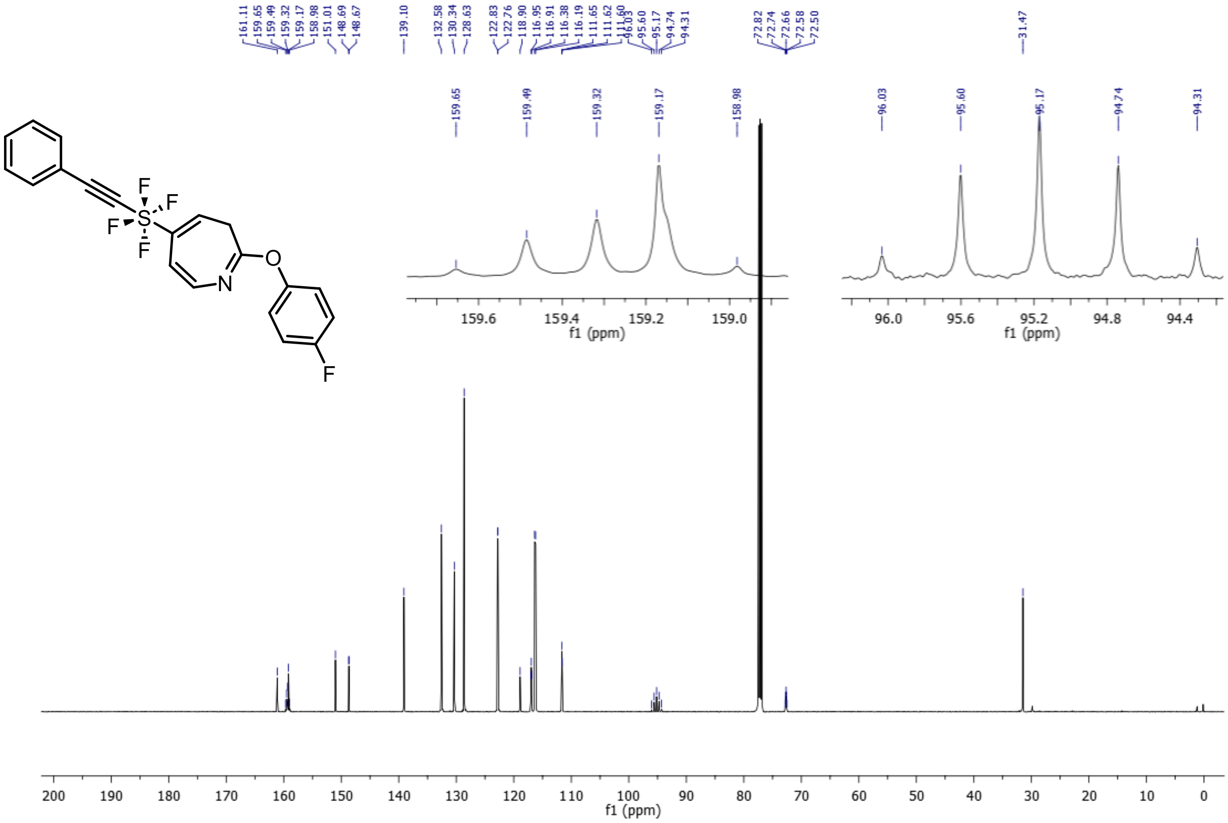

^{19}F **NMR** (282 MHz, $CDCl_3$) : **8d**

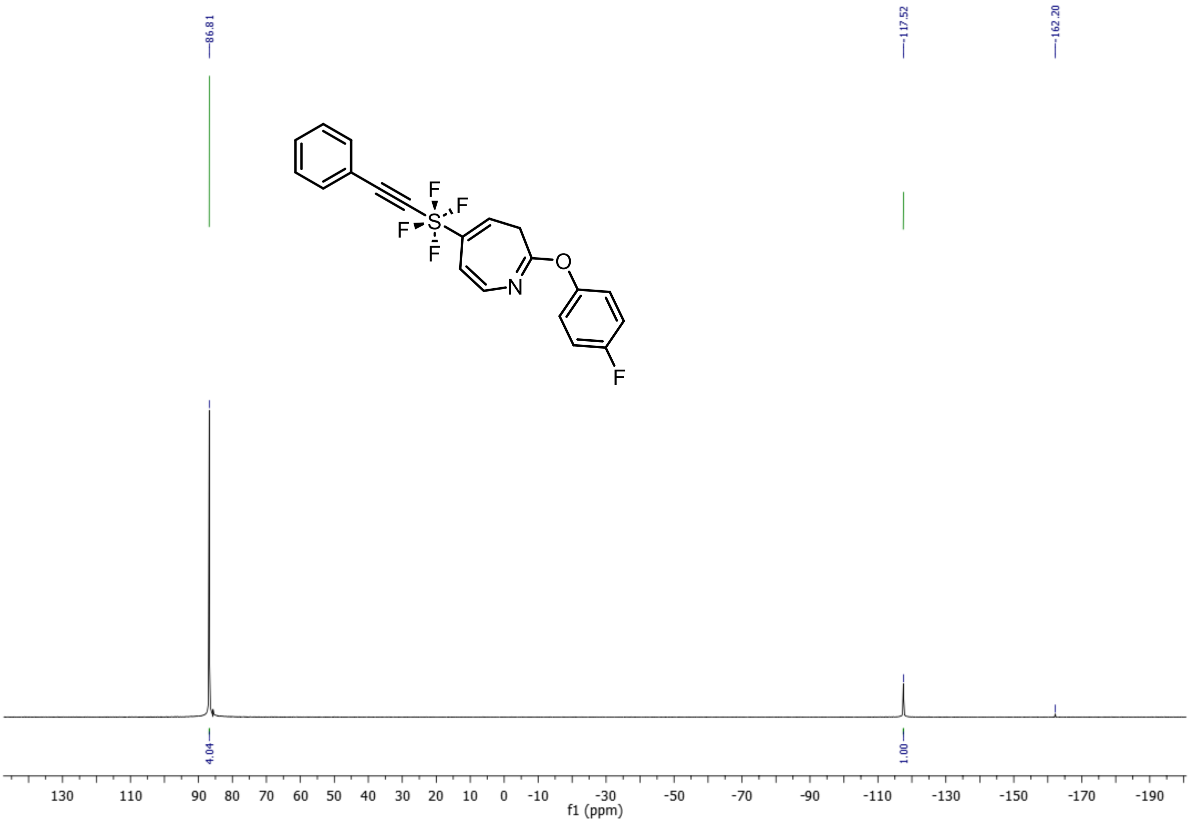

^{1}H NMR (500 MHz, $CDCl_3$) : **8e**

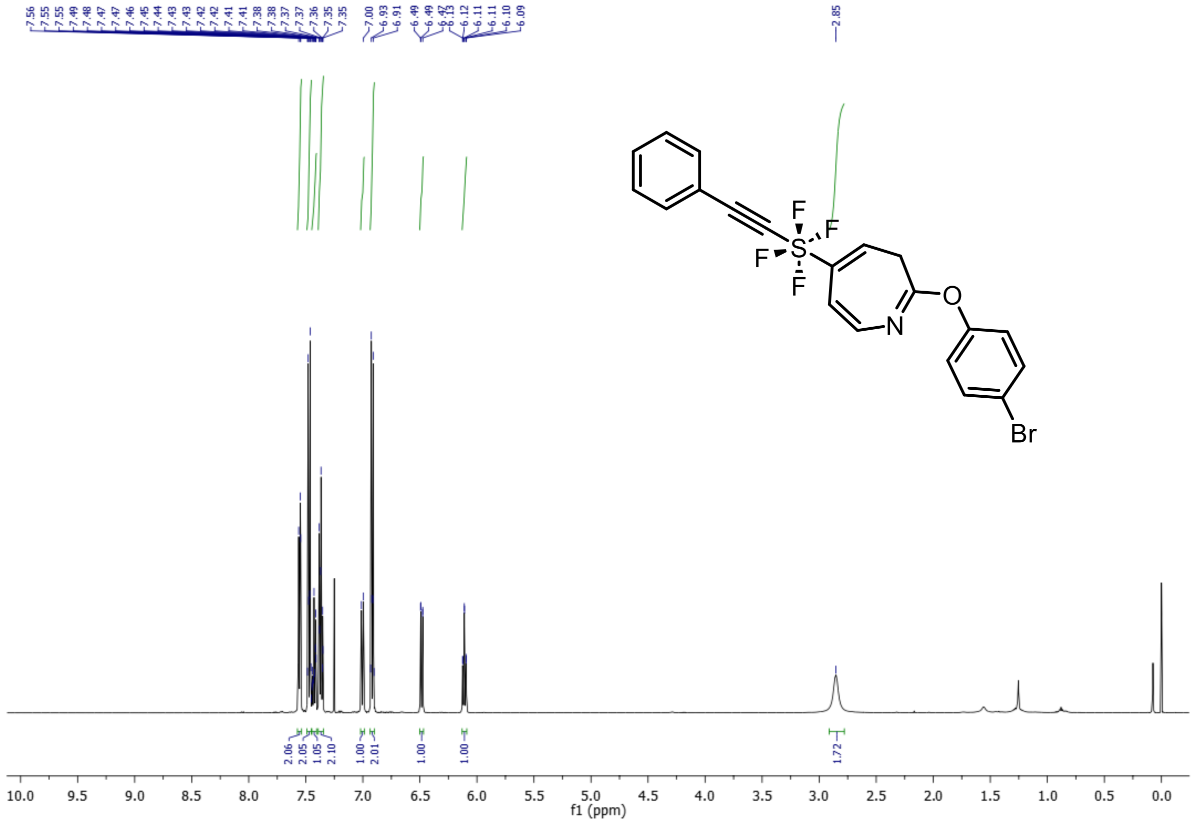

^{13}C NMR (126 MHz, $CDCl_3$) : **8e**

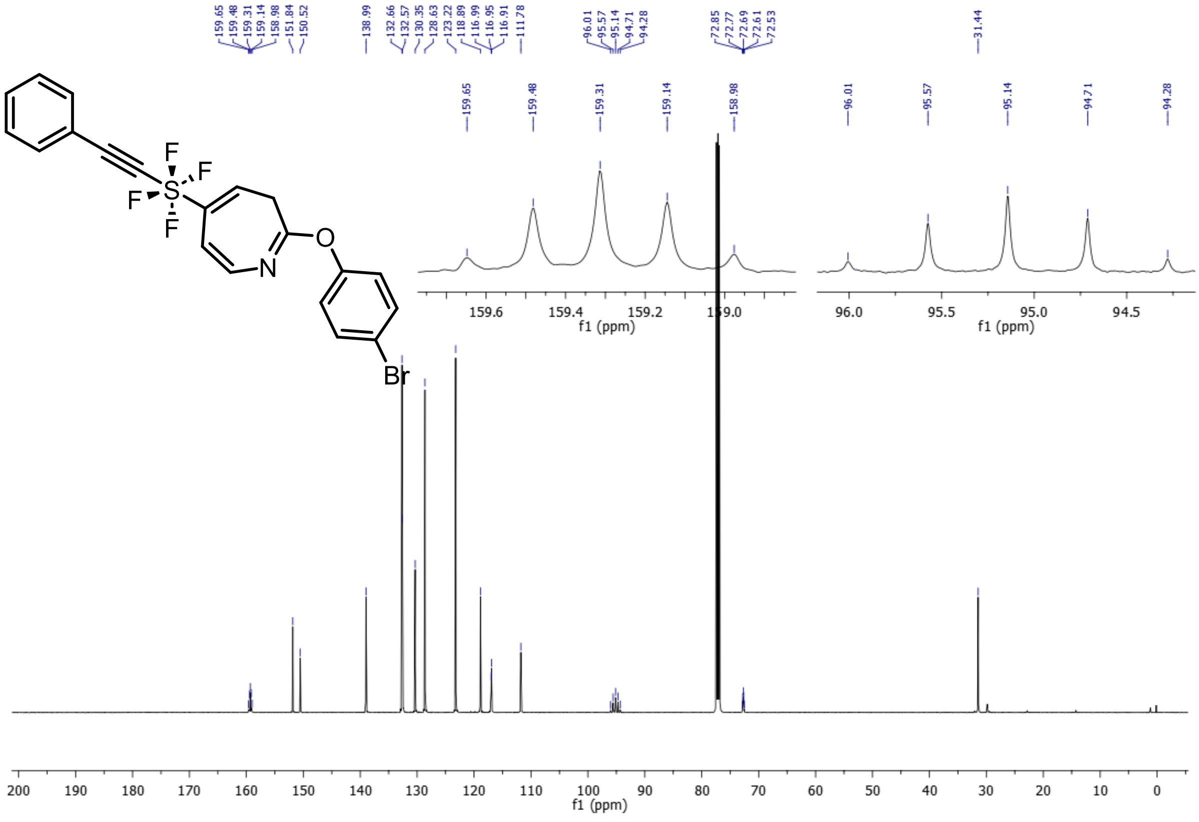

^{19}F NMR (658 MHz, $CDCl_3$) : **8e**

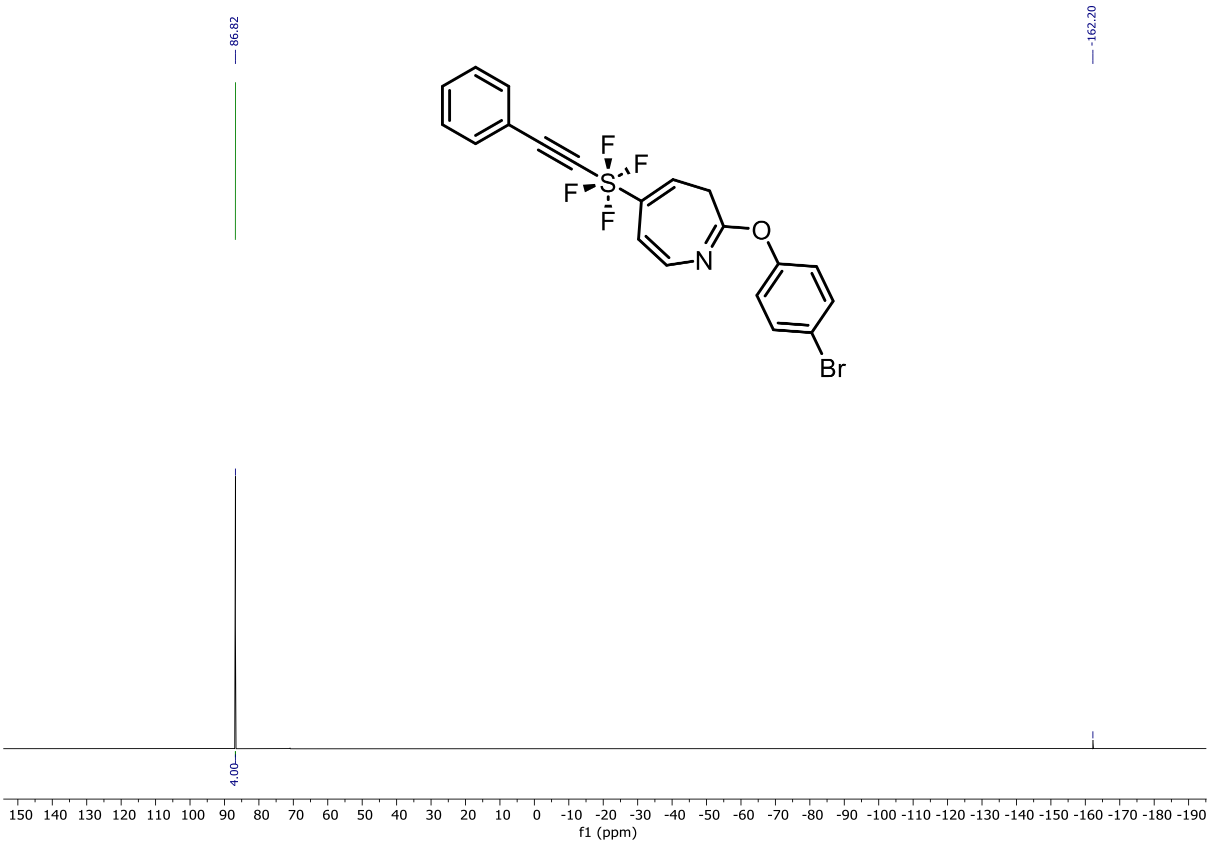

^{1}H NMR (500 MHz, $CDCl_3$) : **8f**

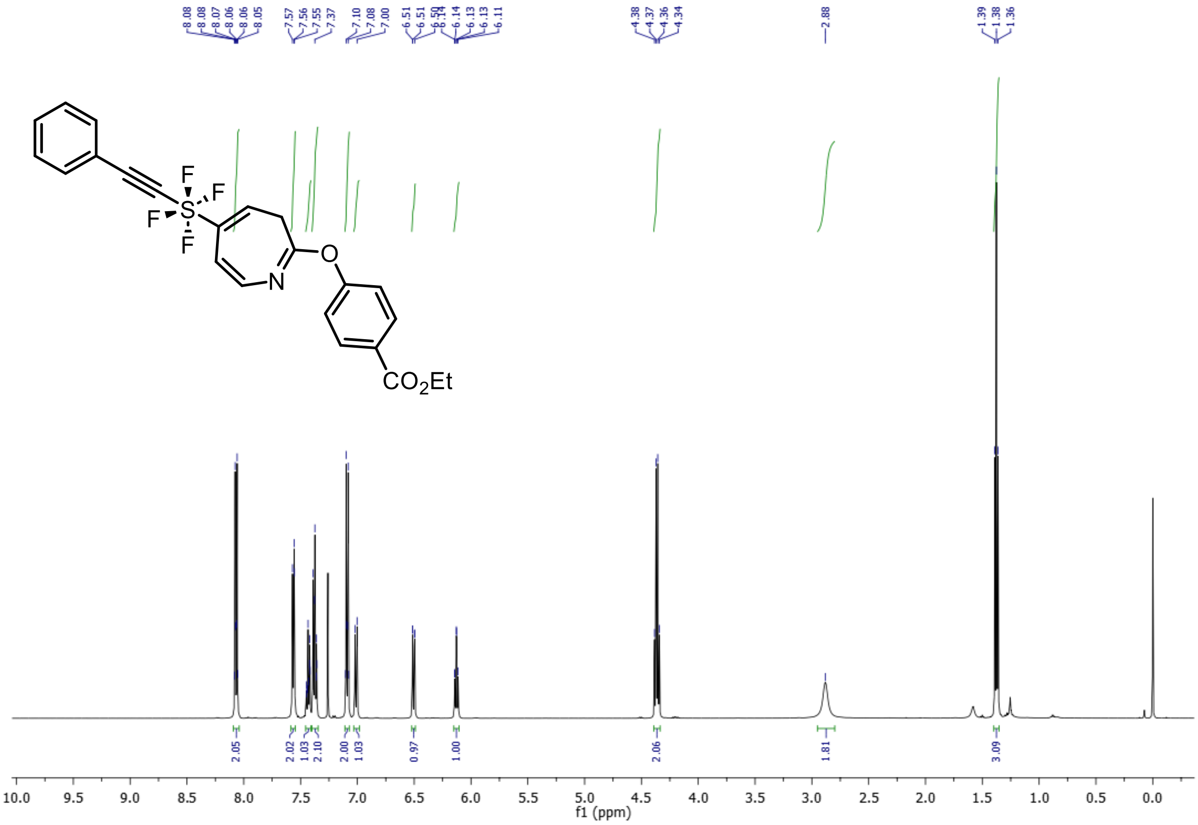

^{13}C NMR (126 MHz, $CDCl_3$) : **8f**

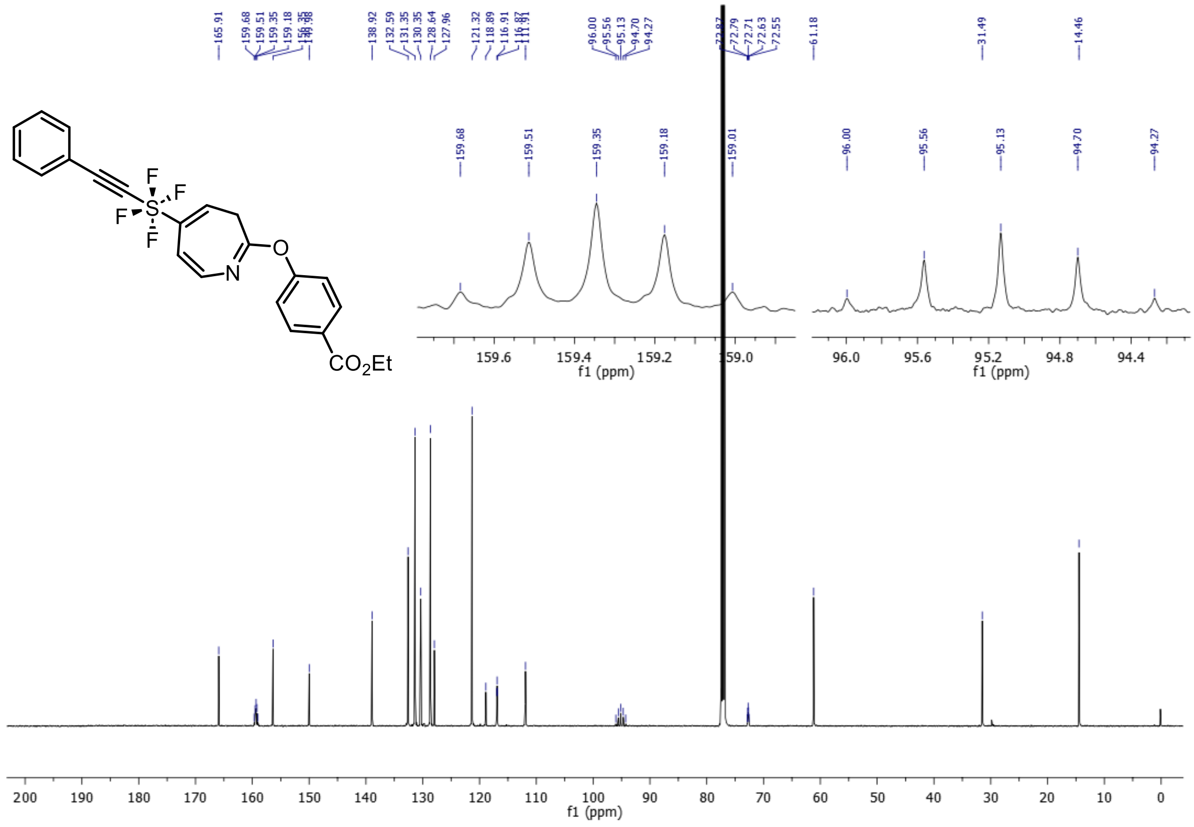

^{19}F NMR (282 MHz, $CDCl_3$) : **8f**

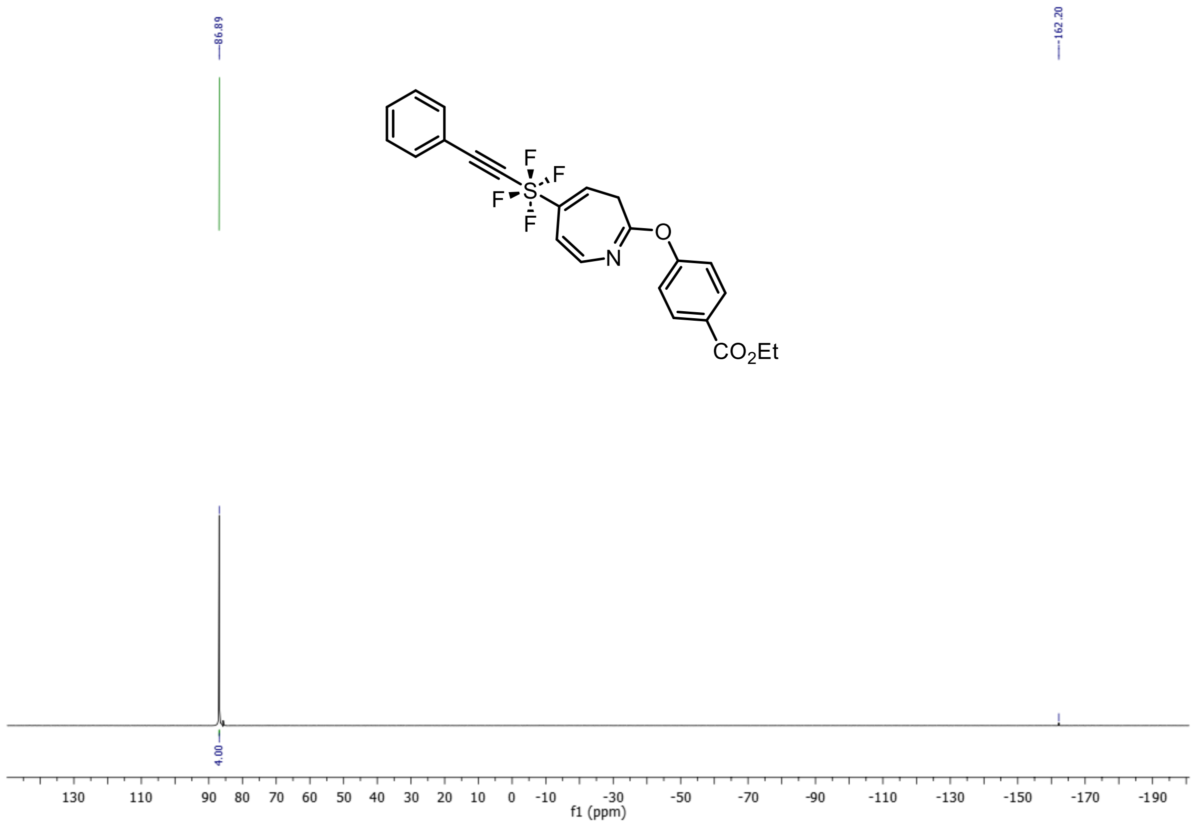

^{1}H NMR (500 MHz, $CDCl_3$) : **8g**

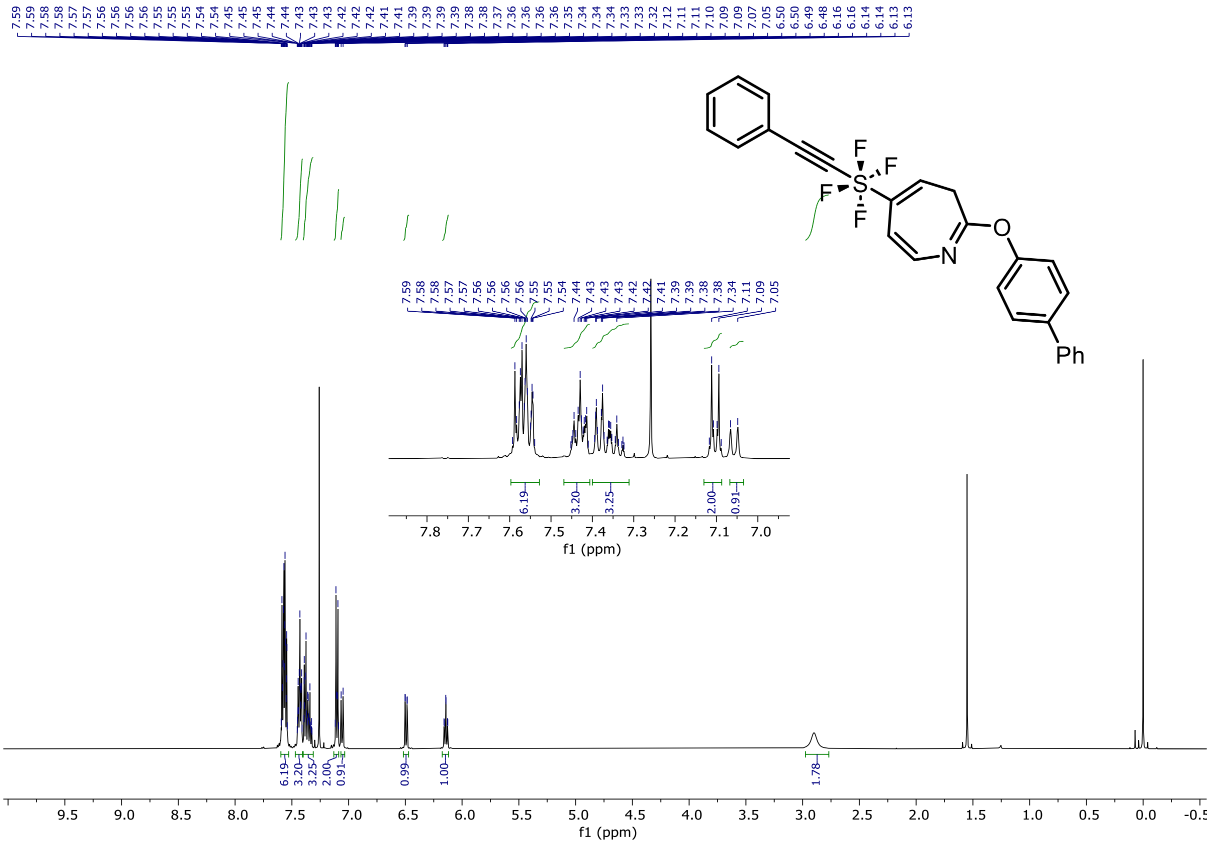

^{13}C NMR (126 MHz, $CDCl_3$) : **8g**

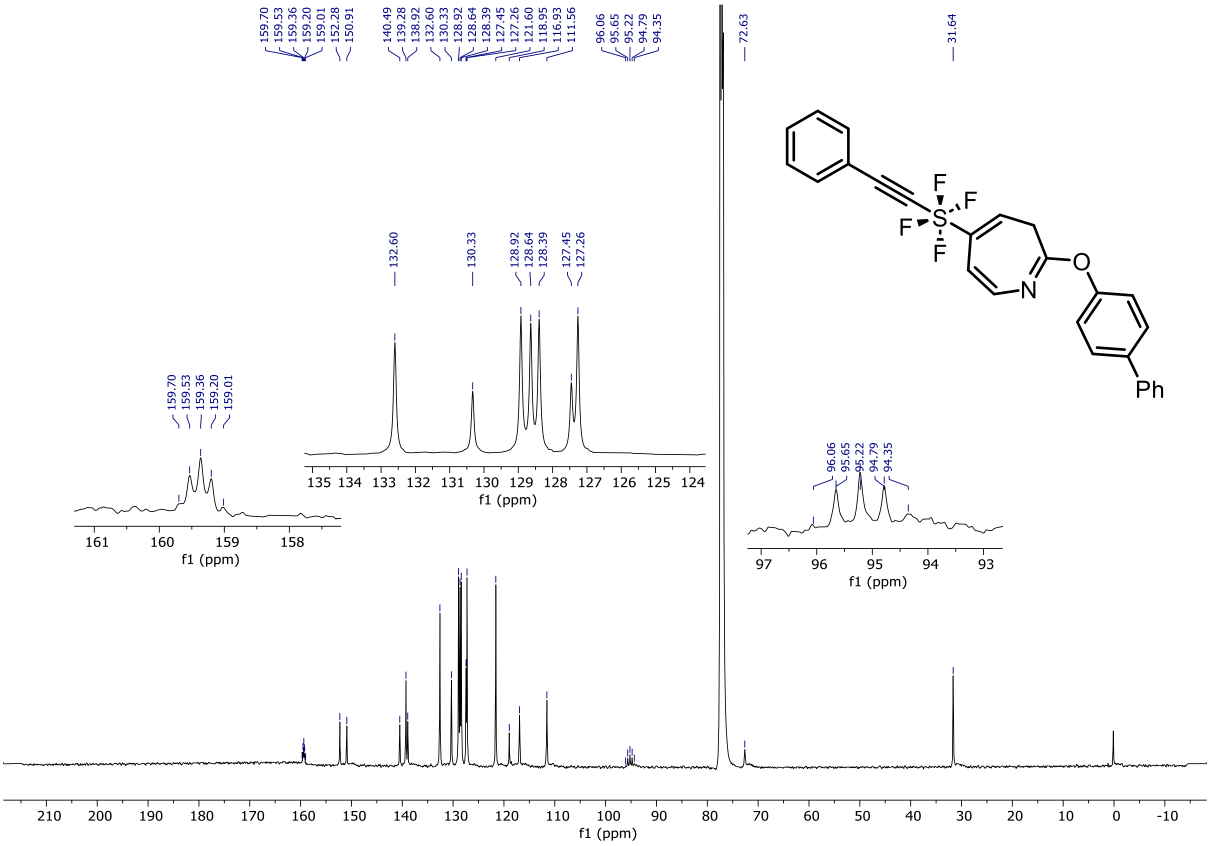

^{19}F NMR (282 MHz, $CDCl_3$) : **8g**

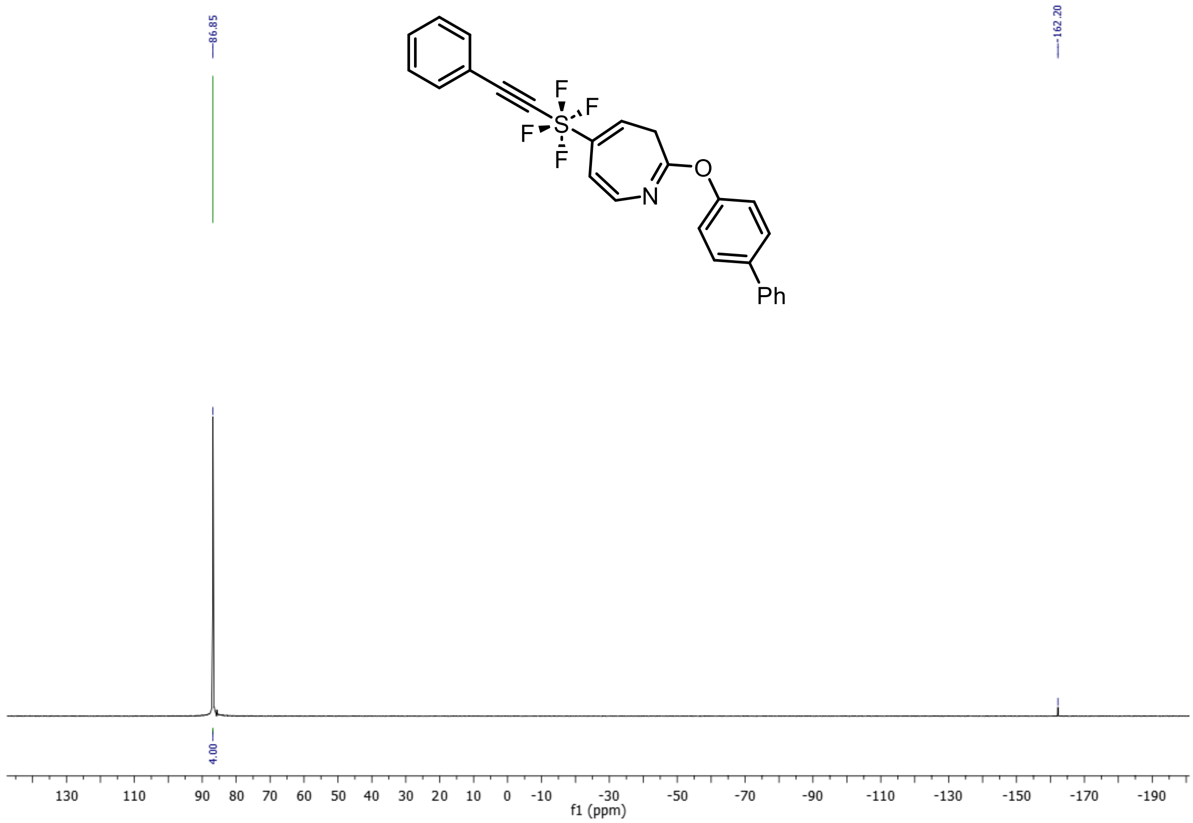

^{1}H NMR (500 MHz, $CDCl_3$) : **9a**

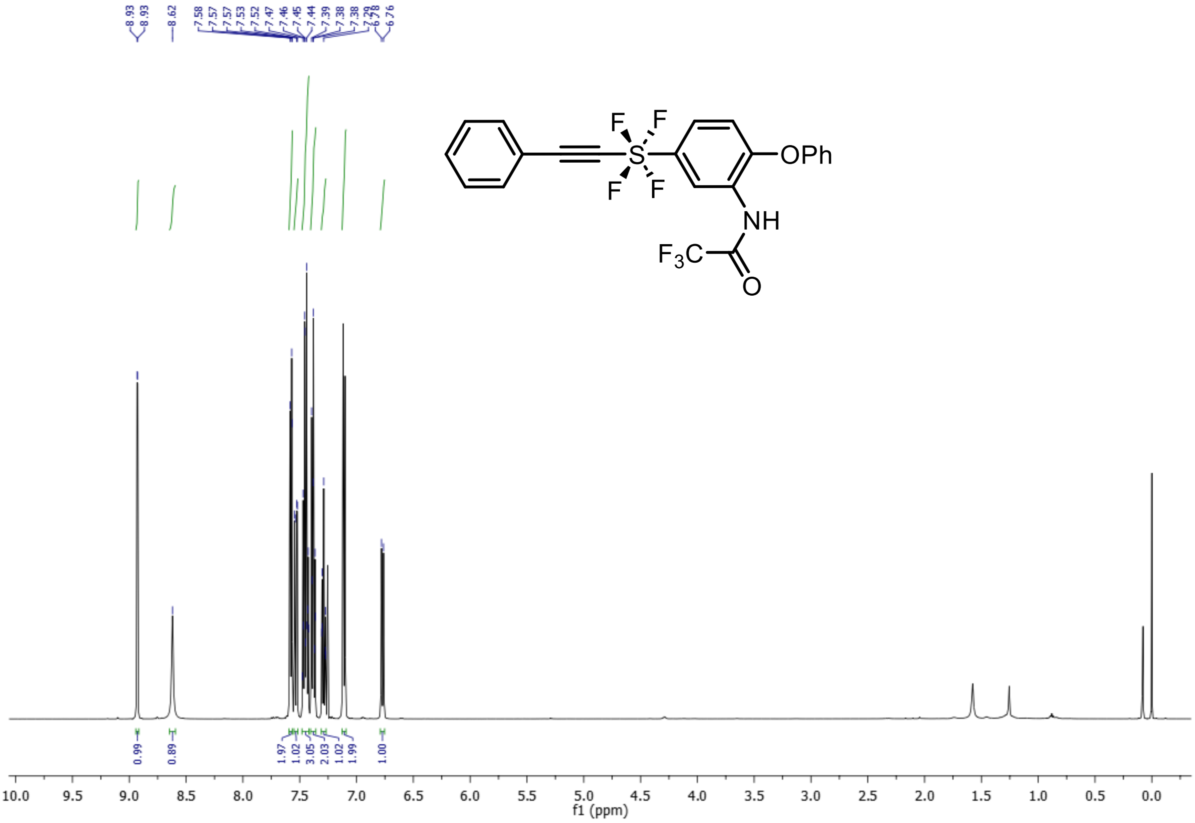

^{13}C NMR (126 MHz, $CDCl_3$) : **9a**

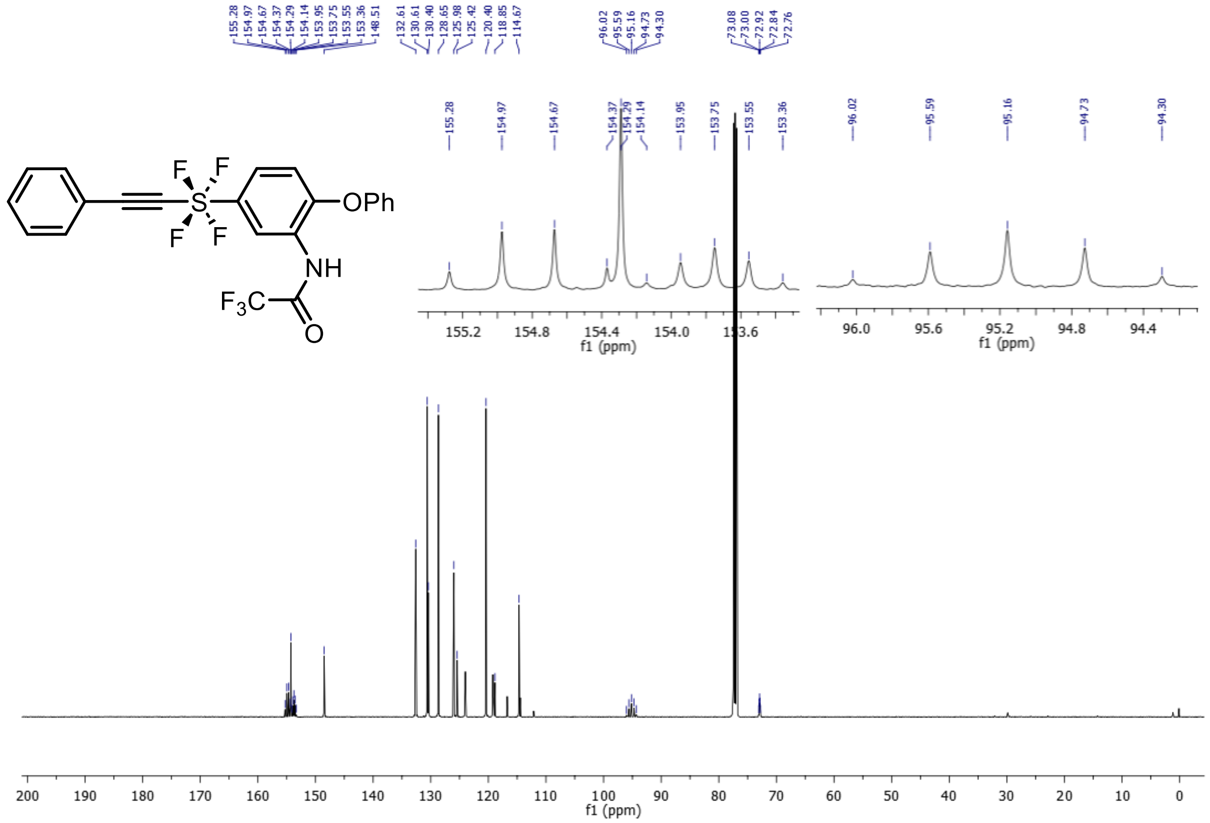

^{19}F NMR (282 MHz, $CDCl_3$) : **9a**

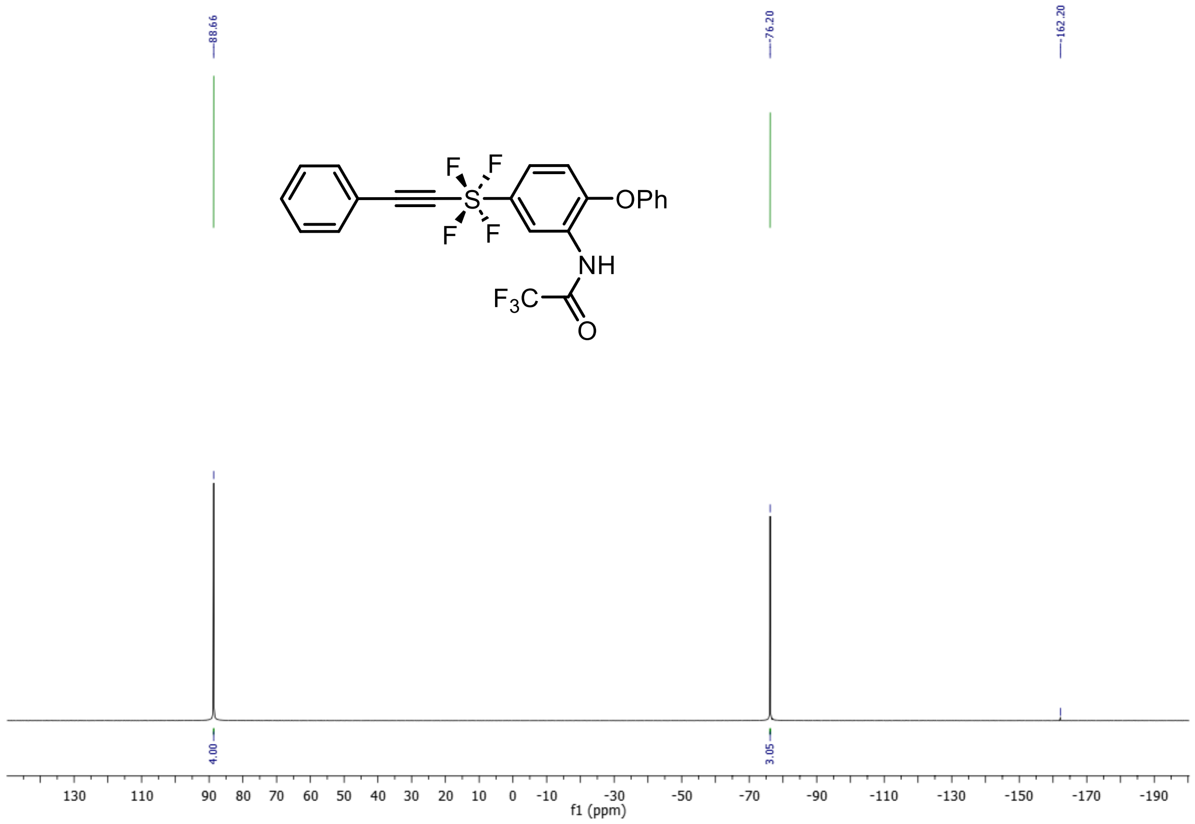

^{1}H NMR (500 MHz, $CDCl_3$) : **9b**

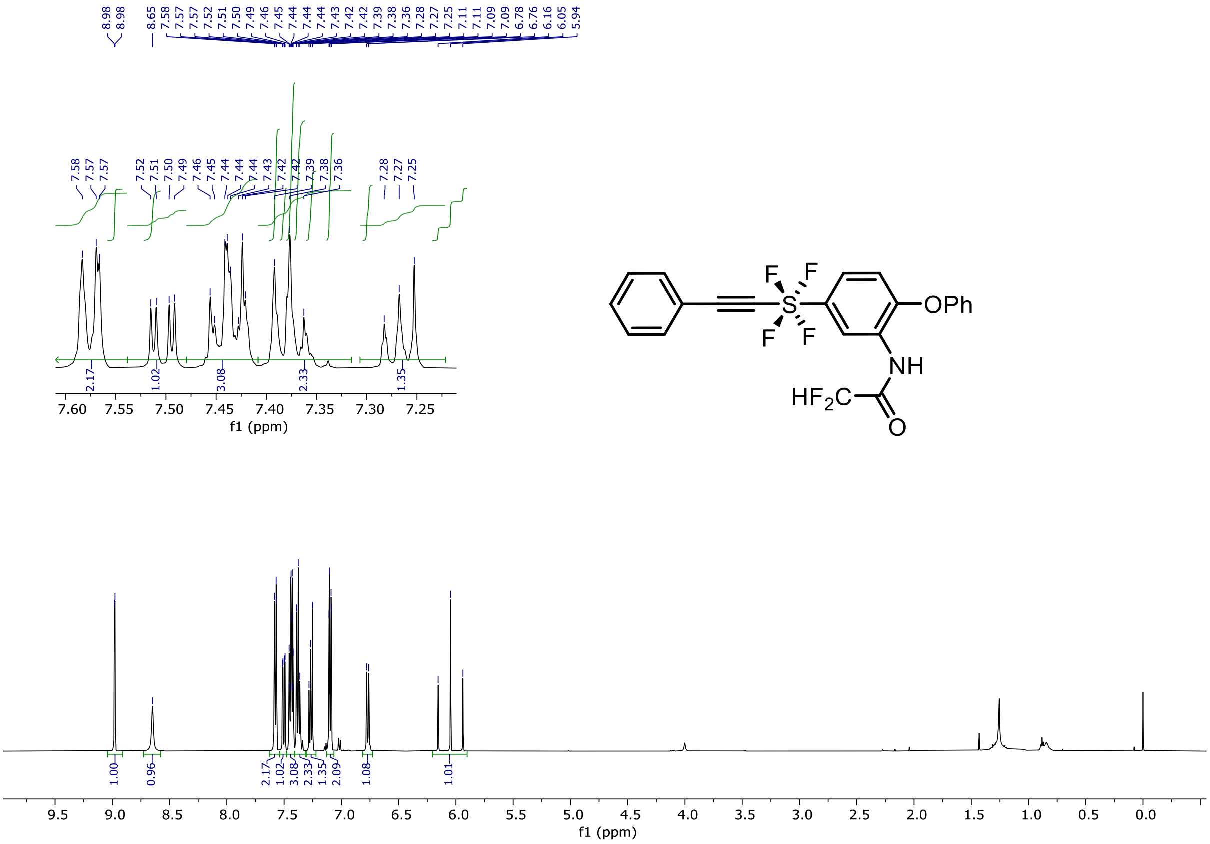

^{13}C NMR (126 MHz, $CDCl_3$) : **9b**

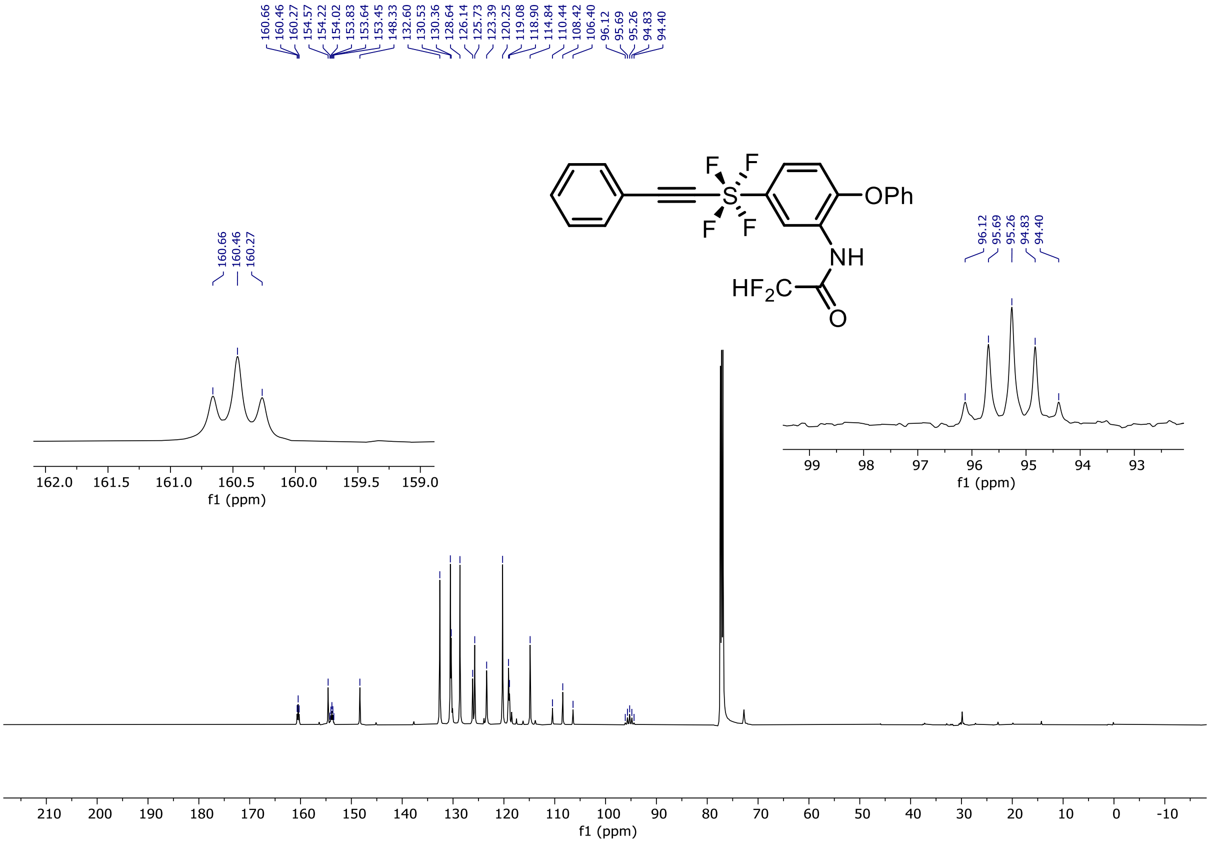

^{19}**F NMR** (658 MHz, $CDCl_3$) : **9b**

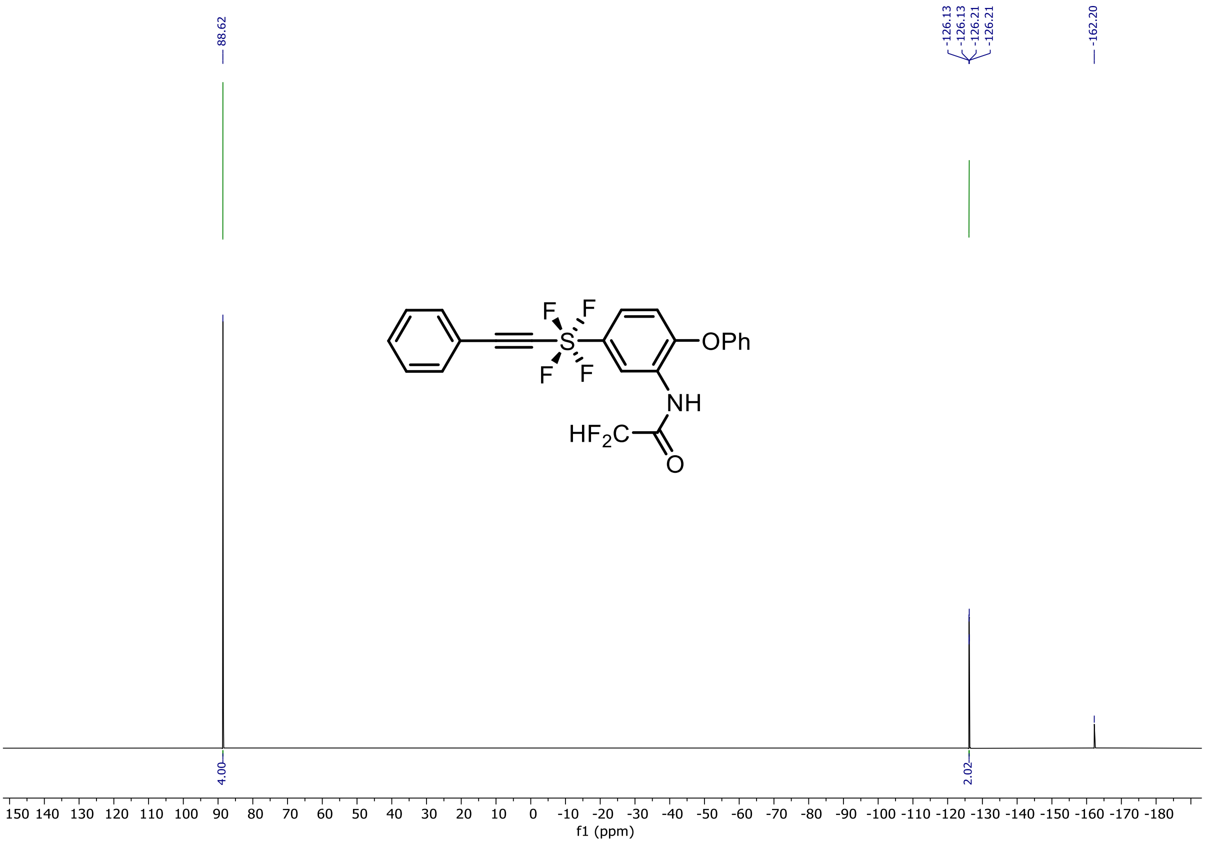

^{1}**H NMR** (500 MHz, $CDCl_3$) : **10**

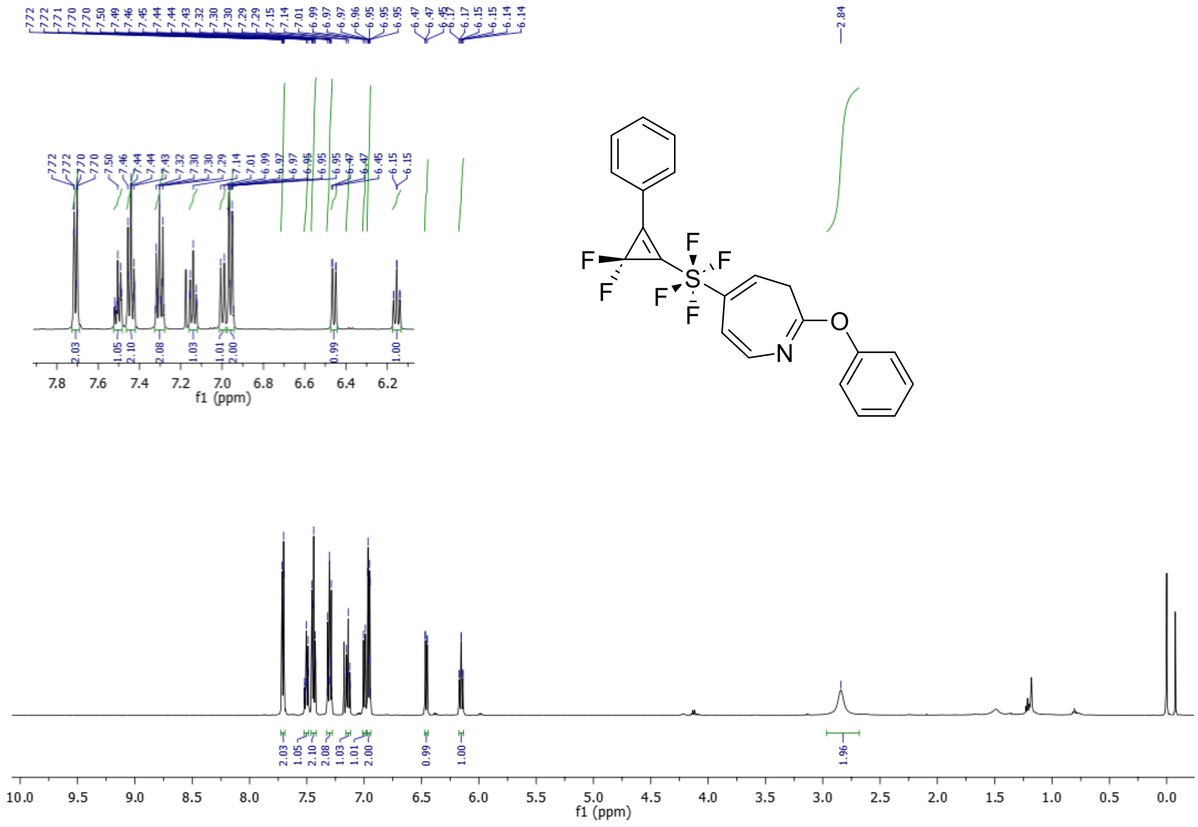

^{13}C NMR (126 MHz, $CDCl_3$) : **10**

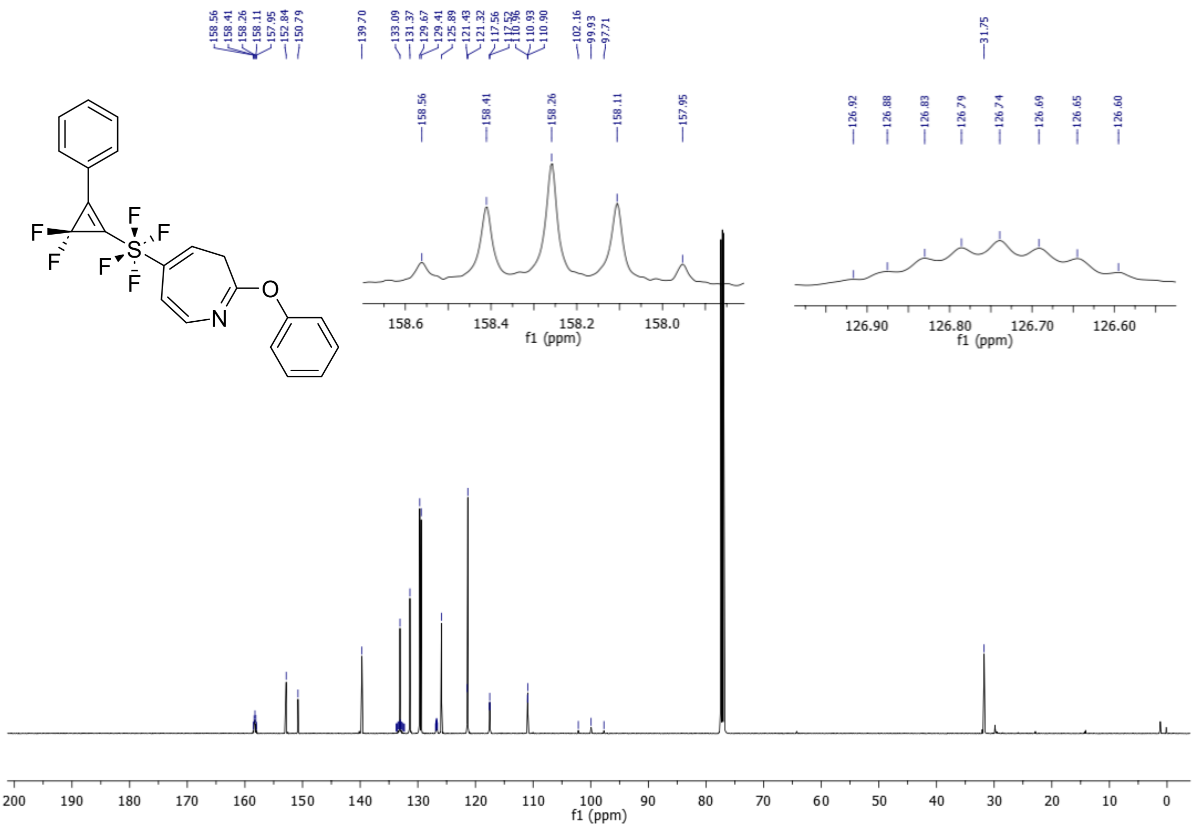

^{19}F NMR (282 MHz, $CDCl_3$) : **10**

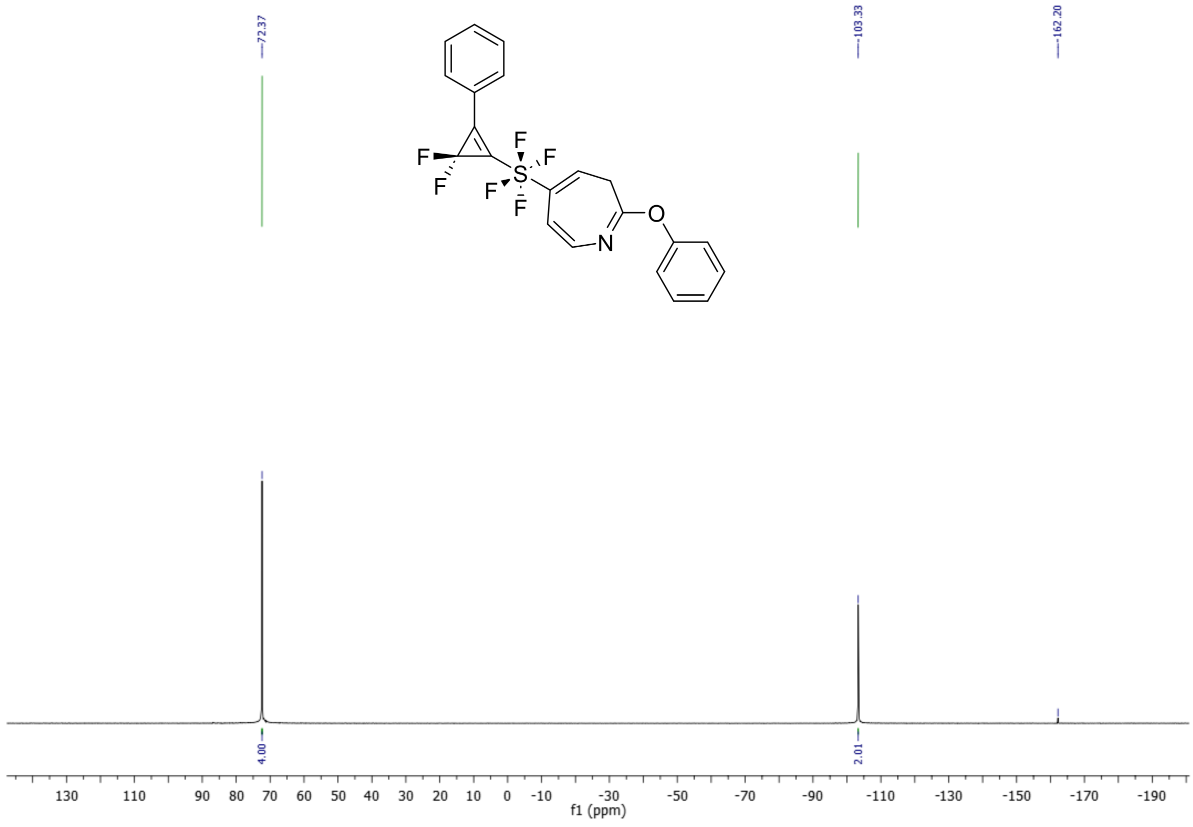

^{1}H NMR (500 MHz, $CDCl_3$) : **11**

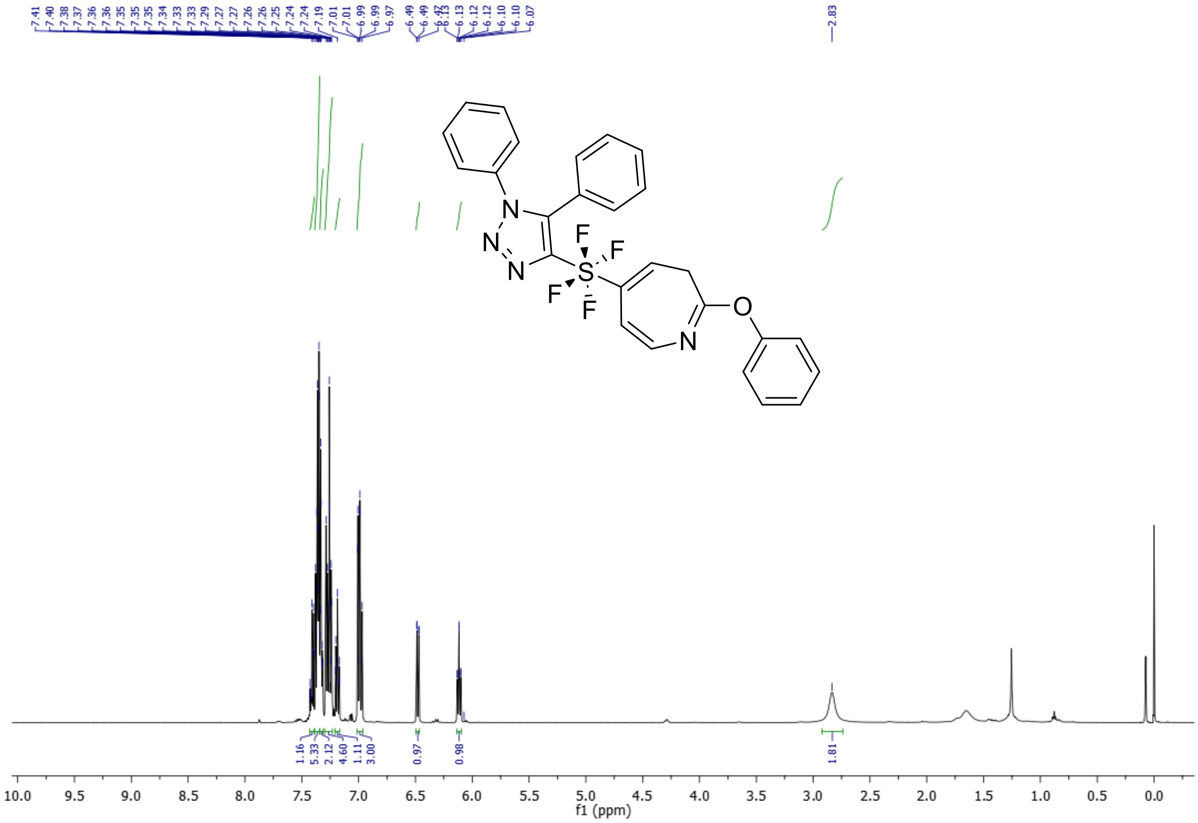

^{13}C NMR (126 MHz, $CDCl_3$) : **11**

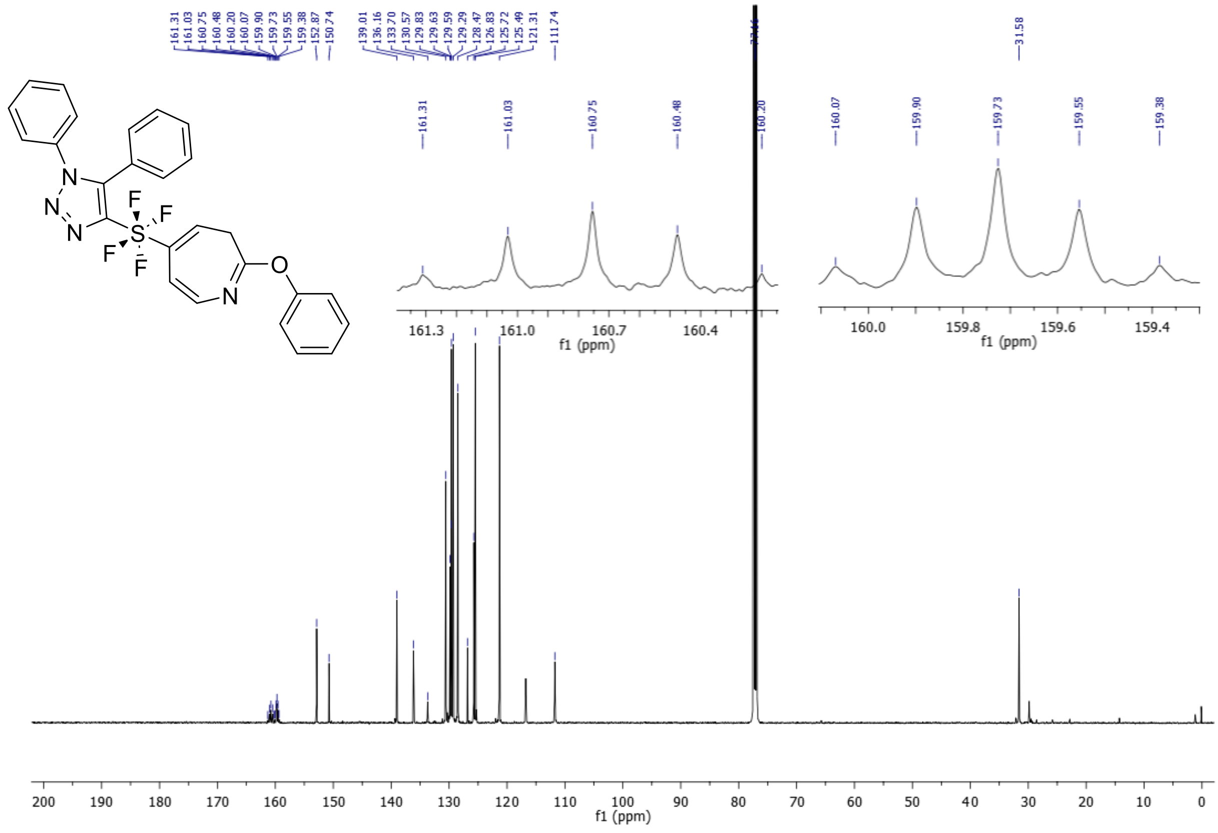

^{19}F NMR (282 MHz, $CDCl_3$) : **11**

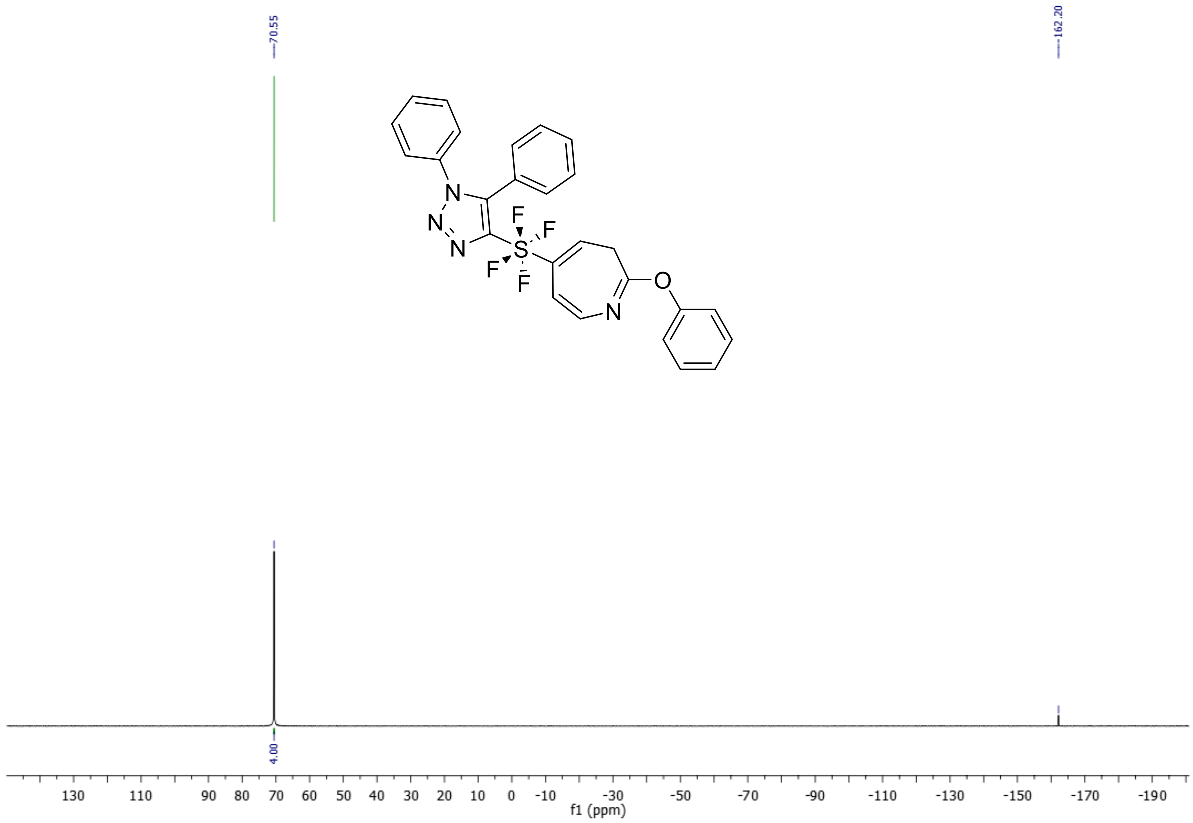

^{1}H NMR (500 MHz, $CDCl_3$) : **13**

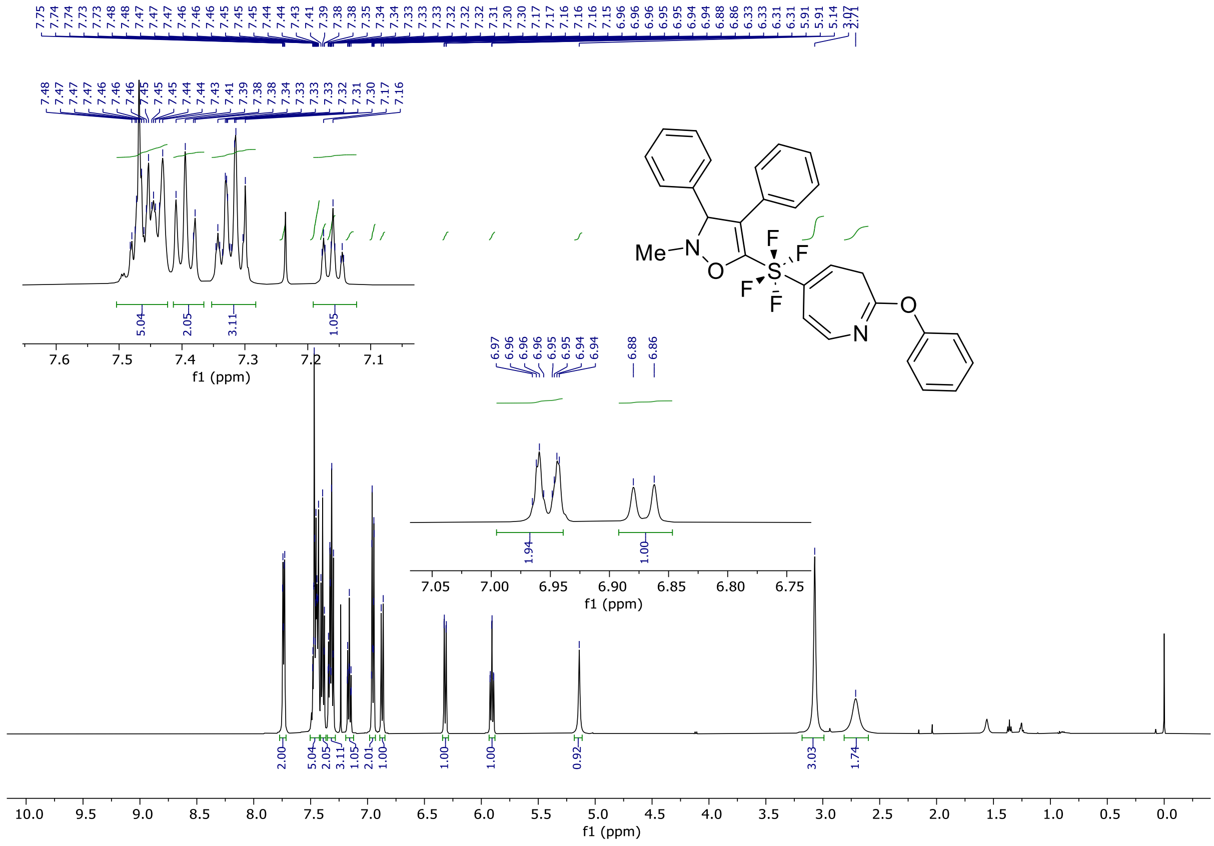

^{13}C NMR (126 MHz, $CDCl_3$) : **13**

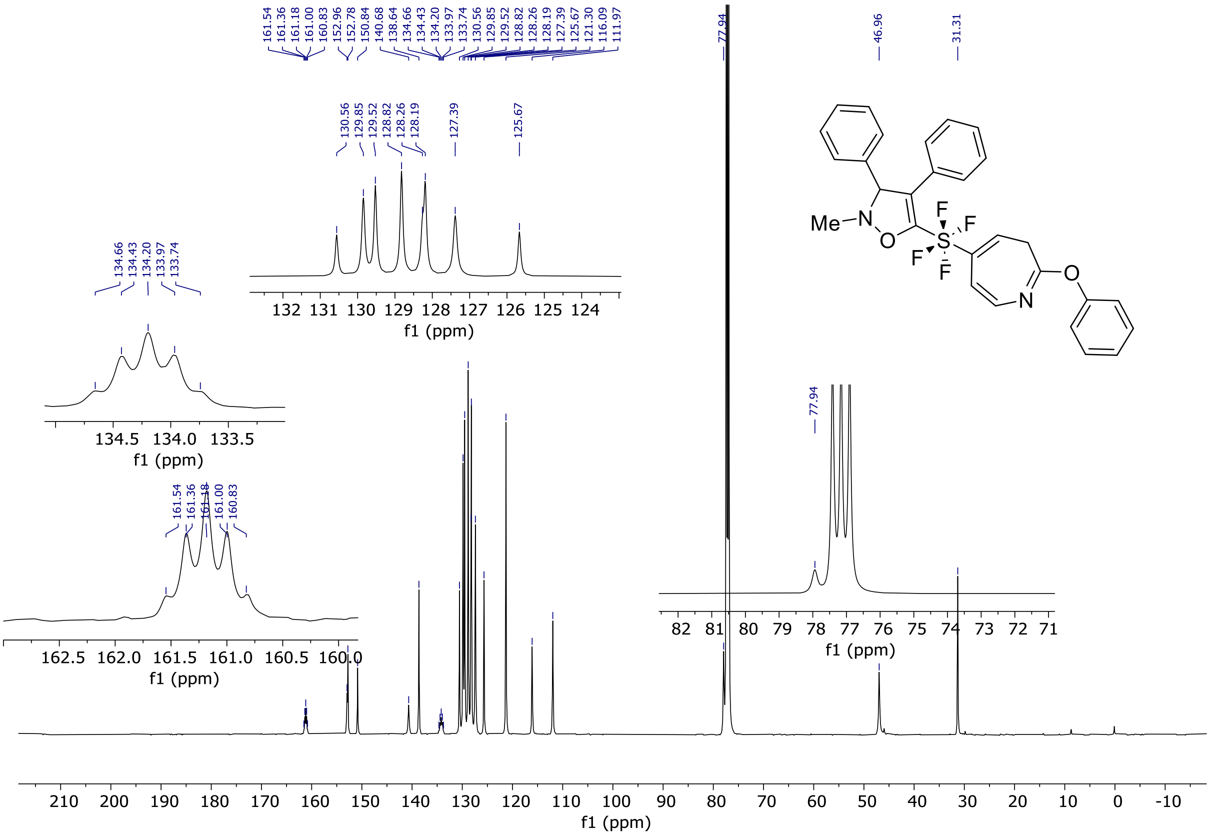

^{19}F NMR (658 MHz, $CDCl_3$) : **13**

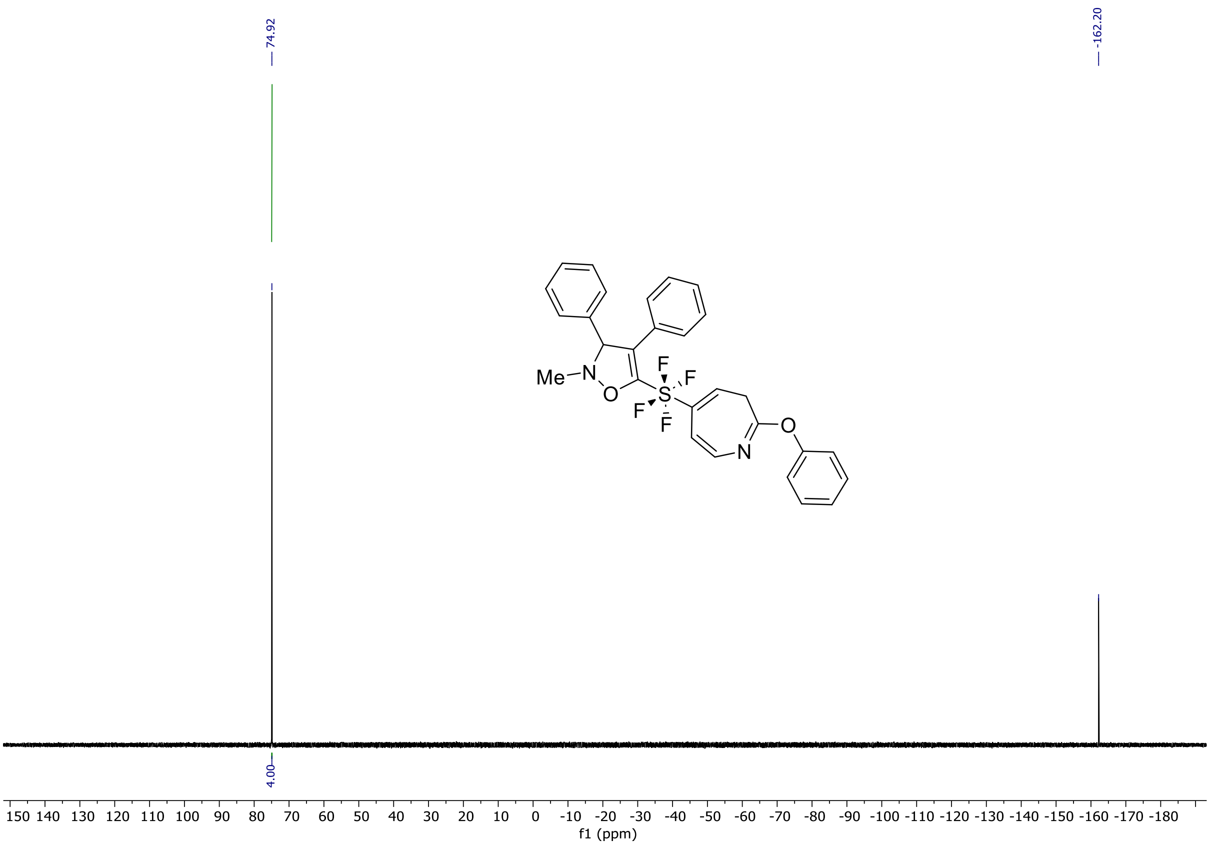

Supplement: SC-017-D5SC08177G-s001 [file SC-017-D5SC08177G-s001.pdf]
